# Highly abundant core taxa in the blow within and across captive bottlenose dolphins provide evidence for a temporally stable airway microbiota

**DOI:** 10.1186/s12866-020-02076-z

**Published:** 2021-01-09

**Authors:** Catharina Vendl, Tiffanie Nelson, Belinda Ferrari, Torsten Thomas, Tracey Rogers

**Affiliations:** 1 Evolution and Ecology Research Centre, School of Biological, Earth and Environmental Sciences, University of New South Wales, Sydney, NSW 2052 Australia; 2 Queensland Facility for Advanced Bioinformatics, Griffith University, Gold Coast, Southport, QLD 4215 Australia; 3 School of Biotechnology and Biomolecular Sciences, University of New South Wales, Sydney, NSW 2052 Australia; 4 Centre for Marine Science and Innovation, School of Biological, Earth and Environmental Sciences, University of New South Wales, Sydney, NSW 2052 Australia

**Keywords:** Dolphin, Cetacean, Respiratory health, Core microbiota, Antimicrobial treatment

## Abstract

**Background:**

The analysis of blow microbiota has been proposed as a biomarker for respiratory health analysis in cetaceans. Yet, we lack crucial knowledge on the long-term stability of the blow microbiota and its potential changes during disease. Research in humans and mice have provided evidence that respiratory disease is accompanied by a shift in microbial communities of the airways. We investigate here the stability of the community composition of the blow microbiota for 13 captive bottlenose dolphins over eight months including both sick and healthy individuals. We used barcoded tag sequencing of the bacterial 16S rRNA gene. Four of the dolphins experienced distinct medical conditions and received systemic antimicrobial treatment during the study.

**Results:**

We showed that each dolphin harboured a unique community of zero-radius operational taxonomic units (zOTUs) that was present throughout the entire sampling period (‘intra-core’). Although for most dolphins there was significant variation over time, overall the intra-core accounted for an average of 73% of relative abundance of the blow microbiota. In addition, the dolphins shared between 8 and 66 zOTUs on any of the sampling occasions (‘inter-core’), accounting for a relative abundance between 17 and 41% of any dolphin’s airway microbiota. The majority of the intra-core and all of the inter-core zOTUs in this study are commonly found in captive and free-ranging dolphins and have previously been reported from several different body sites. While we did not find a clear effect of microbial treatment on blow microbiota, age and sex of the dolphins did have such an effect.

**Conclusions:**

The airways of dolphins were colonized by an individual intra-core ‘signature’ that varied in abundance relative to more temporary bacteria. We speculate that the intra-core bacteria interact with the immune response of the respiratory tract and support its function. This study provides the first evidence of individual-specific airway microbiota in cetaceans that is stable over eight months.

**Supplementary Information:**

The online version contains supplementary material available at 10.1186/s12866-020-02076-z.

## Background

Dolphins harbour rich and diverse bacterial communities in the exhaled breath condensate (blow) that they forcefully expel from their airways through their blowhole when at the sea surface [1–6]. The airway microbiota of dolphins are distinct to those of their other body sites, their surrounding seawater [1, 6], air, their prey (fish and squid) and the hand and nose of their human carers in the case of captive dolphins [2]. The bacterial communities in the airways are unique to each individual dolphin. The changes the microbiota of an individual dolphin undergo over relatively short periods of time (two months) are minor compared to the differences between individuals [4].

The analysis of blow microbiota has been considered a promising tool for the health assessment of cetaceans [4–8]. However, to establish blow microbiota as a viable biomarker, crucial knowledge is still lacking: Is the healthy airway microbiota stable over a period longer than two months? Are certain highly prevalent ‘core’ taxa present that represent a healthy and stable microbiota? Does the airway microbiota of cetaceans reflect the physiological state and the health of its host?

Research on humans and mice has indicated a correlation between airway microbiota and the physical state of the host. Germ-free mice are more susceptible to respiratory infections than conspecifics that carry microbial communities in their airways [9] highlighting the role of the microbiota in disease protection and prevention. Additionally, de Steenhuijsen Piters et al. [10] and Esposito and Principi [11] suggested the human respiratory microbiota to contribute to health regulation. Nevertheless, the temporal stability of the human airway microbiota is under debate [12]. Dickson and Huffnagle [13] and Venkataraman et al. [14] postulated that the bacterial communities in airways of healthy humans are relatively dynamic and transient with few site-specific taxa. In addition, Charlson et al. [15] did not find any shared (core) bacterial sequences in human airways. The same was observed in mice, as Dickson et al. [16] did not find any bacteria common to all mice in their study despite the mice being genetically identical.

Factors, like microbial immigration, elimination and growth rates, as well as the concentration of bacteria in the air determine the community composition of respiratory microbiota in humans [13]. The majority of bacteria in the human airways derives from the oral cavity and reaches the airways via microaspiration, as digestive and respiratory tract intersect [14, 17–19]. The separation of airways and digestive tract in cetaceans [20–22] makes an overlap of microbiota from these two body regions unlikely. And indeed, Bik et al. [1] showed that the blow microbiota in dolphins are very different from those in the oral cavity. The blow microbiota might therefore be a better bacterial representation of the airways in cetaceans than the airway microbiota in humans. On the other hand, due to their marine lifestyle cetaceans developed a more forceful breathing mechanism than humans, as they exhale up to 90% of their lung volume in one breath [23–25] compared to only 50% in a physically active human [26, 27]. This might cause a higher turnover rate of bacteria in cetacean airways, potentially making them even more transient than in humans.

A state of disease or even a slight change of immunological capacity of the host [16, 19] can change the diversity and overall microbial composition in the airways [28, 29]. Yet, very little is known about the specific functions airway bacteria may play and how they interact with their host [30]. Furthermore, systemic antibiotic treatment alters [31–33] and suppresses airway microbiota [16] and potentially disturbs the relationship between microbiota and host [34]. Shade and Handelsman [35], Hernandez et al. [36] and Apprill et al. [7] hypothesized that highly prevalent and abundant bacterial species, defined as the core, represent a stable and healthy microbial community as these bacteria interact and benefit the immune system of the host.

Here, to test the temporal stability of dolphin airway microbiota, we characterise the blow microbiota of 13 captive bottlenose dolphins sampled at monthly to bi-monthly intervals over an eight-month period. We examine the core bacteria within and across dolphins, as well as the influence of environmental factors, the impact of disease and consequential systemic antimicrobial treatment. We hypothesize that the airways of each dolphin harbour a unique bacterial community throughout the sampling period that maintains a certain level of temporal stability as expressed in a certain number and relative abundance of core bacteria. Furthermore, we predict a significant influence of the antimicrobial treatment and potentially of the compromised health on the microbial blow communities of the dolphins. We seek to contribute new knowledge about the blow microbiota in dolphins to facilitate non-invasive respiratory health assessment in cetaceans in the future.

## Results

### Sample collection

**Fig. 1:**
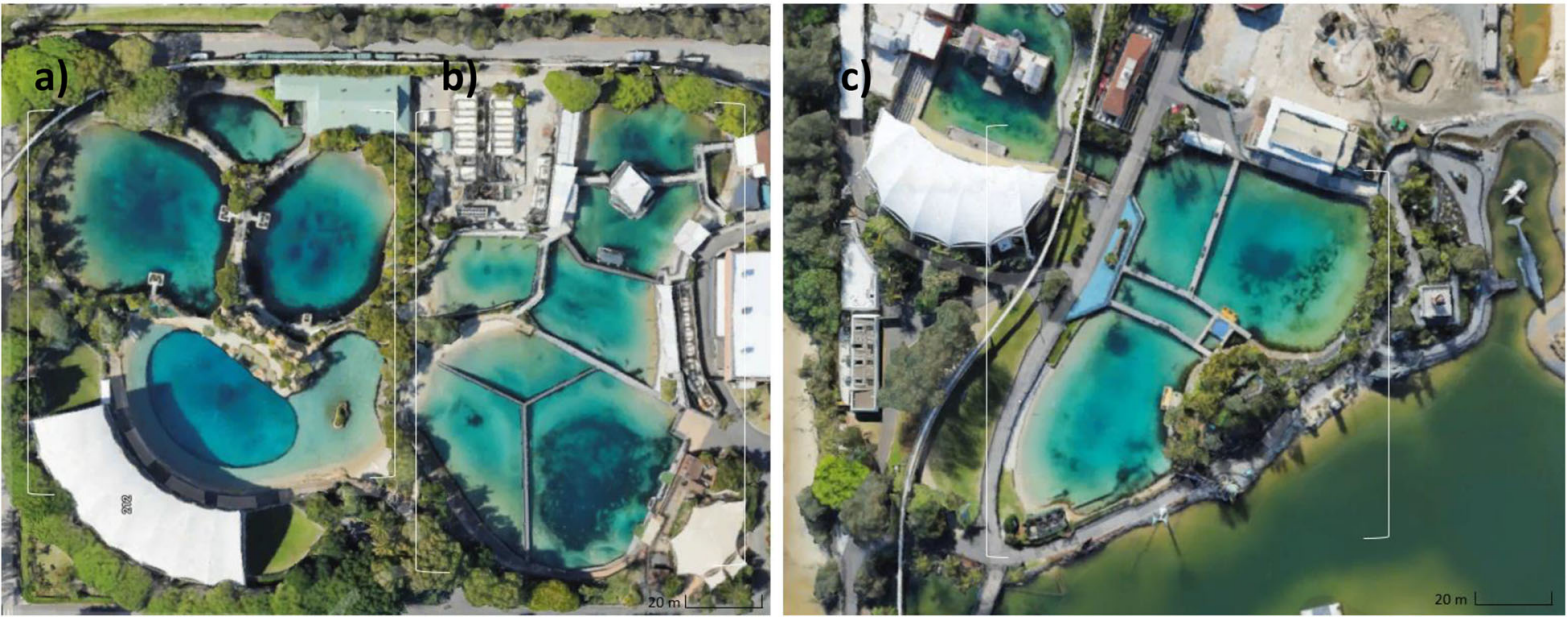
Aerial view [37] of the three pool systems. **a** Dolphin Beach, **b** Dolphin Bay, **c** Endeavour) at Sea World where sampled dolphins were kept in. Even when partition gates between ‘subpools’ were closed, water flow between ‘subpools’ still was still retained. The image was obtained from Google Maps [37]

The dolphin trainers of Sea World Marine Park Gold Coast, Australia, collected samples of exhaled breath condensate (‘blow’) of 13 captive bottlenose dolphins (*Tursiops truncatus*) over a period of eight months from April to December 2017. Table 1 presents the sampling schedule listing a total of 86 blow samples, while Table 2 provides additional information on the dolphins, including sex, age and the pool system the animals were kept in. The date of birth was unknown for three dolphins, as they were rescued from the wild. Nine out of the 13 dolphins were in good health conditions throughout the sampling period. Four dolphins (‘Gemma’, ‘Howie’, ‘Nudgee’, ‘Stella’) suffered from a medical condition that required antimicrobial treatment at least once while sampling was performed (Table 2). Table 3 includes their medical condition as well as type and date of antimicrobial treatment. For the nine healthy dolphins, six blow samples each, collected in weeks 2, 6, 11, 19, 28 and 37, were included into the data analysis (Table 1). For those dolphins that received antimicrobial treatment, we applied a more frequent sampling schedule to be able to closely monitor potential changes of their blow microbiota in response to the treatment (Table 1). Gaps in the extended sampling schedule of the sick dolphins were due to logistical issues at Sea World. Each sampling event was accompanied by one water sample per pool system. Sea World had three large dolphin-holding pool systems (‘Dolphin Bay’, ‘Dolphin Beach’, ‘Endeavour’) and a separate quarantine pool (‘QVC’) (Fig. 1).

**Table 1:**
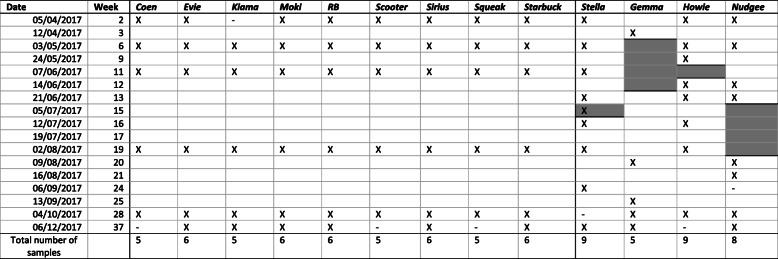
Sample collection dates

‘X‘indicates that a blow samples was collected and included in the data analysis. ‘-‘indicates that a blow sample was collected, but was discarded due to its number of reads being below 6000. A vertical bold line between the columns of ‘Starbuck‘and ‘Stella‘indicates the separation between healthy/ untreated dolphins to the left from the treated dolphins to the right. The time period of antimicrobial treatment in ‘Stella‘, ‘Gemma‘, ‘Howie‘and ‘Nudgee‘is indicated by the bold horizontal cell borders and the shaded cells in the associated columns

**Table 2:** Sex, age, pool system the dolphins were kept in and antimicrobial (AB) treatment (Y=Yes, N=No)

Dolphin ID	Sex	Year of birth	Age level	Pool system	AB treatment
Coen	M	1995	2	Dolphin Beach/ Dolphin Bay	N
Evie	F	2008	1	Dolphin Beach	N
Gemma	F	1988	3	Dolphin Beach	Y
Howie	M	unknown	unknown	Endeavour	Y
Kiama	M	2003	2	Dolphin Beach	N
Moki	F	unknown	unknown	Dolphin Beach	N
Nudgee	M	unknown	unknown	Endeadvour/QVC	Y
RB	M	1990	3	Dolphin Bay	N
Scooter	F	1983	4	Dolphin Bay	N
Sirius	M	1979	4	Dolphin Beach	N
Squeak	F	1979	4	Dolphin Beach	N
Starbuck	M	1998	2	Dolphin Beach/ Dolphin Bay	N
Stella	F	2013	1	Dolphin Beach	Y

Ten dolphins stayed in the same pool for the entire sampling period. Three dolphins were moved between two pools at least once. We assigned four different age levels: 1: 0–10 years, 2: 11–20 years, 3: 21–30 years, 4: 31–40 years

**Table 3:** Cause, type, date and period of antimicrobial (AB) treatment of four dolphins

Dolphin ID	Cause of AB treatment	AB medication	AB dosage	Period of treatment
Gemma	Aborted calf, prevention of infection	Amoxycillin	Oral administration, 2500 mg, twice a day	02/05 to 13/06/2017 (6 weeks)
Howie	Ocular infection	Doxycycline	Oral administration, 250 mg, twice a day	30/05 to 07/062017 (7 days)
Nudgee	Septic arthritis in shoulder joint	Amoxycillin	Oral administration, 3000 mg, twice a day	22/06 to 27/07/2017 (5 weeks)
		Gentomicin	Oral administration, 500 mg, once a week	
Stella	Inappetence	Amoxycillin	Oral administration, 4000 mg	05/07/2017 (only once)

### Dataset overview

In 86 dolphin blow and 28 pool water samples we detected a total of 4,101,122 raw 16S rRNA gene sequences, which were clustered into 2175 zero-distance operational taxonomic units (zOTUs). We removed 23 zOTU as they had an overall relative abundance of less than 0.0001%. We deleted five dolphin blow samples from the dataset, as these samples did not meet the set cut-off score of a minimum of 6000 reads (Table 1). Furthermore, we deleted 157 zOTUs from the dataset as these were identified as technical contaminants (Table S1, Figs. S1 and S2). The resulting dataset of 81 dolphin blow and 28 pool water samples contained 1991 zOTUs with a mean of 29,168 reads per sample (sd = 13,578). The rarefaction curves of dolphin blow and pool water samples (Figs. S3 and S4) and Good’s coverage (Table 4) after filtering showed that the majority of samples was sequenced to near-saturation. After comparing the beta diversity of dolphin blow and pool water microbiota we determined those zOTUs in the dolphin blow that likely originated from pool water and deleted them (Figs. S5 and S6). 1471 zOTUs remained in the dataset as ‘true’ dolphin blow microbiota.

**Table 4:** Good’s coverage and alpha diversity parameters of dolphin blow samples

Dolphin ID	Good’s coverage, mean (sd)	Richness, mean (sd)	Diversity, mean (sd)	Chao1, mean (sd)	ACE, mean (sd)	No of intra-core zOTUs
Coen	99.86 (0.05)	311 (26)	3.62 (0.40)	352 (36)	327 (29)	217
Evie	99.79 (0.05)	361 (75)	4.12 (0.68)	417 (68)	381 (75)	167
Gemma	99.88 (0.07)	205 (122)	3.48 (0.83)	241 (118)	221 (122)	NA
Howie	99.82 (0.07)	405 (70)	4.27 (0.43)	463 (68)	429 (71)	309
Kiama	99.82 (0.10)	335 (50)	4.04 (0.38)	380 (64)	352 (54)	191
Moki	99.81 (0.11)	282 (126)	3.67 (0.79)	329 (138)	297 (131)	42
Nudgee	99.89 (0.04)	198 (43)	3.44 (0.49)	239 (60)	210 (48)	115
RB	99.83 (0.04)	315 (48)	4.09 (0.45)	351 (43)	331 (46)	180
Scooter	99.88 (0.11)	262 (135)	4.15 (0.56)	305 (145)	275 (134)	51
Sirius	99.92 (0.03)	224 (100)	3.81 (0.75)	259 (102)	234 (101)	38
Squeak	99.88 (0.06)	270 (155)	4.12 (0.60)	301 (165)	284 (156)	87
Starbuck	99.84 (0.03)	391 (26)	4.17 (0.30)	439 (33)	406 (26)	306
Stella	99.88 (0.04)	224 (99)	3.11 (0.87)	273 (107)	240 (103)	NA

The table shows the average of Good’s coverage as well as richness, diversity, Chao1 and ACE species estimators of blow samples per dolphin and number of intra-core zOTUs over a period of five months (weeks 6, 11, 19, 28). Gemma and Stella were not included in the calculation of intra-core zOTUs, as they lacked the according samples (No of core zOTUs: NA)

### Alpha diversity parameters differ across individuals but stay stable over time

After deleting technical and pool water contaminants from the dataset, we calculated four different parameters of alpha diversity (richness, Shannon-Wiener diversity index, Chao1 [38, 39] and ACE [40, 41] species estimators) for each dolphin after rarefying the reads of each sample to the lowest number of reads (16,578) to account for the difference in sampling depth. The alpha diversity parameters averaged to 292 (richness, sd = 108), 4 (Shannon-Wiener diversity, sd = 0.68), 336 (Chao1, sd = 115), 307 (ACE, sd = 111) across all 81 dolphin samples. The changes of alpha diversity parameters across time are visualized in Fig. S7 (richness) as well as in Fig. S8 Shannon-Wiener diversity, S9 (Chao1) and S10 (ACE). These parameters differed significantly between dolphins (richness: *p* = 0.0015, diversity: *p* = 0.0163, Chao1: *p* = 0.0008, ACE: *p* = 0.0008) and there was no statistical support for changes over the sampling period within the individual dolphins (richness: *p* = 0.5368, diversity: *p* = 0.0938, Chao1: *p* = 0.4404, ACE: *p* = 0.4998) (Table 4 & S2).

### Beta diversity remains fairly stable in individual dolphins, but changes with time

**Fig. 2:**
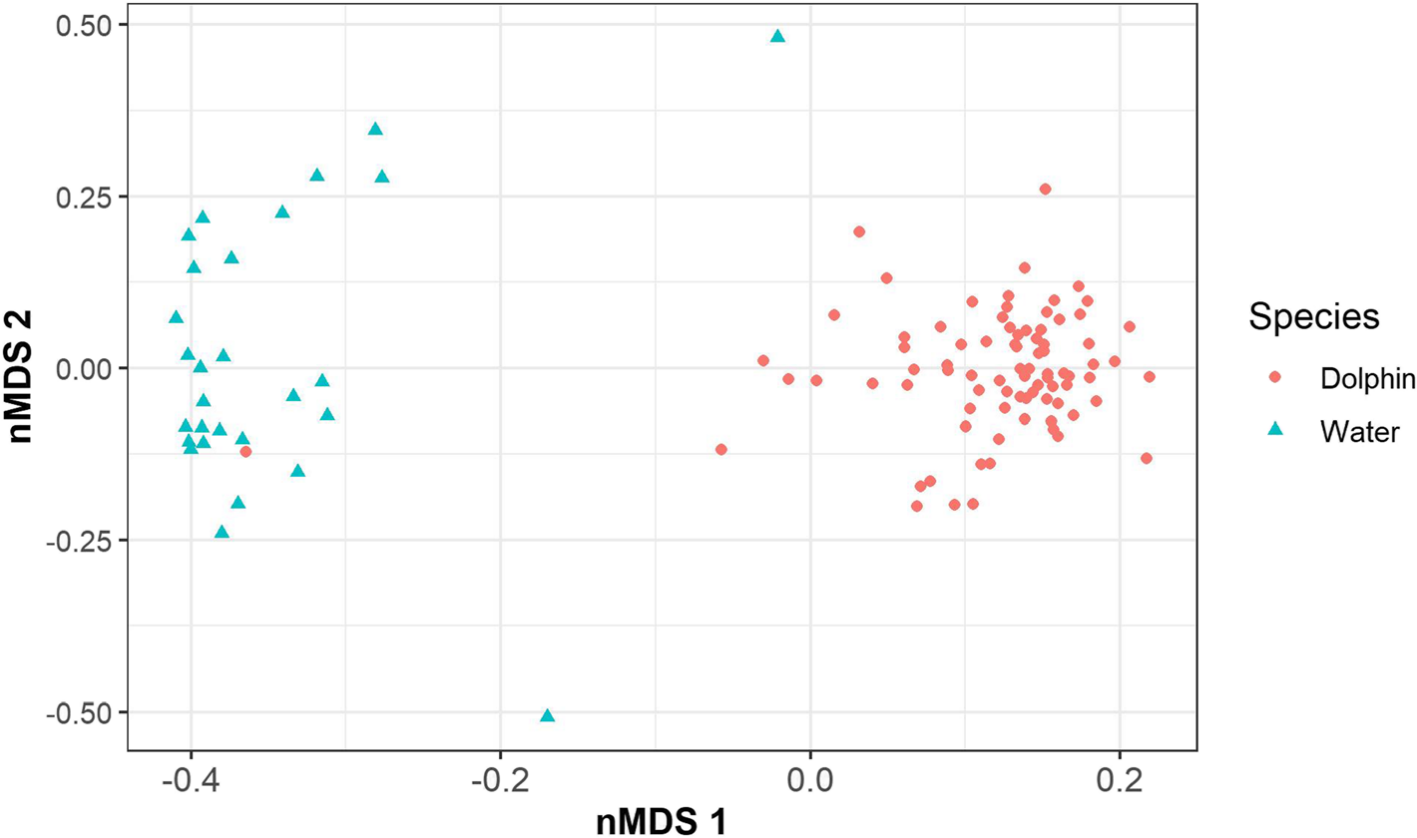
nMDS of microbiota, based on Bray-Curtis dissimilarity and unrarefied data, found in 81 dolphin blow and 28 pool water samples. A distinct separation between dolphin blow and pool water samples is present

**Fig. 3:**
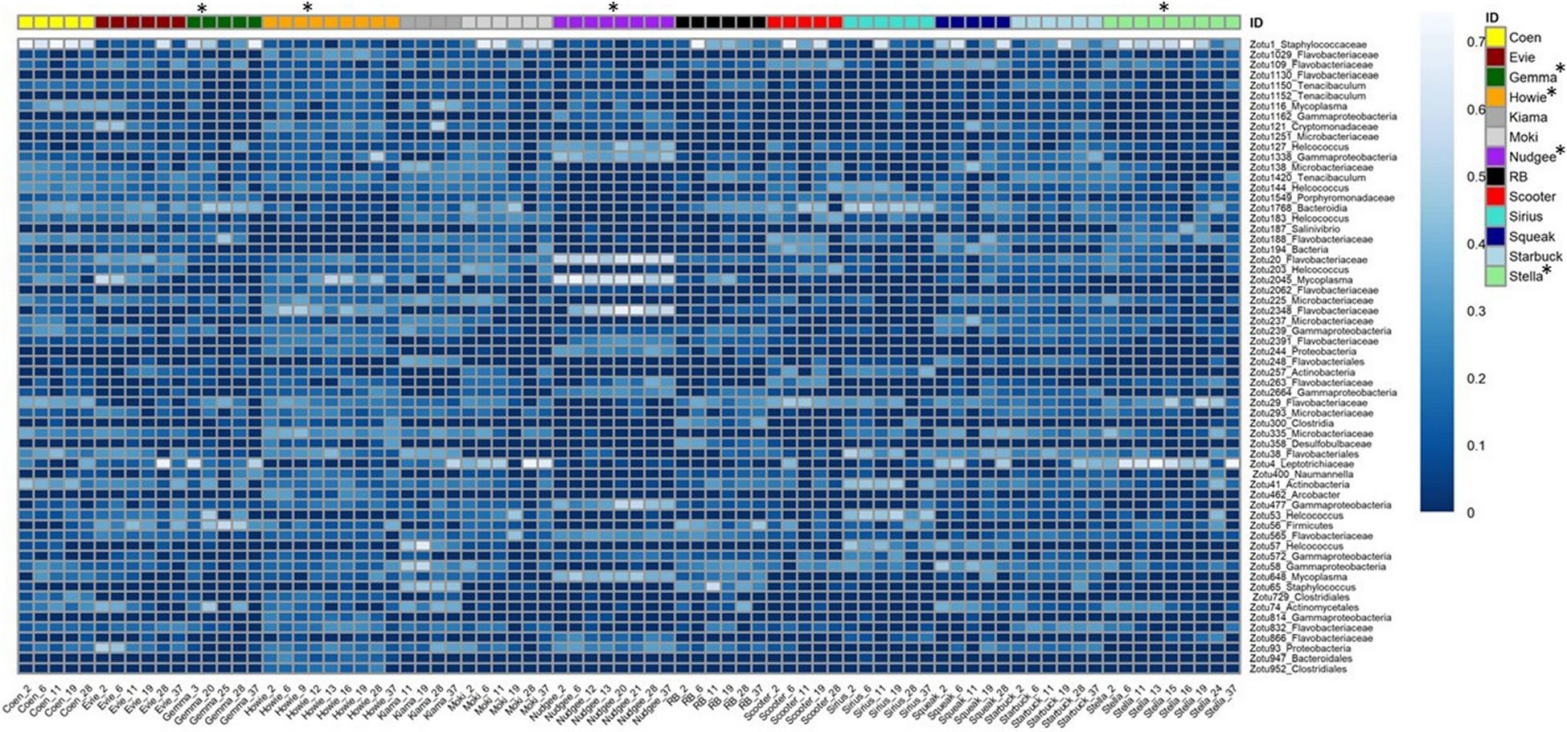
Heatmap of fourth-root transformed relative abundance of 62 zOTUs identified as significantly different between dolphins. The microbial communities of blow across individual dolphins were shown to be significantly different. The blow samples per dolphin (columns) are listed in chronological order. ‘Gemma’, ‘Howie’, ‘Nudgee’ and ‘Stella’ were marked with an asterisk to indicate their antimicrobial treatment. The position of the asterisk in the diagram indicates the timing of their treatment

We visualized the beta diversity of microbial communities by creating non-metric multidimensional scaling (nMDS) plots, based on Bray-Curtis dissimilarity and unrarefied data, that showed distinct clustering of dolphin and pool water samples (Fig. 2). To determine if the composition of microbial communities differed between dolphin blow and pool water, we fitted log-link negative binomial models to each zOTU using *mvabund* [42], with ‘dolphin’ or ‘pool water’ as an explanatory factor, Statistical significance was evaluated with *anova.manyglm* using pit-trap resampling [43], considering the sum of likelihood ratio statistics compared to an intercept-only model. This showed a significant difference between dolphin blow and pool water (sum-of-LR = 24,980 *p* = 0.001). In a similar way, we tested other factors of interest that potentially impacted the microbial communities. Time was one of those factors that was significantly associated with changes in the microbial communities in the airways of the dolphins and on those of the pool water the dolphins were housed in, as the microbiota changed over the eight months of sample collection (dolphin blow: sum-of-LR = 20,140, *p* = 0.002; pool water: sum-of-LR = 14,064, *p* = 0.002) (changes in dolphin blow microbiota over time: Fig. S11). In addition, the microbial communities of the blow differed between individual dolphins (sum-of-LR = 40,632, *p* = 0.001) (Fig. S12). The heatmap of Fig. 3 shows the distribution and relative abundance of 62 zOTUs that most significantly contributed to the differences across the individual dolphins.

When treated as fixed effects, dolphin ID (12 degrees of freedom) accounted for a deviance of 4470 across all zOTUs, whereas time (15 degrees of freedom) accounted for a deviance of 2591. Expressed relative to the total deviance — as a multivariate version of McFadden’s pseudo-R2 [44] — dolphin ID accounted for approximately 10% of the total deviance unexplained by time, while time accounted for about 6% of the total deviance unexplained by dolphin ID. When treated as random effects, we found that the standard deviation of log(expected zOTU counts) between dolphins at the same time was about 3.5 times that between time points for the same dolphin.; i.e. zOTU counts are more variable between dolphins at the same time than they are between time points for the same dolphin. That means that the difference we observed in the blow microbiota between the dolphins were larger than the differences across time. Furthermore, the microbiota of the water differed across the pool systems (sum of LR = 9987, *p* = 0.001), and there was an effect on the blow microbiota depending on the pool system the dolphins were kept in (sum-of-LR = 1736, *p* = 0.001). The dolphins’ sex (sum-of-LR = 2128, *p* = 0.002) (Fig. S13) and age (sum-of-LR = 2762, *p* = 0.001) also had an effect on the blow microbiota (Fig. S14).

### Diverse and abundant intra-core in dolphins present over five months

**Fig. 4:**
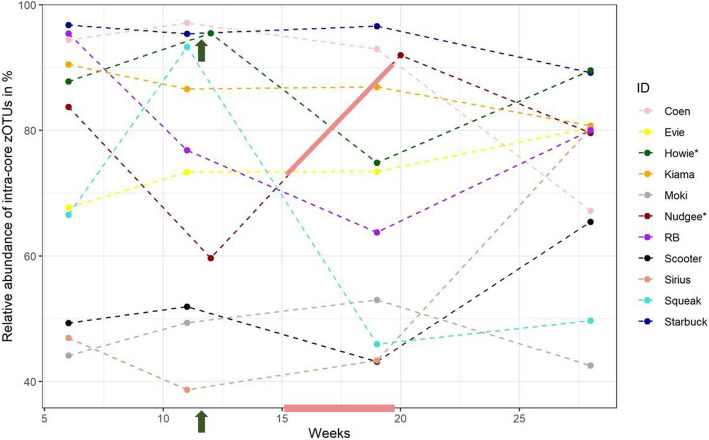
Total relative abundance of intra-core zOTUs in the blow of 11 bottlenose dolphins at four data points over five months. Only three dolphins showed a relatively stable abundance of their intra-core (variations within 10% of relative abundance), whereas others displayed a large variation over time. Howie and Nudgee were marked with an asterisk, as they received antimicrobial treatment. The light red bars indicate the period of antimicrobial treatment of Nudgee over several weeks, whereas the green arrows indicate treatment of Howie over a single week

We determined those zOTUs that persisted in each individual dolphin over time (across weeks 6, 11, 19 & 28) and labelled them as ‘intra-core’*.* Each dolphin harboured an average of 176 intra-core zOTUs in their blow on each of the four sampling occasions over five months (Table 4). In total, we found 503 intra-core zOTUs. Most intra-core zOTUs were only present in one or two dolphins. Only two intra-core zOTUs were shared by all 11 dolphins (Fig. S15). The relative abundance of the individual intra-core varied over time and among dolphins (Fig. 4), averaging to 73% relative read abundance across all dolphins and time points. Whereas the relative abundance in some dolphins like ‘Starbuck’ and ‘Kiama’ remained fairly stable over time, others showed massive variations. ‘Starbuck’ harboured the highest relative abundance (94%) of intra-core zOTUs, averaged over the four time points measured. In contrast, ‘Moki’s’ intra-core only accounted for an average of 47% of relative read abundance. Most of the intra-core zOTUs (449 of the 503) had been reported previously in the blowhole, mouth, rectum or forestomach of bottlenose dolphins as described by Bik et al. (1) (Genbank accession number: e.g., KC260893.1) or Johnson et al. (3) (e.g., FJ959551.1) (Table 5). Those intra-core zOTUs previously found in dolphins accounted for an average of 38% of each dolphin’s total relative read abundance. The taxa with the largest number of intra-core zOTUs present were *Gammaproteobacteria, Flavobacteriaceae, Bacteroidia* and *Helcococcus* (Table 5). Those dolphin-associated intra-core zOTUs with a relative abundance above 1% were zOTU1 (6.41%, ranging from 0 to 29%, *Staphylococcus, CP054831.1*), zOTU3 (4.60%, ranging from 0 to 20%, *Flavobacteriaceae*, JQ194124.1), zOTU6 (3.21%, ranging from 0 to 26%, *Corynebacteriaceae*, JQ216503.1), zOTU9 (3.03%, ranging from 0 to 19%, Gammaproteobacteria, JQ193892.1), zOTU2627 (2.98%, ranging from 0 to 43%, *Flavobacteriaceae*, FJ960391.1), zOTU1843 (2.82%, ranging from 0 to 15%, *Flavobacteriaceae*, JQ193481.1), zOTU12 (2.21%, ranging from 0 to 7%, *Gammaproteobacteria*, JQ209784.1), zOTU8 (2.11%, ranging from 0 to 15%, Gammaproteobacteria, JQ194121.1), zOTU4 (2.10%, ranging from 0 to 26%, *Leptotrichiaceae*, FJ960123.1), zOTU16 (1.94%, ranging from 0 to 11%, *Flavobacteriaceae*, FJ960162.1), zOTU2045 (0.1.78%, ranging from 0 to 27%, *Mycoplasmataceae,* JQ194126.1), zOTU22 (1.48%, ranging from 0 to 25%, bacteria, JQ193497.1), zOTU20 (1.27%, ranging from 0 to 15%, *Flavobacteriaceae*, JQ193429.1), zOTU1768 (1.24%, ranging from 0 to 86%, *Bacteroidia*, JQ194579.1), zOTU120 (1.16%, ranging from 0 to 10%, *Bacteria*, JQ214918.1) and zOTU2348 (1.05%, ranging from 0 to 22%, *Flavobacteriaceae*, FJ959551.1). The remaining 55 intra-core zOTUs that were not previously detected in dolphins have mostly been reported as present in wastewater (e.g., JX515418.1) or on the skin, the gut or mouth of terrestrial mammals (e.g., EU681994.1) (Table 6). The majority of intra-core bacteria were novel and could therefore not be classified to genus level. *Actinobacillus, Arcobacter, Helcococcus* and *Tenacibaculum* were some of the few intra-core taxa that were classified to genus level and that were highly abundant within the core (Tables 5 and 6).

**Table 5:** Taxonomic affiliations of 449 intra-core zOTUs that were previously found in the mouth, forestomach or blowhole of bottlenose dolphins and their Genbank accession number, present across weeks 6, 11, 19 and 28 in 11 dolphins. The majority of listed taxa included more than one intra-core zOTU

No. of core zOTU with the same taxonomic affiliation	Taxonomic affiliation	Genbank accession (e.g.)
129	Gammaproteobacteria	JQ209784.1
119	*Flavobacteriaceae*	FJ959551.1
45	Bacteria	JQ215599.1
20	*Helcococcus*	FJ959658.1
11	Bacteroidia	FJ959660.1
10	Clostridiales	JQ214550.1
9	SR1_genera_incertae_sedis	JQ216558.1
8	*Actinobacillus*	KC257787.1
5	Bacteroidales	FJ959660.1
5	Bacteroidetes	JQ215352.1
4	*Alcaligenaceae*	FJ960346.1
4	Campylobacterales	FJ959586.1
4	Flavobacteriales	JQ194338.1
4	*Mycoplasma*	JQ194022.1
3	Desulfobulbaceae	JQ214362.1
3	*Marinicella*	FJ959830.1
3	*Microbacteriaceae*	JQ194103.1
4	Oceanospirillales	JQ213884.1
3	*Phocoenobacter*	KC260904.1
3	*Porphyromonadaceae*	JQ209212.1
2	Actinomycetales	JQ208827.1
2	Cardiobacteriales	FJ960414.1
2	*Guggenheimella*	FJ959658.1
2	Firmicutes	JF917274.1
2	*Lachnospiraceae*	JQ214724.1
2	Mycoplasmataceae	JQ194022.1
2	*Peptoniphilaceae*	JQ211561.1
2	Proteobacteria	FJ959558.1
2	Pseudomonadales	JQ194701.1
2	Sphingobacteriia	FJ959720.1
1	Actinobacteria	JQ216846.1
1	*Alicyclobacillus*	KC259493.1
1	*Anaerovorax*	FJ959595.1
1	Betaproteobacteria	FJ959731.1
1	Burkholderiales	FJ959744.1
1	*Corynebacteriaceae*	JQ216503.1
1	*Cryomorphaceae*	JQ194623.1
1	Deltaproteobacteria	JQ214362.1
1	Epsilonproteobacteria	FJ959609.1
1	Firmicutes	JQ194061.1
1	Leptotrichiacea*e*	FJ960123.1
1	*Microbacteriaceae*	LR640309.1
1	*Naumannella*	KC259559.1
1	*Parcubacteria_genera_incertae_sedis*	JQ209049.1
1	*Pasteurellaceae*	JQ193892.1
1	Proteobacteria	KM823720.1
1	*Pseudomonas*	CP041013.1
1	*Salinivibrio*	JQ194108.1
1	*Spirochaetaceae*	KC259401.1
1	Spirochaetes	KC259034.1
1	Spirochaetia	KC260523.1
1	Staphylococcaceae	CP054831.1
1	Staphylococcus	CP054831.1
1	*Sulfurospirillum*	JQ215956.1
1	*Treponema*	KC260218.1

**Table 6:** Intra-core zOTUs that were not previously found in bottlenose dolphins, their taxonomic affiliation, source, average relative abundance across dolphins and Genbank accession number, present across weeks 6, 11, 19 and 28 in 11 dolphins

zOTU no.	Taxonomic affiliation	Environment of most similar sequences	Genbank accession
Zotu0001	*Microbacteriaceae*	Human urinary tract	CP054831.1
Zotu0025	*Staphylococcaceae*	Human urinary tract	CP054831.1
Zotu0033	*Corynebacteriaceae*	Human skin	KU689893.1
Zotu0056	Firmicutes	Mineral hot springs	JF917274.1
Zotu0065	*Staphylococcus aureus*	Human blood	HG795797.1
Zotu0072	*Staphylococcus*	Frog skin	HM330254.1
Zotu0079	Bacteroidetes	California sea lion stomach	KF067368.1
Zotu0138	*Microbacterium esteraromaticum*	bioaerosol	MG751356.1
Zotu0141	*Beta*proteobacterium	hypersaline lake	MG282144.1
Zotu0149	*Microbacteriaceae*	wastewater	GQ062150
Zotu0175	*Leucobacter sp.*	soil	GU235593.1
Zotu0180	Bacteria	seawater	MK129353.1
Zotu0210	*Salinivibrio costicola*	Human skin	JN683955.1
Zotu0221	Gammaproteobacteria	Human skin	JF153221.1
Zotu0223	*Bacteriovoracaceae*	seawater	JX294354.1
Zotu0237	*Microbacteriaceae*	cheese rind	LT698608.1
Zotu0259	*Microbacteriaceae*	Wastewater treatment system	LR640309.1
Zotu0279	*Clostridia*	human skin	JQ205124.1
Zotu0300	*Leucobacter sp.*	human skin	FN823848.1
Zotu0313	*Okibacterium sp.*	soil	GU235593.1
Zotu0335	*Comamonadaceae*	soil	EU681994.1
Zotu0355	Marine bacteria	Human skin	MG099642.1
Zotu0368	*Vibrio sp.*	seawater	MG705679.1
Zotu0377	*Bacteriovoracaceae*	marine sediment	GU235593.1
Zotu0385	*Gracilibacteria bacterium oral taxon 873*	wastewater	GU235593.1
Zotu0417	*Prevotella melaninogenica*	human oral cavity	MK129353.1
Zotu0420	*Bacteriovoracaceae*	human skin	JN713493.1
Zotu0422	*Gammaproteobacteria*	seawater	JQ380127.2
Zotu0460	*Vibrio*	California sea lion mouth	EU137548.1
Zotu0491	*Marine bacteria*	unknown	CP014053.1
Zotu0508	*SR1_genera_incertae_sedis*	Seawater	HQ122382.1
Zotu0521	*Vibrio sp.*	Dog gut	KF571771.1
Zotu0570	*Microbacterium esteraromaticum*	coral	CP032548.1
Zotu0590	Marine bacteria	bioaerosol	MK506675.1
Zotu0645	Marine bacteria	seawater	KU689893.1
Zotu0723	*Vibrio sp.*	seawater	HG795797.1
Zotu0763	*Gracilibacteria bacterium canine oral taxon 323*	marine sediment	HM330254.1
Zotu0835	Bacteria	dog’s oral cavity	KF067368.1
Zotu0954	Bacteria	coral	JF087814.1
Zotu0959	*Bacteriovoracaceae*	prairie dog flea	MG751356.1
Zotu1083	*Vibrio alginolyticus*	Seawater	KU689893.1
Zotu1157	*Escherichia coli*	Seawater	MG282144.1
Zotu1214	Bacteroidetes	human feces	JF087814.1
Zotu1449	Marine bacteria	Seawater	GQ062150
Zotu1620	*Methylophaga*	Seawater	GU235593.1
Zotu1639	Marine bacteria	fresh water fish	KF571771.1
Zotu1644	*Tenacibaculum sp. DSM 106434*	Seawater	GU235593.1
Zotu1840	*Staphylococcus*	fish skin	MK129353.1
Zotu1968	Gammaproteobacteria	Human skin	CP054831.1
Zotu2070	*Staphylococcus*	frog skin	JN683955.1
Zotu2659	*Corynebacteriaceae*	human skin	HM330254.1

Furthermore, we determined any ‘inter-core’ zOTUs and their relative abundance. The inter-core was defined as those zOTUs that were present across ten dolphins, randomly selected from our 13 study dolphins, at a specific time point. We picked weeks 2, 6, 11, 19, 28 and 37 as these points in time. Ten dolphins shared an average of 32 (sd = 20) inter-core zOTUs at any sample collection point in time (weeks 2, 6, 11, 19, 28, 37). The temporal dynamics of the inter-core from week 2 to 37 are visualized in Fig. S20. These inter-core zOTUs accounted for a mean relative read abundance of 25% (sd = 9) in each dolphin. All 97 inter-core zOTUs detected were also part of the intra-core and were previously collected from the mouth, blowhole or forestomach of bottlenose dolphins [1, 3]. About half of the inter-core zOTUs were only present across all ten dolphins at one sampling point (Fig. S16).

### Few potential pathogens among intra-core zOTUs

Within the intra-core of healthy and health-compromised dolphins, 10 zOTUs belonged to genera that comprise pathogenic ‘species’ linked to infectious disease in marine mammals. These genera included *Pseudomonas, Staphylococcus* and *Mycoplasma* (Tables 5 and 6). Venn-Watson [45, 46] associated *Pseudomonas* and *Staphylococcus* with pneumonia and mortality in bottlenose dolphins, while *Mycoplasma* is a common causative agent of infectious disease in pinnipeds [47]. We are unable to tell, whether the intra-core, consisting of 449 zOTUs, contained any additional potentially pathogenic genera, as most of the core members were novel and could therefore not be classified to genus level.

### Antimicrobial treatment might have short term effect on airway microbiota

To test the potential effect of the medical condition and antimicrobial treatment of four dolphins on their microbial communities, we classified the blow samples into four groups: samples from dolphins that were in good physical conditions and therefore didn’t receive any antimicrobial treatment were labelled as ‘None‘. Samples of physiologically compromised and treated dolphins that were collected before the animal was treated received the label ‘Before‘. Those samples that were collected (within one week) after the animals were treated were called ‘Directly_After‘and those samples collected at least two weeks after treatment were named ‘After‘. The blow microbiota of the dolphins with medical conditions (Table 3) generally (compared over the entire sampling period) did not differ from those of the healthy dolphins (sum-of-LR = 7607, *p* = 0.121) (Fig. S17). The healthy dolphins’ blow bacterial communities were also not significantly different to those of the sick dolphins before (‘None‘vs. ‘Before‘: sum-of-LR = 11.1, *p* = 0.077), directly after (‘None‘vs. ‘Directly_After‘: sum-of-LR = 881.8, *p* = 0.105) and after antimicrobial treatment (‘None‘vs. ‘After‘: sum-of-LR = 20.32, *p* = 0.108). In addition, the microbial communities of the sick dolphins did not differ ‘before’ and ‘after’ the antimicrobial treatment (sum-of-LR = 2102, *p* = 0.166). However, we detected a slight difference between the blow microbiota ‘before’ and ‘directly after’ the treatment (sum-of-LR = 1910, *p* = 0.049) (Fig. S18). Interestingly, we also found a significant difference between the samples of the sick dolphins collected ‘directly after’ and at least two weeks ‘after’ the application of antibiotics (sum-of-LR = 2545, *p* = 0.016) (Fig. S19).

Two of the dolphins included in this study (‘Gemma’ and ‘Nudgee’) were both treated with Amoxycillin twice a day over a period of five to six weeks (Table 3). To test for a potential impact of the Amoxycillin treatment on the airway microbiota of the two dolphins, we firstly compared the samples of ‘Gemma’ and ‘Nudgee’ after their treatment with those of the other dolphins that did not receive any antimicrobial treatment during the course of the sample collection. We received a result which was close to significant (sum-of-LR = 1214, *p* = 0.054). Secondly, we compared the samples of ‘Gemma’ and ‘Nudgee’ before they were treated with Amoxycillin with those after their treatment. In this case, we did not find evidence of a significant difference (sum-of-LR = 0, *p* = 0.999).

## Discussion

### Dolphins harboured individual-specific ‘signature’ microbiota (intra-core) in blow

The dolphins in this study harboured bacterial communities in their blow that changed in their alpha and beta diversity over the sampling period of eight months. However, we found evidence to conclude that individual identity of the dolphins had a higher determining impact on the blow microbiota than the factor of time (Fig. 3 & S15). Thus, our findings support the short-term results of Lima et al. [4] over a substantially longer time scale. In addition, each dolphin showed a unique intra-core ‘signature’ (Fig. 4), as the majority of intra-core zOTUs were present in a single or two dolphins (Fig. S13). With an average of 73% (ranging between 47 to 94%) of the relative read abundance, the intra-core zOTUs dominated the blow microbiota of individual dolphins, whereas in humans and mice proof of a communal bacterial core of the airways is still lacking (15, 16). Thus, our findings provide evidence that the bacterial communities in dolphin blow are not merely transient. In fact, as much as 38% of the blow microbiota in our study animals had been reported previously in bottlenose dolphins. We propose that dolphins harbour a relatively stable individual-specific microbiota that colonizes the airways. Nevertheless, we found that the relative read abundance of intra-core zOTUs varied across time in most dolphins, including the healthy animals (Fig. 4). Segal et al. [19] and Dickson et al. [16] demonstrated in humans and mice that changes in the airway microbiota can occur even without any obvious clinical signs. Slight shifts in immune function were found to impact the respiratory microbial communities and they may have been at play here. The strong variation in intra-core zOTUs within individuals highlights that the lung microbiota of dolphins is dynamic and there are likely internal physiological as well as external factors such as temperature [48] involved that impact the bacterial communities on a day-to-day basis.

### Dolphins shared significant number of inter-core with conspecifics

In addition, the dolphins did not only maintain a stable intra-core over time, but also harboured a significant number of inter-core zOTUs that they shared with their conspecifics across pools at certain time points. With a mean of 25% of relative read abundance, the inter-core in our dolphins was less abundant than that Apprill et al. [7] found in the blow across 26 humpback whales. Yet, Apprill et al. [7] only determined the inter-core at one single time point, whereas we noticed a large variation in relative abundance of inter-core with a range of 17 and 41% in our dolphins across time (Fig. S20). However, it is interesting to notice that Apprill et al. [7] found the humpback whales to share more than a third of their blow microbiota across two populations (north Atlantic and Pacific Ocean), whereas our study dolphins were kept in the same facility and some even shared the same pool system. This provides additional evidence for the airway microbiota of cetaceans to maintain a certain ratio of stable individual-specific core residents that may be relatively independent of environmental influences.

### Impact of antimicrobial treatment was not clearly evident

We did not find clear evidence that the antimicrobial treatment that the dolphins received in this study had a significant impact on their blow microbiota. There was no general difference between the microbial communities of the sick vs. the health dolphins, seen over the entire sampling period. However, within the dolphins that received treatment, we found evidence that the bacterial blow communities were impacted right after the antimicrobial treatment and returned back to ‘normal’ within two weeks of treatment (Figs. S18 & S19). We can only speculate that the blow microbiota were indeed briefly impacted by the treatment and returned back to their physiological state soon after (no difference to the blow of healthy, untreated dolphins). It is interesting to notice that the relative read abundance of intra-core zOTUs and its variation over time did not show any obvious changes right after the application of antimicrobial treatment (Fig. 4). When comparing the samples of the two dolphins that were treated with Amoxycillin over several weeks with those of the dolphins that did not receive any treatment, we received an outcome which was just above the significance threshold. Although the statistical result was not clear, it might hint towards an impact of the Amoxycillin treatment on the microbial communities.

### Sex, age and social setting of dolphins appeared to impact blow microbiota

In contrast to Lima et al. [4] and Bik et al. [1], we found an impact of sex and age of the dolphins on their airway microbiota (Figs. S13 & S14). Furthermore, the pool system the dolphins were kept in also made a significant difference. Although the bacterial communities differed across the pool systems, we assumed that the filtering of dolphin blow samples from the pool water bacteria early on in our analysis would have prevented a large impact from the pool water itself on the airway microbiota of dolphins. Hence, we followed speculations of Bik et al. [1] and hypothesized that dolphins kept in the same pool system inoculate each other’s airways with their blow microbiota and therefore contribute to their pool mates’ blow community composition. A similar effect has been shown for children whose airway microbiota changed with the number of their social contacts [49] as well as for mice housed in the same cage, known as cage-effect [50].

## Conclusions

We conclude that the airways of dolphins are colonized by resident core bacteria that can differ in abundance in relation to more transient bacteria. Although we did not find a clear correlation between these core bacteria and the health of the dolphins, potentially due to the small sample size of sick dolphins and the variation of their illnesses, we speculate that these individual-specific core bacteria interact with the immune response of the respiratory tract and support its function. To support our assumption, a larger number of dolphins with a consistent respiratory pathophysiology and additional local immune parameters need to be analysed. For now, we confirm the potential of the analysis of blow microbiota as a future biomarker for the physiological state of the airways in cetaceans.

## Methods

### Sample collection

Sea World Marine Park Gold Coast, Australia, granted us permission to use the blow samples of 13 captive bottlenose dolphins (*Tursiops truncatus*) for this study. We tested the microbiota in the blow samples for temporal stability over a period of eight months. The dolphins were kept in three separate pool systems and a quarantine pool (Fig. 1). Three of the 13 sampled dolphins were moved between pools at least once (Table 2). The pools the dolphins were kept in were fed by a constant influx of seawater from the adjacent Pacific Ocean. The pool systems (Fig. 1) could be divided into four to six ‘subpools’ by closing the partition gates. Even when gates between ‘subpools’ were closed, water flow was retained between the pools. Therefore, a water sample of any of the ‘subpools’ was representative for the entire pool system.

The dolphins were trained to exhale on command. Sample collection was generally performed on Monday mornings at 9 am, as part of a feeding and enrichment session. After blow sample collected for this study was completed, the dolphins remained in the care of Sea World. For each sampling event the trainer held a sterile ‘yellow cap’ container (Techno Plas Pty Ltd., St Marys, South Australia, Australia) with a maximum volume of 70 ml, with its lid removed, upside down about 10 cm above the dolphin’s blowhole. Once the dolphin had exhaled, the trainer screwed the lid back on and stored the sample in an Esky on ice. Each sample contained a volume of approximately 200 μl. Every time the trainers collected blow samples from the dolphins, they also took a sample of the surface water of each pool system as controls. A volume of about 200 ml was collected per water sample using a sterile ‘yellow cap’ container (Techno Plas Pty Ltd., St Marys, South Australia, Australia). We selected the surface water for sample collection, as this is the layer the dolphins were most likely to inhale small quantities of during their breathing cycle [6]. Samples were transferred from the ice-filled Esky to a − 20 °C freezer following collection and then shipped to UNSW on dry ice.

### DNA extraction and 16S rRNA gene sequencing

The water samples were filtered through a Sterivex filter unit (0.22 μm, EMD Millipore Corporation, Billerica, USA). The liquid of the blow samples was transferred from collection containers into the tubes provided with the FastDNA Spin Kit for Soil (MP Biomedicals, Santa Ana, California, USA). We extracted the nucleic acids from blow and water samples following the manufacturer’s protocol (MP Biomedicals, Santa Ana, California, USA). For amplification of the genetic regions (V1 – V3) of the samples’ nucleic acids, we followed the protocol in Vendl et al. [51]. We included the following samples as technical controls in the DNA amplification process: two positive PCR controls (PCR reagents plus the genomic DNA of *Escherischia coli*), seven negative PCR controls (PCR reagents only with sterile water instead of DNA sample) and four blank DNA extractions (reagents for DNA extraction only). We sent the PCR products to the Ramaciotti Centre for Genomics (UNSW Sydney, Australia) where samples were processed for purification, library preparation and paired-end amplicon sequencing (2 × 300 bp) on the Illumina MiSeq platform.

### Sequence data processing

We performed an initial quality check with *FastQC* [52], and processed the paired-end reads with *USEARCH* version 10.240 [53]. We followed the protocol of Vendl et al. [51] for further bioinformatic processing. As in Vendl et al. [51] the reads were clustered into zero-radius operational taxonomic units (zOTUs) with 100% similarity. The use of zOTUs is therefore a similar approach to the formation of amplicon sequence variants (ASV) [54].

### Data analysis

We removed those zOTUs from the dataset that were shown to be Archaea, chloroplasts or mitochondria. Prevalence-based filtering of potential technical contaminant zOTUs (derived from positive and negative PCR controls and blank DNA extractions) was performed using the R package *decontam* (v 3.12) [55]. We used zOTU tables as input for both the *isContaminant()* and *isNotContaminant()* function. For both functions the following parameters were applied: (method = “prevalence,” threshold = 0.5, normalize = TRUE, detailed = TRUE) as demonstrated in Seferovic [56]. We identified 157 zOTUs that likely originated from technical controls (Table S1, Figs. S1 and S2) and deleted them from the dolphin blow dataset. Figure S2 shows the bimodal division between dolphin zOTUs and technical control zOTUs. Furthermore, after filtering the technical contaminants, we scanned for those zOTUs in the dolphin microbiota that likely derived from pool water. We pooled all 28 water samples collected over the sampling period and compared them to the dolphin zOTUs. To identify water contaminants, we used the same parameters as for the technical contaminants within the R package *decontam.* 520 zOTUs were identified as belonging to the pool water samples and were deleted, while 1471 zOTUs remained in the dolphin microbiota. Figure S5 shows the bimodal division between dolphin zOTUs and water control zOTUs.

We created rarefaction curves of the samples using the package *phyloseq* (v1.24.2) [57]. The rarefaction curves and the related rarefaction analysis tested, if the blow and seawater microbiota were sampled to saturation and therefore well represented the microbial communities they were sampled from. We discarded six dolphin samples, as their number of counts were below 6000 reads (Table 1). We used the R package *nlme* (v3.1–140) [55] to assess whether the four alpha diversity parameters were associated with the time of sample collection or the individual dolphins [58]. For each parameter of alpha diversity (e.g., richness, Chao1), we fitted linear mixed effects models with a fixed effect of ‘Dolphin ID’ (individual dolphins) or ‘time’ (the week sample was collected), depending on which factor the model tested for, random intercepts for ‘time’ or ‘Dolphin ID’, and a continuous AR(1) residual structure, and applied a likelihood ratio test to compare models with and without a fixed effect of ‘Dolphin ID’ or ‘time’. To assess the importance of the random intercepts in this model (impact of individual dolphins), we performed a restricted likelihood ratio test using the *RLRsim* package (v3.1–3) in R [59].

To determine if any of the factors of interest (e.g., dolphin blow vs. pool water, dolphin ID, time, antimicrobial treatment, age, sex) were associated with the composition of the microbial communities tested, we fitted log-link negative binomial models to each zOTU, with the factor(s) in question included as fixed effects using *mvabund* (v4.0.1) [42], in each case, the log of total sequence counts per sample included as an offset to account for differences in sampling depth. Statistical significance was evaluated with the *anova.manyglm* function, which uses pit-trap resampling [43], considering the sum-of-likelihood ratio statistics compared to a model excluding the factor of interest. This approach to the analysis of microbiota composition with *mvabund* has been previously described in detail by Vendl et al. [51] (Supplementary materials S3).

As an alternative method of examining the relative contribution of dolphin ID and time to the variation in microbiome composition, we fit a similar log-link negative binomial model using *glmmTMB* (v1.0.2.1) [60], this time including dolphin ID and time as multivariate random effects across zOTUs. Here, the outcome of main interest was the relative size of the estimated variances of these random effects.

Bacterial beta diversity of pool water and dolphin blow samples, as well as that of some of the other factors in question (e.g., age, sex, dolphin ID) was visualized using the package *vegan* (v2.5–5) for community ecology analysis [61] .

To ensure comparability of intra-core zOTUs across dolphins, we exclusively considered four samples per dolphin (weeks 6, 11, 19, 28) over five months. We excluded the weeks 2 and 37, as we lacked the samples of several dolphins for these weeks (Table 1). Including those would have resulted in a smaller number of dolphins in the analysis of intra-core, as we wanted to make sure to use the same number of samples (four) per dolphin. We excluded ‘Stella’ and ‘Gemma’ from the intra-core analysis, as they did not have all required samples over the above-mentioned weeks. Missing samples of ‘Howie’ and ‘Nudgee’ were replaced by those obtained a week before or after. ‘Howie’ and ‘Nudgee’ were the only two dolphins included in the intra-core analysis that were treated with antibiotics. Statistical analysis of microbial community results was performed using R statistical software (v3.5.1) (http://cran.r-project.org/).

## Supplementary Information

**Additional file 1: Fig. S1.** Shows a scatterplot of the technical contaminant zOTUs of control samples (‘TRUE’ in green) and dolphins blow zOTUs (‘FALSE’ in red). The R package decontam determined 157 technical contaminants which were then deleted from the 81 dolphin blow samples. **Fig. S2.** Shows a histogram of the technical contaminant zOTUs of control samples (bars on the left) and dolphins blow zOTUs (bars on the right). The R package decontam determined 157 technical contaminants which were then deleted from the 81 dolphin blow samples. The figure shows the bimodal division between dolphin zOTUs and technical control zOTUs. **Fig. S3.** Rarefaction curves of dolphin blow samples. The majority of samples was sampled to saturation. **Fig. S4.** Rarefaction curves of pool water samples. The majority of samples was sampled to saturation. **Fig. S5.** Shows a scatterplot of the contaminant zOTUs of pool water samples (‘TRUE’ in green) and dolphins blow zOTUs (‘FALSE’ in red). The R package decontam determined 520 water contaminants which were then deleted from the 81 dolphin blow samples. **Fig. S6.** Shows a histogram of the water contaminant zOTUs of pool water samples (bars on the left) and dolphins blow zOTUs (bars on the right). The R package decontam determined 520 water contaminants which were then deleted from the 81 dolphin blow samples. The figure shows the bimodal division between dolphin zOTUs and water zOTUs. **Fig. S7.** Shows the alpha diversity parameter, richness, across 37 weeks of sample collection in the 13 study dolphins. **Fig. S8.** Shows the alpha diversity parameter, Shannon-Wiener diversity, across 37 weeks of sample collection in the 13 study dolphins. **Fig. S9.** Shows the alpha diversity parameter, Chao1, across 37 weeks of sample collection in the 13 study dolphins. **Fig. S10.** Shows the alpha diversity parameter, ACE, across 37 weeks of sample collection in the 13 study dolphins. **Fig. S11.** nMDS plot based on Bray-Curtis dissimilarity matrix of 81 dolphin blow and 28 pool water samples. A clear distinction between the microbial community composition in dolphin blow and pool water is evident. **Fig. S12.** nMDS plot based on Bray-Curtis dissimilarity matrix of 81 dolphin blow samples coloured according to their ID (individual dolphins). Although not clearly evident in this plot, the mvabund-based analysis showed a significant impact of the factor ‘dolphin ID’. **Fig. S13.** nMDS plot based on Bray-Curtis dissimilarity matrix of 81 dolphin blow samples coloured according to the dolphins’ sex. The diagram provides a hint that the mvabund-based analysis showed a significant impact of the factor ‘sex’ on the microbial communities. **Fig. S14.** nMDS plot based on Bray-Curtis dissimilarity matrix of 81 dolphin blow samples coloured according to the dolphins’ age groups. The diagram provides some evidence that the mvabund-based analysis showed a significant impact of the factor ‘age’ on the microbial communities. The analysis was based on four age levels: 1: 0–10 years, 2: 11–20 years, 3: 21–30 years, 4: 31–40 years. **Fig. S15.** Frequency histogram showing the presence of 503 intra-core zOTUs across 11 bottlenose dolphins. Although a large number of intra-core zOTUs is present, only a minority is shared by most dolphins. **Fig. S16.** Frequency histogram showing the presence of 97 inter-core zOTUs across ten dolphins in week 2, 6, 11, 19, 28, 37. More than half of the inter-core zOTUs were only present at one sampling point. **Fig. S17.** nMDS plot based on Bray-Curtis dissimilarity matrix of 81 dolphin blow samples coloured according to their health status. The mvabund-based analysis did not provide evidence for a general difference between healthy/untreated and sick/treated dolphin. **Fig. S18.** nMDS plot based on Bray-Curtis dissimilarity matrix of 16 blow samples of dolphins that had received an antimicrobial treatment during the sample collection period. The samples are coloured according to the timing of their collection relative to the timing of treatment (‘Before’: samples collected before treatment started; ‘Directly_after’: samples collected within one week after treatment). The mvabund-based analysis provided evidence for a significant difference between the ‘Before’ and the ‘Directly_after’ samples. **Fig. S19.** nMDS plot based on Bray-Curtis dissimilarity matrix of 18 blow samples of dolphins that had received an antimicrobial treatment during the sample collection period. The samples are coloured according to the timing of their collection relative to the timing of treatment (‘After’: samples collected at least two weeks after treatment started; ‘Directly_after’: samples collected within one week after treatment). The mvabund-based analysis provided evidence for a significant difference between the ‘After’ and the ‘Directly_after’ samples. **Fig. S20.** This scatterplot shows the temporal dynamics of the inter-core in the studied dolphins from week 2 to 37. The inter-core was defined as those zOTUs that were present across ten dolphins, randomly selected from our 13 study dolphins, at a specific time point. We picked weeks 2, 6, 11, 19, 28 and 37 as these points in time. **Table S1.** Shows those 157 zOTUs and their taxonomy that were identified as technical contaminants and subsequently deleted from the dataset of dolphin blow microbiota. **Table S2.** Alpha diversity parameters of dolphin blow microbiota for each time point over the sampling period of 37 weeks: richness, diversity, Chao1 and ACE species estimators.

## Data Availability

Sequence data of the dolphin blow, water and technical control samples are available in the NCBI Sequence Read Archive under BioProject accession no PRJNA562386. The code script of the statistical analysis in R studio is available as supplementary material S1 (*S1_RCode_Dolphin.blow.manuscript.nb*).
