# Supplementary material for: Highly abundant core taxa in the blow within and across captive bottlenose dolphins provide evidence for a temporally stable airway microbiota
Source: BMC Microbiol. 2021 Jan 9;21:20. doi: 10.1186/s12866-020-02076-z (PMC7796641; doi:10.1186/s12866-020-02076-z)
Supplement: Supplementary file 1 — Additional file 1: Fig. S1. Shows a scatterplot of the technical contaminant zOTUs of control samples (‘TRUE’ in green) and dolphins blow zOTUs (‘FALSE’ in red). The R package decontam determined 157 technical contaminants which were then deleted from the 81 dolphin blow samples. Fig. S2. Shows a histogram of the technical contaminant zOTUs of control samples (bars on the left) and dolphins blow zOTUs (bars on the right). The R package decontam determined 157 technical contaminants which were then deleted from the 81 dolphin blow samples. The figure shows the bimodal division between dolphin zOTUs and technical control zOTUs. Fig. S3. Rarefaction curves of dolphin blow samples. The majority of samples was sampled to saturation. Fig. S4. Rarefaction curves of pool water samples. The majority of samples was sampled to saturation. Fig. S5. Shows a scatterplot of the contaminant zOTUs of pool water samples (‘TRUE’ in green) and dolphins blow zOTUs (‘FALSE’ in red). The R package decontam determined 520 water contaminants which were then deleted from the 81 dolphin blow samples. Fig. S6. Shows a histogram of the water contaminant zOTUs of pool water samples (bars on the left) and dolphins blow zOTUs (bars on the right). The R package decontam determined 520 water contaminants which were then deleted from the 81 dolphin blow samples. The figure shows the bimodal division between dolphin zOTUs and water zOTUs. Fig. S7. Shows the alpha diversity parameter, richness, across 37 weeks of sample collection in the 13 study dolphins. Fig. S8. Shows the alpha diversity parameter, Shannon-Wiener diversity, across 37 weeks of sample collection in the 13 study dolphins. Fig. S9. Shows the alpha diversity parameter, Chao1, across 37 weeks of sample collection in the 13 study dolphins. Fig. S10. Shows the alpha diversity parameter, ACE, across 37 weeks of sample collection in the 13 study dolphins. Fig. S11. nMDS plot based on Bray-Curtis dissimilarity matrix of 81 dolphin blow and 28 [file 12866_2020_2076_MOESM1_ESM.zip › S1_RCode_Dolphin.blow.manuscript.nb.html]

Dolphin\_blow\_uparse\_Oct2020\_2


Code 

- Show All Code
- Hide All Code
- Download Rmd

# Dolphin\_blow\_uparse\_Oct2020\_2

This is an R Markdown Notebook. When you execute code within the notebook, the results appear beneath the code.

Try executing this chunk by clicking the *Run* button within the chunk or by placing your cursor inside it and pressing *Ctrl+Shift+Enter*.

Add a new chunk by clicking the *Insert Chunk* button on the toolbar or by pressing *Ctrl+Alt+I*.

When you save the notebook, an HTML file containing the code and output will be saved alongside it (click the *Preview* button or press *Ctrl+Shift+K* to preview the HTML file).

The preview shows you a rendered HTML copy of the contents of the editor. Consequently, unlike *Knit*, *Preview* does not run any R code chunks. Instead, the output of the chunk when it was last run in the editor is displayed.

############Directories


```
#Harold
setwd("Z:/SCI/BEES/MMR/Students_Postdocs/Cat/PhD/Dolphin blow_Sea world/Statistics_2/Output.files_uparse_stats.dolphin")

#Jack
#setwd("Z:/Students_Postdocs/Cat/PhD/Dolphin blow_Sea world/Statistics_2/Output.files_uparse_stats.dolphin")
```


############Load libraries


```
library(seqinr)
library(pheatmap)
library(pracma)
library(plyr)
library(ggplot2)
library(BiocInstaller)
library(RColorBrewer)
library(microbiome)
library(hablar)
library(digest)
library(MASS)
library(mvabund)
library(reshape2)
library(GGally)
library(vegan)
library(tidyr)
library(pillar)
library(tibble)
library(phyloseq)
library(data.table)
library(stringi)
library(fansi)
library(nlme)
library(fossil)
library(dplyr)
library(RLRsim)
library(ggfortify)
library(anchors)
library(decontam); packageVersion("decontam")
library(arm)
library(glmmTMB)
library(purrr)
```

############Save myEnvironment


```
#save.image(file='myEnvironment.Dolphinblow_5.RData')
load('myEnvironment.Dolphinblow_5.RData')
```

############Read in datasets


```
###zOTU table
dat.dol.full = read.csv("AllSamples_unoise_otu_table.Silva.mod_2.csv")

#Metadata
DolBlow_var = read.csv("DolBlow_var.3.csv", header=T,sep=',')
```

############Process datasets


```
dim(dat.dol_t.12)
```


```
[1]  129 2150
```


###Determine richness and number of reads of tech controls


```
dim(dat.dol_t.9)
#124 2148

#Transpose dat.dol_t.9
dat.dol_t.t = as.data.frame(t(dat.dol_t.9))
#View(dat.dol_t.t[,1:10])
#names(dat.dol_t.t)

#Keep cols with tech controls only
dat.dol_t.t.tech = dat.dol_t.t[,c(81:86,108:111,115:117)]
#names(dat.dol_t.t.tech)
dim(dat.dol_t.t.tech)
#2148   13

DolBlow_var$Dolphin

DolBlow_var.tech = DolBlow_var[c(16:19,67:73,83:84),2:3]
dim(DolBlow_var.tech)
#13  2

DolBlow_var.tech.2 = DolBlow_var.tech
#rownames(DolBlow_var.tech.2) = DolBlow_var.tech.2$Dolphin

dat.dol_t.t.tech.2 = dat.dol_t.t.tech
dat.dol_t.t.tech.2$variable = rownames(dat.dol_t.t.tech.2)
dim(dat.dol_t.t.tech.2)
#2148   14

dat.dol_t.t.tech.3 = dat.dol_t.t.tech.2 %>% 
  mutate(sum_of_rows = rowSums(dat.dol_t.t.tech.2[,1:13]))

dat.dol_t.t.tech.4 = dat.dol_t.t.tech.3 %>% filter (sum_of_rows > 0)

dim(dat.dol_t.t.tech.4)
# 385 15

zOTUs.tech = dat.dol_t.t.tech.4$variable

###zOTUs that are present in tech. controls
zOTUs.tech
length(zOTUs.tech)
#385
```


###Create phyloseq-class object to filter technical contaminants


```
###################
###Metadata: DolBlow_var

#Provide DolBlow_var with col 'Sample_or_Control', remove all water samples, and rename col 'Dolphin' to X.SampleID
DolBlow_var_no.water <- DolBlow_var %>% filter(!Species == 'Water')
#View(DolBlow_var_no.water)

Sample_or_Control <- as.data.frame(c(rep('Control', 4), rep('True Sample', 37), rep('Control', 7), rep('True Sample', 9), rep('Control', 2), rep('True Sample', 37)))

names(Sample_or_Control) <- 'Sample_or_Control'

DolBlow_var_no.water.2 <- cbind(Sample_or_Control, DolBlow_var_no.water)
#View(DolBlow_var_no.water.2)

colnames(DolBlow_var_no.water.2)[4] <- 'X.SampleID'

#Leave in cols 'Sample_or_Control' and 'X.SampleID' only
DolBlow_var_no.water.3 <- DolBlow_var_no.water.2[,c(1,4)]
#View(DolBlow_var_no.water.3)
DolBlow_var_no.water.3 <- as.data.frame(DolBlow_var_no.water.3)

rownames(DolBlow_var_no.water.3) <- DolBlow_var_no.water.3$X.SampleID
```

###Delete surplus zOTUs from taxonomy table: attributes.dol\_rdp (mitochondria, chloroplasts)


```
dim(attributes.dol_rdp)
#2282    7

attributes.dol_rdp.2 <- subset(attributes.dol_rdp, variable %in% zOTUs.abund)
dim(attributes.dol_rdp.2)
#2148    7
```


###Create phyloseq-class object to filter (zOTU table with count data, not relative abundance)


```
###zOTU table

#View(dat.dol_t.10[,1:10])
dim(dat.dol_t.10)
#124 2149

#Delete water samples
#rownames(dat.dol_t.10)
dat.dol_t.no.water <- dat.dol_t.10[-c(90:107,112:114,118:124),]
dim(dat.dol_t.no.water)
#96 2149

#View(dat.dol_t.no.water[,2140:2149])

#Delete col dolphin
dat.dol_t.no.water.2 <- dat.dol_t.no.water[,-2149]

#transpose dat.dol_t.no.water.2 -> dat.dol_t.no.water.2.t
dat.dol_t.no.water.2.t <- as.data.frame(t(dat.dol_t.no.water.2))
#names(dat.dol_t.no.water.2.t)
#rownames(dat.dol_t.no.water.2.t)

dim(dat.dol_t.no.water.2.t)
#2148    96

#Sort attributes.dol_rdp.2 by $variable
attributes.dol_rdp.3 <- attributes.dol_rdp.2[order(attributes.dol_rdp.2$variable),] 
#View(attributes.dol_rdp.3)

DolBlow_var_no.water.4 <- DolBlow_var_no.water.3

rownames(DolBlow_var_no.water.4) <- DolBlow_var_no.water.4$X.SampleID
```

### Create phyloseq-class object to filter technical controls


```
## These files need to become matrix files to work with phyloseq
dolphin_otu_nm = as.matrix(sapply(dat.dol_t.no.water.2.t, as.numeric))
dolphin_tax_cm = as.matrix(sapply(attributes.dol_rdp.3, as.character))

## Convert files into phyloseq object types
zOTU = otu_table(dolphin_otu_nm, taxa_are_rows = TRUE)
TAX = tax_table(dolphin_tax_cm)
dolphindata = sample_data(data.frame(DolBlow_var_no.water.4, stringsAsFactors = F))

## merge the files with phyloseq
dolphin1 = phyloseq(zOTU, TAX, dolphindata)

# phyloseq-class experiment-level object

# phyloseq-class experiment-level object
# otu_table()   OTU Table:         [ 2148 taxa and 96 samples ]
# sample_data() Sample Data:       [ 96 samples by 2 sample variables ]
# tax_table()   Taxonomy Table:    [ 2148 taxa by 7 taxonomic ranks ]

head(sample_data(dolphin1))

# Put sample_data into a ggplot-friendly data.frame
df<- as.data.frame(sample_data(dolphin1))

#Check library size
df$LibrarySize <- sample_sums(dolphin1)
df<- df[order(df$LibrarySize),]
df$Index <- seq(nrow(df))
ggplot(data=df, aes(x=Index, y=LibrarySize, color=Sample_or_Control)) + geom_point() + geom_jitter()
```


###Identify Contaminants with decontam-method Prevalence #decontam: https://benjjneb.github.io/decontam/vignettes/decontam\_intro.html


```
sample_data(dolphin1)$is.neg <- sample_data(dolphin1)$Sample_or_Control == "Control"

###################
###threshold=0.5
contamdf.prev05  <- isContaminant(dolphin1, method="prevalence", neg="is.neg", threshold=0.5)
table(contamdf.prev05$contaminant)

#--> False are non-contaminants!!!
#FALSE  TRUE 
# 1991     157

# Make phyloseq object of presence-absence in negative controls and true samples
ps.pa <- transform_sample_counts(dolphin1, function(abund) 1*(abund>0))

ps.pa.neg <- prune_samples(sample_data(ps.pa)$Sample_or_Control == "Control", ps.pa)

ps.pa.pos <- prune_samples(sample_data(ps.pa)$Sample_or_Control == "True Sample", ps.pa)

# Make data.frame of prevalence in positive and negative samples
df.pa05 <- data.frame(pa.pos=taxa_sums(ps.pa.pos), pa.neg=taxa_sums(ps.pa.neg),
                    contaminant=contamdf.prev05$contaminant)

decontam.plot.0.5 <- ggplot(data=df.pa05, aes(x=pa.neg, y=pa.pos, color=contaminant)) + geom_point() +
  xlab("Prevalence (Negative Controls)") + ylab("Prevalence (True Samples)") + geom_jitter()

ggsave("decontam.plot.05.jpg", plot = decontam.plot.0.5 , device = 'jpg', width = 168, height = 130, units = "mm",
       dpi = 300, limitsize = TRUE) 

###Frequency scores of zOTUs 
hist(contamdf.prev05$p)
decontam.freq.score <- ggplot(contamdf.prev05, aes(p)) + geom_histogram(binwidth = 0.1)

ggsave("decontam.freq.score.05.jpg", plot = decontam.freq.score , device = 'jpg', width = 168, height = 130, units = "mm",
       dpi = 300, limitsize = TRUE)
```


###After determining the contaminant zOTUs, we delete them from the data set dat.dol\_t.no.water.2.t


```
###contamdf.prev05 contains the list of contaminant zOTUs

###Extract col $contamint from contamdf.prev05 and combine with #dat.dol_t.no.water.2.t
###dat.dol_t.no.water.2.t
dim(dat.dol_t.no.water.2.t)
#2148   96

zOTU_fil.progress <- contamdf.prev05$contaminant

dat.dol_t.no.water.2.t.2 <- cbind(zOTU_fil.progress,dat.dol_t.no.water.2.t)
dim(dat.dol_t.no.water.2.t.2)
#2148   97

#View(dat.dol_t.no.water.2.t.2[1:10,])

###Only keep zOTUS == False (non-contaminants)

dat.dol_t.no.water.2.t.3 <- dat.dol_t.no.water.2.t.2

dat.dol_t.no.water.2.t.3$variable <- rownames(dat.dol_t.no.water.2.t.3)
dim(dat.dol_t.no.water.2.t.3)
#2148   98

dat.dol_fil <- dat.dol_t.no.water.2.t.3 %>% filter (!zOTU_fil.progress == 'TRUE')
dim(dat.dol_fil)
#1991   98

#Delete col $zOTU_fil.progress, bring col $variable to the front
dat.dol_fil.2 <- dat.dol_fil[,c(98, 2:97)]
#View(dat.dol_fil.2[1:10,])

#Transpose once more
dat.dol_fil.3 <- dat.dol_fil.2
rownames(dat.dol_fil.3) <- dat.dol_fil.3$variable
dat.dol_fil.4 <- dat.dol_fil.3[,-1]

dat.dol_fil.t <- as.data.frame(t(dat.dol_fil.4))
dim(dat.dol_fil.t)
#96 1991
#View(dat.dol_fil.t[,1:10])

#Bring in order: Dolphin samples on top, controls at bottom
dat.dol_fil.t.2 <- dat.dol_fil.t[c(1:80,87:89,81:86,90:96),]
#rownames(dat.dol_fil.t.2)

#Delete Rows with controls:
dat.dol_fil.t.3 <- dat.dol_fil.t.2[1:83,]
#rownames(dat.dol_fil.t.3)
dim(dat.dol_fil.t.3)
#83 1991
```

###Delete all technical contaminant zOTUs in attributes.dol\_rdp.3


```
###attributes.dol_rdp.3

###Extract col $contamint from contamdf.prev05 and combine with #dat.dol_t.no.water.2.t and attributes.dol_rdp.3

dim(attributes.dol_rdp.3)
#2148   7
#View(attributes.dol_rdp.3)

attributes.dol_rdp.4 <- cbind(zOTU_fil.progress,attributes.dol_rdp.3)
dim(attributes.dol_rdp.4)
#2148   8

#View(attributes.dol_rdp.4)

###Only keep zOTUS == False (non-contaminants)
attributes.dol_rdp.5 <- attributes.dol_rdp.4

attributes.dol_rdp_fil <- attributes.dol_rdp.5 %>% filter (!zOTU_fil.progress == 'TRUE')
dim(attributes.dol_rdp_fil)
#1991   8

#Delete col $zOTU_fil.progress
attributes.dol_rdp_fil.2 <- attributes.dol_rdp_fil[,-1]
#View(attributes.dol_rdp_fil.2)
```

###Create table with tech contaminants zOTUs that were deleted


```
###Only keep zOTUS == TRUE (contaminants)
attributes.dol_rdp_conts <- attributes.dol_rdp.5 %>% filter (zOTU_fil.progress == 'TRUE')
dim(attributes.dol_rdp_conts)
#157   8

###Delete col 1 & 8
attributes.dol_rdp_conts.2 <- attributes.dol_rdp_conts[,-c(1,8)]

###Save as csv-file (for supplements)
write.csv(file = 'attributes.dol_rdp_conts.2.csv', attributes.dol_rdp_conts.2)
```

###Delete all control samples in DolBlow\_var\_no.water.4


```
#View(DolBlow_var.2)
DolBlow_var.fil <- DolBlow_var.2 %>% filter (!Species == 'tech.control')
dim(DolBlow_var.fil)
#111   3

###Delete col 1 $Sample
DolBlow_var.fil.2 <- DolBlow_var.fil[,-1]
```

###Combine zOTUs tables of dolphin samples and water samples again


#########What’s the average number of reads per samples?


```
dim(dat.dol_2.fil.t)
#111 1991

dat.dol_2.fil.t_10 = mutate (dat.dol_2.fil.t, sum_of_rows=rowSums(dat.dol_2.fil.t))

#View(dat.dol_2.fil.t_10)
mean(dat.dol_2.fil.t_10$sum_of_rows)
sd(dat.dol_2.fil.t_10$sum_of_rows)
```

############Beta Diversity of dolphin samples vs pool samples


###############Unifrac

#######Rarefaction curves and Good’s coverage of dolphins and pool water samples (before filtering out pool water zOTUs) ###Prepare datasets


```
#dat.dol_2.fil.t, DolBlow_var.fil.2, attributes.dol_rdp_fil.2
#View(dat.dol_2.fil.t)
#View(DolBlow_var.fil.2)
#View(attributes.dol_rdp_fil.2)

###Give attributes.dol_rdp_fil.2 rownames and delete last column
attributes.dol_rdp_fil.3 <- attributes.dol_rdp_fil.2 [, -7]
rownames(attributes.dol_rdp_fil.3) <- attributes.dol_rdp_fil.3$variable

rownames(DolBlow_var.fil.2) = DolBlow_var.fil.2$Whale

#Transpose dat.dol_2.fil.t
dat.dol_2.fil.t_t = as.data.frame(t(dat.dol_2.fil.t))
```


#######Rarefaction curves: function


```
require(parallel)
ggrare <- function(physeq, step = 10, label = NULL, color = NULL, plot = TRUE, parallel = FALSE, se = TRUE) {
  x <- as(otu_table(physeq), "matrix")
  if (taxa_are_rows(physeq)) { x <- t(x) }
  
  ## This script is adapted from vegan `rarecurve` function
  tot <- rowSums(x)
  S <- rowSums(x > 0)
  nr <- nrow(x)
  
  rarefun <- function(i) {
    cat(paste("rarefying sample", rownames(x)[i]), sep = "\n")
    n <- seq(1, tot[i], by = step)
    if (n[length(n)] != tot[i]) {
      n <- c(n, tot[i])
    }
    y <- rarefy(x[i, ,drop = FALSE], n, se = se)
    if (nrow(y) != 1) {
      rownames(y) <- c(".S", ".se")
      return(data.frame(t(y), Size = n, Sample = rownames(x)[i]))
    } else {
      return(data.frame(.S = y[1, ], Size = n, Sample = rownames(x)[i]))
    }
  }
  if (parallel) {
    out <- mclapply(seq_len(nr), rarefun, mc.preschedule = FALSE)
  } else {
    out <- lapply(seq_len(nr), rarefun)
  }
  df <- do.call(rbind, out)
  
  ## Get sample data 
  if (!is.null(sample_data(physeq, FALSE))) {
    sdf <- as(sample_data(physeq), "data.frame")
    sdf$Sample <- rownames(sdf)
    data <- merge(df, sdf, by = "Sample")
    labels <- data.frame(x = tot, y = S, Sample = rownames(x))
    labels <- merge(labels, sdf, by = "Sample")
  }
  
  ## Add, any custom-supplied plot-mapped variables
  if( length(color) > 1 ){
    data$color <- color
    names(data)[names(data)=="color"] <- deparse(substitute(color))
    color <- deparse(substitute(color))
  }
  if( length(label) > 1 ){
    labels$label <- label
    names(labels)[names(labels)=="label"] <- deparse(substitute(label))
    label <- deparse(substitute(label))
  }
  
  p <- ggplot(data = data, aes_string(x = "Size", y = ".S", group = "Sample", color = color)) +
    theme_bw() +
    theme(plot.title = element_text(size = 14, family = "Tahoma", face = "bold"),
          text = element_text(size = 12, family = "Tahoma"),
          axis.title = element_text(face="bold"),
          axis.text.x=element_text(size = 11)) +
    scale_fill_brewer(palette = "Accent") +
    theme(axis.text.x=element_text(angle=90))
  
  p <- p + labs(x = "Sample Size", y = "Species Richness")
  if (!is.null(label)) {
    p <- p + geom_text(data = labels, aes_string(x = "x", y = "y", label = label, color = color),
                       size = 4, hjust = 0)
  }
  p <- p + geom_line()
  if (se) { ## add standard error if available
    p <- p + geom_ribbon(aes_string(ymin = ".S - .se", ymax = ".S + .se", color = NULL, fill = color), alpha = 0.2)
  }
  if (plot) {
    plot(p)
  }
  invisible(p)
}

phylodiv <- function(physeq) {
  ## Args:
  ## - physeq: phyloseq class object, from which phylogeny and abundance data are extracted
  x <- as(otu_table(physeq), "matrix")
  if (taxa_are_rows(physeq)) { x <- t(x) }
  phy <- phy_tree(physeq)
  
  ## Construct incidence matrix of the tree
  incidence <- incidenceMatrix(phy)
  
  ## Order incidence matrix according to community tables
  incidence <- incidence[colnames(x), ]
  
  ## Create community phylogeny matrix by multiplying (community x edge matrix)
  ## where cpm_{ij} gives the abundance of OTUs originating from branch j in community i. 
  cpm <- x %*% incidence
  ## Convert to incidence matrix (0/1) and multiply by edge length to obtain PD per community.
  cpm[cpm > 0] <- 1
  pd <-  cpm %*% phy$edge.length
  
  ## Add sample data information
  if (!is.null(sample_data(physeq, FALSE))) {
    sdf <- as(sample_data(physeq), "data.frame")
    sdf$pd <- as.vector(pd)
    pd <- sdf
  }
  
  return (pd)
}
```


########Create rarefaction curves separately by dolphin and pool

###Coen


```
sd(goods.Coen$goods)
```


```
[1] 0.05015695
```


###Evie


```
mean(goods.Evie$goods)
```


```
[1] 99.79485
```


```
sd(goods.Evie$goods)
```


```
[1] 0.05164822
```


###Kiama


```
mean(goods.Kiama$goods)
```


```
[1] 99.82134
```


```
sd(goods.Kiama$goods)
```


```
[1] 0.1001233
```

###Moki


```
mean(goods.Moki$goods)
```


```
[1] 99.81388
```


```
sd(goods.Moki$goods)
```


```
[1] 0.1058761
```

###RB


```
mean(goods.RB$goods)
```


```
[1] 99.83142
```


```
sd(goods.RB$goods)
```


```
[1] 0.04426928
```

###Scooter


```
mean(goods.Scooter$goods)
```


```
[1] 99.88251
```


```
sd(goods.Scooter$goods)
```


```
[1] 0.1119296
```

###Sirius


```
mean(goods.Sirius$goods)
```


```
[1] 99.91981
```


```
sd(goods.Sirius$goods)
```


```
[1] 0.02734815
```

###Squeak


```
mean(goods.Squeak$goods)
```


```
[1] 99.8792
```


```
sd(goods.Squeak$goods)
```


```
[1] 0.06004199
```

###Starbuck


```
mean(goods.Starbuck$goods)
```


```
[1] 99.84276
```


```
sd(goods.Starbuck$goods)
```


```
[1] 0.03080761
```

###Gemma


```
mean(goods.Gemma$goods)
```


```
[1] 99.87807
```


```
sd(goods.Gemma$goods)
```


```
[1] 0.06908713
```

###Howie


```
mean(goods.Howie$goods)
```


```
[1] 99.81757
```


```
sd(goods.Howie$goods)
```


```
[1] 0.0730471
```

###Nudgee


```
mean(goods.Nudgee$goods)
```


```
[1] 99.89074
```


```
sd(goods.Nudgee$goods)
```


```
[1] 0.03996851
```

###Stella


```
mean(goods.Stella$goods)
```


```
[1] 99.88139
```


```
sd(goods.Stella$goods)
```


```
[1] 0.04454545
```

###Bay


```
###Bay

#dat.dol_2.fil.t_t, DolBlow_var.fil.2, attributes.dol_rdp_fil.3
#names(dat.dol_2.fil.t_t)
#DolBlow_var.fil.2$Dolphin

dat.dol_2.fil.t_t.Bay = dat.dol_2.fil.t_t[,c(1:6)]
DolBlow_var.fil.Bay = DolBlow_var.fil.2[c(1:6),]
rownames(DolBlow_var.fil.Bay) = DolBlow_var.fil.Bay$Dolphin

dat.dol_2.fil.t_t.Bay.2 = dat.dol_2.fil.t_t.Bay
dat.dol_2.fil.t_t.Bay.2$variable = rownames(dat.dol_2.fil.t_t.Bay.2)

dat.dol_2.fil.t_t.Bay.3 = dat.dol_2.fil.t_t.Bay.2 %>% 
                         mutate (sum_of_rows = rowSums(dat.dol_2.fil.t_t.Bay.2[,1:6]))

dat.dol_2.fil.t_t.Bay.4 = dat.dol_2.fil.t_t.Bay.3 %>% filter (sum_of_rows > 0)

dim(dat.dol_2.fil.t_t.Bay.4)
# 540   8

zOTUs.Bay = dat.dol_2.fil.t_t.Bay.4$variable

dat.dol_2.fil.t_t.Bay.5 = dat.dol_2.fil.t_t.Bay.4[,-c(7,8)]
rownames(dat.dol_2.fil.t_t.Bay.5) = dat.dol_2.fil.t_t.Bay.4$variable

attributes.dol_rdp_fil.3.Bay = attributes.dol_rdp_fil.3 [zOTUs.Bay,-1]

## they need to become matrix files to work with phyloseq
whale_otu_nm.Bay = as.matrix(sapply(dat.dol_2.fil.t_t.Bay.5, as.numeric)) 
whale_tax_cm.Bay = as.matrix(sapply(attributes.dol_rdp_fil.3.Bay, as.character))

DolBlow_var.fil.Bay$Species <- as.character(DolBlow_var.fil.Bay$Species)
DolBlow_var.fil.Bay$Dolphin <- as.character(DolBlow_var.fil.Bay$Dolphin)

## convert files into phyloseq object types
OTU.Bay = otu_table(whale_otu_nm.Bay, taxa_are_rows = TRUE)
TAX.Bay = tax_table(whale_tax_cm.Bay)
whaledata.Bay = sample_data(data.frame(DolBlow_var.fil.Bay, stringsAsFactors = F))

## merge the files with phyloseq
Dolphin.Bay = phyloseq(OTU.Bay, TAX.Bay, whaledata.Bay)

Dolphin.Bay.2 <- methods::as(phyloseq::otu_table(Dolphin.Bay), "matrix")

if (phyloseq::taxa_are_rows(Dolphin.Bay)) { Dolphin.Bay.2 <- t(Dolphin.Bay.2) }

specnumber(Dolphin.Bay.2)

#Rarefaction curves
p.Bay = ggrare(Dolphin.Bay, step = 100, color = "Dolphin", se = FALSE)

p.Bay_2 = p.Bay + facet_wrap(~Species)

ggsave("rarefaction.curves.Bay.jpg", plot = p.Bay_2 , device = 'jpg', width = 168, height = 130, units = "mm",
       dpi = 300, limitsize = TRUE)

##########################
##Get good's coverage
## need samples as rows
t.OTU.table.Bay = t(otu_table(Dolphin.Bay)) # transpose the table
sample_data(Dolphin.Bay)

#devtools::install_github("jfq3/QsRutils")
library(QsRutils)

goods.Bay = goods(otu_table(t.OTU.table.Bay))

var.Bay = sample_data(Dolphin.Bay)
goods_var.Bay = cbind(goods.Bay,  var.Bay)

goods.Bay

mean(goods.Bay$goods)

sd(goods.Bay$goods)
```

###Beach


```
###Beach

#dat.dol_2.fil.t_t, DolBlow_var.fil.2, attributes.dol_rdp_fil.3
#names(dat.dol_2.fil.t_t)
#DolBlow_var.fil.2$Dolphin

dat.dol_2.fil.t_t.Beach = dat.dol_2.fil.t_t[,c(7:15)]
DolBlow_var.fil.Beach = DolBlow_var.fil.2[c(7:15),]
rownames(DolBlow_var.fil.Beach) = DolBlow_var.fil.Beach$Dolphin

dat.dol_2.fil.t_t.Beach.2 = dat.dol_2.fil.t_t.Beach
dat.dol_2.fil.t_t.Beach.2$variable = rownames(dat.dol_2.fil.t_t.Beach.2)

dat.dol_2.fil.t_t.Beach.3 = dat.dol_2.fil.t_t.Beach.2 %>% 
                         mutate (sum_of_rows = rowSums(dat.dol_2.fil.t_t.Beach.2[,1:9]))

dat.dol_2.fil.t_t.Beach.4 = dat.dol_2.fil.t_t.Beach.3 %>% filter (sum_of_rows > 0)

dim(dat.dol_2.fil.t_t.Beach.4)
# 540   8

zOTUs.Beach = dat.dol_2.fil.t_t.Beach.4$variable

dat.dol_2.fil.t_t.Beach.5 = dat.dol_2.fil.t_t.Beach.4[,-c(10,11)]
rownames(dat.dol_2.fil.t_t.Beach.5) = dat.dol_2.fil.t_t.Beach.4$variable

attributes.dol_rdp_fil.3.Beach = attributes.dol_rdp_fil.3 [zOTUs.Beach,-1]

## they need to become matrix files to work with phyloseq
whale_otu_nm.Beach = as.matrix(sapply(dat.dol_2.fil.t_t.Beach.5, as.numeric)) 
whale_tax_cm.Beach = as.matrix(sapply(attributes.dol_rdp_fil.3.Beach, as.character))

DolBlow_var.fil.Beach$Species <- as.character(DolBlow_var.fil.Beach$Species)
DolBlow_var.fil.Beach$Dolphin <- as.character(DolBlow_var.fil.Beach$Dolphin)

## convert files into phyloseq object types
OTU.Beach = otu_table(whale_otu_nm.Beach, taxa_are_rows = TRUE)
TAX.Beach = tax_table(whale_tax_cm.Beach)
whaledata.Beach = sample_data(data.frame(DolBlow_var.fil.Beach, stringsAsFactors = F))

## merge the files with phyloseq
Dolphin.Beach = phyloseq(OTU.Beach, TAX.Beach, whaledata.Beach)

Dolphin.Beach.2 <- methods::as(phyloseq::otu_table(Dolphin.Beach), "matrix")

if (phyloseq::taxa_are_rows(Dolphin.Beach)) { Dolphin.Beach.2 <- t(Dolphin.Beach.2) }

specnumber(Dolphin.Beach.2)

#Rarefaction curves
p.Beach = ggrare(Dolphin.Beach, step = 100, color = "Dolphin", se = FALSE)

p.Beach_2 = p.Beach + facet_wrap(~Species)

ggsave("rarefaction.curves.Beach.jpg", plot = p.Beach_2 , device = 'jpg', width = 168, height = 130, units = "mm",
       dpi = 300, limitsize = TRUE)

##########################
##Get good's coverage
## need samples as rows
t.OTU.table.Beach = t(otu_table(Dolphin.Beach)) # transpose the table
sample_data(Dolphin.Beach)

#devtools::install_github("jfq3/QsRutils")
library(QsRutils)

goods.Beach = goods(otu_table(t.OTU.table.Beach))

var.Beach = sample_data(Dolphin.Beach)
goods_var.Beach = cbind(goods.Beach,  var.Beach)

goods.Beach

mean(goods.Beach$goods)

sd(goods.Beach$goods)
```

###QVC


```
###QVC

#dat.dol_2.fil.t_t, DolBlow_var.fil.2, attributes.dol_rdp_fil.3
#names(dat.dol_2.fil.t_t)
#DolBlow_var.fil.2$Dolphin

dat.dol_2.fil.t_t.QVC = dat.dol_2.fil.t_t[,c(72:74)]
DolBlow_var.fil.QVC = DolBlow_var.fil.2[c(72:74),]
rownames(DolBlow_var.fil.QVC) = DolBlow_var.fil.QVC$Dolphin

dat.dol_2.fil.t_t.QVC.2 = dat.dol_2.fil.t_t.QVC
dat.dol_2.fil.t_t.QVC.2$variable = rownames(dat.dol_2.fil.t_t.QVC.2)

dat.dol_2.fil.t_t.QVC.3 = dat.dol_2.fil.t_t.QVC.2 %>% 
                         mutate (sum_of_rows = rowSums(dat.dol_2.fil.t_t.QVC.2[,1:3]))

dat.dol_2.fil.t_t.QVC.4 = dat.dol_2.fil.t_t.QVC.3 %>% filter (sum_of_rows > 0)

dim(dat.dol_2.fil.t_t.QVC.4)
# 540   8

zOTUs.QVC = dat.dol_2.fil.t_t.QVC.4$variable

dat.dol_2.fil.t_t.QVC.5 = dat.dol_2.fil.t_t.QVC.4[,-c(4,5)]
rownames(dat.dol_2.fil.t_t.QVC.5) = dat.dol_2.fil.t_t.QVC.4$variable

attributes.dol_rdp_fil.3.QVC = attributes.dol_rdp_fil.3 [zOTUs.QVC,-1]

## they need to become matrix files to work with phyloseq
whale_otu_nm.QVC = as.matrix(sapply(dat.dol_2.fil.t_t.QVC.5, as.numeric)) 
whale_tax_cm.QVC = as.matrix(sapply(attributes.dol_rdp_fil.3.QVC, as.character))

DolBlow_var.fil.QVC$Species <- as.character(DolBlow_var.fil.QVC$Species)
DolBlow_var.fil.QVC$Dolphin <- as.character(DolBlow_var.fil.QVC$Dolphin)

## convert files into phyloseq object types
OTU.QVC = otu_table(whale_otu_nm.QVC, taxa_are_rows = TRUE)
TAX.QVC = tax_table(whale_tax_cm.QVC)
whaledata.QVC = sample_data(data.frame(DolBlow_var.fil.QVC, stringsAsFactors = F))

## merge the files with phyloseq
Dolphin.QVC = phyloseq(OTU.QVC, TAX.QVC, whaledata.QVC)

Dolphin.QVC.2 <- methods::as(phyloseq::otu_table(Dolphin.QVC), "matrix")

if (phyloseq::taxa_are_rows(Dolphin.QVC)) { Dolphin.QVC.2 <- t(Dolphin.QVC.2) }

specnumber(Dolphin.QVC.2)

#Rarefaction curves
p.QVC = ggrare(Dolphin.QVC, step = 100, color = "Dolphin", se = FALSE)

p.QVC_2 = p.QVC + facet_wrap(~Species)

ggsave("rarefaction.curves.QVC.jpg", plot = p.QVC_2 , device = 'jpg', width = 168, height = 130, units = "mm",
       dpi = 300, limitsize = TRUE)

##########################
##Get good's coverage
## need samples as rows
t.OTU.table.QVC = t(otu_table(Dolphin.QVC)) # transpose the table
sample_data(Dolphin.QVC)

#devtools::install_github("jfq3/QsRutils")
library(QsRutils)

goods.QVC = goods(otu_table(t.OTU.table.QVC))

var.QVC = sample_data(Dolphin.QVC)
goods_var.QVC = cbind(goods.QVC,  var.QVC)

goods.QVC

mean(goods.QVC$goods)

sd(goods.QVC$goods)
```


###Test if microbiota of water samples and dolphin samples are significantly different from eachother


```
###################
###Create zOTU datatable with cols of Species, X.SampleID and Sample_or_Control.2 (also used for filtering of water zOTUs in later step)

#Metadata: DolBlow_var.fil.2

#Provide DolBlow_var.fil.2 with col 'Sample_or_Control', and rename col 'Dolphin' to X.SampleID
#DolBlow_var.fil.2$Dolphin

Sample_or_Control.2 <- as.data.frame(c(rep('Control', 15), rep('True Sample', 6), rep('Control', 10), rep('True Sample', 80)))
dim(Sample_or_Control.2)
#111  1

names(Sample_or_Control.2) <- 'Sample_or_Control.2'

DolBlow_var.fil.3 <- cbind(Sample_or_Control.2, DolBlow_var.fil.2)
#View(DolBlow_var.fil.3)

colnames(DolBlow_var.fil.3)[3] <- 'X.SampleID'

#Leave in cols 'Sample_or_Control.2' and 'X.SampleID' only
DolBlow_var.fil.4 <- DolBlow_var.fil.3[,c(-2)]
#View(DolBlow_var.fil.4)

rownames(DolBlow_var.fil.4) <- DolBlow_var.fil.4$c
```


```
###Merge DolBlow_var.fil.3 and dat.dol_2.fil.t
dim(dat.dol_2.fil.t)
```


```
Error: object 'dat.dol_2.fil.t' not found
```


###Create phyloseq-class object to filter out pool water contaminants


```
###Use DolBlow_var.fil.4, attributes.dol_rdp_fil.2 & dat.dol_t.19.t.fil.2

## These files need to become matrix files to work with phyloseq
dolphin_otu_nm.w = as.matrix(sapply(dat.dol_t.19.t.fil.2, as.numeric))
dolphin_tax_cm.w = as.matrix(sapply(attributes.dol_rdp_fil.2, as.character))

## Convert files into phyloseq object types
zOTU.w = otu_table(dolphin_otu_nm.w, taxa_are_rows = TRUE)
TAX.w = tax_table(dolphin_tax_cm.w)
dolphindata.w = sample_data(data.frame(DolBlow_var.fil.4, stringsAsFactors = F))

## merge the files with phyloseq
dolphin1.w = phyloseq(zOTU.w, TAX.w, dolphindata.w)

# phyloseq-class experiment-level object

# otu_table()   OTU Table:         [ 1991 taxa and 111 samples ]
# sample_data() Sample Data:       [ 111 samples by 2 sample variables ]
# tax_table()   Taxonomy Table:    [ 1991 taxa by 7 taxonomic ranks ]

head(sample_data(dolphin1.w))

# Put sample_data into a ggplot-friendly data.frame
df.w<- as.data.frame(sample_data(dolphin1.w))

#Check library size
df.w$LibrarySize <- sample_sums(dolphin1.w)
df.w<- df[order(df.w$LibrarySize),]
df.w$Index <- seq(nrow(df.w))
ggplot(data=df, aes(x=Index, y=LibrarySize, color=Sample_or_Control)) + geom_point() + geom_jitter()
```

###Identify Contaminants with decontam-method Prevalence #decontam: https://benjjneb.github.io/decontam/vignettes/decontam\_intro.html


```
sample_data(dolphin1.w)$is.neg <- sample_data(dolphin1.w)$Sample_or_Control.2 == "Control"

###################
###threshold=0.5
contamdf.prev05.w  <- isContaminant(dolphin1.w, method="prevalence", neg="is.neg", threshold=0.5)
table(contamdf.prev05.w$contaminant)

#--> False are non-contaminants!!!
#FALSE  TRUE 
# 1471   520

# Make phyloseq object of presence-absence in negative controls and true samples
ps.pa.w <- transform_sample_counts(dolphin1.w, function(abund) 1*(abund>0))

ps.pa.neg.w <- prune_samples(sample_data(ps.pa.w)$Sample_or_Control.2 == "Control", ps.pa.w)

ps.pa.pos.w <- prune_samples(sample_data(ps.pa.w)$Sample_or_Control.2 == "True Sample", ps.pa.w)

# Make data.frame of prevalence in positive and negative samples
df.pa05.w <- data.frame(pa.pos.w=taxa_sums(ps.pa.pos.w), pa.neg.w=taxa_sums(ps.pa.neg.w),
                    contaminant=contamdf.prev05.w$contaminant)

decontam.plot.0.5.w <- ggplot(data=df.pa05.w, aes(x=pa.neg.w, y=pa.pos.w, color=contaminant)) + geom_point() +
  xlab("Prevalence (Negative Controls)") + ylab("Prevalence (True Samples)") + geom_jitter()

ggsave("decontam.plot.0.5.w.jpg", plot = decontam.plot.0.5.w , device = 'jpg', width = 168, height = 130, units = "mm",
       dpi = 300, limitsize = TRUE) 

###Frequency scores of zOTUs 
hist(contamdf.prev05.w$p)
decontam.freq.score.w <- ggplot(contamdf.prev05.w, aes(p)) + geom_histogram(binwidth = 0.1)

ggsave("decontam.freq.score.05.w.jpg", plot = decontam.freq.score.w , device = 'jpg', width = 168, height = 130, units = "mm",
       dpi = 300, limitsize = TRUE)
```


###After determining the pool water zOTUs, we delete them from the data set #dat.dol\_t.no.water.2.t


```
###contamdf.prev05.w contains the list of contaminant zOTUs

###Extract col $contamint from contamdf.prev05.w and combine with dat.dol_t.19.t.fil.2
#View(dat.dol_t.19.t.fil.2)
dim(dat.dol_t.19.t.fil.2)
#1991  111

zOTU_fil.water <- contamdf.prev05.w$contaminant

dat.dol_t.19.t.fil.w <- cbind(zOTU_fil.water,dat.dol_t.19.t.fil.2)
dim(dat.dol_t.19.t.fil.w)
#1991  112

#View(dat.dol_t.19.t.fil.w[1:10,])

###Only keep zOTUS == False (non-contaminants)

dat.dol_t.19.t.fil.w.2 <- dat.dol_t.19.t.fil.w

dat.dol_t.19.t.fil.w.2$variable <- rownames(dat.dol_t.19.t.fil.w.2)
dim(dat.dol_t.19.t.fil.w.2)
#1991  113

dat.dol_water.fil <- dat.dol_t.19.t.fil.w.2 %>% filter (!zOTU_fil.water == 'TRUE')
dim(dat.dol_water.fil)
#1471  113

#Delete col $zOTU_fil.water, bring col $variable to the front
dat.dol_water.fil.2 <- dat.dol_water.fil[,c(113, 2:112)]
#View(dat.dol_water.fil.2[1:10,])

#Transpose once more
dat.dol_water.fil.3 <- dat.dol_water.fil.2
rownames(dat.dol_water.fil.3) <- dat.dol_water.fil.3$variable
dat.dol_water.fil.4 <- dat.dol_water.fil.3[,-1]

dat.dol_water.fil.t <- as.data.frame(t(dat.dol_water.fil.4))
dim(dat.dol_water.fil.t)
#111 1471
#View(dat.dol_water.fil.t[,1:10])
#rownames(dat.dol_water.fil.t)

#Delete Rows with controls:
dat.dol_water.fil.t.2 <- dat.dol_water.fil.t[c(16:21,32:71,75:111),]
#rownames(dat.dol_water.fil.t.2)
dim(dat.dol_water.fil.t.2)
#83 1471
```

###Delete all water zOTUs in attributes.dol\_rdp\_fil.2


```
###attributes.dol_rdp_fil.2

###Extract col $contaminant from contamdf.prev05 and combine with #dat.dol_t.no.water.2.t and attributes.dol_rdp.3

dim(attributes.dol_rdp_fil.2)
#1991    7
#View(attributes.dol_rdp_fil.2)

attributes.dol_rdp_fil.3 <- cbind(zOTU_fil.water,attributes.dol_rdp_fil.2)
dim(attributes.dol_rdp_fil.3)
#1991   8

#View(attributes.dol_rdp_fil.3)

###Only keep zOTUS == False (non-contaminants)
attributes.dol_rdp_fil.4 <- attributes.dol_rdp_fil.3

attributes.dol_rdp_water.fil <- attributes.dol_rdp_fil.4 %>% filter (!zOTU_fil.water == 'TRUE')
dim(attributes.dol_rdp_water.fil)
#1471  8

#Delete col $zOTU_fil.water
attributes.dol_rdp_water.fil.2 <- attributes.dol_rdp_water.fil[,-1]
#View(attributes.dol_rdp_water.fil.2)
```

###Create table with pool water zOTUs that were deleted


```
###Only keep zOTUS == TRUE (contaminants)
attributes.dol_rdp_water.conts <- attributes.dol_rdp_fil.4 %>% filter (zOTU_fil.water == 'TRUE')
dim(attributes.dol_rdp_water.conts)
#520   8

###Delete col 1 & 8
attributes.dol_rdp_water.conts.2 <- attributes.dol_rdp_water.conts[,-c(1,8)]

###Save as csv-file (for supplements)
write.csv(file = 'attributes.dol_rdp_water.conts.2.csv', attributes.dol_rdp_water.conts.2)
```

###Delete all pool water samples in DolBlow\_var.fil.4


```
#View(DolBlow_var.2)
DolBlow_var.water.fil <- DolBlow_var.fil.4 %>% filter (!Sample_or_Control.2 == 'Control')
dim(DolBlow_var.water.fil)
#111   3

###Delete col 1 $Sample
DolBlow_var.water.fil.2 <- DolBlow_var.water.fil[,-1]
```

###Datasets without water zOTUs ###attributes.dol\_rdp\_water.fil.2, DolBlow\_var.water.fil.2, dat.dol\_water.fil.t.2 #################################

###Alpha diversity of dolphin samples without pool water contaminants


```
dim(dat.dol_water.fil.t.2)
#116 1887
#View(dat.dol_water.fil.t.2[,1:10])

dat.dol_water.fil.t.3 <- dat.dol_water.fil.t.2

dat.dol_water.fil.t.3$Dolphin <- rownames(dat.dol_water.fil.t.3)
dim(dat.dol_water.fil.t.3)
#83 1472
dat.dol_water.fil.t.4 <- dat.dol_water.fil.t.3[,c(1472, 1:1471)]


# Rarefaction
min(rowSums(dat.dol_water.fil.t.4[,2:1472]))
#1,327
#rownames(dat.dol_water.fil.t.4)

###Nudgee_24 has the lowest read count = 1327 --> delete for alpha diversity
dat.dol_water.fil.t.alpha = dat.dol_water.fil.t.4[-c(40),]
#87 1887

# Rarefaction
min(rowSums(dat.dol_water.fil.t.alpha[,2:1472]))
#16,578

###--> Smallest number of reads is 16,578.

#Delete col 1 for rarefaction
dat.dol_water.fil.t.alpha.2 = dat.dol_water.fil.t.alpha[,-c(1)]

dat.dol_water.fil.t.alpha.rff = rrarefy(x = dat.dol_water.fil.t.alpha.2, sample = 16578)
rowSums(dat.dol_water.fil.t.alpha.rff)

# Calculating alpha diversity
species_richness.water.fil = NULL
species_diversity.water.fil = NULL
species_estimator.water.fil = NULL

for(i in 1:100)
{
  print(i)
  dat.dol_water.fil.t.alpha.rff = rrarefy(x = dat.dol_water.fil.t.alpha.2, sample = 16578)
  species_richness.water.fil = rbind(species_richness.water.fil, specnumber(dat.dol_water.fil.t.alpha.rff))
  species_diversity.water.fil = rbind(species_diversity.water.fil, diversity(dat.dol_water.fil.t.alpha.rff))
  species_estimator.water.fil = rbind(species_estimator.water.fil, estimateR(dat.dol_water.fil.t.alpha.rff)[2,])
}
species_richness.water.fil_avg = colMeans(species_richness.water.fil)
species_diversity.water.fil_avg = log(colMeans(exp(species_diversity.water.fil)))
species_estimator.water.fil_avg = colMeans(species_estimator.water.fil)

alpha.dolphin.water.fil = data.frame(species_richness.water.fil_avg, species_diversity.water.fil_avg, species_estimator.water.fil_avg)

alpha.dolphin.water.fil.2 = alpha.dolphin.water.fil

###Join alpha.dolphin.water.fil.2 with DolBlow_var.tech.fil
alpha.dolphin.water.fil.2$Dolphin = rownames(alpha.dolphin.water.fil.2)

#mean richness.dol
mean(alpha.dolphin.water.fil.2[,1])

sd(alpha.dolphin.water.fil.2[,1])
```


##########Check richness between dolphin samples


```
###Use alpha.dolphin.water.fil.2
# Multiple rows per individual dolphin, one for each measurement occasion). 
# This should have a column that identifies the dolphin, a column that identifies 
# the time point, any dolphin characteristics that you want to adjust for (e.g. pool) 
# and a column with the outcome. In the following code, these are 
# called ID, time, pool and richness

#View(alpha.dolphin.water.fil.2)
names(alpha.dolphin.water.fil.2)[1:3] <- c('richness', 'diversity', 'species_estimator')

#View(DolBlow_var)
DolBlow_var_2 <- DolBlow_var[order(DolBlow_var$Species),]
DolBlow_var_3 <- DolBlow_var_2[,c(3,7)]

alpha.dolphin.water.fil.3 <- alpha.dolphin.water.fil.2
alpha.dolphin.water.fil.3$Dolphin.2 <- rownames(alpha.dolphin.water.fil.3)

#Join alpha.dolphin.water.fil.3 and DolBlow_var_3
alpha.dolphin.water.fil.4 <- alpha.dolphin.water.fil.3 %>% left_join(DolBlow_var_3)

#Separate col Dolphin
alpha.dolphin.water.fil.5 <- separate(alpha.dolphin.water.fil.4,Dolphin.2, c('ID','time'))
#View(alpha.dolphin.water.fil.5)

alpha.dolphin.water.fil.6 <- alpha.dolphin.water.fil.5
alpha.dolphin.water.fil.6$Water_sample.2 <- alpha.dolphin.water.fil.6$Water_sample

#Separate col Water_sample
alpha.dolphin.water.fil.7 <- separate(alpha.dolphin.water.fil.6,Water_sample, c('pool','pool.time'))
#View(alpha.dolphin.water.fil.7)

write.csv(alpha.dolphin.water.fil.7, file = 'alpha.diversity.dolphins.csv')

#names(alpha.dolphin.water.fil.7)

mean(alpha.dolphin.water.fil.7$richness)

sd(alpha.dolphin.water.fil.7$richness)

###Make sure all numerics are numerics
str(alpha.dolphin.water.fil.7)
alpha.dolphin.water.fil.7$time <- as.numeric(alpha.dolphin.water.fil.7$time)
```

###########Test differences in alpha-diversity ###Richness


```
###lme: Linear Mixed-Effects Models
#This generic function fits a linear mixed-effects model 
#in the formulation described in Laird and Ware (1982) but 
#allowing for nested random effects. The within-group errors 
#are allowed to be correlated and/or have unequal variances.

##########################
###Influence of time:
hist(alpha.dolphin.water.fil.7$richness)

alpha.dolphin.water.fil.7$time <- as.factor(alpha.dolphin.water.fil.7$time)

### Were there changes in richness over the sampling period within the individual dolphin?
lme.richness <- lme(richness ~ time, random = ~1|ID, data = alpha.dolphin.water.fil.7)

summary(lme.richness)
anova(lme.richness)

#             numDF denDF   F-value p-value
# (Intercept)     1    54 254.96180  <.0001
# time           15    54   0.93104  0.5368

###--> Time does no have an effect on richness in individual dolphins.


##########################
###Influence of ID:

# Model with random intercepts
model1 <- lme(richness ~ time, random = ~ 1 | ID, data = alpha.dolphin.water.fil.7,
              method = "REML")
# Model without random intercepts
model0 <- lm(richness ~ time, data = alpha.dolphin.water.fil.7)

# Observed likelihood ratio test statistic
obs.lr <- as.numeric(-2 * (logLik(model0, REML = TRUE) - logLik(model1, REML = TRUE)))

# Simulate data from the model without random effects
simdat <- simulate(model0, nsim = 10000, seed = 20201106)

# Function to fit both models and calculate the LR statistic
cmp_models <- function(y) {
  ndat <- cbind(alpha.dolphin.water.fil.7, y = y)
  mdl1 <- lme(y ~ time , random = ~ 1 | ID, data = ndat, method = "REML")
  mdl0 <- lm(y ~ time , data = ndat)
  a0 <- as.numeric(-2 * (logLik(mdl0, REML = TRUE) - logLik(mdl1, REML = TRUE)))
  a0
}

# Get parametric bootstrap test statistics
cmp0 <- map_dbl(simdat, cmp_models)
# Get parametric bootstrap p-value
para.p <- mean(c(cmp0, obs.lr) >= obs.lr)
para.p
#0.00149985
```

###Shannon-Wiener diversity


```
mean(alpha.dolphin.water.fil.7$diversity)

sd(alpha.dolphin.water.fil.7$diversity)

##########################
###Influence of time:
hist(alpha.dolphin.water.fil.7$diversity)

### Were there changes in diversity over the sampling period within the individual dolphin?
lme.diversity <- lme(diversity ~ time, random = ~1|ID, data = alpha.dolphin.water.fil.7)

summary(lme.diversity)
anova(lme.diversity)

#             numDF denDF   F-value p-value
# (Intercept)     1    54 1576.8116  <.0001
# time           15    54    1.6389  0.0938

###--> Time does no have an effect on diversity in individual dolphins.

##########################
###Influence of ID:

# Model with random intercepts
model1 <- lme(diversity ~ time, random = ~ 1 | ID, data = alpha.dolphin.water.fil.7,
              method = "REML")
# Model without random intercepts
model0 <- lm(diversity ~ time, data = alpha.dolphin.water.fil.7)

# Observed likelihood ratio test statistic
obs.lr <- as.numeric(-2 * (logLik(model0, REML = TRUE) - logLik(model1, REML = TRUE)))

# Simulate data from the model without random effects
simdat <- simulate(model0, nsim = 10000, seed = 20201106)

# Function to fit both models and calculate the LR statistic
cmp_models <- function(y) {
  ndat <- cbind(alpha.dolphin.water.fil.7, y = y)
  mdl1 <- lme(y ~ time , random = ~ 1 | ID, data = ndat, method = "REML")
  mdl0 <- lm(y ~ time , data = ndat)
  a0 <- as.numeric(-2 * (logLik(mdl0, REML = TRUE) - logLik(mdl1, REML = TRUE)))
  a0
}

# Get parametric bootstrap test statistics
cmp0 <- map_dbl(simdat, cmp_models)
# Get parametric bootstrap p-value
para.p <- mean(c(cmp0, obs.lr) >= obs.lr)
para.p
#0.01639836
```


###Chao1


```
###Chao1
#Calculate Chao1 on rarefied data
#Chao species estimator for abundance
#Use dat.dol_t.10.tech.fil.alpha.rff and DolBlow_var.tech.fil

Chao1.water.fil <- as_tibble(apply(dat.dol_water.fil.t.alpha.rff, 1, chao1))

Chao1.water.fil.2 <- cbind(rownames(dat.dol_water.fil.t.alpha.rff),Chao1.water.fil)

names(Chao1.water.fil.2)[1] <- 'Dolphin'
names(Chao1.water.fil.2)[2] <- 'Chao1'

#Join Chao1.water.fil.2 and alpha.dolphin.tech.fil.dol.7
alpha.dolphin.water.fil.8 <- alpha.dolphin.water.fil.7 %>% left_join(Chao1.water.fil.2)

mean(alpha.dolphin.water.fil.8$Chao1)

sd(alpha.dolphin.water.fil.8$Chao1)

##########################
###Influence of time:
hist(alpha.dolphin.water.fil.8$Chao1)

### Were there changes in Chao1 over the sampling period within the individual dolphin?
lme.Chao1 <- lme(Chao1 ~ time, random = ~1|ID, data = alpha.dolphin.water.fil.8)

summary(lme.Chao1)
anova(lme.Chao1)

#             numDF denDF   F-value p-value
#(Intercept)     1    54 283.47473  <.0001
#time           15    54   1.03026  0.4404

###--> Time does no have an effect on Chao1 in individual dolphins.

##########################
###Influence of ID:

# Model with random intercepts
model1 <- lme(Chao1 ~ time, random = ~ 1 | ID, data = alpha.dolphin.water.fil.8,
              method = "REML")
# Model without random intercepts
model0 <- lm(Chao1 ~ time, data = alpha.dolphin.water.fil.8)

# Observed likelihood ratio test statistic
obs.lr <- as.numeric(-2 * (logLik(model0, REML = TRUE) - logLik(model1, REML = TRUE)))

# Simulate data from the model without random effects
simdat <- simulate(model0, nsim = 10000, seed = 20201106)

# Function to fit both models and calculate the LR statistic
cmp_models <- function(y) {
  ndat <- cbind(alpha.dolphin.water.fil.8, y = y)
  mdl1 <- lme(y ~ time , random = ~ 1 | ID, data = ndat, method = "REML")
  mdl0 <- lm(y ~ time , data = ndat)
  a0 <- as.numeric(-2 * (logLik(mdl0, REML = TRUE) - logLik(mdl1, REML = TRUE)))
  a0
}

# Get parametric bootstrap test statistics
cmp0 <- map_dbl(simdat, cmp_models)
# Get parametric bootstrap p-value
para.p <- mean(c(cmp0, obs.lr) >= obs.lr)
para.p
```


###ACE


```
###################### ###################### 
###ACE
#Calculate ACE on rarefied data
#ACE species estimator for abundance

ACE.water.fil <- as_tibble(apply(dat.dol_water.fil.t.alpha.rff, 1, ACE))

ACE.water.fil.2 <- cbind(rownames(dat.dol_water.fil.t.alpha.rff),ACE.water.fil)

names(ACE.water.fil.2)[1] <- 'Dolphin'
names(ACE.water.fil.2)[2] <- 'ACE'

#Join ACE.tech.fil.2 and alpha.dolphin.tech.fil.dol.7
alpha.dolphin.water.fil.9 <- left_join(ACE.water.fil.2, alpha.dolphin.water.fil.7)

mean(alpha.dolphin.water.fil.9$ACE)

sd(alpha.dolphin.water.fil.9$ACE)

##########################
###Influence of time:
hist(alpha.dolphin.water.fil.9$ACE)

### Were there changes in ACE over the sampling period within the individual dolphin?
lme.ACE <- lme(ACE ~ time, random = ~1|ID, data = alpha.dolphin.water.fil.9)

summary(lme.ACE)
anova(lme.ACE)

#             numDF denDF   F-value p-value
#(Intercept)     1    54 256.49398  <.0001
#time           15    54   0.96807  0.4998

###--> Time does no have an effect on ACE in individual dolphins.

##########################
###Influence of ID:

# Model with random intercepts
model1 <- lme(ACE ~ time, random = ~ 1 | ID, data = alpha.dolphin.water.fil.9,
              method = "REML")
# Model without random intercepts
model0 <- lm(ACE ~ time, data = alpha.dolphin.water.fil.9)

# Observed likelihood ratio test statistic
obs.lr <- as.numeric(-2 * (logLik(model0, REML = TRUE) - logLik(model1, REML = TRUE)))

# Simulate data from the model without random effects
simdat <- simulate(model0, nsim = 10000, seed = 20201106)

# Function to fit both models and calculate the LR statistic
cmp_models <- function(y) {
  ndat <- cbind(alpha.dolphin.water.fil.9, y = y)
  mdl1 <- lme(y ~ time , random = ~ 1 | ID, data = ndat, method = "REML")
  mdl0 <- lm(y ~ time , data = ndat)
  a0 <- as.numeric(-2 * (logLik(mdl0, REML = TRUE) - logLik(mdl1, REML = TRUE)))
  a0
}

# Get parametric bootstrap test statistics
cmp0 <- map_dbl(simdat, cmp_models)
# Get parametric bootstrap p-value
para.p <- mean(c(cmp0, obs.lr) >= obs.lr)
para.p


write.csv(alpha.dolphin.water.fil.8, file <- 'alpha.dolphin.water.fil.8.csv')
write.csv(alpha.dolphin.water.fil.9, file <- 'alpha.dolphin.water.fil.9.csv')
```

###Create scatterplots of alpha diversity parameters of each dolphin across time

###Calculate average of alpha diversity parameters per dolphin


```
###Richness
alpha.dolphin.water.fil.dol.richness = alpha.dolphin.water.fil.9[,c(3,6)]
richness.dol.ave = aggregate(alpha.dolphin.water.fil.dol.richness[1],   list(alpha.dolphin.water.fil.dol.richness$ID), mean)
richness.dol.sd = aggregate(alpha.dolphin.water.fil.dol.richness[1], list(alpha.dolphin.water.fil.dol.richness$ID), sd)

###Diversity
alpha.dolphin.water.fil.dol.diversity = alpha.dolphin.water.fil.9[,c(4,6)]
diversity.dol.ave = aggregate(alpha.dolphin.water.fil.dol.diversity[1], list(alpha.dolphin.water.fil.dol.diversity$ID), mean)
diversity.dol.sd = aggregate(alpha.dolphin.water.fil.dol.diversity[1], list(alpha.dolphin.water.fil.dol.diversity$ID), sd)

###Chao1
alpha.dolphin.water.fil.8.Chao1 = alpha.dolphin.water.fil.8[,c(5,9)]
Chao1.dol.ave = aggregate(alpha.dolphin.water.fil.8.Chao1[2], list(alpha.dolphin.water.fil.8.Chao1$ID), mean)
Chao1.dol.sd = aggregate(alpha.dolphin.water.fil.8.Chao1[2], list(alpha.dolphin.water.fil.8.Chao1$ID), sd)

###ACE
alpha.dolphin.water.fil.ACE = alpha.dolphin.water.fil.9[,c(2,6)]
ACE.dol.ave = aggregate(alpha.dolphin.water.fil.ACE[1], list(alpha.dolphin.water.fil.ACE$ID), mean)
ACE.dol.sd = aggregate(alpha.dolphin.water.fil.ACE[1], list(alpha.dolphin.water.fil.ACE$ID), sd)

alpha.para.dol.2 = cbind(richness.dol.ave,richness.dol.sd,diversity.dol.ave,diversity.dol.sd,Chao1.dol.ave,Chao1.dol.sd,ACE.dol.ave,ACE.dol.sd)
write.csv(alpha.para.dol.2, file = 'alpha.para.dol.3.csv')
```

###Did time have an effect on the microbial communities in the dolphin blow?


```
###Fit the generalised linear models
zOTU.water.fil = mvabund(dat.dol_water.fil.t.9[,4:1474])

###Create variable for log of total abundance of zOTU's per sample
dat.dol_water.fil.t.10 = dat.dol_water.fil.t.9
dat.dol_water.fil.t.10$logTotalAbundance = log(apply(dat.dol_water.fil.t.10[,4:1474],1,sum))

Pre.fit1.water.fil.time = manyglm(zOTU.water.fil ~ time + ID + offset(logTotalAbundance), data = dat.dol_water.fil.t.10, family="negative.binomial")

#check assumptions
plot(Pre.fit1.water.fil.time)

#This is the actual significance test               
fit.1.water.fil.time.1 = manyglm(zOTU.water.fil ~ time + ID + offset(logTotalAbundance), data = dat.dol_water.fil.t.10)
fit.2.water.fil.time.2 = manyglm(zOTU.water.fil ~ ID + offset(logTotalAbundance), data = dat.dol_water.fil.t.10)

globalTest1.dol.water.fil.adjusted.time = anova(fit.1.water.fil.time.1, fit.2.water.fil.time.2, nBoot=1000, p.uni='adjusted')

# Analysis of Deviance Table
# 
# fit.2.water.fil.time.2: zOTU.water.fil ~ ID + offset(logTotalAbundance)
# fit.1.water.fil.time.1: zOTU.water.fil ~ time + ID + offset(logTotalAbundance)
# 
# Multivariate test:
#                        Res.Df Df.diff   Dev Pr(>Dev)   
# fit.2.water.fil.time.2     70                          
# fit.1.water.fil.time.1     55      15 20140    0.002 **
# ---
# Signif. codes:  0 ‘***’ 0.001 ‘**’ 0.01 ‘*’ 0.05 ‘.’ 0.1 ‘ ’ 1

#save(globalTest1.dol.water.fil.adjusted.time, file='globalTest1.dol.water.fil.adjusted.time.Rdata')
```


######This tells us that there is an impact of time########

############Did ID have an effect on the microbial communities in the dolphin blow?


```
#This is the actual significance test 
fit.1.water.fil.ID.1 = manyglm(zOTU.water.fil ~ time + ID + offset(logTotalAbundance), data = dat.dol_water.fil.t.10)
fit.2.water.fil.ID.2 = manyglm(zOTU.water.fil ~ time + offset(logTotalAbundance), data = dat.dol_water.fil.t.10)

globalTest1.dol.water.fil.adjusted.ID = anova(fit.1.water.fil.ID.1, fit.2.water.fil.ID.2, nBoot=1000, p.uni='adjusted')

# Analysis of Deviance Table
# 
# fit.2.water.fil.ID.2: zOTU.water.fil ~ time + offset(logTotalAbundance)
# fit.1.water.fil.ID.1: zOTU.water.fil ~ time + ID + offset(logTotalAbundance)
# 
# Multivariate test:
#                      Res.Df Df.diff   Dev Pr(>Dev)    
# fit.2.water.fil.ID.2     67                           
# fit.1.water.fil.ID.1     55      12 40632    0.001 ***

#save(globalTest1.dol.water.fil.adjusted.ID, file='globalTest1.dol.water.fil.adjusted.ID.Rdata')
```


######This tells us that there is an impact of ID, but it doesn’t tell us if the differences between dolphins stay constant (Effect of dolphin is larger than effect of time)########

############Check if the effect of ID is larger than that of time ###Variance component analysis

#############1. Deviance ‘explained’. The idea here is that you can look at # #1. 1) how well knowing the dolphin ID helps you explain the variance between samples taken #at the same time, versus #(2) how well knowing the time helps you explain the variance between samples taken from the #same dolphin. #If (1) is much larger than (2), then you could argue that samples from the same dolphin #across time are more similar than samples from different dolphins at the same time, which I #think is your argument? #I have attached some R code that uses one of the example datasets in the mvabund package to #do this; for your data you would obviously use dolphinID and time instead of soil and sand. #One tricky thing is that when considering two categorical variables (I wasn’t sure if time #is categorical in your model, but the point stands either way), the one with more levels is #expected to explain more of the deviance, even if the two variables are equally important. #This is the point of the “Divide by degrees of freedom” lines, which take this into account #— albeit in a very very rough way — which should give two measures of deviance explained #that are comparable to each other. The pseudo R2s have the same problem as the unscaled #deviances. Again, because these are rough measures, you would only be able to really draw a #conclusion if there is a big difference between them.


```
dat.dol_water.fil.t.9.mvabund <- mvabund(dat.dol_water.fil.t.9[,4:1474])

glm.dol.null <- manyglm(dat.dol_water.fil.t.9.mvabund ~ 1, family = "negative.binomial")

glm.dol.ID <- manyglm(dat.dol_water.fil.t.9.mvabund ~ ID, data = dat.dol_water.fil.t.9, family = "negative.binomial")

glm.dol.time <- manyglm(dat.dol_water.fil.t.9.mvabund ~ time, data = dat.dol_water.fil.t.9, family = "negative.binomial")

glm.dol.full <- manyglm(dat.dol_water.fil.t.9.mvabund ~ ID + time, data = dat.dol_water.fil.t.9, family = "negative.binomial")

# Total deviance to be explained
sum(glm.dol.null$deviance)
#46,182.21

# Deviance explained by ID, after conditioning on time
sum(glm.dol.time$deviance - glm.dol.full$deviance)
#4,470.378

# Divide by degrees of freedom (1 in this case, but will be K - 1 for categorical variable with K levels)
sum(glm.dol.time$deviance - glm.dol.full$deviance) / (nrow(glm.dol.full$coefficients) - nrow(glm.dol.time$coefficients))
#372.5315

# Deviance explained by time, after conditioning on ID
sum(glm.dol.ID$deviance - glm.dol.full$deviance)
#2591.475

# Divide by degrees of freedom (1 in this case, but will be K - 1 for categorical variable with K levels)
sum(glm.dol.ID$deviance - glm.dol.full$deviance) / (nrow(glm.dol.full$coefficients) - nrow(glm.dol.ID$coefficients))
#172.765

# Pseudo R^2 for ID
sum(glm.dol.time$deviance - glm.dol.full$deviance) / sum(glm.dol.time$deviance)
#0.09902719

# Pseudo R^2 for time
sum(glm.dol.ID$deviance - glm.dol.full$deviance) / sum(glm.dol.ID$deviance)
#0.05989905
```


#########How many Intra-core did the dolphin harbour? (Intra-core: zOTUs that persisted in each individual dolphin over four sampling occasions over five months)


```
##################### ###################### ###################### ###################### ######################
###Intra-core of full counts (qualitative core analysis) with only four samples included

############
###Determine 100% core of dolphins of UNrarefied data in week 6,11,19,28
#dat.dol_water.fil.t.2

dim(dat.dol_water.fil.t.2)
#83 1471

###Delete all dolphin samples apart from week 6,11,19,28 (Coen,Evie,Kiama,Moki,RB,
#Scooter,Sirius,Squeak,Starbuck,Howie,Nudgee)

dat.dol_water.fil.4samp.core = dat.dol_water.fil.t.2[-c(1,5,7,11,13:17,18,20,22,24,25,30,32,36,38,40, 42,44,46,47,51,53,58,62,64,69,73,75,77:83),]
```


###Coen


```
###Coen
#Calculate core taxa that 100 % of Coen's samples have in common
#dat.dol_water.fil.4samp.core

dat.dol_water.fil.4samp.core.Coen <-  dat.dol_water.fil.4samp.core[c(1:4),]

dim(dat.dol_water.fil.4samp.core.Coen)
#4 1571

dat.dol_water.fil.4samp.core.binary.Coen <- dat.dol_water.fil.4samp.core.Coen

#Convert dataframe into binary
dat.dol_water.fil.4samp.core.binary.Coen[] <- +(dat.dol_water.fil.4samp.core.binary.Coen  > 0)
#View(dat.dol_water.fil.4samp.core.binary.Coen [,1:10])

#Transpose dat.dol_water.fil.4samp.core.binary.Coen
dat.dol_water.fil.4samp.core.binary.Coen_t <- as.data.frame(t(dat.dol_water.fil.4samp.core.binary.Coen))
#View(dat.dol_water.fil.4samp.core.binary.Coen_t[1:10,])

#Create col Total.rel.abund.
dat.dol_water.fil.4samp.core.binary.Coen_t.2 <- dat.dol_water.fil.4samp.core.binary.Coen_t %>% 
  mutate(Total.rel.abund.zOTU = rowSums(dat.dol_water.fil.4samp.core.binary.Coen_t)/4)

#View(dat.dol_water.fil.4samp.core.binary.Coen_t.2[1:10,])

#Give dat.dol_water.fil.4samp.core.binary.Coen_t.2 rownames as col variable
dat.dol_water.fil.4samp.core.binary.Coen_t.2$variable <- rownames(dat.dol_water.fil.4samp.core.binary.Coen_t)
dim(dat.dol_water.fil.4samp.core.binary.Coen_t.2)
#1471 6

#Reorder
dat.dol_water.fil.4samp.core.binary.Coen_t.3 <- dat.dol_water.fil.4samp.core.binary.Coen_t.2[,c(5,6,1:4)]

#Join dat.dol_water.fil.4samp.core.binary.Coen_t.3 with attributes.migration_rdp
Core.zOTU.Coen.4samp <- dat.dol_water.fil.4samp.core.binary.Coen_t.3 %>% left_join(attributes.dol_rdp_water.fil.2)
dim(Core.zOTU.Coen.4samp)
# 1471   12

#View(Core.zOTU.Coen.4samp[1:10,])

#Reorder
Core.zOTU.Coen.4samp_2 = Core.zOTU.Coen.4samp[,c(1,2,11,3:6)]
dim(Core.zOTU.Coen.4samp_2)
##1471    7

#names(Core.zOTU.Coen.4samp_2)

#Order according to Core.zOTU.Coen.4samp_2$Total.rel.abund.zOTU
Core.zOTU.Coen.4samp_3 = Core.zOTU.Coen.4samp_2[order(Core.zOTU.Coen.4samp_2$Total.rel.abund.zOTU),]
#View(Core.zOTU.Coen.4samp_3[,1:2])

#Any coreOTUs above 100% (== 1.0)?
Core.zOTU.Coen.4samp_1.0 = Core.zOTU.Coen.4samp_3 %>% filter (Total.rel.abund.zOTU == 1.0)
dim(Core.zOTU.Coen.4samp_1.0)
#217 7

#######
###How many zOTUs are present per Coen's sample on average?
dat.dol_water.fil.4samp.core.binary.Coen_2 = dat.dol_water.fil.4samp.core.binary.Coen %>% mutate(sum_of_counts = rowSums(dat.dol_water.fil.4samp.core.binary.Coen))

mean(dat.dol_water.fil.4samp.core.binary.Coen_2$sum_of_counts)
#351

#######
#What's the prevalence of the core zOTUs in Coen over the four samples?

#View(dat.dol_water.fil.4samp.core.Coen[,1:10])

Core.Coen = Core.zOTU.Coen.4samp_1.0$variable

dat.dol_water.fil.4samp.core.Coen_2 = dat.dol_water.fil.4samp.core.Coen/rowSums(dat.dol_water.fil.4samp.core.Coen)
#View(dat.dol_water.fil.4samp.core.Coen_2)

dat.dol_water.fil.4samp.core.Coen_3 = dat.dol_water.fil.4samp.core.Coen_2[,Core.Coen]
#View(dat.dol_water.fil.4samp.core.Coen_3)

#rownames(dat.dol_water.fil.4samp.core.Coen_3)
#[1]  "Coen_28" "Coen_19" "Coen_11" "Coen_6"   

dat.dol_water.fil.4samp.core.Coen_4 = dat.dol_water.fil.4samp.core.Coen_3 %>% mutate(sum_of_rows = rowSums(dat.dol_water.fil.4samp.core.Coen_3))
#View(dat.dol_water.fil.4samp.core.Coen_4)
dat.dol_water.fil.4samp.core.Coen_4$sum_of_rows

#"Coen_6":  0.9441065
#"Coen_11": 0.9711325
#"Coen_19": 0.9298557
#"Coen_28": 0.6722240

mean(dat.dol_water.fil.4samp.core.Coen_4$sum_of_rows)
#0.8793297
```


###Evie


```
###Evie
#Calculate core taxa that 100 % of Evie's samples have in common
#dat.dol_water.fil.4samp.core

dat.dol_water.fil.4samp.core.Evie <-  dat.dol_water.fil.4samp.core[c(5:8),]

dim(dat.dol_water.fil.4samp.core.Evie)
#4 1571

dat.dol_water.fil.4samp.core.binary.Evie <- dat.dol_water.fil.4samp.core.Evie

#Convert dataframe into binary
dat.dol_water.fil.4samp.core.binary.Evie[] <- +(dat.dol_water.fil.4samp.core.binary.Evie  > 0)
#View(dat.dol_water.fil.4samp.core.binary.Evie [,1:10])

#Transpose dat.dol_water.fil.4samp.core.binary.Evie
dat.dol_water.fil.4samp.core.binary.Evie_t <- as.data.frame(t(dat.dol_water.fil.4samp.core.binary.Evie))
#View(dat.dol_water.fil.4samp.core.binary.Evie_t[1:10,])

#Create col Total.rel.abund.
dat.dol_water.fil.4samp.core.binary.Evie_t.2 <- dat.dol_water.fil.4samp.core.binary.Evie_t %>% 
  mutate(Total.rel.abund.zOTU = rowSums(dat.dol_water.fil.4samp.core.binary.Evie_t)/4)

#View(dat.dol_water.fil.4samp.core.binary.Evie_t.2[1:10,])

#Give dat.dol_water.fil.4samp.core.binary.Evie_t.2 rownames as col variable
dat.dol_water.fil.4samp.core.binary.Evie_t.2$variable <- rownames(dat.dol_water.fil.4samp.core.binary.Evie_t)
dim(dat.dol_water.fil.4samp.core.binary.Evie_t.2)
#1471 6

#Reorder
dat.dol_water.fil.4samp.core.binary.Evie_t.3 <- dat.dol_water.fil.4samp.core.binary.Evie_t.2[,c(5,6,1:4)]

#Join dat.dol_water.fil.4samp.core.binary.Evie_t.3 with attributes.migration_rdp
Core.zOTU.Evie.4samp <- dat.dol_water.fil.4samp.core.binary.Evie_t.3 %>% left_join(attributes.dol_rdp_water.fil.2)
dim(Core.zOTU.Evie.4samp)
# 1471   12

#View(Core.zOTU.Evie.4samp[1:10,])

#Reorder
Core.zOTU.Evie.4samp_2 = Core.zOTU.Evie.4samp[,c(1,2,11,3:6)]
dim(Core.zOTU.Evie.4samp_2)
##1471    7

#names(Core.zOTU.Evie.4samp_2)

#Order according to Core.zOTU.Evie.4samp_2$Total.rel.abund.zOTU
Core.zOTU.Evie.4samp_3 = Core.zOTU.Evie.4samp_2[order(Core.zOTU.Evie.4samp_2$Total.rel.abund.zOTU),]
#View(Core.zOTU.Evie.4samp_3[,1:2])

#Any coreOTUs above 100% (== 1.0)?
Core.zOTU.Evie.4samp_1.0 = Core.zOTU.Evie.4samp_3 %>% filter (Total.rel.abund.zOTU == 1.0)
dim(Core.zOTU.Evie.4samp_1.0)
#167 7

#######
###How many zOTUs are present per Evie's sample on average?
dat.dol_water.fil.4samp.core.binary.Evie_2 = dat.dol_water.fil.4samp.core.binary.Evie %>% mutate(sum_of_counts = rowSums(dat.dol_water.fil.4samp.core.binary.Evie))

mean(dat.dol_water.fil.4samp.core.binary.Evie_2$sum_of_counts)
#390

#######
#What's the prevalence of the core zOTUs in Evie over the four samples?

#View(dat.dol_water.fil.4samp.core.Evie[,1:10])

Core.Evie = Core.zOTU.Evie.4samp_1.0$variable

dat.dol_water.fil.4samp.core.Evie_2 = dat.dol_water.fil.4samp.core.Evie/rowSums(dat.dol_water.fil.4samp.core.Evie)
#View(dat.dol_water.fil.4samp.core.Evie_2)

dat.dol_water.fil.4samp.core.Evie_3 = dat.dol_water.fil.4samp.core.Evie_2[,Core.Evie]
#View(dat.dol_water.fil.4samp.core.Evie_3)

#rownames(dat.dol_water.fil.4samp.core.Evie_3)
#[1]  "Evie_28" "Evie_19" "Evie_11" "Evie_6"   

dat.dol_water.fil.4samp.core.Evie_4 = dat.dol_water.fil.4samp.core.Evie_3 %>% mutate(sum_of_rows = rowSums(dat.dol_water.fil.4samp.core.Evie_3))
#View(dat.dol_water.fil.4samp.core.Evie_4)
dat.dol_water.fil.4samp.core.Evie_4$sum_of_rows

#"Evie_6":  0.6769615 
#"Evie_11": 0.7337181 
#"Evie_19": 0.7346466 
#"Evie_28": 0.8045232 

mean(dat.dol_water.fil.4samp.core.Evie_4$sum_of_rows)
#0.7374624
```


###Kiama


```
###Kiama
#Calculate core taxa that 100 % of Kiama's samples have in common
#dat.dol_water.fil.4samp.core

dat.dol_water.fil.4samp.core.Kiama <-  dat.dol_water.fil.4samp.core[c(13:16),]

dim(dat.dol_water.fil.4samp.core.Kiama)
#4 1571

dat.dol_water.fil.4samp.core.binary.Kiama <- dat.dol_water.fil.4samp.core.Kiama

#Convert dataframe into binary
dat.dol_water.fil.4samp.core.binary.Kiama[] <- +(dat.dol_water.fil.4samp.core.binary.Kiama  > 0)
#View(dat.dol_water.fil.4samp.core.binary.Kiama [,1:10])

#Transpose dat.dol_water.fil.4samp.core.binary.Kiama
dat.dol_water.fil.4samp.core.binary.Kiama_t <- as.data.frame(t(dat.dol_water.fil.4samp.core.binary.Kiama))
#View(dat.dol_water.fil.4samp.core.binary.Kiama_t[1:10,])

#Create col Total.rel.abund.
dat.dol_water.fil.4samp.core.binary.Kiama_t.2 <- dat.dol_water.fil.4samp.core.binary.Kiama_t %>% 
  mutate(Total.rel.abund.zOTU = rowSums(dat.dol_water.fil.4samp.core.binary.Kiama_t)/4)

#View(dat.dol_water.fil.4samp.core.binary.Kiama_t.2[1:10,])

#Give dat.dol_water.fil.4samp.core.binary.Kiama_t.2 rownames as col variable
dat.dol_water.fil.4samp.core.binary.Kiama_t.2$variable <- rownames(dat.dol_water.fil.4samp.core.binary.Kiama_t)
dim(dat.dol_water.fil.4samp.core.binary.Kiama_t.2)
#1471 6

#Reorder
dat.dol_water.fil.4samp.core.binary.Kiama_t.3 <- dat.dol_water.fil.4samp.core.binary.Kiama_t.2[,c(5,6,1:4)]

#Join dat.dol_water.fil.4samp.core.binary.Kiama_t.3 with attributes.migration_rdp
Core.zOTU.Kiama.4samp <- dat.dol_water.fil.4samp.core.binary.Kiama_t.3 %>% left_join(attributes.dol_rdp_water.fil.2)
dim(Core.zOTU.Kiama.4samp)
# 1471   12

#View(Core.zOTU.Kiama.4samp[1:10,])

#Reorder
Core.zOTU.Kiama.4samp_2 = Core.zOTU.Kiama.4samp[,c(1,2,11,3:6)]
dim(Core.zOTU.Kiama.4samp_2)
##1471    7

#names(Core.zOTU.Kiama.4samp_2)

#Order according to Core.zOTU.Kiama.4samp_2$Total.rel.abund.zOTU
Core.zOTU.Kiama.4samp_3 = Core.zOTU.Kiama.4samp_2[order(Core.zOTU.Kiama.4samp_2$Total.rel.abund.zOTU),]
#View(Core.zOTU.Kiama.4samp_3[,1:2])

#Any coreOTUs above 100% (== 1.0)?
Core.zOTU.Kiama.4samp_1.0 = Core.zOTU.Kiama.4samp_3 %>% filter (Total.rel.abund.zOTU == 1.0)
dim(Core.zOTU.Kiama.4samp_1.0)
#191 7

#######
###How many zOTUs are present per Kiama's sample on average?
dat.dol_water.fil.4samp.core.binary.Kiama_2 = dat.dol_water.fil.4samp.core.binary.Kiama %>% mutate(sum_of_counts = rowSums(dat.dol_water.fil.4samp.core.binary.Kiama))

mean(dat.dol_water.fil.4samp.core.binary.Kiama_2$sum_of_counts)
#363

#######
#What's the prevalence of the core zOTUs in Kiama over the four samples?

#View(dat.dol_water.fil.4samp.core.Kiama[,1:10])

Core.Kiama = Core.zOTU.Kiama.4samp_1.0$variable

dat.dol_water.fil.4samp.core.Kiama_2 = dat.dol_water.fil.4samp.core.Kiama/rowSums(dat.dol_water.fil.4samp.core.Kiama)
#View(dat.dol_water.fil.4samp.core.Kiama_2)

dat.dol_water.fil.4samp.core.Kiama_3 = dat.dol_water.fil.4samp.core.Kiama_2[,Core.Kiama]
#View(dat.dol_water.fil.4samp.core.Kiama_3)

#rownames(dat.dol_water.fil.4samp.core.Kiama_3)
#[1]  "Kiama_28" "Kiama_19" "Kiama_11" "Kiama_6"   

dat.dol_water.fil.4samp.core.Kiama_4 = dat.dol_water.fil.4samp.core.Kiama_3 %>% mutate(sum_of_rows = rowSums(dat.dol_water.fil.4samp.core.Kiama_3))
#View(dat.dol_water.fil.4samp.core.Kiama_4)
dat.dol_water.fil.4samp.core.Kiama_4$sum_of_rows

#"Kiama_6":  0.9049215 
#"Kiama_11": 0.8656922 
#"Kiama_19": 0.8689472 
#"Kiama_28": 0.8074661

mean(dat.dol_water.fil.4samp.core.Kiama_4$sum_of_rows)
#0.8793297
```


###Moki


```
###Moki
#Calculate core taxa that 100 % of Moki's samples have in common
#dat.dol_water.fil.4samp.core

dat.dol_water.fil.4samp.core.Moki <-  dat.dol_water.fil.4samp.core[c(17:20),]

dim(dat.dol_water.fil.4samp.core.Moki)
#4 1571

dat.dol_water.fil.4samp.core.binary.Moki <- dat.dol_water.fil.4samp.core.Moki

#Convert dataframe into binary
dat.dol_water.fil.4samp.core.binary.Moki[] <- +(dat.dol_water.fil.4samp.core.binary.Moki  > 0)
#View(dat.dol_water.fil.4samp.core.binary.Moki [,1:10])

#Transpose dat.dol_water.fil.4samp.core.binary.Moki
dat.dol_water.fil.4samp.core.binary.Moki_t <- as.data.frame(t(dat.dol_water.fil.4samp.core.binary.Moki))
#View(dat.dol_water.fil.4samp.core.binary.Moki_t[1:10,])

#Create col Total.rel.abund.
dat.dol_water.fil.4samp.core.binary.Moki_t.2 <- dat.dol_water.fil.4samp.core.binary.Moki_t %>% 
  mutate(Total.rel.abund.zOTU = rowSums(dat.dol_water.fil.4samp.core.binary.Moki_t)/4)

#View(dat.dol_water.fil.4samp.core.binary.Moki_t.2[1:10,])

#Give dat.dol_water.fil.4samp.core.binary.Moki_t.2 rownames as col variable
dat.dol_water.fil.4samp.core.binary.Moki_t.2$variable <- rownames(dat.dol_water.fil.4samp.core.binary.Moki_t)
dim(dat.dol_water.fil.4samp.core.binary.Moki_t.2)
#1471 6

#Reorder
dat.dol_water.fil.4samp.core.binary.Moki_t.3 <- dat.dol_water.fil.4samp.core.binary.Moki_t.2[,c(5,6,1:4)]

#Join dat.dol_water.fil.4samp.core.binary.Moki_t.3 with attributes.migration_rdp
Core.zOTU.Moki.4samp <- dat.dol_water.fil.4samp.core.binary.Moki_t.3 %>% left_join(attributes.dol_rdp_water.fil.2)
dim(Core.zOTU.Moki.4samp)
# 1471   12

#View(Core.zOTU.Moki.4samp[1:10,])

#Reorder
Core.zOTU.Moki.4samp_2 = Core.zOTU.Moki.4samp[,c(1,2,11,3:6)]
dim(Core.zOTU.Moki.4samp_2)
##1471    7

#names(Core.zOTU.Moki.4samp_2)

#Order according to Core.zOTU.Moki.4samp_2$Total.rel.abund.zOTU
Core.zOTU.Moki.4samp_3 = Core.zOTU.Moki.4samp_2[order(Core.zOTU.Moki.4samp_2$Total.rel.abund.zOTU),]
#View(Core.zOTU.Moki.4samp_3[,1:2])

#Any coreOTUs above 100% (== 1.0)?
Core.zOTU.Moki.4samp_1.0 = Core.zOTU.Moki.4samp_3 %>% filter (Total.rel.abund.zOTU == 1.0)
dim(Core.zOTU.Moki.4samp_1.0)
#42 7

#######
###How many zOTUs are present per Moki's sample on average?
dat.dol_water.fil.4samp.core.binary.Moki_2 = dat.dol_water.fil.4samp.core.binary.Moki %>% mutate(sum_of_counts = rowSums(dat.dol_water.fil.4samp.core.binary.Moki))

mean(dat.dol_water.fil.4samp.core.binary.Moki_2$sum_of_counts)
#280

#######
#What's the prevalence of the core zOTUs in Moki over the four samples?

#View(dat.dol_water.fil.4samp.core.Moki[,1:10])

Core.Moki = Core.zOTU.Moki.4samp_1.0$variable

dat.dol_water.fil.4samp.core.Moki_2 = dat.dol_water.fil.4samp.core.Moki/rowSums(dat.dol_water.fil.4samp.core.Moki)
#View(dat.dol_water.fil.4samp.core.Moki_2)

dat.dol_water.fil.4samp.core.Moki_3 = dat.dol_water.fil.4samp.core.Moki_2[,Core.Moki]
#View(dat.dol_water.fil.4samp.core.Moki_3)

#rownames(dat.dol_water.fil.4samp.core.Moki_3)
#[1]  "Moki_28" "Moki_19" "Moki_11" "Moki_6"   

dat.dol_water.fil.4samp.core.Moki_4 = dat.dol_water.fil.4samp.core.Moki_3 %>% mutate(sum_of_rows = rowSums(dat.dol_water.fil.4samp.core.Moki_3))
#View(dat.dol_water.fil.4samp.core.Moki_4)
dat.dol_water.fil.4samp.core.Moki_4$sum_of_rows

#"Moki_6":  0.4411286 
#"Moki_11": 0.4935058
#"Moki_19": 0.5298532 
#"Moki_28": 0.4254895

mean(dat.dol_water.fil.4samp.core.Moki_4$sum_of_rows)
#0.4724942
```


###RB


```
###RB
#Calculate core taxa that 100 % of RB's samples have in common
#dat.dol_water.fil.4samp.core

dat.dol_water.fil.4samp.core.RB <-  dat.dol_water.fil.4samp.core[c(25:28),]

dim(dat.dol_water.fil.4samp.core.RB)
#4 1571

dat.dol_water.fil.4samp.core.binary.RB <- dat.dol_water.fil.4samp.core.RB

#Convert dataframe into binary
dat.dol_water.fil.4samp.core.binary.RB[] <- +(dat.dol_water.fil.4samp.core.binary.RB  > 0)
#View(dat.dol_water.fil.4samp.core.binary.RB [,1:10])

#Transpose dat.dol_water.fil.4samp.core.binary.RB
dat.dol_water.fil.4samp.core.binary.RB_t <- as.data.frame(t(dat.dol_water.fil.4samp.core.binary.RB))
#View(dat.dol_water.fil.4samp.core.binary.RB_t[1:10,])

#Create col Total.rel.abund.
dat.dol_water.fil.4samp.core.binary.RB_t.2 <- dat.dol_water.fil.4samp.core.binary.RB_t %>% 
  mutate(Total.rel.abund.zOTU = rowSums(dat.dol_water.fil.4samp.core.binary.RB_t)/4)

#View(dat.dol_water.fil.4samp.core.binary.RB_t.2[1:10,])

#Give dat.dol_water.fil.4samp.core.binary.RB_t.2 rownames as col variable
dat.dol_water.fil.4samp.core.binary.RB_t.2$variable <- rownames(dat.dol_water.fil.4samp.core.binary.RB_t)
dim(dat.dol_water.fil.4samp.core.binary.RB_t.2)
#1471 6

#Reorder
dat.dol_water.fil.4samp.core.binary.RB_t.3 <- dat.dol_water.fil.4samp.core.binary.RB_t.2[,c(5,6,1:4)]

#Join dat.dol_water.fil.4samp.core.binary.RB_t.3 with attributes.migration_rdp
Core.zOTU.RB.4samp <- dat.dol_water.fil.4samp.core.binary.RB_t.3 %>% left_join(attributes.dol_rdp_water.fil.2)
dim(Core.zOTU.RB.4samp)
# 1471   12

#View(Core.zOTU.RB.4samp[1:10,])

#Reorder
Core.zOTU.RB.4samp_2 = Core.zOTU.RB.4samp[,c(1,2,11,3:6)]
dim(Core.zOTU.RB.4samp_2)
##1471    7

#names(Core.zOTU.RB.4samp_2)

#Order according to Core.zOTU.RB.4samp_2$Total.rel.abund.zOTU
Core.zOTU.RB.4samp_3 = Core.zOTU.RB.4samp_2[order(Core.zOTU.RB.4samp_2$Total.rel.abund.zOTU),]
#View(Core.zOTU.RB.4samp_3[,1:2])

#Any coreOTUs above 100% (== 1.0)?
Core.zOTU.RB.4samp_1.0 = Core.zOTU.RB.4samp_3 %>% filter (Total.rel.abund.zOTU == 1.0)
dim(Core.zOTU.RB.4samp_1.0)
#180 7

#######
###How many zOTUs are present per RB's sample on average?
dat.dol_water.fil.4samp.core.binary.RB_2 = dat.dol_water.fil.4samp.core.binary.RB %>% mutate(sum_of_counts = rowSums(dat.dol_water.fil.4samp.core.binary.RB))

mean(dat.dol_water.fil.4samp.core.binary.RB_2$sum_of_counts)
#331

#######
#What's the prevalence of the core zOTUs in RB over the four samples?

#View(dat.dol_water.fil.4samp.core.RB[,1:10])

Core.RB = Core.zOTU.RB.4samp_1.0$variable

dat.dol_water.fil.4samp.core.RB_2 = dat.dol_water.fil.4samp.core.RB/rowSums(dat.dol_water.fil.4samp.core.RB)
#View(dat.dol_water.fil.4samp.core.RB_2)

dat.dol_water.fil.4samp.core.RB_3 = dat.dol_water.fil.4samp.core.RB_2[,Core.RB]
#View(dat.dol_water.fil.4samp.core.RB_3)

#rownames(dat.dol_water.fil.4samp.core.RB_3)
#[1]  "RB_28" "RB_19" "RB_11" "RB_6"   

dat.dol_water.fil.4samp.core.RB_4 = dat.dol_water.fil.4samp.core.RB_3 %>% mutate(sum_of_rows = rowSums(dat.dol_water.fil.4samp.core.RB_3))
#View(dat.dol_water.fil.4samp.core.RB_4)
dat.dol_water.fil.4samp.core.RB_4$sum_of_rows

#"RB_6":  0.9541434 
#"RB_11": 0.7682679
#"RB_19": 0.6376292 
#"RB_28": 0.8003591 

mean(dat.dol_water.fil.4samp.core.RB_4$sum_of_rows)
#0.7900999
```


###Scooter


```
###Scooter
#Calculate core taxa that 100 % of Scooter's samples have in common
#dat.dol_water.fil.4samp.core

dat.dol_water.fil.4samp.core.Scooter <-  dat.dol_water.fil.4samp.core[c(29:32),]

dim(dat.dol_water.fil.4samp.core.Scooter)
#4 1571

dat.dol_water.fil.4samp.core.binary.Scooter <- dat.dol_water.fil.4samp.core.Scooter

#Convert dataframe into binary
dat.dol_water.fil.4samp.core.binary.Scooter[] <- +(dat.dol_water.fil.4samp.core.binary.Scooter  > 0)
#View(dat.dol_water.fil.4samp.core.binary.Scooter [,1:10])

#Transpose dat.dol_water.fil.4samp.core.binary.Scooter
dat.dol_water.fil.4samp.core.binary.Scooter_t <- as.data.frame(t(dat.dol_water.fil.4samp.core.binary.Scooter))
#View(dat.dol_water.fil.4samp.core.binary.Scooter_t[1:10,])

#Create col Total.rel.abund.
dat.dol_water.fil.4samp.core.binary.Scooter_t.2 <- dat.dol_water.fil.4samp.core.binary.Scooter_t %>% 
  mutate(Total.rel.abund.zOTU = rowSums(dat.dol_water.fil.4samp.core.binary.Scooter_t)/4)

#View(dat.dol_water.fil.4samp.core.binary.Scooter_t.2[1:10,])

#Give dat.dol_water.fil.4samp.core.binary.Scooter_t.2 rownames as col variable
dat.dol_water.fil.4samp.core.binary.Scooter_t.2$variable <- rownames(dat.dol_water.fil.4samp.core.binary.Scooter_t)
dim(dat.dol_water.fil.4samp.core.binary.Scooter_t.2)
#1471 6

#Reorder
dat.dol_water.fil.4samp.core.binary.Scooter_t.3 <- dat.dol_water.fil.4samp.core.binary.Scooter_t.2[,c(5,6,1:4)]

#Join dat.dol_water.fil.4samp.core.binary.Scooter_t.3 with attributes.migration_rdp
Core.zOTU.Scooter.4samp <- dat.dol_water.fil.4samp.core.binary.Scooter_t.3 %>% left_join(attributes.dol_rdp_water.fil.2)
dim(Core.zOTU.Scooter.4samp)
# 1471   12

#View(Core.zOTU.Scooter.4samp[1:10,])

#Reorder
Core.zOTU.Scooter.4samp_2 = Core.zOTU.Scooter.4samp[,c(1,2,11,3:6)]
dim(Core.zOTU.Scooter.4samp_2)
##1471    7

#names(Core.zOTU.Scooter.4samp_2)

#Order according to Core.zOTU.Scooter.4samp_2$Total.rel.abund.zOTU
Core.zOTU.Scooter.4samp_3 = Core.zOTU.Scooter.4samp_2[order(Core.zOTU.Scooter.4samp_2$Total.rel.abund.zOTU),]
#View(Core.zOTU.Scooter.4samp_3[,1:2])

#Any coreOTUs above 100% (== 1.0)?
Core.zOTU.Scooter.4samp_1.0 = Core.zOTU.Scooter.4samp_3 %>% filter (Total.rel.abund.zOTU == 1.0)
dim(Core.zOTU.Scooter.4samp_1.0)
#51 7

#######
###How many zOTUs are present per Scooter's sample on average?
dat.dol_water.fil.4samp.core.binary.Scooter_2 = dat.dol_water.fil.4samp.core.binary.Scooter %>% mutate(sum_of_counts = rowSums(dat.dol_water.fil.4samp.core.binary.Scooter))

mean(dat.dol_water.fil.4samp.core.binary.Scooter_2$sum_of_counts)
#261

#######
#What's the prevalence of the core zOTUs in Scooter over the four samples?

#View(dat.dol_water.fil.4samp.core.Scooter[,1:10])

Core.Scooter = Core.zOTU.Scooter.4samp_1.0$variable

dat.dol_water.fil.4samp.core.Scooter_2 = dat.dol_water.fil.4samp.core.Scooter/rowSums(dat.dol_water.fil.4samp.core.Scooter)
#View(dat.dol_water.fil.4samp.core.Scooter_2)

dat.dol_water.fil.4samp.core.Scooter_3 = dat.dol_water.fil.4samp.core.Scooter_2[,Core.Scooter]
#View(dat.dol_water.fil.4samp.core.Scooter_3)

#rownames(dat.dol_water.fil.4samp.core.Scooter_3)
#[1]  "Scooter_28" "Scooter_19" "Scooter_11" "Scooter_6"   

dat.dol_water.fil.4samp.core.Scooter_4 = dat.dol_water.fil.4samp.core.Scooter_3 %>% mutate(sum_of_rows = rowSums(dat.dol_water.fil.4samp.core.Scooter_3))
#View(dat.dol_water.fil.4samp.core.Scooter_4)
dat.dol_water.fil.4samp.core.Scooter_4$sum_of_rows

#"Scooter_6":  0.4930344 
#"Scooter_11": 0.5192407  
#"Scooter_19": 0.4316927  
#"Scooter_28": 0.6540935

mean(dat.dol_water.fil.4samp.core.Scooter_4$sum_of_rows)
#0.5245153
```


###Sirius


```
###Sirius
#Calculate core taxa that 100 % of Sirius's samples have in common
#dat.dol_water.fil.4samp.core

dat.dol_water.fil.4samp.core.Sirius <-  dat.dol_water.fil.4samp.core[c(33:36),]

dim(dat.dol_water.fil.4samp.core.Sirius)
#4 1571

dat.dol_water.fil.4samp.core.binary.Sirius <- dat.dol_water.fil.4samp.core.Sirius

#Convert dataframe into binary
dat.dol_water.fil.4samp.core.binary.Sirius[] <- +(dat.dol_water.fil.4samp.core.binary.Sirius  > 0)
#View(dat.dol_water.fil.4samp.core.binary.Sirius [,1:10])

#Transpose dat.dol_water.fil.4samp.core.binary.Sirius
dat.dol_water.fil.4samp.core.binary.Sirius_t <- as.data.frame(t(dat.dol_water.fil.4samp.core.binary.Sirius))
#View(dat.dol_water.fil.4samp.core.binary.Sirius_t[1:10,])

#Create col Total.rel.abund.
dat.dol_water.fil.4samp.core.binary.Sirius_t.2 <- dat.dol_water.fil.4samp.core.binary.Sirius_t %>% 
  mutate(Total.rel.abund.zOTU = rowSums(dat.dol_water.fil.4samp.core.binary.Sirius_t)/4)

#View(dat.dol_water.fil.4samp.core.binary.Sirius_t.2[1:10,])

#Give dat.dol_water.fil.4samp.core.binary.Sirius_t.2 rownames as col variable
dat.dol_water.fil.4samp.core.binary.Sirius_t.2$variable <- rownames(dat.dol_water.fil.4samp.core.binary.Sirius_t)
dim(dat.dol_water.fil.4samp.core.binary.Sirius_t.2)
#1471 6

#Reorder
dat.dol_water.fil.4samp.core.binary.Sirius_t.3 <- dat.dol_water.fil.4samp.core.binary.Sirius_t.2[,c(5,6,1:4)]

#Join dat.dol_water.fil.4samp.core.binary.Sirius_t.3 with attributes.migration_rdp
Core.zOTU.Sirius.4samp <- dat.dol_water.fil.4samp.core.binary.Sirius_t.3 %>% left_join(attributes.dol_rdp_water.fil.2)
dim(Core.zOTU.Sirius.4samp)
# 1471   12

#View(Core.zOTU.Sirius.4samp[1:10,])

#Reorder
Core.zOTU.Sirius.4samp_2 = Core.zOTU.Sirius.4samp[,c(1,2,11,3:6)]
dim(Core.zOTU.Sirius.4samp_2)
##1471    7

#names(Core.zOTU.Sirius.4samp_2)

#Order according to Core.zOTU.Sirius.4samp_2$Total.rel.abund.zOTU
Core.zOTU.Sirius.4samp_3 = Core.zOTU.Sirius.4samp_2[order(Core.zOTU.Sirius.4samp_2$Total.rel.abund.zOTU),]
#View(Core.zOTU.Sirius.4samp_3[,1:2])

#Any coreOTUs above 100% (== 1.0)?
Core.zOTU.Sirius.4samp_1.0 = Core.zOTU.Sirius.4samp_3 %>% filter (Total.rel.abund.zOTU == 1.0)
dim(Core.zOTU.Sirius.4samp_1.0)
#38 7

#######
###How many zOTUs are present per Sirius's sample on average?
dat.dol_water.fil.4samp.core.binary.Sirius_2 = dat.dol_water.fil.4samp.core.binary.Sirius %>% mutate(sum_of_counts = rowSums(dat.dol_water.fil.4samp.core.binary.Sirius))

mean(dat.dol_water.fil.4samp.core.binary.Sirius_2$sum_of_counts)
#246

#######
#What's the prevalence of the core zOTUs in Sirius over the four samples?

#View(dat.dol_water.fil.4samp.core.Sirius[,1:10])

Core.Sirius = Core.zOTU.Sirius.4samp_1.0$variable

dat.dol_water.fil.4samp.core.Sirius_2 = dat.dol_water.fil.4samp.core.Sirius/rowSums(dat.dol_water.fil.4samp.core.Sirius)
#View(dat.dol_water.fil.4samp.core.Sirius_2)

dat.dol_water.fil.4samp.core.Sirius_3 = dat.dol_water.fil.4samp.core.Sirius_2[,Core.Sirius]
#View(dat.dol_water.fil.4samp.core.Sirius_3)

#rownames(dat.dol_water.fil.4samp.core.Sirius_3)
#[1]  "Sirius_28" "Sirius_19" "Sirius_11" "Sirius_6"   

dat.dol_water.fil.4samp.core.Sirius_4 = dat.dol_water.fil.4samp.core.Sirius_3 %>% mutate(sum_of_rows = rowSums(dat.dol_water.fil.4samp.core.Sirius_3))
#View(dat.dol_water.fil.4samp.core.Sirius_4)
dat.dol_water.fil.4samp.core.Sirius_4$sum_of_rows

#"Sirius_6":  0.4691971 
#"Sirius_11": 0.3868308 
#"Sirius_19": 0.4333038 
#"Sirius_28": 0.8032238 

mean(dat.dol_water.fil.4samp.core.Sirius_4$sum_of_rows)
#0.5231389
```


###Squeak


```
###Squeak
#Calculate core taxa that 100 % of Squeak's samples have in common
#dat.dol_water.fil.4samp.core

dat.dol_water.fil.4samp.core.Squeak <-  dat.dol_water.fil.4samp.core[c(37:40),]

dim(dat.dol_water.fil.4samp.core.Squeak)
#4 1571

dat.dol_water.fil.4samp.core.binary.Squeak <- dat.dol_water.fil.4samp.core.Squeak

#Convert dataframe into binary
dat.dol_water.fil.4samp.core.binary.Squeak[] <- +(dat.dol_water.fil.4samp.core.binary.Squeak  > 0)
#View(dat.dol_water.fil.4samp.core.binary.Squeak [,1:10])

#Transpose dat.dol_water.fil.4samp.core.binary.Squeak
dat.dol_water.fil.4samp.core.binary.Squeak_t <- as.data.frame(t(dat.dol_water.fil.4samp.core.binary.Squeak))
#View(dat.dol_water.fil.4samp.core.binary.Squeak_t[1:10,])

#Create col Total.rel.abund.
dat.dol_water.fil.4samp.core.binary.Squeak_t.2 <- dat.dol_water.fil.4samp.core.binary.Squeak_t %>% 
  mutate(Total.rel.abund.zOTU = rowSums(dat.dol_water.fil.4samp.core.binary.Squeak_t)/4)

#View(dat.dol_water.fil.4samp.core.binary.Squeak_t.2[1:10,])

#Give dat.dol_water.fil.4samp.core.binary.Squeak_t.2 rownames as col variable
dat.dol_water.fil.4samp.core.binary.Squeak_t.2$variable <- rownames(dat.dol_water.fil.4samp.core.binary.Squeak_t)
dim(dat.dol_water.fil.4samp.core.binary.Squeak_t.2)
#1471 6

#Reorder
dat.dol_water.fil.4samp.core.binary.Squeak_t.3 <- dat.dol_water.fil.4samp.core.binary.Squeak_t.2[,c(5,6,1:4)]

#Join dat.dol_water.fil.4samp.core.binary.Squeak_t.3 with attributes.migration_rdp
Core.zOTU.Squeak.4samp <- dat.dol_water.fil.4samp.core.binary.Squeak_t.3 %>% left_join(attributes.dol_rdp_water.fil.2)
dim(Core.zOTU.Squeak.4samp)
# 1471   12

#View(Core.zOTU.Squeak.4samp[1:10,])

#Reorder
Core.zOTU.Squeak.4samp_2 = Core.zOTU.Squeak.4samp[,c(1,2,11,3:6)]
dim(Core.zOTU.Squeak.4samp_2)
##1471    7

#names(Core.zOTU.Squeak.4samp_2)

#Order according to Core.zOTU.Squeak.4samp_2$Total.rel.abund.zOTU
Core.zOTU.Squeak.4samp_3 = Core.zOTU.Squeak.4samp_2[order(Core.zOTU.Squeak.4samp_2$Total.rel.abund.zOTU),]
#View(Core.zOTU.Squeak.4samp_3[,1:2])

#Any coreOTUs above 100% (== 1.0)?
Core.zOTU.Squeak.4samp_1.0 = Core.zOTU.Squeak.4samp_3 %>% filter (Total.rel.abund.zOTU == 1.0)
dim(Core.zOTU.Squeak.4samp_1.0)
#87 7

#######
###How many zOTUs are present per Squeak's sample on average?
dat.dol_water.fil.4samp.core.binary.Squeak_2 = dat.dol_water.fil.4samp.core.binary.Squeak %>% mutate(sum_of_counts = rowSums(dat.dol_water.fil.4samp.core.binary.Squeak))

mean(dat.dol_water.fil.4samp.core.binary.Squeak_2$sum_of_counts)
#334

#######
#What's the prevalence of the core zOTUs in Squeak over the four samples?

#View(dat.dol_water.fil.4samp.core.Squeak[,1:10])

Core.Squeak = Core.zOTU.Squeak.4samp_1.0$variable

dat.dol_water.fil.4samp.core.Squeak_2 = dat.dol_water.fil.4samp.core.Squeak/rowSums(dat.dol_water.fil.4samp.core.Squeak)
#View(dat.dol_water.fil.4samp.core.Squeak_2)

dat.dol_water.fil.4samp.core.Squeak_3 = dat.dol_water.fil.4samp.core.Squeak_2[,Core.Squeak]
#View(dat.dol_water.fil.4samp.core.Squeak_3)

#rownames(dat.dol_water.fil.4samp.core.Squeak_3)
#[1]  "Squeak_28" "Squeak_19" "Squeak_11" "Squeak_6"   

dat.dol_water.fil.4samp.core.Squeak_4 = dat.dol_water.fil.4samp.core.Squeak_3 %>% mutate(sum_of_rows = rowSums(dat.dol_water.fil.4samp.core.Squeak_3))
#View(dat.dol_water.fil.4samp.core.Squeak_4)
dat.dol_water.fil.4samp.core.Squeak_4$sum_of_rows

#"Squeak_6":  0.6652991 
#"Squeak_11": 0.9330231 
#"Squeak_19": 0.4595734 
#"Squeak_28": 0.4969427 

mean(dat.dol_water.fil.4samp.core.Squeak_4$sum_of_rows)
#0.6387096
```


###Starbuck


```
###Starbuck
#Calculate core taxa that 100 % of Starbuck's samples have in common
#dat.dol_water.fil.4samp.core

dat.dol_water.fil.4samp.core.Starbuck <-  dat.dol_water.fil.4samp.core[c(41:44),]

dim(dat.dol_water.fil.4samp.core.Starbuck)
#4 1571

dat.dol_water.fil.4samp.core.binary.Starbuck <- dat.dol_water.fil.4samp.core.Starbuck

#Convert dataframe into binary
dat.dol_water.fil.4samp.core.binary.Starbuck[] <- +(dat.dol_water.fil.4samp.core.binary.Starbuck  > 0)
#View(dat.dol_water.fil.4samp.core.binary.Starbuck [,1:10])

#Transpose dat.dol_water.fil.4samp.core.binary.Starbuck
dat.dol_water.fil.4samp.core.binary.Starbuck_t <- as.data.frame(t(dat.dol_water.fil.4samp.core.binary.Starbuck))
#View(dat.dol_water.fil.4samp.core.binary.Starbuck_t[1:10,])

#Create col Total.rel.abund.
dat.dol_water.fil.4samp.core.binary.Starbuck_t.2 <- dat.dol_water.fil.4samp.core.binary.Starbuck_t %>% 
  mutate(Total.rel.abund.zOTU = rowSums(dat.dol_water.fil.4samp.core.binary.Starbuck_t)/4)

#View(dat.dol_water.fil.4samp.core.binary.Starbuck_t.2[1:10,])

#Give dat.dol_water.fil.4samp.core.binary.Starbuck_t.2 rownames as col variable
dat.dol_water.fil.4samp.core.binary.Starbuck_t.2$variable <- rownames(dat.dol_water.fil.4samp.core.binary.Starbuck_t)
dim(dat.dol_water.fil.4samp.core.binary.Starbuck_t.2)
#1471 6

#Reorder
dat.dol_water.fil.4samp.core.binary.Starbuck_t.3 <- dat.dol_water.fil.4samp.core.binary.Starbuck_t.2[,c(5,6,1:4)]

#Join dat.dol_water.fil.4samp.core.binary.Starbuck_t.3 with attributes.migration_rdp
Core.zOTU.Starbuck.4samp <- dat.dol_water.fil.4samp.core.binary.Starbuck_t.3 %>% left_join(attributes.dol_rdp_water.fil.2)
dim(Core.zOTU.Starbuck.4samp)
# 1471   12

#View(Core.zOTU.Starbuck.4samp[1:10,])

#Reorder
Core.zOTU.Starbuck.4samp_2 = Core.zOTU.Starbuck.4samp[,c(1,2,11,3:6)]
dim(Core.zOTU.Starbuck.4samp_2)
##1471    7

#names(Core.zOTU.Starbuck.4samp_2)

#Order according to Core.zOTU.Starbuck.4samp_2$Total.rel.abund.zOTU
Core.zOTU.Starbuck.4samp_3 = Core.zOTU.Starbuck.4samp_2[order(Core.zOTU.Starbuck.4samp_2$Total.rel.abund.zOTU),]
#View(Core.zOTU.Starbuck.4samp_3[,1:2])

#Any coreOTUs above 100% (== 1.0)?
Core.zOTU.Starbuck.4samp_1.0 = Core.zOTU.Starbuck.4samp_3 %>% filter (Total.rel.abund.zOTU == 1.0)
dim(Core.zOTU.Starbuck.4samp_1.0)
#306 7

#######
###How many zOTUs are present per Starbuck's sample on average?
dat.dol_water.fil.4samp.core.binary.Starbuck_2 = dat.dol_water.fil.4samp.core.binary.Starbuck %>% mutate(sum_of_counts = rowSums(dat.dol_water.fil.4samp.core.binary.Starbuck))

mean(dat.dol_water.fil.4samp.core.binary.Starbuck_2$sum_of_counts)
#428

#######
#What's the prevalence of the core zOTUs in Starbuck over the four samples?

#View(dat.dol_water.fil.4samp.core.Starbuck[,1:10])

Core.Starbuck = Core.zOTU.Starbuck.4samp_1.0$variable

dat.dol_water.fil.4samp.core.Starbuck_2 = dat.dol_water.fil.4samp.core.Starbuck/rowSums(dat.dol_water.fil.4samp.core.Starbuck)
#View(dat.dol_water.fil.4samp.core.Starbuck_2)

dat.dol_water.fil.4samp.core.Starbuck_3 = dat.dol_water.fil.4samp.core.Starbuck_2[,Core.Starbuck]
#View(dat.dol_water.fil.4samp.core.Starbuck_3)

#rownames(dat.dol_water.fil.4samp.core.Starbuck_3)
#[1]  "Starbuck_28" "Starbuck_19" "Starbuck_11" "Starbuck_6"   

dat.dol_water.fil.4samp.core.Starbuck_4 = dat.dol_water.fil.4samp.core.Starbuck_3 %>% mutate(sum_of_rows = rowSums(dat.dol_water.fil.4samp.core.Starbuck_3))
#View(dat.dol_water.fil.4samp.core.Starbuck_4)
dat.dol_water.fil.4samp.core.Starbuck_4$sum_of_rows

#"Starbuck_6":  0.9679476 
#"Starbuck_11": 0.9538967   
#"Starbuck_19": 0.9658208   
#"Starbuck_28": 0.8919512   

mean(dat.dol_water.fil.4samp.core.Starbuck_4$sum_of_rows)
#0.944904
```


###Howie


```
###Howie
#Calculate core taxa that 100 % of Howie's samples have in common
#dat.dol_water.fil.4samp.core

dat.dol_water.fil.4samp.core.Howie <-  dat.dol_water.fil.4samp.core[c(9:12),]

dim(dat.dol_water.fil.4samp.core.Howie)
#4 1571

dat.dol_water.fil.4samp.core.binary.Howie <- dat.dol_water.fil.4samp.core.Howie

#Convert dataframe into binary
dat.dol_water.fil.4samp.core.binary.Howie[] <- +(dat.dol_water.fil.4samp.core.binary.Howie  > 0)
#View(dat.dol_water.fil.4samp.core.binary.Howie [,1:10])

#Transpose dat.dol_water.fil.4samp.core.binary.Howie
dat.dol_water.fil.4samp.core.binary.Howie_t <- as.data.frame(t(dat.dol_water.fil.4samp.core.binary.Howie))
#View(dat.dol_water.fil.4samp.core.binary.Howie_t[1:10,])

#Create col Total.rel.abund.
dat.dol_water.fil.4samp.core.binary.Howie_t.2 <- dat.dol_water.fil.4samp.core.binary.Howie_t %>% 
  mutate(Total.rel.abund.zOTU = rowSums(dat.dol_water.fil.4samp.core.binary.Howie_t)/4)

#View(dat.dol_water.fil.4samp.core.binary.Howie_t.2[1:10,])

#Give dat.dol_water.fil.4samp.core.binary.Howie_t.2 rownames as col variable
dat.dol_water.fil.4samp.core.binary.Howie_t.2$variable <- rownames(dat.dol_water.fil.4samp.core.binary.Howie_t)
dim(dat.dol_water.fil.4samp.core.binary.Howie_t.2)
#1471 6

#Reorder
dat.dol_water.fil.4samp.core.binary.Howie_t.3 <- dat.dol_water.fil.4samp.core.binary.Howie_t.2[,c(5,6,1:4)]

#Join dat.dol_water.fil.4samp.core.binary.Howie_t.3 with attributes.migration_rdp
Core.zOTU.Howie.4samp <- dat.dol_water.fil.4samp.core.binary.Howie_t.3 %>% left_join(attributes.dol_rdp_water.fil.2)
dim(Core.zOTU.Howie.4samp)
# 1471   12

#View(Core.zOTU.Howie.4samp[1:10,])

#Reorder
Core.zOTU.Howie.4samp_2 = Core.zOTU.Howie.4samp[,c(1,2,11,3:6)]
dim(Core.zOTU.Howie.4samp_2)
##1471    7

#names(Core.zOTU.Howie.4samp_2)

#Order according to Core.zOTU.Howie.4samp_2$Total.rel.abund.zOTU
Core.zOTU.Howie.4samp_3 = Core.zOTU.Howie.4samp_2[order(Core.zOTU.Howie.4samp_2$Total.rel.abund.zOTU),]
#View(Core.zOTU.Howie.4samp_3[,1:2])

#Any coreOTUs above 100% (== 1.0)?
Core.zOTU.Howie.4samp_1.0 = Core.zOTU.Howie.4samp_3 %>% filter (Total.rel.abund.zOTU == 1.0)
dim(Core.zOTU.Howie.4samp_1.0)
#309  7

#######
###How many zOTUs are present per Howie's sample on average?
dat.dol_water.fil.4samp.core.binary.Howie_2 = dat.dol_water.fil.4samp.core.binary.Howie %>% mutate(sum_of_counts = rowSums(dat.dol_water.fil.4samp.core.binary.Howie))

mean(dat.dol_water.fil.4samp.core.binary.Howie_2$sum_of_counts)
#465

#######
#What's the prevalence of the core zOTUs in Howie over the four samples?

#View(dat.dol_water.fil.4samp.core.Howie[,1:10])

Core.Howie = Core.zOTU.Howie.4samp_1.0$variable

dat.dol_water.fil.4samp.core.Howie_2 = dat.dol_water.fil.4samp.core.Howie/rowSums(dat.dol_water.fil.4samp.core.Howie)
#View(dat.dol_water.fil.4samp.core.Howie_2)

dat.dol_water.fil.4samp.core.Howie_3 = dat.dol_water.fil.4samp.core.Howie_2[,Core.Howie]
#View(dat.dol_water.fil.4samp.core.Howie_3)

#rownames(dat.dol_water.fil.4samp.core.Howie_3)
#[1]  "Howie_28" "Howie_19" "Howie_11" "Howie_6"   

dat.dol_water.fil.4samp.core.Howie_4 = dat.dol_water.fil.4samp.core.Howie_3 %>% mutate(sum_of_rows = rowSums(dat.dol_water.fil.4samp.core.Howie_3))
#View(dat.dol_water.fil.4samp.core.Howie_4)
dat.dol_water.fil.4samp.core.Howie_4$sum_of_rows

#"Howie_6":  0.8777274 
#"Howie_12": 0.9546271 
#"Howie_19": 0.7486298 
#"Howie_28": 0.8954603 

mean(dat.dol_water.fil.4samp.core.Howie_4$sum_of_rows)
#0.8691112
```


###Nudgee


```
###Nudgee
#Calculate core taxa that 100 % of Nudgee's samples have in common
#dat.dol_water.fil.4samp.core

dat.dol_water.fil.4samp.core.Nudgee <-  dat.dol_water.fil.4samp.core[c(21:24),]

dim(dat.dol_water.fil.4samp.core.Nudgee)
#4 1571

dat.dol_water.fil.4samp.core.binary.Nudgee <- dat.dol_water.fil.4samp.core.Nudgee

#Convert dataframe into binary
dat.dol_water.fil.4samp.core.binary.Nudgee[] <- +(dat.dol_water.fil.4samp.core.binary.Nudgee  > 0)
#View(dat.dol_water.fil.4samp.core.binary.Nudgee [,1:10])

#Transpose dat.dol_water.fil.4samp.core.binary.Nudgee
dat.dol_water.fil.4samp.core.binary.Nudgee_t <- as.data.frame(t(dat.dol_water.fil.4samp.core.binary.Nudgee))
#View(dat.dol_water.fil.4samp.core.binary.Nudgee_t[1:10,])

#Create col Total.rel.abund.
dat.dol_water.fil.4samp.core.binary.Nudgee_t.2 <- dat.dol_water.fil.4samp.core.binary.Nudgee_t %>% 
  mutate(Total.rel.abund.zOTU = rowSums(dat.dol_water.fil.4samp.core.binary.Nudgee_t)/4)

#View(dat.dol_water.fil.4samp.core.binary.Nudgee_t.2[1:10,])

#Give dat.dol_water.fil.4samp.core.binary.Nudgee_t.2 rownames as col variable
dat.dol_water.fil.4samp.core.binary.Nudgee_t.2$variable <- rownames(dat.dol_water.fil.4samp.core.binary.Nudgee_t)
dim(dat.dol_water.fil.4samp.core.binary.Nudgee_t.2)
#1471 6

#Reorder
dat.dol_water.fil.4samp.core.binary.Nudgee_t.3 <- dat.dol_water.fil.4samp.core.binary.Nudgee_t.2[,c(5,6,1:4)]

#Join dat.dol_water.fil.4samp.core.binary.Nudgee_t.3 with attributes.migration_rdp
Core.zOTU.Nudgee.4samp <- dat.dol_water.fil.4samp.core.binary.Nudgee_t.3 %>% left_join(attributes.dol_rdp_water.fil.2)
dim(Core.zOTU.Nudgee.4samp)
# 1471   12

#View(Core.zOTU.Nudgee.4samp[1:10,])

#Reorder
Core.zOTU.Nudgee.4samp_2 = Core.zOTU.Nudgee.4samp[,c(1,2,11,3:6)]
dim(Core.zOTU.Nudgee.4samp_2)
##1471    7

#names(Core.zOTU.Nudgee.4samp_2)

#Order according to Core.zOTU.Nudgee.4samp_2$Total.rel.abund.zOTU
Core.zOTU.Nudgee.4samp_3 = Core.zOTU.Nudgee.4samp_2[order(Core.zOTU.Nudgee.4samp_2$Total.rel.abund.zOTU),]
#View(Core.zOTU.Nudgee.4samp_3[,1:2])

#Any coreOTUs above 100% (== 1.0)?
Core.zOTU.Nudgee.4samp_1.0 = Core.zOTU.Nudgee.4samp_3 %>% filter (Total.rel.abund.zOTU == 1.0)
dim(Core.zOTU.Nudgee.4samp_1.0)
#115   7

#######
###How many zOTUs are present per Nudgee's sample on average?
dat.dol_water.fil.4samp.core.binary.Nudgee_2 = dat.dol_water.fil.4samp.core.binary.Nudgee %>% mutate(sum_of_counts = rowSums(dat.dol_water.fil.4samp.core.binary.Nudgee))

mean(dat.dol_water.fil.4samp.core.binary.Nudgee_2$sum_of_counts)
#245

#######
#What's the prevalence of the core zOTUs in Nudgee over the four samples?

#View(dat.dol_water.fil.4samp.core.Nudgee[,1:10])

Core.Nudgee = Core.zOTU.Nudgee.4samp_1.0$variable

dat.dol_water.fil.4samp.core.Nudgee_2 = dat.dol_water.fil.4samp.core.Nudgee/rowSums(dat.dol_water.fil.4samp.core.Nudgee)
#View(dat.dol_water.fil.4samp.core.Nudgee_2)

dat.dol_water.fil.4samp.core.Nudgee_3 = dat.dol_water.fil.4samp.core.Nudgee_2[,Core.Nudgee]
#View(dat.dol_water.fil.4samp.core.Nudgee_3)

#rownames(dat.dol_water.fil.4samp.core.Nudgee_3)

dat.dol_water.fil.4samp.core.Nudgee_4 = dat.dol_water.fil.4samp.core.Nudgee_3 %>% mutate(sum_of_rows = rowSums(dat.dol_water.fil.4samp.core.Nudgee_3))
#View(dat.dol_water.fil.4samp.core.Nudgee_4)
dat.dol_water.fil.4samp.core.Nudgee_4$sum_of_rows

#"Nudgee_6":  0.8374775 
#"Nudgee_12": 0.5964184 
#"Nudgee_20": 0.9198765 
#"Nudgee_28": 0.7960500 

mean(dat.dol_water.fil.4samp.core.Nudgee_4$sum_of_rows)
#0.7874556
```


#####Do dolphins have similar intra-core zOTUs? Let’s check!


```
#4samples (UNrarefied)
#100%
Core.zOTU.4samp_1.0 = c(Core.zOTU.Coen.4samp_1.0$variable,Core.zOTU.Evie.4samp_1.0$variable, Core.zOTU.Kiama.4samp_1.0$variable,Core.zOTU.Moki.4samp_1.0$variable,
                       Core.zOTU.RB.4samp_1.0$variable,Core.zOTU.Scooter.4samp_1.0$variable,
                       Core.zOTU.Sirius.4samp_1.0$variable,Core.zOTU.Squeak.4samp_1.0$variable,
                       Core.zOTU.Starbuck.4samp_1.0$variable,Core.zOTU.Howie.4samp_1.0$variable,
                       Core.zOTU.Nudgee.4samp_1.0$variable)

length(Core.zOTU.4samp_1.0)
#1703
length(unique(Core.zOTU.4samp_1.0))
#503

Core.zOTU.4samp_1.0.unique = unique(Core.zOTU.4samp_1.0)
write.csv(Core.zOTU.4samp_1.0.unique,file='Core.zOTU.4samp_1.0.unique_NEW.csv')
```

###Determine rel. abund. of intra-core zOTUs


```
sum(dat.dol_water.fil.4samp.core.rel.abund_t.core.2$mean.rel.abund)
```


```
[1] 0.955076
```


###Create line plot with rel.abund. of core

###Create frequency histogram of intra-core


```
Core.zOTU.Coen.4samp_1.0_2 <- Core.zOTU.Coen.4samp_1.0[,-c(1,3,5:7)]
Core.zOTU.Evie.4samp_1.0_2 <- Core.zOTU.Evie.4samp_1.0[,-c(1,3,5:7)]
Core.zOTU.Moki.4samp_1.0_2 <- Core.zOTU.Moki.4samp_1.0[,-c(1,3,5:7)]
Core.zOTU.Kiama.4samp_1.0_2 <- Core.zOTU.Kiama.4samp_1.0[,-c(1,3,5:7)]
Core.zOTU.RB.4samp_1.0_2 <- Core.zOTU.RB.4samp_1.0[,-c(1,3,5:7)]
Core.zOTU.Scooter.4samp_1.0_2 <- Core.zOTU.Scooter.4samp_1.0[,-c(1,3,5:7)]
Core.zOTU.Sirius.4samp_1.0_2 <- Core.zOTU.Sirius.4samp_1.0[,-c(1,3,5:7)]
Core.zOTU.Squeak.4samp_1.0_2 <- Core.zOTU.Squeak.4samp_1.0[,-c(1,3,5:7)]
Core.zOTU.Starbuck.4samp_1.0_2 <- Core.zOTU.Starbuck.4samp_1.0[,-c(1,3,5:7)]
Core.zOTU.Howie.4samp_1.0_2 <- Core.zOTU.Howie.4samp_1.0[,-c(1,3,5:7)]
Core.zOTU.Nudgee.4samp_1.0_2 <- Core.zOTU.Nudgee.4samp_1.0[,-c(1,3,5:7)]


Core.zOTU.all.dol <- full_join (by = c('variable'), Core.zOTU.Coen.4samp_1.0_2, Core.zOTU.Evie.4samp_1.0_2)
Core.zOTU.all.dol.2 <- full_join (by = c('variable'), Core.zOTU.all.dol, Core.zOTU.Moki.4samp_1.0_2)
Core.zOTU.all.dol.3 <- full_join (by = c('variable'), Core.zOTU.all.dol.2, Core.zOTU.Kiama.4samp_1.0_2)
Core.zOTU.all.dol.4 <- full_join (by = c('variable'), Core.zOTU.all.dol.3, Core.zOTU.Scooter.4samp_1.0_2)
Core.zOTU.all.dol.5 <- full_join (by = c('variable'), Core.zOTU.all.dol.4, Core.zOTU.RB.4samp_1.0_2)
Core.zOTU.all.dol.6 <- full_join (by = c('variable'), Core.zOTU.all.dol.5, Core.zOTU.Sirius.4samp_1.0_2)
Core.zOTU.all.dol.7 <- full_join (by = c('variable'), Core.zOTU.all.dol.6, Core.zOTU.Squeak.4samp_1.0_2)
Core.zOTU.all.dol.8 <- full_join (by = c('variable'), Core.zOTU.all.dol.7, Core.zOTU.Starbuck.4samp_1.0_2)
Core.zOTU.all.dol.9 <- full_join (by = c('variable'), Core.zOTU.all.dol.8, Core.zOTU.Howie.4samp_1.0_2)
Core.zOTU.all.dol.10 <- full_join (by = c('variable'), Core.zOTU.all.dol.9, Core.zOTU.Nudgee.4samp_1.0_2)

dim(Core.zOTU.all.dol.10)

Core.zOTU.all.dol.11 <- Core.zOTU.all.dol.10 %>% mutate (sum_of_rows=rowSums(Core.zOTU.all.dol.10[,2:12],na.rm=TRUE)) %>% arrange(sum_of_rows)
#View(Core.zOTU.all.dol.11)

Intra.core.histo <- as.data.frame(Core.zOTU.all.dol.11$sum_of_rows)
names(Intra.core.histo) <- 'Intra.core.histo'

###Draw histogram with Core.zOTU.all.dol.11$sum_of_rows
Intra.core.histogram =
  ggplot(Intra.core.histo, aes(x = Intra.core.histo)) +
  geom_histogram(binwidth = 0.5)+
  theme_bw() +
  theme(plot.title = element_text(size = 14, family = "Tahoma", face = "bold"),
        text = element_text(size = 12, family = "Tahoma"),
        axis.title = element_text(),
        axis.text.x=element_text(size = 11)) +
  scale_x_continuous(name = "Number of dolphins that harbour core zOTUs", 
                     breaks = seq(1:11)) +
  scale_y_continuous(name = "Number of intra-core zOTUs")

ggsave("Intra.core.histogram.jpg", plot = Intra.core.histogram , device = 'jpg', width = 168, height = 130, units = "mm",
       dpi = 300, limitsize = TRUE)
```

#######Determine inter-core (bacteria all dolphins share at any point in time)


```
###Use dat.dol_water.fil.t.2
dim(dat.dol_water.fil.t.2)
#83 1471
dat.dol_water.fil.t.15 <- dat.dol_water.fil.t.2
dat.dol_water.fil.t.15$Dolphin <- rownames(dat.dol_water.fil.t.15)
dat.dol_water.fil.t.16 <- dat.dol_water.fil.t.15
dat.dol_water.fil.t.16$Dolphin.2 <- dat.dol_water.fil.t.16$Dolphin

dim(dat.dol_water.fil.t.16)
#83 1473

###Re-order, bring cols Dolphin & Dolphin.2 to front
dat.dol_water.fil.t.17 = dat.dol_water.fil.t.16[,c(1472,1473,1:1471)]

dat.dol_water.fil.t.18 = separate(dat.dol_water.fil.t.17, Dolphin.2, c('ID','time'))

dat.dol_water.fil.t.19 = dat.dol_water.fil.t.18[order(dat.dol_water.fil.t.18$time),]
```

###Separate inter-core by week #####################

###Week2


```
#####################
##2 (10 dolphins)
dat.dol_water.fil.week.2 <- 
  dat.dol_water.fil.t.19[c(31:40),]

#View(dat.dol_water.fil.week.2[,1:10])
dim(dat.dol_water.fil.week.2)
#10 1474

write.csv(dat.dol_water.fil.week.2, 'dat.dol_water.fil.week.2.csv')

###Relative abundance
dat.dol_water.fil.week.2.2 <- dat.dol_water.fil.week.2[,-c(1,3)]
dim(dat.dol_water.fil.week.2.2)
#10 1472

dat.dol_water.fil.week.2_rel.abund <- dat.dol_water.fil.week.2.2[,2:1472] /rowSums(dat.dol_water.fil.week.2.2[,2:1472])

#Transpose
dat.dol_water.fil.week.2_rel.abund_t <- as.data.frame(t(dat.dol_water.fil.week.2_rel.abund))

dat.dol_water.fil.week.2_rel.abund_t$variable <- rownames(dat.dol_water.fil.week.2_rel.abund_t)
dim(dat.dol_water.fil.week.2_rel.abund_t)
#1471   11

dat.dol_water.fil.week.2_rel.abund_t.2 <- dat.dol_water.fil.week.2_rel.abund_t %>% mutate(mean_of_rows = rowMeans(dat.dol_water.fil.week.2_rel.abund_t[,1:10]))

Rel.abund_week.2 <- dat.dol_water.fil.week.2_rel.abund_t.2[,11:12]

#Convert dataframe into binary
dat.dol_water.fil.week.2.binary <- dat.dol_water.fil.week.2[,4:1471]

dat.dol_water.fil.week.2.binary []  <-  +(dat.dol_water.fil.week.2.binary  > 0)

#View(dat.dol_water.fil.week.2.binary [,1:10])

#Transpose dat.dol_water.fil.week.2.binary
dat.dol_water.fil.week.2.binary_t <- as.data.frame(t(dat.dol_water.fil.week.2.binary))
#View(dat.dol_water.fil.week.2.binary[1:10,])

#Create col Total.rel.abund.
dat.dol_water.fil.week.2.binary_t.2 <- dat.dol_water.fil.week.2.binary_t %>% 
  mutate (Total.rel.abund.zOTU = rowSums(dat.dol_water.fil.week.2.binary_t)/10)

#View(dat.dol_water.fil.week.2.binary_t.2[1:10,])

#Give dat.dol_water.fil.week.2.binary_t.2 rownames as col variable
dat.dol_water.fil.week.2.binary_t.2$variable <- rownames(dat.dol_water.fil.week.2.binary_t)
dim(dat.dol_water.fil.week.2.binary_t.2)
#1468 12

#Reorder
dat.dol_water.fil.week.2.binary_t.3 <- dat.dol_water.fil.week.2.binary_t.2[,c(12,11,1:10)]

#Join dat.dol_water.fil.week.2.binary_t.3 with attributes.dol_rdp_water.fil.2
Inter.core.zOTU.week.2 <- dat.dol_water.fil.week.2.binary_t.3 %>% left_join(attributes.dol_rdp_water.fil.2)
dim(Inter.core.zOTU.week.2)
#1468   18
#View(Inter.core.zOTU.week.2[1:10,])

#Reorder
Inter.core.zOTU.week.2_2 <- Inter.core.zOTU.week.2[,c(1,2,17,3:12)]
dim(Inter.core.zOTU.week.2_2)
##1885 13

names(Inter.core.zOTU.week.2_2)

#Order according to Inter.core.zOTU.week.2_2$Total.rel.abund.zOTU
Inter.core.zOTU.week.2_3 <- Inter.core.zOTU.week.2_2[order(Inter.core.zOTU.week.2_2$Total.rel.abund.zOTU),]
#View(Inter.core.zOTU.week.2_3[,1:2])

#Any inter.core.OTUs above 100% (== 1.0)?
Inter.core.zOTU.week.2_1.0 <- Inter.core.zOTU.week.2_3 %>% filter (Total.rel.abund.zOTU == 1.0)
dim(Inter.core.zOTU.week.2_1.0)
#32

#Determine relative abundance of those core zOTUs
Inter.core_week.2 <- Inter.core.zOTU.week.2_1.0$variable

Rel.abund_week.2.2 <- Rel.abund_week.2

rownames(Rel.abund_week.2.2) <- Rel.abund_week.2.2$variable

Rel.abund_week.2.Core <- Rel.abund_week.2.2[Inter.core_week.2,]

sum(Rel.abund_week.2.Core[,2])
#0.2729034

write.csv(Rel.abund_week.2.Core, 'Rel.abund_week.2.Core.csv')
```


##Week 6 (10 dolphins)


```
sum(Rel.abund_week.6.Core[,2])
```


```
[1] 0.1856986
```

##Week 11 (10 dolphins)


```
sum(Rel.abund_week.11.Core[,2])
```


```
[1] 0.2652669
```

###Week 19 (10 dolphins)


```
#####################
##19 (10 dolphins)
dat.dol_water.fil.week.19 <- 
  dat.dol_water.fil.t.19[c(20:29),]

#View(dat.dol_water.fil.week.19[,1:10])
dim(dat.dol_water.fil.week.19)
#10 1474

write.csv(dat.dol_water.fil.week.19, 'dat.dol_water.fil.week.19.csv')

###Relative abundance
dat.dol_water.fil.week.19.2 <- dat.dol_water.fil.week.19[,-c(1,3)]
dim(dat.dol_water.fil.week.19.2)
#10 1472

dat.dol_water.fil.week.19_rel.abund <- dat.dol_water.fil.week.19.2[,2:1472] /rowSums(dat.dol_water.fil.week.19.2[,2:1472])

#Transpose
dat.dol_water.fil.week.19_rel.abund_t <- as.data.frame(t(dat.dol_water.fil.week.19_rel.abund))

dat.dol_water.fil.week.19_rel.abund_t$variable <- rownames(dat.dol_water.fil.week.19_rel.abund_t)
dim(dat.dol_water.fil.week.19_rel.abund_t)
#1471   11

dat.dol_water.fil.week.19_rel.abund_t.2 <- dat.dol_water.fil.week.19_rel.abund_t %>% mutate(mean_of_rows = rowMeans(dat.dol_water.fil.week.19_rel.abund_t[,1:10]))

Rel.abund_week.19 <- dat.dol_water.fil.week.19_rel.abund_t.2[,11:12]

#Convert dataframe into binary
dat.dol_water.fil.week.19.binary <- dat.dol_water.fil.week.19[,4:1471]

dat.dol_water.fil.week.19.binary []  <-  +(dat.dol_water.fil.week.19.binary  > 0)

#View(dat.dol_water.fil.week.19.binary [,1:10])

#Transpose dat.dol_water.fil.week.19.binary
dat.dol_water.fil.week.19.binary_t <- as.data.frame(t(dat.dol_water.fil.week.19.binary))
#View(dat.dol_water.fil.week.19.binary[1:10,])

#Create col Total.rel.abund.
dat.dol_water.fil.week.19.binary_t.2 <- dat.dol_water.fil.week.19.binary_t %>% 
  mutate (Total.rel.abund.zOTU = rowSums(dat.dol_water.fil.week.19.binary_t)/10)

#View(dat.dol_water.fil.week.19.binary_t.2[1:10,])

#Give dat.dol_water.fil.week.19.binary_t.2 rownames as col variable
dat.dol_water.fil.week.19.binary_t.2$variable <- rownames(dat.dol_water.fil.week.19.binary_t)
dim(dat.dol_water.fil.week.19.binary_t.2)
#1468 12

#Reorder
dat.dol_water.fil.week.19.binary_t.3 <- dat.dol_water.fil.week.19.binary_t.2[,c(12,11,1:10)]

#Join dat.dol_water.fil.week.19.binary_t.3 with attributes.dol_rdp_water.fil.2
Inter.core.zOTU.week.19 <- dat.dol_water.fil.week.19.binary_t.3 %>% left_join(attributes.dol_rdp_water.fil.2)
dim(Inter.core.zOTU.week.19)
#1468   18
#View(Inter.core.zOTU.week.19[1:10,])

#Reorder
Inter.core.zOTU.week.19_2 <- Inter.core.zOTU.week.19[,c(1,2,17,3:12)]
dim(Inter.core.zOTU.week.19_2)
##1468 13

names(Inter.core.zOTU.week.19_2)

#Order according to Inter.core.zOTU.week.19_2$Total.rel.abund.zOTU
Inter.core.zOTU.week.19_3 <- Inter.core.zOTU.week.19_2[order(Inter.core.zOTU.week.19_2$Total.rel.abund.zOTU),]
#View(Inter.core.zOTU.week.19_3[,1:2])

#Any inter.core.OTUs above 100% (== 1.0)?
Inter.core.zOTU.week.19_1.0 <- Inter.core.zOTU.week.19_3 %>% filter (Total.rel.abund.zOTU == 1.0)
dim(Inter.core.zOTU.week.19_1.0)
#66

#Determine relative abundance of those core zOTUs
Inter.core_week.19 <- Inter.core.zOTU.week.19_1.0$variable

Rel.abund_week.19.2 <- Rel.abund_week.19

rownames(Rel.abund_week.19.2) <- Rel.abund_week.19.2$variable

Rel.abund_week.19.2 <- Rel.abund_week.19.2[Inter.core_week.19,]

sum(Inter.core_week.19[,2])
#0.1856986

#Determine relative abundance of those core zOTUs
Inter.core_week.19 <- Inter.core.zOTU.week.19_1.0$variable

Rel.abund_week.19.2 <- Rel.abund_week.19

rownames(Rel.abund_week.19.2) <- Rel.abund_week.19.2$variable

Rel.abund_week.19.Core <- Rel.abund_week.19.2[Inter.core_week.19,]

sum(Rel.abund_week.19.Core[,2])
#0.4132573
```

##Week 28 (10 dolphins)


```
#####################
##28 (10 dolphins)
dat.dol_water.fil.week.28 <- 
  dat.dol_water.fil.t.19[c(48:57),]

#View(dat.dol_water.fil.week.28[,1:10])
dim(dat.dol_water.fil.week.28)
#10 1474

write.csv(dat.dol_water.fil.week.28, 'dat.dol_water.fil.week.28.csv')

###Relative abundance
dat.dol_water.fil.week.28.2 <- dat.dol_water.fil.week.28[,-c(1,3)]
dim(dat.dol_water.fil.week.28.2)
#10 1472

dat.dol_water.fil.week.28_rel.abund <- dat.dol_water.fil.week.28.2[,2:1472] /rowSums(dat.dol_water.fil.week.28.2[,2:1472])

#Transpose
dat.dol_water.fil.week.28_rel.abund_t <- as.data.frame(t(dat.dol_water.fil.week.28_rel.abund))

dat.dol_water.fil.week.28_rel.abund_t$variable <- rownames(dat.dol_water.fil.week.28_rel.abund_t)
dim(dat.dol_water.fil.week.28_rel.abund_t)
#1471   11

dat.dol_water.fil.week.28_rel.abund_t.2 <- dat.dol_water.fil.week.28_rel.abund_t %>% mutate(mean_of_rows = rowMeans(dat.dol_water.fil.week.28_rel.abund_t[,1:10]))

Rel.abund_week.28 <- dat.dol_water.fil.week.28_rel.abund_t.2[,11:12]

#Convert dataframe into binary
dat.dol_water.fil.week.28.binary <- dat.dol_water.fil.week.28[,4:1471]

dat.dol_water.fil.week.28.binary []  <-  +(dat.dol_water.fil.week.28.binary  > 0)

#View(dat.dol_water.fil.week.28.binary [,1:10])

#Transpose dat.dol_water.fil.week.28.binary
dat.dol_water.fil.week.28.binary_t <- as.data.frame(t(dat.dol_water.fil.week.28.binary))
#View(dat.dol_water.fil.week.28.binary[1:10,])

#Create col Total.rel.abund.
dat.dol_water.fil.week.28.binary_t.2 <- dat.dol_water.fil.week.28.binary_t %>% 
  mutate (Total.rel.abund.zOTU = rowSums(dat.dol_water.fil.week.28.binary_t)/10)

#View(dat.dol_water.fil.week.28.binary_t.2[1:10,])

#Give dat.dol_water.fil.week.28.binary_t.2 rownames as col variable
dat.dol_water.fil.week.28.binary_t.2$variable <- rownames(dat.dol_water.fil.week.28.binary_t)
dim(dat.dol_water.fil.week.28.binary_t.2)
#1468 12

#Reorder
dat.dol_water.fil.week.28.binary_t.3 <- dat.dol_water.fil.week.28.binary_t.2[,c(12,11,1:10)]

#Join dat.dol_water.fil.week.28.binary_t.3 with attributes.dol_rdp_water.fil.2
Inter.core.zOTU.week.28 <- dat.dol_water.fil.week.28.binary_t.3 %>% left_join(attributes.dol_rdp_water.fil.2)
dim(Inter.core.zOTU.week.28)
#1468   18
#View(Inter.core.zOTU.week.28[1:10,])

#Reorder
Inter.core.zOTU.week.28_2 <- Inter.core.zOTU.week.28[,c(1,2,17,3:12)]
dim(Inter.core.zOTU.week.28_2)
##1468 13

names(Inter.core.zOTU.week.28_2)

#Order according to Inter.core.zOTU.week.28_2$Total.rel.abund.zOTU
Inter.core.zOTU.week.28_3 <- Inter.core.zOTU.week.28_2[order(Inter.core.zOTU.week.28_2$Total.rel.abund.zOTU),]
#View(Inter.core.zOTU.week.28_3[,1:2])

#Any inter.core.OTUs above 100% (== 1.0)?
Inter.core.zOTU.week.28_1.0 <- Inter.core.zOTU.week.28_3 %>% filter (Total.rel.abund.zOTU == 1.0)
dim(Inter.core.zOTU.week.28_1.0)
#8

#Determine relative abundance of those core zOTUs
Inter.core_week.28 <- Inter.core.zOTU.week.28_1.0$variable

Rel.abund_week.28.2 <- Rel.abund_week.28

rownames(Rel.abund_week.28.2) <- Rel.abund_week.28.2$variable

Rel.abund_week.28.Core <- Rel.abund_week.28.2[Inter.core_week.6,]

sum(Rel.abund_week.28.Core[,2])
#0.1932688

write.csv(Rel.abund_week.28.Core, 'Rel.abund_week.28.Core.csv')
```

###Week 37 (10 dolphins)


```
#####################
##37 (10 dolphins)
dat.dol_water.fil.week.37 <- 
  dat.dol_water.fil.t.19[c(61:70),]

#View(dat.dol_water.fil.week.37[,1:10])
dim(dat.dol_water.fil.week.37)
#10 1474

write.csv(dat.dol_water.fil.week.37, 'dat.dol_water.fil.week.37.csv')

###Relative abundance
dat.dol_water.fil.week.37.2 <- dat.dol_water.fil.week.37[,-c(1,3)]
dim(dat.dol_water.fil.week.37.2)
#10 1472

dat.dol_water.fil.week.37_rel.abund <- dat.dol_water.fil.week.37.2[,2:1472] /rowSums(dat.dol_water.fil.week.37.2[,2:1472])

#Transpose
dat.dol_water.fil.week.37_rel.abund_t <- as.data.frame(t(dat.dol_water.fil.week.37_rel.abund))

dat.dol_water.fil.week.37_rel.abund_t$variable <- rownames(dat.dol_water.fil.week.37_rel.abund_t)
dim(dat.dol_water.fil.week.37_rel.abund_t)
#1471   11

dat.dol_water.fil.week.37_rel.abund_t.2 <- dat.dol_water.fil.week.37_rel.abund_t %>% mutate(mean_of_rows = rowMeans(dat.dol_water.fil.week.37_rel.abund_t[,1:10]))

Rel.abund_week.37 <- dat.dol_water.fil.week.37_rel.abund_t.2[,11:12]

#Convert dataframe into binary
dat.dol_water.fil.week.37.binary <- dat.dol_water.fil.week.37[,4:1471]

dat.dol_water.fil.week.37.binary []  <-  +(dat.dol_water.fil.week.37.binary  > 0)

#View(dat.dol_water.fil.week.37.binary [,1:10])

#Transpose dat.dol_water.fil.week.37.binary
dat.dol_water.fil.week.37.binary_t <- as.data.frame(t(dat.dol_water.fil.week.37.binary))
#View(dat.dol_water.fil.week.37.binary[1:10,])

#Create col Total.rel.abund.
dat.dol_water.fil.week.37.binary_t.2 <- dat.dol_water.fil.week.37.binary_t %>% 
  mutate (Total.rel.abund.zOTU = rowSums(dat.dol_water.fil.week.37.binary_t)/10)

#View(dat.dol_water.fil.week.37.binary_t.2[1:10,])

#Give dat.dol_water.fil.week.37.binary_t.2 rownames as col variable
dat.dol_water.fil.week.37.binary_t.2$variable <- rownames(dat.dol_water.fil.week.37.binary_t)
dim(dat.dol_water.fil.week.37.binary_t.2)
#1468 12

#Reorder
dat.dol_water.fil.week.37.binary_t.3 <- dat.dol_water.fil.week.37.binary_t.2[,c(12,11,1:10)]

#Join dat.dol_water.fil.week.37.binary_t.3 with attributes.dol_rdp_water.fil.2
Inter.core.zOTU.week.37 <- dat.dol_water.fil.week.37.binary_t.3 %>% left_join(attributes.dol_rdp_water.fil.2)
dim(Inter.core.zOTU.week.37)
#1468   18
#View(Inter.core.zOTU.week.37[1:10,])

#Reorder
Inter.core.zOTU.week.37_2 <- Inter.core.zOTU.week.37[,c(1,2,17,3:12)]
dim(Inter.core.zOTU.week.37_2)
##1468 13

names(Inter.core.zOTU.week.37_2)

#Order according to Inter.core.zOTU.week.37_2$Total.rel.abund.zOTU
Inter.core.zOTU.week.37_3 <- Inter.core.zOTU.week.37_2[order(Inter.core.zOTU.week.37_2$Total.rel.abund.zOTU),]
#View(Inter.core.zOTU.week.37_3[,1:2])

#Any inter.core.OTUs above 100% (== 1.0)?
Inter.core.zOTU.week.37_1.0 <- Inter.core.zOTU.week.37_3 %>% filter (Total.rel.abund.zOTU == 1.0)
dim(Inter.core.zOTU.week.37_1.0)
#16

#Determine relative abundance of those core zOTUs
Inter.core_week.37 <- Inter.core.zOTU.week.37_1.0$variable

Rel.abund_week.37.2 <- Rel.abund_week.37

rownames(Rel.abund_week.37.2) <- Rel.abund_week.37.2$variable

Rel.abund_week.37.Core <- Rel.abund_week.37.2[Inter.core_week.6,]

sum(Rel.abund_week.37.Core[,2])
#0.1664256

write.csv(Rel.abund_week.37.Core, 'Rel.abund_week.37.Core.csv')
```


#######How many inter-core zOTUs did 10 dolphins at any sample collection point in time (weeks 2, 6, 11, 19, 28, 37)?


```
max(c(sum(Rel.abund_week.2.Core[,2]),sum(Rel.abund_week.11.Core[,2]),sum(Rel.abund_week.19.Core[,2]),sum(Rel.abund_week.28.Core[,2]),sum(Rel.abund_week.37.Core[,2]),sum(Rel.abund_week.6.Core[,2])))
```


```
[1] 0.4132573
```

###Create frequency histogram of inter-core


```
###Prepare dataframes (e.g., Inter.core.zOTU.week.2_1.0)
Inter.core.all.week.2 <- Inter.core.zOTU.week.2_1.0[,c(1,4)]
Inter.core.all.week.6 <- Inter.core.zOTU.week.6_1.0[,c(1,4)]
Inter.core.all.week.11 <- Inter.core.zOTU.week.11_1.0[,c(1,4)]
Inter.core.all.week.19 <- Inter.core.zOTU.week.19_1.0[,c(1,4)]
Inter.core.all.week.28 <- Inter.core.zOTU.week.28_1.0[,c(1,4)]
Inter.core.all.week.37 <- Inter.core.zOTU.week.37_1.0[,c(1,4)]


Inter.core.all.weeks <- full_join (by = c('variable'), Inter.core.all.week.2, Inter.core.all.week.6)
Inter.core.all.weeks.2 <- full_join (by = c('variable'), Inter.core.all.weeks, Inter.core.all.week.11)
Inter.core.all.weeks.3 <- full_join (by = c('variable'), Inter.core.all.weeks.2, Inter.core.all.week.19)
Inter.core.all.weeks.4 <- full_join (by = c('variable'), Inter.core.all.weeks.3, Inter.core.all.week.28)
Inter.core.all.weeks.5 <- full_join (by = c('variable'), Inter.core.all.weeks.4, Inter.core.all.week.37)

dim(Inter.core.all.weeks.5)
#97  7

Inter.core.all.weeks.6 <- Inter.core.all.weeks.5 %>% mutate (sum_of_rows=rowSums(Inter.core.all.weeks.5[,2:7],na.rm=TRUE)) %>% arrange(sum_of_rows)
#View(Inter.core.all.weeks.6)

Inter.core.histo <- as.data.frame(Inter.core.all.weeks.6$sum_of_rows)
names(Inter.core.histo) <- 'Inter.core.histo'

Inter.core.histogram =
  ggplot(Inter.core.histo, aes(x = Inter.core.histo)) +
  geom_histogram(binwidth = 0.5)+
  theme_bw() +
  theme(plot.title = element_text(size = 14, family = "Tahoma", face = "bold"),
        text = element_text(size = 12, family = "Tahoma"),
        axis.title = element_text(),
        axis.text.x=element_text(size = 11)) +
  scale_x_continuous(name = "Number of sampling points when inter-core zOTUs were found", 
                     breaks = seq(1:6)) +
  scale_y_continuous(name = "Number of inter-core zOTUs")

ggsave("Inter.core.histogram.jpg", plot = Inter.core.histogram , device = 'jpg', width = 168, height = 130, units = "mm",
       dpi = 300, limitsize = TRUE)
```


#######Temporal dynamics of inter\_core: Scatterplot

#######Impact of AB treatment (all levels) –> non-significant


```
###Check all dolphins for impact of AB treatment (None, Before, After, Directly_After)

###Prepare dataset, create cols ID, time and AB.Treatment.all.dol
dat.dol_water.fil.t.30 <- dat.dol_water.fil.t.2

dat.dol_water.fil.t.30$Dolphin   <- rownames(dat.dol_water.fil.t.30)
dat.dol_water.fil.t.31           <- dat.dol_water.fil.t.30
dat.dol_water.fil.t.31$Dolphin.2 <- dat.dol_water.fil.t.31$Dolphin
dim(dat.dol_water.fil.t.31)
#83 1473

dat.dol_water.fil.t.32 <- dat.dol_water.fil.t.31[,c(1472,1473,1:1471)]

dat.dol_water.fil.t.33 <- separate(dat.dol_water.fil.t.32,Dolphin.2, c('ID','time'))
dat.dol_water.fil.t.34 <- arrange(dat.dol_water.fil.t.33, Dolphin)
dat.dol_water.fil.t.35 <- dat.dol_water.fil.t.34[-c(2,43),]

###Add col 'AB.Treatment.all.dol'

AB.treatment <- as.data.frame(c(rep('None',11), rep('After',3),rep('Before',1),rep('After',1), rep('Directly_after',1), rep('After',3), rep('Before',1), rep('After',2), rep('Before',2), rep('None',11),
                                rep('Before',3), rep('Directly_after',1), rep('After',3),rep('Before',1), rep('None', 28), 
                                rep('Before',3), rep('Directly_after',1), rep('After',1), rep('Before',1), rep('After',2), rep('Before',1)))

names(AB.treatment)<- 'AB.Treatment.all.dol'
dim(AB.treatment)

dat.dol_water.fil.t.36 <- cbind(AB.treatment,dat.dol_water.fil.t.35)
#View(dat.dol_water.fil.t.36)
dim(dat.dol_water.fil.t.36)
#81 1475

dat.dol_water.fil.t.36$logTotalAbundance <- log(apply(dat.dol_water.fil.t.36[,5:1475],1,sum))

zOTU.water.fil.AB <- mvabund(dat.dol_water.fil.t.36[,5:1475])

Pre.fit1.water.fil.AB <- manyglm(zOTU.water.fil.AB ~ AB.Treatment.all.dol + time + ID + offset(logTotalAbundance), data = dat.dol_water.fil.t.36, family="negative.binomial")

#check assumptions
plot(Pre.fit1.water.fil.AB)

#This is the actual significance test               
fit.1.dol.tech.fil.AB = manyglm(zOTU.water.fil.AB ~ AB.Treatment.all.dol + time + ID + offset(logTotalAbundance), data = dat.dol_water.fil.t.36)
fit.2.dol.tech.fil.AB = manyglm(zOTU.water.fil.AB ~ time + ID + offset(logTotalAbundance), data = dat.dol_water.fil.t.36)

#adjusted
#globalTest1.dol.water.fil.adjusted.AB = anova(fit.1.dol.tech.fil.AB, fit.2.dol.tech.fil.AB, nBoot=1000, p.uni='adjusted')

# Multivariate test:
#                       Res.Df Df.diff  Dev Pr(>Dev)  
# fit.2.dol.tech.fil.AB     53                        
# fit.1.dol.tech.fil.AB     51       2 3071    0.097 .

#save(globalTest1.dol.water.fil.adjusted.AB, file='globalTest1.dol.water.fil.adjusted.AB.Rdata')
```

#######Impact of AB treatment (sick vs. healthy)


```
#View(dat.dol_water.fil.Treatment)
dim(dat.dol_water.fil.Treatment)
```


```
[1]   81 1475
```

#######nMDS plot for treated vs untreated dolphins

#######nMDS plot for treated vs untreated dolphins: Show all levels


#####Check dolphins for difference between ‘None‘ vs. ‘Before‘ —> Non-Significant


```
dat.dol_water.fil.t_None.Before <- filter(dat.dol_water.fil.t.36, !AB.Treatment.all.dol == 'After')

dat.dol_water.fil.t_None.Before.2 <- filter(dat.dol_water.fil.t_None.Before, !AB.Treatment.all.dol == 'Directly_after')

dat.dol_water.fil.t_None.Before.2$logTotalAbundance <- log(apply(dat.dol_water.fil.t_None.Before.2[,5:1475],1,sum))

zOTU.water.fil.AB_None.Before <- mvabund(dat.dol_water.fil.t_None.Before.2[,5:1475])

Pre.fit1.water.fil.AB_None.Before <- manyglm(zOTU.water.fil.AB_None.Before ~ AB.Treatment.all.dol + time + ID + offset(logTotalAbundance), data = dat.dol_water.fil.t_None.Before.2, family="negative.binomial")

#check assumptions
plot(Pre.fit1.water.fil.AB_None.Before)

#This is the actual significance test               
fit.1.dol.tech.fil.AB_None.Before = manyglm(zOTU.water.fil.AB_None.Before ~ AB.Treatment.all.dol + time + ID + offset(logTotalAbundance), data = dat.dol_water.fil.t_None.Before.2)
fit.2.dol.tech.fil.AB_None.Before = manyglm(zOTU.water.fil.AB_None.Before ~ time + ID + offset(logTotalAbundance), data = dat.dol_water.fil.t_None.Before.2)

#adjusted
#globalTest1.dol.water.fil.adjusted.AB_None.Before = anova(fit.1.dol.tech.fil.AB_None.Before, fit.2.dol.tech.fil.AB_None.Before, nBoot=1000, p.uni='adjusted')

# Multivariate test:
#                                   Res.Df Df.diff  Dev Pr(>Dev)  
# fit.1.dol.tech.fil.AB_None.Before     41                        
# fit.2.dol.tech.fil.AB_None.Before     41       0 11.1    0.077 

#save(globalTest1.dol.water.fil.adjusted.AB_None.Before, file='globalTest1.dol.water.fil.adjusted.AB_None.Before.Rdata')
```

#####Check dolphins for difference between ‘None‘ vs. ‘Directly\_After‘ –> Non-significant


```
dat.dol_water.fil.t_None.Directly_After <- filter(dat.dol_water.fil.t.36, !AB.Treatment.all.dol == 'After')

dat.dol_water.fil.t_None.Directly_After.2 <- filter(dat.dol_water.fil.t_None.Directly_After, !AB.Treatment.all.dol == 'Before')

dat.dol_water.fil.t_None.Directly_After.2$logTotalAbundance <- log(apply(dat.dol_water.fil.t_None.Directly_After.2[,5:1475],1,sum))

zOTU.water.fil.AB_None.Directly_After <- mvabund(dat.dol_water.fil.t_None.Directly_After.2[,5:1475])

Pre.fit1.water.fil.AB_None.Directly_After <- manyglm(zOTU.water.fil.AB_None.Directly_After ~ AB.Treatment.all.dol + time + ID + offset(logTotalAbundance), data = dat.dol_water.fil.t_None.Directly_After.2, family="negative.binomial")

#check assumptions
plot(Pre.fit1.water.fil.AB_None.Directly_After)

#This is the actual significance test               
fit.1.dol.tech.fil.AB_None.Directly_After <- manyglm(zOTU.water.fil.AB_None.Directly_After ~ AB.Treatment.all.dol + time + ID + offset(logTotalAbundance), data = dat.dol_water.fil.t_None.Directly_After.2)
fit.2.dol.tech.fil.AB_None.Directly_After <- manyglm(zOTU.water.fil.AB_None.Directly_After ~ time + ID + offset(logTotalAbundance), data = dat.dol_water.fil.t_None.Directly_After.2)

#adjusted
#globalTest1.dol.water.fil.adjusted.AB_None.Directly_After <- anova(fit.1.dol.tech.fil.AB_None.Directly_After, fit.2.dol.tech.fil.AB_None.Directly_After, nBoot=1000, p.uni='adjusted')

#Multivariate test:
#                                          Res.Df Df.diff   Dev Pr(>Dev)
#fit.2.dol.tech.fil.AB_None.Directly_After     42                       
#fit.1.dol.tech.fil.AB_None.Directly_After     41       1 881.8    0.105

#save(globalTest1.dol.water.fil.adjusted.AB_None.Directly_After, file='globalTest1.dol.water.fil.adjusted.AB_None.Directly_After.Rdata')
```

#####Check dolphins for difference between ‘None‘ vs. ‘After‘ –> non-significant


```
dat.dol_water.fil.t_None.After <- filter(dat.dol_water.fil.t.36, !AB.Treatment.all.dol == 'Before')

dat.dol_water.fil.t_None.After.2 <- filter(dat.dol_water.fil.t_None.After, !AB.Treatment.all.dol == 'Directly_after')

dat.dol_water.fil.t_None.After.2$logTotalAbundance <- log(apply(dat.dol_water.fil.t_None.After.2[,5:1475],1,sum))

zOTU.water.fil.AB_None.After <- mvabund(dat.dol_water.fil.t_None.After.2[,5:1475])

Pre.fit1.water.fil.AB_None.After <- manyglm(zOTU.water.fil.AB_None.After ~ AB.Treatment.all.dol + time + ID + offset(logTotalAbundance), data = dat.dol_water.fil.t_None.After.2, family="negative.binomial")

#check assumptions
plot(Pre.fit1.water.fil.AB_None.After)

#This is the actual significance test               
fit.1.dol.tech.fil.AB_None.After <- manyglm(zOTU.water.fil.AB_None.After ~ AB.Treatment.all.dol + time + ID + offset(logTotalAbundance), data = dat.dol_water.fil.t_None.After.2)
fit.2.dol.tech.fil.AB_None.After <- manyglm(zOTU.water.fil.AB_None.After ~ time + ID + offset(logTotalAbundance), data = dat.dol_water.fil.t_None.After.2)

#adjusted
#globalTest1.dol.water.fil.adjusted.AB_None.After = anova(fit.1.dol.tech.fil.AB_None.After, dat.dol_water.fil.t_None.After.2, nBoot=1000, p.uni='adjusted')

#Multivariate test:
#                                 Res.Df Df.diff   Dev Pr(>Dev)
#fit.1.dol.tech.fil.AB_None.After     41                       
#fit.2.dol.tech.fil.AB_None.After     41       0 20.32    0.108

#save(globalTest1.dol.water.fil.adjusted.AB_None.After, file='globalTest1.dol.water.fil.adjusted.AB_None.After.Rdata')
```

#####Check dolphins for difference between ‘Before‘ vs. ‘After‘ –> Not significant


```
dat.dol_water.fil.t_Before.After <- filter(dat.dol_water.fil.t.36, !AB.Treatment.all.dol == 'None')

dat.dol_water.fil.t_Before.After.2 <- filter(dat.dol_water.fil.t_Before.After, !AB.Treatment.all.dol == 'Directly_after')

dat.dol_water.fil.t_Before.After.2$logTotalAbundance <- log(apply(dat.dol_water.fil.t_Before.After.2[,5:1475],1,sum))

zOTU.water.fil.AB_Before.After <- mvabund(dat.dol_water.fil.t_Before.After.2[,5:1475])

Pre.fit1.water.fil.AB_Before.After <- manyglm(zOTU.water.fil.AB_Before.After ~ AB.Treatment.all.dol + time + ID + offset(logTotalAbundance), data = dat.dol_water.fil.t_Before.After.2, family="negative.binomial")

#check assumptions
plot(Pre.fit1.water.fil.AB_Before.After)

#This is the actual significance test               
fit.1.dol.tech.fil.AB_Before.After <- manyglm(zOTU.water.fil.AB_Before.After ~ AB.Treatment.all.dol + time + ID + offset(logTotalAbundance), data = dat.dol_water.fil.t_Before.After.2)
fit.2.dol.tech.fil.AB_Before.After <- manyglm(zOTU.water.fil.AB_Before.After ~ time + ID + offset(logTotalAbundance), data = dat.dol_water.fil.t_Before.After.2)

#adjusted
#globalTest1.dol.water.fil.adjusted.AB_Before.After = anova(fit.1.dol.tech.fil.AB_Before.After, fit.2.dol.tech.fil.AB_Before.After, nBoot=1000, p.uni='adjusted')

# Multivariate test:
#                                    Res.Df Df.diff  Dev Pr(>Dev)
# fit.2.dol.tech.fil.AB_Before.After      9                      
# fit.1.dol.tech.fil.AB_Before.After      8       1 2102    0.166

#save(globalTest1.dol.water.fil.adjusted.AB_Before.After, file='globalTest1.dol.water.fil.adjusted.AB_Before.After.Rdata')
```

#####Check dolphins for difference between ‘Before‘ vs. ‘Directly\_After‘ –> Just significant


```
dat.dol_water.fil.t_Before.Directly_After <- filter(dat.dol_water.fil.t.36, !AB.Treatment.all.dol == 'After')

dat.dol_water.fil.t_Before.Directly_After.2 <- filter(dat.dol_water.fil.t_Before.Directly_After, !AB.Treatment.all.dol == 'None')

dat.dol_water.fil.t_Before.Directly_After.2$logTotalAbundance <- log(apply(dat.dol_water.fil.t_Before.Directly_After.2[,5:1475],1,sum))

zOTU.water.fil.AB_Before.Directly_After <- mvabund(dat.dol_water.fil.t_Before.Directly_After.2[,5:1475])

Pre.fit1.water.fil.AB_Before.Directly_After <- manyglm(zOTU.water.fil.AB_Before.Directly_After ~ AB.Treatment.all.dol + time + ID + offset(logTotalAbundance), data = dat.dol_water.fil.t_Before.Directly_After.2, family="negative.binomial")

#check assumptions
plot(Pre.fit1.water.fil.AB_Before.Directly_After)

#This is the actual significance test               
fit.1.dol.tech.fil.AB_Before.Directly_After <- manyglm(zOTU.water.fil.AB_Before.Directly_After ~ AB.Treatment.all.dol + time + ID + offset(logTotalAbundance), data = dat.dol_water.fil.t_Before.Directly_After.2)
fit.2.dol.tech.fil.AB_Before.Directly_After <- manyglm(zOTU.water.fil.AB_Before.Directly_After ~ time + ID + offset(logTotalAbundance), data = dat.dol_water.fil.t_Before.Directly_After.2)

#adjusted
#globalTest1.dol.water.fil.adjusted.AB_Before.Directly_After = anova(fit.1.dol.tech.fil.AB_Before.Directly_After, fit.2.dol.tech.fil.AB_Before.Directly_After, nBoot=1000, p.uni='adjusted')

#Multivariate test:
#                                               Res.Df Df.diff   Dev Pr(>Dev)  
#fit.2.dol.tech.fil.AB_Before.Directly_After      4        
#fit.1.dol.tech.fil.AB_Before.Directly_After      3       1   1910    0.049 *

#save(globalTest1.dol.water.fil.adjusted.AB_Before.Directly_After, file='globalTest1.dol.water.fil.adjusted.AB_Before.Directly_After.Rdata')
```

#######nMDS plot for ‘Before‘ vs. ‘Directly\_After‘ –> Just significant


#####Check dolphins for difference between ‘After ‘ vs. ‘Directly\_After‘ –> Significant


```
dat.dol_water.fil.t_Directly_After.After <- filter(dat.dol_water.fil.t.36, !AB.Treatment.all.dol == 'None')

dat.dol_water.fil.t_Directly_After.After.2 <- filter(dat.dol_water.fil.t_Directly_After.After, !AB.Treatment.all.dol == 'Before')

dat.dol_water.fil.t_Directly_After.After.2$logTotalAbundance <- log(apply(dat.dol_water.fil.t_Directly_After.After.2[,5:1475],1,sum))

zOTU.water.fil.AB_Directly_After.After <- mvabund(dat.dol_water.fil.t_Directly_After.After.2[,5:1475])

Pre.fit1.water.fil.AB_None.Before <- manyglm(zOTU.water.fil.AB_Directly_After.After ~ AB.Treatment.all.dol + time + ID + offset(logTotalAbundance), data = dat.dol_water.fil.t_Directly_After.After.2, family="negative.binomial")

#check assumptions
plot(Pre.fit1.water.fil.AB_None.Before)

#This is the actual significance test               
fit.1.dol.tech.fil.AB_Directly_After.After <- manyglm(zOTU.water.fil.AB_Directly_After.After ~ AB.Treatment.all.dol + time + ID + offset(logTotalAbundance), data = dat.dol_water.fil.t_Directly_After.After.2)
fit.2.dol.tech.fil.AB_Directly_After.After <- manyglm(zOTU.water.fil.AB_Directly_After.After ~ time + ID + offset(logTotalAbundance), data = dat.dol_water.fil.t_Directly_After.After.2)

#adjusted
#globalTest1.dol.water.fil.adjusted.AB_Directly_After.After <- anova(fit.1.dol.tech.fil.AB_Directly_After.After, fit.2.dol.tech.fil.AB_Directly_After.After, nBoot=1000, p.uni='adjusted')

# Analysis of Deviance Table
# 
# fit.2.dol.tech.fil.AB_Directly_After.After: zOTU.water.fil.AB_Directly_After.After ~ time + ID + offset(logTotalAbundance)
# fit.1.dol.tech.fil.AB_Directly_After.After: zOTU.water.fil.AB_Directly_After.After ~ AB.Treatment.all.dol + time + ID + offset(logTotalAbundance)
# 
# Multivariate test:
#                                            Res.Df Df.diff  Dev Pr(>Dev)  
# fit.2.dol.tech.fil.AB_Directly_After.After      5                        
# fit.1.dol.tech.fil.AB_Directly_After.After      4       1 2545    0.016 *
  
#save(globalTest1.dol.water.fil.adjusted.AB_Directly_After.After, file='globalTest1.dol.water.fil.adjusted.AB_Directly_After.After.Rdata')
```

#######nMDS plot for ‘After ‘ vs. ‘Directly\_After‘ –> Just significant


#####Was there a common impact of Amoxycillin in Gemma and Nudgee? –> just not significant


```
###Is there a difference between Gemma and Nudgee (('directly_after', 'after') -> 'Amox') compared to the other dolphins that were not treated ('None')?

dat.dol_water.fil.Amox_G.N <-dat.dol_water.fil.t.36 %>% filter(!ID =='Howie')
dat.dol_water.fil.Amox_G.N.2 <-dat.dol_water.fil.Amox_G.N %>% filter(!ID =='Stella')

###Replace 'directly_after' & 'after' in Gemma and Nudgee with 'Amox' and 'Before' in Gemma and Nudgee with 'None'
dat.dol_water.fil.Amox_G.N.3 <- dat.dol_water.fil.Amox_G.N.2
dat.dol_water.fil.Amox_G.N.3$AB.Treatment.all.dol <- as.character(dat.dol_water.fil.Amox_G.N.3$AB.Treatment.all.dol)
dat.dol_water.fil.Amox_G.N.3[c(12:14,16,31:34),1] <- 'Amox'
dat.dol_water.fil.Amox_G.N.4 <- dat.dol_water.fil.Amox_G.N.3
dat.dol_water.fil.Amox_G.N.4[c(15,28:30,35),1] <- 'None'
dat.dol_water.fil.Amox_G.N.5 <- dat.dol_water.fil.Amox_G.N.4
dat.dol_water.fil.Amox_G.N.5$AB.Treatment.all.dol <- as.factor(dat.dol_water.fil.Amox_G.N.5$AB.Treatment.all.dol)

dat.dol_water.fil.Amox_G.N.5$logTotalAbundance <- log(apply(dat.dol_water.fil.Amox_G.N.5[,5:1475],1,sum))

zOTU.water.fil.Amox_G.N <- mvabund(dat.dol_water.fil.Amox_G.N.5[,5:1475])

Pre.fit1.water.fil.Amox_G.N <- manyglm(zOTU.water.fil.Amox_G.N ~ AB.Treatment.all.dol + time + ID + offset(logTotalAbundance), data = dat.dol_water.fil.Amox_G.N.5, family="negative.binomial")

#check assumptions
plot(Pre.fit1.water.fil.Amox_G.N)

#This is the actual significance test               
fit.1.dol.tech.fil.Amox_G.N <- manyglm(zOTU.water.fil.Amox_G.N ~ AB.Treatment.all.dol + time + ID + offset(logTotalAbundance), data = dat.dol_water.fil.Amox_G.N.5)
fit.2.dol.tech.fil.Amox_G.N <- manyglm(zOTU.water.fil.Amox_G.N ~ time + ID + offset(logTotalAbundance), data = dat.dol_water.fil.Amox_G.N.5)

#adjusted
#globalTest1.dol.water.fil.adjusted.Amox_G.N <- anova(fit.1.dol.tech.fil.Amox_G.N, fit.2.dol.tech.fil.Amox_G.N, nBoot=1000, p.uni='adjusted')

# Analysis of Deviance Table
# 
# fit.2.dol.tech.fil.Amox_G.N: zOTU.water.fil.Amox_G.N ~ time + ID + offset(logTotalAbundance)
# fit.1.dol.tech.fil.Amox_G.N: zOTU.water.fil.Amox_G.N ~ AB.Treatment.all.dol + time + ID + offset(logTotalAbundance)
# 
# Multivariate test:
#                             Res.Df Df.diff  Dev Pr(>Dev)  
# fit.2.dol.tech.fil.Amox_G.N     41                        
# fit.1.dol.tech.fil.Amox_G.N     40       1 1214    0.054 .
# ---
# Signif. codes:  0 ‘***’ 0.001 ‘**’ 0.01 ‘*’ 0.05 ‘.’ 0.1 ‘ ’ 1

#save(globalTest1.dol.water.fil.adjusted.Amox_G.N, file='globalTest1.dol.water.fil.adjusted.Amox_G.N.Rdata')
```

###Reviewer request: What would be the result of comparing a random subset of 4 healthy dolphins at different timepoints against the remaining healthy dolphins?


############2nd run


```
dim(dat.dol_water.fil.t.healthy.test2)
```


```
[1]   50 1477
```

############3rd run


```
###3rd run

#sick: Moki,  Sirius, Squeak, Starbuck, 
#Healthy: Coen, RB, Evie, Kiama, Scooter
#dat.dol_water.fil.t.healthy.4$ID
Test.3 <- c(rep('healthy',5),rep('healthy',6),rep('healthy',5), rep('sick',6), rep('healthy',6), rep('healthy',5), rep('sick',6), rep('sick',5), rep('sick',6))

dat.dol_water.fil.t.healthy.test3 <- cbind(Test.3,dat.dol_water.fil.t.healthy.4)

dim(dat.dol_water.fil.t.healthy.test3)
#50 1477

zOTU.water.fil.healthy.test3 <- mvabund(dat.dol_water.fil.t.healthy.test3[,6:1476])

Pre.fit1.water.fil.healthy.test3 <- manyglm(zOTU.water.fil.healthy.test3 ~ Test.3 + time + ID + offset(logTotalAbundance), data = dat.dol_water.fil.t.healthy.test3, family="negative.binomial")

#check assumptions
plot(Pre.fit1.water.fil.healthy.test3)

#This is the actual significance test               
fit.1.dol.tech.fil.healthy.test3 <- manyglm(zOTU.water.fil.healthy.test3 ~ Test.3 + time + ID + offset(logTotalAbundance), data = dat.dol_water.fil.t.healthy.test3)
fit.2.dol.tech.fil.healthy.test3 <- manyglm(zOTU.water.fil.healthy.test3 ~ time + ID + offset(logTotalAbundance), data = dat.dol_water.fil.t.healthy.test3)

#adjusted
#globalTest1.dol.water.fil.healthy.test3 <- anova(fit.1.dol.tech.fil.healthy.test3, fit.2.dol.tech.fil.healthy.test3, nBoot=1000, p.uni='adjusted')

# Multivariate test:
#                                  Res.Df Df.diff    Dev Pr(>Dev)
# fit.1.dol.tech.fil.healthy.test3     36                        
# fit.2.dol.tech.fil.healthy.test3     36       0 -0.042    0.963

#save(globalTest1.dol.water.fil.healthy.test3, file='globalTest1.dol.water.fil.healthy.test3.Rdata')
```


#####Is there a difference in Gemma and Nudgee between ‘Before’ vs. ‘Directly\_after/After’? –> Not significant


```
#####################################################
#####Is there a difference in Gemma and Nudgee between 'Before' vs. 'Directly_after/After'? --> Not significant

#Delete all rows that are not Gemma and Nudgee, convert 'Directly_after' into 'After', to compare 'Before' and 'After' only
dat.dol_water.fil.Amox_G.N_2 <-dat.dol_water.fil.t.36 %>% filter(!AB.Treatment.all.dol =='None')
dat.dol_water.fil.Amox_G.N_2.2 <-dat.dol_water.fil.Amox_G.N_2 %>% filter(!ID =='Stella')
dat.dol_water.fil.Amox_G.N_2.3 <-dat.dol_water.fil.Amox_G.N_2.2 %>% filter(!ID =='Howie')
dat.dol_water.fil.Amox_G.N_2.4 <- dat.dol_water.fil.Amox_G.N_2.3
dat.dol_water.fil.Amox_G.N_2.4[9,1] <- 'After'

dat.dol_water.fil.Amox_G.N_2.4$logTotalAbundance <- log(apply(dat.dol_water.fil.Amox_G.N_2.4[,5:1475],1,sum))

zOTU.water.fil.Amox_G.N_2 <- mvabund(dat.dol_water.fil.Amox_G.N_2.4[,5:1475])

Pre.fit1.water.fil.Amox_G.N_2 <- manyglm(zOTU.water.fil.Amox_G.N_2 ~ AB.Treatment.all.dol + time + ID + offset(logTotalAbundance), data = dat.dol_water.fil.Amox_G.N_2.4, family="negative.binomial")

#check assumptions
plot(Pre.fit1.water.fil.Amox_G.N_2)

#This is the actual significance test               
fit.1.dol.tech.fil.Amox_G.N_2 <- manyglm(zOTU.water.fil.Amox_G.N_2 ~ AB.Treatment.all.dol + time + ID + offset(logTotalAbundance), data = dat.dol_water.fil.Amox_G.N_2.4)
fit.2.dol.tech.fil.Amox_G.N_2 <- manyglm(zOTU.water.fil.Amox_G.N_2 ~ time + ID + offset(logTotalAbundance), data = dat.dol_water.fil.Amox_G.N_2.4)

#adjusted
#globalTest1.dol.water.fil.adjusted.Amox_G.N_2 = anova(fit.1.dol.tech.fil.Amox_G.N_2, fit.2.dol.tech.fil.Amox_G.N_2, nBoot=1000, p.uni='adjusted')

#Multivariate test:
#                              Res.Df Df.diff Dev Pr(>Dev)
#fit.1.dol.tech.fil.Amox_G.N_2      2                     
#fit.2.dol.tech.fil.Amox_G.N_2      2       0   0    0.999

#save(globalTest1.dol.water.fil.adjusted.Amox_G.N_2, file='globalTest1.dol.water.fil.adjusted.Amox_G.N_2.Rdata')
```


#####Did the pool water change over time? –> Significant


```
###Did the pool water change over time?
###Prepare dataset: pool samples only: dat.dol_t.19.t.fil.2

###Transpose
dat.dol_t.19.t.fil.2_t <- as.data.frame(t(dat.dol_t.19.t.fil.2))
dat.dol_t.19.t.fil.2_t.2 <- dat.dol_t.19.t.fil.2_t
dat.dol_t.19.t.fil.2_t.2$Pool <- rownames(dat.dol_t.19.t.fil.2_t.2)
dat.dol_t.19.t.fil.2_t.3 <- dat.dol_t.19.t.fil.2_t.2
dat.dol_t.19.t.fil.2_t.3$Pool.2 <- rownames(dat.dol_t.19.t.fil.2_t.2)
dim(dat.dol_t.19.t.fil.2_t.3)
#111 1993
dat.dol_t.19.t.fil.2_t.4 <- dat.dol_t.19.t.fil.2_t.3[,c(1992,1993,1:1991)]

dat.dol_t.19.t.fil.2_t.5 <- separate(dat.dol_t.19.t.fil.2_t.4, Pool.2, into = c("ID", "time"), sep = "_")

###Delete all dolphin samples
dat.dol_t.water <- dat.dol_t.19.t.fil.2_t.5[c(1:15,72:74),]
dat.dol_t.water.2 <- dat.dol_t.water
dim(dat.dol_t.water.2)

dat.dol_t.water.2$logTotalAbundance <- log(apply(dat.dol_t.water.2[,4:1994],1,sum))

zOTU.water <- mvabund(dat.dol_t.water.2[,4:1994])

Pre.fit1.water <- manyglm(zOTU.water ~ time + ID + offset(logTotalAbundance), data = dat.dol_t.water.2, family="negative.binomial")

#check assumptions
plot(Pre.fit1.water)

#This is the actual significance test               
fit.1.water <- manyglm(zOTU.water ~ time + ID + offset(logTotalAbundance), data = dat.dol_t.water.2)
fit.2.water <- manyglm(zOTU.water ~ ID + offset(logTotalAbundance), data = dat.dol_t.water.2)

#adjusted
#globalTest1.water = anova(fit.1.water, fit.2.water, nBoot=1000, p.uni='adjusted')

# Multivariate test:
#             Res.Df Df.diff   Dev Pr(>Dev)   
# fit.2.water     15                          
# fit.1.water      6       9 14064    0.002 **
# ---
# Signif. codes:  0 ‘***’ 0.001 ‘**’ 0.01 ‘*’ 0.05 ‘.’ 0.1 ‘ ’ 1

save(globalTest1.water, file='globalTest1.water.Rdata')
```

#####Did the pool water differ by pool? –> significant


```
###Did the pool water differ by pool?

#This is the actual significance test               
fit.1.water.ID <- manyglm(zOTU.water ~ time + ID + offset(logTotalAbundance), data = dat.dol_t.water.2)
fit.2.water.ID <- manyglm(zOTU.water ~ time + offset(logTotalAbundance), data = dat.dol_t.water.2)

#adjusted
globalTest1.water.ID = anova(fit.1.water.ID, fit.2.water.ID, nBoot=1000, p.uni='adjusted')

#Multivariate test:
#                   Res.Df   Df.diff  Dev Pr(>Dev)    
#fit.2.water.ID      8                          
#fit.1.water.ID      6       2     9987    0.001 ***


save(globalTest1.water.ID, file='globalTest1.water.ID.Rdata')
```

#####Does sex have an impact on dolphin blow microbiota? –> significant


```
###Use dat.dol_water.fil.t.36 & DolBlow_var

###Prepare DolBlow_var
DolBlow_var.10 <- DolBlow_var[,3:7]

###Join with DolBlow_var to add sex, pool and age data
dat.dol_water.fil.age.sex <- DolBlow_var.10 %>% left_join(dat.dol_water.fil.t.36[,-1])

dat.dol_water.fil.age.sex.2 <- dat.dol_water.fil.age.sex[-c(1:19,24,26:35,67:73,83:87),]

dat.dol_water.fil.age.sex.3 <- dat.dol_water.fil.age.sex.2 %>% separate(Water_sample, c('Pool','Week.2'))
dat.dol_water.fil.age.sex.4 <- dat.dol_water.fil.age.sex.3[,-c(4,6)]
rownames(dat.dol_water.fil.age.sex.4) <- dat.dol_water.fil.age.sex.4$Dolphin
dim(dat.dol_water.fil.age.sex.4)
#82 1478

dat.dol_water.fil.age.sex.4$logTotalAbundance <- log(apply(dat.dol_water.fil.age.sex.4[,8:1477],1,sum))

zOTU.sex <- mvabund(dat.dol_water.fil.age.sex.4[,8:1477])

Pre.fit1.sex <- manyglm(zOTU.sex ~ Sex + Age + Pool + offset(logTotalAbundance), data = dat.dol_water.fil.age.sex.4, family="negative.binomial")

#check assumptions
plot(Pre.fit1.sex)

#This is the actual significance test               
fit.1.sex <- manyglm(zOTU.sex ~ Sex + Age + Pool + offset(logTotalAbundance), data = dat.dol_water.fil.age.sex.4)
fit.2.sex <- manyglm(zOTU.sex ~ Age + Pool + offset(logTotalAbundance), data = dat.dol_water.fil.age.sex.4)

#adjusted
#globalTest1.sex = anova(fit.1.sex, fit.2.sex, nBoot=1000, p.uni='adjusted')

# Multivariate test:
#           Res.Df Df.diff  Dev Pr(>Dev)   
# fit.2.sex     55                         
# fit.1.sex     54       1 2128    0.002 **
# ---
# Signif. codes:  0 ‘***’ 0.001 ‘**’ 0.01 ‘*’ 0.05 ‘.’ 0.1 ‘ ’ 1

#save(globalTest1.sex, file='globalTest1.sex.Rdata')
```

#######nMDS plot for impact of sex have an impact on dolphin blow microbiota


#####Does age have an impact on dolphin blow microbiota? –> Significant


```
###Delete all rows with Age == 'NA'
dat.dol_water.fil.age.sex.5 <- dat.dol_water.fil.age.sex.4[-39,]
dat.dol_water.fil.age.sex.6 <- filter(dat.dol_water.fil.age.sex.5, !Age == 'NA')


zOTU.age <- mvabund(dat.dol_water.fil.age.sex.6[,8:1477])

#This is the actual significance test               
fit.1.age <- manyglm(zOTU.age ~ Sex + Age + offset(logTotalAbundance), data = dat.dol_water.fil.age.sex.6)
fit.2.age <- manyglm(zOTU.age ~ Sex +       offset(logTotalAbundance), data = dat.dol_water.fil.age.sex.6)

#adjusted
globalTest1.age = anova(fit.1.age, fit.2.age, nBoot=1000, p.uni='adjusted')

# Multivariate test:
#           Res.Df Df.diff  Dev Pr(>Dev)    
# fit.2.age     56                          
# fit.1.age     55       1 2762    0.001 ***

#save(globalTest1.age, file='globalTest1.age.Rdata')
```

#######nMDS plot for impact of age on dolphin blow microbiota

#######nMDS plot for impact of time on dolphin blow microbiota

#######nMDS plot for impact of ID on dolphin blow microbiota


#####Does the pool system have an impact on dolphin blow microbiota? –> Significant


```
#This is the actual significance test               
fit.1.pool <- manyglm(zOTU.sex ~ Sex + Age + Pool + offset(logTotalAbundance), data = dat.dol_water.fil.age.sex.4)
fit.2.pool <- manyglm(zOTU.sex ~ Sex + Age +        offset(logTotalAbundance), data = dat.dol_water.fil.age.sex.4)

#adjusted
#globalTest1.pool = anova(fit.1.pool, fit.2.pool, nBoot=1000, p.uni='adjusted')

#Multivariate test:
#           Res.Df Df.diff  Dev Pr(>Dev)    
#fit.2.pool     55                          
#fit.1.pool     54       1 1736    0.001 ***


#save(globalTest1.pool, file='globalTest1.pool.Rdata')
```

#####Create heatmap of those zOTUs that were identified as significantly different beetween the individual dolphins

LS0tDQp0aXRsZTogIkRvbHBoaW5fYmxvd191cGFyc2VfT2N0MjAyMF8yIg0Kb3V0cHV0OiBodG1sX25vdGVib29rDQotLS0NCg0KVGhpcyBpcyBhbiBbUiBNYXJrZG93bl0oaHR0cDovL3JtYXJrZG93bi5yc3R1ZGlvLmNvbSkgTm90ZWJvb2suIFdoZW4geW91IGV4ZWN1dGUgY29kZSB3aXRoaW4gdGhlIG5vdGVib29rLCB0aGUgcmVzdWx0cyBhcHBlYXIgYmVuZWF0aCB0aGUgY29kZS4gDQoNClRyeSBleGVjdXRpbmcgdGhpcyBjaHVuayBieSBjbGlja2luZyB0aGUgKlJ1biogYnV0dG9uIHdpdGhpbiB0aGUgY2h1bmsgb3IgYnkgcGxhY2luZyB5b3VyIGN1cnNvciBpbnNpZGUgaXQgYW5kIHByZXNzaW5nICpDdHJsK1NoaWZ0K0VudGVyKi4gDQoNCkFkZCBhIG5ldyBjaHVuayBieSBjbGlja2luZyB0aGUgKkluc2VydCBDaHVuayogYnV0dG9uIG9uIHRoZSB0b29sYmFyIG9yIGJ5IHByZXNzaW5nICpDdHJsK0FsdCtJKi4NCg0KV2hlbiB5b3Ugc2F2ZSB0aGUgbm90ZWJvb2ssIGFuIEhUTUwgZmlsZSBjb250YWluaW5nIHRoZSBjb2RlIGFuZCBvdXRwdXQgd2lsbCBiZSBzYXZlZCBhbG9uZ3NpZGUgaXQgKGNsaWNrIHRoZSAqUHJldmlldyogYnV0dG9uIG9yIHByZXNzICpDdHJsK1NoaWZ0K0sqIHRvIHByZXZpZXcgdGhlIEhUTUwgZmlsZSkuDQoNClRoZSBwcmV2aWV3IHNob3dzIHlvdSBhIHJlbmRlcmVkIEhUTUwgY29weSBvZiB0aGUgY29udGVudHMgb2YgdGhlIGVkaXRvci4gQ29uc2VxdWVudGx5LCB1bmxpa2UgKktuaXQqLCAqUHJldmlldyogZG9lcyBub3QgcnVuIGFueSBSIGNvZGUgY2h1bmtzLiBJbnN0ZWFkLCB0aGUgb3V0cHV0IG9mIHRoZSBjaHVuayB3aGVuIGl0IHdhcyBsYXN0IHJ1biBpbiB0aGUgZWRpdG9yIGlzIGRpc3BsYXllZC4NCg0KDQojIyMjIyMjIyMjIyMjIw0KIyMjIyMjIyMjIyMjRGlyZWN0b3JpZXMNCg0KYGBge3J9DQojSGFyb2xkDQpzZXR3ZCgiWjovU0NJL0JFRVMvTU1SL1N0dWRlbnRzX1Bvc3Rkb2NzL0NhdC9QaEQvRG9scGhpbiBibG93X1NlYSB3b3JsZC9TdGF0aXN0aWNzXzIvT3V0cHV0LmZpbGVzX3VwYXJzZV9zdGF0cy5kb2xwaGluIikNCg0KI0phY2sNCiNzZXR3ZCgiWjovU3R1ZGVudHNfUG9zdGRvY3MvQ2F0L1BoRC9Eb2xwaGluIGJsb3dfU2VhIHdvcmxkL1N0YXRpc3RpY3NfMi9PdXRwdXQuZmlsZXNfdXBhcnNlX3N0YXRzLmRvbHBoaW4iKQ0KDQpgYGANCg0KIyMjIyMjIyMjIyMjIyMjIyMjIyMNCiMjIyMjIyMjIyMjI0xvYWQgbGlicmFyaWVzDQoNCmBgYHtyfQ0KbGlicmFyeShzZXFpbnIpDQpsaWJyYXJ5KHBoZWF0bWFwKQ0KbGlicmFyeShwcmFjbWEpDQpsaWJyYXJ5KHBseXIpDQpsaWJyYXJ5KGdncGxvdDIpDQpsaWJyYXJ5KEJpb2NJbnN0YWxsZXIpDQpsaWJyYXJ5KFJDb2xvckJyZXdlcikNCmxpYnJhcnkobWljcm9iaW9tZSkNCmxpYnJhcnkoaGFibGFyKQ0KbGlicmFyeShkaWdlc3QpDQpsaWJyYXJ5KE1BU1MpDQpsaWJyYXJ5KG12YWJ1bmQpDQpsaWJyYXJ5KHJlc2hhcGUyKQ0KbGlicmFyeShHR2FsbHkpDQpsaWJyYXJ5KHZlZ2FuKQ0KbGlicmFyeSh0aWR5cikNCmxpYnJhcnkocGlsbGFyKQ0KbGlicmFyeSh0aWJibGUpDQpsaWJyYXJ5KHBoeWxvc2VxKQ0KbGlicmFyeShkYXRhLnRhYmxlKQ0KbGlicmFyeShzdHJpbmdpKQ0KbGlicmFyeShmYW5zaSkNCmxpYnJhcnkobmxtZSkNCmxpYnJhcnkoZm9zc2lsKQ0KbGlicmFyeShkcGx5cikNCmxpYnJhcnkoUkxSc2ltKQ0KbGlicmFyeShnZ2ZvcnRpZnkpDQpsaWJyYXJ5KGFuY2hvcnMpDQpsaWJyYXJ5KGRlY29udGFtKTsgcGFja2FnZVZlcnNpb24oImRlY29udGFtIikNCmxpYnJhcnkoYXJtKQ0KbGlicmFyeShnbG1tVE1CKQ0KbGlicmFyeShwdXJycikNCg0KYGBgDQoNCiMjIyMjIyMjIyMjIyMjIyMjIyMjDQojIyMjIyMjIyMjIyNTYXZlIG15RW52aXJvbm1lbnQNCmBgYHtyfQ0KI3NhdmUuaW1hZ2UoZmlsZT0nbXlFbnZpcm9ubWVudC5Eb2xwaGluYmxvd181LlJEYXRhJykNCmxvYWQoJ215RW52aXJvbm1lbnQuRG9scGhpbmJsb3dfNS5SRGF0YScpDQpgYGANCg0KIyMjIyMjIyMjIyMjIyMjIyMjIyMNCiMjIyMjIyMjIyMjI1JlYWQgaW4gZGF0YXNldHMNCmBgYHtyfQ0KIyMjek9UVSB0YWJsZQ0KZGF0LmRvbC5mdWxsID0gcmVhZC5jc3YoIkFsbFNhbXBsZXNfdW5vaXNlX290dV90YWJsZS5TaWx2YS5tb2RfMi5jc3YiKQ0KDQojTWV0YWRhdGENCkRvbEJsb3dfdmFyID0gcmVhZC5jc3YoIkRvbEJsb3dfdmFyLjMuY3N2IiwgaGVhZGVyPVQsc2VwPScsJykNCmBgYA0KDQojIyMjIyMjIyMjIyMjIyMjIyMjIw0KIyMjIyMjIyMjIyMjUHJvY2VzcyBkYXRhc2V0cw0KYGBge3J9DQpkaW0oZGF0LmRvbC5mdWxsKQ0KIzIyNTMgIDEzMQ0KI1ZpZXcoZGF0LmRvbC5mdWxsWzE6MTAsXSkNCiNWaWV3KGRhdC5kb2wuZnVsbFsxMjY6MTMxLF0pDQojVmlldyhEb2xCbG93X3ZhcikNCmRpbShEb2xCbG93X3ZhcikNCiMxMjQgICA4DQoNCiMjI0ZpbHRlciBvdXQgT1RVIHRoYXQgYXJlIGNobG9yb3BsYXN0cyEhIQ0KZGF0LmRvbC5mdWxsLjIgPSBkYXQuZG9sLmZ1bGwgJT4lIGZpbHRlcighT3JkZXI9PSdDaGxvcm9wbGFzdCcpDQpkYXQuZG9sLmZ1bGwuMyA9IGRhdC5kb2wuZnVsbC4yICU+JSBmaWx0ZXIoIUZhbWlseT09J01pdG9jaG9uZHJpYScpDQoNCmRpbShkYXQuZG9sLmZ1bGwuMikNCiMtLT4gNTYgQ2hsb3JvcGxhc3Qgek9UVXMgd2VyZSBkZWxldGVkISEhDQoNCmRpbShkYXQuZG9sLmZ1bGwuMykNCg0KIy0tPiAyMyBNaXRvY2hvbmRyaWEgT1RVcyB3ZXJlIGRlbGV0ZWQhISENCg0KIyNTcGxpdCBTaWx2YS10YXggZnJvbSBkYXQuZG9sLmZ1bGwuMw0KYXR0cmlidXRlcy5kb2wgPSBkYXQuZG9sLmZ1bGwuM1ssYygxLDEyNjoxMzEpXQ0KZGltKGF0dHJpYnV0ZXMuZG9sKQ0KI1ZpZXcoYXR0cmlidXRlcy5kb2wpDQoNCiNDb252ZXJ0IGF0dHJpYnV0ZXMuZG9sIGJhY2sgaW50byBjaGFyYWN0ZXJzDQphdHRyaWJ1dGVzLmRvbC4yID0gZGF0YS5mcmFtZShsYXBwbHkoYXR0cmlidXRlcy5kb2wsIGFzLmNoYXJhY3RlciksIHN0cmluZ3NBc0ZhY3RvcnM9RkFMU0UpDQoNCiNDb252ZXJ0IHZhcmlhYmxlIGludG8gcm93bmFtZXMgaW4gRG9scGhpbi5hdHRyaWJ1dGVzLjcgYW5kIGRlbGV0ZSBjb2wgMQ0KYXR0cmlidXRlcy5kb2wuMyA9IGF0dHJpYnV0ZXMuZG9sLjINCnJvd25hbWVzKGF0dHJpYnV0ZXMuZG9sLjMpID0gYXR0cmlidXRlcy5kb2wuM1ssMV0NCmF0dHJpYnV0ZXMuZG9sLjQgPSBhdHRyaWJ1dGVzLmRvbC4zWywtMV0NCg0KI1JlYWQgaW4gcmRwLnRheA0KYXR0cmlidXRlcy5kb2xfcmRwID0gcmVhZC5jc3YoIkFsbFNhbXBsZXNfdW5vaXNlX25iY19yZHAuY3N2IiwgZmlsbD1UUlVFLCBoZWFkZXI9VCwgc2VwPScsJykNCmRpbShhdHRyaWJ1dGVzLmRvbF9yZHApDQoNCg0KI0RlbGV0ZSB0YXggb2YgZnVsbC5kYXQubWlncmF0aW9uDQpkYXQuZG9sID0gZGF0LmRvbC5mdWxsLjNbLC1jKDEyNjoxMzEpXQ0KZGltKGRhdC5kb2wpDQoNCg0KI1R1cm4gY29sICd2YXJpYWJsZScgaW50byByb3duYW1lcw0KZGF0LmRvbC4yID0gZGF0LmRvbA0KI3Jvd25hbWVzKGRhdC5kb2wuMikgPSBkYXQuZG9sLjJbLDFdDQpkaW0oZGF0LmRvbC4yKQ0KDQojVmlldyhkYXQuZG9sLjJbMToxMCxdKQ0KDQojQ3JlYXRlIGNvbCAnc3VtX29mX2NvdW50cycNCmRhdC5kb2wuMyA9IG11dGF0ZSAoZGF0LmRvbC4yLCBzdW1fb2Zfcm93cz1yb3dTdW1zKGRhdC5kb2wuMlssMjoxMjVdKSkNCmRpbShkYXQuZG9sLjMpDQoNCg0KI2ZpbHRlciByb3dzIHdpdGggJ3N1bV9vZl9yb3dzJyB0aGF0IGFyZSBzbWFsbGVyIHRoYW4gMiANCiMocmVtb3ZhbCBvZiBPVFVzIHdpdGhvdXQgYW55IGNvdW50cyBhbmQgb2Ygc2luZ2xldG9ucykNCmRhdC5kb2wuNCA9IGRhdC5kb2wuMyAlPiUgZmlsdGVyKHN1bV9vZl9yb3dzID4gMSkNCmRpbShkYXQuZG9sLjQpDQoNCiMtLT4gMjMgek9UVXMgaGFkIGEgY291bnQgb2YgMCBvciAxIQ0KDQojRGV0ZXJtaW5lIHRvdGFsIG51bWJlciBvZiByZWFkcw0Kc3VtKGRhdC5kb2wuNCRzdW1fb2Zfcm93cykNCiMgNCwxMDEsMTIyDQoNCiNHaXZlIGRhdC5pbnRlcmFubi4zIHJvd25hbWVzIGJhY2sNCmRhdC5kb2wuNSA9IGRhdC5kb2wuNA0Kcm93bmFtZXMoZGF0LmRvbC41KSA9IGRhdC5kb2wuNSR2YXJpYWJsZQ0KDQojRGVsZXRlIGNvbHMgMSAodmFyaWFibGUpIGFuZCAxMjYgKHN1bV9vZl9yb3dzKQ0KZGF0LmRvbC42ID0gZGF0LmRvbC41WywtYygxLDEyNildDQoNCiNUcmFuc3Bvc2UgdGhlIG1hdHJpeA0KZGF0LmRvbF90ID0gYXMuZGF0YS5mcmFtZSh0KGRhdC5kb2wuNikpDQpkaW0oZGF0LmRvbF90KQ0KIzEyNCAyMTUxDQoNCmRhdC5kb2xfdC4yID0gZGF0LmRvbF90DQpkYXQuZG9sX3QuMiRTYW1wbGUgPSByb3duYW1lcyhkYXQuZG9sX3QuMikNCndoaWNoKGNvbG5hbWVzKGRhdC5kb2xfdC4yKT09J1NhbXBsZScpDQojMjE1Mg0KDQojUmVvcmRlciB0byBicmluZyBjb2wgJ1NhbXBsZScgdG8gZnJvbnQNCmRhdC5kb2xfdC4zID0gZGF0LmRvbF90LjJbLGMoMjE1MiwxOjIxNTEpXQ0KZGltKGRhdC5kb2xfdC4zKQ0KIzEyNCAyMTUyDQojVmlldyhkYXQuZG9sX3QuM1ssMToxMF0pDQoNCiNPbmx5IGtlZXAgZmlyc3QgMyBjb2xzIGZvciBub3cNCkRvbEJsb3dfdmFyLjIgPSBEb2xCbG93X3ZhclssMTozXQ0KDQojSm9pbiBkYXQuZG9sX3QuMyB3aXRoIERvbEJsb3dfdmFyDQpkYXQuZG9sX3QuNCA9IGRhdC5kb2xfdC4zICU+JSBsZWZ0X2pvaW4oRG9sQmxvd192YXIuMikNCmRpbShkYXQuZG9sX3QuNCkNCiMxMjkgMjE1NA0KDQojUmVvcmRlciBkYXQuZG9sX3QuNA0KZGF0LmRvbF90LjUgPSBkYXQuZG9sX3QuNFssYygxLDIxNTM6MjE1NCwyOjIxNTIpXQ0KI1ZpZXcoZGF0LmRvbF90LjVbLDE6MTBdKQ0KDQojRGVsZXRlIGNvbCAxLyAkU2FtcGxlDQpkYXQuZG9sX3QuNiA9IGRhdC5kb2xfdC41WywtMV0NCiNWaWV3KGRhdC5kb2xfdC42WywxOjEwXSkNCg0KIyMjRm9yIGNhbGN1bGF0aW5nIHJlbGF0aXZlIGFidW5kYW5jZSwgZ2V0IHJpZCBvZiBmaXJzdCB0d28gY29sdW1uIChhcyB0aGVzZSBhcmUgY2hhcmFjdGVycyksIEdpdmUgZGF0LmRvbF90LjYgcm93bmFtZXMNCmRhdC5kb2xfdC43ID0gZGF0LmRvbF90LjYNCnJvd25hbWVzKGRhdC5kb2xfdC43KSA9IGRhdC5kb2xfdC43JERvbHBoaW4NCmRhdC5kb2xfdC44ID0gZGF0LmRvbF90LjcgWywtYygxLDIpXQ0KI1ZpZXcoZGF0LmRvbF90LjhbLDE6MTBdKQ0KZGltKGRhdC5kb2xfdC44KQ0KIzEyNCAyMTUxDQoNCiNMb29rIGF0IHJvd1N1bXMgb2YgZGF0LmRvbF90LjgNCiNWaWV3KHJvd1N1bXMoZGF0LmRvbF90LjgpKQ0KDQptaW4ocm93U3VtcyhkYXQuZG9sX3QuOCkpDQojMTMsMTAyDQoNCiNDYWxjdWxhdGUgcmVsYXRpdmUgYWJ1bmRhbmNlDQpkYXQuZG9sX3QucmVsLmFidW5kID0gZGF0LmRvbF90Ljgvcm93U3VtcyhkYXQuZG9sX3QuOCkNCiNWaWV3KGRhdC5kb2xfdC5yZWwuYWJ1bmRbLDE6MTBdKQ0KDQojUmVtb3ZlIE9UVXMgd2l0aCBsZXNzIHRoYW4gMC4wMDAxJSBvZiB0b3RhbCByZWwuIGFidW5kYW5jZQ0KZGF0LmRvbF90LnJlbC5hYnVuZC5hYnVuZCA9IGRhdC5kb2xfdC5yZWwuYWJ1bmRbLGNvbFN1bXMoZGF0LmRvbF90LnJlbC5hYnVuZCkgPjAuMDAwMV0NCmRpbShkYXQuZG9sX3QucmVsLmFidW5kLmFidW5kKQ0KIyAxMjQgMjE0OA0KDQojLS0+IFdlIGRlbGV0ZWQgMyB6T1RVcyB0aGF0IGhhZCByZWwuIGFidW5kIGJlbG93IDAuMDAwMQ0KDQp6T1RVcy5hYnVuZCA9IG5hbWVzKGRhdC5kb2xfdC5yZWwuYWJ1bmQuYWJ1bmQpDQoNCiNGaWx0ZXIgcmFyZSB6T1RVcyBmcm9tIHJhdyBjb3VudCBkYXRhZnJhbWUgKGRhdC5kb2xfdC44KQ0KZGF0LmRvbF90LjkgPSBkYXQuZG9sX3QuOFssek9UVXMuYWJ1bmRdDQpkaW0oZGF0LmRvbF90LjkpDQojIDEyNCAyMTQ4DQojVmlldyhkYXQuZG9sX3QuOVssMToxMF0pDQoNCmRhdC5kb2xfdC4xMCA9IGRhdC5kb2xfdC45DQpkYXQuZG9sX3QuMTAkRG9scGhpbiA9IHJvd25hbWVzKGRhdC5kb2xfdC4xMCkNCiNWaWV3KGRhdC5kb2xfdC4xMFssMToxMF0pDQoNCiMjIyMjIyMjIyMjIyMjIyMjIyMjIyMjIyMjIyMjIyMjIyMjIyMjIyMjIyMjIyMjIyMjIyMjIyMjIyMjDQojIyNDaGVjayBudW1iZXIgb2YgY291bnRzIG9mIHNhbXBsZXM6DQpkaW0oZGF0LmRvbF90LjEwKQ0KIzEyNCAyMTQ5DQoNCiNSZW9yZGVyIGFuZCBicmluZyBjb2wgJGRvbHBoaW4gdG8gdGhlIGZyb250DQpkYXQuZG9sX3QuMTEgPC0gZGF0LmRvbF90LjEwWyxjKDIxNDksMToyMTQ4KV0NCg0KI0NyZWF0ZSBzdW0gb2Ygcm93cw0KZGF0LmRvbF90LjEyID0gbXV0YXRlIChkYXQuZG9sX3QuMTEsIHN1bV9vZl9yb3dzPXJvd1N1bXMoZGF0LmRvbF90LjExWywyOjIxNDldKSkNCmRpbShkYXQuZG9sX3QuMTIpDQojMTI0IDIxNTANCg0KI1Jlb3JkZXINCmRhdC5kb2xfdC4xMyA8LSBkYXQuZG9sX3QuMTJbLGMoMjE1MCwxOjIxNDkpXQ0KDQojIyMjIyMjIyMjIyMjIyMjIyMjIyMjIyMjIyMjIyMjIyMjIyMjIyMjIyMjIyMjIyMjIyMjIyMjIyMjDQojIyMjI2RhdC5kb2xfdC4xMCBoYXJib3VycyBhIHRvdGFsIG9mIDIsMTQ4IHpPVFVzLg0KIyMjIyMjIyMjIyMjIyMjIyMjIyMjIyMjIyMjIyMjIyMjIyMjIyMjIyMjIyMjIyMjIyMjIyMjIyMjIw0KYGBgDQoNCiMjIyMjIyMjIyMjIyMjIyMjIyMjIyMjIyMjIyMjIyMjIyMjIyMjIyMjDQojIyNEZXRlcm1pbmUgcmljaG5lc3MgYW5kIG51bWJlciBvZiByZWFkcyBvZiB0ZWNoIGNvbnRyb2xzDQpgYGB7cn0NCmRpbShkYXQuZG9sX3QuOSkNCiMxMjQgMjE0OA0KDQojVHJhbnNwb3NlIGRhdC5kb2xfdC45DQpkYXQuZG9sX3QudCA9IGFzLmRhdGEuZnJhbWUodChkYXQuZG9sX3QuOSkpDQojVmlldyhkYXQuZG9sX3QudFssMToxMF0pDQojbmFtZXMoZGF0LmRvbF90LnQpDQoNCiNLZWVwIGNvbHMgd2l0aCB0ZWNoIGNvbnRyb2xzIG9ubHkNCmRhdC5kb2xfdC50LnRlY2ggPSBkYXQuZG9sX3QudFssYyg4MTo4NiwxMDg6MTExLDExNToxMTcpXQ0KI25hbWVzKGRhdC5kb2xfdC50LnRlY2gpDQpkaW0oZGF0LmRvbF90LnQudGVjaCkNCiMyMTQ4ICAgMTMNCg0KRG9sQmxvd192YXIkRG9scGhpbg0KDQpEb2xCbG93X3Zhci50ZWNoID0gRG9sQmxvd192YXJbYygxNjoxOSw2Nzo3Myw4Mzo4NCksMjozXQ0KZGltKERvbEJsb3dfdmFyLnRlY2gpDQojMTMgIDINCg0KRG9sQmxvd192YXIudGVjaC4yID0gRG9sQmxvd192YXIudGVjaA0KI3Jvd25hbWVzKERvbEJsb3dfdmFyLnRlY2guMikgPSBEb2xCbG93X3Zhci50ZWNoLjIkRG9scGhpbg0KDQpkYXQuZG9sX3QudC50ZWNoLjIgPSBkYXQuZG9sX3QudC50ZWNoDQpkYXQuZG9sX3QudC50ZWNoLjIkdmFyaWFibGUgPSByb3duYW1lcyhkYXQuZG9sX3QudC50ZWNoLjIpDQpkaW0oZGF0LmRvbF90LnQudGVjaC4yKQ0KIzIxNDggICAxNA0KDQpkYXQuZG9sX3QudC50ZWNoLjMgPSBkYXQuZG9sX3QudC50ZWNoLjIgJT4lIA0KICBtdXRhdGUoc3VtX29mX3Jvd3MgPSByb3dTdW1zKGRhdC5kb2xfdC50LnRlY2guMlssMToxM10pKQ0KDQpkYXQuZG9sX3QudC50ZWNoLjQgPSBkYXQuZG9sX3QudC50ZWNoLjMgJT4lIGZpbHRlciAoc3VtX29mX3Jvd3MgPiAwKQ0KDQpkaW0oZGF0LmRvbF90LnQudGVjaC40KQ0KIyAzODUgMTUNCg0Kek9UVXMudGVjaCA9IGRhdC5kb2xfdC50LnRlY2guNCR2YXJpYWJsZQ0KDQojIyN6T1RVcyB0aGF0IGFyZSBwcmVzZW50IGluIHRlY2guIGNvbnRyb2xzDQp6T1RVcy50ZWNoDQpsZW5ndGgoek9UVXMudGVjaCkNCiMzODUNCmBgYA0KDQojIyMjIyMjIyMjIyMjIyMjIyMjIyMjIyMjIyMjIyMjIyMjIyMjIyMjIyMjIyMjIyMjIyMjIyMjIyMjIyMjIyMjIyMjIyMjIyMjIyMjIyMjIyMjIyMjIyMjIyMjIyMjIyMjIyMjIyMjIyMjIyMjIyMjIyMjIyMjIyMjIyMjIyMjIyMNCiMjI0NyZWF0ZSBwaHlsb3NlcS1jbGFzcyBvYmplY3QgdG8gZmlsdGVyIHRlY2huaWNhbCBjb250YW1pbmFudHMNCg0KYGBge3J9DQojIyMjIyMjIyMjIyMjIyMjIyMjDQojIyNNZXRhZGF0YTogRG9sQmxvd192YXINCg0KI1Byb3ZpZGUgRG9sQmxvd192YXIgd2l0aCBjb2wgJ1NhbXBsZV9vcl9Db250cm9sJywgcmVtb3ZlIGFsbCB3YXRlciBzYW1wbGVzLCBhbmQgcmVuYW1lIGNvbCAnRG9scGhpbicgdG8gWC5TYW1wbGVJRA0KRG9sQmxvd192YXJfbm8ud2F0ZXIgPC0gRG9sQmxvd192YXIgJT4lIGZpbHRlcighU3BlY2llcyA9PSAnV2F0ZXInKQ0KI1ZpZXcoRG9sQmxvd192YXJfbm8ud2F0ZXIpDQoNClNhbXBsZV9vcl9Db250cm9sIDwtIGFzLmRhdGEuZnJhbWUoYyhyZXAoJ0NvbnRyb2wnLCA0KSwgcmVwKCdUcnVlIFNhbXBsZScsIDM3KSwgcmVwKCdDb250cm9sJywgNyksIHJlcCgnVHJ1ZSBTYW1wbGUnLCA5KSwgcmVwKCdDb250cm9sJywgMiksIHJlcCgnVHJ1ZSBTYW1wbGUnLCAzNykpKQ0KDQpuYW1lcyhTYW1wbGVfb3JfQ29udHJvbCkgPC0gJ1NhbXBsZV9vcl9Db250cm9sJw0KDQpEb2xCbG93X3Zhcl9uby53YXRlci4yIDwtIGNiaW5kKFNhbXBsZV9vcl9Db250cm9sLCBEb2xCbG93X3Zhcl9uby53YXRlcikNCiNWaWV3KERvbEJsb3dfdmFyX25vLndhdGVyLjIpDQoNCmNvbG5hbWVzKERvbEJsb3dfdmFyX25vLndhdGVyLjIpWzRdIDwtICdYLlNhbXBsZUlEJw0KDQojTGVhdmUgaW4gY29scyAnU2FtcGxlX29yX0NvbnRyb2wnIGFuZCAnWC5TYW1wbGVJRCcgb25seQ0KRG9sQmxvd192YXJfbm8ud2F0ZXIuMyA8LSBEb2xCbG93X3Zhcl9uby53YXRlci4yWyxjKDEsNCldDQojVmlldyhEb2xCbG93X3Zhcl9uby53YXRlci4zKQ0KRG9sQmxvd192YXJfbm8ud2F0ZXIuMyA8LSBhcy5kYXRhLmZyYW1lKERvbEJsb3dfdmFyX25vLndhdGVyLjMpDQoNCnJvd25hbWVzKERvbEJsb3dfdmFyX25vLndhdGVyLjMpIDwtIERvbEJsb3dfdmFyX25vLndhdGVyLjMkWC5TYW1wbGVJRA0KYGBgDQoNCiMjIyMjIyMjIyMjIyMjIyMjIyMjIyMjIyMjIyMjIyMjIyMjIyMjIyMjIyMjIyMjIyMjDQojIyNEZWxldGUgc3VycGx1cyB6T1RVcyBmcm9tIHRheG9ub215IHRhYmxlOiBhdHRyaWJ1dGVzLmRvbF9yZHAgKG1pdG9jaG9uZHJpYSwgY2hsb3JvcGxhc3RzKQ0KDQpgYGB7cn0NCmRpbShhdHRyaWJ1dGVzLmRvbF9yZHApDQojMjI4MiAgICA3DQoNCmF0dHJpYnV0ZXMuZG9sX3JkcC4yIDwtIHN1YnNldChhdHRyaWJ1dGVzLmRvbF9yZHAsIHZhcmlhYmxlICVpbiUgek9UVXMuYWJ1bmQpDQpkaW0oYXR0cmlidXRlcy5kb2xfcmRwLjIpDQojMjE0OCAgICA3DQpgYGANCg0KIyMjIyMjIyMjIyMjIyMjIyMjIyMjIyMjIyMjIyMjIyMjIyMjIyMjIyMjIyMjIyMjIyMjIyMjIyMjIyMjIyMjIyMjIyMNCiMjI0NyZWF0ZSBwaHlsb3NlcS1jbGFzcyBvYmplY3QgdG8gZmlsdGVyICh6T1RVIHRhYmxlIHdpdGggY291bnQgZGF0YSwgbm90IHJlbGF0aXZlIGFidW5kYW5jZSkNCg0KYGBge3J9DQojIyN6T1RVIHRhYmxlDQoNCiNWaWV3KGRhdC5kb2xfdC4xMFssMToxMF0pDQpkaW0oZGF0LmRvbF90LjEwKQ0KIzEyNCAyMTQ5DQoNCiNEZWxldGUgd2F0ZXIgc2FtcGxlcw0KI3Jvd25hbWVzKGRhdC5kb2xfdC4xMCkNCmRhdC5kb2xfdC5uby53YXRlciA8LSBkYXQuZG9sX3QuMTBbLWMoOTA6MTA3LDExMjoxMTQsMTE4OjEyNCksXQ0KZGltKGRhdC5kb2xfdC5uby53YXRlcikNCiM5NiAyMTQ5DQoNCiNWaWV3KGRhdC5kb2xfdC5uby53YXRlclssMjE0MDoyMTQ5XSkNCg0KI0RlbGV0ZSBjb2wgZG9scGhpbg0KZGF0LmRvbF90Lm5vLndhdGVyLjIgPC0gZGF0LmRvbF90Lm5vLndhdGVyWywtMjE0OV0NCg0KI3RyYW5zcG9zZSBkYXQuZG9sX3Qubm8ud2F0ZXIuMiAtPiBkYXQuZG9sX3Qubm8ud2F0ZXIuMi50DQpkYXQuZG9sX3Qubm8ud2F0ZXIuMi50IDwtIGFzLmRhdGEuZnJhbWUodChkYXQuZG9sX3Qubm8ud2F0ZXIuMikpDQojbmFtZXMoZGF0LmRvbF90Lm5vLndhdGVyLjIudCkNCiNyb3duYW1lcyhkYXQuZG9sX3Qubm8ud2F0ZXIuMi50KQ0KDQpkaW0oZGF0LmRvbF90Lm5vLndhdGVyLjIudCkNCiMyMTQ4ICAgIDk2DQoNCiNTb3J0IGF0dHJpYnV0ZXMuZG9sX3JkcC4yIGJ5ICR2YXJpYWJsZQ0KYXR0cmlidXRlcy5kb2xfcmRwLjMgPC0gYXR0cmlidXRlcy5kb2xfcmRwLjJbb3JkZXIoYXR0cmlidXRlcy5kb2xfcmRwLjIkdmFyaWFibGUpLF0gDQojVmlldyhhdHRyaWJ1dGVzLmRvbF9yZHAuMykNCg0KRG9sQmxvd192YXJfbm8ud2F0ZXIuNCA8LSBEb2xCbG93X3Zhcl9uby53YXRlci4zDQoNCnJvd25hbWVzKERvbEJsb3dfdmFyX25vLndhdGVyLjQpIDwtIERvbEJsb3dfdmFyX25vLndhdGVyLjQkWC5TYW1wbGVJRA0KDQpgYGANCiMjIyMjIyMjIyMjIyMjIyMjIyMjIyMjIyMjIyMjIyMjIyMjIyMjIyMjIyMjIyMjIyMjIyMjIyMjIyMjIyMjIyMjIyMjDQojIyMgQ3JlYXRlIHBoeWxvc2VxLWNsYXNzIG9iamVjdCB0byBmaWx0ZXIgdGVjaG5pY2FsIGNvbnRyb2xzDQoNCmBgYHtyfQ0KIyMgVGhlc2UgZmlsZXMgbmVlZCB0byBiZWNvbWUgbWF0cml4IGZpbGVzIHRvIHdvcmsgd2l0aCBwaHlsb3NlcQ0KZG9scGhpbl9vdHVfbm0gPSBhcy5tYXRyaXgoc2FwcGx5KGRhdC5kb2xfdC5uby53YXRlci4yLnQsIGFzLm51bWVyaWMpKQ0KZG9scGhpbl90YXhfY20gPSBhcy5tYXRyaXgoc2FwcGx5KGF0dHJpYnV0ZXMuZG9sX3JkcC4zLCBhcy5jaGFyYWN0ZXIpKQ0KDQojIyBDb252ZXJ0IGZpbGVzIGludG8gcGh5bG9zZXEgb2JqZWN0IHR5cGVzDQp6T1RVID0gb3R1X3RhYmxlKGRvbHBoaW5fb3R1X25tLCB0YXhhX2FyZV9yb3dzID0gVFJVRSkNClRBWCA9IHRheF90YWJsZShkb2xwaGluX3RheF9jbSkNCmRvbHBoaW5kYXRhID0gc2FtcGxlX2RhdGEoZGF0YS5mcmFtZShEb2xCbG93X3Zhcl9uby53YXRlci40LCBzdHJpbmdzQXNGYWN0b3JzID0gRikpDQoNCiMjIG1lcmdlIHRoZSBmaWxlcyB3aXRoIHBoeWxvc2VxDQpkb2xwaGluMSA9IHBoeWxvc2VxKHpPVFUsIFRBWCwgZG9scGhpbmRhdGEpDQoNCiMgcGh5bG9zZXEtY2xhc3MgZXhwZXJpbWVudC1sZXZlbCBvYmplY3QNCg0KIyBwaHlsb3NlcS1jbGFzcyBleHBlcmltZW50LWxldmVsIG9iamVjdA0KIyBvdHVfdGFibGUoKSAgIE9UVSBUYWJsZTogICAgICAgICBbIDIxNDggdGF4YSBhbmQgOTYgc2FtcGxlcyBdDQojIHNhbXBsZV9kYXRhKCkgU2FtcGxlIERhdGE6ICAgICAgIFsgOTYgc2FtcGxlcyBieSAyIHNhbXBsZSB2YXJpYWJsZXMgXQ0KIyB0YXhfdGFibGUoKSAgIFRheG9ub215IFRhYmxlOiAgICBbIDIxNDggdGF4YSBieSA3IHRheG9ub21pYyByYW5rcyBdDQoNCmhlYWQoc2FtcGxlX2RhdGEoZG9scGhpbjEpKQ0KDQojIFB1dCBzYW1wbGVfZGF0YSBpbnRvIGEgZ2dwbG90LWZyaWVuZGx5IGRhdGEuZnJhbWUNCmRmPC0gYXMuZGF0YS5mcmFtZShzYW1wbGVfZGF0YShkb2xwaGluMSkpDQoNCiNDaGVjayBsaWJyYXJ5IHNpemUNCmRmJExpYnJhcnlTaXplIDwtIHNhbXBsZV9zdW1zKGRvbHBoaW4xKQ0KZGY8LSBkZltvcmRlcihkZiRMaWJyYXJ5U2l6ZSksXQ0KZGYkSW5kZXggPC0gc2VxKG5yb3coZGYpKQ0KZ2dwbG90KGRhdGE9ZGYsIGFlcyh4PUluZGV4LCB5PUxpYnJhcnlTaXplLCBjb2xvcj1TYW1wbGVfb3JfQ29udHJvbCkpICsgZ2VvbV9wb2ludCgpICsgZ2VvbV9qaXR0ZXIoKQ0KYGBgDQoNCiMjIyMjIyMjIyMjIyMjIyMjIyMjIyMjIyMjIyMjIyMjIyMjIyMjIyMjIw0KIyMjSWRlbnRpZnkgQ29udGFtaW5hbnRzIHdpdGggZGVjb250YW0tbWV0aG9kIFByZXZhbGVuY2UNCiNkZWNvbnRhbTogaHR0cHM6Ly9iZW5qam5lYi5naXRodWIuaW8vZGVjb250YW0vdmlnbmV0dGVzL2RlY29udGFtX2ludHJvLmh0bWwNCmBgYHtyfQ0Kc2FtcGxlX2RhdGEoZG9scGhpbjEpJGlzLm5lZyA8LSBzYW1wbGVfZGF0YShkb2xwaGluMSkkU2FtcGxlX29yX0NvbnRyb2wgPT0gIkNvbnRyb2wiDQoNCiMjIyMjIyMjIyMjIyMjIyMjIyMNCiMjI3RocmVzaG9sZD0wLjUNCmNvbnRhbWRmLnByZXYwNSAgPC0gaXNDb250YW1pbmFudChkb2xwaGluMSwgbWV0aG9kPSJwcmV2YWxlbmNlIiwgbmVnPSJpcy5uZWciLCB0aHJlc2hvbGQ9MC41KQ0KdGFibGUoY29udGFtZGYucHJldjA1JGNvbnRhbWluYW50KQ0KDQojLS0+IEZhbHNlIGFyZSBub24tY29udGFtaW5hbnRzISEhDQojRkFMU0UgIFRSVUUgDQojIDE5OTEgICAgIDE1Nw0KDQojIE1ha2UgcGh5bG9zZXEgb2JqZWN0IG9mIHByZXNlbmNlLWFic2VuY2UgaW4gbmVnYXRpdmUgY29udHJvbHMgYW5kIHRydWUgc2FtcGxlcw0KcHMucGEgPC0gdHJhbnNmb3JtX3NhbXBsZV9jb3VudHMoZG9scGhpbjEsIGZ1bmN0aW9uKGFidW5kKSAxKihhYnVuZD4wKSkNCg0KcHMucGEubmVnIDwtIHBydW5lX3NhbXBsZXMoc2FtcGxlX2RhdGEocHMucGEpJFNhbXBsZV9vcl9Db250cm9sID09ICJDb250cm9sIiwgcHMucGEpDQoNCnBzLnBhLnBvcyA8LSBwcnVuZV9zYW1wbGVzKHNhbXBsZV9kYXRhKHBzLnBhKSRTYW1wbGVfb3JfQ29udHJvbCA9PSAiVHJ1ZSBTYW1wbGUiLCBwcy5wYSkNCg0KIyBNYWtlIGRhdGEuZnJhbWUgb2YgcHJldmFsZW5jZSBpbiBwb3NpdGl2ZSBhbmQgbmVnYXRpdmUgc2FtcGxlcw0KZGYucGEwNSA8LSBkYXRhLmZyYW1lKHBhLnBvcz10YXhhX3N1bXMocHMucGEucG9zKSwgcGEubmVnPXRheGFfc3Vtcyhwcy5wYS5uZWcpLA0KICAgICAgICAgICAgICAgICAgICBjb250YW1pbmFudD1jb250YW1kZi5wcmV2MDUkY29udGFtaW5hbnQpDQoNCmRlY29udGFtLnBsb3QuMC41IDwtIGdncGxvdChkYXRhPWRmLnBhMDUsIGFlcyh4PXBhLm5lZywgeT1wYS5wb3MsIGNvbG9yPWNvbnRhbWluYW50KSkgKyBnZW9tX3BvaW50KCkgKw0KICB4bGFiKCJQcmV2YWxlbmNlIChOZWdhdGl2ZSBDb250cm9scykiKSArIHlsYWIoIlByZXZhbGVuY2UgKFRydWUgU2FtcGxlcykiKSArIGdlb21faml0dGVyKCkNCg0KZ2dzYXZlKCJkZWNvbnRhbS5wbG90LjA1LmpwZyIsIHBsb3QgPSBkZWNvbnRhbS5wbG90LjAuNSAsIGRldmljZSA9ICdqcGcnLCB3aWR0aCA9IDE2OCwgaGVpZ2h0ID0gMTMwLCB1bml0cyA9ICJtbSIsDQogICAgICAgZHBpID0gMzAwLCBsaW1pdHNpemUgPSBUUlVFKSANCg0KIyMjRnJlcXVlbmN5IHNjb3JlcyBvZiB6T1RVcyANCmhpc3QoY29udGFtZGYucHJldjA1JHApDQpkZWNvbnRhbS5mcmVxLnNjb3JlIDwtIGdncGxvdChjb250YW1kZi5wcmV2MDUsIGFlcyhwKSkgKyBnZW9tX2hpc3RvZ3JhbShiaW53aWR0aCA9IDAuMSkNCg0KZ2dzYXZlKCJkZWNvbnRhbS5mcmVxLnNjb3JlLjA1LmpwZyIsIHBsb3QgPSBkZWNvbnRhbS5mcmVxLnNjb3JlICwgZGV2aWNlID0gJ2pwZycsIHdpZHRoID0gMTY4LCBoZWlnaHQgPSAxMzAsIHVuaXRzID0gIm1tIiwNCiAgICAgICBkcGkgPSAzMDAsIGxpbWl0c2l6ZSA9IFRSVUUpDQoNCmBgYA0KIyMjIyMjIyMjIyMjIyMjIyMjIyMjIyMjIyMjIyMjIyMjIyMjIyMjIyMjIyMjIyMjIyMjIyMjIyMjIyMjIyMjIyMjIyMjIyMjIyMjIyMjIyMjIyMjIyMjIyMjIyMjIyMjIyMjIyMjIyMNCiMjI0FmdGVyIGRldGVybWluaW5nIHRoZSBjb250YW1pbmFudCB6T1RVcywgd2UgZGVsZXRlIHRoZW0gZnJvbSB0aGUgZGF0YSBzZXQgZGF0LmRvbF90Lm5vLndhdGVyLjIudA0KYGBge3J9DQojIyNjb250YW1kZi5wcmV2MDUgY29udGFpbnMgdGhlIGxpc3Qgb2YgY29udGFtaW5hbnQgek9UVXMNCg0KIyMjRXh0cmFjdCBjb2wgJGNvbnRhbWludCBmcm9tIGNvbnRhbWRmLnByZXYwNSBhbmQgY29tYmluZSB3aXRoICNkYXQuZG9sX3Qubm8ud2F0ZXIuMi50DQojIyNkYXQuZG9sX3Qubm8ud2F0ZXIuMi50DQpkaW0oZGF0LmRvbF90Lm5vLndhdGVyLjIudCkNCiMyMTQ4ICAgOTYNCg0Kek9UVV9maWwucHJvZ3Jlc3MgPC0gY29udGFtZGYucHJldjA1JGNvbnRhbWluYW50DQoNCmRhdC5kb2xfdC5uby53YXRlci4yLnQuMiA8LSBjYmluZCh6T1RVX2ZpbC5wcm9ncmVzcyxkYXQuZG9sX3Qubm8ud2F0ZXIuMi50KQ0KZGltKGRhdC5kb2xfdC5uby53YXRlci4yLnQuMikNCiMyMTQ4ICAgOTcNCg0KI1ZpZXcoZGF0LmRvbF90Lm5vLndhdGVyLjIudC4yWzE6MTAsXSkNCg0KIyMjT25seSBrZWVwIHpPVFVTID09IEZhbHNlIChub24tY29udGFtaW5hbnRzKQ0KDQpkYXQuZG9sX3Qubm8ud2F0ZXIuMi50LjMgPC0gZGF0LmRvbF90Lm5vLndhdGVyLjIudC4yDQoNCmRhdC5kb2xfdC5uby53YXRlci4yLnQuMyR2YXJpYWJsZSA8LSByb3duYW1lcyhkYXQuZG9sX3Qubm8ud2F0ZXIuMi50LjMpDQpkaW0oZGF0LmRvbF90Lm5vLndhdGVyLjIudC4zKQ0KIzIxNDggICA5OA0KDQpkYXQuZG9sX2ZpbCA8LSBkYXQuZG9sX3Qubm8ud2F0ZXIuMi50LjMgJT4lIGZpbHRlciAoIXpPVFVfZmlsLnByb2dyZXNzID09ICdUUlVFJykNCmRpbShkYXQuZG9sX2ZpbCkNCiMxOTkxICAgOTgNCg0KI0RlbGV0ZSBjb2wgJHpPVFVfZmlsLnByb2dyZXNzLCBicmluZyBjb2wgJHZhcmlhYmxlIHRvIHRoZSBmcm9udA0KZGF0LmRvbF9maWwuMiA8LSBkYXQuZG9sX2ZpbFssYyg5OCwgMjo5NyldDQojVmlldyhkYXQuZG9sX2ZpbC4yWzE6MTAsXSkNCg0KI1RyYW5zcG9zZSBvbmNlIG1vcmUNCmRhdC5kb2xfZmlsLjMgPC0gZGF0LmRvbF9maWwuMg0Kcm93bmFtZXMoZGF0LmRvbF9maWwuMykgPC0gZGF0LmRvbF9maWwuMyR2YXJpYWJsZQ0KZGF0LmRvbF9maWwuNCA8LSBkYXQuZG9sX2ZpbC4zWywtMV0NCg0KZGF0LmRvbF9maWwudCA8LSBhcy5kYXRhLmZyYW1lKHQoZGF0LmRvbF9maWwuNCkpDQpkaW0oZGF0LmRvbF9maWwudCkNCiM5NiAxOTkxDQojVmlldyhkYXQuZG9sX2ZpbC50WywxOjEwXSkNCg0KI0JyaW5nIGluIG9yZGVyOiBEb2xwaGluIHNhbXBsZXMgb24gdG9wLCBjb250cm9scyBhdCBib3R0b20NCmRhdC5kb2xfZmlsLnQuMiA8LSBkYXQuZG9sX2ZpbC50W2MoMTo4MCw4Nzo4OSw4MTo4Niw5MDo5NiksXQ0KI3Jvd25hbWVzKGRhdC5kb2xfZmlsLnQuMikNCg0KI0RlbGV0ZSBSb3dzIHdpdGggY29udHJvbHM6DQpkYXQuZG9sX2ZpbC50LjMgPC0gZGF0LmRvbF9maWwudC4yWzE6ODMsXQ0KI3Jvd25hbWVzKGRhdC5kb2xfZmlsLnQuMykNCmRpbShkYXQuZG9sX2ZpbC50LjMpDQojODMgMTk5MQ0KYGBgDQojIyMjIyMjIyMjIyMjIyMjIyMjIyMjIyMjIyMjIyMjIyMNCiMjI0RlbGV0ZSBhbGwgdGVjaG5pY2FsIGNvbnRhbWluYW50IHpPVFVzIGluIGF0dHJpYnV0ZXMuZG9sX3JkcC4zDQoNCmBgYHtyfQ0KIyMjYXR0cmlidXRlcy5kb2xfcmRwLjMNCg0KIyMjRXh0cmFjdCBjb2wgJGNvbnRhbWludCBmcm9tIGNvbnRhbWRmLnByZXYwNSBhbmQgY29tYmluZSB3aXRoICNkYXQuZG9sX3Qubm8ud2F0ZXIuMi50IGFuZCBhdHRyaWJ1dGVzLmRvbF9yZHAuMw0KDQpkaW0oYXR0cmlidXRlcy5kb2xfcmRwLjMpDQojMjE0OCAgIDcNCiNWaWV3KGF0dHJpYnV0ZXMuZG9sX3JkcC4zKQ0KDQphdHRyaWJ1dGVzLmRvbF9yZHAuNCA8LSBjYmluZCh6T1RVX2ZpbC5wcm9ncmVzcyxhdHRyaWJ1dGVzLmRvbF9yZHAuMykNCmRpbShhdHRyaWJ1dGVzLmRvbF9yZHAuNCkNCiMyMTQ4ICAgOA0KDQojVmlldyhhdHRyaWJ1dGVzLmRvbF9yZHAuNCkNCg0KIyMjT25seSBrZWVwIHpPVFVTID09IEZhbHNlIChub24tY29udGFtaW5hbnRzKQ0KYXR0cmlidXRlcy5kb2xfcmRwLjUgPC0gYXR0cmlidXRlcy5kb2xfcmRwLjQNCg0KYXR0cmlidXRlcy5kb2xfcmRwX2ZpbCA8LSBhdHRyaWJ1dGVzLmRvbF9yZHAuNSAlPiUgZmlsdGVyICghek9UVV9maWwucHJvZ3Jlc3MgPT0gJ1RSVUUnKQ0KZGltKGF0dHJpYnV0ZXMuZG9sX3JkcF9maWwpDQojMTk5MSAgIDgNCg0KI0RlbGV0ZSBjb2wgJHpPVFVfZmlsLnByb2dyZXNzDQphdHRyaWJ1dGVzLmRvbF9yZHBfZmlsLjIgPC0gYXR0cmlidXRlcy5kb2xfcmRwX2ZpbFssLTFdDQojVmlldyhhdHRyaWJ1dGVzLmRvbF9yZHBfZmlsLjIpDQpgYGANCg0KIyMjIyMjIyMjIyMjIyMjIyMjIyMjIyMjIyMjIyMjIyMjDQojIyNDcmVhdGUgdGFibGUgd2l0aCB0ZWNoIGNvbnRhbWluYW50cyB6T1RVcyB0aGF0IHdlcmUgZGVsZXRlZA0KYGBge3J9DQojIyNPbmx5IGtlZXAgek9UVVMgPT0gVFJVRSAoY29udGFtaW5hbnRzKQ0KYXR0cmlidXRlcy5kb2xfcmRwX2NvbnRzIDwtIGF0dHJpYnV0ZXMuZG9sX3JkcC41ICU+JSBmaWx0ZXIgKHpPVFVfZmlsLnByb2dyZXNzID09ICdUUlVFJykNCmRpbShhdHRyaWJ1dGVzLmRvbF9yZHBfY29udHMpDQojMTU3ICAgOA0KDQojIyNEZWxldGUgY29sIDEgJiA4DQphdHRyaWJ1dGVzLmRvbF9yZHBfY29udHMuMiA8LSBhdHRyaWJ1dGVzLmRvbF9yZHBfY29udHNbLC1jKDEsOCldDQoNCiMjI1NhdmUgYXMgY3N2LWZpbGUgKGZvciBzdXBwbGVtZW50cykNCndyaXRlLmNzdihmaWxlID0gJ2F0dHJpYnV0ZXMuZG9sX3JkcF9jb250cy4yLmNzdicsIGF0dHJpYnV0ZXMuZG9sX3JkcF9jb250cy4yKQ0KYGBgDQojIyMjIyMjIyMjIyMjIyMjIyMjIyMjIyMjIyMjIyMjIyMNCiMjI0RlbGV0ZSBhbGwgY29udHJvbCBzYW1wbGVzIGluIERvbEJsb3dfdmFyX25vLndhdGVyLjQNCmBgYHtyfQ0KI1ZpZXcoRG9sQmxvd192YXIuMikNCkRvbEJsb3dfdmFyLmZpbCA8LSBEb2xCbG93X3Zhci4yICU+JSBmaWx0ZXIgKCFTcGVjaWVzID09ICd0ZWNoLmNvbnRyb2wnKQ0KZGltKERvbEJsb3dfdmFyLmZpbCkNCiMxMTEgICAzDQoNCiMjI0RlbGV0ZSBjb2wgMSAkU2FtcGxlDQpEb2xCbG93X3Zhci5maWwuMiA8LSBEb2xCbG93X3Zhci5maWxbLC0xXQ0KYGBgDQojIyMjIyMjIyMjIyMjIyMjIyMjIyMjIyMjIyMjIyMjIyMNCiMjI0NvbWJpbmUgek9UVXMgdGFibGVzIG9mIGRvbHBoaW4gc2FtcGxlcyBhbmQgd2F0ZXIgc2FtcGxlcyBhZ2Fpbg0KYGBge3J9DQojIyN6T1RVLXRhYmxlIGluY2x1ZGluZyB3YXRlciwgY29udHJvbCBhbmQgZG9scGhpbiBzYW1wbGVzDQoNCmRpbShkYXQuZG9sX3QuMTApDQojMTI0IDIxNDkNCg0KI1ZpZXcoZGF0LmRvbF90LjEwWywxOjEwXSkNCg0KIyMjVHVybiByb3duYW1lcyBpbnRvIGNvbA0KZGF0LmRvbF90LjE1IDwtIGRhdC5kb2xfdC4xMA0KZGF0LmRvbF90LjE1JERvbHBoaW4gPC0gcm93bmFtZXMoZGF0LmRvbF90LjE1KQ0KDQojIyNNZXJnZSBEb2xCbG93X3Zhci5maWwuMiBhbmQgZGF0LmRvbF90LjEwDQpkYXQuZG9sX3QuMTYgPC0gbGVmdF9qb2luKERvbEJsb3dfdmFyLmZpbC4yLCBkYXQuZG9sX3QuMTUpDQojVmlldyhkYXQuZG9sX3QuMTYpDQoNCiMjIyMjIyMjIyMjRGVsZXRlIGFsbCBjb250YW1pbnQgek9UVXMgKHpPVFVfZmlsLnByb2dyZXNzKQ0KDQojIyNEZWxldGUgY29sIDEgb2YgZGF0LmRvbF90LjE2DQpkYXQuZG9sX3QuMTcgPC0gZGF0LmRvbF90LjE2WywtMV0NCg0KI1R1cm4gY29sICREb2xwaGluIGludG8gcm93bmFtZXMNCmRhdC5kb2xfdC4xOCA8LSBkYXQuZG9sX3QuMTcNCnJvd25hbWVzKGRhdC5kb2xfdC4xOCkgPC0gZGF0LmRvbF90LjE4JERvbHBoaW4NCmRhdC5kb2xfdC4xOSA8LSBkYXQuZG9sX3QuMTggWywgLTFdDQojVmlldyhkYXQuZG9sX3QuMTkpDQoNCiMjI1RyYW5zcG9zZSBkYXQuZG9sX3QuMTkNCmRhdC5kb2xfdC4xOS50IDwtIGFzLmRhdGEuZnJhbWUodChkYXQuZG9sX3QuMTkpKQ0KDQojIyNUdXJuIHJvd25hbWVzIGJhY2sgaW50byBjb2wNCmRpbShkYXQuZG9sX3QuMTkudCkNCiMyMTQ4ICAxMTENCmRhdC5kb2xfdC4xOS50LjIgPC0gZGF0LmRvbF90LjE5LnQNCmRhdC5kb2xfdC4xOS50LjIkdmFyaWFibGUgPC0gcm93bmFtZXMoZGF0LmRvbF90LjE5LnQuMikNCmRpbShkYXQuZG9sX3QuMTkudC4yKQ0KIzIxNDggIDExMg0KDQojIyNCcmluZyBjb2wgJHZhcmlhYmxlIHRvIHRoZSBmcm9udA0KZGF0LmRvbF90LjE5LnQuMyA8LSBkYXQuZG9sX3QuMTkudC4yIFssYygxMTIsIDE6MTExKV0NCg0KIyMjRGVsZXRlIGFsbCBjb250YW1pbnQgek9UVXMgKHpPVFVfZmlsLnByb2dyZXNzKSAtICMjI09ubHkga2VlcCB6T1RVUyA9PSBGYWxzZSAobm9uLWNvbnRhbWluYW50cykNCmRhdC5kb2xfdC4xOS50LjQgPC0gY2JpbmQoek9UVV9maWwucHJvZ3Jlc3MsZGF0LmRvbF90LjE5LnQuMykNCg0KZGF0LmRvbF90LjE5LnQuZmlsIDwtIGRhdC5kb2xfdC4xOS50LjQgJT4lIGZpbHRlciAoek9UVV9maWwucHJvZ3Jlc3MgPT0gRkFMU0UpIA0KZGltKGRhdC5kb2xfdC4xOS50LmZpbCkNCiMxOTkxIDExMw0KDQojIyNGdXJ0aGVyIHByZXBhcmUgZGF0LmRvbF90LjE5LnQuZmlsIGZvciBkZWNvbnRhbQ0KDQojIyNUdXJuICR2YXJpYWJsZSBpbnRvIHJvd25hbWVzDQpyb3duYW1lcyhkYXQuZG9sX3QuMTkudC5maWwpIDwtIGRhdC5kb2xfdC4xOS50LmZpbCR2YXJpYWJsZQ0KDQojIyNEZWxldGUgY29sIDEgJiAyDQpkYXQuZG9sX3QuMTkudC5maWwuMiA8LSBkYXQuZG9sX3QuMTkudC5maWxbLC1jKDEsMildDQoNCiNUcmFuc3Bvc2UNCmRhdC5kb2xfMi5maWwudCA8LSBhcy5kYXRhLmZyYW1lKHQoZGF0LmRvbF90LjE5LnQuZmlsLjIpKQ0KI3Jvd25hbWVzKGRhdC5kb2xfMi5maWwudCkNCmBgYA0KDQojIyMjIyMjIyMjIyMjIyMjIyMjIyMjIyMjIyMjIyMjIyMjIyMjIyMjIyMjIyMjIyMjIyMjDQojIyMjIyMjIyNXaGF0J3MgdGhlIGF2ZXJhZ2UgbnVtYmVyIG9mIHJlYWRzIHBlciBzYW1wbGVzPw0KYGBge3J9DQpkaW0oZGF0LmRvbF8yLmZpbC50KQ0KIzExMSAxOTkxDQoNCmRhdC5kb2xfMi5maWwudF8xMCA9IG11dGF0ZSAoZGF0LmRvbF8yLmZpbC50LCBzdW1fb2Zfcm93cz1yb3dTdW1zKGRhdC5kb2xfMi5maWwudCkpDQoNCiNWaWV3KGRhdC5kb2xfMi5maWwudF8xMCkNCm1lYW4oZGF0LmRvbF8yLmZpbC50XzEwJHN1bV9vZl9yb3dzKQ0Kc2QoZGF0LmRvbF8yLmZpbC50XzEwJHN1bV9vZl9yb3dzKQ0KYGBgDQoNCiMjIyMjIyMjIyMjIyMjIyMjIyMjIyMjIyMjIyMjIyMjIyMjIyMjIyMjIyMNCiMjIyMjIyMjIyMjI0JldGEgRGl2ZXJzaXR5IG9mIGRvbHBoaW4gc2FtcGxlcyB2cyBwb29sIHNhbXBsZXMNCmBgYHtyfQ0KIyMjbk1EUyBwbG90DQoNCiMjI1VzZSBkYXQuZG9sXzIuZmlsLnQgYW5kIERvbEJsb3dfdmFyLmZpbC4yDQojVmlldyhkYXQuZG9sXzIuZmlsLnQpDQojVmlldyhEb2xCbG93X3Zhci5maWwuMikNCg0KIyMjVHVybiBkYXQuZG9sXzIuZmlsLnQgaW50byByZWwgYWJ1bmQNCmRhdC5kb2xfdC50ZWNoLmZpbC5yZWwuYWJ1bmQgPSBkYXQuZG9sXzIuZmlsLnQvcm93U3VtcyhkYXQuZG9sXzIuZmlsLnQpDQoNCiNDb252ZXJ0IHRvIGxvZw0KZGF0LmRvbF90LnRlY2guZmlsLnJlbC5hYnVuZC5sb2cgPSBsb2coZGF0LmRvbF90LnRlY2guZmlsLnJlbC5hYnVuZCArMSkNCg0KI0JyYXktQ3VydGlzIGRpc3NpbWlsYXJpdHkgbWF0cml4IHdpdGggbG9nDQpkYXQuZG9sX3QudGVjaC5maWwucmVsLmFidW5kLmxvZy5iYyA9IHZlZ2Rpc3QoZGF0LmRvbF90LnRlY2guZmlsLnJlbC5hYnVuZC5sb2csIG1ldGhvZCA9ICJicmF5IikNCg0KIyMjI25NRFMNCiMgbm1kcyBwbG90IHdpdGggZG90cyBvZiAyIGNvbG91cnMsIChzdHJlc3Mgc2hvdWxkIGJlIDwwLjIpDQpkYXQuZG9sX3QudGVjaC5maWwucmVsLmFidW5kLmxvZy5oY2x1cy5tZHMgPSBtZXRhTURTKGRhdC5kb2xfdC50ZWNoLmZpbC5yZWwuYWJ1bmQubG9nLmJjLCBhdXRvdHJhbnNmb3JtID0gRiwgdHJhY2UgPSBGLCB0cnltYXg9NTApDQpkYXQuZG9sX3QudGVjaC5maWwucmVsLmFidW5kLmxvZy5oY2x1cy5tZHMgIyBzdHJlc3MgPSAwLjE0Njg5MTkNCg0KIyMjQ3JlYXRlIHBsb3RkYXRhDQpwbG90RGF0YS5kb2xwaGluLnRlY2guZmlsID0gZGF0YS5mcmFtZShkYXQuZG9sX3QudGVjaC5maWwucmVsLmFidW5kLmxvZy5oY2x1cy5tZHMkcG9pbnRzLCBEb2xCbG93X3Zhci5maWwuMiRTcGVjaWVzKQ0KbmFtZXMoKSA9IGMoIngiLCJ5IiwiU3BlY2llcyIpDQojcGxvdERhdGEuZG9scGhpbi50ZWNoLmZpbC5yZW0gPSBwbG90RGF0YS5kb2xwaGluLnRlY2guZmlsW3Bsb3REYXRhLmRvbHBoaW4udGVjaC5maWwkeCA8IDAuMixdDQoNCm5NRFMudGVjaC5maWwgPSBnZ3Bsb3QocGxvdERhdGEuZG9scGhpbi50ZWNoLmZpbCwgYWVzKHgseSxjb2xvdXI9U3BlY2llcywgc2hhcGU9U3BlY2llcykpICsgZ2VvbV9wb2ludCgpICsNCiAgdGhlbWVfYncoKSArDQogIHRoZW1lKHBsb3QudGl0bGUgPSBlbGVtZW50X3RleHQoc2l6ZSA9IDE0LCBmYW1pbHkgPSAiVGFob21hIiwgZmFjZSA9ICJib2xkIiksDQogICAgICAgIHRleHQgPSBlbGVtZW50X3RleHQoc2l6ZSA9IDEyLCBmYW1pbHkgPSAiVGFob21hIiksDQogICAgICAgIGF4aXMudGl0bGUgPSBlbGVtZW50X3RleHQoZmFjZT0iYm9sZCIpLA0KICAgICAgICBheGlzLnRleHQueD1lbGVtZW50X3RleHQoc2l6ZSA9IDExKSkgKw0KICAgICAgICBzY2FsZV9maWxsX2JyZXdlcihwYWxldHRlID0gIkFjY2VudCIpICsgZ2VvbV90ZXh0KGxhYmVsID0gcm93bmFtZXMoZGF0LmRvbF90LnRlY2guZmlsLnJlbC5hYnVuZC5sb2cpKQ0KDQpnZ3NhdmUoIm5NRFMudGVjaC5maWwubmFtZXMuanBnIiwgcGxvdCA9IG5NRFMudGVjaC5maWwsIGRldmljZSA9ICdqcGcnLCB3aWR0aCA9IDE2OCwgaGVpZ2h0ID0gMTAwLCB1bml0cyA9ICJtbSIsDQogICAgICAgZHBpID0gMzAwLCBsaW1pdHNpemUgPSBUUlVFKQ0KDQojIyNXaXRob3V0IG5hbWVzDQpuTURTLnRlY2guZmlsLm5vTmFtZXMgPSBnZ3Bsb3QocGxvdERhdGEuZG9scGhpbi50ZWNoLmZpbCwgYWVzKHgseSxjb2xvdXI9U3BlY2llcywgc2hhcGU9U3BlY2llcykpICsgZ2VvbV9wb2ludCgpICsNCiAgdGhlbWVfYncoKSArDQogIHRoZW1lKHBsb3QudGl0bGUgPSBlbGVtZW50X3RleHQoc2l6ZSA9IDE0LCBmYW1pbHkgPSAiVGFob21hIiwgZmFjZSA9ICJib2xkIiksDQogICAgICAgIHRleHQgPSBlbGVtZW50X3RleHQoc2l6ZSA9IDEyLCBmYW1pbHkgPSAiVGFob21hIiksDQogICAgICAgIGF4aXMudGl0bGUgPSBlbGVtZW50X3RleHQoZmFjZT0iYm9sZCIpLA0KICAgICAgICBheGlzLnRleHQueD1lbGVtZW50X3RleHQoc2l6ZSA9IDExKSkgKw0KICBzY2FsZV9maWxsX2JyZXdlcihwYWxldHRlID0gIkFjY2VudCIpICsNCiAgeGxhYigibk1EUyAxIikgKyB5bGFiKCJuTURTIDIiKQ0KDQpnZ3NhdmUoIm5NRFMudGVjaC5maWwubm9OYW1lcy5qcGciLCBwbG90ID0gbk1EUy50ZWNoLmZpbC5ub05hbWVzLCBkZXZpY2UgPSAnanBnJywgd2lkdGggPSAxNjgsIGhlaWdodCA9IDEwMCwgdW5pdHMgPSAibW0iLA0KICAgICAgIGRwaSA9IDMwMCwgbGltaXRzaXplID0gVFJVRSkNCmBgYA0KDQojIyMjIyMjIyMjIyMjIyMjIyMjIyMjIyMjIyMjIyMjIyMjIyMjIyMjIyMjIyMjIyMjIyMNCiMjIyMjIyMjIyMjIyMjI1VuaWZyYWMNCmBgYHtyfQ0KDQpgYGANCg0KIyMjIyMjIyMjIyMjIyMjIyMjIyMjIyMjIyMjIyMjIyMjIyMjIyMjIyMjIyMjIyMjIyMNCiMjIyMjIyNSYXJlZmFjdGlvbiBjdXJ2ZXMgYW5kIEdvb2QncyBjb3ZlcmFnZSBvZiBkb2xwaGlucyBhbmQgcG9vbCB3YXRlciBzYW1wbGVzIChiZWZvcmUgZmlsdGVyaW5nIG91dCBwb29sIHdhdGVyIHpPVFVzKQ0KIyMjUHJlcGFyZSBkYXRhc2V0cw0KYGBge3J9DQojZGF0LmRvbF8yLmZpbC50LCBEb2xCbG93X3Zhci5maWwuMiwgYXR0cmlidXRlcy5kb2xfcmRwX2ZpbC4yDQojVmlldyhkYXQuZG9sXzIuZmlsLnQpDQojVmlldyhEb2xCbG93X3Zhci5maWwuMikNCiNWaWV3KGF0dHJpYnV0ZXMuZG9sX3JkcF9maWwuMikNCg0KIyMjR2l2ZSBhdHRyaWJ1dGVzLmRvbF9yZHBfZmlsLjIgcm93bmFtZXMgYW5kIGRlbGV0ZSBsYXN0IGNvbHVtbg0KYXR0cmlidXRlcy5kb2xfcmRwX2ZpbC4zIDwtIGF0dHJpYnV0ZXMuZG9sX3JkcF9maWwuMiBbLCAtN10NCnJvd25hbWVzKGF0dHJpYnV0ZXMuZG9sX3JkcF9maWwuMykgPC0gYXR0cmlidXRlcy5kb2xfcmRwX2ZpbC4zJHZhcmlhYmxlDQoNCnJvd25hbWVzKERvbEJsb3dfdmFyLmZpbC4yKSA9IERvbEJsb3dfdmFyLmZpbC4yJFdoYWxlDQoNCiNUcmFuc3Bvc2UgZGF0LmRvbF8yLmZpbC50DQpkYXQuZG9sXzIuZmlsLnRfdCA9IGFzLmRhdGEuZnJhbWUodChkYXQuZG9sXzIuZmlsLnQpKQ0KYGBgDQoNCiMjIyMjIyNSYXJlZmFjdGlvbiBjdXJ2ZXM6IGZ1bmN0aW9uDQpgYGB7cn0NCnJlcXVpcmUocGFyYWxsZWwpDQpnZ3JhcmUgPC0gZnVuY3Rpb24ocGh5c2VxLCBzdGVwID0gMTAsIGxhYmVsID0gTlVMTCwgY29sb3IgPSBOVUxMLCBwbG90ID0gVFJVRSwgcGFyYWxsZWwgPSBGQUxTRSwgc2UgPSBUUlVFKSB7DQogIHggPC0gYXMob3R1X3RhYmxlKHBoeXNlcSksICJtYXRyaXgiKQ0KICBpZiAodGF4YV9hcmVfcm93cyhwaHlzZXEpKSB7IHggPC0gdCh4KSB9DQogIA0KICAjIyBUaGlzIHNjcmlwdCBpcyBhZGFwdGVkIGZyb20gdmVnYW4gYHJhcmVjdXJ2ZWAgZnVuY3Rpb24NCiAgdG90IDwtIHJvd1N1bXMoeCkNCiAgUyA8LSByb3dTdW1zKHggPiAwKQ0KICBuciA8LSBucm93KHgpDQogIA0KICByYXJlZnVuIDwtIGZ1bmN0aW9uKGkpIHsNCiAgICBjYXQocGFzdGUoInJhcmVmeWluZyBzYW1wbGUiLCByb3duYW1lcyh4KVtpXSksIHNlcCA9ICJcbiIpDQogICAgbiA8LSBzZXEoMSwgdG90W2ldLCBieSA9IHN0ZXApDQogICAgaWYgKG5bbGVuZ3RoKG4pXSAhPSB0b3RbaV0pIHsNCiAgICAgIG4gPC0gYyhuLCB0b3RbaV0pDQogICAgfQ0KICAgIHkgPC0gcmFyZWZ5KHhbaSwgLGRyb3AgPSBGQUxTRV0sIG4sIHNlID0gc2UpDQogICAgaWYgKG5yb3coeSkgIT0gMSkgew0KICAgICAgcm93bmFtZXMoeSkgPC0gYygiLlMiLCAiLnNlIikNCiAgICAgIHJldHVybihkYXRhLmZyYW1lKHQoeSksIFNpemUgPSBuLCBTYW1wbGUgPSByb3duYW1lcyh4KVtpXSkpDQogICAgfSBlbHNlIHsNCiAgICAgIHJldHVybihkYXRhLmZyYW1lKC5TID0geVsxLCBdLCBTaXplID0gbiwgU2FtcGxlID0gcm93bmFtZXMoeClbaV0pKQ0KICAgIH0NCiAgfQ0KICBpZiAocGFyYWxsZWwpIHsNCiAgICBvdXQgPC0gbWNsYXBwbHkoc2VxX2xlbihuciksIHJhcmVmdW4sIG1jLnByZXNjaGVkdWxlID0gRkFMU0UpDQogIH0gZWxzZSB7DQogICAgb3V0IDwtIGxhcHBseShzZXFfbGVuKG5yKSwgcmFyZWZ1bikNCiAgfQ0KICBkZiA8LSBkby5jYWxsKHJiaW5kLCBvdXQpDQogIA0KICAjIyBHZXQgc2FtcGxlIGRhdGEgDQogIGlmICghaXMubnVsbChzYW1wbGVfZGF0YShwaHlzZXEsIEZBTFNFKSkpIHsNCiAgICBzZGYgPC0gYXMoc2FtcGxlX2RhdGEocGh5c2VxKSwgImRhdGEuZnJhbWUiKQ0KICAgIHNkZiRTYW1wbGUgPC0gcm93bmFtZXMoc2RmKQ0KICAgIGRhdGEgPC0gbWVyZ2UoZGYsIHNkZiwgYnkgPSAiU2FtcGxlIikNCiAgICBsYWJlbHMgPC0gZGF0YS5mcmFtZSh4ID0gdG90LCB5ID0gUywgU2FtcGxlID0gcm93bmFtZXMoeCkpDQogICAgbGFiZWxzIDwtIG1lcmdlKGxhYmVscywgc2RmLCBieSA9ICJTYW1wbGUiKQ0KICB9DQogIA0KICAjIyBBZGQsIGFueSBjdXN0b20tc3VwcGxpZWQgcGxvdC1tYXBwZWQgdmFyaWFibGVzDQogIGlmKCBsZW5ndGgoY29sb3IpID4gMSApew0KICAgIGRhdGEkY29sb3IgPC0gY29sb3INCiAgICBuYW1lcyhkYXRhKVtuYW1lcyhkYXRhKT09ImNvbG9yIl0gPC0gZGVwYXJzZShzdWJzdGl0dXRlKGNvbG9yKSkNCiAgICBjb2xvciA8LSBkZXBhcnNlKHN1YnN0aXR1dGUoY29sb3IpKQ0KICB9DQogIGlmKCBsZW5ndGgobGFiZWwpID4gMSApew0KICAgIGxhYmVscyRsYWJlbCA8LSBsYWJlbA0KICAgIG5hbWVzKGxhYmVscylbbmFtZXMobGFiZWxzKT09ImxhYmVsIl0gPC0gZGVwYXJzZShzdWJzdGl0dXRlKGxhYmVsKSkNCiAgICBsYWJlbCA8LSBkZXBhcnNlKHN1YnN0aXR1dGUobGFiZWwpKQ0KICB9DQogIA0KICBwIDwtIGdncGxvdChkYXRhID0gZGF0YSwgYWVzX3N0cmluZyh4ID0gIlNpemUiLCB5ID0gIi5TIiwgZ3JvdXAgPSAiU2FtcGxlIiwgY29sb3IgPSBjb2xvcikpICsNCiAgICB0aGVtZV9idygpICsNCiAgICB0aGVtZShwbG90LnRpdGxlID0gZWxlbWVudF90ZXh0KHNpemUgPSAxNCwgZmFtaWx5ID0gIlRhaG9tYSIsIGZhY2UgPSAiYm9sZCIpLA0KICAgICAgICAgIHRleHQgPSBlbGVtZW50X3RleHQoc2l6ZSA9IDEyLCBmYW1pbHkgPSAiVGFob21hIiksDQogICAgICAgICAgYXhpcy50aXRsZSA9IGVsZW1lbnRfdGV4dChmYWNlPSJib2xkIiksDQogICAgICAgICAgYXhpcy50ZXh0Lng9ZWxlbWVudF90ZXh0KHNpemUgPSAxMSkpICsNCiAgICBzY2FsZV9maWxsX2JyZXdlcihwYWxldHRlID0gIkFjY2VudCIpICsNCiAgICB0aGVtZShheGlzLnRleHQueD1lbGVtZW50X3RleHQoYW5nbGU9OTApKQ0KICANCiAgcCA8LSBwICsgbGFicyh4ID0gIlNhbXBsZSBTaXplIiwgeSA9ICJTcGVjaWVzIFJpY2huZXNzIikNCiAgaWYgKCFpcy5udWxsKGxhYmVsKSkgew0KICAgIHAgPC0gcCArIGdlb21fdGV4dChkYXRhID0gbGFiZWxzLCBhZXNfc3RyaW5nKHggPSAieCIsIHkgPSAieSIsIGxhYmVsID0gbGFiZWwsIGNvbG9yID0gY29sb3IpLA0KICAgICAgICAgICAgICAgICAgICAgICBzaXplID0gNCwgaGp1c3QgPSAwKQ0KICB9DQogIHAgPC0gcCArIGdlb21fbGluZSgpDQogIGlmIChzZSkgeyAjIyBhZGQgc3RhbmRhcmQgZXJyb3IgaWYgYXZhaWxhYmxlDQogICAgcCA8LSBwICsgZ2VvbV9yaWJib24oYWVzX3N0cmluZyh5bWluID0gIi5TIC0gLnNlIiwgeW1heCA9ICIuUyArIC5zZSIsIGNvbG9yID0gTlVMTCwgZmlsbCA9IGNvbG9yKSwgYWxwaGEgPSAwLjIpDQogIH0NCiAgaWYgKHBsb3QpIHsNCiAgICBwbG90KHApDQogIH0NCiAgaW52aXNpYmxlKHApDQp9DQoNCnBoeWxvZGl2IDwtIGZ1bmN0aW9uKHBoeXNlcSkgew0KICAjIyBBcmdzOg0KICAjIyAtIHBoeXNlcTogcGh5bG9zZXEgY2xhc3Mgb2JqZWN0LCBmcm9tIHdoaWNoIHBoeWxvZ2VueSBhbmQgYWJ1bmRhbmNlIGRhdGEgYXJlIGV4dHJhY3RlZA0KICB4IDwtIGFzKG90dV90YWJsZShwaHlzZXEpLCAibWF0cml4IikNCiAgaWYgKHRheGFfYXJlX3Jvd3MocGh5c2VxKSkgeyB4IDwtIHQoeCkgfQ0KICBwaHkgPC0gcGh5X3RyZWUocGh5c2VxKQ0KICANCiAgIyMgQ29uc3RydWN0IGluY2lkZW5jZSBtYXRyaXggb2YgdGhlIHRyZWUNCiAgaW5jaWRlbmNlIDwtIGluY2lkZW5jZU1hdHJpeChwaHkpDQogIA0KICAjIyBPcmRlciBpbmNpZGVuY2UgbWF0cml4IGFjY29yZGluZyB0byBjb21tdW5pdHkgdGFibGVzDQogIGluY2lkZW5jZSA8LSBpbmNpZGVuY2VbY29sbmFtZXMoeCksIF0NCiAgDQogICMjIENyZWF0ZSBjb21tdW5pdHkgcGh5bG9nZW55IG1hdHJpeCBieSBtdWx0aXBseWluZyAoY29tbXVuaXR5IHggZWRnZSBtYXRyaXgpDQogICMjIHdoZXJlIGNwbV97aWp9IGdpdmVzIHRoZSBhYnVuZGFuY2Ugb2YgT1RVcyBvcmlnaW5hdGluZyBmcm9tIGJyYW5jaCBqIGluIGNvbW11bml0eSBpLiANCiAgY3BtIDwtIHggJSolIGluY2lkZW5jZQ0KICAjIyBDb252ZXJ0IHRvIGluY2lkZW5jZSBtYXRyaXggKDAvMSkgYW5kIG11bHRpcGx5IGJ5IGVkZ2UgbGVuZ3RoIHRvIG9idGFpbiBQRCBwZXIgY29tbXVuaXR5Lg0KICBjcG1bY3BtID4gMF0gPC0gMQ0KICBwZCA8LSAgY3BtICUqJSBwaHkkZWRnZS5sZW5ndGgNCiAgDQogICMjIEFkZCBzYW1wbGUgZGF0YSBpbmZvcm1hdGlvbg0KICBpZiAoIWlzLm51bGwoc2FtcGxlX2RhdGEocGh5c2VxLCBGQUxTRSkpKSB7DQogICAgc2RmIDwtIGFzKHNhbXBsZV9kYXRhKHBoeXNlcSksICJkYXRhLmZyYW1lIikNCiAgICBzZGYkcGQgPC0gYXMudmVjdG9yKHBkKQ0KICAgIHBkIDwtIHNkZg0KICB9DQogIA0KICByZXR1cm4gKHBkKQ0KfQ0KYGBgDQojIyMjIyMjIyMjIyMjIyMjIyMjIyMjIyMjIyMjIyMjIyMjIyMjIyMjIyMjIyMjIyMjIyMjDQojIyMjIyMjI0NyZWF0ZSByYXJlZmFjdGlvbiBjdXJ2ZXMgc2VwYXJhdGVseSBieSBkb2xwaGluIGFuZCBwb29sDQoNCiMjIyMjIyMjIyMjIyMjIyMNCiMjI0NvZW4NCmBgYHtyfQ0KIyMjQ29lbg0KDQojZGF0LmRvbF8yLmZpbC50X3QsIERvbEJsb3dfdmFyLmZpbC4yLCBhdHRyaWJ1dGVzLmRvbF9yZHBfZmlsLjMNCiNuYW1lcyhkYXQuZG9sXzIuZmlsLnRfdCkNCiNEb2xCbG93X3Zhci5maWwuMiREb2xwaGluDQoNCmRhdC5kb2xfMi5maWwudF90LkNvZW4gPSBkYXQuZG9sXzIuZmlsLnRfdFssYygxNjoyMSldDQpEb2xCbG93X3Zhci5maWwuQ29lbiA9IERvbEJsb3dfdmFyLmZpbC4yW2MoMTY6MjEpLF0NCnJvd25hbWVzKERvbEJsb3dfdmFyLmZpbC5Db2VuKSA9IERvbEJsb3dfdmFyLmZpbC5Db2VuJERvbHBoaW4NCg0KZGF0LmRvbF8yLmZpbC50X3QuQ29lbi4yID0gZGF0LmRvbF8yLmZpbC50X3QuQ29lbg0KZGF0LmRvbF8yLmZpbC50X3QuQ29lbi4yJHZhcmlhYmxlID0gcm93bmFtZXMoZGF0LmRvbF8yLmZpbC50X3QuQ29lbi4yKQ0KDQpkYXQuZG9sXzIuZmlsLnRfdC5Db2VuLjMgPSBkYXQuZG9sXzIuZmlsLnRfdC5Db2VuLjIgJT4lIA0KICAgICAgICAgICAgICAgICAgICAgICAgIG11dGF0ZSAoc3VtX29mX3Jvd3MgPSByb3dTdW1zKGRhdC5kb2xfMi5maWwudF90LkNvZW4uMlssMTo2XSkpDQoNCmRhdC5kb2xfMi5maWwudF90LkNvZW4uNCA9IGRhdC5kb2xfMi5maWwudF90LkNvZW4uMyAlPiUgZmlsdGVyIChzdW1fb2Zfcm93cyA+IDApDQoNCmRpbShkYXQuZG9sXzIuZmlsLnRfdC5Db2VuLjQpDQojIDU0MCAgIDgNCg0Kek9UVXMuQ29lbiA9IGRhdC5kb2xfMi5maWwudF90LkNvZW4uNCR2YXJpYWJsZQ0KDQpkYXQuZG9sXzIuZmlsLnRfdC5Db2VuLjUgPSBkYXQuZG9sXzIuZmlsLnRfdC5Db2VuLjRbLC1jKDcsOCldDQpyb3duYW1lcyhkYXQuZG9sXzIuZmlsLnRfdC5Db2VuLjUpID0gZGF0LmRvbF8yLmZpbC50X3QuQ29lbi40JHZhcmlhYmxlDQoNCmF0dHJpYnV0ZXMuZG9sX3JkcF9maWwuMy5Db2VuID0gYXR0cmlidXRlcy5kb2xfcmRwX2ZpbC4zIFt6T1RVcy5Db2VuLC0xXQ0KDQojIyB0aGV5IG5lZWQgdG8gYmVjb21lIG1hdHJpeCBmaWxlcyB0byB3b3JrIHdpdGggcGh5bG9zZXENCndoYWxlX290dV9ubS5Db2VuID0gYXMubWF0cml4KHNhcHBseShkYXQuZG9sXzIuZmlsLnRfdC5Db2VuLjUsIGFzLm51bWVyaWMpKSANCndoYWxlX3RheF9jbS5Db2VuID0gYXMubWF0cml4KHNhcHBseShhdHRyaWJ1dGVzLmRvbF9yZHBfZmlsLjMuQ29lbiwgYXMuY2hhcmFjdGVyKSkNCg0KRG9sQmxvd192YXIuZmlsLkNvZW4kU3BlY2llcyA8LSBhcy5jaGFyYWN0ZXIoRG9sQmxvd192YXIuZmlsLkNvZW4kU3BlY2llcykNCkRvbEJsb3dfdmFyLmZpbC5Db2VuJERvbHBoaW4gPC0gYXMuY2hhcmFjdGVyKERvbEJsb3dfdmFyLmZpbC5Db2VuJERvbHBoaW4pDQoNCiMjIGNvbnZlcnQgZmlsZXMgaW50byBwaHlsb3NlcSBvYmplY3QgdHlwZXMNCk9UVS5Db2VuID0gb3R1X3RhYmxlKHdoYWxlX290dV9ubS5Db2VuLCB0YXhhX2FyZV9yb3dzID0gVFJVRSkNClRBWC5Db2VuID0gdGF4X3RhYmxlKHdoYWxlX3RheF9jbS5Db2VuKQ0Kd2hhbGVkYXRhLkNvZW4gPSBzYW1wbGVfZGF0YShkYXRhLmZyYW1lKERvbEJsb3dfdmFyLmZpbC5Db2VuLCBzdHJpbmdzQXNGYWN0b3JzID0gRikpDQoNCiMjIG1lcmdlIHRoZSBmaWxlcyB3aXRoIHBoeWxvc2VxDQpEb2xwaGluLkNvZW4gPSBwaHlsb3NlcShPVFUuQ29lbiwgVEFYLkNvZW4sIHdoYWxlZGF0YS5Db2VuKQ0KDQpEb2xwaGluLkNvZW4uMiA8LSBtZXRob2RzOjphcyhwaHlsb3NlcTo6b3R1X3RhYmxlKERvbHBoaW4uQ29lbiksICJtYXRyaXgiKQ0KDQppZiAocGh5bG9zZXE6OnRheGFfYXJlX3Jvd3MoRG9scGhpbi5Db2VuKSkgeyBEb2xwaGluLkNvZW4uMiA8LSB0KERvbHBoaW4uQ29lbi4yKSB9DQoNCnNwZWNudW1iZXIoRG9scGhpbi5Db2VuLjIpDQoNCiNSYXJlZmFjdGlvbiBjdXJ2ZXMNCnAuQ29lbiA9IGdncmFyZShEb2xwaGluLkNvZW4sIHN0ZXAgPSAxMDAsIGNvbG9yID0gIkRvbHBoaW4iLCBzZSA9IEZBTFNFKQ0KDQpwLkNvZW5fMiA9IHAuQ29lbiArIGZhY2V0X3dyYXAoflNwZWNpZXMpDQoNCmdnc2F2ZSgicmFyZWZhY3Rpb24uY3VydmVzLkNvZW4uanBnIiwgcGxvdCA9IHAuQ29lbl8yICwgZGV2aWNlID0gJ2pwZycsIHdpZHRoID0gMTY4LCBoZWlnaHQgPSAxMzAsIHVuaXRzID0gIm1tIiwNCiAgICAgICBkcGkgPSAzMDAsIGxpbWl0c2l6ZSA9IFRSVUUpDQoNCiMjIyMjIyMjIyMjIyMjIyMjIyMjIyMjIyMjDQojI0dldCBnb29kJ3MgY292ZXJhZ2UNCiMjIG5lZWQgc2FtcGxlcyBhcyByb3dzDQp0Lk9UVS50YWJsZS5Db2VuID0gdChvdHVfdGFibGUoRG9scGhpbi5Db2VuKSkgIyB0cmFuc3Bvc2UgdGhlIHRhYmxlDQpzYW1wbGVfZGF0YShEb2xwaGluLkNvZW4pDQoNCiNkZXZ0b29sczo6aW5zdGFsbF9naXRodWIoImpmcTMvUXNSdXRpbHMiKQ0KbGlicmFyeShRc1J1dGlscykNCg0KZ29vZHMuQ29lbiA9IGdvb2RzKG90dV90YWJsZSh0Lk9UVS50YWJsZS5Db2VuKSkNCg0KdmFyLkNvZW4gPSBzYW1wbGVfZGF0YShEb2xwaGluLkNvZW4pDQpnb29kc192YXIuQ29lbiA9IGNiaW5kKGdvb2RzLkNvZW4sICB2YXIuQ29lbikNCg0KZ29vZHMuQ29lbg0KDQptZWFuKGdvb2RzLkNvZW4kZ29vZHMpIA0KDQpzZChnb29kcy5Db2VuJGdvb2RzKQ0KYGBgDQoNCiMjIyMjIyMjIyMjIyMjIyMjIyMNCiMjI0V2aWUNCmBgYHtyfQ0KIyMjRXZpZQ0KDQojZGF0LmRvbF8yLmZpbC50X3QsIERvbEJsb3dfdmFyLmZpbC4yLCBhdHRyaWJ1dGVzLmRvbF9yZHBfZmlsLjMNCiNuYW1lcyhkYXQuZG9sXzIuZmlsLnRfdCkNCiNEb2xCbG93X3Zhci5maWwuMiREb2xwaGluDQoNCmRhdC5kb2xfMi5maWwudF90LkV2aWUgPSBkYXQuZG9sXzIuZmlsLnRfdFssYygzMjozNyldDQpEb2xCbG93X3Zhci5maWwuRXZpZSA9IERvbEJsb3dfdmFyLmZpbC4yW2MoMzI6MzcpLF0NCnJvd25hbWVzKERvbEJsb3dfdmFyLmZpbC5FdmllKSA9IERvbEJsb3dfdmFyLmZpbC5FdmllJERvbHBoaW4NCg0KZGF0LmRvbF8yLmZpbC50X3QuRXZpZS4yID0gZGF0LmRvbF8yLmZpbC50X3QuRXZpZQ0KZGF0LmRvbF8yLmZpbC50X3QuRXZpZS4yJHZhcmlhYmxlID0gcm93bmFtZXMoZGF0LmRvbF8yLmZpbC50X3QuRXZpZS4yKQ0KDQpkYXQuZG9sXzIuZmlsLnRfdC5FdmllLjMgPSBkYXQuZG9sXzIuZmlsLnRfdC5FdmllLjIgJT4lIA0KICAgICAgICAgICAgICAgICAgICAgICAgIG11dGF0ZSAoc3VtX29mX3Jvd3MgPSByb3dTdW1zKGRhdC5kb2xfMi5maWwudF90LkV2aWUuMlssMTo2XSkpDQoNCmRhdC5kb2xfMi5maWwudF90LkV2aWUuNCA9IGRhdC5kb2xfMi5maWwudF90LkV2aWUuMyAlPiUgZmlsdGVyIChzdW1fb2Zfcm93cyA+IDApDQoNCmRpbShkYXQuZG9sXzIuZmlsLnRfdC5FdmllLjQpDQojIDU0MCAgIDgNCg0Kek9UVXMuRXZpZSA9IGRhdC5kb2xfMi5maWwudF90LkV2aWUuNCR2YXJpYWJsZQ0KDQpkYXQuZG9sXzIuZmlsLnRfdC5FdmllLjUgPSBkYXQuZG9sXzIuZmlsLnRfdC5FdmllLjRbLC1jKDcsOCldDQpyb3duYW1lcyhkYXQuZG9sXzIuZmlsLnRfdC5FdmllLjUpID0gZGF0LmRvbF8yLmZpbC50X3QuRXZpZS40JHZhcmlhYmxlDQoNCmF0dHJpYnV0ZXMuZG9sX3JkcF9maWwuMy5FdmllID0gYXR0cmlidXRlcy5kb2xfcmRwX2ZpbC4zIFt6T1RVcy5FdmllLC0xXQ0KDQojIyB0aGV5IG5lZWQgdG8gYmVjb21lIG1hdHJpeCBmaWxlcyB0byB3b3JrIHdpdGggcGh5bG9zZXENCndoYWxlX290dV9ubS5FdmllID0gYXMubWF0cml4KHNhcHBseShkYXQuZG9sXzIuZmlsLnRfdC5FdmllLjUsIGFzLm51bWVyaWMpKSANCndoYWxlX3RheF9jbS5FdmllID0gYXMubWF0cml4KHNhcHBseShhdHRyaWJ1dGVzLmRvbF9yZHBfZmlsLjMuRXZpZSwgYXMuY2hhcmFjdGVyKSkNCg0KRG9sQmxvd192YXIuZmlsLkV2aWUkU3BlY2llcyA8LSBhcy5jaGFyYWN0ZXIoRG9sQmxvd192YXIuZmlsLkV2aWUkU3BlY2llcykNCkRvbEJsb3dfdmFyLmZpbC5FdmllJERvbHBoaW4gPC0gYXMuY2hhcmFjdGVyKERvbEJsb3dfdmFyLmZpbC5FdmllJERvbHBoaW4pDQoNCiMjIGNvbnZlcnQgZmlsZXMgaW50byBwaHlsb3NlcSBvYmplY3QgdHlwZXMNCk9UVS5FdmllID0gb3R1X3RhYmxlKHdoYWxlX290dV9ubS5FdmllLCB0YXhhX2FyZV9yb3dzID0gVFJVRSkNClRBWC5FdmllID0gdGF4X3RhYmxlKHdoYWxlX3RheF9jbS5FdmllKQ0Kd2hhbGVkYXRhLkV2aWUgPSBzYW1wbGVfZGF0YShkYXRhLmZyYW1lKERvbEJsb3dfdmFyLmZpbC5FdmllLCBzdHJpbmdzQXNGYWN0b3JzID0gRikpDQoNCiMjIG1lcmdlIHRoZSBmaWxlcyB3aXRoIHBoeWxvc2VxDQpEb2xwaGluLkV2aWUgPSBwaHlsb3NlcShPVFUuRXZpZSwgVEFYLkV2aWUsIHdoYWxlZGF0YS5FdmllKQ0KDQpEb2xwaGluLkV2aWUuMiA8LSBtZXRob2RzOjphcyhwaHlsb3NlcTo6b3R1X3RhYmxlKERvbHBoaW4uRXZpZSksICJtYXRyaXgiKQ0KDQppZiAocGh5bG9zZXE6OnRheGFfYXJlX3Jvd3MoRG9scGhpbi5FdmllKSkgeyBEb2xwaGluLkV2aWUuMiA8LSB0KERvbHBoaW4uRXZpZS4yKSB9DQoNCnNwZWNudW1iZXIoRG9scGhpbi5FdmllLjIpDQoNCiNSYXJlZmFjdGlvbiBjdXJ2ZXMNCnAuRXZpZSA9IGdncmFyZShEb2xwaGluLkV2aWUsIHN0ZXAgPSAxMDAsIGNvbG9yID0gIkRvbHBoaW4iLCBzZSA9IEZBTFNFKQ0KDQpwLkV2aWVfMiA9IHAuRXZpZSArIGZhY2V0X3dyYXAoflNwZWNpZXMpDQoNCmdnc2F2ZSgicmFyZWZhY3Rpb24uY3VydmVzLkV2aWUuanBnIiwgcGxvdCA9IHAuRXZpZV8yICwgZGV2aWNlID0gJ2pwZycsIHdpZHRoID0gMTY4LCBoZWlnaHQgPSAxMzAsIHVuaXRzID0gIm1tIiwNCiAgICAgICBkcGkgPSAzMDAsIGxpbWl0c2l6ZSA9IFRSVUUpDQoNCiMjIyMjIyMjIyMjIyMjIyMjIyMjIyMjIyMjDQojI0dldCBnb29kJ3MgY292ZXJhZ2UNCiMjIG5lZWQgc2FtcGxlcyBhcyByb3dzDQp0Lk9UVS50YWJsZS5FdmllID0gdChvdHVfdGFibGUoRG9scGhpbi5FdmllKSkgIyB0cmFuc3Bvc2UgdGhlIHRhYmxlDQpzYW1wbGVfZGF0YShEb2xwaGluLkV2aWUpDQoNCiNkZXZ0b29sczo6aW5zdGFsbF9naXRodWIoImpmcTMvUXNSdXRpbHMiKQ0KbGlicmFyeShRc1J1dGlscykNCg0KZ29vZHMuRXZpZSA9IGdvb2RzKG90dV90YWJsZSh0Lk9UVS50YWJsZS5FdmllKSkNCg0KdmFyLkV2aWUgPSBzYW1wbGVfZGF0YShEb2xwaGluLkV2aWUpDQpnb29kc192YXIuRXZpZSA9IGNiaW5kKGdvb2RzLkV2aWUsICB2YXIuRXZpZSkNCg0KZ29vZHMuRXZpZQ0KDQptZWFuKGdvb2RzLkV2aWUkZ29vZHMpDQoNCnNkKGdvb2RzLkV2aWUkZ29vZHMpDQpgYGANCg0KIyMjIyMjIyMjIyMjIyMjIyMjIyMjIyMjIyMjIyMjIyMNCiMjI0tpYW1hDQpgYGB7cn0NCiMjI0tpYW1hDQoNCiNkYXQuZG9sXzIuZmlsLnRfdCwgRG9sQmxvd192YXIuZmlsLjIsIGF0dHJpYnV0ZXMuZG9sX3JkcF9maWwuMw0KI25hbWVzKGRhdC5kb2xfMi5maWwudF90KQ0KI0RvbEJsb3dfdmFyLmZpbC4yJERvbHBoaW4NCg0KZGF0LmRvbF8yLmZpbC50X3QuS2lhbWEgPSBkYXQuZG9sXzIuZmlsLnRfdFssYyg1Mjo1NildDQpEb2xCbG93X3Zhci5maWwuS2lhbWEgPSBEb2xCbG93X3Zhci5maWwuMltjKDUyOjU2KSxdDQpyb3duYW1lcyhEb2xCbG93X3Zhci5maWwuS2lhbWEpID0gRG9sQmxvd192YXIuZmlsLktpYW1hJERvbHBoaW4NCg0KZGF0LmRvbF8yLmZpbC50X3QuS2lhbWEuMiA9IGRhdC5kb2xfMi5maWwudF90LktpYW1hDQpkYXQuZG9sXzIuZmlsLnRfdC5LaWFtYS4yJHZhcmlhYmxlID0gcm93bmFtZXMoZGF0LmRvbF8yLmZpbC50X3QuS2lhbWEuMikNCg0KZGF0LmRvbF8yLmZpbC50X3QuS2lhbWEuMyA9IGRhdC5kb2xfMi5maWwudF90LktpYW1hLjIgJT4lIA0KICAgICAgICAgICAgICAgICAgICAgICAgIG11dGF0ZSAoc3VtX29mX3Jvd3MgPSByb3dTdW1zKGRhdC5kb2xfMi5maWwudF90LktpYW1hLjJbLDE6NV0pKQ0KDQpkYXQuZG9sXzIuZmlsLnRfdC5LaWFtYS40ID0gZGF0LmRvbF8yLmZpbC50X3QuS2lhbWEuMyAlPiUgZmlsdGVyIChzdW1fb2Zfcm93cyA+IDApDQoNCmRpbShkYXQuZG9sXzIuZmlsLnRfdC5LaWFtYS40KQ0KIyA1NDAgICA4DQoNCnpPVFVzLktpYW1hID0gZGF0LmRvbF8yLmZpbC50X3QuS2lhbWEuNCR2YXJpYWJsZQ0KDQpkYXQuZG9sXzIuZmlsLnRfdC5LaWFtYS41ID0gZGF0LmRvbF8yLmZpbC50X3QuS2lhbWEuNFssLWMoNiw3KV0NCnJvd25hbWVzKGRhdC5kb2xfMi5maWwudF90LktpYW1hLjUpID0gZGF0LmRvbF8yLmZpbC50X3QuS2lhbWEuNCR2YXJpYWJsZQ0KDQphdHRyaWJ1dGVzLmRvbF9yZHBfZmlsLjMuS2lhbWEgPSBhdHRyaWJ1dGVzLmRvbF9yZHBfZmlsLjMgW3pPVFVzLktpYW1hLC0xXQ0KDQojIyB0aGV5IG5lZWQgdG8gYmVjb21lIG1hdHJpeCBmaWxlcyB0byB3b3JrIHdpdGggcGh5bG9zZXENCndoYWxlX290dV9ubS5LaWFtYSA9IGFzLm1hdHJpeChzYXBwbHkoZGF0LmRvbF8yLmZpbC50X3QuS2lhbWEuNSwgYXMubnVtZXJpYykpIA0Kd2hhbGVfdGF4X2NtLktpYW1hID0gYXMubWF0cml4KHNhcHBseShhdHRyaWJ1dGVzLmRvbF9yZHBfZmlsLjMuS2lhbWEsIGFzLmNoYXJhY3RlcikpDQoNCkRvbEJsb3dfdmFyLmZpbC5LaWFtYSRTcGVjaWVzIDwtIGFzLmNoYXJhY3RlcihEb2xCbG93X3Zhci5maWwuS2lhbWEkU3BlY2llcykNCkRvbEJsb3dfdmFyLmZpbC5LaWFtYSREb2xwaGluIDwtIGFzLmNoYXJhY3RlcihEb2xCbG93X3Zhci5maWwuS2lhbWEkRG9scGhpbikNCg0KIyMgY29udmVydCBmaWxlcyBpbnRvIHBoeWxvc2VxIG9iamVjdCB0eXBlcw0KT1RVLktpYW1hID0gb3R1X3RhYmxlKHdoYWxlX290dV9ubS5LaWFtYSwgdGF4YV9hcmVfcm93cyA9IFRSVUUpDQpUQVguS2lhbWEgPSB0YXhfdGFibGUod2hhbGVfdGF4X2NtLktpYW1hKQ0Kd2hhbGVkYXRhLktpYW1hID0gc2FtcGxlX2RhdGEoZGF0YS5mcmFtZShEb2xCbG93X3Zhci5maWwuS2lhbWEsIHN0cmluZ3NBc0ZhY3RvcnMgPSBGKSkNCg0KIyMgbWVyZ2UgdGhlIGZpbGVzIHdpdGggcGh5bG9zZXENCkRvbHBoaW4uS2lhbWEgPSBwaHlsb3NlcShPVFUuS2lhbWEsIFRBWC5LaWFtYSwgd2hhbGVkYXRhLktpYW1hKQ0KDQpEb2xwaGluLktpYW1hLjIgPC0gbWV0aG9kczo6YXMocGh5bG9zZXE6Om90dV90YWJsZShEb2xwaGluLktpYW1hKSwgIm1hdHJpeCIpDQoNCmlmIChwaHlsb3NlcTo6dGF4YV9hcmVfcm93cyhEb2xwaGluLktpYW1hKSkgeyBEb2xwaGluLktpYW1hLjIgPC0gdChEb2xwaGluLktpYW1hLjIpIH0NCg0Kc3BlY251bWJlcihEb2xwaGluLktpYW1hLjIpDQoNCiNSYXJlZmFjdGlvbiBjdXJ2ZXMNCnAuS2lhbWEgPSBnZ3JhcmUoRG9scGhpbi5LaWFtYSwgc3RlcCA9IDEwMCwgY29sb3IgPSAiRG9scGhpbiIsIHNlID0gRkFMU0UpDQoNCnAuS2lhbWFfMiA9IHAuS2lhbWEgKyBmYWNldF93cmFwKH5TcGVjaWVzKQ0KDQpnZ3NhdmUoInJhcmVmYWN0aW9uLmN1cnZlcy5LaWFtYS5qcGciLCBwbG90ID0gcC5LaWFtYV8yICwgZGV2aWNlID0gJ2pwZycsIHdpZHRoID0gMTY4LCBoZWlnaHQgPSAxMzAsIHVuaXRzID0gIm1tIiwNCiAgICAgICBkcGkgPSAzMDAsIGxpbWl0c2l6ZSA9IFRSVUUpDQoNCiMjIyMjIyMjIyMjIyMjIyMjIyMjIyMjIyMjDQojI0dldCBnb29kJ3MgY292ZXJhZ2UNCiMjIG5lZWQgc2FtcGxlcyBhcyByb3dzDQp0Lk9UVS50YWJsZS5LaWFtYSA9IHQob3R1X3RhYmxlKERvbHBoaW4uS2lhbWEpKSAjIHRyYW5zcG9zZSB0aGUgdGFibGUNCnNhbXBsZV9kYXRhKERvbHBoaW4uS2lhbWEpDQoNCiNkZXZ0b29sczo6aW5zdGFsbF9naXRodWIoImpmcTMvUXNSdXRpbHMiKQ0KbGlicmFyeShRc1J1dGlscykNCg0KZ29vZHMuS2lhbWEgPSBnb29kcyhvdHVfdGFibGUodC5PVFUudGFibGUuS2lhbWEpKQ0KDQp2YXIuS2lhbWEgPSBzYW1wbGVfZGF0YShEb2xwaGluLktpYW1hKQ0KZ29vZHNfdmFyLktpYW1hID0gY2JpbmQoZ29vZHMuS2lhbWEsICB2YXIuS2lhbWEpDQoNCmdvb2RzLktpYW1hDQoNCm1lYW4oZ29vZHMuS2lhbWEkZ29vZHMpDQoNCnNkKGdvb2RzLktpYW1hJGdvb2RzKQ0KYGBgDQoNCiMjIyMjIyMjIyMjIyMjIyMjIyMjIyMjIyMjIyMjIyMjDQojIyNNb2tpDQpgYGB7cn0NCiMjI01va2kNCg0KI2RhdC5kb2xfMi5maWwudF90LCBEb2xCbG93X3Zhci5maWwuMiwgYXR0cmlidXRlcy5kb2xfcmRwX2ZpbC4zDQojbmFtZXMoZGF0LmRvbF8yLmZpbC50X3QpDQojRG9sQmxvd192YXIuZmlsLjIkRG9scGhpbg0KDQpkYXQuZG9sXzIuZmlsLnRfdC5Nb2tpID0gZGF0LmRvbF8yLmZpbC50X3RbLGMoNTc6NjIpXQ0KRG9sQmxvd192YXIuZmlsLk1va2kgPSBEb2xCbG93X3Zhci5maWwuMltjKDU3OjYyKSxdDQpyb3duYW1lcyhEb2xCbG93X3Zhci5maWwuTW9raSkgPSBEb2xCbG93X3Zhci5maWwuTW9raSREb2xwaGluDQoNCmRhdC5kb2xfMi5maWwudF90Lk1va2kuMiA9IGRhdC5kb2xfMi5maWwudF90Lk1va2kNCmRhdC5kb2xfMi5maWwudF90Lk1va2kuMiR2YXJpYWJsZSA9IHJvd25hbWVzKGRhdC5kb2xfMi5maWwudF90Lk1va2kuMikNCg0KZGF0LmRvbF8yLmZpbC50X3QuTW9raS4zID0gZGF0LmRvbF8yLmZpbC50X3QuTW9raS4yICU+JSANCiAgICAgICAgICAgICAgICAgICAgICAgICBtdXRhdGUgKHN1bV9vZl9yb3dzID0gcm93U3VtcyhkYXQuZG9sXzIuZmlsLnRfdC5Nb2tpLjJbLDE6Nl0pKQ0KDQpkYXQuZG9sXzIuZmlsLnRfdC5Nb2tpLjQgPSBkYXQuZG9sXzIuZmlsLnRfdC5Nb2tpLjMgJT4lIGZpbHRlciAoc3VtX29mX3Jvd3MgPiAwKQ0KDQpkaW0oZGF0LmRvbF8yLmZpbC50X3QuTW9raS40KQ0KIyA1NDAgICA4DQoNCnpPVFVzLk1va2kgPSBkYXQuZG9sXzIuZmlsLnRfdC5Nb2tpLjQkdmFyaWFibGUNCg0KZGF0LmRvbF8yLmZpbC50X3QuTW9raS41ID0gZGF0LmRvbF8yLmZpbC50X3QuTW9raS40WywtYyg3LDgpXQ0Kcm93bmFtZXMoZGF0LmRvbF8yLmZpbC50X3QuTW9raS41KSA9IGRhdC5kb2xfMi5maWwudF90Lk1va2kuNCR2YXJpYWJsZQ0KDQphdHRyaWJ1dGVzLmRvbF9yZHBfZmlsLjMuTW9raSA9IGF0dHJpYnV0ZXMuZG9sX3JkcF9maWwuMyBbek9UVXMuTW9raSwtMV0NCg0KIyMgdGhleSBuZWVkIHRvIGJlY29tZSBtYXRyaXggZmlsZXMgdG8gd29yayB3aXRoIHBoeWxvc2VxDQp3aGFsZV9vdHVfbm0uTW9raSA9IGFzLm1hdHJpeChzYXBwbHkoZGF0LmRvbF8yLmZpbC50X3QuTW9raS41LCBhcy5udW1lcmljKSkgDQp3aGFsZV90YXhfY20uTW9raSA9IGFzLm1hdHJpeChzYXBwbHkoYXR0cmlidXRlcy5kb2xfcmRwX2ZpbC4zLk1va2ksIGFzLmNoYXJhY3RlcikpDQoNCkRvbEJsb3dfdmFyLmZpbC5Nb2tpJFNwZWNpZXMgPC0gYXMuY2hhcmFjdGVyKERvbEJsb3dfdmFyLmZpbC5Nb2tpJFNwZWNpZXMpDQpEb2xCbG93X3Zhci5maWwuTW9raSREb2xwaGluIDwtIGFzLmNoYXJhY3RlcihEb2xCbG93X3Zhci5maWwuTW9raSREb2xwaGluKQ0KDQojIyBjb252ZXJ0IGZpbGVzIGludG8gcGh5bG9zZXEgb2JqZWN0IHR5cGVzDQpPVFUuTW9raSA9IG90dV90YWJsZSh3aGFsZV9vdHVfbm0uTW9raSwgdGF4YV9hcmVfcm93cyA9IFRSVUUpDQpUQVguTW9raSA9IHRheF90YWJsZSh3aGFsZV90YXhfY20uTW9raSkNCndoYWxlZGF0YS5Nb2tpID0gc2FtcGxlX2RhdGEoZGF0YS5mcmFtZShEb2xCbG93X3Zhci5maWwuTW9raSwgc3RyaW5nc0FzRmFjdG9ycyA9IEYpKQ0KDQojIyBtZXJnZSB0aGUgZmlsZXMgd2l0aCBwaHlsb3NlcQ0KRG9scGhpbi5Nb2tpID0gcGh5bG9zZXEoT1RVLk1va2ksIFRBWC5Nb2tpLCB3aGFsZWRhdGEuTW9raSkNCg0KRG9scGhpbi5Nb2tpLjIgPC0gbWV0aG9kczo6YXMocGh5bG9zZXE6Om90dV90YWJsZShEb2xwaGluLk1va2kpLCAibWF0cml4IikNCg0KaWYgKHBoeWxvc2VxOjp0YXhhX2FyZV9yb3dzKERvbHBoaW4uTW9raSkpIHsgRG9scGhpbi5Nb2tpLjIgPC0gdChEb2xwaGluLk1va2kuMikgfQ0KDQpzcGVjbnVtYmVyKERvbHBoaW4uTW9raS4yKQ0KDQojUmFyZWZhY3Rpb24gY3VydmVzDQpwLk1va2kgPSBnZ3JhcmUoRG9scGhpbi5Nb2tpLCBzdGVwID0gMTAwLCBjb2xvciA9ICJEb2xwaGluIiwgc2UgPSBGQUxTRSkNCg0KcC5Nb2tpXzIgPSBwLk1va2kgKyBmYWNldF93cmFwKH5TcGVjaWVzKQ0KDQpnZ3NhdmUoInJhcmVmYWN0aW9uLmN1cnZlcy5Nb2tpLmpwZyIsIHBsb3QgPSBwLk1va2lfMiAsIGRldmljZSA9ICdqcGcnLCB3aWR0aCA9IDE2OCwgaGVpZ2h0ID0gMTMwLCB1bml0cyA9ICJtbSIsDQogICAgICAgZHBpID0gMzAwLCBsaW1pdHNpemUgPSBUUlVFKQ0KDQojIyMjIyMjIyMjIyMjIyMjIyMjIyMjIyMjIw0KIyNHZXQgZ29vZCdzIGNvdmVyYWdlDQojIyBuZWVkIHNhbXBsZXMgYXMgcm93cw0KdC5PVFUudGFibGUuTW9raSA9IHQob3R1X3RhYmxlKERvbHBoaW4uTW9raSkpICMgdHJhbnNwb3NlIHRoZSB0YWJsZQ0Kc2FtcGxlX2RhdGEoRG9scGhpbi5Nb2tpKQ0KDQojZGV2dG9vbHM6Omluc3RhbGxfZ2l0aHViKCJqZnEzL1FzUnV0aWxzIikNCmxpYnJhcnkoUXNSdXRpbHMpDQoNCmdvb2RzLk1va2kgPSBnb29kcyhvdHVfdGFibGUodC5PVFUudGFibGUuTW9raSkpDQoNCnZhci5Nb2tpID0gc2FtcGxlX2RhdGEoRG9scGhpbi5Nb2tpKQ0KZ29vZHNfdmFyLk1va2kgPSBjYmluZChnb29kcy5Nb2tpLCAgdmFyLk1va2kpDQoNCmdvb2RzLk1va2kNCg0KbWVhbihnb29kcy5Nb2tpJGdvb2RzKQ0KDQpzZChnb29kcy5Nb2tpJGdvb2RzKQ0KYGBgDQoNCiMjIyMjIyMjIyMjIyMjIyMjIyMjIyMjIyMjIyMjIyMjDQojIyNSQg0KYGBge3J9DQojIyNSQg0KDQojZGF0LmRvbF8yLmZpbC50X3QsIERvbEJsb3dfdmFyLmZpbC4yLCBhdHRyaWJ1dGVzLmRvbF9yZHBfZmlsLjMNCiNuYW1lcyhkYXQuZG9sXzIuZmlsLnRfdCkNCiNEb2xCbG93X3Zhci5maWwuMiREb2xwaGluDQoNCmRhdC5kb2xfMi5maWwudF90LlJCID0gZGF0LmRvbF8yLmZpbC50X3RbLGMoNzU6ODApXQ0KRG9sQmxvd192YXIuZmlsLlJCID0gRG9sQmxvd192YXIuZmlsLjJbYyg3NTo4MCksXQ0Kcm93bmFtZXMoRG9sQmxvd192YXIuZmlsLlJCKSA9IERvbEJsb3dfdmFyLmZpbC5SQiREb2xwaGluDQoNCmRhdC5kb2xfMi5maWwudF90LlJCLjIgPSBkYXQuZG9sXzIuZmlsLnRfdC5SQg0KZGF0LmRvbF8yLmZpbC50X3QuUkIuMiR2YXJpYWJsZSA9IHJvd25hbWVzKGRhdC5kb2xfMi5maWwudF90LlJCLjIpDQoNCmRhdC5kb2xfMi5maWwudF90LlJCLjMgPSBkYXQuZG9sXzIuZmlsLnRfdC5SQi4yICU+JSANCiAgICAgICAgICAgICAgICAgICAgICAgICBtdXRhdGUgKHN1bV9vZl9yb3dzID0gcm93U3VtcyhkYXQuZG9sXzIuZmlsLnRfdC5SQi4yWywxOjZdKSkNCg0KZGF0LmRvbF8yLmZpbC50X3QuUkIuNCA9IGRhdC5kb2xfMi5maWwudF90LlJCLjMgJT4lIGZpbHRlciAoc3VtX29mX3Jvd3MgPiAwKQ0KDQpkaW0oZGF0LmRvbF8yLmZpbC50X3QuUkIuNCkNCiMgNTQwICAgOA0KDQp6T1RVcy5SQiA9IGRhdC5kb2xfMi5maWwudF90LlJCLjQkdmFyaWFibGUNCg0KZGF0LmRvbF8yLmZpbC50X3QuUkIuNSA9IGRhdC5kb2xfMi5maWwudF90LlJCLjRbLC1jKDcsOCldDQpyb3duYW1lcyhkYXQuZG9sXzIuZmlsLnRfdC5SQi41KSA9IGRhdC5kb2xfMi5maWwudF90LlJCLjQkdmFyaWFibGUNCg0KYXR0cmlidXRlcy5kb2xfcmRwX2ZpbC4zLlJCID0gYXR0cmlidXRlcy5kb2xfcmRwX2ZpbC4zIFt6T1RVcy5SQiwtMV0NCg0KIyMgdGhleSBuZWVkIHRvIGJlY29tZSBtYXRyaXggZmlsZXMgdG8gd29yayB3aXRoIHBoeWxvc2VxDQp3aGFsZV9vdHVfbm0uUkIgPSBhcy5tYXRyaXgoc2FwcGx5KGRhdC5kb2xfMi5maWwudF90LlJCLjUsIGFzLm51bWVyaWMpKSANCndoYWxlX3RheF9jbS5SQiA9IGFzLm1hdHJpeChzYXBwbHkoYXR0cmlidXRlcy5kb2xfcmRwX2ZpbC4zLlJCLCBhcy5jaGFyYWN0ZXIpKQ0KDQpEb2xCbG93X3Zhci5maWwuUkIkU3BlY2llcyA8LSBhcy5jaGFyYWN0ZXIoRG9sQmxvd192YXIuZmlsLlJCJFNwZWNpZXMpDQpEb2xCbG93X3Zhci5maWwuUkIkRG9scGhpbiA8LSBhcy5jaGFyYWN0ZXIoRG9sQmxvd192YXIuZmlsLlJCJERvbHBoaW4pDQoNCiMjIGNvbnZlcnQgZmlsZXMgaW50byBwaHlsb3NlcSBvYmplY3QgdHlwZXMNCk9UVS5SQiA9IG90dV90YWJsZSh3aGFsZV9vdHVfbm0uUkIsIHRheGFfYXJlX3Jvd3MgPSBUUlVFKQ0KVEFYLlJCID0gdGF4X3RhYmxlKHdoYWxlX3RheF9jbS5SQikNCndoYWxlZGF0YS5SQiA9IHNhbXBsZV9kYXRhKGRhdGEuZnJhbWUoRG9sQmxvd192YXIuZmlsLlJCLCBzdHJpbmdzQXNGYWN0b3JzID0gRikpDQoNCiMjIG1lcmdlIHRoZSBmaWxlcyB3aXRoIHBoeWxvc2VxDQpEb2xwaGluLlJCID0gcGh5bG9zZXEoT1RVLlJCLCBUQVguUkIsIHdoYWxlZGF0YS5SQikNCg0KRG9scGhpbi5SQi4yIDwtIG1ldGhvZHM6OmFzKHBoeWxvc2VxOjpvdHVfdGFibGUoRG9scGhpbi5SQiksICJtYXRyaXgiKQ0KDQppZiAocGh5bG9zZXE6OnRheGFfYXJlX3Jvd3MoRG9scGhpbi5SQikpIHsgRG9scGhpbi5SQi4yIDwtIHQoRG9scGhpbi5SQi4yKSB9DQoNCnNwZWNudW1iZXIoRG9scGhpbi5SQi4yKQ0KDQojUmFyZWZhY3Rpb24gY3VydmVzDQpwLlJCID0gZ2dyYXJlKERvbHBoaW4uUkIsIHN0ZXAgPSAxMDAsIGNvbG9yID0gIkRvbHBoaW4iLCBzZSA9IEZBTFNFKQ0KDQpwLlJCXzIgPSBwLlJCICsgZmFjZXRfd3JhcCh+U3BlY2llcykNCg0KZ2dzYXZlKCJyYXJlZmFjdGlvbi5jdXJ2ZXMuUkIuanBnIiwgcGxvdCA9IHAuUkJfMiAsIGRldmljZSA9ICdqcGcnLCB3aWR0aCA9IDE2OCwgaGVpZ2h0ID0gMTMwLCB1bml0cyA9ICJtbSIsDQogICAgICAgZHBpID0gMzAwLCBsaW1pdHNpemUgPSBUUlVFKQ0KDQojIyMjIyMjIyMjIyMjIyMjIyMjIyMjIyMjIw0KIyNHZXQgZ29vZCdzIGNvdmVyYWdlDQojIyBuZWVkIHNhbXBsZXMgYXMgcm93cw0KdC5PVFUudGFibGUuUkIgPSB0KG90dV90YWJsZShEb2xwaGluLlJCKSkgIyB0cmFuc3Bvc2UgdGhlIHRhYmxlDQpzYW1wbGVfZGF0YShEb2xwaGluLlJCKQ0KDQojZGV2dG9vbHM6Omluc3RhbGxfZ2l0aHViKCJqZnEzL1FzUnV0aWxzIikNCmxpYnJhcnkoUXNSdXRpbHMpDQoNCmdvb2RzLlJCID0gZ29vZHMob3R1X3RhYmxlKHQuT1RVLnRhYmxlLlJCKSkNCg0KdmFyLlJCID0gc2FtcGxlX2RhdGEoRG9scGhpbi5SQikNCmdvb2RzX3Zhci5SQiA9IGNiaW5kKGdvb2RzLlJCLCAgdmFyLlJCKQ0KDQpnb29kcy5SQg0KDQptZWFuKGdvb2RzLlJCJGdvb2RzKQ0KDQpzZChnb29kcy5SQiRnb29kcykNCmBgYA0KDQojIyMjIyMjIyMjIyMjIyMjIyMjIyMjIyMjIyMjIyMjIw0KIyMjU2Nvb3Rlcg0KYGBge3J9DQojIyNTY29vdGVyDQoNCiNkYXQuZG9sXzIuZmlsLnRfdCwgRG9sQmxvd192YXIuZmlsLjIsIGF0dHJpYnV0ZXMuZG9sX3JkcF9maWwuMw0KI25hbWVzKGRhdC5kb2xfMi5maWwudF90KQ0KI0RvbEJsb3dfdmFyLmZpbC4yJERvbHBoaW4NCg0KZGF0LmRvbF8yLmZpbC50X3QuU2Nvb3RlciA9IGRhdC5kb2xfMi5maWwudF90WyxjKDgxOjg1KV0NCkRvbEJsb3dfdmFyLmZpbC5TY29vdGVyID0gRG9sQmxvd192YXIuZmlsLjJbYyg4MTo4NSksXQ0Kcm93bmFtZXMoRG9sQmxvd192YXIuZmlsLlNjb290ZXIpID0gRG9sQmxvd192YXIuZmlsLlNjb290ZXIkRG9scGhpbg0KDQpkYXQuZG9sXzIuZmlsLnRfdC5TY29vdGVyLjIgPSBkYXQuZG9sXzIuZmlsLnRfdC5TY29vdGVyDQpkYXQuZG9sXzIuZmlsLnRfdC5TY29vdGVyLjIkdmFyaWFibGUgPSByb3duYW1lcyhkYXQuZG9sXzIuZmlsLnRfdC5TY29vdGVyLjIpDQoNCmRhdC5kb2xfMi5maWwudF90LlNjb290ZXIuMyA9IGRhdC5kb2xfMi5maWwudF90LlNjb290ZXIuMiAlPiUgDQogICAgICAgICAgICAgICAgICAgICAgICAgbXV0YXRlIChzdW1fb2Zfcm93cyA9IHJvd1N1bXMoZGF0LmRvbF8yLmZpbC50X3QuU2Nvb3Rlci4yWywxOjVdKSkNCg0KZGF0LmRvbF8yLmZpbC50X3QuU2Nvb3Rlci40ID0gZGF0LmRvbF8yLmZpbC50X3QuU2Nvb3Rlci4zICU+JSBmaWx0ZXIgKHN1bV9vZl9yb3dzID4gMCkNCg0KZGltKGRhdC5kb2xfMi5maWwudF90LlNjb290ZXIuNCkNCiMgNTQwICAgOA0KDQp6T1RVcy5TY29vdGVyID0gZGF0LmRvbF8yLmZpbC50X3QuU2Nvb3Rlci40JHZhcmlhYmxlDQoNCmRhdC5kb2xfMi5maWwudF90LlNjb290ZXIuNSA9IGRhdC5kb2xfMi5maWwudF90LlNjb290ZXIuNFssLWMoNiw3KV0NCnJvd25hbWVzKGRhdC5kb2xfMi5maWwudF90LlNjb290ZXIuNSkgPSBkYXQuZG9sXzIuZmlsLnRfdC5TY29vdGVyLjQkdmFyaWFibGUNCg0KYXR0cmlidXRlcy5kb2xfcmRwX2ZpbC4zLlNjb290ZXIgPSBhdHRyaWJ1dGVzLmRvbF9yZHBfZmlsLjMgW3pPVFVzLlNjb290ZXIsLTFdDQoNCiMjIHRoZXkgbmVlZCB0byBiZWNvbWUgbWF0cml4IGZpbGVzIHRvIHdvcmsgd2l0aCBwaHlsb3NlcQ0Kd2hhbGVfb3R1X25tLlNjb290ZXIgPSBhcy5tYXRyaXgoc2FwcGx5KGRhdC5kb2xfMi5maWwudF90LlNjb290ZXIuNSwgYXMubnVtZXJpYykpIA0Kd2hhbGVfdGF4X2NtLlNjb290ZXIgPSBhcy5tYXRyaXgoc2FwcGx5KGF0dHJpYnV0ZXMuZG9sX3JkcF9maWwuMy5TY29vdGVyLCBhcy5jaGFyYWN0ZXIpKQ0KDQpEb2xCbG93X3Zhci5maWwuU2Nvb3RlciRTcGVjaWVzIDwtIGFzLmNoYXJhY3RlcihEb2xCbG93X3Zhci5maWwuU2Nvb3RlciRTcGVjaWVzKQ0KRG9sQmxvd192YXIuZmlsLlNjb290ZXIkRG9scGhpbiA8LSBhcy5jaGFyYWN0ZXIoRG9sQmxvd192YXIuZmlsLlNjb290ZXIkRG9scGhpbikNCg0KIyMgY29udmVydCBmaWxlcyBpbnRvIHBoeWxvc2VxIG9iamVjdCB0eXBlcw0KT1RVLlNjb290ZXIgPSBvdHVfdGFibGUod2hhbGVfb3R1X25tLlNjb290ZXIsIHRheGFfYXJlX3Jvd3MgPSBUUlVFKQ0KVEFYLlNjb290ZXIgPSB0YXhfdGFibGUod2hhbGVfdGF4X2NtLlNjb290ZXIpDQp3aGFsZWRhdGEuU2Nvb3RlciA9IHNhbXBsZV9kYXRhKGRhdGEuZnJhbWUoRG9sQmxvd192YXIuZmlsLlNjb290ZXIsIHN0cmluZ3NBc0ZhY3RvcnMgPSBGKSkNCg0KIyMgbWVyZ2UgdGhlIGZpbGVzIHdpdGggcGh5bG9zZXENCkRvbHBoaW4uU2Nvb3RlciA9IHBoeWxvc2VxKE9UVS5TY29vdGVyLCBUQVguU2Nvb3Rlciwgd2hhbGVkYXRhLlNjb290ZXIpDQoNCkRvbHBoaW4uU2Nvb3Rlci4yIDwtIG1ldGhvZHM6OmFzKHBoeWxvc2VxOjpvdHVfdGFibGUoRG9scGhpbi5TY29vdGVyKSwgIm1hdHJpeCIpDQoNCmlmIChwaHlsb3NlcTo6dGF4YV9hcmVfcm93cyhEb2xwaGluLlNjb290ZXIpKSB7IERvbHBoaW4uU2Nvb3Rlci4yIDwtIHQoRG9scGhpbi5TY29vdGVyLjIpIH0NCg0Kc3BlY251bWJlcihEb2xwaGluLlNjb290ZXIuMikNCg0KI1JhcmVmYWN0aW9uIGN1cnZlcw0KcC5TY29vdGVyID0gZ2dyYXJlKERvbHBoaW4uU2Nvb3Rlciwgc3RlcCA9IDEwMCwgY29sb3IgPSAiRG9scGhpbiIsIHNlID0gRkFMU0UpDQoNCnAuU2Nvb3Rlcl8yID0gcC5TY29vdGVyICsgZmFjZXRfd3JhcCh+U3BlY2llcykNCg0KZ2dzYXZlKCJyYXJlZmFjdGlvbi5jdXJ2ZXMuU2Nvb3Rlci5qcGciLCBwbG90ID0gcC5TY29vdGVyXzIgLCBkZXZpY2UgPSAnanBnJywgd2lkdGggPSAxNjgsIGhlaWdodCA9IDEzMCwgdW5pdHMgPSAibW0iLA0KICAgICAgIGRwaSA9IDMwMCwgbGltaXRzaXplID0gVFJVRSkNCg0KIyMjIyMjIyMjIyMjIyMjIyMjIyMjIyMjIyMNCiMjR2V0IGdvb2QncyBjb3ZlcmFnZQ0KIyMgbmVlZCBzYW1wbGVzIGFzIHJvd3MNCnQuT1RVLnRhYmxlLlNjb290ZXIgPSB0KG90dV90YWJsZShEb2xwaGluLlNjb290ZXIpKSAjIHRyYW5zcG9zZSB0aGUgdGFibGUNCnNhbXBsZV9kYXRhKERvbHBoaW4uU2Nvb3RlcikNCg0KI2RldnRvb2xzOjppbnN0YWxsX2dpdGh1YigiamZxMy9Rc1J1dGlscyIpDQpsaWJyYXJ5KFFzUnV0aWxzKQ0KDQpnb29kcy5TY29vdGVyID0gZ29vZHMob3R1X3RhYmxlKHQuT1RVLnRhYmxlLlNjb290ZXIpKQ0KDQp2YXIuU2Nvb3RlciA9IHNhbXBsZV9kYXRhKERvbHBoaW4uU2Nvb3RlcikNCmdvb2RzX3Zhci5TY29vdGVyID0gY2JpbmQoZ29vZHMuU2Nvb3RlciwgIHZhci5TY29vdGVyKQ0KDQpnb29kcy5TY29vdGVyDQoNCm1lYW4oZ29vZHMuU2Nvb3RlciRnb29kcykNCg0Kc2QoZ29vZHMuU2Nvb3RlciRnb29kcykNCmBgYA0KDQojIyMjIyMjIyMjIyMjIyMjIyMjIyMjIyMjIyMjIyMjIw0KIyMjU2lyaXVzDQpgYGB7cn0NCiMjI1Npcml1cw0KDQojZGF0LmRvbF8yLmZpbC50X3QsIERvbEJsb3dfdmFyLmZpbC4yLCBhdHRyaWJ1dGVzLmRvbF9yZHBfZmlsLjMNCiNuYW1lcyhkYXQuZG9sXzIuZmlsLnRfdCkNCiNEb2xCbG93X3Zhci5maWwuMiREb2xwaGluDQoNCmRhdC5kb2xfMi5maWwudF90LlNpcml1cyA9IGRhdC5kb2xfMi5maWwudF90WyxjKDg2OjkxKV0NCkRvbEJsb3dfdmFyLmZpbC5TaXJpdXMgPSBEb2xCbG93X3Zhci5maWwuMltjKDg2OjkxKSxdDQpyb3duYW1lcyhEb2xCbG93X3Zhci5maWwuU2lyaXVzKSA9IERvbEJsb3dfdmFyLmZpbC5TaXJpdXMkRG9scGhpbg0KDQpkYXQuZG9sXzIuZmlsLnRfdC5TaXJpdXMuMiA9IGRhdC5kb2xfMi5maWwudF90LlNpcml1cw0KZGF0LmRvbF8yLmZpbC50X3QuU2lyaXVzLjIkdmFyaWFibGUgPSByb3duYW1lcyhkYXQuZG9sXzIuZmlsLnRfdC5TaXJpdXMuMikNCg0KZGF0LmRvbF8yLmZpbC50X3QuU2lyaXVzLjMgPSBkYXQuZG9sXzIuZmlsLnRfdC5TaXJpdXMuMiAlPiUgDQogICAgICAgICAgICAgICAgICAgICAgICAgbXV0YXRlIChzdW1fb2Zfcm93cyA9IHJvd1N1bXMoZGF0LmRvbF8yLmZpbC50X3QuU2lyaXVzLjJbLDE6Nl0pKQ0KDQpkYXQuZG9sXzIuZmlsLnRfdC5TaXJpdXMuNCA9IGRhdC5kb2xfMi5maWwudF90LlNpcml1cy4zICU+JSBmaWx0ZXIgKHN1bV9vZl9yb3dzID4gMCkNCg0KZGltKGRhdC5kb2xfMi5maWwudF90LlNpcml1cy40KQ0KIyA1NDAgICA4DQoNCnpPVFVzLlNpcml1cyA9IGRhdC5kb2xfMi5maWwudF90LlNpcml1cy40JHZhcmlhYmxlDQoNCmRhdC5kb2xfMi5maWwudF90LlNpcml1cy41ID0gZGF0LmRvbF8yLmZpbC50X3QuU2lyaXVzLjRbLC1jKDcsOCldDQpyb3duYW1lcyhkYXQuZG9sXzIuZmlsLnRfdC5TaXJpdXMuNSkgPSBkYXQuZG9sXzIuZmlsLnRfdC5TaXJpdXMuNCR2YXJpYWJsZQ0KDQphdHRyaWJ1dGVzLmRvbF9yZHBfZmlsLjMuU2lyaXVzID0gYXR0cmlidXRlcy5kb2xfcmRwX2ZpbC4zIFt6T1RVcy5TaXJpdXMsLTFdDQoNCiMjIHRoZXkgbmVlZCB0byBiZWNvbWUgbWF0cml4IGZpbGVzIHRvIHdvcmsgd2l0aCBwaHlsb3NlcQ0Kd2hhbGVfb3R1X25tLlNpcml1cyA9IGFzLm1hdHJpeChzYXBwbHkoZGF0LmRvbF8yLmZpbC50X3QuU2lyaXVzLjUsIGFzLm51bWVyaWMpKSANCndoYWxlX3RheF9jbS5TaXJpdXMgPSBhcy5tYXRyaXgoc2FwcGx5KGF0dHJpYnV0ZXMuZG9sX3JkcF9maWwuMy5TaXJpdXMsIGFzLmNoYXJhY3RlcikpDQoNCkRvbEJsb3dfdmFyLmZpbC5TaXJpdXMkU3BlY2llcyA8LSBhcy5jaGFyYWN0ZXIoRG9sQmxvd192YXIuZmlsLlNpcml1cyRTcGVjaWVzKQ0KRG9sQmxvd192YXIuZmlsLlNpcml1cyREb2xwaGluIDwtIGFzLmNoYXJhY3RlcihEb2xCbG93X3Zhci5maWwuU2lyaXVzJERvbHBoaW4pDQoNCiMjIGNvbnZlcnQgZmlsZXMgaW50byBwaHlsb3NlcSBvYmplY3QgdHlwZXMNCk9UVS5TaXJpdXMgPSBvdHVfdGFibGUod2hhbGVfb3R1X25tLlNpcml1cywgdGF4YV9hcmVfcm93cyA9IFRSVUUpDQpUQVguU2lyaXVzID0gdGF4X3RhYmxlKHdoYWxlX3RheF9jbS5TaXJpdXMpDQp3aGFsZWRhdGEuU2lyaXVzID0gc2FtcGxlX2RhdGEoZGF0YS5mcmFtZShEb2xCbG93X3Zhci5maWwuU2lyaXVzLCBzdHJpbmdzQXNGYWN0b3JzID0gRikpDQoNCiMjIG1lcmdlIHRoZSBmaWxlcyB3aXRoIHBoeWxvc2VxDQpEb2xwaGluLlNpcml1cyA9IHBoeWxvc2VxKE9UVS5TaXJpdXMsIFRBWC5TaXJpdXMsIHdoYWxlZGF0YS5TaXJpdXMpDQoNCkRvbHBoaW4uU2lyaXVzLjIgPC0gbWV0aG9kczo6YXMocGh5bG9zZXE6Om90dV90YWJsZShEb2xwaGluLlNpcml1cyksICJtYXRyaXgiKQ0KDQppZiAocGh5bG9zZXE6OnRheGFfYXJlX3Jvd3MoRG9scGhpbi5TaXJpdXMpKSB7IERvbHBoaW4uU2lyaXVzLjIgPC0gdChEb2xwaGluLlNpcml1cy4yKSB9DQoNCnNwZWNudW1iZXIoRG9scGhpbi5TaXJpdXMuMikNCg0KI1JhcmVmYWN0aW9uIGN1cnZlcw0KcC5TaXJpdXMgPSBnZ3JhcmUoRG9scGhpbi5TaXJpdXMsIHN0ZXAgPSAxMDAsIGNvbG9yID0gIkRvbHBoaW4iLCBzZSA9IEZBTFNFKQ0KDQpwLlNpcml1c18yID0gcC5TaXJpdXMgKyBmYWNldF93cmFwKH5TcGVjaWVzKQ0KDQpnZ3NhdmUoInJhcmVmYWN0aW9uLmN1cnZlcy5TaXJpdXMuanBnIiwgcGxvdCA9IHAuU2lyaXVzXzIgLCBkZXZpY2UgPSAnanBnJywgd2lkdGggPSAxNjgsIGhlaWdodCA9IDEzMCwgdW5pdHMgPSAibW0iLA0KICAgICAgIGRwaSA9IDMwMCwgbGltaXRzaXplID0gVFJVRSkNCg0KIyMjIyMjIyMjIyMjIyMjIyMjIyMjIyMjIyMNCiMjR2V0IGdvb2QncyBjb3ZlcmFnZQ0KIyMgbmVlZCBzYW1wbGVzIGFzIHJvd3MNCnQuT1RVLnRhYmxlLlNpcml1cyA9IHQob3R1X3RhYmxlKERvbHBoaW4uU2lyaXVzKSkgIyB0cmFuc3Bvc2UgdGhlIHRhYmxlDQpzYW1wbGVfZGF0YShEb2xwaGluLlNpcml1cykNCg0KI2RldnRvb2xzOjppbnN0YWxsX2dpdGh1YigiamZxMy9Rc1J1dGlscyIpDQpsaWJyYXJ5KFFzUnV0aWxzKQ0KDQpnb29kcy5TaXJpdXMgPSBnb29kcyhvdHVfdGFibGUodC5PVFUudGFibGUuU2lyaXVzKSkNCg0KdmFyLlNpcml1cyA9IHNhbXBsZV9kYXRhKERvbHBoaW4uU2lyaXVzKQ0KZ29vZHNfdmFyLlNpcml1cyA9IGNiaW5kKGdvb2RzLlNpcml1cywgIHZhci5TaXJpdXMpDQoNCmdvb2RzLlNpcml1cw0KDQptZWFuKGdvb2RzLlNpcml1cyRnb29kcykNCg0Kc2QoZ29vZHMuU2lyaXVzJGdvb2RzKQ0KYGBgDQoNCiMjIyMjIyMjIyMjIyMjIyMjIyMjIyMjIyMjIyMjIyMjDQojIyNTcXVlYWsNCmBgYHtyfQ0KIyMjU3F1ZWFrDQoNCiNkYXQuZG9sXzIuZmlsLnRfdCwgRG9sQmxvd192YXIuZmlsLjIsIGF0dHJpYnV0ZXMuZG9sX3JkcF9maWwuMw0KI25hbWVzKGRhdC5kb2xfMi5maWwudF90KQ0KI0RvbEJsb3dfdmFyLmZpbC4yJERvbHBoaW4NCg0KZGF0LmRvbF8yLmZpbC50X3QuU3F1ZWFrID0gZGF0LmRvbF8yLmZpbC50X3RbLGMoOTI6OTYpXQ0KRG9sQmxvd192YXIuZmlsLlNxdWVhayA9IERvbEJsb3dfdmFyLmZpbC4yW2MoOTI6OTYpLF0NCnJvd25hbWVzKERvbEJsb3dfdmFyLmZpbC5TcXVlYWspID0gRG9sQmxvd192YXIuZmlsLlNxdWVhayREb2xwaGluDQoNCmRhdC5kb2xfMi5maWwudF90LlNxdWVhay4yID0gZGF0LmRvbF8yLmZpbC50X3QuU3F1ZWFrDQpkYXQuZG9sXzIuZmlsLnRfdC5TcXVlYWsuMiR2YXJpYWJsZSA9IHJvd25hbWVzKGRhdC5kb2xfMi5maWwudF90LlNxdWVhay4yKQ0KDQpkYXQuZG9sXzIuZmlsLnRfdC5TcXVlYWsuMyA9IGRhdC5kb2xfMi5maWwudF90LlNxdWVhay4yICU+JSANCiAgICAgICAgICAgICAgICAgICAgICAgICBtdXRhdGUgKHN1bV9vZl9yb3dzID0gcm93U3VtcyhkYXQuZG9sXzIuZmlsLnRfdC5TcXVlYWsuMlssMTo1XSkpDQoNCmRhdC5kb2xfMi5maWwudF90LlNxdWVhay40ID0gZGF0LmRvbF8yLmZpbC50X3QuU3F1ZWFrLjMgJT4lIGZpbHRlciAoc3VtX29mX3Jvd3MgPiAwKQ0KDQpkaW0oZGF0LmRvbF8yLmZpbC50X3QuU3F1ZWFrLjQpDQojIDU0MCAgIDgNCg0Kek9UVXMuU3F1ZWFrID0gZGF0LmRvbF8yLmZpbC50X3QuU3F1ZWFrLjQkdmFyaWFibGUNCg0KZGF0LmRvbF8yLmZpbC50X3QuU3F1ZWFrLjUgPSBkYXQuZG9sXzIuZmlsLnRfdC5TcXVlYWsuNFssLWMoNiw3KV0NCnJvd25hbWVzKGRhdC5kb2xfMi5maWwudF90LlNxdWVhay41KSA9IGRhdC5kb2xfMi5maWwudF90LlNxdWVhay40JHZhcmlhYmxlDQoNCmF0dHJpYnV0ZXMuZG9sX3JkcF9maWwuMy5TcXVlYWsgPSBhdHRyaWJ1dGVzLmRvbF9yZHBfZmlsLjMgW3pPVFVzLlNxdWVhaywtMV0NCg0KIyMgdGhleSBuZWVkIHRvIGJlY29tZSBtYXRyaXggZmlsZXMgdG8gd29yayB3aXRoIHBoeWxvc2VxDQp3aGFsZV9vdHVfbm0uU3F1ZWFrID0gYXMubWF0cml4KHNhcHBseShkYXQuZG9sXzIuZmlsLnRfdC5TcXVlYWsuNSwgYXMubnVtZXJpYykpIA0Kd2hhbGVfdGF4X2NtLlNxdWVhayA9IGFzLm1hdHJpeChzYXBwbHkoYXR0cmlidXRlcy5kb2xfcmRwX2ZpbC4zLlNxdWVhaywgYXMuY2hhcmFjdGVyKSkNCg0KRG9sQmxvd192YXIuZmlsLlNxdWVhayRTcGVjaWVzIDwtIGFzLmNoYXJhY3RlcihEb2xCbG93X3Zhci5maWwuU3F1ZWFrJFNwZWNpZXMpDQpEb2xCbG93X3Zhci5maWwuU3F1ZWFrJERvbHBoaW4gPC0gYXMuY2hhcmFjdGVyKERvbEJsb3dfdmFyLmZpbC5TcXVlYWskRG9scGhpbikNCg0KIyMgY29udmVydCBmaWxlcyBpbnRvIHBoeWxvc2VxIG9iamVjdCB0eXBlcw0KT1RVLlNxdWVhayA9IG90dV90YWJsZSh3aGFsZV9vdHVfbm0uU3F1ZWFrLCB0YXhhX2FyZV9yb3dzID0gVFJVRSkNClRBWC5TcXVlYWsgPSB0YXhfdGFibGUod2hhbGVfdGF4X2NtLlNxdWVhaykNCndoYWxlZGF0YS5TcXVlYWsgPSBzYW1wbGVfZGF0YShkYXRhLmZyYW1lKERvbEJsb3dfdmFyLmZpbC5TcXVlYWssIHN0cmluZ3NBc0ZhY3RvcnMgPSBGKSkNCg0KIyMgbWVyZ2UgdGhlIGZpbGVzIHdpdGggcGh5bG9zZXENCkRvbHBoaW4uU3F1ZWFrID0gcGh5bG9zZXEoT1RVLlNxdWVhaywgVEFYLlNxdWVhaywgd2hhbGVkYXRhLlNxdWVhaykNCg0KRG9scGhpbi5TcXVlYWsuMiA8LSBtZXRob2RzOjphcyhwaHlsb3NlcTo6b3R1X3RhYmxlKERvbHBoaW4uU3F1ZWFrKSwgIm1hdHJpeCIpDQoNCmlmIChwaHlsb3NlcTo6dGF4YV9hcmVfcm93cyhEb2xwaGluLlNxdWVhaykpIHsgRG9scGhpbi5TcXVlYWsuMiA8LSB0KERvbHBoaW4uU3F1ZWFrLjIpIH0NCg0Kc3BlY251bWJlcihEb2xwaGluLlNxdWVhay4yKQ0KDQojUmFyZWZhY3Rpb24gY3VydmVzDQpwLlNxdWVhayA9IGdncmFyZShEb2xwaGluLlNxdWVhaywgc3RlcCA9IDEwMCwgY29sb3IgPSAiRG9scGhpbiIsIHNlID0gRkFMU0UpDQoNCnAuU3F1ZWFrXzIgPSBwLlNxdWVhayArIGZhY2V0X3dyYXAoflNwZWNpZXMpDQoNCmdnc2F2ZSgicmFyZWZhY3Rpb24uY3VydmVzLlNxdWVhay5qcGciLCBwbG90ID0gcC5TcXVlYWtfMiAsIGRldmljZSA9ICdqcGcnLCB3aWR0aCA9IDE2OCwgaGVpZ2h0ID0gMTMwLCB1bml0cyA9ICJtbSIsDQogICAgICAgZHBpID0gMzAwLCBsaW1pdHNpemUgPSBUUlVFKQ0KDQojIyMjIyMjIyMjIyMjIyMjIyMjIyMjIyMjIw0KIyNHZXQgZ29vZCdzIGNvdmVyYWdlDQojIyBuZWVkIHNhbXBsZXMgYXMgcm93cw0KdC5PVFUudGFibGUuU3F1ZWFrID0gdChvdHVfdGFibGUoRG9scGhpbi5TcXVlYWspKSAjIHRyYW5zcG9zZSB0aGUgdGFibGUNCnNhbXBsZV9kYXRhKERvbHBoaW4uU3F1ZWFrKQ0KDQojZGV2dG9vbHM6Omluc3RhbGxfZ2l0aHViKCJqZnEzL1FzUnV0aWxzIikNCmxpYnJhcnkoUXNSdXRpbHMpDQoNCmdvb2RzLlNxdWVhayA9IGdvb2RzKG90dV90YWJsZSh0Lk9UVS50YWJsZS5TcXVlYWspKQ0KDQp2YXIuU3F1ZWFrID0gc2FtcGxlX2RhdGEoRG9scGhpbi5TcXVlYWspDQpnb29kc192YXIuU3F1ZWFrID0gY2JpbmQoZ29vZHMuU3F1ZWFrLCAgdmFyLlNxdWVhaykNCg0KZ29vZHMuU3F1ZWFrDQoNCm1lYW4oZ29vZHMuU3F1ZWFrJGdvb2RzKQ0KDQpzZChnb29kcy5TcXVlYWskZ29vZHMpDQpgYGANCg0KIyMjIyMjIyMjIyMjIyMjIyMjIyMjIyMjIyMjIyMjIyMNCiMjI1N0YXJidWNrDQpgYGB7cn0NCiMjI1N0YXJidWNrDQoNCiNkYXQuZG9sXzIuZmlsLnRfdCwgRG9sQmxvd192YXIuZmlsLjIsIGF0dHJpYnV0ZXMuZG9sX3JkcF9maWwuMw0KI25hbWVzKGRhdC5kb2xfMi5maWwudF90KQ0KI0RvbEJsb3dfdmFyLmZpbC4yJERvbHBoaW4NCg0KZGF0LmRvbF8yLmZpbC50X3QuU3RhcmJ1Y2sgPSBkYXQuZG9sXzIuZmlsLnRfdFssYyg5NzoxMDIpXQ0KRG9sQmxvd192YXIuZmlsLlN0YXJidWNrID0gRG9sQmxvd192YXIuZmlsLjJbYyg5NzoxMDIpLF0NCnJvd25hbWVzKERvbEJsb3dfdmFyLmZpbC5TdGFyYnVjaykgPSBEb2xCbG93X3Zhci5maWwuU3RhcmJ1Y2skRG9scGhpbg0KDQpkYXQuZG9sXzIuZmlsLnRfdC5TdGFyYnVjay4yID0gZGF0LmRvbF8yLmZpbC50X3QuU3RhcmJ1Y2sNCmRhdC5kb2xfMi5maWwudF90LlN0YXJidWNrLjIkdmFyaWFibGUgPSByb3duYW1lcyhkYXQuZG9sXzIuZmlsLnRfdC5TdGFyYnVjay4yKQ0KDQpkYXQuZG9sXzIuZmlsLnRfdC5TdGFyYnVjay4zID0gZGF0LmRvbF8yLmZpbC50X3QuU3RhcmJ1Y2suMiAlPiUgDQogICAgICAgICAgICAgICAgICAgICAgICAgbXV0YXRlIChzdW1fb2Zfcm93cyA9IHJvd1N1bXMoZGF0LmRvbF8yLmZpbC50X3QuU3RhcmJ1Y2suMlssMTo2XSkpDQoNCmRhdC5kb2xfMi5maWwudF90LlN0YXJidWNrLjQgPSBkYXQuZG9sXzIuZmlsLnRfdC5TdGFyYnVjay4zICU+JSBmaWx0ZXIgKHN1bV9vZl9yb3dzID4gMCkNCg0KZGltKGRhdC5kb2xfMi5maWwudF90LlN0YXJidWNrLjQpDQojIDU0MCAgIDgNCg0Kek9UVXMuU3RhcmJ1Y2sgPSBkYXQuZG9sXzIuZmlsLnRfdC5TdGFyYnVjay40JHZhcmlhYmxlDQoNCmRhdC5kb2xfMi5maWwudF90LlN0YXJidWNrLjUgPSBkYXQuZG9sXzIuZmlsLnRfdC5TdGFyYnVjay40WywtYyg3LDgpXQ0Kcm93bmFtZXMoZGF0LmRvbF8yLmZpbC50X3QuU3RhcmJ1Y2suNSkgPSBkYXQuZG9sXzIuZmlsLnRfdC5TdGFyYnVjay40JHZhcmlhYmxlDQoNCmF0dHJpYnV0ZXMuZG9sX3JkcF9maWwuMy5TdGFyYnVjayA9IGF0dHJpYnV0ZXMuZG9sX3JkcF9maWwuMyBbek9UVXMuU3RhcmJ1Y2ssLTFdDQoNCiMjIHRoZXkgbmVlZCB0byBiZWNvbWUgbWF0cml4IGZpbGVzIHRvIHdvcmsgd2l0aCBwaHlsb3NlcQ0Kd2hhbGVfb3R1X25tLlN0YXJidWNrID0gYXMubWF0cml4KHNhcHBseShkYXQuZG9sXzIuZmlsLnRfdC5TdGFyYnVjay41LCBhcy5udW1lcmljKSkgDQp3aGFsZV90YXhfY20uU3RhcmJ1Y2sgPSBhcy5tYXRyaXgoc2FwcGx5KGF0dHJpYnV0ZXMuZG9sX3JkcF9maWwuMy5TdGFyYnVjaywgYXMuY2hhcmFjdGVyKSkNCg0KRG9sQmxvd192YXIuZmlsLlN0YXJidWNrJFNwZWNpZXMgPC0gYXMuY2hhcmFjdGVyKERvbEJsb3dfdmFyLmZpbC5TdGFyYnVjayRTcGVjaWVzKQ0KRG9sQmxvd192YXIuZmlsLlN0YXJidWNrJERvbHBoaW4gPC0gYXMuY2hhcmFjdGVyKERvbEJsb3dfdmFyLmZpbC5TdGFyYnVjayREb2xwaGluKQ0KDQojIyBjb252ZXJ0IGZpbGVzIGludG8gcGh5bG9zZXEgb2JqZWN0IHR5cGVzDQpPVFUuU3RhcmJ1Y2sgPSBvdHVfdGFibGUod2hhbGVfb3R1X25tLlN0YXJidWNrLCB0YXhhX2FyZV9yb3dzID0gVFJVRSkNClRBWC5TdGFyYnVjayA9IHRheF90YWJsZSh3aGFsZV90YXhfY20uU3RhcmJ1Y2spDQp3aGFsZWRhdGEuU3RhcmJ1Y2sgPSBzYW1wbGVfZGF0YShkYXRhLmZyYW1lKERvbEJsb3dfdmFyLmZpbC5TdGFyYnVjaywgc3RyaW5nc0FzRmFjdG9ycyA9IEYpKQ0KDQojIyBtZXJnZSB0aGUgZmlsZXMgd2l0aCBwaHlsb3NlcQ0KRG9scGhpbi5TdGFyYnVjayA9IHBoeWxvc2VxKE9UVS5TdGFyYnVjaywgVEFYLlN0YXJidWNrLCB3aGFsZWRhdGEuU3RhcmJ1Y2spDQoNCkRvbHBoaW4uU3RhcmJ1Y2suMiA8LSBtZXRob2RzOjphcyhwaHlsb3NlcTo6b3R1X3RhYmxlKERvbHBoaW4uU3RhcmJ1Y2spLCAibWF0cml4IikNCg0KaWYgKHBoeWxvc2VxOjp0YXhhX2FyZV9yb3dzKERvbHBoaW4uU3RhcmJ1Y2spKSB7IERvbHBoaW4uU3RhcmJ1Y2suMiA8LSB0KERvbHBoaW4uU3RhcmJ1Y2suMikgfQ0KDQpzcGVjbnVtYmVyKERvbHBoaW4uU3RhcmJ1Y2suMikNCg0KI1JhcmVmYWN0aW9uIGN1cnZlcw0KcC5TdGFyYnVjayA9IGdncmFyZShEb2xwaGluLlN0YXJidWNrLCBzdGVwID0gMTAwLCBjb2xvciA9ICJEb2xwaGluIiwgc2UgPSBGQUxTRSkNCg0KcC5TdGFyYnVja18yID0gcC5TdGFyYnVjayArIGZhY2V0X3dyYXAoflNwZWNpZXMpDQoNCmdnc2F2ZSgicmFyZWZhY3Rpb24uY3VydmVzLlN0YXJidWNrLmpwZyIsIHBsb3QgPSBwLlN0YXJidWNrXzIgLCBkZXZpY2UgPSAnanBnJywgd2lkdGggPSAxNjgsIGhlaWdodCA9IDEzMCwgdW5pdHMgPSAibW0iLA0KICAgICAgIGRwaSA9IDMwMCwgbGltaXRzaXplID0gVFJVRSkNCg0KIyMjIyMjIyMjIyMjIyMjIyMjIyMjIyMjIyMNCiMjR2V0IGdvb2QncyBjb3ZlcmFnZQ0KIyMgbmVlZCBzYW1wbGVzIGFzIHJvd3MNCnQuT1RVLnRhYmxlLlN0YXJidWNrID0gdChvdHVfdGFibGUoRG9scGhpbi5TdGFyYnVjaykpICMgdHJhbnNwb3NlIHRoZSB0YWJsZQ0Kc2FtcGxlX2RhdGEoRG9scGhpbi5TdGFyYnVjaykNCg0KI2RldnRvb2xzOjppbnN0YWxsX2dpdGh1YigiamZxMy9Rc1J1dGlscyIpDQpsaWJyYXJ5KFFzUnV0aWxzKQ0KDQpnb29kcy5TdGFyYnVjayA9IGdvb2RzKG90dV90YWJsZSh0Lk9UVS50YWJsZS5TdGFyYnVjaykpDQoNCnZhci5TdGFyYnVjayA9IHNhbXBsZV9kYXRhKERvbHBoaW4uU3RhcmJ1Y2spDQpnb29kc192YXIuU3RhcmJ1Y2sgPSBjYmluZChnb29kcy5TdGFyYnVjaywgIHZhci5TdGFyYnVjaykNCg0KZ29vZHMuU3RhcmJ1Y2sNCg0KbWVhbihnb29kcy5TdGFyYnVjayRnb29kcykNCg0Kc2QoZ29vZHMuU3RhcmJ1Y2skZ29vZHMpDQpgYGANCg0KIyMjIyMjIyMjIyMjIyMjIyMjIyMjIyMjIyMjIyMjIyMNCiMjI0dlbW1hDQpgYGB7cn0NCiMjI0dlbW1hDQoNCiNkYXQuZG9sXzIuZmlsLnRfdCwgRG9sQmxvd192YXIuZmlsLjIsIGF0dHJpYnV0ZXMuZG9sX3JkcF9maWwuMw0KI25hbWVzKGRhdC5kb2xfMi5maWwudF90KQ0KI0RvbEJsb3dfdmFyLmZpbC4yJERvbHBoaW4NCg0KZGF0LmRvbF8yLmZpbC50X3QuR2VtbWEgPSBkYXQuZG9sXzIuZmlsLnRfdFssYygzODo0MildDQpEb2xCbG93X3Zhci5maWwuR2VtbWEgPSBEb2xCbG93X3Zhci5maWwuMltjKDM4OjQyKSxdDQpyb3duYW1lcyhEb2xCbG93X3Zhci5maWwuR2VtbWEpID0gRG9sQmxvd192YXIuZmlsLkdlbW1hJERvbHBoaW4NCg0KZGF0LmRvbF8yLmZpbC50X3QuR2VtbWEuMiA9IGRhdC5kb2xfMi5maWwudF90LkdlbW1hDQpkYXQuZG9sXzIuZmlsLnRfdC5HZW1tYS4yJHZhcmlhYmxlID0gcm93bmFtZXMoZGF0LmRvbF8yLmZpbC50X3QuR2VtbWEuMikNCg0KZGF0LmRvbF8yLmZpbC50X3QuR2VtbWEuMyA9IGRhdC5kb2xfMi5maWwudF90LkdlbW1hLjIgJT4lIA0KICAgICAgICAgICAgICAgICAgICAgICAgIG11dGF0ZSAoc3VtX29mX3Jvd3MgPSByb3dTdW1zKGRhdC5kb2xfMi5maWwudF90LkdlbW1hLjJbLDE6NV0pKQ0KDQpkYXQuZG9sXzIuZmlsLnRfdC5HZW1tYS40ID0gZGF0LmRvbF8yLmZpbC50X3QuR2VtbWEuMyAlPiUgZmlsdGVyIChzdW1fb2Zfcm93cyA+IDApDQoNCmRpbShkYXQuZG9sXzIuZmlsLnRfdC5HZW1tYS40KQ0KIyA1NDAgICA4DQoNCnpPVFVzLkdlbW1hID0gZGF0LmRvbF8yLmZpbC50X3QuR2VtbWEuNCR2YXJpYWJsZQ0KDQpkYXQuZG9sXzIuZmlsLnRfdC5HZW1tYS41ID0gZGF0LmRvbF8yLmZpbC50X3QuR2VtbWEuNFssLWMoNiw3KV0NCnJvd25hbWVzKGRhdC5kb2xfMi5maWwudF90LkdlbW1hLjUpID0gZGF0LmRvbF8yLmZpbC50X3QuR2VtbWEuNCR2YXJpYWJsZQ0KDQphdHRyaWJ1dGVzLmRvbF9yZHBfZmlsLjMuR2VtbWEgPSBhdHRyaWJ1dGVzLmRvbF9yZHBfZmlsLjMgW3pPVFVzLkdlbW1hLC0xXQ0KDQojIyB0aGV5IG5lZWQgdG8gYmVjb21lIG1hdHJpeCBmaWxlcyB0byB3b3JrIHdpdGggcGh5bG9zZXENCndoYWxlX290dV9ubS5HZW1tYSA9IGFzLm1hdHJpeChzYXBwbHkoZGF0LmRvbF8yLmZpbC50X3QuR2VtbWEuNSwgYXMubnVtZXJpYykpIA0Kd2hhbGVfdGF4X2NtLkdlbW1hID0gYXMubWF0cml4KHNhcHBseShhdHRyaWJ1dGVzLmRvbF9yZHBfZmlsLjMuR2VtbWEsIGFzLmNoYXJhY3RlcikpDQoNCkRvbEJsb3dfdmFyLmZpbC5HZW1tYSRTcGVjaWVzIDwtIGFzLmNoYXJhY3RlcihEb2xCbG93X3Zhci5maWwuR2VtbWEkU3BlY2llcykNCkRvbEJsb3dfdmFyLmZpbC5HZW1tYSREb2xwaGluIDwtIGFzLmNoYXJhY3RlcihEb2xCbG93X3Zhci5maWwuR2VtbWEkRG9scGhpbikNCg0KIyMgY29udmVydCBmaWxlcyBpbnRvIHBoeWxvc2VxIG9iamVjdCB0eXBlcw0KT1RVLkdlbW1hID0gb3R1X3RhYmxlKHdoYWxlX290dV9ubS5HZW1tYSwgdGF4YV9hcmVfcm93cyA9IFRSVUUpDQpUQVguR2VtbWEgPSB0YXhfdGFibGUod2hhbGVfdGF4X2NtLkdlbW1hKQ0Kd2hhbGVkYXRhLkdlbW1hID0gc2FtcGxlX2RhdGEoZGF0YS5mcmFtZShEb2xCbG93X3Zhci5maWwuR2VtbWEsIHN0cmluZ3NBc0ZhY3RvcnMgPSBGKSkNCg0KIyMgbWVyZ2UgdGhlIGZpbGVzIHdpdGggcGh5bG9zZXENCkRvbHBoaW4uR2VtbWEgPSBwaHlsb3NlcShPVFUuR2VtbWEsIFRBWC5HZW1tYSwgd2hhbGVkYXRhLkdlbW1hKQ0KDQpEb2xwaGluLkdlbW1hLjIgPC0gbWV0aG9kczo6YXMocGh5bG9zZXE6Om90dV90YWJsZShEb2xwaGluLkdlbW1hKSwgIm1hdHJpeCIpDQoNCmlmIChwaHlsb3NlcTo6dGF4YV9hcmVfcm93cyhEb2xwaGluLkdlbW1hKSkgeyBEb2xwaGluLkdlbW1hLjIgPC0gdChEb2xwaGluLkdlbW1hLjIpIH0NCg0Kc3BlY251bWJlcihEb2xwaGluLkdlbW1hLjIpDQoNCiNSYXJlZmFjdGlvbiBjdXJ2ZXMNCnAuR2VtbWEgPSBnZ3JhcmUoRG9scGhpbi5HZW1tYSwgc3RlcCA9IDEwMCwgY29sb3IgPSAiRG9scGhpbiIsIHNlID0gRkFMU0UpDQoNCnAuR2VtbWFfMiA9IHAuR2VtbWEgKyBmYWNldF93cmFwKH5TcGVjaWVzKQ0KDQpnZ3NhdmUoInJhcmVmYWN0aW9uLmN1cnZlcy5HZW1tYS5qcGciLCBwbG90ID0gcC5HZW1tYV8yICwgZGV2aWNlID0gJ2pwZycsIHdpZHRoID0gMTY4LCBoZWlnaHQgPSAxMzAsIHVuaXRzID0gIm1tIiwNCiAgICAgICBkcGkgPSAzMDAsIGxpbWl0c2l6ZSA9IFRSVUUpDQoNCiMjIyMjIyMjIyMjIyMjIyMjIyMjIyMjIyMjDQojI0dldCBnb29kJ3MgY292ZXJhZ2UNCiMjIG5lZWQgc2FtcGxlcyBhcyByb3dzDQp0Lk9UVS50YWJsZS5HZW1tYSA9IHQob3R1X3RhYmxlKERvbHBoaW4uR2VtbWEpKSAjIHRyYW5zcG9zZSB0aGUgdGFibGUNCnNhbXBsZV9kYXRhKERvbHBoaW4uR2VtbWEpDQoNCiNkZXZ0b29sczo6aW5zdGFsbF9naXRodWIoImpmcTMvUXNSdXRpbHMiKQ0KbGlicmFyeShRc1J1dGlscykNCg0KZ29vZHMuR2VtbWEgPSBnb29kcyhvdHVfdGFibGUodC5PVFUudGFibGUuR2VtbWEpKQ0KDQp2YXIuR2VtbWEgPSBzYW1wbGVfZGF0YShEb2xwaGluLkdlbW1hKQ0KZ29vZHNfdmFyLkdlbW1hID0gY2JpbmQoZ29vZHMuR2VtbWEsICB2YXIuR2VtbWEpDQoNCmdvb2RzLkdlbW1hDQoNCm1lYW4oZ29vZHMuR2VtbWEkZ29vZHMpDQoNCnNkKGdvb2RzLkdlbW1hJGdvb2RzKQ0KYGBgDQoNCiMjIyMjIyMjIyMjIyMjIyMjIyMjIyMjIyMjIyMjIyMjDQojIyNIb3dpZQ0KYGBge3J9DQojIyNIb3dpZQ0KDQojZGF0LmRvbF8yLmZpbC50X3QsIERvbEJsb3dfdmFyLmZpbC4yLCBhdHRyaWJ1dGVzLmRvbF9yZHBfZmlsLjMNCiNuYW1lcyhkYXQuZG9sXzIuZmlsLnRfdCkNCiNEb2xCbG93X3Zhci5maWwuMiREb2xwaGluDQoNCmRhdC5kb2xfMi5maWwudF90Lkhvd2llID0gZGF0LmRvbF8yLmZpbC50X3RbLGMoNDM6NTEpXQ0KRG9sQmxvd192YXIuZmlsLkhvd2llID0gRG9sQmxvd192YXIuZmlsLjJbYyg0Mzo1MSksXQ0Kcm93bmFtZXMoRG9sQmxvd192YXIuZmlsLkhvd2llKSA9IERvbEJsb3dfdmFyLmZpbC5Ib3dpZSREb2xwaGluDQoNCmRhdC5kb2xfMi5maWwudF90Lkhvd2llLjIgPSBkYXQuZG9sXzIuZmlsLnRfdC5Ib3dpZQ0KZGF0LmRvbF8yLmZpbC50X3QuSG93aWUuMiR2YXJpYWJsZSA9IHJvd25hbWVzKGRhdC5kb2xfMi5maWwudF90Lkhvd2llLjIpDQoNCmRhdC5kb2xfMi5maWwudF90Lkhvd2llLjMgPSBkYXQuZG9sXzIuZmlsLnRfdC5Ib3dpZS4yICU+JSANCiAgICAgICAgICAgICAgICAgICAgICAgICBtdXRhdGUgKHN1bV9vZl9yb3dzID0gcm93U3VtcyhkYXQuZG9sXzIuZmlsLnRfdC5Ib3dpZS4yWywxOjldKSkNCg0KZGF0LmRvbF8yLmZpbC50X3QuSG93aWUuNCA9IGRhdC5kb2xfMi5maWwudF90Lkhvd2llLjMgJT4lIGZpbHRlciAoc3VtX29mX3Jvd3MgPiAwKQ0KDQpkaW0oZGF0LmRvbF8yLmZpbC50X3QuSG93aWUuNCkNCiMgNTQwICAgOA0KDQp6T1RVcy5Ib3dpZSA9IGRhdC5kb2xfMi5maWwudF90Lkhvd2llLjQkdmFyaWFibGUNCg0KZGF0LmRvbF8yLmZpbC50X3QuSG93aWUuNSA9IGRhdC5kb2xfMi5maWwudF90Lkhvd2llLjRbLC1jKDEwOjExKV0NCnJvd25hbWVzKGRhdC5kb2xfMi5maWwudF90Lkhvd2llLjUpID0gZGF0LmRvbF8yLmZpbC50X3QuSG93aWUuNCR2YXJpYWJsZQ0KDQphdHRyaWJ1dGVzLmRvbF9yZHBfZmlsLjMuSG93aWUgPSBhdHRyaWJ1dGVzLmRvbF9yZHBfZmlsLjMgW3pPVFVzLkhvd2llLC0xXQ0KDQojIyB0aGV5IG5lZWQgdG8gYmVjb21lIG1hdHJpeCBmaWxlcyB0byB3b3JrIHdpdGggcGh5bG9zZXENCndoYWxlX290dV9ubS5Ib3dpZSA9IGFzLm1hdHJpeChzYXBwbHkoZGF0LmRvbF8yLmZpbC50X3QuSG93aWUuNSwgYXMubnVtZXJpYykpIA0Kd2hhbGVfdGF4X2NtLkhvd2llID0gYXMubWF0cml4KHNhcHBseShhdHRyaWJ1dGVzLmRvbF9yZHBfZmlsLjMuSG93aWUsIGFzLmNoYXJhY3RlcikpDQoNCkRvbEJsb3dfdmFyLmZpbC5Ib3dpZSRTcGVjaWVzIDwtIGFzLmNoYXJhY3RlcihEb2xCbG93X3Zhci5maWwuSG93aWUkU3BlY2llcykNCkRvbEJsb3dfdmFyLmZpbC5Ib3dpZSREb2xwaGluIDwtIGFzLmNoYXJhY3RlcihEb2xCbG93X3Zhci5maWwuSG93aWUkRG9scGhpbikNCg0KIyMgY29udmVydCBmaWxlcyBpbnRvIHBoeWxvc2VxIG9iamVjdCB0eXBlcw0KT1RVLkhvd2llID0gb3R1X3RhYmxlKHdoYWxlX290dV9ubS5Ib3dpZSwgdGF4YV9hcmVfcm93cyA9IFRSVUUpDQpUQVguSG93aWUgPSB0YXhfdGFibGUod2hhbGVfdGF4X2NtLkhvd2llKQ0Kd2hhbGVkYXRhLkhvd2llID0gc2FtcGxlX2RhdGEoZGF0YS5mcmFtZShEb2xCbG93X3Zhci5maWwuSG93aWUsIHN0cmluZ3NBc0ZhY3RvcnMgPSBGKSkNCg0KIyMgbWVyZ2UgdGhlIGZpbGVzIHdpdGggcGh5bG9zZXENCkRvbHBoaW4uSG93aWUgPSBwaHlsb3NlcShPVFUuSG93aWUsIFRBWC5Ib3dpZSwgd2hhbGVkYXRhLkhvd2llKQ0KDQpEb2xwaGluLkhvd2llLjIgPC0gbWV0aG9kczo6YXMocGh5bG9zZXE6Om90dV90YWJsZShEb2xwaGluLkhvd2llKSwgIm1hdHJpeCIpDQoNCmlmIChwaHlsb3NlcTo6dGF4YV9hcmVfcm93cyhEb2xwaGluLkhvd2llKSkgeyBEb2xwaGluLkhvd2llLjIgPC0gdChEb2xwaGluLkhvd2llLjIpIH0NCg0Kc3BlY251bWJlcihEb2xwaGluLkhvd2llLjIpDQoNCiNSYXJlZmFjdGlvbiBjdXJ2ZXMNCnAuSG93aWUgPSBnZ3JhcmUoRG9scGhpbi5Ib3dpZSwgc3RlcCA9IDEwMCwgY29sb3IgPSAiRG9scGhpbiIsIHNlID0gRkFMU0UpDQoNCnAuSG93aWVfMiA9IHAuSG93aWUgKyBmYWNldF93cmFwKH5TcGVjaWVzKQ0KDQpnZ3NhdmUoInJhcmVmYWN0aW9uLmN1cnZlcy5Ib3dpZS5qcGciLCBwbG90ID0gcC5Ib3dpZV8yICwgZGV2aWNlID0gJ2pwZycsIHdpZHRoID0gMTY4LCBoZWlnaHQgPSAxMzAsIHVuaXRzID0gIm1tIiwNCiAgICAgICBkcGkgPSAzMDAsIGxpbWl0c2l6ZSA9IFRSVUUpDQoNCiMjIyMjIyMjIyMjIyMjIyMjIyMjIyMjIyMjDQojI0dldCBnb29kJ3MgY292ZXJhZ2UNCiMjIG5lZWQgc2FtcGxlcyBhcyByb3dzDQp0Lk9UVS50YWJsZS5Ib3dpZSA9IHQob3R1X3RhYmxlKERvbHBoaW4uSG93aWUpKSAjIHRyYW5zcG9zZSB0aGUgdGFibGUNCnNhbXBsZV9kYXRhKERvbHBoaW4uSG93aWUpDQoNCiNkZXZ0b29sczo6aW5zdGFsbF9naXRodWIoImpmcTMvUXNSdXRpbHMiKQ0KbGlicmFyeShRc1J1dGlscykNCg0KZ29vZHMuSG93aWUgPSBnb29kcyhvdHVfdGFibGUodC5PVFUudGFibGUuSG93aWUpKQ0KDQp2YXIuSG93aWUgPSBzYW1wbGVfZGF0YShEb2xwaGluLkhvd2llKQ0KZ29vZHNfdmFyLkhvd2llID0gY2JpbmQoZ29vZHMuSG93aWUsICB2YXIuSG93aWUpDQoNCmdvb2RzLkhvd2llDQoNCm1lYW4oZ29vZHMuSG93aWUkZ29vZHMpDQoNCnNkKGdvb2RzLkhvd2llJGdvb2RzKQ0KYGBgDQoNCiMjIyMjIyMjIyMjIyMjIyMjIyMjIyMjIyMjIyMjIyMjDQojIyNOdWRnZWUNCmBgYHtyfQ0KIyMjTnVkZ2VlDQoNCiNkYXQuZG9sXzIuZmlsLnRfdCwgRG9sQmxvd192YXIuZmlsLjIsIGF0dHJpYnV0ZXMuZG9sX3JkcF9maWwuMw0KI25hbWVzKGRhdC5kb2xfMi5maWwudF90KQ0KI0RvbEJsb3dfdmFyLmZpbC4yJERvbHBoaW4NCg0KZGF0LmRvbF8yLmZpbC50X3QuTnVkZ2VlID0gZGF0LmRvbF8yLmZpbC50X3RbLGMoNjM6NzEpXQ0KRG9sQmxvd192YXIuZmlsLk51ZGdlZSA9IERvbEJsb3dfdmFyLmZpbC4yW2MoMTY6NzEpLF0NCnJvd25hbWVzKERvbEJsb3dfdmFyLmZpbC5OdWRnZWUpID0gRG9sQmxvd192YXIuZmlsLk51ZGdlZSREb2xwaGluDQoNCmRhdC5kb2xfMi5maWwudF90Lk51ZGdlZS4yID0gZGF0LmRvbF8yLmZpbC50X3QuTnVkZ2VlDQpkYXQuZG9sXzIuZmlsLnRfdC5OdWRnZWUuMiR2YXJpYWJsZSA9IHJvd25hbWVzKGRhdC5kb2xfMi5maWwudF90Lk51ZGdlZS4yKQ0KDQpkYXQuZG9sXzIuZmlsLnRfdC5OdWRnZWUuMyA9IGRhdC5kb2xfMi5maWwudF90Lk51ZGdlZS4yICU+JSANCiAgICAgICAgICAgICAgICAgICAgICAgICBtdXRhdGUgKHN1bV9vZl9yb3dzID0gcm93U3VtcyhkYXQuZG9sXzIuZmlsLnRfdC5OdWRnZWUuMlssMTo5XSkpDQoNCmRhdC5kb2xfMi5maWwudF90Lk51ZGdlZS40ID0gZGF0LmRvbF8yLmZpbC50X3QuTnVkZ2VlLjMgJT4lIGZpbHRlciAoc3VtX29mX3Jvd3MgPiAwKQ0KDQpkaW0oZGF0LmRvbF8yLmZpbC50X3QuTnVkZ2VlLjQpDQojIDU0MCAgIDgNCg0Kek9UVXMuTnVkZ2VlID0gZGF0LmRvbF8yLmZpbC50X3QuTnVkZ2VlLjQkdmFyaWFibGUNCg0KZGF0LmRvbF8yLmZpbC50X3QuTnVkZ2VlLjUgPSBkYXQuZG9sXzIuZmlsLnRfdC5OdWRnZWUuNFssLWMoMTAsMTEpXQ0Kcm93bmFtZXMoZGF0LmRvbF8yLmZpbC50X3QuTnVkZ2VlLjUpID0gZGF0LmRvbF8yLmZpbC50X3QuTnVkZ2VlLjQkdmFyaWFibGUNCg0KYXR0cmlidXRlcy5kb2xfcmRwX2ZpbC4zLk51ZGdlZSA9IGF0dHJpYnV0ZXMuZG9sX3JkcF9maWwuMyBbek9UVXMuTnVkZ2VlLC0xXQ0KDQojIyB0aGV5IG5lZWQgdG8gYmVjb21lIG1hdHJpeCBmaWxlcyB0byB3b3JrIHdpdGggcGh5bG9zZXENCndoYWxlX290dV9ubS5OdWRnZWUgPSBhcy5tYXRyaXgoc2FwcGx5KGRhdC5kb2xfMi5maWwudF90Lk51ZGdlZS41LCBhcy5udW1lcmljKSkgDQp3aGFsZV90YXhfY20uTnVkZ2VlID0gYXMubWF0cml4KHNhcHBseShhdHRyaWJ1dGVzLmRvbF9yZHBfZmlsLjMuTnVkZ2VlLCBhcy5jaGFyYWN0ZXIpKQ0KDQpEb2xCbG93X3Zhci5maWwuTnVkZ2VlJFNwZWNpZXMgPC0gYXMuY2hhcmFjdGVyKERvbEJsb3dfdmFyLmZpbC5OdWRnZWUkU3BlY2llcykNCkRvbEJsb3dfdmFyLmZpbC5OdWRnZWUkRG9scGhpbiA8LSBhcy5jaGFyYWN0ZXIoRG9sQmxvd192YXIuZmlsLk51ZGdlZSREb2xwaGluKQ0KDQojIyBjb252ZXJ0IGZpbGVzIGludG8gcGh5bG9zZXEgb2JqZWN0IHR5cGVzDQpPVFUuTnVkZ2VlID0gb3R1X3RhYmxlKHdoYWxlX290dV9ubS5OdWRnZWUsIHRheGFfYXJlX3Jvd3MgPSBUUlVFKQ0KVEFYLk51ZGdlZSA9IHRheF90YWJsZSh3aGFsZV90YXhfY20uTnVkZ2VlKQ0Kd2hhbGVkYXRhLk51ZGdlZSA9IHNhbXBsZV9kYXRhKGRhdGEuZnJhbWUoRG9sQmxvd192YXIuZmlsLk51ZGdlZSwgc3RyaW5nc0FzRmFjdG9ycyA9IEYpKQ0KDQojIyBtZXJnZSB0aGUgZmlsZXMgd2l0aCBwaHlsb3NlcQ0KRG9scGhpbi5OdWRnZWUgPSBwaHlsb3NlcShPVFUuTnVkZ2VlLCBUQVguTnVkZ2VlLCB3aGFsZWRhdGEuTnVkZ2VlKQ0KDQpEb2xwaGluLk51ZGdlZS4yIDwtIG1ldGhvZHM6OmFzKHBoeWxvc2VxOjpvdHVfdGFibGUoRG9scGhpbi5OdWRnZWUpLCAibWF0cml4IikNCg0KaWYgKHBoeWxvc2VxOjp0YXhhX2FyZV9yb3dzKERvbHBoaW4uTnVkZ2VlKSkgeyBEb2xwaGluLk51ZGdlZS4yIDwtIHQoRG9scGhpbi5OdWRnZWUuMikgfQ0KDQpzcGVjbnVtYmVyKERvbHBoaW4uTnVkZ2VlLjIpDQoNCiNSYXJlZmFjdGlvbiBjdXJ2ZXMNCnAuTnVkZ2VlID0gZ2dyYXJlKERvbHBoaW4uTnVkZ2VlLCBzdGVwID0gMTAwLCBjb2xvciA9ICJEb2xwaGluIiwgc2UgPSBGQUxTRSkNCg0KcC5OdWRnZWVfMiA9IHAuTnVkZ2VlICsgZmFjZXRfd3JhcCh+U3BlY2llcykNCg0KZ2dzYXZlKCJyYXJlZmFjdGlvbi5jdXJ2ZXMuTnVkZ2VlLmpwZyIsIHBsb3QgPSBwLk51ZGdlZV8yICwgZGV2aWNlID0gJ2pwZycsIHdpZHRoID0gMTY4LCBoZWlnaHQgPSAxMzAsIHVuaXRzID0gIm1tIiwNCiAgICAgICBkcGkgPSAzMDAsIGxpbWl0c2l6ZSA9IFRSVUUpDQoNCiMjIyMjIyMjIyMjIyMjIyMjIyMjIyMjIyMjDQojI0dldCBnb29kJ3MgY292ZXJhZ2UNCiMjIG5lZWQgc2FtcGxlcyBhcyByb3dzDQp0Lk9UVS50YWJsZS5OdWRnZWUgPSB0KG90dV90YWJsZShEb2xwaGluLk51ZGdlZSkpICMgdHJhbnNwb3NlIHRoZSB0YWJsZQ0Kc2FtcGxlX2RhdGEoRG9scGhpbi5OdWRnZWUpDQoNCiNkZXZ0b29sczo6aW5zdGFsbF9naXRodWIoImpmcTMvUXNSdXRpbHMiKQ0KbGlicmFyeShRc1J1dGlscykNCg0KZ29vZHMuTnVkZ2VlID0gZ29vZHMob3R1X3RhYmxlKHQuT1RVLnRhYmxlLk51ZGdlZSkpDQoNCnZhci5OdWRnZWUgPSBzYW1wbGVfZGF0YShEb2xwaGluLk51ZGdlZSkNCmdvb2RzX3Zhci5OdWRnZWUgPSBjYmluZChnb29kcy5OdWRnZWUsICB2YXIuTnVkZ2VlKQ0KDQpnb29kcy5OdWRnZWUNCg0KbWVhbihnb29kcy5OdWRnZWUkZ29vZHMpDQoNCnNkKGdvb2RzLk51ZGdlZSRnb29kcykNCmBgYA0KDQojIyMjIyMjIyMjIyMjIyMjIyMjIyMjIyMjIyMjIyMjIw0KIyMjU3RlbGxhDQpgYGB7cn0NCiMjI1N0ZWxsYQ0KDQojZGF0LmRvbF8yLmZpbC50X3QsIERvbEJsb3dfdmFyLmZpbC4yLCBhdHRyaWJ1dGVzLmRvbF9yZHBfZmlsLjMNCiNuYW1lcyhkYXQuZG9sXzIuZmlsLnRfdCkNCiNEb2xCbG93X3Zhci5maWwuMiREb2xwaGluDQoNCmRhdC5kb2xfMi5maWwudF90LlN0ZWxsYSA9IGRhdC5kb2xfMi5maWwudF90WyxjKDEwMzoxMTEpXQ0KRG9sQmxvd192YXIuZmlsLlN0ZWxsYSA9IERvbEJsb3dfdmFyLmZpbC4yW2MoMTAzOjExMSksXQ0Kcm93bmFtZXMoRG9sQmxvd192YXIuZmlsLlN0ZWxsYSkgPSBEb2xCbG93X3Zhci5maWwuU3RlbGxhJERvbHBoaW4NCg0KZGF0LmRvbF8yLmZpbC50X3QuU3RlbGxhLjIgPSBkYXQuZG9sXzIuZmlsLnRfdC5TdGVsbGENCmRhdC5kb2xfMi5maWwudF90LlN0ZWxsYS4yJHZhcmlhYmxlID0gcm93bmFtZXMoZGF0LmRvbF8yLmZpbC50X3QuU3RlbGxhLjIpDQoNCmRhdC5kb2xfMi5maWwudF90LlN0ZWxsYS4zID0gZGF0LmRvbF8yLmZpbC50X3QuU3RlbGxhLjIgJT4lIA0KICAgICAgICAgICAgICAgICAgICAgICAgIG11dGF0ZSAoc3VtX29mX3Jvd3MgPSByb3dTdW1zKGRhdC5kb2xfMi5maWwudF90LlN0ZWxsYS4yWywxOjldKSkNCg0KZGF0LmRvbF8yLmZpbC50X3QuU3RlbGxhLjQgPSBkYXQuZG9sXzIuZmlsLnRfdC5TdGVsbGEuMyAlPiUgZmlsdGVyIChzdW1fb2Zfcm93cyA+IDApDQoNCmRpbShkYXQuZG9sXzIuZmlsLnRfdC5TdGVsbGEuNCkNCiMgNTQwICAgOA0KDQp6T1RVcy5TdGVsbGEgPSBkYXQuZG9sXzIuZmlsLnRfdC5TdGVsbGEuNCR2YXJpYWJsZQ0KDQpkYXQuZG9sXzIuZmlsLnRfdC5TdGVsbGEuNSA9IGRhdC5kb2xfMi5maWwudF90LlN0ZWxsYS40WywtYygxMCwxMSldDQpyb3duYW1lcyhkYXQuZG9sXzIuZmlsLnRfdC5TdGVsbGEuNSkgPSBkYXQuZG9sXzIuZmlsLnRfdC5TdGVsbGEuNCR2YXJpYWJsZQ0KDQphdHRyaWJ1dGVzLmRvbF9yZHBfZmlsLjMuU3RlbGxhID0gYXR0cmlidXRlcy5kb2xfcmRwX2ZpbC4zIFt6T1RVcy5TdGVsbGEsLTFdDQoNCiMjIHRoZXkgbmVlZCB0byBiZWNvbWUgbWF0cml4IGZpbGVzIHRvIHdvcmsgd2l0aCBwaHlsb3NlcQ0Kd2hhbGVfb3R1X25tLlN0ZWxsYSA9IGFzLm1hdHJpeChzYXBwbHkoZGF0LmRvbF8yLmZpbC50X3QuU3RlbGxhLjUsIGFzLm51bWVyaWMpKSANCndoYWxlX3RheF9jbS5TdGVsbGEgPSBhcy5tYXRyaXgoc2FwcGx5KGF0dHJpYnV0ZXMuZG9sX3JkcF9maWwuMy5TdGVsbGEsIGFzLmNoYXJhY3RlcikpDQoNCkRvbEJsb3dfdmFyLmZpbC5TdGVsbGEkU3BlY2llcyA8LSBhcy5jaGFyYWN0ZXIoRG9sQmxvd192YXIuZmlsLlN0ZWxsYSRTcGVjaWVzKQ0KRG9sQmxvd192YXIuZmlsLlN0ZWxsYSREb2xwaGluIDwtIGFzLmNoYXJhY3RlcihEb2xCbG93X3Zhci5maWwuU3RlbGxhJERvbHBoaW4pDQoNCiMjIGNvbnZlcnQgZmlsZXMgaW50byBwaHlsb3NlcSBvYmplY3QgdHlwZXMNCk9UVS5TdGVsbGEgPSBvdHVfdGFibGUod2hhbGVfb3R1X25tLlN0ZWxsYSwgdGF4YV9hcmVfcm93cyA9IFRSVUUpDQpUQVguU3RlbGxhID0gdGF4X3RhYmxlKHdoYWxlX3RheF9jbS5TdGVsbGEpDQp3aGFsZWRhdGEuU3RlbGxhID0gc2FtcGxlX2RhdGEoZGF0YS5mcmFtZShEb2xCbG93X3Zhci5maWwuU3RlbGxhLCBzdHJpbmdzQXNGYWN0b3JzID0gRikpDQoNCiMjIG1lcmdlIHRoZSBmaWxlcyB3aXRoIHBoeWxvc2VxDQpEb2xwaGluLlN0ZWxsYSA9IHBoeWxvc2VxKE9UVS5TdGVsbGEsIFRBWC5TdGVsbGEsIHdoYWxlZGF0YS5TdGVsbGEpDQoNCkRvbHBoaW4uU3RlbGxhLjIgPC0gbWV0aG9kczo6YXMocGh5bG9zZXE6Om90dV90YWJsZShEb2xwaGluLlN0ZWxsYSksICJtYXRyaXgiKQ0KDQppZiAocGh5bG9zZXE6OnRheGFfYXJlX3Jvd3MoRG9scGhpbi5TdGVsbGEpKSB7IERvbHBoaW4uU3RlbGxhLjIgPC0gdChEb2xwaGluLlN0ZWxsYS4yKSB9DQoNCnNwZWNudW1iZXIoRG9scGhpbi5TdGVsbGEuMikNCg0KI1JhcmVmYWN0aW9uIGN1cnZlcw0KcC5TdGVsbGEgPSBnZ3JhcmUoRG9scGhpbi5TdGVsbGEsIHN0ZXAgPSAxMDAsIGNvbG9yID0gIkRvbHBoaW4iLCBzZSA9IEZBTFNFKQ0KDQpwLlN0ZWxsYV8yID0gcC5TdGVsbGEgKyBmYWNldF93cmFwKH5TcGVjaWVzKQ0KDQpnZ3NhdmUoInJhcmVmYWN0aW9uLmN1cnZlcy5TdGVsbGEuanBnIiwgcGxvdCA9IHAuU3RlbGxhXzIgLCBkZXZpY2UgPSAnanBnJywgd2lkdGggPSAxNjgsIGhlaWdodCA9IDEzMCwgdW5pdHMgPSAibW0iLA0KICAgICAgIGRwaSA9IDMwMCwgbGltaXRzaXplID0gVFJVRSkNCg0KIyMjIyMjIyMjIyMjIyMjIyMjIyMjIyMjIyMNCiMjR2V0IGdvb2QncyBjb3ZlcmFnZQ0KIyMgbmVlZCBzYW1wbGVzIGFzIHJvd3MNCnQuT1RVLnRhYmxlLlN0ZWxsYSA9IHQob3R1X3RhYmxlKERvbHBoaW4uU3RlbGxhKSkgIyB0cmFuc3Bvc2UgdGhlIHRhYmxlDQpzYW1wbGVfZGF0YShEb2xwaGluLlN0ZWxsYSkNCg0KI2RldnRvb2xzOjppbnN0YWxsX2dpdGh1YigiamZxMy9Rc1J1dGlscyIpDQpsaWJyYXJ5KFFzUnV0aWxzKQ0KDQpnb29kcy5TdGVsbGEgPSBnb29kcyhvdHVfdGFibGUodC5PVFUudGFibGUuU3RlbGxhKSkNCg0KdmFyLlN0ZWxsYSA9IHNhbXBsZV9kYXRhKERvbHBoaW4uU3RlbGxhKQ0KZ29vZHNfdmFyLlN0ZWxsYSA9IGNiaW5kKGdvb2RzLlN0ZWxsYSwgIHZhci5TdGVsbGEpDQoNCmdvb2RzLlN0ZWxsYQ0KDQptZWFuKGdvb2RzLlN0ZWxsYSRnb29kcykNCg0Kc2QoZ29vZHMuU3RlbGxhJGdvb2RzKQ0KYGBgDQoNCiMjIyMjIyMjIyMjIyMjIyMjIyMjIyMjIyMjIyMjIyMjDQojIyNCYXkNCmBgYHtyfQ0KIyMjQmF5DQoNCiNkYXQuZG9sXzIuZmlsLnRfdCwgRG9sQmxvd192YXIuZmlsLjIsIGF0dHJpYnV0ZXMuZG9sX3JkcF9maWwuMw0KI25hbWVzKGRhdC5kb2xfMi5maWwudF90KQ0KI0RvbEJsb3dfdmFyLmZpbC4yJERvbHBoaW4NCg0KZGF0LmRvbF8yLmZpbC50X3QuQmF5ID0gZGF0LmRvbF8yLmZpbC50X3RbLGMoMTo2KV0NCkRvbEJsb3dfdmFyLmZpbC5CYXkgPSBEb2xCbG93X3Zhci5maWwuMltjKDE6NiksXQ0Kcm93bmFtZXMoRG9sQmxvd192YXIuZmlsLkJheSkgPSBEb2xCbG93X3Zhci5maWwuQmF5JERvbHBoaW4NCg0KZGF0LmRvbF8yLmZpbC50X3QuQmF5LjIgPSBkYXQuZG9sXzIuZmlsLnRfdC5CYXkNCmRhdC5kb2xfMi5maWwudF90LkJheS4yJHZhcmlhYmxlID0gcm93bmFtZXMoZGF0LmRvbF8yLmZpbC50X3QuQmF5LjIpDQoNCmRhdC5kb2xfMi5maWwudF90LkJheS4zID0gZGF0LmRvbF8yLmZpbC50X3QuQmF5LjIgJT4lIA0KICAgICAgICAgICAgICAgICAgICAgICAgIG11dGF0ZSAoc3VtX29mX3Jvd3MgPSByb3dTdW1zKGRhdC5kb2xfMi5maWwudF90LkJheS4yWywxOjZdKSkNCg0KZGF0LmRvbF8yLmZpbC50X3QuQmF5LjQgPSBkYXQuZG9sXzIuZmlsLnRfdC5CYXkuMyAlPiUgZmlsdGVyIChzdW1fb2Zfcm93cyA+IDApDQoNCmRpbShkYXQuZG9sXzIuZmlsLnRfdC5CYXkuNCkNCiMgNTQwICAgOA0KDQp6T1RVcy5CYXkgPSBkYXQuZG9sXzIuZmlsLnRfdC5CYXkuNCR2YXJpYWJsZQ0KDQpkYXQuZG9sXzIuZmlsLnRfdC5CYXkuNSA9IGRhdC5kb2xfMi5maWwudF90LkJheS40WywtYyg3LDgpXQ0Kcm93bmFtZXMoZGF0LmRvbF8yLmZpbC50X3QuQmF5LjUpID0gZGF0LmRvbF8yLmZpbC50X3QuQmF5LjQkdmFyaWFibGUNCg0KYXR0cmlidXRlcy5kb2xfcmRwX2ZpbC4zLkJheSA9IGF0dHJpYnV0ZXMuZG9sX3JkcF9maWwuMyBbek9UVXMuQmF5LC0xXQ0KDQojIyB0aGV5IG5lZWQgdG8gYmVjb21lIG1hdHJpeCBmaWxlcyB0byB3b3JrIHdpdGggcGh5bG9zZXENCndoYWxlX290dV9ubS5CYXkgPSBhcy5tYXRyaXgoc2FwcGx5KGRhdC5kb2xfMi5maWwudF90LkJheS41LCBhcy5udW1lcmljKSkgDQp3aGFsZV90YXhfY20uQmF5ID0gYXMubWF0cml4KHNhcHBseShhdHRyaWJ1dGVzLmRvbF9yZHBfZmlsLjMuQmF5LCBhcy5jaGFyYWN0ZXIpKQ0KDQpEb2xCbG93X3Zhci5maWwuQmF5JFNwZWNpZXMgPC0gYXMuY2hhcmFjdGVyKERvbEJsb3dfdmFyLmZpbC5CYXkkU3BlY2llcykNCkRvbEJsb3dfdmFyLmZpbC5CYXkkRG9scGhpbiA8LSBhcy5jaGFyYWN0ZXIoRG9sQmxvd192YXIuZmlsLkJheSREb2xwaGluKQ0KDQojIyBjb252ZXJ0IGZpbGVzIGludG8gcGh5bG9zZXEgb2JqZWN0IHR5cGVzDQpPVFUuQmF5ID0gb3R1X3RhYmxlKHdoYWxlX290dV9ubS5CYXksIHRheGFfYXJlX3Jvd3MgPSBUUlVFKQ0KVEFYLkJheSA9IHRheF90YWJsZSh3aGFsZV90YXhfY20uQmF5KQ0Kd2hhbGVkYXRhLkJheSA9IHNhbXBsZV9kYXRhKGRhdGEuZnJhbWUoRG9sQmxvd192YXIuZmlsLkJheSwgc3RyaW5nc0FzRmFjdG9ycyA9IEYpKQ0KDQojIyBtZXJnZSB0aGUgZmlsZXMgd2l0aCBwaHlsb3NlcQ0KRG9scGhpbi5CYXkgPSBwaHlsb3NlcShPVFUuQmF5LCBUQVguQmF5LCB3aGFsZWRhdGEuQmF5KQ0KDQpEb2xwaGluLkJheS4yIDwtIG1ldGhvZHM6OmFzKHBoeWxvc2VxOjpvdHVfdGFibGUoRG9scGhpbi5CYXkpLCAibWF0cml4IikNCg0KaWYgKHBoeWxvc2VxOjp0YXhhX2FyZV9yb3dzKERvbHBoaW4uQmF5KSkgeyBEb2xwaGluLkJheS4yIDwtIHQoRG9scGhpbi5CYXkuMikgfQ0KDQpzcGVjbnVtYmVyKERvbHBoaW4uQmF5LjIpDQoNCiNSYXJlZmFjdGlvbiBjdXJ2ZXMNCnAuQmF5ID0gZ2dyYXJlKERvbHBoaW4uQmF5LCBzdGVwID0gMTAwLCBjb2xvciA9ICJEb2xwaGluIiwgc2UgPSBGQUxTRSkNCg0KcC5CYXlfMiA9IHAuQmF5ICsgZmFjZXRfd3JhcCh+U3BlY2llcykNCg0KZ2dzYXZlKCJyYXJlZmFjdGlvbi5jdXJ2ZXMuQmF5LmpwZyIsIHBsb3QgPSBwLkJheV8yICwgZGV2aWNlID0gJ2pwZycsIHdpZHRoID0gMTY4LCBoZWlnaHQgPSAxMzAsIHVuaXRzID0gIm1tIiwNCiAgICAgICBkcGkgPSAzMDAsIGxpbWl0c2l6ZSA9IFRSVUUpDQoNCiMjIyMjIyMjIyMjIyMjIyMjIyMjIyMjIyMjDQojI0dldCBnb29kJ3MgY292ZXJhZ2UNCiMjIG5lZWQgc2FtcGxlcyBhcyByb3dzDQp0Lk9UVS50YWJsZS5CYXkgPSB0KG90dV90YWJsZShEb2xwaGluLkJheSkpICMgdHJhbnNwb3NlIHRoZSB0YWJsZQ0Kc2FtcGxlX2RhdGEoRG9scGhpbi5CYXkpDQoNCiNkZXZ0b29sczo6aW5zdGFsbF9naXRodWIoImpmcTMvUXNSdXRpbHMiKQ0KbGlicmFyeShRc1J1dGlscykNCg0KZ29vZHMuQmF5ID0gZ29vZHMob3R1X3RhYmxlKHQuT1RVLnRhYmxlLkJheSkpDQoNCnZhci5CYXkgPSBzYW1wbGVfZGF0YShEb2xwaGluLkJheSkNCmdvb2RzX3Zhci5CYXkgPSBjYmluZChnb29kcy5CYXksICB2YXIuQmF5KQ0KDQpnb29kcy5CYXkNCg0KbWVhbihnb29kcy5CYXkkZ29vZHMpDQoNCnNkKGdvb2RzLkJheSRnb29kcykNCmBgYA0KDQojIyMjIyMjIyMjIyMjIyMjIyMjIyMjIyMjIyMjIyMjIw0KIyMjQmVhY2gNCmBgYHtyfQ0KIyMjQmVhY2gNCg0KI2RhdC5kb2xfMi5maWwudF90LCBEb2xCbG93X3Zhci5maWwuMiwgYXR0cmlidXRlcy5kb2xfcmRwX2ZpbC4zDQojbmFtZXMoZGF0LmRvbF8yLmZpbC50X3QpDQojRG9sQmxvd192YXIuZmlsLjIkRG9scGhpbg0KDQpkYXQuZG9sXzIuZmlsLnRfdC5CZWFjaCA9IGRhdC5kb2xfMi5maWwudF90WyxjKDc6MTUpXQ0KRG9sQmxvd192YXIuZmlsLkJlYWNoID0gRG9sQmxvd192YXIuZmlsLjJbYyg3OjE1KSxdDQpyb3duYW1lcyhEb2xCbG93X3Zhci5maWwuQmVhY2gpID0gRG9sQmxvd192YXIuZmlsLkJlYWNoJERvbHBoaW4NCg0KZGF0LmRvbF8yLmZpbC50X3QuQmVhY2guMiA9IGRhdC5kb2xfMi5maWwudF90LkJlYWNoDQpkYXQuZG9sXzIuZmlsLnRfdC5CZWFjaC4yJHZhcmlhYmxlID0gcm93bmFtZXMoZGF0LmRvbF8yLmZpbC50X3QuQmVhY2guMikNCg0KZGF0LmRvbF8yLmZpbC50X3QuQmVhY2guMyA9IGRhdC5kb2xfMi5maWwudF90LkJlYWNoLjIgJT4lIA0KICAgICAgICAgICAgICAgICAgICAgICAgIG11dGF0ZSAoc3VtX29mX3Jvd3MgPSByb3dTdW1zKGRhdC5kb2xfMi5maWwudF90LkJlYWNoLjJbLDE6OV0pKQ0KDQpkYXQuZG9sXzIuZmlsLnRfdC5CZWFjaC40ID0gZGF0LmRvbF8yLmZpbC50X3QuQmVhY2guMyAlPiUgZmlsdGVyIChzdW1fb2Zfcm93cyA+IDApDQoNCmRpbShkYXQuZG9sXzIuZmlsLnRfdC5CZWFjaC40KQ0KIyA1NDAgICA4DQoNCnpPVFVzLkJlYWNoID0gZGF0LmRvbF8yLmZpbC50X3QuQmVhY2guNCR2YXJpYWJsZQ0KDQpkYXQuZG9sXzIuZmlsLnRfdC5CZWFjaC41ID0gZGF0LmRvbF8yLmZpbC50X3QuQmVhY2guNFssLWMoMTAsMTEpXQ0Kcm93bmFtZXMoZGF0LmRvbF8yLmZpbC50X3QuQmVhY2guNSkgPSBkYXQuZG9sXzIuZmlsLnRfdC5CZWFjaC40JHZhcmlhYmxlDQoNCmF0dHJpYnV0ZXMuZG9sX3JkcF9maWwuMy5CZWFjaCA9IGF0dHJpYnV0ZXMuZG9sX3JkcF9maWwuMyBbek9UVXMuQmVhY2gsLTFdDQoNCiMjIHRoZXkgbmVlZCB0byBiZWNvbWUgbWF0cml4IGZpbGVzIHRvIHdvcmsgd2l0aCBwaHlsb3NlcQ0Kd2hhbGVfb3R1X25tLkJlYWNoID0gYXMubWF0cml4KHNhcHBseShkYXQuZG9sXzIuZmlsLnRfdC5CZWFjaC41LCBhcy5udW1lcmljKSkgDQp3aGFsZV90YXhfY20uQmVhY2ggPSBhcy5tYXRyaXgoc2FwcGx5KGF0dHJpYnV0ZXMuZG9sX3JkcF9maWwuMy5CZWFjaCwgYXMuY2hhcmFjdGVyKSkNCg0KRG9sQmxvd192YXIuZmlsLkJlYWNoJFNwZWNpZXMgPC0gYXMuY2hhcmFjdGVyKERvbEJsb3dfdmFyLmZpbC5CZWFjaCRTcGVjaWVzKQ0KRG9sQmxvd192YXIuZmlsLkJlYWNoJERvbHBoaW4gPC0gYXMuY2hhcmFjdGVyKERvbEJsb3dfdmFyLmZpbC5CZWFjaCREb2xwaGluKQ0KDQojIyBjb252ZXJ0IGZpbGVzIGludG8gcGh5bG9zZXEgb2JqZWN0IHR5cGVzDQpPVFUuQmVhY2ggPSBvdHVfdGFibGUod2hhbGVfb3R1X25tLkJlYWNoLCB0YXhhX2FyZV9yb3dzID0gVFJVRSkNClRBWC5CZWFjaCA9IHRheF90YWJsZSh3aGFsZV90YXhfY20uQmVhY2gpDQp3aGFsZWRhdGEuQmVhY2ggPSBzYW1wbGVfZGF0YShkYXRhLmZyYW1lKERvbEJsb3dfdmFyLmZpbC5CZWFjaCwgc3RyaW5nc0FzRmFjdG9ycyA9IEYpKQ0KDQojIyBtZXJnZSB0aGUgZmlsZXMgd2l0aCBwaHlsb3NlcQ0KRG9scGhpbi5CZWFjaCA9IHBoeWxvc2VxKE9UVS5CZWFjaCwgVEFYLkJlYWNoLCB3aGFsZWRhdGEuQmVhY2gpDQoNCkRvbHBoaW4uQmVhY2guMiA8LSBtZXRob2RzOjphcyhwaHlsb3NlcTo6b3R1X3RhYmxlKERvbHBoaW4uQmVhY2gpLCAibWF0cml4IikNCg0KaWYgKHBoeWxvc2VxOjp0YXhhX2FyZV9yb3dzKERvbHBoaW4uQmVhY2gpKSB7IERvbHBoaW4uQmVhY2guMiA8LSB0KERvbHBoaW4uQmVhY2guMikgfQ0KDQpzcGVjbnVtYmVyKERvbHBoaW4uQmVhY2guMikNCg0KI1JhcmVmYWN0aW9uIGN1cnZlcw0KcC5CZWFjaCA9IGdncmFyZShEb2xwaGluLkJlYWNoLCBzdGVwID0gMTAwLCBjb2xvciA9ICJEb2xwaGluIiwgc2UgPSBGQUxTRSkNCg0KcC5CZWFjaF8yID0gcC5CZWFjaCArIGZhY2V0X3dyYXAoflNwZWNpZXMpDQoNCmdnc2F2ZSgicmFyZWZhY3Rpb24uY3VydmVzLkJlYWNoLmpwZyIsIHBsb3QgPSBwLkJlYWNoXzIgLCBkZXZpY2UgPSAnanBnJywgd2lkdGggPSAxNjgsIGhlaWdodCA9IDEzMCwgdW5pdHMgPSAibW0iLA0KICAgICAgIGRwaSA9IDMwMCwgbGltaXRzaXplID0gVFJVRSkNCg0KIyMjIyMjIyMjIyMjIyMjIyMjIyMjIyMjIyMNCiMjR2V0IGdvb2QncyBjb3ZlcmFnZQ0KIyMgbmVlZCBzYW1wbGVzIGFzIHJvd3MNCnQuT1RVLnRhYmxlLkJlYWNoID0gdChvdHVfdGFibGUoRG9scGhpbi5CZWFjaCkpICMgdHJhbnNwb3NlIHRoZSB0YWJsZQ0Kc2FtcGxlX2RhdGEoRG9scGhpbi5CZWFjaCkNCg0KI2RldnRvb2xzOjppbnN0YWxsX2dpdGh1YigiamZxMy9Rc1J1dGlscyIpDQpsaWJyYXJ5KFFzUnV0aWxzKQ0KDQpnb29kcy5CZWFjaCA9IGdvb2RzKG90dV90YWJsZSh0Lk9UVS50YWJsZS5CZWFjaCkpDQoNCnZhci5CZWFjaCA9IHNhbXBsZV9kYXRhKERvbHBoaW4uQmVhY2gpDQpnb29kc192YXIuQmVhY2ggPSBjYmluZChnb29kcy5CZWFjaCwgIHZhci5CZWFjaCkNCg0KZ29vZHMuQmVhY2gNCg0KbWVhbihnb29kcy5CZWFjaCRnb29kcykNCg0Kc2QoZ29vZHMuQmVhY2gkZ29vZHMpDQpgYGANCg0KIyMjIyMjIyMjIyMjIyMjIyMjIyMjIyMjIyMjIyMjIyMNCiMjI1FWQw0KYGBge3J9DQojIyNRVkMNCg0KI2RhdC5kb2xfMi5maWwudF90LCBEb2xCbG93X3Zhci5maWwuMiwgYXR0cmlidXRlcy5kb2xfcmRwX2ZpbC4zDQojbmFtZXMoZGF0LmRvbF8yLmZpbC50X3QpDQojRG9sQmxvd192YXIuZmlsLjIkRG9scGhpbg0KDQpkYXQuZG9sXzIuZmlsLnRfdC5RVkMgPSBkYXQuZG9sXzIuZmlsLnRfdFssYyg3Mjo3NCldDQpEb2xCbG93X3Zhci5maWwuUVZDID0gRG9sQmxvd192YXIuZmlsLjJbYyg3Mjo3NCksXQ0Kcm93bmFtZXMoRG9sQmxvd192YXIuZmlsLlFWQykgPSBEb2xCbG93X3Zhci5maWwuUVZDJERvbHBoaW4NCg0KZGF0LmRvbF8yLmZpbC50X3QuUVZDLjIgPSBkYXQuZG9sXzIuZmlsLnRfdC5RVkMNCmRhdC5kb2xfMi5maWwudF90LlFWQy4yJHZhcmlhYmxlID0gcm93bmFtZXMoZGF0LmRvbF8yLmZpbC50X3QuUVZDLjIpDQoNCmRhdC5kb2xfMi5maWwudF90LlFWQy4zID0gZGF0LmRvbF8yLmZpbC50X3QuUVZDLjIgJT4lIA0KICAgICAgICAgICAgICAgICAgICAgICAgIG11dGF0ZSAoc3VtX29mX3Jvd3MgPSByb3dTdW1zKGRhdC5kb2xfMi5maWwudF90LlFWQy4yWywxOjNdKSkNCg0KZGF0LmRvbF8yLmZpbC50X3QuUVZDLjQgPSBkYXQuZG9sXzIuZmlsLnRfdC5RVkMuMyAlPiUgZmlsdGVyIChzdW1fb2Zfcm93cyA+IDApDQoNCmRpbShkYXQuZG9sXzIuZmlsLnRfdC5RVkMuNCkNCiMgNTQwICAgOA0KDQp6T1RVcy5RVkMgPSBkYXQuZG9sXzIuZmlsLnRfdC5RVkMuNCR2YXJpYWJsZQ0KDQpkYXQuZG9sXzIuZmlsLnRfdC5RVkMuNSA9IGRhdC5kb2xfMi5maWwudF90LlFWQy40WywtYyg0LDUpXQ0Kcm93bmFtZXMoZGF0LmRvbF8yLmZpbC50X3QuUVZDLjUpID0gZGF0LmRvbF8yLmZpbC50X3QuUVZDLjQkdmFyaWFibGUNCg0KYXR0cmlidXRlcy5kb2xfcmRwX2ZpbC4zLlFWQyA9IGF0dHJpYnV0ZXMuZG9sX3JkcF9maWwuMyBbek9UVXMuUVZDLC0xXQ0KDQojIyB0aGV5IG5lZWQgdG8gYmVjb21lIG1hdHJpeCBmaWxlcyB0byB3b3JrIHdpdGggcGh5bG9zZXENCndoYWxlX290dV9ubS5RVkMgPSBhcy5tYXRyaXgoc2FwcGx5KGRhdC5kb2xfMi5maWwudF90LlFWQy41LCBhcy5udW1lcmljKSkgDQp3aGFsZV90YXhfY20uUVZDID0gYXMubWF0cml4KHNhcHBseShhdHRyaWJ1dGVzLmRvbF9yZHBfZmlsLjMuUVZDLCBhcy5jaGFyYWN0ZXIpKQ0KDQpEb2xCbG93X3Zhci5maWwuUVZDJFNwZWNpZXMgPC0gYXMuY2hhcmFjdGVyKERvbEJsb3dfdmFyLmZpbC5RVkMkU3BlY2llcykNCkRvbEJsb3dfdmFyLmZpbC5RVkMkRG9scGhpbiA8LSBhcy5jaGFyYWN0ZXIoRG9sQmxvd192YXIuZmlsLlFWQyREb2xwaGluKQ0KDQojIyBjb252ZXJ0IGZpbGVzIGludG8gcGh5bG9zZXEgb2JqZWN0IHR5cGVzDQpPVFUuUVZDID0gb3R1X3RhYmxlKHdoYWxlX290dV9ubS5RVkMsIHRheGFfYXJlX3Jvd3MgPSBUUlVFKQ0KVEFYLlFWQyA9IHRheF90YWJsZSh3aGFsZV90YXhfY20uUVZDKQ0Kd2hhbGVkYXRhLlFWQyA9IHNhbXBsZV9kYXRhKGRhdGEuZnJhbWUoRG9sQmxvd192YXIuZmlsLlFWQywgc3RyaW5nc0FzRmFjdG9ycyA9IEYpKQ0KDQojIyBtZXJnZSB0aGUgZmlsZXMgd2l0aCBwaHlsb3NlcQ0KRG9scGhpbi5RVkMgPSBwaHlsb3NlcShPVFUuUVZDLCBUQVguUVZDLCB3aGFsZWRhdGEuUVZDKQ0KDQpEb2xwaGluLlFWQy4yIDwtIG1ldGhvZHM6OmFzKHBoeWxvc2VxOjpvdHVfdGFibGUoRG9scGhpbi5RVkMpLCAibWF0cml4IikNCg0KaWYgKHBoeWxvc2VxOjp0YXhhX2FyZV9yb3dzKERvbHBoaW4uUVZDKSkgeyBEb2xwaGluLlFWQy4yIDwtIHQoRG9scGhpbi5RVkMuMikgfQ0KDQpzcGVjbnVtYmVyKERvbHBoaW4uUVZDLjIpDQoNCiNSYXJlZmFjdGlvbiBjdXJ2ZXMNCnAuUVZDID0gZ2dyYXJlKERvbHBoaW4uUVZDLCBzdGVwID0gMTAwLCBjb2xvciA9ICJEb2xwaGluIiwgc2UgPSBGQUxTRSkNCg0KcC5RVkNfMiA9IHAuUVZDICsgZmFjZXRfd3JhcCh+U3BlY2llcykNCg0KZ2dzYXZlKCJyYXJlZmFjdGlvbi5jdXJ2ZXMuUVZDLmpwZyIsIHBsb3QgPSBwLlFWQ18yICwgZGV2aWNlID0gJ2pwZycsIHdpZHRoID0gMTY4LCBoZWlnaHQgPSAxMzAsIHVuaXRzID0gIm1tIiwNCiAgICAgICBkcGkgPSAzMDAsIGxpbWl0c2l6ZSA9IFRSVUUpDQoNCiMjIyMjIyMjIyMjIyMjIyMjIyMjIyMjIyMjDQojI0dldCBnb29kJ3MgY292ZXJhZ2UNCiMjIG5lZWQgc2FtcGxlcyBhcyByb3dzDQp0Lk9UVS50YWJsZS5RVkMgPSB0KG90dV90YWJsZShEb2xwaGluLlFWQykpICMgdHJhbnNwb3NlIHRoZSB0YWJsZQ0Kc2FtcGxlX2RhdGEoRG9scGhpbi5RVkMpDQoNCiNkZXZ0b29sczo6aW5zdGFsbF9naXRodWIoImpmcTMvUXNSdXRpbHMiKQ0KbGlicmFyeShRc1J1dGlscykNCg0KZ29vZHMuUVZDID0gZ29vZHMob3R1X3RhYmxlKHQuT1RVLnRhYmxlLlFWQykpDQoNCnZhci5RVkMgPSBzYW1wbGVfZGF0YShEb2xwaGluLlFWQykNCmdvb2RzX3Zhci5RVkMgPSBjYmluZChnb29kcy5RVkMsICB2YXIuUVZDKQ0KDQpnb29kcy5RVkMNCg0KbWVhbihnb29kcy5RVkMkZ29vZHMpDQoNCnNkKGdvb2RzLlFWQyRnb29kcykNCmBgYA0KIyMjIyMjIyMjIyMjIyMjIyMjIyMjIyMjIyMjIyMjIyMjIyMjIyMjIyMjIyMjIyMjIyMjIyMjIyMjIyMjIw0KIyMjVGVzdCBpZiBtaWNyb2Jpb3RhIG9mIHdhdGVyIHNhbXBsZXMgYW5kIGRvbHBoaW4gc2FtcGxlcyBhcmUgc2lnbmlmaWNhbnRseSBkaWZmZXJlbnQgZnJvbSBlYWNob3RoZXINCg0KYGBge3J9DQojIyMjIyMjIyMjIyMjIyMjIyMjDQojIyNDcmVhdGUgek9UVSBkYXRhdGFibGUgd2l0aCBjb2xzIG9mIFNwZWNpZXMsIFguU2FtcGxlSUQgYW5kIFNhbXBsZV9vcl9Db250cm9sLjIgKGFsc28gdXNlZCBmb3IgZmlsdGVyaW5nIG9mIHdhdGVyIHpPVFVzIGluIGxhdGVyIHN0ZXApDQoNCiNNZXRhZGF0YTogRG9sQmxvd192YXIuZmlsLjINCg0KI1Byb3ZpZGUgRG9sQmxvd192YXIuZmlsLjIgd2l0aCBjb2wgJ1NhbXBsZV9vcl9Db250cm9sJywgYW5kIHJlbmFtZSBjb2wgJ0RvbHBoaW4nIHRvIFguU2FtcGxlSUQNCiNEb2xCbG93X3Zhci5maWwuMiREb2xwaGluDQoNClNhbXBsZV9vcl9Db250cm9sLjIgPC0gYXMuZGF0YS5mcmFtZShjKHJlcCgnQ29udHJvbCcsIDE1KSwgcmVwKCdUcnVlIFNhbXBsZScsIDYpLCByZXAoJ0NvbnRyb2wnLCAxMCksIHJlcCgnVHJ1ZSBTYW1wbGUnLCA4MCkpKQ0KZGltKFNhbXBsZV9vcl9Db250cm9sLjIpDQojMTExICAxDQoNCm5hbWVzKFNhbXBsZV9vcl9Db250cm9sLjIpIDwtICdTYW1wbGVfb3JfQ29udHJvbC4yJw0KDQpEb2xCbG93X3Zhci5maWwuMyA8LSBjYmluZChTYW1wbGVfb3JfQ29udHJvbC4yLCBEb2xCbG93X3Zhci5maWwuMikNCiNWaWV3KERvbEJsb3dfdmFyLmZpbC4zKQ0KDQpjb2xuYW1lcyhEb2xCbG93X3Zhci5maWwuMylbM10gPC0gJ1guU2FtcGxlSUQnDQoNCiNMZWF2ZSBpbiBjb2xzICdTYW1wbGVfb3JfQ29udHJvbC4yJyBhbmQgJ1guU2FtcGxlSUQnIG9ubHkNCkRvbEJsb3dfdmFyLmZpbC40IDwtIERvbEJsb3dfdmFyLmZpbC4zWyxjKC0yKV0NCiNWaWV3KERvbEJsb3dfdmFyLmZpbC40KQ0KDQpyb3duYW1lcyhEb2xCbG93X3Zhci5maWwuNCkgPC0gRG9sQmxvd192YXIuZmlsLjQkYw0KYGBgDQoNCmBgYHtyfQ0KIyMjTWVyZ2UgRG9sQmxvd192YXIuZmlsLjMgYW5kIGRhdC5kb2xfMi5maWwudA0KZGltKGRhdC5kb2xfMi5maWwudCkNCiMxMTEgMTk5MQ0KI1ZpZXcoZGF0LmRvbF8yLmZpbC50WywgMTk4NToxOTkxXSkNCmRhdC5kb2xfMi5maWwudC4yIDwtIGRhdC5kb2xfMi5maWwudA0KZGF0LmRvbF8yLmZpbC50LjIkWC5TYW1wbGVJRCA8LSByb3duYW1lcyhkYXQuZG9sXzIuZmlsLnQuMikNCg0KZGF0LmRvbF8yLmZpbC50LjMgPC0gbGVmdF9qb2luKERvbEJsb3dfdmFyLmZpbC4zLGRhdC5kb2xfMi5maWwudC4yICkNCmRhdC5kb2xfMi5maWwudC40IDwtIGRhdC5kb2xfMi5maWwudC4zWywtMV0NCmRpbShkYXQuZG9sXzIuZmlsLnQuNCkNCiMxMTEgMTk5Mw0KDQojIyNGaXQgdGhlIGdlbmVyYWxpc2VkIGxpbmVhciBtb2RlbHMNCnpPVFUudGVjaC5maWwgPSBtdmFidW5kKGRhdC5kb2xfMi5maWwudC40WzM6MTk5M10pDQoNCiNjcmVhdGUgdmFyaWFibGUgZm9yIGxvZyBvZiB0b3RhbCBhYnVuZGFuY2Ugb2YgT1RVJ3MgcGVyIHNhbXBsZQ0KZGF0LmRvbF8yLmZpbC50LjUgPSBkYXQuZG9sXzIuZmlsLnQuNA0KZGltKGRhdC5kb2xfMi5maWwudC41KQ0KIzExMSAxOTkzDQoNCmRhdC5kb2xfMi5maWwudC41JGxvZ1RvdGFsQWJ1bmRhbmNlID0gbG9nKGFwcGx5KGRhdC5kb2xfMi5maWwudC41WywzOjE5OTNdLDEsc3VtKSkNCg0KUHJlLmZpdDEudGVjaC5maWwgPSBtYW55Z2xtKHpPVFUudGVjaC5maWwgfiBTcGVjaWVzICsgb2Zmc2V0KGxvZ1RvdGFsQWJ1bmRhbmNlKSwgZGF0YSA9IGRhdC5kb2xfMi5maWwudC41LCBmYW1pbHk9Im5lZ2F0aXZlLmJpbm9taWFsIikNCg0KI2NoZWNrIGFzc3VtcHRpb25zDQpwbG90KFByZS5maXQxLnRlY2guZmlsKQ0KDQojVGhpcyBpcyB0aGUgYWN0dWFsIHNpZ25pZmljYW5jZSB0ZXN0ICAgICAgICAgICAgICAgDQpmaXQuMS5kb2wudGVjaC5maWwgPSBtYW55Z2xtKHpPVFUudGVjaC5maWwgfiBTcGVjaWVzICsgb2Zmc2V0KGRhdC5kb2xfMi5maWwudC41JGxvZ1RvdGFsQWJ1bmRhbmNlKSwgZGF0YSA9IGRhdC5kb2xfMi5maWwudC41KQ0KZml0LjIuZG9sLnRlY2guZmlsID0gbWFueWdsbSh6T1RVLnRlY2guZmlsIH4gb2Zmc2V0KGRhdC5kb2xfMi5maWwudC41JGxvZ1RvdGFsQWJ1bmRhbmNlKSwgZGF0YSA9IGRhdC5kb2xfMi5maWwudC41KQ0KDQojYWRqdXN0ZWQNCiNnbG9iYWxUZXN0MS5kb2wudGVjaC5maWwuYWRqdXN0ZWQud2F0ZXIgPSBhbm92YShmaXQuMS5kb2wudGVjaC5maWwsIGZpdC4yLmRvbC50ZWNoLmZpbCwgbkJvb3Q9MTAwMCwgcC51bmk9J2FkanVzdGVkJykNCg0KIyBBbmFseXNpcyBvZiBEZXZpYW5jZSBUYWJsZQ0KIyANCiMgZml0LjIuZG9sLnRlY2guZmlsOiB6T1RVLnRlY2guZmlsIH4gb2Zmc2V0KGRhdC5kb2xfMi5maWwudC41JGxvZ1RvdGFsQWJ1bmRhbmNlKQ0KIyBmaXQuMS5kb2wudGVjaC5maWw6IHpPVFUudGVjaC5maWwgfiBTcGVjaWVzICsgb2Zmc2V0KGRhdC5kb2xfMi5maWwudC41JGxvZ1RvdGFsQWJ1bmRhbmNlKQ0KIyANCiMgTXVsdGl2YXJpYXRlIHRlc3Q6DQojICAgICAgICAgICAgICAgICAgICBSZXMuRGYgRGYuZGlmZiAgIERldiBQcig+RGV2KSAgICANCiMgZml0LjIuZG9sLnRlY2guZmlsICAgIDExMCAgICAgICAgICAgICAgICAgICAgICAgICAgIA0KIyBmaXQuMS5kb2wudGVjaC5maWwgICAgMTA5ICAgICAgIDEgMjQ5ODAgICAgMC4wMDEgKioqDQojIC0tLQ0KIyBTaWduaWYuIGNvZGVzOiAgMCDigJgqKirigJkgMC4wMDEg4oCYKirigJkgMC4wMSDigJgq4oCZIDAuMDUg4oCYLuKAmSAwLjEg4oCYIOKAmSAxDQoNCnNhdmUoZ2xvYmFsVGVzdDEuZG9sLnRlY2guZmlsLmFkanVzdGVkLndhdGVyLCBmaWxlPSdnbG9iYWxUZXN0MS5kb2wudGVjaC5maWwuYWRqdXN0ZWQud2F0ZXIuUmRhdGEnKQ0KDQpnbG9iYWxUZXN0MV9zaWduaWZpY2FudC5kb2wudGVjaC5maWwuYWRqdXN0ZWQud2F0ZXIgPSBjb2xuYW1lcyhnbG9iYWxUZXN0MS5kb2wudGVjaC5maWwuYWRqdXN0ZWQud2F0ZXIkdW5pLnApW3doaWNoKGdsb2JhbFRlc3QxLmRvbC50ZWNoLmZpbC5hZGp1c3RlZC53YXRlciR1bmkucFsyLF08MC4wNSldDQpsZW5ndGgoZ2xvYmFsVGVzdDFfc2lnbmlmaWNhbnQuZG9sLnRlY2guZmlsLmFkanVzdGVkLndhdGVyKQ0KIzEyMQ0KDQpgYGANCg0KIyMjIyMjIyMjIyMjIyMjIyMjIyMjIyMjIyMjIyMjIyMjIyMjIyMjIyMjIyMjIyMjIyMjIyMjIyMjIyMjIyMjIyMjIyMNCiMjI0NyZWF0ZSBwaHlsb3NlcS1jbGFzcyBvYmplY3QgdG8gZmlsdGVyIG91dCBwb29sIHdhdGVyIGNvbnRhbWluYW50cw0KDQpgYGB7cn0NCiMjI1VzZSBEb2xCbG93X3Zhci5maWwuNCwgYXR0cmlidXRlcy5kb2xfcmRwX2ZpbC4yICYgZGF0LmRvbF90LjE5LnQuZmlsLjINCg0KIyMgVGhlc2UgZmlsZXMgbmVlZCB0byBiZWNvbWUgbWF0cml4IGZpbGVzIHRvIHdvcmsgd2l0aCBwaHlsb3NlcQ0KZG9scGhpbl9vdHVfbm0udyA9IGFzLm1hdHJpeChzYXBwbHkoZGF0LmRvbF90LjE5LnQuZmlsLjIsIGFzLm51bWVyaWMpKQ0KZG9scGhpbl90YXhfY20udyA9IGFzLm1hdHJpeChzYXBwbHkoYXR0cmlidXRlcy5kb2xfcmRwX2ZpbC4yLCBhcy5jaGFyYWN0ZXIpKQ0KDQojIyBDb252ZXJ0IGZpbGVzIGludG8gcGh5bG9zZXEgb2JqZWN0IHR5cGVzDQp6T1RVLncgPSBvdHVfdGFibGUoZG9scGhpbl9vdHVfbm0udywgdGF4YV9hcmVfcm93cyA9IFRSVUUpDQpUQVgudyA9IHRheF90YWJsZShkb2xwaGluX3RheF9jbS53KQ0KZG9scGhpbmRhdGEudyA9IHNhbXBsZV9kYXRhKGRhdGEuZnJhbWUoRG9sQmxvd192YXIuZmlsLjQsIHN0cmluZ3NBc0ZhY3RvcnMgPSBGKSkNCg0KIyMgbWVyZ2UgdGhlIGZpbGVzIHdpdGggcGh5bG9zZXENCmRvbHBoaW4xLncgPSBwaHlsb3NlcSh6T1RVLncsIFRBWC53LCBkb2xwaGluZGF0YS53KQ0KDQojIHBoeWxvc2VxLWNsYXNzIGV4cGVyaW1lbnQtbGV2ZWwgb2JqZWN0DQoNCiMgb3R1X3RhYmxlKCkgICBPVFUgVGFibGU6ICAgICAgICAgWyAxOTkxIHRheGEgYW5kIDExMSBzYW1wbGVzIF0NCiMgc2FtcGxlX2RhdGEoKSBTYW1wbGUgRGF0YTogICAgICAgWyAxMTEgc2FtcGxlcyBieSAyIHNhbXBsZSB2YXJpYWJsZXMgXQ0KIyB0YXhfdGFibGUoKSAgIFRheG9ub215IFRhYmxlOiAgICBbIDE5OTEgdGF4YSBieSA3IHRheG9ub21pYyByYW5rcyBdDQoNCmhlYWQoc2FtcGxlX2RhdGEoZG9scGhpbjEudykpDQoNCiMgUHV0IHNhbXBsZV9kYXRhIGludG8gYSBnZ3Bsb3QtZnJpZW5kbHkgZGF0YS5mcmFtZQ0KZGYudzwtIGFzLmRhdGEuZnJhbWUoc2FtcGxlX2RhdGEoZG9scGhpbjEudykpDQoNCiNDaGVjayBsaWJyYXJ5IHNpemUNCmRmLnckTGlicmFyeVNpemUgPC0gc2FtcGxlX3N1bXMoZG9scGhpbjEudykNCmRmLnc8LSBkZltvcmRlcihkZi53JExpYnJhcnlTaXplKSxdDQpkZi53JEluZGV4IDwtIHNlcShucm93KGRmLncpKQ0KZ2dwbG90KGRhdGE9ZGYsIGFlcyh4PUluZGV4LCB5PUxpYnJhcnlTaXplLCBjb2xvcj1TYW1wbGVfb3JfQ29udHJvbCkpICsgZ2VvbV9wb2ludCgpICsgZ2VvbV9qaXR0ZXIoKQ0KYGBgDQoNCiMjIyMjIyMjIyMjIyMjIyMjIyMjIyMjIyMjIyMjIyMjIyMjIyMjIyMjIw0KIyMjSWRlbnRpZnkgQ29udGFtaW5hbnRzIHdpdGggZGVjb250YW0tbWV0aG9kIFByZXZhbGVuY2UNCiNkZWNvbnRhbTogaHR0cHM6Ly9iZW5qam5lYi5naXRodWIuaW8vZGVjb250YW0vdmlnbmV0dGVzL2RlY29udGFtX2ludHJvLmh0bWwNCmBgYHtyfQ0Kc2FtcGxlX2RhdGEoZG9scGhpbjEudykkaXMubmVnIDwtIHNhbXBsZV9kYXRhKGRvbHBoaW4xLncpJFNhbXBsZV9vcl9Db250cm9sLjIgPT0gIkNvbnRyb2wiDQoNCiMjIyMjIyMjIyMjIyMjIyMjIyMNCiMjI3RocmVzaG9sZD0wLjUNCmNvbnRhbWRmLnByZXYwNS53ICA8LSBpc0NvbnRhbWluYW50KGRvbHBoaW4xLncsIG1ldGhvZD0icHJldmFsZW5jZSIsIG5lZz0iaXMubmVnIiwgdGhyZXNob2xkPTAuNSkNCnRhYmxlKGNvbnRhbWRmLnByZXYwNS53JGNvbnRhbWluYW50KQ0KDQojLS0+IEZhbHNlIGFyZSBub24tY29udGFtaW5hbnRzISEhDQojRkFMU0UgIFRSVUUgDQojIDE0NzEgICA1MjANCg0KIyBNYWtlIHBoeWxvc2VxIG9iamVjdCBvZiBwcmVzZW5jZS1hYnNlbmNlIGluIG5lZ2F0aXZlIGNvbnRyb2xzIGFuZCB0cnVlIHNhbXBsZXMNCnBzLnBhLncgPC0gdHJhbnNmb3JtX3NhbXBsZV9jb3VudHMoZG9scGhpbjEudywgZnVuY3Rpb24oYWJ1bmQpIDEqKGFidW5kPjApKQ0KDQpwcy5wYS5uZWcudyA8LSBwcnVuZV9zYW1wbGVzKHNhbXBsZV9kYXRhKHBzLnBhLncpJFNhbXBsZV9vcl9Db250cm9sLjIgPT0gIkNvbnRyb2wiLCBwcy5wYS53KQ0KDQpwcy5wYS5wb3MudyA8LSBwcnVuZV9zYW1wbGVzKHNhbXBsZV9kYXRhKHBzLnBhLncpJFNhbXBsZV9vcl9Db250cm9sLjIgPT0gIlRydWUgU2FtcGxlIiwgcHMucGEudykNCg0KIyBNYWtlIGRhdGEuZnJhbWUgb2YgcHJldmFsZW5jZSBpbiBwb3NpdGl2ZSBhbmQgbmVnYXRpdmUgc2FtcGxlcw0KZGYucGEwNS53IDwtIGRhdGEuZnJhbWUocGEucG9zLnc9dGF4YV9zdW1zKHBzLnBhLnBvcy53KSwgcGEubmVnLnc9dGF4YV9zdW1zKHBzLnBhLm5lZy53KSwNCiAgICAgICAgICAgICAgICAgICAgY29udGFtaW5hbnQ9Y29udGFtZGYucHJldjA1LnckY29udGFtaW5hbnQpDQoNCmRlY29udGFtLnBsb3QuMC41LncgPC0gZ2dwbG90KGRhdGE9ZGYucGEwNS53LCBhZXMoeD1wYS5uZWcudywgeT1wYS5wb3MudywgY29sb3I9Y29udGFtaW5hbnQpKSArIGdlb21fcG9pbnQoKSArDQogIHhsYWIoIlByZXZhbGVuY2UgKE5lZ2F0aXZlIENvbnRyb2xzKSIpICsgeWxhYigiUHJldmFsZW5jZSAoVHJ1ZSBTYW1wbGVzKSIpICsgZ2VvbV9qaXR0ZXIoKQ0KDQpnZ3NhdmUoImRlY29udGFtLnBsb3QuMC41LncuanBnIiwgcGxvdCA9IGRlY29udGFtLnBsb3QuMC41LncgLCBkZXZpY2UgPSAnanBnJywgd2lkdGggPSAxNjgsIGhlaWdodCA9IDEzMCwgdW5pdHMgPSAibW0iLA0KICAgICAgIGRwaSA9IDMwMCwgbGltaXRzaXplID0gVFJVRSkgDQoNCiMjI0ZyZXF1ZW5jeSBzY29yZXMgb2Ygek9UVXMgDQpoaXN0KGNvbnRhbWRmLnByZXYwNS53JHApDQpkZWNvbnRhbS5mcmVxLnNjb3JlLncgPC0gZ2dwbG90KGNvbnRhbWRmLnByZXYwNS53LCBhZXMocCkpICsgZ2VvbV9oaXN0b2dyYW0oYmlud2lkdGggPSAwLjEpDQoNCmdnc2F2ZSgiZGVjb250YW0uZnJlcS5zY29yZS4wNS53LmpwZyIsIHBsb3QgPSBkZWNvbnRhbS5mcmVxLnNjb3JlLncgLCBkZXZpY2UgPSAnanBnJywgd2lkdGggPSAxNjgsIGhlaWdodCA9IDEzMCwgdW5pdHMgPSAibW0iLA0KICAgICAgIGRwaSA9IDMwMCwgbGltaXRzaXplID0gVFJVRSkNCg0KYGBgDQojIyMjIyMjIyMjIyMjIyMjIyMjIyMjIyMjIyMjIyMjIyMjIyMjIyMjIyMjIyMjIw0KIyMjQWZ0ZXIgZGV0ZXJtaW5pbmcgdGhlIHBvb2wgd2F0ZXIgek9UVXMsIHdlIGRlbGV0ZSB0aGVtIGZyb20gdGhlIGRhdGEgc2V0ICNkYXQuZG9sX3Qubm8ud2F0ZXIuMi50DQpgYGB7cn0NCiMjI2NvbnRhbWRmLnByZXYwNS53IGNvbnRhaW5zIHRoZSBsaXN0IG9mIGNvbnRhbWluYW50IHpPVFVzDQoNCiMjI0V4dHJhY3QgY29sICRjb250YW1pbnQgZnJvbSBjb250YW1kZi5wcmV2MDUudyBhbmQgY29tYmluZSB3aXRoIGRhdC5kb2xfdC4xOS50LmZpbC4yDQojVmlldyhkYXQuZG9sX3QuMTkudC5maWwuMikNCmRpbShkYXQuZG9sX3QuMTkudC5maWwuMikNCiMxOTkxICAxMTENCg0Kek9UVV9maWwud2F0ZXIgPC0gY29udGFtZGYucHJldjA1LnckY29udGFtaW5hbnQNCg0KZGF0LmRvbF90LjE5LnQuZmlsLncgPC0gY2JpbmQoek9UVV9maWwud2F0ZXIsZGF0LmRvbF90LjE5LnQuZmlsLjIpDQpkaW0oZGF0LmRvbF90LjE5LnQuZmlsLncpDQojMTk5MSAgMTEyDQoNCiNWaWV3KGRhdC5kb2xfdC4xOS50LmZpbC53WzE6MTAsXSkNCg0KIyMjT25seSBrZWVwIHpPVFVTID09IEZhbHNlIChub24tY29udGFtaW5hbnRzKQ0KDQpkYXQuZG9sX3QuMTkudC5maWwudy4yIDwtIGRhdC5kb2xfdC4xOS50LmZpbC53DQoNCmRhdC5kb2xfdC4xOS50LmZpbC53LjIkdmFyaWFibGUgPC0gcm93bmFtZXMoZGF0LmRvbF90LjE5LnQuZmlsLncuMikNCmRpbShkYXQuZG9sX3QuMTkudC5maWwudy4yKQ0KIzE5OTEgIDExMw0KDQpkYXQuZG9sX3dhdGVyLmZpbCA8LSBkYXQuZG9sX3QuMTkudC5maWwudy4yICU+JSBmaWx0ZXIgKCF6T1RVX2ZpbC53YXRlciA9PSAnVFJVRScpDQpkaW0oZGF0LmRvbF93YXRlci5maWwpDQojMTQ3MSAgMTEzDQoNCiNEZWxldGUgY29sICR6T1RVX2ZpbC53YXRlciwgYnJpbmcgY29sICR2YXJpYWJsZSB0byB0aGUgZnJvbnQNCmRhdC5kb2xfd2F0ZXIuZmlsLjIgPC0gZGF0LmRvbF93YXRlci5maWxbLGMoMTEzLCAyOjExMildDQojVmlldyhkYXQuZG9sX3dhdGVyLmZpbC4yWzE6MTAsXSkNCg0KI1RyYW5zcG9zZSBvbmNlIG1vcmUNCmRhdC5kb2xfd2F0ZXIuZmlsLjMgPC0gZGF0LmRvbF93YXRlci5maWwuMg0Kcm93bmFtZXMoZGF0LmRvbF93YXRlci5maWwuMykgPC0gZGF0LmRvbF93YXRlci5maWwuMyR2YXJpYWJsZQ0KZGF0LmRvbF93YXRlci5maWwuNCA8LSBkYXQuZG9sX3dhdGVyLmZpbC4zWywtMV0NCg0KZGF0LmRvbF93YXRlci5maWwudCA8LSBhcy5kYXRhLmZyYW1lKHQoZGF0LmRvbF93YXRlci5maWwuNCkpDQpkaW0oZGF0LmRvbF93YXRlci5maWwudCkNCiMxMTEgMTQ3MQ0KI1ZpZXcoZGF0LmRvbF93YXRlci5maWwudFssMToxMF0pDQojcm93bmFtZXMoZGF0LmRvbF93YXRlci5maWwudCkNCg0KI0RlbGV0ZSBSb3dzIHdpdGggY29udHJvbHM6DQpkYXQuZG9sX3dhdGVyLmZpbC50LjIgPC0gZGF0LmRvbF93YXRlci5maWwudFtjKDE2OjIxLDMyOjcxLDc1OjExMSksXQ0KI3Jvd25hbWVzKGRhdC5kb2xfd2F0ZXIuZmlsLnQuMikNCmRpbShkYXQuZG9sX3dhdGVyLmZpbC50LjIpDQojODMgMTQ3MQ0KYGBgDQoNCiMjIyMjIyMjIyMjIyMjIyMjIyMjIyMjIyMjIyMjIyMjIw0KIyMjRGVsZXRlIGFsbCB3YXRlciB6T1RVcyBpbiBhdHRyaWJ1dGVzLmRvbF9yZHBfZmlsLjINCmBgYHtyfQ0KIyMjYXR0cmlidXRlcy5kb2xfcmRwX2ZpbC4yDQoNCiMjI0V4dHJhY3QgY29sICRjb250YW1pbmFudCBmcm9tIGNvbnRhbWRmLnByZXYwNSBhbmQgY29tYmluZSB3aXRoICNkYXQuZG9sX3Qubm8ud2F0ZXIuMi50IGFuZCBhdHRyaWJ1dGVzLmRvbF9yZHAuMw0KDQpkaW0oYXR0cmlidXRlcy5kb2xfcmRwX2ZpbC4yKQ0KIzE5OTEgICAgNw0KI1ZpZXcoYXR0cmlidXRlcy5kb2xfcmRwX2ZpbC4yKQ0KDQphdHRyaWJ1dGVzLmRvbF9yZHBfZmlsLjMgPC0gY2JpbmQoek9UVV9maWwud2F0ZXIsYXR0cmlidXRlcy5kb2xfcmRwX2ZpbC4yKQ0KZGltKGF0dHJpYnV0ZXMuZG9sX3JkcF9maWwuMykNCiMxOTkxICAgOA0KDQojVmlldyhhdHRyaWJ1dGVzLmRvbF9yZHBfZmlsLjMpDQoNCiMjI09ubHkga2VlcCB6T1RVUyA9PSBGYWxzZSAobm9uLWNvbnRhbWluYW50cykNCmF0dHJpYnV0ZXMuZG9sX3JkcF9maWwuNCA8LSBhdHRyaWJ1dGVzLmRvbF9yZHBfZmlsLjMNCg0KYXR0cmlidXRlcy5kb2xfcmRwX3dhdGVyLmZpbCA8LSBhdHRyaWJ1dGVzLmRvbF9yZHBfZmlsLjQgJT4lIGZpbHRlciAoIXpPVFVfZmlsLndhdGVyID09ICdUUlVFJykNCmRpbShhdHRyaWJ1dGVzLmRvbF9yZHBfd2F0ZXIuZmlsKQ0KIzE0NzEgIDgNCg0KI0RlbGV0ZSBjb2wgJHpPVFVfZmlsLndhdGVyDQphdHRyaWJ1dGVzLmRvbF9yZHBfd2F0ZXIuZmlsLjIgPC0gYXR0cmlidXRlcy5kb2xfcmRwX3dhdGVyLmZpbFssLTFdDQojVmlldyhhdHRyaWJ1dGVzLmRvbF9yZHBfd2F0ZXIuZmlsLjIpDQpgYGANCg0KIyMjIyMjIyMjIyMjIyMjIyMjIyMjIyMjIyMjIyMjIyMjDQojIyNDcmVhdGUgdGFibGUgd2l0aCBwb29sIHdhdGVyIHpPVFVzIHRoYXQgd2VyZSBkZWxldGVkDQpgYGB7cn0NCiMjI09ubHkga2VlcCB6T1RVUyA9PSBUUlVFIChjb250YW1pbmFudHMpDQphdHRyaWJ1dGVzLmRvbF9yZHBfd2F0ZXIuY29udHMgPC0gYXR0cmlidXRlcy5kb2xfcmRwX2ZpbC40ICU+JSBmaWx0ZXIgKHpPVFVfZmlsLndhdGVyID09ICdUUlVFJykNCmRpbShhdHRyaWJ1dGVzLmRvbF9yZHBfd2F0ZXIuY29udHMpDQojNTIwICAgOA0KDQojIyNEZWxldGUgY29sIDEgJiA4DQphdHRyaWJ1dGVzLmRvbF9yZHBfd2F0ZXIuY29udHMuMiA8LSBhdHRyaWJ1dGVzLmRvbF9yZHBfd2F0ZXIuY29udHNbLC1jKDEsOCldDQoNCiMjI1NhdmUgYXMgY3N2LWZpbGUgKGZvciBzdXBwbGVtZW50cykNCndyaXRlLmNzdihmaWxlID0gJ2F0dHJpYnV0ZXMuZG9sX3JkcF93YXRlci5jb250cy4yLmNzdicsIGF0dHJpYnV0ZXMuZG9sX3JkcF93YXRlci5jb250cy4yKQ0KYGBgDQoNCiMjIyMjIyMjIyMjIyMjIyMjIyMjIyMjIyMjIyMjIyMjIw0KIyMjRGVsZXRlIGFsbCBwb29sIHdhdGVyIHNhbXBsZXMgaW4gRG9sQmxvd192YXIuZmlsLjQNCmBgYHtyfQ0KI1ZpZXcoRG9sQmxvd192YXIuMikNCkRvbEJsb3dfdmFyLndhdGVyLmZpbCA8LSBEb2xCbG93X3Zhci5maWwuNCAlPiUgZmlsdGVyICghU2FtcGxlX29yX0NvbnRyb2wuMiA9PSAnQ29udHJvbCcpDQpkaW0oRG9sQmxvd192YXIud2F0ZXIuZmlsKQ0KIzExMSAgIDMNCg0KIyMjRGVsZXRlIGNvbCAxICRTYW1wbGUNCkRvbEJsb3dfdmFyLndhdGVyLmZpbC4yIDwtIERvbEJsb3dfdmFyLndhdGVyLmZpbFssLTFdDQpgYGANCg0KIyMjIyMjIyMjIyMjIyMjIyMjIyMjIyMjIyMjIyMjIyMjDQojIyNEYXRhc2V0cyB3aXRob3V0IHdhdGVyIHpPVFVzDQojIyNhdHRyaWJ1dGVzLmRvbF9yZHBfd2F0ZXIuZmlsLjIsIERvbEJsb3dfdmFyLndhdGVyLmZpbC4yLCBkYXQuZG9sX3dhdGVyLmZpbC50LjINCiMjIyMjIyMjIyMjIyMjIyMjIyMjIyMjIyMjIyMjIyMjIw0KDQojIyMjIyMjIyMjIyMjIyMjIyMjIyMjIyMjIyMjIyMjIyMNCiMjI0FscGhhIGRpdmVyc2l0eSBvZiBkb2xwaGluIHNhbXBsZXMgd2l0aG91dCBwb29sIHdhdGVyIGNvbnRhbWluYW50cw0KDQpgYGB7cn0NCg0KZGltKGRhdC5kb2xfd2F0ZXIuZmlsLnQuMikNCiMxMTYgMTg4Nw0KI1ZpZXcoZGF0LmRvbF93YXRlci5maWwudC4yWywxOjEwXSkNCg0KZGF0LmRvbF93YXRlci5maWwudC4zIDwtIGRhdC5kb2xfd2F0ZXIuZmlsLnQuMg0KDQpkYXQuZG9sX3dhdGVyLmZpbC50LjMkRG9scGhpbiA8LSByb3duYW1lcyhkYXQuZG9sX3dhdGVyLmZpbC50LjMpDQpkaW0oZGF0LmRvbF93YXRlci5maWwudC4zKQ0KIzgzIDE0NzINCmRhdC5kb2xfd2F0ZXIuZmlsLnQuNCA8LSBkYXQuZG9sX3dhdGVyLmZpbC50LjNbLGMoMTQ3MiwgMToxNDcxKV0NCg0KDQojIFJhcmVmYWN0aW9uDQptaW4ocm93U3VtcyhkYXQuZG9sX3dhdGVyLmZpbC50LjRbLDI6MTQ3Ml0pKQ0KIzEsMzI3DQojcm93bmFtZXMoZGF0LmRvbF93YXRlci5maWwudC40KQ0KDQojIyNOdWRnZWVfMjQgaGFzIHRoZSBsb3dlc3QgcmVhZCBjb3VudCA9IDEzMjcgLS0+IGRlbGV0ZSBmb3IgYWxwaGEgZGl2ZXJzaXR5DQpkYXQuZG9sX3dhdGVyLmZpbC50LmFscGhhID0gZGF0LmRvbF93YXRlci5maWwudC40Wy1jKDQwKSxdDQojODcgMTg4Nw0KDQojIFJhcmVmYWN0aW9uDQptaW4ocm93U3VtcyhkYXQuZG9sX3dhdGVyLmZpbC50LmFscGhhWywyOjE0NzJdKSkNCiMxNiw1NzgNCg0KIyMjLS0+IFNtYWxsZXN0IG51bWJlciBvZiByZWFkcyBpcyAxNiw1NzguDQoNCiNEZWxldGUgY29sIDEgZm9yIHJhcmVmYWN0aW9uDQpkYXQuZG9sX3dhdGVyLmZpbC50LmFscGhhLjIgPSBkYXQuZG9sX3dhdGVyLmZpbC50LmFscGhhWywtYygxKV0NCg0KZGF0LmRvbF93YXRlci5maWwudC5hbHBoYS5yZmYgPSBycmFyZWZ5KHggPSBkYXQuZG9sX3dhdGVyLmZpbC50LmFscGhhLjIsIHNhbXBsZSA9IDE2NTc4KQ0Kcm93U3VtcyhkYXQuZG9sX3dhdGVyLmZpbC50LmFscGhhLnJmZikNCg0KIyBDYWxjdWxhdGluZyBhbHBoYSBkaXZlcnNpdHkNCnNwZWNpZXNfcmljaG5lc3Mud2F0ZXIuZmlsID0gTlVMTA0Kc3BlY2llc19kaXZlcnNpdHkud2F0ZXIuZmlsID0gTlVMTA0Kc3BlY2llc19lc3RpbWF0b3Iud2F0ZXIuZmlsID0gTlVMTA0KDQpmb3IoaSBpbiAxOjEwMCkNCnsNCiAgcHJpbnQoaSkNCiAgZGF0LmRvbF93YXRlci5maWwudC5hbHBoYS5yZmYgPSBycmFyZWZ5KHggPSBkYXQuZG9sX3dhdGVyLmZpbC50LmFscGhhLjIsIHNhbXBsZSA9IDE2NTc4KQ0KICBzcGVjaWVzX3JpY2huZXNzLndhdGVyLmZpbCA9IHJiaW5kKHNwZWNpZXNfcmljaG5lc3Mud2F0ZXIuZmlsLCBzcGVjbnVtYmVyKGRhdC5kb2xfd2F0ZXIuZmlsLnQuYWxwaGEucmZmKSkNCiAgc3BlY2llc19kaXZlcnNpdHkud2F0ZXIuZmlsID0gcmJpbmQoc3BlY2llc19kaXZlcnNpdHkud2F0ZXIuZmlsLCBkaXZlcnNpdHkoZGF0LmRvbF93YXRlci5maWwudC5hbHBoYS5yZmYpKQ0KICBzcGVjaWVzX2VzdGltYXRvci53YXRlci5maWwgPSByYmluZChzcGVjaWVzX2VzdGltYXRvci53YXRlci5maWwsIGVzdGltYXRlUihkYXQuZG9sX3dhdGVyLmZpbC50LmFscGhhLnJmZilbMixdKQ0KfQ0Kc3BlY2llc19yaWNobmVzcy53YXRlci5maWxfYXZnID0gY29sTWVhbnMoc3BlY2llc19yaWNobmVzcy53YXRlci5maWwpDQpzcGVjaWVzX2RpdmVyc2l0eS53YXRlci5maWxfYXZnID0gbG9nKGNvbE1lYW5zKGV4cChzcGVjaWVzX2RpdmVyc2l0eS53YXRlci5maWwpKSkNCnNwZWNpZXNfZXN0aW1hdG9yLndhdGVyLmZpbF9hdmcgPSBjb2xNZWFucyhzcGVjaWVzX2VzdGltYXRvci53YXRlci5maWwpDQoNCmFscGhhLmRvbHBoaW4ud2F0ZXIuZmlsID0gZGF0YS5mcmFtZShzcGVjaWVzX3JpY2huZXNzLndhdGVyLmZpbF9hdmcsIHNwZWNpZXNfZGl2ZXJzaXR5LndhdGVyLmZpbF9hdmcsIHNwZWNpZXNfZXN0aW1hdG9yLndhdGVyLmZpbF9hdmcpDQoNCmFscGhhLmRvbHBoaW4ud2F0ZXIuZmlsLjIgPSBhbHBoYS5kb2xwaGluLndhdGVyLmZpbA0KDQojIyNKb2luIGFscGhhLmRvbHBoaW4ud2F0ZXIuZmlsLjIgd2l0aCBEb2xCbG93X3Zhci50ZWNoLmZpbA0KYWxwaGEuZG9scGhpbi53YXRlci5maWwuMiREb2xwaGluID0gcm93bmFtZXMoYWxwaGEuZG9scGhpbi53YXRlci5maWwuMikNCg0KI21lYW4gcmljaG5lc3MuZG9sDQptZWFuKGFscGhhLmRvbHBoaW4ud2F0ZXIuZmlsLjJbLDFdKQ0KDQpzZChhbHBoYS5kb2xwaGluLndhdGVyLmZpbC4yWywxXSkNCg0KYGBgDQoNCiMjIyMjIyMjIyMjIyMjIyMjIyMjIyMjIyMjIyMjIyMjIyMjIyMjIyMjIyMjIyMjIyMjIyMjIyMjIw0KIyMjIyMjIyMjI0NoZWNrIHJpY2huZXNzIGJldHdlZW4gZG9scGhpbiBzYW1wbGVzDQpgYGB7cn0NCiMjI1VzZSBhbHBoYS5kb2xwaGluLndhdGVyLmZpbC4yDQojIE11bHRpcGxlIHJvd3MgcGVyIGluZGl2aWR1YWwgZG9scGhpbiwgb25lIGZvciBlYWNoIG1lYXN1cmVtZW50IG9jY2FzaW9uKS4gDQojIFRoaXMgc2hvdWxkIGhhdmUgYSBjb2x1bW4gdGhhdCBpZGVudGlmaWVzIHRoZSBkb2xwaGluLCBhIGNvbHVtbiB0aGF0IGlkZW50aWZpZXMgDQojIHRoZSB0aW1lIHBvaW50LCBhbnkgZG9scGhpbiBjaGFyYWN0ZXJpc3RpY3MgdGhhdCB5b3Ugd2FudCB0byBhZGp1c3QgZm9yIChlLmcuIHBvb2wpIA0KIyBhbmQgYSBjb2x1bW4gd2l0aCB0aGUgb3V0Y29tZS4gSW4gdGhlIGZvbGxvd2luZyBjb2RlLCB0aGVzZSBhcmUgDQojIGNhbGxlZCBJRCwgdGltZSwgcG9vbCBhbmQgcmljaG5lc3MNCg0KI1ZpZXcoYWxwaGEuZG9scGhpbi53YXRlci5maWwuMikNCm5hbWVzKGFscGhhLmRvbHBoaW4ud2F0ZXIuZmlsLjIpWzE6M10gPC0gYygncmljaG5lc3MnLCAnZGl2ZXJzaXR5JywgJ3NwZWNpZXNfZXN0aW1hdG9yJykNCg0KI1ZpZXcoRG9sQmxvd192YXIpDQpEb2xCbG93X3Zhcl8yIDwtIERvbEJsb3dfdmFyW29yZGVyKERvbEJsb3dfdmFyJFNwZWNpZXMpLF0NCkRvbEJsb3dfdmFyXzMgPC0gRG9sQmxvd192YXJfMlssYygzLDcpXQ0KDQphbHBoYS5kb2xwaGluLndhdGVyLmZpbC4zIDwtIGFscGhhLmRvbHBoaW4ud2F0ZXIuZmlsLjINCmFscGhhLmRvbHBoaW4ud2F0ZXIuZmlsLjMkRG9scGhpbi4yIDwtIHJvd25hbWVzKGFscGhhLmRvbHBoaW4ud2F0ZXIuZmlsLjMpDQoNCiNKb2luIGFscGhhLmRvbHBoaW4ud2F0ZXIuZmlsLjMgYW5kIERvbEJsb3dfdmFyXzMNCmFscGhhLmRvbHBoaW4ud2F0ZXIuZmlsLjQgPC0gYWxwaGEuZG9scGhpbi53YXRlci5maWwuMyAlPiUgbGVmdF9qb2luKERvbEJsb3dfdmFyXzMpDQoNCiNTZXBhcmF0ZSBjb2wgRG9scGhpbg0KYWxwaGEuZG9scGhpbi53YXRlci5maWwuNSA8LSBzZXBhcmF0ZShhbHBoYS5kb2xwaGluLndhdGVyLmZpbC40LERvbHBoaW4uMiwgYygnSUQnLCd0aW1lJykpDQojVmlldyhhbHBoYS5kb2xwaGluLndhdGVyLmZpbC41KQ0KDQphbHBoYS5kb2xwaGluLndhdGVyLmZpbC42IDwtIGFscGhhLmRvbHBoaW4ud2F0ZXIuZmlsLjUNCmFscGhhLmRvbHBoaW4ud2F0ZXIuZmlsLjYkV2F0ZXJfc2FtcGxlLjIgPC0gYWxwaGEuZG9scGhpbi53YXRlci5maWwuNiRXYXRlcl9zYW1wbGUNCg0KI1NlcGFyYXRlIGNvbCBXYXRlcl9zYW1wbGUNCmFscGhhLmRvbHBoaW4ud2F0ZXIuZmlsLjcgPC0gc2VwYXJhdGUoYWxwaGEuZG9scGhpbi53YXRlci5maWwuNixXYXRlcl9zYW1wbGUsIGMoJ3Bvb2wnLCdwb29sLnRpbWUnKSkNCiNWaWV3KGFscGhhLmRvbHBoaW4ud2F0ZXIuZmlsLjcpDQoNCndyaXRlLmNzdihhbHBoYS5kb2xwaGluLndhdGVyLmZpbC43LCBmaWxlID0gJ2FscGhhLmRpdmVyc2l0eS5kb2xwaGlucy5jc3YnKQ0KDQojbmFtZXMoYWxwaGEuZG9scGhpbi53YXRlci5maWwuNykNCg0KbWVhbihhbHBoYS5kb2xwaGluLndhdGVyLmZpbC43JHJpY2huZXNzKQ0KDQpzZChhbHBoYS5kb2xwaGluLndhdGVyLmZpbC43JHJpY2huZXNzKQ0KDQojIyNNYWtlIHN1cmUgYWxsIG51bWVyaWNzIGFyZSBudW1lcmljcw0Kc3RyKGFscGhhLmRvbHBoaW4ud2F0ZXIuZmlsLjcpDQphbHBoYS5kb2xwaGluLndhdGVyLmZpbC43JHRpbWUgPC0gYXMubnVtZXJpYyhhbHBoYS5kb2xwaGluLndhdGVyLmZpbC43JHRpbWUpDQpgYGANCg0KIyMjIyMjIyMjIyMjIyMjIyMjIyMjIyMjIyMjIyMjIyMjIyMjIyMjIyMjIyMjIyMjDQojIyMjIyMjIyMjI1Rlc3QgZGlmZmVyZW5jZXMgaW4gYWxwaGEtZGl2ZXJzaXR5DQojIyNSaWNobmVzcw0KYGBge3J9DQojIyNsbWU6IExpbmVhciBNaXhlZC1FZmZlY3RzIE1vZGVscw0KI1RoaXMgZ2VuZXJpYyBmdW5jdGlvbiBmaXRzIGEgbGluZWFyIG1peGVkLWVmZmVjdHMgbW9kZWwgDQojaW4gdGhlIGZvcm11bGF0aW9uIGRlc2NyaWJlZCBpbiBMYWlyZCBhbmQgV2FyZSAoMTk4MikgYnV0IA0KI2FsbG93aW5nIGZvciBuZXN0ZWQgcmFuZG9tIGVmZmVjdHMuIFRoZSB3aXRoaW4tZ3JvdXAgZXJyb3JzIA0KI2FyZSBhbGxvd2VkIHRvIGJlIGNvcnJlbGF0ZWQgYW5kL29yIGhhdmUgdW5lcXVhbCB2YXJpYW5jZXMuDQoNCiMjIyMjIyMjIyMjIyMjIyMjIyMjIyMjIyMjDQojIyNJbmZsdWVuY2Ugb2YgdGltZToNCmhpc3QoYWxwaGEuZG9scGhpbi53YXRlci5maWwuNyRyaWNobmVzcykNCg0KYWxwaGEuZG9scGhpbi53YXRlci5maWwuNyR0aW1lIDwtIGFzLmZhY3RvcihhbHBoYS5kb2xwaGluLndhdGVyLmZpbC43JHRpbWUpDQoNCiMjIwlXZXJlIHRoZXJlIGNoYW5nZXMgaW4gcmljaG5lc3Mgb3ZlciB0aGUgc2FtcGxpbmcgcGVyaW9kIHdpdGhpbiB0aGUgaW5kaXZpZHVhbCBkb2xwaGluPw0KbG1lLnJpY2huZXNzIDwtIGxtZShyaWNobmVzcyB+IHRpbWUsIHJhbmRvbSA9IH4xfElELCBkYXRhID0gYWxwaGEuZG9scGhpbi53YXRlci5maWwuNykNCg0Kc3VtbWFyeShsbWUucmljaG5lc3MpDQphbm92YShsbWUucmljaG5lc3MpDQoNCiMgICAgICAgICAgICAgbnVtREYgZGVuREYgICBGLXZhbHVlIHAtdmFsdWUNCiMgKEludGVyY2VwdCkgICAgIDEgICAgNTQgMjU0Ljk2MTgwICA8LjAwMDENCiMgdGltZSAgICAgICAgICAgMTUgICAgNTQgICAwLjkzMTA0ICAwLjUzNjgNCg0KIyMjLS0+IFRpbWUgZG9lcyBubyBoYXZlIGFuIGVmZmVjdCBvbiByaWNobmVzcyBpbiBpbmRpdmlkdWFsIGRvbHBoaW5zLg0KDQoNCiMjIyMjIyMjIyMjIyMjIyMjIyMjIyMjIyMjDQojIyNJbmZsdWVuY2Ugb2YgSUQ6DQoNCiMgTW9kZWwgd2l0aCByYW5kb20gaW50ZXJjZXB0cw0KbW9kZWwxIDwtIGxtZShyaWNobmVzcyB+IHRpbWUsIHJhbmRvbSA9IH4gMSB8IElELCBkYXRhID0gYWxwaGEuZG9scGhpbi53YXRlci5maWwuNywNCiAgICAgICAgICAgICAgbWV0aG9kID0gIlJFTUwiKQ0KIyBNb2RlbCB3aXRob3V0IHJhbmRvbSBpbnRlcmNlcHRzDQptb2RlbDAgPC0gbG0ocmljaG5lc3MgfiB0aW1lLCBkYXRhID0gYWxwaGEuZG9scGhpbi53YXRlci5maWwuNykNCg0KIyBPYnNlcnZlZCBsaWtlbGlob29kIHJhdGlvIHRlc3Qgc3RhdGlzdGljDQpvYnMubHIgPC0gYXMubnVtZXJpYygtMiAqIChsb2dMaWsobW9kZWwwLCBSRU1MID0gVFJVRSkgLSBsb2dMaWsobW9kZWwxLCBSRU1MID0gVFJVRSkpKQ0KDQojIFNpbXVsYXRlIGRhdGEgZnJvbSB0aGUgbW9kZWwgd2l0aG91dCByYW5kb20gZWZmZWN0cw0Kc2ltZGF0IDwtIHNpbXVsYXRlKG1vZGVsMCwgbnNpbSA9IDEwMDAwLCBzZWVkID0gMjAyMDExMDYpDQoNCiMgRnVuY3Rpb24gdG8gZml0IGJvdGggbW9kZWxzIGFuZCBjYWxjdWxhdGUgdGhlIExSIHN0YXRpc3RpYw0KY21wX21vZGVscyA8LSBmdW5jdGlvbih5KSB7DQogIG5kYXQgPC0gY2JpbmQoYWxwaGEuZG9scGhpbi53YXRlci5maWwuNywgeSA9IHkpDQogIG1kbDEgPC0gbG1lKHkgfiB0aW1lICwgcmFuZG9tID0gfiAxIHwgSUQsIGRhdGEgPSBuZGF0LCBtZXRob2QgPSAiUkVNTCIpDQogIG1kbDAgPC0gbG0oeSB+IHRpbWUgLCBkYXRhID0gbmRhdCkNCiAgYTAgPC0gYXMubnVtZXJpYygtMiAqIChsb2dMaWsobWRsMCwgUkVNTCA9IFRSVUUpIC0gbG9nTGlrKG1kbDEsIFJFTUwgPSBUUlVFKSkpDQogIGEwDQp9DQoNCiMgR2V0IHBhcmFtZXRyaWMgYm9vdHN0cmFwIHRlc3Qgc3RhdGlzdGljcw0KY21wMCA8LSBtYXBfZGJsKHNpbWRhdCwgY21wX21vZGVscykNCiMgR2V0IHBhcmFtZXRyaWMgYm9vdHN0cmFwIHAtdmFsdWUNCnBhcmEucCA8LSBtZWFuKGMoY21wMCwgb2JzLmxyKSA+PSBvYnMubHIpDQpwYXJhLnANCiMwLjAwMTQ5OTg1DQoNCmBgYA0KDQojIyMjIyMjIyMjIyMjIyMjIyMjIyMjIyMjIyMjIyMjIyMjIyMjDQojIyNTaGFubm9uLVdpZW5lciBkaXZlcnNpdHkNCmBgYHtyfQ0KbWVhbihhbHBoYS5kb2xwaGluLndhdGVyLmZpbC43JGRpdmVyc2l0eSkNCg0Kc2QoYWxwaGEuZG9scGhpbi53YXRlci5maWwuNyRkaXZlcnNpdHkpDQoNCiMjIyMjIyMjIyMjIyMjIyMjIyMjIyMjIyMjDQojIyNJbmZsdWVuY2Ugb2YgdGltZToNCmhpc3QoYWxwaGEuZG9scGhpbi53YXRlci5maWwuNyRkaXZlcnNpdHkpDQoNCiMjIwlXZXJlIHRoZXJlIGNoYW5nZXMgaW4gZGl2ZXJzaXR5IG92ZXIgdGhlIHNhbXBsaW5nIHBlcmlvZCB3aXRoaW4gdGhlIGluZGl2aWR1YWwgZG9scGhpbj8NCmxtZS5kaXZlcnNpdHkgPC0gbG1lKGRpdmVyc2l0eSB+IHRpbWUsIHJhbmRvbSA9IH4xfElELCBkYXRhID0gYWxwaGEuZG9scGhpbi53YXRlci5maWwuNykNCg0Kc3VtbWFyeShsbWUuZGl2ZXJzaXR5KQ0KYW5vdmEobG1lLmRpdmVyc2l0eSkNCg0KIyAgICAgICAgICAgICBudW1ERiBkZW5ERiAgIEYtdmFsdWUgcC12YWx1ZQ0KIyAoSW50ZXJjZXB0KSAgICAgMSAgICA1NCAxNTc2LjgxMTYgIDwuMDAwMQ0KIyB0aW1lICAgICAgICAgICAxNSAgICA1NCAgICAxLjYzODkgIDAuMDkzOA0KDQojIyMtLT4gVGltZSBkb2VzIG5vIGhhdmUgYW4gZWZmZWN0IG9uIGRpdmVyc2l0eSBpbiBpbmRpdmlkdWFsIGRvbHBoaW5zLg0KDQojIyMjIyMjIyMjIyMjIyMjIyMjIyMjIyMjIw0KIyMjSW5mbHVlbmNlIG9mIElEOg0KDQojIE1vZGVsIHdpdGggcmFuZG9tIGludGVyY2VwdHMNCm1vZGVsMSA8LSBsbWUoZGl2ZXJzaXR5IH4gdGltZSwgcmFuZG9tID0gfiAxIHwgSUQsIGRhdGEgPSBhbHBoYS5kb2xwaGluLndhdGVyLmZpbC43LA0KICAgICAgICAgICAgICBtZXRob2QgPSAiUkVNTCIpDQojIE1vZGVsIHdpdGhvdXQgcmFuZG9tIGludGVyY2VwdHMNCm1vZGVsMCA8LSBsbShkaXZlcnNpdHkgfiB0aW1lLCBkYXRhID0gYWxwaGEuZG9scGhpbi53YXRlci5maWwuNykNCg0KIyBPYnNlcnZlZCBsaWtlbGlob29kIHJhdGlvIHRlc3Qgc3RhdGlzdGljDQpvYnMubHIgPC0gYXMubnVtZXJpYygtMiAqIChsb2dMaWsobW9kZWwwLCBSRU1MID0gVFJVRSkgLSBsb2dMaWsobW9kZWwxLCBSRU1MID0gVFJVRSkpKQ0KDQojIFNpbXVsYXRlIGRhdGEgZnJvbSB0aGUgbW9kZWwgd2l0aG91dCByYW5kb20gZWZmZWN0cw0Kc2ltZGF0IDwtIHNpbXVsYXRlKG1vZGVsMCwgbnNpbSA9IDEwMDAwLCBzZWVkID0gMjAyMDExMDYpDQoNCiMgRnVuY3Rpb24gdG8gZml0IGJvdGggbW9kZWxzIGFuZCBjYWxjdWxhdGUgdGhlIExSIHN0YXRpc3RpYw0KY21wX21vZGVscyA8LSBmdW5jdGlvbih5KSB7DQogIG5kYXQgPC0gY2JpbmQoYWxwaGEuZG9scGhpbi53YXRlci5maWwuNywgeSA9IHkpDQogIG1kbDEgPC0gbG1lKHkgfiB0aW1lICwgcmFuZG9tID0gfiAxIHwgSUQsIGRhdGEgPSBuZGF0LCBtZXRob2QgPSAiUkVNTCIpDQogIG1kbDAgPC0gbG0oeSB+IHRpbWUgLCBkYXRhID0gbmRhdCkNCiAgYTAgPC0gYXMubnVtZXJpYygtMiAqIChsb2dMaWsobWRsMCwgUkVNTCA9IFRSVUUpIC0gbG9nTGlrKG1kbDEsIFJFTUwgPSBUUlVFKSkpDQogIGEwDQp9DQoNCiMgR2V0IHBhcmFtZXRyaWMgYm9vdHN0cmFwIHRlc3Qgc3RhdGlzdGljcw0KY21wMCA8LSBtYXBfZGJsKHNpbWRhdCwgY21wX21vZGVscykNCiMgR2V0IHBhcmFtZXRyaWMgYm9vdHN0cmFwIHAtdmFsdWUNCnBhcmEucCA8LSBtZWFuKGMoY21wMCwgb2JzLmxyKSA+PSBvYnMubHIpDQpwYXJhLnANCiMwLjAxNjM5ODM2DQoNCmBgYA0KDQojIyNDaGFvMQ0KYGBge3J9DQojIyNDaGFvMQ0KI0NhbGN1bGF0ZSBDaGFvMSBvbiByYXJlZmllZCBkYXRhDQojQ2hhbyBzcGVjaWVzIGVzdGltYXRvciBmb3IgYWJ1bmRhbmNlDQojVXNlIGRhdC5kb2xfdC4xMC50ZWNoLmZpbC5hbHBoYS5yZmYgYW5kIERvbEJsb3dfdmFyLnRlY2guZmlsDQoNCkNoYW8xLndhdGVyLmZpbCA8LSBhc190aWJibGUoYXBwbHkoZGF0LmRvbF93YXRlci5maWwudC5hbHBoYS5yZmYsIDEsIGNoYW8xKSkNCg0KQ2hhbzEud2F0ZXIuZmlsLjIgPC0gY2JpbmQocm93bmFtZXMoZGF0LmRvbF93YXRlci5maWwudC5hbHBoYS5yZmYpLENoYW8xLndhdGVyLmZpbCkNCg0KbmFtZXMoQ2hhbzEud2F0ZXIuZmlsLjIpWzFdIDwtICdEb2xwaGluJw0KbmFtZXMoQ2hhbzEud2F0ZXIuZmlsLjIpWzJdIDwtICdDaGFvMScNCg0KI0pvaW4gQ2hhbzEud2F0ZXIuZmlsLjIgYW5kIGFscGhhLmRvbHBoaW4udGVjaC5maWwuZG9sLjcNCmFscGhhLmRvbHBoaW4ud2F0ZXIuZmlsLjggPC0gYWxwaGEuZG9scGhpbi53YXRlci5maWwuNyAlPiUgbGVmdF9qb2luKENoYW8xLndhdGVyLmZpbC4yKQ0KDQptZWFuKGFscGhhLmRvbHBoaW4ud2F0ZXIuZmlsLjgkQ2hhbzEpDQoNCnNkKGFscGhhLmRvbHBoaW4ud2F0ZXIuZmlsLjgkQ2hhbzEpDQoNCiMjIyMjIyMjIyMjIyMjIyMjIyMjIyMjIyMjDQojIyNJbmZsdWVuY2Ugb2YgdGltZToNCmhpc3QoYWxwaGEuZG9scGhpbi53YXRlci5maWwuOCRDaGFvMSkNCg0KIyMjCVdlcmUgdGhlcmUgY2hhbmdlcyBpbiBDaGFvMSBvdmVyIHRoZSBzYW1wbGluZyBwZXJpb2Qgd2l0aGluIHRoZSBpbmRpdmlkdWFsIGRvbHBoaW4/DQpsbWUuQ2hhbzEgPC0gbG1lKENoYW8xIH4gdGltZSwgcmFuZG9tID0gfjF8SUQsIGRhdGEgPSBhbHBoYS5kb2xwaGluLndhdGVyLmZpbC44KQ0KDQpzdW1tYXJ5KGxtZS5DaGFvMSkNCmFub3ZhKGxtZS5DaGFvMSkNCg0KIyAgICAgICAgICAgICBudW1ERiBkZW5ERiAgIEYtdmFsdWUgcC12YWx1ZQ0KIyhJbnRlcmNlcHQpICAgICAxICAgIDU0IDI4My40NzQ3MyAgPC4wMDAxDQojdGltZSAgICAgICAgICAgMTUgICAgNTQgICAxLjAzMDI2ICAwLjQ0MDQNCg0KIyMjLS0+IFRpbWUgZG9lcyBubyBoYXZlIGFuIGVmZmVjdCBvbiBDaGFvMSBpbiBpbmRpdmlkdWFsIGRvbHBoaW5zLg0KDQojIyMjIyMjIyMjIyMjIyMjIyMjIyMjIyMjIw0KIyMjSW5mbHVlbmNlIG9mIElEOg0KDQojIE1vZGVsIHdpdGggcmFuZG9tIGludGVyY2VwdHMNCm1vZGVsMSA8LSBsbWUoQ2hhbzEgfiB0aW1lLCByYW5kb20gPSB+IDEgfCBJRCwgZGF0YSA9IGFscGhhLmRvbHBoaW4ud2F0ZXIuZmlsLjgsDQogICAgICAgICAgICAgIG1ldGhvZCA9ICJSRU1MIikNCiMgTW9kZWwgd2l0aG91dCByYW5kb20gaW50ZXJjZXB0cw0KbW9kZWwwIDwtIGxtKENoYW8xIH4gdGltZSwgZGF0YSA9IGFscGhhLmRvbHBoaW4ud2F0ZXIuZmlsLjgpDQoNCiMgT2JzZXJ2ZWQgbGlrZWxpaG9vZCByYXRpbyB0ZXN0IHN0YXRpc3RpYw0Kb2JzLmxyIDwtIGFzLm51bWVyaWMoLTIgKiAobG9nTGlrKG1vZGVsMCwgUkVNTCA9IFRSVUUpIC0gbG9nTGlrKG1vZGVsMSwgUkVNTCA9IFRSVUUpKSkNCg0KIyBTaW11bGF0ZSBkYXRhIGZyb20gdGhlIG1vZGVsIHdpdGhvdXQgcmFuZG9tIGVmZmVjdHMNCnNpbWRhdCA8LSBzaW11bGF0ZShtb2RlbDAsIG5zaW0gPSAxMDAwMCwgc2VlZCA9IDIwMjAxMTA2KQ0KDQojIEZ1bmN0aW9uIHRvIGZpdCBib3RoIG1vZGVscyBhbmQgY2FsY3VsYXRlIHRoZSBMUiBzdGF0aXN0aWMNCmNtcF9tb2RlbHMgPC0gZnVuY3Rpb24oeSkgew0KICBuZGF0IDwtIGNiaW5kKGFscGhhLmRvbHBoaW4ud2F0ZXIuZmlsLjgsIHkgPSB5KQ0KICBtZGwxIDwtIGxtZSh5IH4gdGltZSAsIHJhbmRvbSA9IH4gMSB8IElELCBkYXRhID0gbmRhdCwgbWV0aG9kID0gIlJFTUwiKQ0KICBtZGwwIDwtIGxtKHkgfiB0aW1lICwgZGF0YSA9IG5kYXQpDQogIGEwIDwtIGFzLm51bWVyaWMoLTIgKiAobG9nTGlrKG1kbDAsIFJFTUwgPSBUUlVFKSAtIGxvZ0xpayhtZGwxLCBSRU1MID0gVFJVRSkpKQ0KICBhMA0KfQ0KDQojIEdldCBwYXJhbWV0cmljIGJvb3RzdHJhcCB0ZXN0IHN0YXRpc3RpY3MNCmNtcDAgPC0gbWFwX2RibChzaW1kYXQsIGNtcF9tb2RlbHMpDQojIEdldCBwYXJhbWV0cmljIGJvb3RzdHJhcCBwLXZhbHVlDQpwYXJhLnAgPC0gbWVhbihjKGNtcDAsIG9icy5scikgPj0gb2JzLmxyKQ0KcGFyYS5wDQpgYGANCg0KIyMjQUNFDQpgYGB7cn0NCiMjIyMjIyMjIyMjIyMjIyMjIyMjIyMgIyMjIyMjIyMjIyMjIyMjIyMjIyMjIyANCiMjI0FDRQ0KI0NhbGN1bGF0ZSBBQ0Ugb24gcmFyZWZpZWQgZGF0YQ0KI0FDRSBzcGVjaWVzIGVzdGltYXRvciBmb3IgYWJ1bmRhbmNlDQoNCkFDRS53YXRlci5maWwgPC0gYXNfdGliYmxlKGFwcGx5KGRhdC5kb2xfd2F0ZXIuZmlsLnQuYWxwaGEucmZmLCAxLCBBQ0UpKQ0KDQpBQ0Uud2F0ZXIuZmlsLjIgPC0gY2JpbmQocm93bmFtZXMoZGF0LmRvbF93YXRlci5maWwudC5hbHBoYS5yZmYpLEFDRS53YXRlci5maWwpDQoNCm5hbWVzKEFDRS53YXRlci5maWwuMilbMV0gPC0gJ0RvbHBoaW4nDQpuYW1lcyhBQ0Uud2F0ZXIuZmlsLjIpWzJdIDwtICdBQ0UnDQoNCiNKb2luIEFDRS50ZWNoLmZpbC4yIGFuZCBhbHBoYS5kb2xwaGluLnRlY2guZmlsLmRvbC43DQphbHBoYS5kb2xwaGluLndhdGVyLmZpbC45IDwtIGxlZnRfam9pbihBQ0Uud2F0ZXIuZmlsLjIsIGFscGhhLmRvbHBoaW4ud2F0ZXIuZmlsLjcpDQoNCm1lYW4oYWxwaGEuZG9scGhpbi53YXRlci5maWwuOSRBQ0UpDQoNCnNkKGFscGhhLmRvbHBoaW4ud2F0ZXIuZmlsLjkkQUNFKQ0KDQojIyMjIyMjIyMjIyMjIyMjIyMjIyMjIyMjIw0KIyMjSW5mbHVlbmNlIG9mIHRpbWU6DQpoaXN0KGFscGhhLmRvbHBoaW4ud2F0ZXIuZmlsLjkkQUNFKQ0KDQojIyMJV2VyZSB0aGVyZSBjaGFuZ2VzIGluIEFDRSBvdmVyIHRoZSBzYW1wbGluZyBwZXJpb2Qgd2l0aGluIHRoZSBpbmRpdmlkdWFsIGRvbHBoaW4/DQpsbWUuQUNFIDwtIGxtZShBQ0UgfiB0aW1lLCByYW5kb20gPSB+MXxJRCwgZGF0YSA9IGFscGhhLmRvbHBoaW4ud2F0ZXIuZmlsLjkpDQoNCnN1bW1hcnkobG1lLkFDRSkNCmFub3ZhKGxtZS5BQ0UpDQoNCiMgICAgICAgICAgICAgbnVtREYgZGVuREYgICBGLXZhbHVlIHAtdmFsdWUNCiMoSW50ZXJjZXB0KSAgICAgMSAgICA1NCAyNTYuNDkzOTggIDwuMDAwMQ0KI3RpbWUgICAgICAgICAgIDE1ICAgIDU0ICAgMC45NjgwNyAgMC40OTk4DQoNCiMjIy0tPiBUaW1lIGRvZXMgbm8gaGF2ZSBhbiBlZmZlY3Qgb24gQUNFIGluIGluZGl2aWR1YWwgZG9scGhpbnMuDQoNCiMjIyMjIyMjIyMjIyMjIyMjIyMjIyMjIyMjDQojIyNJbmZsdWVuY2Ugb2YgSUQ6DQoNCiMgTW9kZWwgd2l0aCByYW5kb20gaW50ZXJjZXB0cw0KbW9kZWwxIDwtIGxtZShBQ0UgfiB0aW1lLCByYW5kb20gPSB+IDEgfCBJRCwgZGF0YSA9IGFscGhhLmRvbHBoaW4ud2F0ZXIuZmlsLjksDQogICAgICAgICAgICAgIG1ldGhvZCA9ICJSRU1MIikNCiMgTW9kZWwgd2l0aG91dCByYW5kb20gaW50ZXJjZXB0cw0KbW9kZWwwIDwtIGxtKEFDRSB+IHRpbWUsIGRhdGEgPSBhbHBoYS5kb2xwaGluLndhdGVyLmZpbC45KQ0KDQojIE9ic2VydmVkIGxpa2VsaWhvb2QgcmF0aW8gdGVzdCBzdGF0aXN0aWMNCm9icy5sciA8LSBhcy5udW1lcmljKC0yICogKGxvZ0xpayhtb2RlbDAsIFJFTUwgPSBUUlVFKSAtIGxvZ0xpayhtb2RlbDEsIFJFTUwgPSBUUlVFKSkpDQoNCiMgU2ltdWxhdGUgZGF0YSBmcm9tIHRoZSBtb2RlbCB3aXRob3V0IHJhbmRvbSBlZmZlY3RzDQpzaW1kYXQgPC0gc2ltdWxhdGUobW9kZWwwLCBuc2ltID0gMTAwMDAsIHNlZWQgPSAyMDIwMTEwNikNCg0KIyBGdW5jdGlvbiB0byBmaXQgYm90aCBtb2RlbHMgYW5kIGNhbGN1bGF0ZSB0aGUgTFIgc3RhdGlzdGljDQpjbXBfbW9kZWxzIDwtIGZ1bmN0aW9uKHkpIHsNCiAgbmRhdCA8LSBjYmluZChhbHBoYS5kb2xwaGluLndhdGVyLmZpbC45LCB5ID0geSkNCiAgbWRsMSA8LSBsbWUoeSB+IHRpbWUgLCByYW5kb20gPSB+IDEgfCBJRCwgZGF0YSA9IG5kYXQsIG1ldGhvZCA9ICJSRU1MIikNCiAgbWRsMCA8LSBsbSh5IH4gdGltZSAsIGRhdGEgPSBuZGF0KQ0KICBhMCA8LSBhcy5udW1lcmljKC0yICogKGxvZ0xpayhtZGwwLCBSRU1MID0gVFJVRSkgLSBsb2dMaWsobWRsMSwgUkVNTCA9IFRSVUUpKSkNCiAgYTANCn0NCg0KIyBHZXQgcGFyYW1ldHJpYyBib290c3RyYXAgdGVzdCBzdGF0aXN0aWNzDQpjbXAwIDwtIG1hcF9kYmwoc2ltZGF0LCBjbXBfbW9kZWxzKQ0KIyBHZXQgcGFyYW1ldHJpYyBib290c3RyYXAgcC12YWx1ZQ0KcGFyYS5wIDwtIG1lYW4oYyhjbXAwLCBvYnMubHIpID49IG9icy5scikNCnBhcmEucA0KDQoNCndyaXRlLmNzdihhbHBoYS5kb2xwaGluLndhdGVyLmZpbC44LCBmaWxlIDwtICdhbHBoYS5kb2xwaGluLndhdGVyLmZpbC44LmNzdicpDQp3cml0ZS5jc3YoYWxwaGEuZG9scGhpbi53YXRlci5maWwuOSwgZmlsZSA8LSAnYWxwaGEuZG9scGhpbi53YXRlci5maWwuOS5jc3YnKQ0KYGBgDQoNCiMjIyMjIyMjIyMjIyMjIyMjIyMjIyMjIyMjIyMjDQojIyNDcmVhdGUgc2NhdHRlcnBsb3RzIG9mIGFscGhhIGRpdmVyc2l0eSBwYXJhbWV0ZXJzIG9mIGVhY2ggZG9scGhpbiBhY3Jvc3MgdGltZQ0KYGBge3J9DQojIyNSaWNobmVzcw0KYWxwaGEuZG9scGhpbi53YXRlci5maWwuMTAgPC0gYWxwaGEuZG9scGhpbi53YXRlci5maWwuOA0KbmFtZXMoYWxwaGEuZG9scGhpbi53YXRlci5maWwuMTApWzZdIDwtICJ3ZWVrIg0KYWxwaGEuZG9scGhpbi53YXRlci5maWwuMTAkd2VlayA8LSBhcy5jaGFyYWN0ZXIoYWxwaGEuZG9scGhpbi53YXRlci5maWwuMTAkd2VlaykNCmFscGhhLmRvbHBoaW4ud2F0ZXIuZmlsLjEwJHdlZWsgPC0gYXMubnVtZXJpYyhhbHBoYS5kb2xwaGluLndhdGVyLmZpbC4xMCR3ZWVrKQ0KDQphbHBoYS5kb2xwaGluLndhdGVyLmZpbC4xMSA8LSBhbHBoYS5kb2xwaGluLndhdGVyLmZpbC45DQpuYW1lcyhhbHBoYS5kb2xwaGluLndhdGVyLmZpbC4xMSlbN10gPC0gIndlZWsiDQoNCmFscGhhLmRvbHBoaW4ud2F0ZXIuZmlsLjExJHdlZWsgPC0gYXMuY2hhcmFjdGVyKGFscGhhLmRvbHBoaW4ud2F0ZXIuZmlsLjExJHdlZWspDQphbHBoYS5kb2xwaGluLndhdGVyLmZpbC4xMSR3ZWVrIDwtIGFzLm51bWVyaWMoYWxwaGEuZG9scGhpbi53YXRlci5maWwuMTEkd2VlaykNCg0KUmljaG5lc3NfYWNyb3NzX3RpbWUgPC0gDQogIGdncGxvdChkYXRhID0gYWxwaGEuZG9scGhpbi53YXRlci5maWwuMTAsIGFlcyh4ID0gd2VlaywgeSA9IHJpY2huZXNzLCBncm91cD0gSUQsY29sb3IgPSBJRCkpICsgDQogIGdlb21fbGluZShsaW5ldHlwZSA9ICJkYXNoZWQiKSsNCiAgdGhlbWVfYncoKSsNCiAgZ2VvbV9wb2ludChzaXplID0gMikgKyANCiAgc2NhbGVfY29sb3JfbWFudWFsKHZhbHVlcyA9IGMoJ3BpbmsnLCd5ZWxsb3cnLCdkYXJrIGdyZWVuJywnb3JhbmdlJywnZGFyayBncmV5JywNCiAgICAgICAgICAgICAgICAgICAgICAgICAgICAgICAgJ2RhcmsgcmVkJywncHVycGxlJywnYmxhY2snLCdkYXJrc2FsbW9uJywNCiAgICAgICAgICAgICAgICAgICAgICAgICAgICAgICAgJ3R1cnF1b2lzZScsJ2RhcmsgYmx1ZScsICdsaWdodCBncmVlbicsICdyZWQnKSkNCg0KZ2dzYXZlKCJSaWNobmVzc19hY3Jvc3NfdGltZS5qcGciLCBwbG90ID0gUmljaG5lc3NfYWNyb3NzX3RpbWUgLCBkZXZpY2UgPSAnanBnJywgd2lkdGggPSAyMjAsIGhlaWdodCA9IDEzMCwgdW5pdHMgPSAibW0iLA0KICAgICAgIGRwaSA9IDMwMCwgbGltaXRzaXplID0gVFJVRSkNCg0KDQojIyNTaGFubm9uLVdpZW5lciBkaXZlcnNpdHkNCkRpdmVyc2l0eV9hY3Jvc3NfdGltZSA8LSANCiAgZ2dwbG90KGRhdGEgPSBhbHBoYS5kb2xwaGluLndhdGVyLmZpbC4xMCwgYWVzKHggPSB3ZWVrLCB5ID0gZGl2ZXJzaXR5LCBncm91cD0gSUQsY29sb3IgPSBJRCkpICsgDQogIGdlb21fbGluZShsaW5ldHlwZSA9ICJkYXNoZWQiKSsNCiAgdGhlbWVfYncoKSsNCiAgZ2VvbV9wb2ludChzaXplID0gMikgKyANCiAgc2NhbGVfY29sb3JfbWFudWFsKHZhbHVlcyA9IGMoJ3BpbmsnLCd5ZWxsb3cnLCdkYXJrIGdyZWVuJywnb3JhbmdlJywnZGFyayBncmV5JywNCiAgICAgICAgICAgICAgICAgICAgICAgICAgICAgICAgJ2RhcmsgcmVkJywncHVycGxlJywnYmxhY2snLCdkYXJrc2FsbW9uJywNCiAgICAgICAgICAgICAgICAgICAgICAgICAgICAgICAgJ3R1cnF1b2lzZScsJ2RhcmsgYmx1ZScsICdsaWdodCBncmVlbicsICdyZWQnKSkNCg0KZ2dzYXZlKGZpbGUgPSAiRGl2ZXJzaXR5X2Fjcm9zc190aW1lLmpwZyIsIHBsb3QgPSBEaXZlcnNpdHlfYWNyb3NzX3RpbWUgLCBkZXZpY2UgPSAnanBnJywgd2lkdGggPSAyMjAsIGhlaWdodCA9IDEzMCwgdW5pdHMgPSAibW0iLA0KICAgICAgIGRwaSA9IDMwMCwgbGltaXRzaXplID0gVFJVRSkNCg0KDQojIyNDaGFvMQ0KQ2hhbzFfYWNyb3NzX3RpbWUgPC0gDQogIGdncGxvdChkYXRhID0gYWxwaGEuZG9scGhpbi53YXRlci5maWwuMTAsIGFlcyh4ID0gd2VlaywgeSA9IENoYW8xLCBncm91cD0gSUQsY29sb3IgPSBJRCkpICsgDQogIGdlb21fbGluZShsaW5ldHlwZSA9ICJkYXNoZWQiKSsNCiAgdGhlbWVfYncoKSsNCiAgZ2VvbV9wb2ludChzaXplID0gMikgKyANCiAgc2NhbGVfY29sb3JfbWFudWFsKHZhbHVlcyA9IGMoJ3BpbmsnLCd5ZWxsb3cnLCdkYXJrIGdyZWVuJywnb3JhbmdlJywnZGFyayBncmV5JywNCiAgICAgICAgICAgICAgICAgICAgICAgICAgICAgICAgJ2RhcmsgcmVkJywncHVycGxlJywnYmxhY2snLCdkYXJrc2FsbW9uJywNCiAgICAgICAgICAgICAgICAgICAgICAgICAgICAgICAgJ3R1cnF1b2lzZScsJ2RhcmsgYmx1ZScsICdsaWdodCBncmVlbicsICdyZWQnKSkNCg0KZ2dzYXZlKGZpbGUgPSAiQ2hhbzFfYWNyb3NzX3RpbWUuanBnIiwgcGxvdCA9IENoYW8xX2Fjcm9zc190aW1lICwgZGV2aWNlID0gJ2pwZycsIHdpZHRoID0gMjIwLCBoZWlnaHQgPSAxMzAsIHVuaXRzID0gIm1tIiwNCiAgICAgICBkcGkgPSAzMDAsIGxpbWl0c2l6ZSA9IFRSVUUpDQoNCg0KIyMjQUNFDQpBQ0VfYWNyb3NzX3RpbWUgPC0gDQogIGdncGxvdChkYXRhID0gYWxwaGEuZG9scGhpbi53YXRlci5maWwuMTEsIGFlcyh4ID0gd2VlaywgeSA9IEFDRSwgZ3JvdXA9IElELGNvbG9yID0gSUQpKSArIA0KICBnZW9tX2xpbmUobGluZXR5cGUgPSAiZGFzaGVkIikrDQogIHRoZW1lX2J3KCkrDQogIGdlb21fcG9pbnQoc2l6ZSA9IDIpICsgDQogIHNjYWxlX2NvbG9yX21hbnVhbCh2YWx1ZXMgPSBjKCdwaW5rJywneWVsbG93JywnZGFyayBncmVlbicsJ29yYW5nZScsJ2RhcmsgZ3JleScsDQogICAgICAgICAgICAgICAgICAgICAgICAgICAgICAgICdkYXJrIHJlZCcsJ3B1cnBsZScsJ2JsYWNrJywnZGFya3NhbG1vbicsDQogICAgICAgICAgICAgICAgICAgICAgICAgICAgICAgICd0dXJxdW9pc2UnLCdkYXJrIGJsdWUnLCAnbGlnaHQgZ3JlZW4nLCAncmVkJykpDQoNCmdnc2F2ZShmaWxlID0gIkFDRV9hY3Jvc3NfdGltZS5qcGciLCBwbG90ID0gQUNFX2Fjcm9zc190aW1lICwgZGV2aWNlID0gJ2pwZycsIHdpZHRoID0gMjIwLCBoZWlnaHQgPSAxMzAsIHVuaXRzID0gIm1tIiwNCiAgICAgICBkcGkgPSAzMDAsIGxpbWl0c2l6ZSA9IFRSVUUpDQoNCmBgYA0KDQojIyMjIyMjIyMjIyMjIyMjIyMjIyMjIyMjIyMjIw0KIyMjQ2FsY3VsYXRlIGF2ZXJhZ2Ugb2YgYWxwaGEgZGl2ZXJzaXR5IHBhcmFtZXRlcnMgcGVyIGRvbHBoaW4NCmBgYHtyfQ0KIyMjUmljaG5lc3MNCmFscGhhLmRvbHBoaW4ud2F0ZXIuZmlsLmRvbC5yaWNobmVzcyA9IGFscGhhLmRvbHBoaW4ud2F0ZXIuZmlsLjlbLGMoMyw2KV0NCnJpY2huZXNzLmRvbC5hdmUgPSBhZ2dyZWdhdGUoYWxwaGEuZG9scGhpbi53YXRlci5maWwuZG9sLnJpY2huZXNzWzFdLCAgIGxpc3QoYWxwaGEuZG9scGhpbi53YXRlci5maWwuZG9sLnJpY2huZXNzJElEKSwgbWVhbikNCnJpY2huZXNzLmRvbC5zZCA9IGFnZ3JlZ2F0ZShhbHBoYS5kb2xwaGluLndhdGVyLmZpbC5kb2wucmljaG5lc3NbMV0sIGxpc3QoYWxwaGEuZG9scGhpbi53YXRlci5maWwuZG9sLnJpY2huZXNzJElEKSwgc2QpDQoNCiMjI0RpdmVyc2l0eQ0KYWxwaGEuZG9scGhpbi53YXRlci5maWwuZG9sLmRpdmVyc2l0eSA9IGFscGhhLmRvbHBoaW4ud2F0ZXIuZmlsLjlbLGMoNCw2KV0NCmRpdmVyc2l0eS5kb2wuYXZlID0gYWdncmVnYXRlKGFscGhhLmRvbHBoaW4ud2F0ZXIuZmlsLmRvbC5kaXZlcnNpdHlbMV0sIGxpc3QoYWxwaGEuZG9scGhpbi53YXRlci5maWwuZG9sLmRpdmVyc2l0eSRJRCksIG1lYW4pDQpkaXZlcnNpdHkuZG9sLnNkID0gYWdncmVnYXRlKGFscGhhLmRvbHBoaW4ud2F0ZXIuZmlsLmRvbC5kaXZlcnNpdHlbMV0sIGxpc3QoYWxwaGEuZG9scGhpbi53YXRlci5maWwuZG9sLmRpdmVyc2l0eSRJRCksIHNkKQ0KDQojIyNDaGFvMQ0KYWxwaGEuZG9scGhpbi53YXRlci5maWwuOC5DaGFvMSA9IGFscGhhLmRvbHBoaW4ud2F0ZXIuZmlsLjhbLGMoNSw5KV0NCkNoYW8xLmRvbC5hdmUgPSBhZ2dyZWdhdGUoYWxwaGEuZG9scGhpbi53YXRlci5maWwuOC5DaGFvMVsyXSwgbGlzdChhbHBoYS5kb2xwaGluLndhdGVyLmZpbC44LkNoYW8xJElEKSwgbWVhbikNCkNoYW8xLmRvbC5zZCA9IGFnZ3JlZ2F0ZShhbHBoYS5kb2xwaGluLndhdGVyLmZpbC44LkNoYW8xWzJdLCBsaXN0KGFscGhhLmRvbHBoaW4ud2F0ZXIuZmlsLjguQ2hhbzEkSUQpLCBzZCkNCg0KIyMjQUNFDQphbHBoYS5kb2xwaGluLndhdGVyLmZpbC5BQ0UgPSBhbHBoYS5kb2xwaGluLndhdGVyLmZpbC45WyxjKDIsNildDQpBQ0UuZG9sLmF2ZSA9IGFnZ3JlZ2F0ZShhbHBoYS5kb2xwaGluLndhdGVyLmZpbC5BQ0VbMV0sIGxpc3QoYWxwaGEuZG9scGhpbi53YXRlci5maWwuQUNFJElEKSwgbWVhbikNCkFDRS5kb2wuc2QgPSBhZ2dyZWdhdGUoYWxwaGEuZG9scGhpbi53YXRlci5maWwuQUNFWzFdLCBsaXN0KGFscGhhLmRvbHBoaW4ud2F0ZXIuZmlsLkFDRSRJRCksIHNkKQ0KDQphbHBoYS5wYXJhLmRvbC4yID0gY2JpbmQocmljaG5lc3MuZG9sLmF2ZSxyaWNobmVzcy5kb2wuc2QsZGl2ZXJzaXR5LmRvbC5hdmUsZGl2ZXJzaXR5LmRvbC5zZCxDaGFvMS5kb2wuYXZlLENoYW8xLmRvbC5zZCxBQ0UuZG9sLmF2ZSxBQ0UuZG9sLnNkKQ0Kd3JpdGUuY3N2KGFscGhhLnBhcmEuZG9sLjIsIGZpbGUgPSAnYWxwaGEucGFyYS5kb2wuMy5jc3YnKQ0KYGBgDQoNCiMjIyMjIyMjIyMjIyMjIyMjIyMNCiMjI0RpZCB0aW1lIGhhdmUgYW4gZWZmZWN0IG9uIHRoZSBtaWNyb2JpYWwgY29tbXVuaXRpZXMgaW4gdGhlIGRvbHBoaW4gYmxvdz8NCmBgYHtyfQ0KIyMjRml0IHRoZSBnZW5lcmFsaXNlZCBsaW5lYXIgbW9kZWxzDQp6T1RVLndhdGVyLmZpbCA9IG12YWJ1bmQoZGF0LmRvbF93YXRlci5maWwudC45Wyw0OjE0NzRdKQ0KDQojIyNDcmVhdGUgdmFyaWFibGUgZm9yIGxvZyBvZiB0b3RhbCBhYnVuZGFuY2Ugb2Ygek9UVSdzIHBlciBzYW1wbGUNCmRhdC5kb2xfd2F0ZXIuZmlsLnQuMTAgPSBkYXQuZG9sX3dhdGVyLmZpbC50LjkNCmRhdC5kb2xfd2F0ZXIuZmlsLnQuMTAkbG9nVG90YWxBYnVuZGFuY2UgPSBsb2coYXBwbHkoZGF0LmRvbF93YXRlci5maWwudC4xMFssNDoxNDc0XSwxLHN1bSkpDQoNClByZS5maXQxLndhdGVyLmZpbC50aW1lID0gbWFueWdsbSh6T1RVLndhdGVyLmZpbCB+IHRpbWUgKyBJRCArIG9mZnNldChsb2dUb3RhbEFidW5kYW5jZSksIGRhdGEgPSBkYXQuZG9sX3dhdGVyLmZpbC50LjEwLCBmYW1pbHk9Im5lZ2F0aXZlLmJpbm9taWFsIikNCg0KI2NoZWNrIGFzc3VtcHRpb25zDQpwbG90KFByZS5maXQxLndhdGVyLmZpbC50aW1lKQ0KDQojVGhpcyBpcyB0aGUgYWN0dWFsIHNpZ25pZmljYW5jZSB0ZXN0ICAgICAgICAgICAgICAgDQpmaXQuMS53YXRlci5maWwudGltZS4xID0gbWFueWdsbSh6T1RVLndhdGVyLmZpbCB+IHRpbWUgKyBJRCArIG9mZnNldChsb2dUb3RhbEFidW5kYW5jZSksIGRhdGEgPSBkYXQuZG9sX3dhdGVyLmZpbC50LjEwKQ0KZml0LjIud2F0ZXIuZmlsLnRpbWUuMiA9IG1hbnlnbG0oek9UVS53YXRlci5maWwgfiBJRCArIG9mZnNldChsb2dUb3RhbEFidW5kYW5jZSksIGRhdGEgPSBkYXQuZG9sX3dhdGVyLmZpbC50LjEwKQ0KDQpnbG9iYWxUZXN0MS5kb2wud2F0ZXIuZmlsLmFkanVzdGVkLnRpbWUgPSBhbm92YShmaXQuMS53YXRlci5maWwudGltZS4xLCBmaXQuMi53YXRlci5maWwudGltZS4yLCBuQm9vdD0xMDAwLCBwLnVuaT0nYWRqdXN0ZWQnKQ0KDQojIEFuYWx5c2lzIG9mIERldmlhbmNlIFRhYmxlDQojIA0KIyBmaXQuMi53YXRlci5maWwudGltZS4yOiB6T1RVLndhdGVyLmZpbCB+IElEICsgb2Zmc2V0KGxvZ1RvdGFsQWJ1bmRhbmNlKQ0KIyBmaXQuMS53YXRlci5maWwudGltZS4xOiB6T1RVLndhdGVyLmZpbCB+IHRpbWUgKyBJRCArIG9mZnNldChsb2dUb3RhbEFidW5kYW5jZSkNCiMgDQojIE11bHRpdmFyaWF0ZSB0ZXN0Og0KIyAgICAgICAgICAgICAgICAgICAgICAgIFJlcy5EZiBEZi5kaWZmICAgRGV2IFByKD5EZXYpICAgDQojIGZpdC4yLndhdGVyLmZpbC50aW1lLjIgICAgIDcwICAgICAgICAgICAgICAgICAgICAgICAgICANCiMgZml0LjEud2F0ZXIuZmlsLnRpbWUuMSAgICAgNTUgICAgICAxNSAyMDE0MCAgICAwLjAwMiAqKg0KIyAtLS0NCiMgU2lnbmlmLiBjb2RlczogIDAg4oCYKioq4oCZIDAuMDAxIOKAmCoq4oCZIDAuMDEg4oCYKuKAmSAwLjA1IOKAmC7igJkgMC4xIOKAmCDigJkgMQ0KDQojc2F2ZShnbG9iYWxUZXN0MS5kb2wud2F0ZXIuZmlsLmFkanVzdGVkLnRpbWUsIGZpbGU9J2dsb2JhbFRlc3QxLmRvbC53YXRlci5maWwuYWRqdXN0ZWQudGltZS5SZGF0YScpDQpgYGANCg0KIyMjIyMjVGhpcyB0ZWxscyB1cyB0aGF0IHRoZXJlIGlzIGFuIGltcGFjdCBvZiB0aW1lIyMjIyMjIyMgDQoNCiMjIyMjIyMjIyMjIyMjIyMjIyMjIyMjIyMjIyMjIyMjIyMjIyMjIyMjIyMjIyMjIyMjIyMjIyMNCiMjIyMjIyMjIyMjI0RpZCBJRCBoYXZlIGFuIGVmZmVjdCBvbiB0aGUgbWljcm9iaWFsIGNvbW11bml0aWVzIGluIHRoZSBkb2xwaGluIGJsb3c/DQpgYGB7cn0NCiNUaGlzIGlzIHRoZSBhY3R1YWwgc2lnbmlmaWNhbmNlIHRlc3QgDQpmaXQuMS53YXRlci5maWwuSUQuMSA9IG1hbnlnbG0oek9UVS53YXRlci5maWwgfiB0aW1lICsgSUQgKyBvZmZzZXQobG9nVG90YWxBYnVuZGFuY2UpLCBkYXRhID0gZGF0LmRvbF93YXRlci5maWwudC4xMCkNCmZpdC4yLndhdGVyLmZpbC5JRC4yID0gbWFueWdsbSh6T1RVLndhdGVyLmZpbCB+IHRpbWUgKyBvZmZzZXQobG9nVG90YWxBYnVuZGFuY2UpLCBkYXRhID0gZGF0LmRvbF93YXRlci5maWwudC4xMCkNCg0KZ2xvYmFsVGVzdDEuZG9sLndhdGVyLmZpbC5hZGp1c3RlZC5JRCA9IGFub3ZhKGZpdC4xLndhdGVyLmZpbC5JRC4xLCBmaXQuMi53YXRlci5maWwuSUQuMiwgbkJvb3Q9MTAwMCwgcC51bmk9J2FkanVzdGVkJykNCg0KIyBBbmFseXNpcyBvZiBEZXZpYW5jZSBUYWJsZQ0KIyANCiMgZml0LjIud2F0ZXIuZmlsLklELjI6IHpPVFUud2F0ZXIuZmlsIH4gdGltZSArIG9mZnNldChsb2dUb3RhbEFidW5kYW5jZSkNCiMgZml0LjEud2F0ZXIuZmlsLklELjE6IHpPVFUud2F0ZXIuZmlsIH4gdGltZSArIElEICsgb2Zmc2V0KGxvZ1RvdGFsQWJ1bmRhbmNlKQ0KIyANCiMgTXVsdGl2YXJpYXRlIHRlc3Q6DQojICAgICAgICAgICAgICAgICAgICAgIFJlcy5EZiBEZi5kaWZmICAgRGV2IFByKD5EZXYpICAgIA0KIyBmaXQuMi53YXRlci5maWwuSUQuMiAgICAgNjcgICAgICAgICAgICAgICAgICAgICAgICAgICANCiMgZml0LjEud2F0ZXIuZmlsLklELjEgICAgIDU1ICAgICAgMTIgNDA2MzIgICAgMC4wMDEgKioqDQoNCiNzYXZlKGdsb2JhbFRlc3QxLmRvbC53YXRlci5maWwuYWRqdXN0ZWQuSUQsIGZpbGU9J2dsb2JhbFRlc3QxLmRvbC53YXRlci5maWwuYWRqdXN0ZWQuSUQuUmRhdGEnKQ0KYGBgDQoNCiMjIyMjI1RoaXMgdGVsbHMgdXMgdGhhdCB0aGVyZSBpcyBhbiBpbXBhY3Qgb2YgSUQsIGJ1dCBpdCBkb2Vzbid0IHRlbGwgdXMgaWYgdGhlIGRpZmZlcmVuY2VzIGJldHdlZW4gZG9scGhpbnMgc3RheSBjb25zdGFudCAoRWZmZWN0IG9mIGRvbHBoaW4gaXMgbGFyZ2VyIHRoYW4gZWZmZWN0IG9mIHRpbWUpIyMjIyMjIyMgDQoNCiMjIyMjIyMjIyMjIyMjIyMjIyMjIyMjIyMjIyMjIyMjIyMjIyMjIyMjIyMjIyMjIyMjIyMjIyMjIyMjIyMjIyMjIyMjIw0KIyMjIyMjIyMjIyMjQ2hlY2sgaWYgdGhlIGVmZmVjdCBvZiBJRCBpcyBsYXJnZXIgdGhhbiB0aGF0IG9mIHRpbWUNCiMjI1ZhcmlhbmNlIGNvbXBvbmVudCBhbmFseXNpcw0KYGBge3J9DQojIyNVc2UgLQlmYWtlX2RvbHBoaW5fZXhhbXBsZV9mb3JfQ2F0IGZyb20gTWFyaw0KDQojIFJlc2hhcGUgdGhpcyBpbnRvIGEgbG9uZyBkYXRhc2V0LCB3aXRoIG9uZSByb3cgZm9yIGV2ZXJ5IHpPVFUNCiMgRmlyc3QgeW91IG5lZWQgYSBsaXN0IG9mIGFsbCBvZiB0aGUgbmFtZXMgb2YgdGhlIHpPVFUgY29sdW1ucw0KZGltKGRhdC5kb2xfd2F0ZXIuZmlsLnQuOSkNCiM4MyAxNDc0DQoNCiMgUmVzaGFwZSB0aGlzIGludG8gYSBsb25nIGRhdGFzZXQsIHdpdGggb25lIHJvdyBmb3IgZXZlcnkgek9UVQ0KIyBGaXJzdCB5b3UgbmVlZCBhIGxpc3Qgb2YgYWxsIG9mIHRoZSBuYW1lcyBvZiB0aGUgek9UVSBjb2x1bW5zDQpzcG5hbWVzIDwtIGNvbG5hbWVzKGRhdC5kb2xfd2F0ZXIuZmlsLnQuOVs0OjE0NzRdKQ0KDQpkYXQuZG9sX3dhdGVyLmZpbC50LjUwIDwtIGRhdC5kb2xfd2F0ZXIuZmlsLnQuOQ0KZGF0LmRvbF93YXRlci5maWwudC41MCREb2xwaGluIDwtIGFzLmZhY3RvcihkYXQuZG9sX3dhdGVyLmZpbC50LjUwJERvbHBoaW4pDQpkYXQuZG9sX3dhdGVyLmZpbC50LjUwJElEIDwtIGFzLmZhY3RvcihkYXQuZG9sX3dhdGVyLmZpbC50LjUwJElEKQ0KZGF0LmRvbF93YXRlci5maWwudC41MCR0aW1lIDwtIGFzLmZhY3RvcihkYXQuZG9sX3dhdGVyLmZpbC50LjUwJHRpbWUpDQoNCiMgRG8gdGhlIHJlc2hhcGluZw0KRG9scGhpbkxvbmcgPC0gZGF0LmRvbF93YXRlci5maWwudC41MCAlPiUgDQogIG11dGF0ZShpZCA9IGZhY3Rvcihyb3dfbnVtYmVyKCkpKSAlPiUgDQogIHBpdm90X2xvbmdlcihjb2xzID0gc3BuYW1lcywgbmFtZXNfdG8gPSAic3BlY2llcyIsDQogICAgICAgICAgICAgICB2YWx1ZXNfdG8gPSAieSIpDQoNCiMgRml0IHRoZSBtb2RlbCAobWF5IHRha2Ugc29tZSB0aW1lIHdpdGggYSBiaWcgZGF0YXNldCkNCiAgICAgICAgICAgICAgICAgICAgDQptZGwuZ2xtbSA8LSBnbG1tVE1CKHkgfiBzcGVjaWVzICsgKDF8RG9scGhpbikgKw0KICAgICAgICAgICAgICAgICAgICAgICgxIHwgc3BlY2llczpJRCkgKw0KICAgICAgICAgICAgICAgICAgICAgICgxIHwgc3BlY2llczp0aW1lKSwNCiAgICAgICAgICAgICAgICAgICAgZmFtaWx5ID0gbmJpbm9tMigpLCBkYXRhID0gRG9scGhpbkxvbmcsDQogICAgICAgICAgICAgICAgICAgIHNwYXJzZVggPSBjKGNvbmQ9VFJVRSkpDQoNCiMgSW50ZXJlc3QgaXMgaW4gdGhlIHJlbGF0aXZlIHZhcmlhbmNlcyBvZiB0aGUgc3BlY2llczpkb2xwaGluIGFuZCBzcGVjaWVzOnRpbWUuY2F0IHJhbmRvbSBlZmZlY3RzDQpWYXJDb3JyKG1kbC5nbG1tKQ0KIyBUaGVzZSBpbmRpY2F0ZSB0aGUgbWFnbml0dWRlIHRvIHdoaWNoIGVhY2ggdmFyaWFibGUgaXMgcmVzcG9uc2libGUgZm9yIHRoZSB2YXJpYW5jZSBiZXR3ZWVuIHNhbXBsZXMNCg0KcHVycnI6Om1hcChtZGwuZ2xtbSRtb2RlbEluZm8kcmVUcm1zJGNvbmQkZmxpc3QsIG5sZXZlbHMpDQpzdHIoZGF0LmRvbF93YXRlci5maWwudC41MCkNCg0KYGBgDQoNCiMjIyMjIyMjIyMjIyMjIyMjIyMjIyMjIyMjIyMjIyMjIyMjIyMjDQojIyMjIyMjIyMjIyMjMS4JRGV2aWFuY2Ug4oCYZXhwbGFpbmVk4oCZLiBUaGUgaWRlYSBoZXJlIGlzIHRoYXQgeW91IGNhbiBsb29rIGF0IA0KIw0KIzEuCTEpIGhvdyB3ZWxsIGtub3dpbmcgdGhlIGRvbHBoaW4gSUQgaGVscHMgeW91IGV4cGxhaW4gdGhlIHZhcmlhbmNlIGJldHdlZW4gc2FtcGxlcyB0YWtlbiAjYXQgdGhlIHNhbWUgdGltZSwgdmVyc3VzIA0KIygyKSBob3cgd2VsbCBrbm93aW5nIHRoZSB0aW1lIGhlbHBzIHlvdSBleHBsYWluIHRoZSB2YXJpYW5jZSBiZXR3ZWVuIHNhbXBsZXMgdGFrZW4gZnJvbSB0aGUgI3NhbWUgZG9scGhpbi4gDQojSWYgKDEpIGlzIG11Y2ggbGFyZ2VyIHRoYW4gKDIpLCB0aGVuIHlvdSBjb3VsZCBhcmd1ZSB0aGF0IHNhbXBsZXMgZnJvbSB0aGUgc2FtZSBkb2xwaGluICNhY3Jvc3MgdGltZSBhcmUgbW9yZSBzaW1pbGFyIHRoYW4gc2FtcGxlcyBmcm9tIGRpZmZlcmVudCBkb2xwaGlucyBhdCB0aGUgc2FtZSB0aW1lLCB3aGljaCBJICN0aGluayBpcyB5b3VyIGFyZ3VtZW50PyANCiNJIGhhdmUgYXR0YWNoZWQgc29tZSBSIGNvZGUgdGhhdCB1c2VzIG9uZSBvZiB0aGUgZXhhbXBsZSBkYXRhc2V0cyBpbiB0aGUgbXZhYnVuZCBwYWNrYWdlIHRvICNkbyB0aGlzOyBmb3IgeW91ciBkYXRhIHlvdSB3b3VsZCBvYnZpb3VzbHkgdXNlIGRvbHBoaW5JRCBhbmQgdGltZSBpbnN0ZWFkIG9mIHNvaWwgYW5kIHNhbmQuICNPbmUgdHJpY2t5IHRoaW5nIGlzIHRoYXQgd2hlbiBjb25zaWRlcmluZyB0d28gY2F0ZWdvcmljYWwgdmFyaWFibGVzIChJIHdhc27igJl0IHN1cmUgaWYgdGltZSAjaXMgY2F0ZWdvcmljYWwgaW4geW91ciBtb2RlbCwgYnV0IHRoZSBwb2ludCBzdGFuZHMgZWl0aGVyIHdheSksIHRoZSBvbmUgd2l0aCBtb3JlIGxldmVscyBpcyAjZXhwZWN0ZWQgdG8gZXhwbGFpbiBtb3JlIG9mIHRoZSBkZXZpYW5jZSwgZXZlbiBpZiB0aGUgdHdvIHZhcmlhYmxlcyBhcmUgZXF1YWxseSBpbXBvcnRhbnQuICNUaGlzIGlzIHRoZSBwb2ludCBvZiB0aGUg4oCcRGl2aWRlIGJ5IGRlZ3JlZXMgb2YgZnJlZWRvbeKAnSBsaW5lcywgd2hpY2ggdGFrZSB0aGlzIGludG8gYWNjb3VudCAjLS0tIGFsYmVpdCBpbiBhIHZlcnkgdmVyeSByb3VnaCB3YXkgLS0tIHdoaWNoIHNob3VsZCBnaXZlIHR3byBtZWFzdXJlcyBvZiBkZXZpYW5jZSBleHBsYWluZWQgI3RoYXQgYXJlIGNvbXBhcmFibGUgdG8gZWFjaCBvdGhlci4gVGhlIHBzZXVkbyBSMnMgaGF2ZSB0aGUgc2FtZSBwcm9ibGVtIGFzIHRoZSB1bnNjYWxlZCAjZGV2aWFuY2VzLiBBZ2FpbiwgYmVjYXVzZSB0aGVzZSBhcmUgcm91Z2ggbWVhc3VyZXMsIHlvdSB3b3VsZCBvbmx5IGJlIGFibGUgdG8gcmVhbGx5IGRyYXcgYSAjY29uY2x1c2lvbiBpZiB0aGVyZSBpcyBhIGJpZyBkaWZmZXJlbmNlIGJldHdlZW4gdGhlbS4NCg0KYGBge3J9DQpkYXQuZG9sX3dhdGVyLmZpbC50LjkubXZhYnVuZCA8LSBtdmFidW5kKGRhdC5kb2xfd2F0ZXIuZmlsLnQuOVssNDoxNDc0XSkNCg0KZ2xtLmRvbC5udWxsIDwtIG1hbnlnbG0oZGF0LmRvbF93YXRlci5maWwudC45Lm12YWJ1bmQgfiAxLCBmYW1pbHkgPSAibmVnYXRpdmUuYmlub21pYWwiKQ0KDQpnbG0uZG9sLklEIDwtIG1hbnlnbG0oZGF0LmRvbF93YXRlci5maWwudC45Lm12YWJ1bmQgfiBJRCwgZGF0YSA9IGRhdC5kb2xfd2F0ZXIuZmlsLnQuOSwgZmFtaWx5ID0gIm5lZ2F0aXZlLmJpbm9taWFsIikNCg0KZ2xtLmRvbC50aW1lIDwtIG1hbnlnbG0oZGF0LmRvbF93YXRlci5maWwudC45Lm12YWJ1bmQgfiB0aW1lLCBkYXRhID0gZGF0LmRvbF93YXRlci5maWwudC45LCBmYW1pbHkgPSAibmVnYXRpdmUuYmlub21pYWwiKQ0KDQpnbG0uZG9sLmZ1bGwgPC0gbWFueWdsbShkYXQuZG9sX3dhdGVyLmZpbC50LjkubXZhYnVuZCB+IElEICsgdGltZSwgZGF0YSA9IGRhdC5kb2xfd2F0ZXIuZmlsLnQuOSwgZmFtaWx5ID0gIm5lZ2F0aXZlLmJpbm9taWFsIikNCg0KIyBUb3RhbCBkZXZpYW5jZSB0byBiZSBleHBsYWluZWQNCnN1bShnbG0uZG9sLm51bGwkZGV2aWFuY2UpDQojNDYsMTgyLjIxDQoNCiMgRGV2aWFuY2UgZXhwbGFpbmVkIGJ5IElELCBhZnRlciBjb25kaXRpb25pbmcgb24gdGltZQ0Kc3VtKGdsbS5kb2wudGltZSRkZXZpYW5jZSAtIGdsbS5kb2wuZnVsbCRkZXZpYW5jZSkNCiM0LDQ3MC4zNzgNCg0KIyBEaXZpZGUgYnkgZGVncmVlcyBvZiBmcmVlZG9tICgxIGluIHRoaXMgY2FzZSwgYnV0IHdpbGwgYmUgSyAtIDEgZm9yIGNhdGVnb3JpY2FsIHZhcmlhYmxlIHdpdGggSyBsZXZlbHMpDQpzdW0oZ2xtLmRvbC50aW1lJGRldmlhbmNlIC0gZ2xtLmRvbC5mdWxsJGRldmlhbmNlKSAvIChucm93KGdsbS5kb2wuZnVsbCRjb2VmZmljaWVudHMpIC0gbnJvdyhnbG0uZG9sLnRpbWUkY29lZmZpY2llbnRzKSkNCiMzNzIuNTMxNQ0KDQojIERldmlhbmNlIGV4cGxhaW5lZCBieSB0aW1lLCBhZnRlciBjb25kaXRpb25pbmcgb24gSUQNCnN1bShnbG0uZG9sLklEJGRldmlhbmNlIC0gZ2xtLmRvbC5mdWxsJGRldmlhbmNlKQ0KIzI1OTEuNDc1DQoNCiMgRGl2aWRlIGJ5IGRlZ3JlZXMgb2YgZnJlZWRvbSAoMSBpbiB0aGlzIGNhc2UsIGJ1dCB3aWxsIGJlIEsgLSAxIGZvciBjYXRlZ29yaWNhbCB2YXJpYWJsZSB3aXRoIEsgbGV2ZWxzKQ0Kc3VtKGdsbS5kb2wuSUQkZGV2aWFuY2UgLSBnbG0uZG9sLmZ1bGwkZGV2aWFuY2UpIC8gKG5yb3coZ2xtLmRvbC5mdWxsJGNvZWZmaWNpZW50cykgLSBucm93KGdsbS5kb2wuSUQkY29lZmZpY2llbnRzKSkNCiMxNzIuNzY1DQoNCiMgUHNldWRvIFJeMiBmb3IgSUQNCnN1bShnbG0uZG9sLnRpbWUkZGV2aWFuY2UgLSBnbG0uZG9sLmZ1bGwkZGV2aWFuY2UpIC8gc3VtKGdsbS5kb2wudGltZSRkZXZpYW5jZSkNCiMwLjA5OTAyNzE5DQoNCiMgUHNldWRvIFJeMiBmb3IgdGltZQ0Kc3VtKGdsbS5kb2wuSUQkZGV2aWFuY2UgLSBnbG0uZG9sLmZ1bGwkZGV2aWFuY2UpIC8gc3VtKGdsbS5kb2wuSUQkZGV2aWFuY2UpDQojMC4wNTk4OTkwNQ0KYGBgDQoNCiMjIyMjIyMjIyMjIyMjIyMjIyMjIyMjIyMjIyMjIyMjIyMjIyMjIyMjIw0KIyMjIyMjIyMjSG93IG1hbnkgSW50cmEtY29yZSBkaWQgdGhlIGRvbHBoaW4gaGFyYm91cj8gKEludHJhLWNvcmU6IHpPVFVzIHRoYXQgcGVyc2lzdGVkIGluIGVhY2ggaW5kaXZpZHVhbCBkb2xwaGluIG92ZXIgZm91ciBzYW1wbGluZyBvY2Nhc2lvbnMgb3ZlciBmaXZlIG1vbnRocykNCmBgYHtyfQ0KIyMjIyMjIyMjIyMjIyMjIyMjIyMjICMjIyMjIyMjIyMjIyMjIyMjIyMjIyMgIyMjIyMjIyMjIyMjIyMjIyMjIyMjIyAjIyMjIyMjIyMjIyMjIyMjIyMjIyMjICMjIyMjIyMjIyMjIyMjIyMjIyMjIyMNCiMjI0ludHJhLWNvcmUgb2YgZnVsbCBjb3VudHMgKHF1YWxpdGF0aXZlIGNvcmUgYW5hbHlzaXMpIHdpdGggb25seSBmb3VyIHNhbXBsZXMgaW5jbHVkZWQNCg0KIyMjIyMjIyMjIyMjDQojIyNEZXRlcm1pbmUgMTAwJSBjb3JlIG9mIGRvbHBoaW5zIG9mIFVOcmFyZWZpZWQgZGF0YSBpbiB3ZWVrIDYsMTEsMTksMjgNCiNkYXQuZG9sX3dhdGVyLmZpbC50LjINCg0KZGltKGRhdC5kb2xfd2F0ZXIuZmlsLnQuMikNCiM4MyAxNDcxDQoNCiMjI0RlbGV0ZSBhbGwgZG9scGhpbiBzYW1wbGVzIGFwYXJ0IGZyb20gd2VlayA2LDExLDE5LDI4IChDb2VuLEV2aWUsS2lhbWEsTW9raSxSQiwNCiNTY29vdGVyLFNpcml1cyxTcXVlYWssU3RhcmJ1Y2ssSG93aWUsTnVkZ2VlKQ0KDQpkYXQuZG9sX3dhdGVyLmZpbC40c2FtcC5jb3JlID0gZGF0LmRvbF93YXRlci5maWwudC4yWy1jKDEsNSw3LDExLDEzOjE3LDE4LDIwLDIyLDI0LDI1LDMwLDMyLDM2LDM4LDQwLCA0Miw0NCw0Niw0Nyw1MSw1Myw1OCw2Miw2NCw2OSw3Myw3NSw3Nzo4MyksXQ0KDQpgYGANCg0KIyMjQ29lbg0KYGBge3J9DQojIyNDb2VuDQojQ2FsY3VsYXRlIGNvcmUgdGF4YSB0aGF0IDEwMCAlIG9mIENvZW4ncyBzYW1wbGVzIGhhdmUgaW4gY29tbW9uDQojZGF0LmRvbF93YXRlci5maWwuNHNhbXAuY29yZQ0KDQpkYXQuZG9sX3dhdGVyLmZpbC40c2FtcC5jb3JlLkNvZW4gPC0gIGRhdC5kb2xfd2F0ZXIuZmlsLjRzYW1wLmNvcmVbYygxOjQpLF0NCg0KZGltKGRhdC5kb2xfd2F0ZXIuZmlsLjRzYW1wLmNvcmUuQ29lbikNCiM0IDE1NzENCg0KZGF0LmRvbF93YXRlci5maWwuNHNhbXAuY29yZS5iaW5hcnkuQ29lbiA8LSBkYXQuZG9sX3dhdGVyLmZpbC40c2FtcC5jb3JlLkNvZW4NCg0KI0NvbnZlcnQgZGF0YWZyYW1lIGludG8gYmluYXJ5DQpkYXQuZG9sX3dhdGVyLmZpbC40c2FtcC5jb3JlLmJpbmFyeS5Db2VuW10gPC0gKyhkYXQuZG9sX3dhdGVyLmZpbC40c2FtcC5jb3JlLmJpbmFyeS5Db2VuICA+IDApDQojVmlldyhkYXQuZG9sX3dhdGVyLmZpbC40c2FtcC5jb3JlLmJpbmFyeS5Db2VuIFssMToxMF0pDQoNCiNUcmFuc3Bvc2UgZGF0LmRvbF93YXRlci5maWwuNHNhbXAuY29yZS5iaW5hcnkuQ29lbg0KZGF0LmRvbF93YXRlci5maWwuNHNhbXAuY29yZS5iaW5hcnkuQ29lbl90IDwtIGFzLmRhdGEuZnJhbWUodChkYXQuZG9sX3dhdGVyLmZpbC40c2FtcC5jb3JlLmJpbmFyeS5Db2VuKSkNCiNWaWV3KGRhdC5kb2xfd2F0ZXIuZmlsLjRzYW1wLmNvcmUuYmluYXJ5LkNvZW5fdFsxOjEwLF0pDQoNCiNDcmVhdGUgY29sIFRvdGFsLnJlbC5hYnVuZC4NCmRhdC5kb2xfd2F0ZXIuZmlsLjRzYW1wLmNvcmUuYmluYXJ5LkNvZW5fdC4yIDwtIGRhdC5kb2xfd2F0ZXIuZmlsLjRzYW1wLmNvcmUuYmluYXJ5LkNvZW5fdCAlPiUgDQogIG11dGF0ZShUb3RhbC5yZWwuYWJ1bmQuek9UVSA9IHJvd1N1bXMoZGF0LmRvbF93YXRlci5maWwuNHNhbXAuY29yZS5iaW5hcnkuQ29lbl90KS80KQ0KDQojVmlldyhkYXQuZG9sX3dhdGVyLmZpbC40c2FtcC5jb3JlLmJpbmFyeS5Db2VuX3QuMlsxOjEwLF0pDQoNCiNHaXZlIGRhdC5kb2xfd2F0ZXIuZmlsLjRzYW1wLmNvcmUuYmluYXJ5LkNvZW5fdC4yIHJvd25hbWVzIGFzIGNvbCB2YXJpYWJsZQ0KZGF0LmRvbF93YXRlci5maWwuNHNhbXAuY29yZS5iaW5hcnkuQ29lbl90LjIkdmFyaWFibGUgPC0gcm93bmFtZXMoZGF0LmRvbF93YXRlci5maWwuNHNhbXAuY29yZS5iaW5hcnkuQ29lbl90KQ0KZGltKGRhdC5kb2xfd2F0ZXIuZmlsLjRzYW1wLmNvcmUuYmluYXJ5LkNvZW5fdC4yKQ0KIzE0NzEgNg0KDQojUmVvcmRlcg0KZGF0LmRvbF93YXRlci5maWwuNHNhbXAuY29yZS5iaW5hcnkuQ29lbl90LjMgPC0gZGF0LmRvbF93YXRlci5maWwuNHNhbXAuY29yZS5iaW5hcnkuQ29lbl90LjJbLGMoNSw2LDE6NCldDQoNCiNKb2luIGRhdC5kb2xfd2F0ZXIuZmlsLjRzYW1wLmNvcmUuYmluYXJ5LkNvZW5fdC4zIHdpdGggYXR0cmlidXRlcy5taWdyYXRpb25fcmRwDQpDb3JlLnpPVFUuQ29lbi40c2FtcCA8LSBkYXQuZG9sX3dhdGVyLmZpbC40c2FtcC5jb3JlLmJpbmFyeS5Db2VuX3QuMyAlPiUgbGVmdF9qb2luKGF0dHJpYnV0ZXMuZG9sX3JkcF93YXRlci5maWwuMikNCmRpbShDb3JlLnpPVFUuQ29lbi40c2FtcCkNCiMgMTQ3MSAgIDEyDQoNCiNWaWV3KENvcmUuek9UVS5Db2VuLjRzYW1wWzE6MTAsXSkNCg0KI1Jlb3JkZXINCkNvcmUuek9UVS5Db2VuLjRzYW1wXzIgPSBDb3JlLnpPVFUuQ29lbi40c2FtcFssYygxLDIsMTEsMzo2KV0NCmRpbShDb3JlLnpPVFUuQ29lbi40c2FtcF8yKQ0KIyMxNDcxICAgIDcNCg0KI25hbWVzKENvcmUuek9UVS5Db2VuLjRzYW1wXzIpDQoNCiNPcmRlciBhY2NvcmRpbmcgdG8gQ29yZS56T1RVLkNvZW4uNHNhbXBfMiRUb3RhbC5yZWwuYWJ1bmQuek9UVQ0KQ29yZS56T1RVLkNvZW4uNHNhbXBfMyA9IENvcmUuek9UVS5Db2VuLjRzYW1wXzJbb3JkZXIoQ29yZS56T1RVLkNvZW4uNHNhbXBfMiRUb3RhbC5yZWwuYWJ1bmQuek9UVSksXQ0KI1ZpZXcoQ29yZS56T1RVLkNvZW4uNHNhbXBfM1ssMToyXSkNCg0KI0FueSBjb3JlT1RVcyBhYm92ZSAxMDAlICg9PSAxLjApPw0KQ29yZS56T1RVLkNvZW4uNHNhbXBfMS4wID0gQ29yZS56T1RVLkNvZW4uNHNhbXBfMyAlPiUgZmlsdGVyIChUb3RhbC5yZWwuYWJ1bmQuek9UVSA9PSAxLjApDQpkaW0oQ29yZS56T1RVLkNvZW4uNHNhbXBfMS4wKQ0KIzIxNyA3DQoNCiMjIyMjIyMNCiMjI0hvdyBtYW55IHpPVFVzIGFyZSBwcmVzZW50IHBlciBDb2VuJ3Mgc2FtcGxlIG9uIGF2ZXJhZ2U/DQpkYXQuZG9sX3dhdGVyLmZpbC40c2FtcC5jb3JlLmJpbmFyeS5Db2VuXzIgPSBkYXQuZG9sX3dhdGVyLmZpbC40c2FtcC5jb3JlLmJpbmFyeS5Db2VuICU+JSBtdXRhdGUoc3VtX29mX2NvdW50cyA9IHJvd1N1bXMoZGF0LmRvbF93YXRlci5maWwuNHNhbXAuY29yZS5iaW5hcnkuQ29lbikpDQoNCm1lYW4oZGF0LmRvbF93YXRlci5maWwuNHNhbXAuY29yZS5iaW5hcnkuQ29lbl8yJHN1bV9vZl9jb3VudHMpDQojMzUxDQoNCiMjIyMjIyMNCiNXaGF0J3MgdGhlIHByZXZhbGVuY2Ugb2YgdGhlIGNvcmUgek9UVXMgaW4gQ29lbiBvdmVyIHRoZSBmb3VyIHNhbXBsZXM/DQoNCiNWaWV3KGRhdC5kb2xfd2F0ZXIuZmlsLjRzYW1wLmNvcmUuQ29lblssMToxMF0pDQoNCkNvcmUuQ29lbiA9IENvcmUuek9UVS5Db2VuLjRzYW1wXzEuMCR2YXJpYWJsZQ0KDQpkYXQuZG9sX3dhdGVyLmZpbC40c2FtcC5jb3JlLkNvZW5fMiA9IGRhdC5kb2xfd2F0ZXIuZmlsLjRzYW1wLmNvcmUuQ29lbi9yb3dTdW1zKGRhdC5kb2xfd2F0ZXIuZmlsLjRzYW1wLmNvcmUuQ29lbikNCiNWaWV3KGRhdC5kb2xfd2F0ZXIuZmlsLjRzYW1wLmNvcmUuQ29lbl8yKQ0KDQpkYXQuZG9sX3dhdGVyLmZpbC40c2FtcC5jb3JlLkNvZW5fMyA9IGRhdC5kb2xfd2F0ZXIuZmlsLjRzYW1wLmNvcmUuQ29lbl8yWyxDb3JlLkNvZW5dDQojVmlldyhkYXQuZG9sX3dhdGVyLmZpbC40c2FtcC5jb3JlLkNvZW5fMykNCg0KI3Jvd25hbWVzKGRhdC5kb2xfd2F0ZXIuZmlsLjRzYW1wLmNvcmUuQ29lbl8zKQ0KI1sxXSAgIkNvZW5fMjgiICJDb2VuXzE5IiAiQ29lbl8xMSIgIkNvZW5fNiIgICANCg0KZGF0LmRvbF93YXRlci5maWwuNHNhbXAuY29yZS5Db2VuXzQgPSBkYXQuZG9sX3dhdGVyLmZpbC40c2FtcC5jb3JlLkNvZW5fMyAlPiUgbXV0YXRlKHN1bV9vZl9yb3dzID0gcm93U3VtcyhkYXQuZG9sX3dhdGVyLmZpbC40c2FtcC5jb3JlLkNvZW5fMykpDQojVmlldyhkYXQuZG9sX3dhdGVyLmZpbC40c2FtcC5jb3JlLkNvZW5fNCkNCmRhdC5kb2xfd2F0ZXIuZmlsLjRzYW1wLmNvcmUuQ29lbl80JHN1bV9vZl9yb3dzDQoNCiMiQ29lbl82IjogIDAuOTQ0MTA2NQ0KIyJDb2VuXzExIjogMC45NzExMzI1DQojIkNvZW5fMTkiOiAwLjkyOTg1NTcNCiMiQ29lbl8yOCI6IDAuNjcyMjI0MA0KDQptZWFuKGRhdC5kb2xfd2F0ZXIuZmlsLjRzYW1wLmNvcmUuQ29lbl80JHN1bV9vZl9yb3dzKQ0KIzAuODc5MzI5Nw0KYGBgDQoNCiMjI0V2aWUNCmBgYHtyfQ0KIyMjRXZpZQ0KI0NhbGN1bGF0ZSBjb3JlIHRheGEgdGhhdCAxMDAgJSBvZiBFdmllJ3Mgc2FtcGxlcyBoYXZlIGluIGNvbW1vbg0KI2RhdC5kb2xfd2F0ZXIuZmlsLjRzYW1wLmNvcmUNCg0KZGF0LmRvbF93YXRlci5maWwuNHNhbXAuY29yZS5FdmllIDwtICBkYXQuZG9sX3dhdGVyLmZpbC40c2FtcC5jb3JlW2MoNTo4KSxdDQoNCmRpbShkYXQuZG9sX3dhdGVyLmZpbC40c2FtcC5jb3JlLkV2aWUpDQojNCAxNTcxDQoNCmRhdC5kb2xfd2F0ZXIuZmlsLjRzYW1wLmNvcmUuYmluYXJ5LkV2aWUgPC0gZGF0LmRvbF93YXRlci5maWwuNHNhbXAuY29yZS5FdmllDQoNCiNDb252ZXJ0IGRhdGFmcmFtZSBpbnRvIGJpbmFyeQ0KZGF0LmRvbF93YXRlci5maWwuNHNhbXAuY29yZS5iaW5hcnkuRXZpZVtdIDwtICsoZGF0LmRvbF93YXRlci5maWwuNHNhbXAuY29yZS5iaW5hcnkuRXZpZSAgPiAwKQ0KI1ZpZXcoZGF0LmRvbF93YXRlci5maWwuNHNhbXAuY29yZS5iaW5hcnkuRXZpZSBbLDE6MTBdKQ0KDQojVHJhbnNwb3NlIGRhdC5kb2xfd2F0ZXIuZmlsLjRzYW1wLmNvcmUuYmluYXJ5LkV2aWUNCmRhdC5kb2xfd2F0ZXIuZmlsLjRzYW1wLmNvcmUuYmluYXJ5LkV2aWVfdCA8LSBhcy5kYXRhLmZyYW1lKHQoZGF0LmRvbF93YXRlci5maWwuNHNhbXAuY29yZS5iaW5hcnkuRXZpZSkpDQojVmlldyhkYXQuZG9sX3dhdGVyLmZpbC40c2FtcC5jb3JlLmJpbmFyeS5FdmllX3RbMToxMCxdKQ0KDQojQ3JlYXRlIGNvbCBUb3RhbC5yZWwuYWJ1bmQuDQpkYXQuZG9sX3dhdGVyLmZpbC40c2FtcC5jb3JlLmJpbmFyeS5FdmllX3QuMiA8LSBkYXQuZG9sX3dhdGVyLmZpbC40c2FtcC5jb3JlLmJpbmFyeS5FdmllX3QgJT4lIA0KICBtdXRhdGUoVG90YWwucmVsLmFidW5kLnpPVFUgPSByb3dTdW1zKGRhdC5kb2xfd2F0ZXIuZmlsLjRzYW1wLmNvcmUuYmluYXJ5LkV2aWVfdCkvNCkNCg0KI1ZpZXcoZGF0LmRvbF93YXRlci5maWwuNHNhbXAuY29yZS5iaW5hcnkuRXZpZV90LjJbMToxMCxdKQ0KDQojR2l2ZSBkYXQuZG9sX3dhdGVyLmZpbC40c2FtcC5jb3JlLmJpbmFyeS5FdmllX3QuMiByb3duYW1lcyBhcyBjb2wgdmFyaWFibGUNCmRhdC5kb2xfd2F0ZXIuZmlsLjRzYW1wLmNvcmUuYmluYXJ5LkV2aWVfdC4yJHZhcmlhYmxlIDwtIHJvd25hbWVzKGRhdC5kb2xfd2F0ZXIuZmlsLjRzYW1wLmNvcmUuYmluYXJ5LkV2aWVfdCkNCmRpbShkYXQuZG9sX3dhdGVyLmZpbC40c2FtcC5jb3JlLmJpbmFyeS5FdmllX3QuMikNCiMxNDcxIDYNCg0KI1Jlb3JkZXINCmRhdC5kb2xfd2F0ZXIuZmlsLjRzYW1wLmNvcmUuYmluYXJ5LkV2aWVfdC4zIDwtIGRhdC5kb2xfd2F0ZXIuZmlsLjRzYW1wLmNvcmUuYmluYXJ5LkV2aWVfdC4yWyxjKDUsNiwxOjQpXQ0KDQojSm9pbiBkYXQuZG9sX3dhdGVyLmZpbC40c2FtcC5jb3JlLmJpbmFyeS5FdmllX3QuMyB3aXRoIGF0dHJpYnV0ZXMubWlncmF0aW9uX3JkcA0KQ29yZS56T1RVLkV2aWUuNHNhbXAgPC0gZGF0LmRvbF93YXRlci5maWwuNHNhbXAuY29yZS5iaW5hcnkuRXZpZV90LjMgJT4lIGxlZnRfam9pbihhdHRyaWJ1dGVzLmRvbF9yZHBfd2F0ZXIuZmlsLjIpDQpkaW0oQ29yZS56T1RVLkV2aWUuNHNhbXApDQojIDE0NzEgICAxMg0KDQojVmlldyhDb3JlLnpPVFUuRXZpZS40c2FtcFsxOjEwLF0pDQoNCiNSZW9yZGVyDQpDb3JlLnpPVFUuRXZpZS40c2FtcF8yID0gQ29yZS56T1RVLkV2aWUuNHNhbXBbLGMoMSwyLDExLDM6NildDQpkaW0oQ29yZS56T1RVLkV2aWUuNHNhbXBfMikNCiMjMTQ3MSAgICA3DQoNCiNuYW1lcyhDb3JlLnpPVFUuRXZpZS40c2FtcF8yKQ0KDQojT3JkZXIgYWNjb3JkaW5nIHRvIENvcmUuek9UVS5FdmllLjRzYW1wXzIkVG90YWwucmVsLmFidW5kLnpPVFUNCkNvcmUuek9UVS5FdmllLjRzYW1wXzMgPSBDb3JlLnpPVFUuRXZpZS40c2FtcF8yW29yZGVyKENvcmUuek9UVS5FdmllLjRzYW1wXzIkVG90YWwucmVsLmFidW5kLnpPVFUpLF0NCiNWaWV3KENvcmUuek9UVS5FdmllLjRzYW1wXzNbLDE6Ml0pDQoNCiNBbnkgY29yZU9UVXMgYWJvdmUgMTAwJSAoPT0gMS4wKT8NCkNvcmUuek9UVS5FdmllLjRzYW1wXzEuMCA9IENvcmUuek9UVS5FdmllLjRzYW1wXzMgJT4lIGZpbHRlciAoVG90YWwucmVsLmFidW5kLnpPVFUgPT0gMS4wKQ0KZGltKENvcmUuek9UVS5FdmllLjRzYW1wXzEuMCkNCiMxNjcgNw0KDQojIyMjIyMjDQojIyNIb3cgbWFueSB6T1RVcyBhcmUgcHJlc2VudCBwZXIgRXZpZSdzIHNhbXBsZSBvbiBhdmVyYWdlPw0KZGF0LmRvbF93YXRlci5maWwuNHNhbXAuY29yZS5iaW5hcnkuRXZpZV8yID0gZGF0LmRvbF93YXRlci5maWwuNHNhbXAuY29yZS5iaW5hcnkuRXZpZSAlPiUgbXV0YXRlKHN1bV9vZl9jb3VudHMgPSByb3dTdW1zKGRhdC5kb2xfd2F0ZXIuZmlsLjRzYW1wLmNvcmUuYmluYXJ5LkV2aWUpKQ0KDQptZWFuKGRhdC5kb2xfd2F0ZXIuZmlsLjRzYW1wLmNvcmUuYmluYXJ5LkV2aWVfMiRzdW1fb2ZfY291bnRzKQ0KIzM5MA0KDQojIyMjIyMjDQojV2hhdCdzIHRoZSBwcmV2YWxlbmNlIG9mIHRoZSBjb3JlIHpPVFVzIGluIEV2aWUgb3ZlciB0aGUgZm91ciBzYW1wbGVzPw0KDQojVmlldyhkYXQuZG9sX3dhdGVyLmZpbC40c2FtcC5jb3JlLkV2aWVbLDE6MTBdKQ0KDQpDb3JlLkV2aWUgPSBDb3JlLnpPVFUuRXZpZS40c2FtcF8xLjAkdmFyaWFibGUNCg0KZGF0LmRvbF93YXRlci5maWwuNHNhbXAuY29yZS5FdmllXzIgPSBkYXQuZG9sX3dhdGVyLmZpbC40c2FtcC5jb3JlLkV2aWUvcm93U3VtcyhkYXQuZG9sX3dhdGVyLmZpbC40c2FtcC5jb3JlLkV2aWUpDQojVmlldyhkYXQuZG9sX3dhdGVyLmZpbC40c2FtcC5jb3JlLkV2aWVfMikNCg0KZGF0LmRvbF93YXRlci5maWwuNHNhbXAuY29yZS5FdmllXzMgPSBkYXQuZG9sX3dhdGVyLmZpbC40c2FtcC5jb3JlLkV2aWVfMlssQ29yZS5FdmllXQ0KI1ZpZXcoZGF0LmRvbF93YXRlci5maWwuNHNhbXAuY29yZS5FdmllXzMpDQoNCiNyb3duYW1lcyhkYXQuZG9sX3dhdGVyLmZpbC40c2FtcC5jb3JlLkV2aWVfMykNCiNbMV0gICJFdmllXzI4IiAiRXZpZV8xOSIgIkV2aWVfMTEiICJFdmllXzYiICAgDQoNCmRhdC5kb2xfd2F0ZXIuZmlsLjRzYW1wLmNvcmUuRXZpZV80ID0gZGF0LmRvbF93YXRlci5maWwuNHNhbXAuY29yZS5FdmllXzMgJT4lIG11dGF0ZShzdW1fb2Zfcm93cyA9IHJvd1N1bXMoZGF0LmRvbF93YXRlci5maWwuNHNhbXAuY29yZS5FdmllXzMpKQ0KI1ZpZXcoZGF0LmRvbF93YXRlci5maWwuNHNhbXAuY29yZS5FdmllXzQpDQpkYXQuZG9sX3dhdGVyLmZpbC40c2FtcC5jb3JlLkV2aWVfNCRzdW1fb2Zfcm93cw0KDQojIkV2aWVfNiI6ICAwLjY3Njk2MTUgDQojIkV2aWVfMTEiOiAwLjczMzcxODEgDQojIkV2aWVfMTkiOiAwLjczNDY0NjYgDQojIkV2aWVfMjgiOiAwLjgwNDUyMzIgDQoNCm1lYW4oZGF0LmRvbF93YXRlci5maWwuNHNhbXAuY29yZS5FdmllXzQkc3VtX29mX3Jvd3MpDQojMC43Mzc0NjI0DQpgYGANCg0KIyMjS2lhbWENCmBgYHtyfQ0KIyMjS2lhbWENCiNDYWxjdWxhdGUgY29yZSB0YXhhIHRoYXQgMTAwICUgb2YgS2lhbWEncyBzYW1wbGVzIGhhdmUgaW4gY29tbW9uDQojZGF0LmRvbF93YXRlci5maWwuNHNhbXAuY29yZQ0KDQpkYXQuZG9sX3dhdGVyLmZpbC40c2FtcC5jb3JlLktpYW1hIDwtICBkYXQuZG9sX3dhdGVyLmZpbC40c2FtcC5jb3JlW2MoMTM6MTYpLF0NCg0KZGltKGRhdC5kb2xfd2F0ZXIuZmlsLjRzYW1wLmNvcmUuS2lhbWEpDQojNCAxNTcxDQoNCmRhdC5kb2xfd2F0ZXIuZmlsLjRzYW1wLmNvcmUuYmluYXJ5LktpYW1hIDwtIGRhdC5kb2xfd2F0ZXIuZmlsLjRzYW1wLmNvcmUuS2lhbWENCg0KI0NvbnZlcnQgZGF0YWZyYW1lIGludG8gYmluYXJ5DQpkYXQuZG9sX3dhdGVyLmZpbC40c2FtcC5jb3JlLmJpbmFyeS5LaWFtYVtdIDwtICsoZGF0LmRvbF93YXRlci5maWwuNHNhbXAuY29yZS5iaW5hcnkuS2lhbWEgID4gMCkNCiNWaWV3KGRhdC5kb2xfd2F0ZXIuZmlsLjRzYW1wLmNvcmUuYmluYXJ5LktpYW1hIFssMToxMF0pDQoNCiNUcmFuc3Bvc2UgZGF0LmRvbF93YXRlci5maWwuNHNhbXAuY29yZS5iaW5hcnkuS2lhbWENCmRhdC5kb2xfd2F0ZXIuZmlsLjRzYW1wLmNvcmUuYmluYXJ5LktpYW1hX3QgPC0gYXMuZGF0YS5mcmFtZSh0KGRhdC5kb2xfd2F0ZXIuZmlsLjRzYW1wLmNvcmUuYmluYXJ5LktpYW1hKSkNCiNWaWV3KGRhdC5kb2xfd2F0ZXIuZmlsLjRzYW1wLmNvcmUuYmluYXJ5LktpYW1hX3RbMToxMCxdKQ0KDQojQ3JlYXRlIGNvbCBUb3RhbC5yZWwuYWJ1bmQuDQpkYXQuZG9sX3dhdGVyLmZpbC40c2FtcC5jb3JlLmJpbmFyeS5LaWFtYV90LjIgPC0gZGF0LmRvbF93YXRlci5maWwuNHNhbXAuY29yZS5iaW5hcnkuS2lhbWFfdCAlPiUgDQogIG11dGF0ZShUb3RhbC5yZWwuYWJ1bmQuek9UVSA9IHJvd1N1bXMoZGF0LmRvbF93YXRlci5maWwuNHNhbXAuY29yZS5iaW5hcnkuS2lhbWFfdCkvNCkNCg0KI1ZpZXcoZGF0LmRvbF93YXRlci5maWwuNHNhbXAuY29yZS5iaW5hcnkuS2lhbWFfdC4yWzE6MTAsXSkNCg0KI0dpdmUgZGF0LmRvbF93YXRlci5maWwuNHNhbXAuY29yZS5iaW5hcnkuS2lhbWFfdC4yIHJvd25hbWVzIGFzIGNvbCB2YXJpYWJsZQ0KZGF0LmRvbF93YXRlci5maWwuNHNhbXAuY29yZS5iaW5hcnkuS2lhbWFfdC4yJHZhcmlhYmxlIDwtIHJvd25hbWVzKGRhdC5kb2xfd2F0ZXIuZmlsLjRzYW1wLmNvcmUuYmluYXJ5LktpYW1hX3QpDQpkaW0oZGF0LmRvbF93YXRlci5maWwuNHNhbXAuY29yZS5iaW5hcnkuS2lhbWFfdC4yKQ0KIzE0NzEgNg0KDQojUmVvcmRlcg0KZGF0LmRvbF93YXRlci5maWwuNHNhbXAuY29yZS5iaW5hcnkuS2lhbWFfdC4zIDwtIGRhdC5kb2xfd2F0ZXIuZmlsLjRzYW1wLmNvcmUuYmluYXJ5LktpYW1hX3QuMlssYyg1LDYsMTo0KV0NCg0KI0pvaW4gZGF0LmRvbF93YXRlci5maWwuNHNhbXAuY29yZS5iaW5hcnkuS2lhbWFfdC4zIHdpdGggYXR0cmlidXRlcy5taWdyYXRpb25fcmRwDQpDb3JlLnpPVFUuS2lhbWEuNHNhbXAgPC0gZGF0LmRvbF93YXRlci5maWwuNHNhbXAuY29yZS5iaW5hcnkuS2lhbWFfdC4zICU+JSBsZWZ0X2pvaW4oYXR0cmlidXRlcy5kb2xfcmRwX3dhdGVyLmZpbC4yKQ0KZGltKENvcmUuek9UVS5LaWFtYS40c2FtcCkNCiMgMTQ3MSAgIDEyDQoNCiNWaWV3KENvcmUuek9UVS5LaWFtYS40c2FtcFsxOjEwLF0pDQoNCiNSZW9yZGVyDQpDb3JlLnpPVFUuS2lhbWEuNHNhbXBfMiA9IENvcmUuek9UVS5LaWFtYS40c2FtcFssYygxLDIsMTEsMzo2KV0NCmRpbShDb3JlLnpPVFUuS2lhbWEuNHNhbXBfMikNCiMjMTQ3MSAgICA3DQoNCiNuYW1lcyhDb3JlLnpPVFUuS2lhbWEuNHNhbXBfMikNCg0KI09yZGVyIGFjY29yZGluZyB0byBDb3JlLnpPVFUuS2lhbWEuNHNhbXBfMiRUb3RhbC5yZWwuYWJ1bmQuek9UVQ0KQ29yZS56T1RVLktpYW1hLjRzYW1wXzMgPSBDb3JlLnpPVFUuS2lhbWEuNHNhbXBfMltvcmRlcihDb3JlLnpPVFUuS2lhbWEuNHNhbXBfMiRUb3RhbC5yZWwuYWJ1bmQuek9UVSksXQ0KI1ZpZXcoQ29yZS56T1RVLktpYW1hLjRzYW1wXzNbLDE6Ml0pDQoNCiNBbnkgY29yZU9UVXMgYWJvdmUgMTAwJSAoPT0gMS4wKT8NCkNvcmUuek9UVS5LaWFtYS40c2FtcF8xLjAgPSBDb3JlLnpPVFUuS2lhbWEuNHNhbXBfMyAlPiUgZmlsdGVyIChUb3RhbC5yZWwuYWJ1bmQuek9UVSA9PSAxLjApDQpkaW0oQ29yZS56T1RVLktpYW1hLjRzYW1wXzEuMCkNCiMxOTEgNw0KDQojIyMjIyMjDQojIyNIb3cgbWFueSB6T1RVcyBhcmUgcHJlc2VudCBwZXIgS2lhbWEncyBzYW1wbGUgb24gYXZlcmFnZT8NCmRhdC5kb2xfd2F0ZXIuZmlsLjRzYW1wLmNvcmUuYmluYXJ5LktpYW1hXzIgPSBkYXQuZG9sX3dhdGVyLmZpbC40c2FtcC5jb3JlLmJpbmFyeS5LaWFtYSAlPiUgbXV0YXRlKHN1bV9vZl9jb3VudHMgPSByb3dTdW1zKGRhdC5kb2xfd2F0ZXIuZmlsLjRzYW1wLmNvcmUuYmluYXJ5LktpYW1hKSkNCg0KbWVhbihkYXQuZG9sX3dhdGVyLmZpbC40c2FtcC5jb3JlLmJpbmFyeS5LaWFtYV8yJHN1bV9vZl9jb3VudHMpDQojMzYzDQoNCiMjIyMjIyMNCiNXaGF0J3MgdGhlIHByZXZhbGVuY2Ugb2YgdGhlIGNvcmUgek9UVXMgaW4gS2lhbWEgb3ZlciB0aGUgZm91ciBzYW1wbGVzPw0KDQojVmlldyhkYXQuZG9sX3dhdGVyLmZpbC40c2FtcC5jb3JlLktpYW1hWywxOjEwXSkNCg0KQ29yZS5LaWFtYSA9IENvcmUuek9UVS5LaWFtYS40c2FtcF8xLjAkdmFyaWFibGUNCg0KZGF0LmRvbF93YXRlci5maWwuNHNhbXAuY29yZS5LaWFtYV8yID0gZGF0LmRvbF93YXRlci5maWwuNHNhbXAuY29yZS5LaWFtYS9yb3dTdW1zKGRhdC5kb2xfd2F0ZXIuZmlsLjRzYW1wLmNvcmUuS2lhbWEpDQojVmlldyhkYXQuZG9sX3dhdGVyLmZpbC40c2FtcC5jb3JlLktpYW1hXzIpDQoNCmRhdC5kb2xfd2F0ZXIuZmlsLjRzYW1wLmNvcmUuS2lhbWFfMyA9IGRhdC5kb2xfd2F0ZXIuZmlsLjRzYW1wLmNvcmUuS2lhbWFfMlssQ29yZS5LaWFtYV0NCiNWaWV3KGRhdC5kb2xfd2F0ZXIuZmlsLjRzYW1wLmNvcmUuS2lhbWFfMykNCg0KI3Jvd25hbWVzKGRhdC5kb2xfd2F0ZXIuZmlsLjRzYW1wLmNvcmUuS2lhbWFfMykNCiNbMV0gICJLaWFtYV8yOCIgIktpYW1hXzE5IiAiS2lhbWFfMTEiICJLaWFtYV82IiAgIA0KDQpkYXQuZG9sX3dhdGVyLmZpbC40c2FtcC5jb3JlLktpYW1hXzQgPSBkYXQuZG9sX3dhdGVyLmZpbC40c2FtcC5jb3JlLktpYW1hXzMgJT4lIG11dGF0ZShzdW1fb2Zfcm93cyA9IHJvd1N1bXMoZGF0LmRvbF93YXRlci5maWwuNHNhbXAuY29yZS5LaWFtYV8zKSkNCiNWaWV3KGRhdC5kb2xfd2F0ZXIuZmlsLjRzYW1wLmNvcmUuS2lhbWFfNCkNCmRhdC5kb2xfd2F0ZXIuZmlsLjRzYW1wLmNvcmUuS2lhbWFfNCRzdW1fb2Zfcm93cw0KDQojIktpYW1hXzYiOiAgMC45MDQ5MjE1IA0KIyJLaWFtYV8xMSI6IDAuODY1NjkyMiANCiMiS2lhbWFfMTkiOiAwLjg2ODk0NzIgDQojIktpYW1hXzI4IjogMC44MDc0NjYxDQoNCm1lYW4oZGF0LmRvbF93YXRlci5maWwuNHNhbXAuY29yZS5LaWFtYV80JHN1bV9vZl9yb3dzKQ0KIzAuODc5MzI5Nw0KYGBgDQoNCiMjI01va2kNCmBgYHtyfQ0KIyMjTW9raQ0KI0NhbGN1bGF0ZSBjb3JlIHRheGEgdGhhdCAxMDAgJSBvZiBNb2tpJ3Mgc2FtcGxlcyBoYXZlIGluIGNvbW1vbg0KI2RhdC5kb2xfd2F0ZXIuZmlsLjRzYW1wLmNvcmUNCg0KZGF0LmRvbF93YXRlci5maWwuNHNhbXAuY29yZS5Nb2tpIDwtICBkYXQuZG9sX3dhdGVyLmZpbC40c2FtcC5jb3JlW2MoMTc6MjApLF0NCg0KZGltKGRhdC5kb2xfd2F0ZXIuZmlsLjRzYW1wLmNvcmUuTW9raSkNCiM0IDE1NzENCg0KZGF0LmRvbF93YXRlci5maWwuNHNhbXAuY29yZS5iaW5hcnkuTW9raSA8LSBkYXQuZG9sX3dhdGVyLmZpbC40c2FtcC5jb3JlLk1va2kNCg0KI0NvbnZlcnQgZGF0YWZyYW1lIGludG8gYmluYXJ5DQpkYXQuZG9sX3dhdGVyLmZpbC40c2FtcC5jb3JlLmJpbmFyeS5Nb2tpW10gPC0gKyhkYXQuZG9sX3dhdGVyLmZpbC40c2FtcC5jb3JlLmJpbmFyeS5Nb2tpICA+IDApDQojVmlldyhkYXQuZG9sX3dhdGVyLmZpbC40c2FtcC5jb3JlLmJpbmFyeS5Nb2tpIFssMToxMF0pDQoNCiNUcmFuc3Bvc2UgZGF0LmRvbF93YXRlci5maWwuNHNhbXAuY29yZS5iaW5hcnkuTW9raQ0KZGF0LmRvbF93YXRlci5maWwuNHNhbXAuY29yZS5iaW5hcnkuTW9raV90IDwtIGFzLmRhdGEuZnJhbWUodChkYXQuZG9sX3dhdGVyLmZpbC40c2FtcC5jb3JlLmJpbmFyeS5Nb2tpKSkNCiNWaWV3KGRhdC5kb2xfd2F0ZXIuZmlsLjRzYW1wLmNvcmUuYmluYXJ5Lk1va2lfdFsxOjEwLF0pDQoNCiNDcmVhdGUgY29sIFRvdGFsLnJlbC5hYnVuZC4NCmRhdC5kb2xfd2F0ZXIuZmlsLjRzYW1wLmNvcmUuYmluYXJ5Lk1va2lfdC4yIDwtIGRhdC5kb2xfd2F0ZXIuZmlsLjRzYW1wLmNvcmUuYmluYXJ5Lk1va2lfdCAlPiUgDQogIG11dGF0ZShUb3RhbC5yZWwuYWJ1bmQuek9UVSA9IHJvd1N1bXMoZGF0LmRvbF93YXRlci5maWwuNHNhbXAuY29yZS5iaW5hcnkuTW9raV90KS80KQ0KDQojVmlldyhkYXQuZG9sX3dhdGVyLmZpbC40c2FtcC5jb3JlLmJpbmFyeS5Nb2tpX3QuMlsxOjEwLF0pDQoNCiNHaXZlIGRhdC5kb2xfd2F0ZXIuZmlsLjRzYW1wLmNvcmUuYmluYXJ5Lk1va2lfdC4yIHJvd25hbWVzIGFzIGNvbCB2YXJpYWJsZQ0KZGF0LmRvbF93YXRlci5maWwuNHNhbXAuY29yZS5iaW5hcnkuTW9raV90LjIkdmFyaWFibGUgPC0gcm93bmFtZXMoZGF0LmRvbF93YXRlci5maWwuNHNhbXAuY29yZS5iaW5hcnkuTW9raV90KQ0KZGltKGRhdC5kb2xfd2F0ZXIuZmlsLjRzYW1wLmNvcmUuYmluYXJ5Lk1va2lfdC4yKQ0KIzE0NzEgNg0KDQojUmVvcmRlcg0KZGF0LmRvbF93YXRlci5maWwuNHNhbXAuY29yZS5iaW5hcnkuTW9raV90LjMgPC0gZGF0LmRvbF93YXRlci5maWwuNHNhbXAuY29yZS5iaW5hcnkuTW9raV90LjJbLGMoNSw2LDE6NCldDQoNCiNKb2luIGRhdC5kb2xfd2F0ZXIuZmlsLjRzYW1wLmNvcmUuYmluYXJ5Lk1va2lfdC4zIHdpdGggYXR0cmlidXRlcy5taWdyYXRpb25fcmRwDQpDb3JlLnpPVFUuTW9raS40c2FtcCA8LSBkYXQuZG9sX3dhdGVyLmZpbC40c2FtcC5jb3JlLmJpbmFyeS5Nb2tpX3QuMyAlPiUgbGVmdF9qb2luKGF0dHJpYnV0ZXMuZG9sX3JkcF93YXRlci5maWwuMikNCmRpbShDb3JlLnpPVFUuTW9raS40c2FtcCkNCiMgMTQ3MSAgIDEyDQoNCiNWaWV3KENvcmUuek9UVS5Nb2tpLjRzYW1wWzE6MTAsXSkNCg0KI1Jlb3JkZXINCkNvcmUuek9UVS5Nb2tpLjRzYW1wXzIgPSBDb3JlLnpPVFUuTW9raS40c2FtcFssYygxLDIsMTEsMzo2KV0NCmRpbShDb3JlLnpPVFUuTW9raS40c2FtcF8yKQ0KIyMxNDcxICAgIDcNCg0KI25hbWVzKENvcmUuek9UVS5Nb2tpLjRzYW1wXzIpDQoNCiNPcmRlciBhY2NvcmRpbmcgdG8gQ29yZS56T1RVLk1va2kuNHNhbXBfMiRUb3RhbC5yZWwuYWJ1bmQuek9UVQ0KQ29yZS56T1RVLk1va2kuNHNhbXBfMyA9IENvcmUuek9UVS5Nb2tpLjRzYW1wXzJbb3JkZXIoQ29yZS56T1RVLk1va2kuNHNhbXBfMiRUb3RhbC5yZWwuYWJ1bmQuek9UVSksXQ0KI1ZpZXcoQ29yZS56T1RVLk1va2kuNHNhbXBfM1ssMToyXSkNCg0KI0FueSBjb3JlT1RVcyBhYm92ZSAxMDAlICg9PSAxLjApPw0KQ29yZS56T1RVLk1va2kuNHNhbXBfMS4wID0gQ29yZS56T1RVLk1va2kuNHNhbXBfMyAlPiUgZmlsdGVyIChUb3RhbC5yZWwuYWJ1bmQuek9UVSA9PSAxLjApDQpkaW0oQ29yZS56T1RVLk1va2kuNHNhbXBfMS4wKQ0KIzQyIDcNCg0KIyMjIyMjIw0KIyMjSG93IG1hbnkgek9UVXMgYXJlIHByZXNlbnQgcGVyIE1va2kncyBzYW1wbGUgb24gYXZlcmFnZT8NCmRhdC5kb2xfd2F0ZXIuZmlsLjRzYW1wLmNvcmUuYmluYXJ5Lk1va2lfMiA9IGRhdC5kb2xfd2F0ZXIuZmlsLjRzYW1wLmNvcmUuYmluYXJ5Lk1va2kgJT4lIG11dGF0ZShzdW1fb2ZfY291bnRzID0gcm93U3VtcyhkYXQuZG9sX3dhdGVyLmZpbC40c2FtcC5jb3JlLmJpbmFyeS5Nb2tpKSkNCg0KbWVhbihkYXQuZG9sX3dhdGVyLmZpbC40c2FtcC5jb3JlLmJpbmFyeS5Nb2tpXzIkc3VtX29mX2NvdW50cykNCiMyODANCg0KIyMjIyMjIw0KI1doYXQncyB0aGUgcHJldmFsZW5jZSBvZiB0aGUgY29yZSB6T1RVcyBpbiBNb2tpIG92ZXIgdGhlIGZvdXIgc2FtcGxlcz8NCg0KI1ZpZXcoZGF0LmRvbF93YXRlci5maWwuNHNhbXAuY29yZS5Nb2tpWywxOjEwXSkNCg0KQ29yZS5Nb2tpID0gQ29yZS56T1RVLk1va2kuNHNhbXBfMS4wJHZhcmlhYmxlDQoNCmRhdC5kb2xfd2F0ZXIuZmlsLjRzYW1wLmNvcmUuTW9raV8yID0gZGF0LmRvbF93YXRlci5maWwuNHNhbXAuY29yZS5Nb2tpL3Jvd1N1bXMoZGF0LmRvbF93YXRlci5maWwuNHNhbXAuY29yZS5Nb2tpKQ0KI1ZpZXcoZGF0LmRvbF93YXRlci5maWwuNHNhbXAuY29yZS5Nb2tpXzIpDQoNCmRhdC5kb2xfd2F0ZXIuZmlsLjRzYW1wLmNvcmUuTW9raV8zID0gZGF0LmRvbF93YXRlci5maWwuNHNhbXAuY29yZS5Nb2tpXzJbLENvcmUuTW9raV0NCiNWaWV3KGRhdC5kb2xfd2F0ZXIuZmlsLjRzYW1wLmNvcmUuTW9raV8zKQ0KDQojcm93bmFtZXMoZGF0LmRvbF93YXRlci5maWwuNHNhbXAuY29yZS5Nb2tpXzMpDQojWzFdICAiTW9raV8yOCIgIk1va2lfMTkiICJNb2tpXzExIiAiTW9raV82IiAgIA0KDQpkYXQuZG9sX3dhdGVyLmZpbC40c2FtcC5jb3JlLk1va2lfNCA9IGRhdC5kb2xfd2F0ZXIuZmlsLjRzYW1wLmNvcmUuTW9raV8zICU+JSBtdXRhdGUoc3VtX29mX3Jvd3MgPSByb3dTdW1zKGRhdC5kb2xfd2F0ZXIuZmlsLjRzYW1wLmNvcmUuTW9raV8zKSkNCiNWaWV3KGRhdC5kb2xfd2F0ZXIuZmlsLjRzYW1wLmNvcmUuTW9raV80KQ0KZGF0LmRvbF93YXRlci5maWwuNHNhbXAuY29yZS5Nb2tpXzQkc3VtX29mX3Jvd3MNCg0KIyJNb2tpXzYiOiAgMC40NDExMjg2IA0KIyJNb2tpXzExIjogMC40OTM1MDU4DQojIk1va2lfMTkiOiAwLjUyOTg1MzIgDQojIk1va2lfMjgiOiAwLjQyNTQ4OTUNCg0KbWVhbihkYXQuZG9sX3dhdGVyLmZpbC40c2FtcC5jb3JlLk1va2lfNCRzdW1fb2Zfcm93cykNCiMwLjQ3MjQ5NDINCmBgYA0KDQojIyNSQg0KYGBge3J9DQojIyNSQg0KI0NhbGN1bGF0ZSBjb3JlIHRheGEgdGhhdCAxMDAgJSBvZiBSQidzIHNhbXBsZXMgaGF2ZSBpbiBjb21tb24NCiNkYXQuZG9sX3dhdGVyLmZpbC40c2FtcC5jb3JlDQoNCmRhdC5kb2xfd2F0ZXIuZmlsLjRzYW1wLmNvcmUuUkIgPC0gIGRhdC5kb2xfd2F0ZXIuZmlsLjRzYW1wLmNvcmVbYygyNToyOCksXQ0KDQpkaW0oZGF0LmRvbF93YXRlci5maWwuNHNhbXAuY29yZS5SQikNCiM0IDE1NzENCg0KZGF0LmRvbF93YXRlci5maWwuNHNhbXAuY29yZS5iaW5hcnkuUkIgPC0gZGF0LmRvbF93YXRlci5maWwuNHNhbXAuY29yZS5SQg0KDQojQ29udmVydCBkYXRhZnJhbWUgaW50byBiaW5hcnkNCmRhdC5kb2xfd2F0ZXIuZmlsLjRzYW1wLmNvcmUuYmluYXJ5LlJCW10gPC0gKyhkYXQuZG9sX3dhdGVyLmZpbC40c2FtcC5jb3JlLmJpbmFyeS5SQiAgPiAwKQ0KI1ZpZXcoZGF0LmRvbF93YXRlci5maWwuNHNhbXAuY29yZS5iaW5hcnkuUkIgWywxOjEwXSkNCg0KI1RyYW5zcG9zZSBkYXQuZG9sX3dhdGVyLmZpbC40c2FtcC5jb3JlLmJpbmFyeS5SQg0KZGF0LmRvbF93YXRlci5maWwuNHNhbXAuY29yZS5iaW5hcnkuUkJfdCA8LSBhcy5kYXRhLmZyYW1lKHQoZGF0LmRvbF93YXRlci5maWwuNHNhbXAuY29yZS5iaW5hcnkuUkIpKQ0KI1ZpZXcoZGF0LmRvbF93YXRlci5maWwuNHNhbXAuY29yZS5iaW5hcnkuUkJfdFsxOjEwLF0pDQoNCiNDcmVhdGUgY29sIFRvdGFsLnJlbC5hYnVuZC4NCmRhdC5kb2xfd2F0ZXIuZmlsLjRzYW1wLmNvcmUuYmluYXJ5LlJCX3QuMiA8LSBkYXQuZG9sX3dhdGVyLmZpbC40c2FtcC5jb3JlLmJpbmFyeS5SQl90ICU+JSANCiAgbXV0YXRlKFRvdGFsLnJlbC5hYnVuZC56T1RVID0gcm93U3VtcyhkYXQuZG9sX3dhdGVyLmZpbC40c2FtcC5jb3JlLmJpbmFyeS5SQl90KS80KQ0KDQojVmlldyhkYXQuZG9sX3dhdGVyLmZpbC40c2FtcC5jb3JlLmJpbmFyeS5SQl90LjJbMToxMCxdKQ0KDQojR2l2ZSBkYXQuZG9sX3dhdGVyLmZpbC40c2FtcC5jb3JlLmJpbmFyeS5SQl90LjIgcm93bmFtZXMgYXMgY29sIHZhcmlhYmxlDQpkYXQuZG9sX3dhdGVyLmZpbC40c2FtcC5jb3JlLmJpbmFyeS5SQl90LjIkdmFyaWFibGUgPC0gcm93bmFtZXMoZGF0LmRvbF93YXRlci5maWwuNHNhbXAuY29yZS5iaW5hcnkuUkJfdCkNCmRpbShkYXQuZG9sX3dhdGVyLmZpbC40c2FtcC5jb3JlLmJpbmFyeS5SQl90LjIpDQojMTQ3MSA2DQoNCiNSZW9yZGVyDQpkYXQuZG9sX3dhdGVyLmZpbC40c2FtcC5jb3JlLmJpbmFyeS5SQl90LjMgPC0gZGF0LmRvbF93YXRlci5maWwuNHNhbXAuY29yZS5iaW5hcnkuUkJfdC4yWyxjKDUsNiwxOjQpXQ0KDQojSm9pbiBkYXQuZG9sX3dhdGVyLmZpbC40c2FtcC5jb3JlLmJpbmFyeS5SQl90LjMgd2l0aCBhdHRyaWJ1dGVzLm1pZ3JhdGlvbl9yZHANCkNvcmUuek9UVS5SQi40c2FtcCA8LSBkYXQuZG9sX3dhdGVyLmZpbC40c2FtcC5jb3JlLmJpbmFyeS5SQl90LjMgJT4lIGxlZnRfam9pbihhdHRyaWJ1dGVzLmRvbF9yZHBfd2F0ZXIuZmlsLjIpDQpkaW0oQ29yZS56T1RVLlJCLjRzYW1wKQ0KIyAxNDcxICAgMTINCg0KI1ZpZXcoQ29yZS56T1RVLlJCLjRzYW1wWzE6MTAsXSkNCg0KI1Jlb3JkZXINCkNvcmUuek9UVS5SQi40c2FtcF8yID0gQ29yZS56T1RVLlJCLjRzYW1wWyxjKDEsMiwxMSwzOjYpXQ0KZGltKENvcmUuek9UVS5SQi40c2FtcF8yKQ0KIyMxNDcxICAgIDcNCg0KI25hbWVzKENvcmUuek9UVS5SQi40c2FtcF8yKQ0KDQojT3JkZXIgYWNjb3JkaW5nIHRvIENvcmUuek9UVS5SQi40c2FtcF8yJFRvdGFsLnJlbC5hYnVuZC56T1RVDQpDb3JlLnpPVFUuUkIuNHNhbXBfMyA9IENvcmUuek9UVS5SQi40c2FtcF8yW29yZGVyKENvcmUuek9UVS5SQi40c2FtcF8yJFRvdGFsLnJlbC5hYnVuZC56T1RVKSxdDQojVmlldyhDb3JlLnpPVFUuUkIuNHNhbXBfM1ssMToyXSkNCg0KI0FueSBjb3JlT1RVcyBhYm92ZSAxMDAlICg9PSAxLjApPw0KQ29yZS56T1RVLlJCLjRzYW1wXzEuMCA9IENvcmUuek9UVS5SQi40c2FtcF8zICU+JSBmaWx0ZXIgKFRvdGFsLnJlbC5hYnVuZC56T1RVID09IDEuMCkNCmRpbShDb3JlLnpPVFUuUkIuNHNhbXBfMS4wKQ0KIzE4MCA3DQoNCiMjIyMjIyMNCiMjI0hvdyBtYW55IHpPVFVzIGFyZSBwcmVzZW50IHBlciBSQidzIHNhbXBsZSBvbiBhdmVyYWdlPw0KZGF0LmRvbF93YXRlci5maWwuNHNhbXAuY29yZS5iaW5hcnkuUkJfMiA9IGRhdC5kb2xfd2F0ZXIuZmlsLjRzYW1wLmNvcmUuYmluYXJ5LlJCICU+JSBtdXRhdGUoc3VtX29mX2NvdW50cyA9IHJvd1N1bXMoZGF0LmRvbF93YXRlci5maWwuNHNhbXAuY29yZS5iaW5hcnkuUkIpKQ0KDQptZWFuKGRhdC5kb2xfd2F0ZXIuZmlsLjRzYW1wLmNvcmUuYmluYXJ5LlJCXzIkc3VtX29mX2NvdW50cykNCiMzMzENCg0KIyMjIyMjIw0KI1doYXQncyB0aGUgcHJldmFsZW5jZSBvZiB0aGUgY29yZSB6T1RVcyBpbiBSQiBvdmVyIHRoZSBmb3VyIHNhbXBsZXM/DQoNCiNWaWV3KGRhdC5kb2xfd2F0ZXIuZmlsLjRzYW1wLmNvcmUuUkJbLDE6MTBdKQ0KDQpDb3JlLlJCID0gQ29yZS56T1RVLlJCLjRzYW1wXzEuMCR2YXJpYWJsZQ0KDQpkYXQuZG9sX3dhdGVyLmZpbC40c2FtcC5jb3JlLlJCXzIgPSBkYXQuZG9sX3dhdGVyLmZpbC40c2FtcC5jb3JlLlJCL3Jvd1N1bXMoZGF0LmRvbF93YXRlci5maWwuNHNhbXAuY29yZS5SQikNCiNWaWV3KGRhdC5kb2xfd2F0ZXIuZmlsLjRzYW1wLmNvcmUuUkJfMikNCg0KZGF0LmRvbF93YXRlci5maWwuNHNhbXAuY29yZS5SQl8zID0gZGF0LmRvbF93YXRlci5maWwuNHNhbXAuY29yZS5SQl8yWyxDb3JlLlJCXQ0KI1ZpZXcoZGF0LmRvbF93YXRlci5maWwuNHNhbXAuY29yZS5SQl8zKQ0KDQojcm93bmFtZXMoZGF0LmRvbF93YXRlci5maWwuNHNhbXAuY29yZS5SQl8zKQ0KI1sxXSAgIlJCXzI4IiAiUkJfMTkiICJSQl8xMSIgIlJCXzYiICAgDQoNCmRhdC5kb2xfd2F0ZXIuZmlsLjRzYW1wLmNvcmUuUkJfNCA9IGRhdC5kb2xfd2F0ZXIuZmlsLjRzYW1wLmNvcmUuUkJfMyAlPiUgbXV0YXRlKHN1bV9vZl9yb3dzID0gcm93U3VtcyhkYXQuZG9sX3dhdGVyLmZpbC40c2FtcC5jb3JlLlJCXzMpKQ0KI1ZpZXcoZGF0LmRvbF93YXRlci5maWwuNHNhbXAuY29yZS5SQl80KQ0KZGF0LmRvbF93YXRlci5maWwuNHNhbXAuY29yZS5SQl80JHN1bV9vZl9yb3dzDQoNCiMiUkJfNiI6ICAwLjk1NDE0MzQgDQojIlJCXzExIjogMC43NjgyNjc5DQojIlJCXzE5IjogMC42Mzc2MjkyIA0KIyJSQl8yOCI6IDAuODAwMzU5MSANCg0KbWVhbihkYXQuZG9sX3dhdGVyLmZpbC40c2FtcC5jb3JlLlJCXzQkc3VtX29mX3Jvd3MpDQojMC43OTAwOTk5DQpgYGANCg0KIyMjU2Nvb3Rlcg0KYGBge3J9DQojIyNTY29vdGVyDQojQ2FsY3VsYXRlIGNvcmUgdGF4YSB0aGF0IDEwMCAlIG9mIFNjb290ZXIncyBzYW1wbGVzIGhhdmUgaW4gY29tbW9uDQojZGF0LmRvbF93YXRlci5maWwuNHNhbXAuY29yZQ0KDQpkYXQuZG9sX3dhdGVyLmZpbC40c2FtcC5jb3JlLlNjb290ZXIgPC0gIGRhdC5kb2xfd2F0ZXIuZmlsLjRzYW1wLmNvcmVbYygyOTozMiksXQ0KDQpkaW0oZGF0LmRvbF93YXRlci5maWwuNHNhbXAuY29yZS5TY29vdGVyKQ0KIzQgMTU3MQ0KDQpkYXQuZG9sX3dhdGVyLmZpbC40c2FtcC5jb3JlLmJpbmFyeS5TY29vdGVyIDwtIGRhdC5kb2xfd2F0ZXIuZmlsLjRzYW1wLmNvcmUuU2Nvb3Rlcg0KDQojQ29udmVydCBkYXRhZnJhbWUgaW50byBiaW5hcnkNCmRhdC5kb2xfd2F0ZXIuZmlsLjRzYW1wLmNvcmUuYmluYXJ5LlNjb290ZXJbXSA8LSArKGRhdC5kb2xfd2F0ZXIuZmlsLjRzYW1wLmNvcmUuYmluYXJ5LlNjb290ZXIgID4gMCkNCiNWaWV3KGRhdC5kb2xfd2F0ZXIuZmlsLjRzYW1wLmNvcmUuYmluYXJ5LlNjb290ZXIgWywxOjEwXSkNCg0KI1RyYW5zcG9zZSBkYXQuZG9sX3dhdGVyLmZpbC40c2FtcC5jb3JlLmJpbmFyeS5TY29vdGVyDQpkYXQuZG9sX3dhdGVyLmZpbC40c2FtcC5jb3JlLmJpbmFyeS5TY29vdGVyX3QgPC0gYXMuZGF0YS5mcmFtZSh0KGRhdC5kb2xfd2F0ZXIuZmlsLjRzYW1wLmNvcmUuYmluYXJ5LlNjb290ZXIpKQ0KI1ZpZXcoZGF0LmRvbF93YXRlci5maWwuNHNhbXAuY29yZS5iaW5hcnkuU2Nvb3Rlcl90WzE6MTAsXSkNCg0KI0NyZWF0ZSBjb2wgVG90YWwucmVsLmFidW5kLg0KZGF0LmRvbF93YXRlci5maWwuNHNhbXAuY29yZS5iaW5hcnkuU2Nvb3Rlcl90LjIgPC0gZGF0LmRvbF93YXRlci5maWwuNHNhbXAuY29yZS5iaW5hcnkuU2Nvb3Rlcl90ICU+JSANCiAgbXV0YXRlKFRvdGFsLnJlbC5hYnVuZC56T1RVID0gcm93U3VtcyhkYXQuZG9sX3dhdGVyLmZpbC40c2FtcC5jb3JlLmJpbmFyeS5TY29vdGVyX3QpLzQpDQoNCiNWaWV3KGRhdC5kb2xfd2F0ZXIuZmlsLjRzYW1wLmNvcmUuYmluYXJ5LlNjb290ZXJfdC4yWzE6MTAsXSkNCg0KI0dpdmUgZGF0LmRvbF93YXRlci5maWwuNHNhbXAuY29yZS5iaW5hcnkuU2Nvb3Rlcl90LjIgcm93bmFtZXMgYXMgY29sIHZhcmlhYmxlDQpkYXQuZG9sX3dhdGVyLmZpbC40c2FtcC5jb3JlLmJpbmFyeS5TY29vdGVyX3QuMiR2YXJpYWJsZSA8LSByb3duYW1lcyhkYXQuZG9sX3dhdGVyLmZpbC40c2FtcC5jb3JlLmJpbmFyeS5TY29vdGVyX3QpDQpkaW0oZGF0LmRvbF93YXRlci5maWwuNHNhbXAuY29yZS5iaW5hcnkuU2Nvb3Rlcl90LjIpDQojMTQ3MSA2DQoNCiNSZW9yZGVyDQpkYXQuZG9sX3dhdGVyLmZpbC40c2FtcC5jb3JlLmJpbmFyeS5TY29vdGVyX3QuMyA8LSBkYXQuZG9sX3dhdGVyLmZpbC40c2FtcC5jb3JlLmJpbmFyeS5TY29vdGVyX3QuMlssYyg1LDYsMTo0KV0NCg0KI0pvaW4gZGF0LmRvbF93YXRlci5maWwuNHNhbXAuY29yZS5iaW5hcnkuU2Nvb3Rlcl90LjMgd2l0aCBhdHRyaWJ1dGVzLm1pZ3JhdGlvbl9yZHANCkNvcmUuek9UVS5TY29vdGVyLjRzYW1wIDwtIGRhdC5kb2xfd2F0ZXIuZmlsLjRzYW1wLmNvcmUuYmluYXJ5LlNjb290ZXJfdC4zICU+JSBsZWZ0X2pvaW4oYXR0cmlidXRlcy5kb2xfcmRwX3dhdGVyLmZpbC4yKQ0KZGltKENvcmUuek9UVS5TY29vdGVyLjRzYW1wKQ0KIyAxNDcxICAgMTINCg0KI1ZpZXcoQ29yZS56T1RVLlNjb290ZXIuNHNhbXBbMToxMCxdKQ0KDQojUmVvcmRlcg0KQ29yZS56T1RVLlNjb290ZXIuNHNhbXBfMiA9IENvcmUuek9UVS5TY29vdGVyLjRzYW1wWyxjKDEsMiwxMSwzOjYpXQ0KZGltKENvcmUuek9UVS5TY29vdGVyLjRzYW1wXzIpDQojIzE0NzEgICAgNw0KDQojbmFtZXMoQ29yZS56T1RVLlNjb290ZXIuNHNhbXBfMikNCg0KI09yZGVyIGFjY29yZGluZyB0byBDb3JlLnpPVFUuU2Nvb3Rlci40c2FtcF8yJFRvdGFsLnJlbC5hYnVuZC56T1RVDQpDb3JlLnpPVFUuU2Nvb3Rlci40c2FtcF8zID0gQ29yZS56T1RVLlNjb290ZXIuNHNhbXBfMltvcmRlcihDb3JlLnpPVFUuU2Nvb3Rlci40c2FtcF8yJFRvdGFsLnJlbC5hYnVuZC56T1RVKSxdDQojVmlldyhDb3JlLnpPVFUuU2Nvb3Rlci40c2FtcF8zWywxOjJdKQ0KDQojQW55IGNvcmVPVFVzIGFib3ZlIDEwMCUgKD09IDEuMCk/DQpDb3JlLnpPVFUuU2Nvb3Rlci40c2FtcF8xLjAgPSBDb3JlLnpPVFUuU2Nvb3Rlci40c2FtcF8zICU+JSBmaWx0ZXIgKFRvdGFsLnJlbC5hYnVuZC56T1RVID09IDEuMCkNCmRpbShDb3JlLnpPVFUuU2Nvb3Rlci40c2FtcF8xLjApDQojNTEgNw0KDQojIyMjIyMjDQojIyNIb3cgbWFueSB6T1RVcyBhcmUgcHJlc2VudCBwZXIgU2Nvb3RlcidzIHNhbXBsZSBvbiBhdmVyYWdlPw0KZGF0LmRvbF93YXRlci5maWwuNHNhbXAuY29yZS5iaW5hcnkuU2Nvb3Rlcl8yID0gZGF0LmRvbF93YXRlci5maWwuNHNhbXAuY29yZS5iaW5hcnkuU2Nvb3RlciAlPiUgbXV0YXRlKHN1bV9vZl9jb3VudHMgPSByb3dTdW1zKGRhdC5kb2xfd2F0ZXIuZmlsLjRzYW1wLmNvcmUuYmluYXJ5LlNjb290ZXIpKQ0KDQptZWFuKGRhdC5kb2xfd2F0ZXIuZmlsLjRzYW1wLmNvcmUuYmluYXJ5LlNjb290ZXJfMiRzdW1fb2ZfY291bnRzKQ0KIzI2MQ0KDQojIyMjIyMjDQojV2hhdCdzIHRoZSBwcmV2YWxlbmNlIG9mIHRoZSBjb3JlIHpPVFVzIGluIFNjb290ZXIgb3ZlciB0aGUgZm91ciBzYW1wbGVzPw0KDQojVmlldyhkYXQuZG9sX3dhdGVyLmZpbC40c2FtcC5jb3JlLlNjb290ZXJbLDE6MTBdKQ0KDQpDb3JlLlNjb290ZXIgPSBDb3JlLnpPVFUuU2Nvb3Rlci40c2FtcF8xLjAkdmFyaWFibGUNCg0KZGF0LmRvbF93YXRlci5maWwuNHNhbXAuY29yZS5TY29vdGVyXzIgPSBkYXQuZG9sX3dhdGVyLmZpbC40c2FtcC5jb3JlLlNjb290ZXIvcm93U3VtcyhkYXQuZG9sX3dhdGVyLmZpbC40c2FtcC5jb3JlLlNjb290ZXIpDQojVmlldyhkYXQuZG9sX3dhdGVyLmZpbC40c2FtcC5jb3JlLlNjb290ZXJfMikNCg0KZGF0LmRvbF93YXRlci5maWwuNHNhbXAuY29yZS5TY29vdGVyXzMgPSBkYXQuZG9sX3dhdGVyLmZpbC40c2FtcC5jb3JlLlNjb290ZXJfMlssQ29yZS5TY29vdGVyXQ0KI1ZpZXcoZGF0LmRvbF93YXRlci5maWwuNHNhbXAuY29yZS5TY29vdGVyXzMpDQoNCiNyb3duYW1lcyhkYXQuZG9sX3dhdGVyLmZpbC40c2FtcC5jb3JlLlNjb290ZXJfMykNCiNbMV0gICJTY29vdGVyXzI4IiAiU2Nvb3Rlcl8xOSIgIlNjb290ZXJfMTEiICJTY29vdGVyXzYiICAgDQoNCmRhdC5kb2xfd2F0ZXIuZmlsLjRzYW1wLmNvcmUuU2Nvb3Rlcl80ID0gZGF0LmRvbF93YXRlci5maWwuNHNhbXAuY29yZS5TY29vdGVyXzMgJT4lIG11dGF0ZShzdW1fb2Zfcm93cyA9IHJvd1N1bXMoZGF0LmRvbF93YXRlci5maWwuNHNhbXAuY29yZS5TY29vdGVyXzMpKQ0KI1ZpZXcoZGF0LmRvbF93YXRlci5maWwuNHNhbXAuY29yZS5TY29vdGVyXzQpDQpkYXQuZG9sX3dhdGVyLmZpbC40c2FtcC5jb3JlLlNjb290ZXJfNCRzdW1fb2Zfcm93cw0KDQojIlNjb290ZXJfNiI6ICAwLjQ5MzAzNDQgDQojIlNjb290ZXJfMTEiOiAwLjUxOTI0MDcgIA0KIyJTY29vdGVyXzE5IjogMC40MzE2OTI3ICANCiMiU2Nvb3Rlcl8yOCI6IDAuNjU0MDkzNQ0KDQptZWFuKGRhdC5kb2xfd2F0ZXIuZmlsLjRzYW1wLmNvcmUuU2Nvb3Rlcl80JHN1bV9vZl9yb3dzKQ0KIzAuNTI0NTE1Mw0KYGBgDQoNCiMjI1Npcml1cw0KYGBge3J9DQojIyNTaXJpdXMNCiNDYWxjdWxhdGUgY29yZSB0YXhhIHRoYXQgMTAwICUgb2YgU2lyaXVzJ3Mgc2FtcGxlcyBoYXZlIGluIGNvbW1vbg0KI2RhdC5kb2xfd2F0ZXIuZmlsLjRzYW1wLmNvcmUNCg0KZGF0LmRvbF93YXRlci5maWwuNHNhbXAuY29yZS5TaXJpdXMgPC0gIGRhdC5kb2xfd2F0ZXIuZmlsLjRzYW1wLmNvcmVbYygzMzozNiksXQ0KDQpkaW0oZGF0LmRvbF93YXRlci5maWwuNHNhbXAuY29yZS5TaXJpdXMpDQojNCAxNTcxDQoNCmRhdC5kb2xfd2F0ZXIuZmlsLjRzYW1wLmNvcmUuYmluYXJ5LlNpcml1cyA8LSBkYXQuZG9sX3dhdGVyLmZpbC40c2FtcC5jb3JlLlNpcml1cw0KDQojQ29udmVydCBkYXRhZnJhbWUgaW50byBiaW5hcnkNCmRhdC5kb2xfd2F0ZXIuZmlsLjRzYW1wLmNvcmUuYmluYXJ5LlNpcml1c1tdIDwtICsoZGF0LmRvbF93YXRlci5maWwuNHNhbXAuY29yZS5iaW5hcnkuU2lyaXVzICA+IDApDQojVmlldyhkYXQuZG9sX3dhdGVyLmZpbC40c2FtcC5jb3JlLmJpbmFyeS5TaXJpdXMgWywxOjEwXSkNCg0KI1RyYW5zcG9zZSBkYXQuZG9sX3dhdGVyLmZpbC40c2FtcC5jb3JlLmJpbmFyeS5TaXJpdXMNCmRhdC5kb2xfd2F0ZXIuZmlsLjRzYW1wLmNvcmUuYmluYXJ5LlNpcml1c190IDwtIGFzLmRhdGEuZnJhbWUodChkYXQuZG9sX3dhdGVyLmZpbC40c2FtcC5jb3JlLmJpbmFyeS5TaXJpdXMpKQ0KI1ZpZXcoZGF0LmRvbF93YXRlci5maWwuNHNhbXAuY29yZS5iaW5hcnkuU2lyaXVzX3RbMToxMCxdKQ0KDQojQ3JlYXRlIGNvbCBUb3RhbC5yZWwuYWJ1bmQuDQpkYXQuZG9sX3dhdGVyLmZpbC40c2FtcC5jb3JlLmJpbmFyeS5TaXJpdXNfdC4yIDwtIGRhdC5kb2xfd2F0ZXIuZmlsLjRzYW1wLmNvcmUuYmluYXJ5LlNpcml1c190ICU+JSANCiAgbXV0YXRlKFRvdGFsLnJlbC5hYnVuZC56T1RVID0gcm93U3VtcyhkYXQuZG9sX3dhdGVyLmZpbC40c2FtcC5jb3JlLmJpbmFyeS5TaXJpdXNfdCkvNCkNCg0KI1ZpZXcoZGF0LmRvbF93YXRlci5maWwuNHNhbXAuY29yZS5iaW5hcnkuU2lyaXVzX3QuMlsxOjEwLF0pDQoNCiNHaXZlIGRhdC5kb2xfd2F0ZXIuZmlsLjRzYW1wLmNvcmUuYmluYXJ5LlNpcml1c190LjIgcm93bmFtZXMgYXMgY29sIHZhcmlhYmxlDQpkYXQuZG9sX3dhdGVyLmZpbC40c2FtcC5jb3JlLmJpbmFyeS5TaXJpdXNfdC4yJHZhcmlhYmxlIDwtIHJvd25hbWVzKGRhdC5kb2xfd2F0ZXIuZmlsLjRzYW1wLmNvcmUuYmluYXJ5LlNpcml1c190KQ0KZGltKGRhdC5kb2xfd2F0ZXIuZmlsLjRzYW1wLmNvcmUuYmluYXJ5LlNpcml1c190LjIpDQojMTQ3MSA2DQoNCiNSZW9yZGVyDQpkYXQuZG9sX3dhdGVyLmZpbC40c2FtcC5jb3JlLmJpbmFyeS5TaXJpdXNfdC4zIDwtIGRhdC5kb2xfd2F0ZXIuZmlsLjRzYW1wLmNvcmUuYmluYXJ5LlNpcml1c190LjJbLGMoNSw2LDE6NCldDQoNCiNKb2luIGRhdC5kb2xfd2F0ZXIuZmlsLjRzYW1wLmNvcmUuYmluYXJ5LlNpcml1c190LjMgd2l0aCBhdHRyaWJ1dGVzLm1pZ3JhdGlvbl9yZHANCkNvcmUuek9UVS5TaXJpdXMuNHNhbXAgPC0gZGF0LmRvbF93YXRlci5maWwuNHNhbXAuY29yZS5iaW5hcnkuU2lyaXVzX3QuMyAlPiUgbGVmdF9qb2luKGF0dHJpYnV0ZXMuZG9sX3JkcF93YXRlci5maWwuMikNCmRpbShDb3JlLnpPVFUuU2lyaXVzLjRzYW1wKQ0KIyAxNDcxICAgMTINCg0KI1ZpZXcoQ29yZS56T1RVLlNpcml1cy40c2FtcFsxOjEwLF0pDQoNCiNSZW9yZGVyDQpDb3JlLnpPVFUuU2lyaXVzLjRzYW1wXzIgPSBDb3JlLnpPVFUuU2lyaXVzLjRzYW1wWyxjKDEsMiwxMSwzOjYpXQ0KZGltKENvcmUuek9UVS5TaXJpdXMuNHNhbXBfMikNCiMjMTQ3MSAgICA3DQoNCiNuYW1lcyhDb3JlLnpPVFUuU2lyaXVzLjRzYW1wXzIpDQoNCiNPcmRlciBhY2NvcmRpbmcgdG8gQ29yZS56T1RVLlNpcml1cy40c2FtcF8yJFRvdGFsLnJlbC5hYnVuZC56T1RVDQpDb3JlLnpPVFUuU2lyaXVzLjRzYW1wXzMgPSBDb3JlLnpPVFUuU2lyaXVzLjRzYW1wXzJbb3JkZXIoQ29yZS56T1RVLlNpcml1cy40c2FtcF8yJFRvdGFsLnJlbC5hYnVuZC56T1RVKSxdDQojVmlldyhDb3JlLnpPVFUuU2lyaXVzLjRzYW1wXzNbLDE6Ml0pDQoNCiNBbnkgY29yZU9UVXMgYWJvdmUgMTAwJSAoPT0gMS4wKT8NCkNvcmUuek9UVS5TaXJpdXMuNHNhbXBfMS4wID0gQ29yZS56T1RVLlNpcml1cy40c2FtcF8zICU+JSBmaWx0ZXIgKFRvdGFsLnJlbC5hYnVuZC56T1RVID09IDEuMCkNCmRpbShDb3JlLnpPVFUuU2lyaXVzLjRzYW1wXzEuMCkNCiMzOCA3DQoNCiMjIyMjIyMNCiMjI0hvdyBtYW55IHpPVFVzIGFyZSBwcmVzZW50IHBlciBTaXJpdXMncyBzYW1wbGUgb24gYXZlcmFnZT8NCmRhdC5kb2xfd2F0ZXIuZmlsLjRzYW1wLmNvcmUuYmluYXJ5LlNpcml1c18yID0gZGF0LmRvbF93YXRlci5maWwuNHNhbXAuY29yZS5iaW5hcnkuU2lyaXVzICU+JSBtdXRhdGUoc3VtX29mX2NvdW50cyA9IHJvd1N1bXMoZGF0LmRvbF93YXRlci5maWwuNHNhbXAuY29yZS5iaW5hcnkuU2lyaXVzKSkNCg0KbWVhbihkYXQuZG9sX3dhdGVyLmZpbC40c2FtcC5jb3JlLmJpbmFyeS5TaXJpdXNfMiRzdW1fb2ZfY291bnRzKQ0KIzI0Ng0KDQojIyMjIyMjDQojV2hhdCdzIHRoZSBwcmV2YWxlbmNlIG9mIHRoZSBjb3JlIHpPVFVzIGluIFNpcml1cyBvdmVyIHRoZSBmb3VyIHNhbXBsZXM/DQoNCiNWaWV3KGRhdC5kb2xfd2F0ZXIuZmlsLjRzYW1wLmNvcmUuU2lyaXVzWywxOjEwXSkNCg0KQ29yZS5TaXJpdXMgPSBDb3JlLnpPVFUuU2lyaXVzLjRzYW1wXzEuMCR2YXJpYWJsZQ0KDQpkYXQuZG9sX3dhdGVyLmZpbC40c2FtcC5jb3JlLlNpcml1c18yID0gZGF0LmRvbF93YXRlci5maWwuNHNhbXAuY29yZS5TaXJpdXMvcm93U3VtcyhkYXQuZG9sX3dhdGVyLmZpbC40c2FtcC5jb3JlLlNpcml1cykNCiNWaWV3KGRhdC5kb2xfd2F0ZXIuZmlsLjRzYW1wLmNvcmUuU2lyaXVzXzIpDQoNCmRhdC5kb2xfd2F0ZXIuZmlsLjRzYW1wLmNvcmUuU2lyaXVzXzMgPSBkYXQuZG9sX3dhdGVyLmZpbC40c2FtcC5jb3JlLlNpcml1c18yWyxDb3JlLlNpcml1c10NCiNWaWV3KGRhdC5kb2xfd2F0ZXIuZmlsLjRzYW1wLmNvcmUuU2lyaXVzXzMpDQoNCiNyb3duYW1lcyhkYXQuZG9sX3dhdGVyLmZpbC40c2FtcC5jb3JlLlNpcml1c18zKQ0KI1sxXSAgIlNpcml1c18yOCIgIlNpcml1c18xOSIgIlNpcml1c18xMSIgIlNpcml1c182IiAgIA0KDQpkYXQuZG9sX3dhdGVyLmZpbC40c2FtcC5jb3JlLlNpcml1c180ID0gZGF0LmRvbF93YXRlci5maWwuNHNhbXAuY29yZS5TaXJpdXNfMyAlPiUgbXV0YXRlKHN1bV9vZl9yb3dzID0gcm93U3VtcyhkYXQuZG9sX3dhdGVyLmZpbC40c2FtcC5jb3JlLlNpcml1c18zKSkNCiNWaWV3KGRhdC5kb2xfd2F0ZXIuZmlsLjRzYW1wLmNvcmUuU2lyaXVzXzQpDQpkYXQuZG9sX3dhdGVyLmZpbC40c2FtcC5jb3JlLlNpcml1c180JHN1bV9vZl9yb3dzDQoNCiMiU2lyaXVzXzYiOiAgMC40NjkxOTcxIA0KIyJTaXJpdXNfMTEiOiAwLjM4NjgzMDggDQojIlNpcml1c18xOSI6IDAuNDMzMzAzOCANCiMiU2lyaXVzXzI4IjogMC44MDMyMjM4IA0KDQptZWFuKGRhdC5kb2xfd2F0ZXIuZmlsLjRzYW1wLmNvcmUuU2lyaXVzXzQkc3VtX29mX3Jvd3MpDQojMC41MjMxMzg5DQpgYGANCg0KIyMjU3F1ZWFrDQpgYGB7cn0NCiMjI1NxdWVhaw0KI0NhbGN1bGF0ZSBjb3JlIHRheGEgdGhhdCAxMDAgJSBvZiBTcXVlYWsncyBzYW1wbGVzIGhhdmUgaW4gY29tbW9uDQojZGF0LmRvbF93YXRlci5maWwuNHNhbXAuY29yZQ0KDQpkYXQuZG9sX3dhdGVyLmZpbC40c2FtcC5jb3JlLlNxdWVhayA8LSAgZGF0LmRvbF93YXRlci5maWwuNHNhbXAuY29yZVtjKDM3OjQwKSxdDQoNCmRpbShkYXQuZG9sX3dhdGVyLmZpbC40c2FtcC5jb3JlLlNxdWVhaykNCiM0IDE1NzENCg0KZGF0LmRvbF93YXRlci5maWwuNHNhbXAuY29yZS5iaW5hcnkuU3F1ZWFrIDwtIGRhdC5kb2xfd2F0ZXIuZmlsLjRzYW1wLmNvcmUuU3F1ZWFrDQoNCiNDb252ZXJ0IGRhdGFmcmFtZSBpbnRvIGJpbmFyeQ0KZGF0LmRvbF93YXRlci5maWwuNHNhbXAuY29yZS5iaW5hcnkuU3F1ZWFrW10gPC0gKyhkYXQuZG9sX3dhdGVyLmZpbC40c2FtcC5jb3JlLmJpbmFyeS5TcXVlYWsgID4gMCkNCiNWaWV3KGRhdC5kb2xfd2F0ZXIuZmlsLjRzYW1wLmNvcmUuYmluYXJ5LlNxdWVhayBbLDE6MTBdKQ0KDQojVHJhbnNwb3NlIGRhdC5kb2xfd2F0ZXIuZmlsLjRzYW1wLmNvcmUuYmluYXJ5LlNxdWVhaw0KZGF0LmRvbF93YXRlci5maWwuNHNhbXAuY29yZS5iaW5hcnkuU3F1ZWFrX3QgPC0gYXMuZGF0YS5mcmFtZSh0KGRhdC5kb2xfd2F0ZXIuZmlsLjRzYW1wLmNvcmUuYmluYXJ5LlNxdWVhaykpDQojVmlldyhkYXQuZG9sX3dhdGVyLmZpbC40c2FtcC5jb3JlLmJpbmFyeS5TcXVlYWtfdFsxOjEwLF0pDQoNCiNDcmVhdGUgY29sIFRvdGFsLnJlbC5hYnVuZC4NCmRhdC5kb2xfd2F0ZXIuZmlsLjRzYW1wLmNvcmUuYmluYXJ5LlNxdWVha190LjIgPC0gZGF0LmRvbF93YXRlci5maWwuNHNhbXAuY29yZS5iaW5hcnkuU3F1ZWFrX3QgJT4lIA0KICBtdXRhdGUoVG90YWwucmVsLmFidW5kLnpPVFUgPSByb3dTdW1zKGRhdC5kb2xfd2F0ZXIuZmlsLjRzYW1wLmNvcmUuYmluYXJ5LlNxdWVha190KS80KQ0KDQojVmlldyhkYXQuZG9sX3dhdGVyLmZpbC40c2FtcC5jb3JlLmJpbmFyeS5TcXVlYWtfdC4yWzE6MTAsXSkNCg0KI0dpdmUgZGF0LmRvbF93YXRlci5maWwuNHNhbXAuY29yZS5iaW5hcnkuU3F1ZWFrX3QuMiByb3duYW1lcyBhcyBjb2wgdmFyaWFibGUNCmRhdC5kb2xfd2F0ZXIuZmlsLjRzYW1wLmNvcmUuYmluYXJ5LlNxdWVha190LjIkdmFyaWFibGUgPC0gcm93bmFtZXMoZGF0LmRvbF93YXRlci5maWwuNHNhbXAuY29yZS5iaW5hcnkuU3F1ZWFrX3QpDQpkaW0oZGF0LmRvbF93YXRlci5maWwuNHNhbXAuY29yZS5iaW5hcnkuU3F1ZWFrX3QuMikNCiMxNDcxIDYNCg0KI1Jlb3JkZXINCmRhdC5kb2xfd2F0ZXIuZmlsLjRzYW1wLmNvcmUuYmluYXJ5LlNxdWVha190LjMgPC0gZGF0LmRvbF93YXRlci5maWwuNHNhbXAuY29yZS5iaW5hcnkuU3F1ZWFrX3QuMlssYyg1LDYsMTo0KV0NCg0KI0pvaW4gZGF0LmRvbF93YXRlci5maWwuNHNhbXAuY29yZS5iaW5hcnkuU3F1ZWFrX3QuMyB3aXRoIGF0dHJpYnV0ZXMubWlncmF0aW9uX3JkcA0KQ29yZS56T1RVLlNxdWVhay40c2FtcCA8LSBkYXQuZG9sX3dhdGVyLmZpbC40c2FtcC5jb3JlLmJpbmFyeS5TcXVlYWtfdC4zICU+JSBsZWZ0X2pvaW4oYXR0cmlidXRlcy5kb2xfcmRwX3dhdGVyLmZpbC4yKQ0KZGltKENvcmUuek9UVS5TcXVlYWsuNHNhbXApDQojIDE0NzEgICAxMg0KDQojVmlldyhDb3JlLnpPVFUuU3F1ZWFrLjRzYW1wWzE6MTAsXSkNCg0KI1Jlb3JkZXINCkNvcmUuek9UVS5TcXVlYWsuNHNhbXBfMiA9IENvcmUuek9UVS5TcXVlYWsuNHNhbXBbLGMoMSwyLDExLDM6NildDQpkaW0oQ29yZS56T1RVLlNxdWVhay40c2FtcF8yKQ0KIyMxNDcxICAgIDcNCg0KI25hbWVzKENvcmUuek9UVS5TcXVlYWsuNHNhbXBfMikNCg0KI09yZGVyIGFjY29yZGluZyB0byBDb3JlLnpPVFUuU3F1ZWFrLjRzYW1wXzIkVG90YWwucmVsLmFidW5kLnpPVFUNCkNvcmUuek9UVS5TcXVlYWsuNHNhbXBfMyA9IENvcmUuek9UVS5TcXVlYWsuNHNhbXBfMltvcmRlcihDb3JlLnpPVFUuU3F1ZWFrLjRzYW1wXzIkVG90YWwucmVsLmFidW5kLnpPVFUpLF0NCiNWaWV3KENvcmUuek9UVS5TcXVlYWsuNHNhbXBfM1ssMToyXSkNCg0KI0FueSBjb3JlT1RVcyBhYm92ZSAxMDAlICg9PSAxLjApPw0KQ29yZS56T1RVLlNxdWVhay40c2FtcF8xLjAgPSBDb3JlLnpPVFUuU3F1ZWFrLjRzYW1wXzMgJT4lIGZpbHRlciAoVG90YWwucmVsLmFidW5kLnpPVFUgPT0gMS4wKQ0KZGltKENvcmUuek9UVS5TcXVlYWsuNHNhbXBfMS4wKQ0KIzg3IDcNCg0KIyMjIyMjIw0KIyMjSG93IG1hbnkgek9UVXMgYXJlIHByZXNlbnQgcGVyIFNxdWVhaydzIHNhbXBsZSBvbiBhdmVyYWdlPw0KZGF0LmRvbF93YXRlci5maWwuNHNhbXAuY29yZS5iaW5hcnkuU3F1ZWFrXzIgPSBkYXQuZG9sX3dhdGVyLmZpbC40c2FtcC5jb3JlLmJpbmFyeS5TcXVlYWsgJT4lIG11dGF0ZShzdW1fb2ZfY291bnRzID0gcm93U3VtcyhkYXQuZG9sX3dhdGVyLmZpbC40c2FtcC5jb3JlLmJpbmFyeS5TcXVlYWspKQ0KDQptZWFuKGRhdC5kb2xfd2F0ZXIuZmlsLjRzYW1wLmNvcmUuYmluYXJ5LlNxdWVha18yJHN1bV9vZl9jb3VudHMpDQojMzM0DQoNCiMjIyMjIyMNCiNXaGF0J3MgdGhlIHByZXZhbGVuY2Ugb2YgdGhlIGNvcmUgek9UVXMgaW4gU3F1ZWFrIG92ZXIgdGhlIGZvdXIgc2FtcGxlcz8NCg0KI1ZpZXcoZGF0LmRvbF93YXRlci5maWwuNHNhbXAuY29yZS5TcXVlYWtbLDE6MTBdKQ0KDQpDb3JlLlNxdWVhayA9IENvcmUuek9UVS5TcXVlYWsuNHNhbXBfMS4wJHZhcmlhYmxlDQoNCmRhdC5kb2xfd2F0ZXIuZmlsLjRzYW1wLmNvcmUuU3F1ZWFrXzIgPSBkYXQuZG9sX3dhdGVyLmZpbC40c2FtcC5jb3JlLlNxdWVhay9yb3dTdW1zKGRhdC5kb2xfd2F0ZXIuZmlsLjRzYW1wLmNvcmUuU3F1ZWFrKQ0KI1ZpZXcoZGF0LmRvbF93YXRlci5maWwuNHNhbXAuY29yZS5TcXVlYWtfMikNCg0KZGF0LmRvbF93YXRlci5maWwuNHNhbXAuY29yZS5TcXVlYWtfMyA9IGRhdC5kb2xfd2F0ZXIuZmlsLjRzYW1wLmNvcmUuU3F1ZWFrXzJbLENvcmUuU3F1ZWFrXQ0KI1ZpZXcoZGF0LmRvbF93YXRlci5maWwuNHNhbXAuY29yZS5TcXVlYWtfMykNCg0KI3Jvd25hbWVzKGRhdC5kb2xfd2F0ZXIuZmlsLjRzYW1wLmNvcmUuU3F1ZWFrXzMpDQojWzFdICAiU3F1ZWFrXzI4IiAiU3F1ZWFrXzE5IiAiU3F1ZWFrXzExIiAiU3F1ZWFrXzYiICAgDQoNCmRhdC5kb2xfd2F0ZXIuZmlsLjRzYW1wLmNvcmUuU3F1ZWFrXzQgPSBkYXQuZG9sX3dhdGVyLmZpbC40c2FtcC5jb3JlLlNxdWVha18zICU+JSBtdXRhdGUoc3VtX29mX3Jvd3MgPSByb3dTdW1zKGRhdC5kb2xfd2F0ZXIuZmlsLjRzYW1wLmNvcmUuU3F1ZWFrXzMpKQ0KI1ZpZXcoZGF0LmRvbF93YXRlci5maWwuNHNhbXAuY29yZS5TcXVlYWtfNCkNCmRhdC5kb2xfd2F0ZXIuZmlsLjRzYW1wLmNvcmUuU3F1ZWFrXzQkc3VtX29mX3Jvd3MNCg0KIyJTcXVlYWtfNiI6ICAwLjY2NTI5OTEgDQojIlNxdWVha18xMSI6IDAuOTMzMDIzMSANCiMiU3F1ZWFrXzE5IjogMC40NTk1NzM0IA0KIyJTcXVlYWtfMjgiOiAwLjQ5Njk0MjcgDQoNCm1lYW4oZGF0LmRvbF93YXRlci5maWwuNHNhbXAuY29yZS5TcXVlYWtfNCRzdW1fb2Zfcm93cykNCiMwLjYzODcwOTYNCmBgYA0KDQojIyNTdGFyYnVjaw0KYGBge3J9DQojIyNTdGFyYnVjaw0KI0NhbGN1bGF0ZSBjb3JlIHRheGEgdGhhdCAxMDAgJSBvZiBTdGFyYnVjaydzIHNhbXBsZXMgaGF2ZSBpbiBjb21tb24NCiNkYXQuZG9sX3dhdGVyLmZpbC40c2FtcC5jb3JlDQoNCmRhdC5kb2xfd2F0ZXIuZmlsLjRzYW1wLmNvcmUuU3RhcmJ1Y2sgPC0gIGRhdC5kb2xfd2F0ZXIuZmlsLjRzYW1wLmNvcmVbYyg0MTo0NCksXQ0KDQpkaW0oZGF0LmRvbF93YXRlci5maWwuNHNhbXAuY29yZS5TdGFyYnVjaykNCiM0IDE1NzENCg0KZGF0LmRvbF93YXRlci5maWwuNHNhbXAuY29yZS5iaW5hcnkuU3RhcmJ1Y2sgPC0gZGF0LmRvbF93YXRlci5maWwuNHNhbXAuY29yZS5TdGFyYnVjaw0KDQojQ29udmVydCBkYXRhZnJhbWUgaW50byBiaW5hcnkNCmRhdC5kb2xfd2F0ZXIuZmlsLjRzYW1wLmNvcmUuYmluYXJ5LlN0YXJidWNrW10gPC0gKyhkYXQuZG9sX3dhdGVyLmZpbC40c2FtcC5jb3JlLmJpbmFyeS5TdGFyYnVjayAgPiAwKQ0KI1ZpZXcoZGF0LmRvbF93YXRlci5maWwuNHNhbXAuY29yZS5iaW5hcnkuU3RhcmJ1Y2sgWywxOjEwXSkNCg0KI1RyYW5zcG9zZSBkYXQuZG9sX3dhdGVyLmZpbC40c2FtcC5jb3JlLmJpbmFyeS5TdGFyYnVjaw0KZGF0LmRvbF93YXRlci5maWwuNHNhbXAuY29yZS5iaW5hcnkuU3RhcmJ1Y2tfdCA8LSBhcy5kYXRhLmZyYW1lKHQoZGF0LmRvbF93YXRlci5maWwuNHNhbXAuY29yZS5iaW5hcnkuU3RhcmJ1Y2spKQ0KI1ZpZXcoZGF0LmRvbF93YXRlci5maWwuNHNhbXAuY29yZS5iaW5hcnkuU3RhcmJ1Y2tfdFsxOjEwLF0pDQoNCiNDcmVhdGUgY29sIFRvdGFsLnJlbC5hYnVuZC4NCmRhdC5kb2xfd2F0ZXIuZmlsLjRzYW1wLmNvcmUuYmluYXJ5LlN0YXJidWNrX3QuMiA8LSBkYXQuZG9sX3dhdGVyLmZpbC40c2FtcC5jb3JlLmJpbmFyeS5TdGFyYnVja190ICU+JSANCiAgbXV0YXRlKFRvdGFsLnJlbC5hYnVuZC56T1RVID0gcm93U3VtcyhkYXQuZG9sX3dhdGVyLmZpbC40c2FtcC5jb3JlLmJpbmFyeS5TdGFyYnVja190KS80KQ0KDQojVmlldyhkYXQuZG9sX3dhdGVyLmZpbC40c2FtcC5jb3JlLmJpbmFyeS5TdGFyYnVja190LjJbMToxMCxdKQ0KDQojR2l2ZSBkYXQuZG9sX3dhdGVyLmZpbC40c2FtcC5jb3JlLmJpbmFyeS5TdGFyYnVja190LjIgcm93bmFtZXMgYXMgY29sIHZhcmlhYmxlDQpkYXQuZG9sX3dhdGVyLmZpbC40c2FtcC5jb3JlLmJpbmFyeS5TdGFyYnVja190LjIkdmFyaWFibGUgPC0gcm93bmFtZXMoZGF0LmRvbF93YXRlci5maWwuNHNhbXAuY29yZS5iaW5hcnkuU3RhcmJ1Y2tfdCkNCmRpbShkYXQuZG9sX3dhdGVyLmZpbC40c2FtcC5jb3JlLmJpbmFyeS5TdGFyYnVja190LjIpDQojMTQ3MSA2DQoNCiNSZW9yZGVyDQpkYXQuZG9sX3dhdGVyLmZpbC40c2FtcC5jb3JlLmJpbmFyeS5TdGFyYnVja190LjMgPC0gZGF0LmRvbF93YXRlci5maWwuNHNhbXAuY29yZS5iaW5hcnkuU3RhcmJ1Y2tfdC4yWyxjKDUsNiwxOjQpXQ0KDQojSm9pbiBkYXQuZG9sX3dhdGVyLmZpbC40c2FtcC5jb3JlLmJpbmFyeS5TdGFyYnVja190LjMgd2l0aCBhdHRyaWJ1dGVzLm1pZ3JhdGlvbl9yZHANCkNvcmUuek9UVS5TdGFyYnVjay40c2FtcCA8LSBkYXQuZG9sX3dhdGVyLmZpbC40c2FtcC5jb3JlLmJpbmFyeS5TdGFyYnVja190LjMgJT4lIGxlZnRfam9pbihhdHRyaWJ1dGVzLmRvbF9yZHBfd2F0ZXIuZmlsLjIpDQpkaW0oQ29yZS56T1RVLlN0YXJidWNrLjRzYW1wKQ0KIyAxNDcxICAgMTINCg0KI1ZpZXcoQ29yZS56T1RVLlN0YXJidWNrLjRzYW1wWzE6MTAsXSkNCg0KI1Jlb3JkZXINCkNvcmUuek9UVS5TdGFyYnVjay40c2FtcF8yID0gQ29yZS56T1RVLlN0YXJidWNrLjRzYW1wWyxjKDEsMiwxMSwzOjYpXQ0KZGltKENvcmUuek9UVS5TdGFyYnVjay40c2FtcF8yKQ0KIyMxNDcxICAgIDcNCg0KI25hbWVzKENvcmUuek9UVS5TdGFyYnVjay40c2FtcF8yKQ0KDQojT3JkZXIgYWNjb3JkaW5nIHRvIENvcmUuek9UVS5TdGFyYnVjay40c2FtcF8yJFRvdGFsLnJlbC5hYnVuZC56T1RVDQpDb3JlLnpPVFUuU3RhcmJ1Y2suNHNhbXBfMyA9IENvcmUuek9UVS5TdGFyYnVjay40c2FtcF8yW29yZGVyKENvcmUuek9UVS5TdGFyYnVjay40c2FtcF8yJFRvdGFsLnJlbC5hYnVuZC56T1RVKSxdDQojVmlldyhDb3JlLnpPVFUuU3RhcmJ1Y2suNHNhbXBfM1ssMToyXSkNCg0KI0FueSBjb3JlT1RVcyBhYm92ZSAxMDAlICg9PSAxLjApPw0KQ29yZS56T1RVLlN0YXJidWNrLjRzYW1wXzEuMCA9IENvcmUuek9UVS5TdGFyYnVjay40c2FtcF8zICU+JSBmaWx0ZXIgKFRvdGFsLnJlbC5hYnVuZC56T1RVID09IDEuMCkNCmRpbShDb3JlLnpPVFUuU3RhcmJ1Y2suNHNhbXBfMS4wKQ0KIzMwNiA3DQoNCiMjIyMjIyMNCiMjI0hvdyBtYW55IHpPVFVzIGFyZSBwcmVzZW50IHBlciBTdGFyYnVjaydzIHNhbXBsZSBvbiBhdmVyYWdlPw0KZGF0LmRvbF93YXRlci5maWwuNHNhbXAuY29yZS5iaW5hcnkuU3RhcmJ1Y2tfMiA9IGRhdC5kb2xfd2F0ZXIuZmlsLjRzYW1wLmNvcmUuYmluYXJ5LlN0YXJidWNrICU+JSBtdXRhdGUoc3VtX29mX2NvdW50cyA9IHJvd1N1bXMoZGF0LmRvbF93YXRlci5maWwuNHNhbXAuY29yZS5iaW5hcnkuU3RhcmJ1Y2spKQ0KDQptZWFuKGRhdC5kb2xfd2F0ZXIuZmlsLjRzYW1wLmNvcmUuYmluYXJ5LlN0YXJidWNrXzIkc3VtX29mX2NvdW50cykNCiM0MjgNCg0KIyMjIyMjIw0KI1doYXQncyB0aGUgcHJldmFsZW5jZSBvZiB0aGUgY29yZSB6T1RVcyBpbiBTdGFyYnVjayBvdmVyIHRoZSBmb3VyIHNhbXBsZXM/DQoNCiNWaWV3KGRhdC5kb2xfd2F0ZXIuZmlsLjRzYW1wLmNvcmUuU3RhcmJ1Y2tbLDE6MTBdKQ0KDQpDb3JlLlN0YXJidWNrID0gQ29yZS56T1RVLlN0YXJidWNrLjRzYW1wXzEuMCR2YXJpYWJsZQ0KDQpkYXQuZG9sX3dhdGVyLmZpbC40c2FtcC5jb3JlLlN0YXJidWNrXzIgPSBkYXQuZG9sX3dhdGVyLmZpbC40c2FtcC5jb3JlLlN0YXJidWNrL3Jvd1N1bXMoZGF0LmRvbF93YXRlci5maWwuNHNhbXAuY29yZS5TdGFyYnVjaykNCiNWaWV3KGRhdC5kb2xfd2F0ZXIuZmlsLjRzYW1wLmNvcmUuU3RhcmJ1Y2tfMikNCg0KZGF0LmRvbF93YXRlci5maWwuNHNhbXAuY29yZS5TdGFyYnVja18zID0gZGF0LmRvbF93YXRlci5maWwuNHNhbXAuY29yZS5TdGFyYnVja18yWyxDb3JlLlN0YXJidWNrXQ0KI1ZpZXcoZGF0LmRvbF93YXRlci5maWwuNHNhbXAuY29yZS5TdGFyYnVja18zKQ0KDQojcm93bmFtZXMoZGF0LmRvbF93YXRlci5maWwuNHNhbXAuY29yZS5TdGFyYnVja18zKQ0KI1sxXSAgIlN0YXJidWNrXzI4IiAiU3RhcmJ1Y2tfMTkiICJTdGFyYnVja18xMSIgIlN0YXJidWNrXzYiICAgDQoNCmRhdC5kb2xfd2F0ZXIuZmlsLjRzYW1wLmNvcmUuU3RhcmJ1Y2tfNCA9IGRhdC5kb2xfd2F0ZXIuZmlsLjRzYW1wLmNvcmUuU3RhcmJ1Y2tfMyAlPiUgbXV0YXRlKHN1bV9vZl9yb3dzID0gcm93U3VtcyhkYXQuZG9sX3dhdGVyLmZpbC40c2FtcC5jb3JlLlN0YXJidWNrXzMpKQ0KI1ZpZXcoZGF0LmRvbF93YXRlci5maWwuNHNhbXAuY29yZS5TdGFyYnVja180KQ0KZGF0LmRvbF93YXRlci5maWwuNHNhbXAuY29yZS5TdGFyYnVja180JHN1bV9vZl9yb3dzDQoNCiMiU3RhcmJ1Y2tfNiI6ICAwLjk2Nzk0NzYgDQojIlN0YXJidWNrXzExIjogMC45NTM4OTY3ICAgDQojIlN0YXJidWNrXzE5IjogMC45NjU4MjA4ICAgDQojIlN0YXJidWNrXzI4IjogMC44OTE5NTEyICAgDQoNCm1lYW4oZGF0LmRvbF93YXRlci5maWwuNHNhbXAuY29yZS5TdGFyYnVja180JHN1bV9vZl9yb3dzKQ0KIzAuOTQ0OTA0DQpgYGANCg0KIyMjSG93aWUNCmBgYHtyfQ0KIyMjSG93aWUNCiNDYWxjdWxhdGUgY29yZSB0YXhhIHRoYXQgMTAwICUgb2YgSG93aWUncyBzYW1wbGVzIGhhdmUgaW4gY29tbW9uDQojZGF0LmRvbF93YXRlci5maWwuNHNhbXAuY29yZQ0KDQpkYXQuZG9sX3dhdGVyLmZpbC40c2FtcC5jb3JlLkhvd2llIDwtICBkYXQuZG9sX3dhdGVyLmZpbC40c2FtcC5jb3JlW2MoOToxMiksXQ0KDQpkaW0oZGF0LmRvbF93YXRlci5maWwuNHNhbXAuY29yZS5Ib3dpZSkNCiM0IDE1NzENCg0KZGF0LmRvbF93YXRlci5maWwuNHNhbXAuY29yZS5iaW5hcnkuSG93aWUgPC0gZGF0LmRvbF93YXRlci5maWwuNHNhbXAuY29yZS5Ib3dpZQ0KDQojQ29udmVydCBkYXRhZnJhbWUgaW50byBiaW5hcnkNCmRhdC5kb2xfd2F0ZXIuZmlsLjRzYW1wLmNvcmUuYmluYXJ5Lkhvd2llW10gPC0gKyhkYXQuZG9sX3dhdGVyLmZpbC40c2FtcC5jb3JlLmJpbmFyeS5Ib3dpZSAgPiAwKQ0KI1ZpZXcoZGF0LmRvbF93YXRlci5maWwuNHNhbXAuY29yZS5iaW5hcnkuSG93aWUgWywxOjEwXSkNCg0KI1RyYW5zcG9zZSBkYXQuZG9sX3dhdGVyLmZpbC40c2FtcC5jb3JlLmJpbmFyeS5Ib3dpZQ0KZGF0LmRvbF93YXRlci5maWwuNHNhbXAuY29yZS5iaW5hcnkuSG93aWVfdCA8LSBhcy5kYXRhLmZyYW1lKHQoZGF0LmRvbF93YXRlci5maWwuNHNhbXAuY29yZS5iaW5hcnkuSG93aWUpKQ0KI1ZpZXcoZGF0LmRvbF93YXRlci5maWwuNHNhbXAuY29yZS5iaW5hcnkuSG93aWVfdFsxOjEwLF0pDQoNCiNDcmVhdGUgY29sIFRvdGFsLnJlbC5hYnVuZC4NCmRhdC5kb2xfd2F0ZXIuZmlsLjRzYW1wLmNvcmUuYmluYXJ5Lkhvd2llX3QuMiA8LSBkYXQuZG9sX3dhdGVyLmZpbC40c2FtcC5jb3JlLmJpbmFyeS5Ib3dpZV90ICU+JSANCiAgbXV0YXRlKFRvdGFsLnJlbC5hYnVuZC56T1RVID0gcm93U3VtcyhkYXQuZG9sX3dhdGVyLmZpbC40c2FtcC5jb3JlLmJpbmFyeS5Ib3dpZV90KS80KQ0KDQojVmlldyhkYXQuZG9sX3dhdGVyLmZpbC40c2FtcC5jb3JlLmJpbmFyeS5Ib3dpZV90LjJbMToxMCxdKQ0KDQojR2l2ZSBkYXQuZG9sX3dhdGVyLmZpbC40c2FtcC5jb3JlLmJpbmFyeS5Ib3dpZV90LjIgcm93bmFtZXMgYXMgY29sIHZhcmlhYmxlDQpkYXQuZG9sX3dhdGVyLmZpbC40c2FtcC5jb3JlLmJpbmFyeS5Ib3dpZV90LjIkdmFyaWFibGUgPC0gcm93bmFtZXMoZGF0LmRvbF93YXRlci5maWwuNHNhbXAuY29yZS5iaW5hcnkuSG93aWVfdCkNCmRpbShkYXQuZG9sX3dhdGVyLmZpbC40c2FtcC5jb3JlLmJpbmFyeS5Ib3dpZV90LjIpDQojMTQ3MSA2DQoNCiNSZW9yZGVyDQpkYXQuZG9sX3dhdGVyLmZpbC40c2FtcC5jb3JlLmJpbmFyeS5Ib3dpZV90LjMgPC0gZGF0LmRvbF93YXRlci5maWwuNHNhbXAuY29yZS5iaW5hcnkuSG93aWVfdC4yWyxjKDUsNiwxOjQpXQ0KDQojSm9pbiBkYXQuZG9sX3dhdGVyLmZpbC40c2FtcC5jb3JlLmJpbmFyeS5Ib3dpZV90LjMgd2l0aCBhdHRyaWJ1dGVzLm1pZ3JhdGlvbl9yZHANCkNvcmUuek9UVS5Ib3dpZS40c2FtcCA8LSBkYXQuZG9sX3dhdGVyLmZpbC40c2FtcC5jb3JlLmJpbmFyeS5Ib3dpZV90LjMgJT4lIGxlZnRfam9pbihhdHRyaWJ1dGVzLmRvbF9yZHBfd2F0ZXIuZmlsLjIpDQpkaW0oQ29yZS56T1RVLkhvd2llLjRzYW1wKQ0KIyAxNDcxICAgMTINCg0KI1ZpZXcoQ29yZS56T1RVLkhvd2llLjRzYW1wWzE6MTAsXSkNCg0KI1Jlb3JkZXINCkNvcmUuek9UVS5Ib3dpZS40c2FtcF8yID0gQ29yZS56T1RVLkhvd2llLjRzYW1wWyxjKDEsMiwxMSwzOjYpXQ0KZGltKENvcmUuek9UVS5Ib3dpZS40c2FtcF8yKQ0KIyMxNDcxICAgIDcNCg0KI25hbWVzKENvcmUuek9UVS5Ib3dpZS40c2FtcF8yKQ0KDQojT3JkZXIgYWNjb3JkaW5nIHRvIENvcmUuek9UVS5Ib3dpZS40c2FtcF8yJFRvdGFsLnJlbC5hYnVuZC56T1RVDQpDb3JlLnpPVFUuSG93aWUuNHNhbXBfMyA9IENvcmUuek9UVS5Ib3dpZS40c2FtcF8yW29yZGVyKENvcmUuek9UVS5Ib3dpZS40c2FtcF8yJFRvdGFsLnJlbC5hYnVuZC56T1RVKSxdDQojVmlldyhDb3JlLnpPVFUuSG93aWUuNHNhbXBfM1ssMToyXSkNCg0KI0FueSBjb3JlT1RVcyBhYm92ZSAxMDAlICg9PSAxLjApPw0KQ29yZS56T1RVLkhvd2llLjRzYW1wXzEuMCA9IENvcmUuek9UVS5Ib3dpZS40c2FtcF8zICU+JSBmaWx0ZXIgKFRvdGFsLnJlbC5hYnVuZC56T1RVID09IDEuMCkNCmRpbShDb3JlLnpPVFUuSG93aWUuNHNhbXBfMS4wKQ0KIzMwOSAgNw0KDQojIyMjIyMjDQojIyNIb3cgbWFueSB6T1RVcyBhcmUgcHJlc2VudCBwZXIgSG93aWUncyBzYW1wbGUgb24gYXZlcmFnZT8NCmRhdC5kb2xfd2F0ZXIuZmlsLjRzYW1wLmNvcmUuYmluYXJ5Lkhvd2llXzIgPSBkYXQuZG9sX3dhdGVyLmZpbC40c2FtcC5jb3JlLmJpbmFyeS5Ib3dpZSAlPiUgbXV0YXRlKHN1bV9vZl9jb3VudHMgPSByb3dTdW1zKGRhdC5kb2xfd2F0ZXIuZmlsLjRzYW1wLmNvcmUuYmluYXJ5Lkhvd2llKSkNCg0KbWVhbihkYXQuZG9sX3dhdGVyLmZpbC40c2FtcC5jb3JlLmJpbmFyeS5Ib3dpZV8yJHN1bV9vZl9jb3VudHMpDQojNDY1DQoNCiMjIyMjIyMNCiNXaGF0J3MgdGhlIHByZXZhbGVuY2Ugb2YgdGhlIGNvcmUgek9UVXMgaW4gSG93aWUgb3ZlciB0aGUgZm91ciBzYW1wbGVzPw0KDQojVmlldyhkYXQuZG9sX3dhdGVyLmZpbC40c2FtcC5jb3JlLkhvd2llWywxOjEwXSkNCg0KQ29yZS5Ib3dpZSA9IENvcmUuek9UVS5Ib3dpZS40c2FtcF8xLjAkdmFyaWFibGUNCg0KZGF0LmRvbF93YXRlci5maWwuNHNhbXAuY29yZS5Ib3dpZV8yID0gZGF0LmRvbF93YXRlci5maWwuNHNhbXAuY29yZS5Ib3dpZS9yb3dTdW1zKGRhdC5kb2xfd2F0ZXIuZmlsLjRzYW1wLmNvcmUuSG93aWUpDQojVmlldyhkYXQuZG9sX3dhdGVyLmZpbC40c2FtcC5jb3JlLkhvd2llXzIpDQoNCmRhdC5kb2xfd2F0ZXIuZmlsLjRzYW1wLmNvcmUuSG93aWVfMyA9IGRhdC5kb2xfd2F0ZXIuZmlsLjRzYW1wLmNvcmUuSG93aWVfMlssQ29yZS5Ib3dpZV0NCiNWaWV3KGRhdC5kb2xfd2F0ZXIuZmlsLjRzYW1wLmNvcmUuSG93aWVfMykNCg0KI3Jvd25hbWVzKGRhdC5kb2xfd2F0ZXIuZmlsLjRzYW1wLmNvcmUuSG93aWVfMykNCiNbMV0gICJIb3dpZV8yOCIgIkhvd2llXzE5IiAiSG93aWVfMTEiICJIb3dpZV82IiAgIA0KDQpkYXQuZG9sX3dhdGVyLmZpbC40c2FtcC5jb3JlLkhvd2llXzQgPSBkYXQuZG9sX3dhdGVyLmZpbC40c2FtcC5jb3JlLkhvd2llXzMgJT4lIG11dGF0ZShzdW1fb2Zfcm93cyA9IHJvd1N1bXMoZGF0LmRvbF93YXRlci5maWwuNHNhbXAuY29yZS5Ib3dpZV8zKSkNCiNWaWV3KGRhdC5kb2xfd2F0ZXIuZmlsLjRzYW1wLmNvcmUuSG93aWVfNCkNCmRhdC5kb2xfd2F0ZXIuZmlsLjRzYW1wLmNvcmUuSG93aWVfNCRzdW1fb2Zfcm93cw0KDQojIkhvd2llXzYiOiAgMC44Nzc3Mjc0IA0KIyJIb3dpZV8xMiI6IDAuOTU0NjI3MSANCiMiSG93aWVfMTkiOiAwLjc0ODYyOTggDQojIkhvd2llXzI4IjogMC44OTU0NjAzIA0KDQptZWFuKGRhdC5kb2xfd2F0ZXIuZmlsLjRzYW1wLmNvcmUuSG93aWVfNCRzdW1fb2Zfcm93cykNCiMwLjg2OTExMTINCmBgYA0KDQojIyNOdWRnZWUNCmBgYHtyfQ0KIyMjTnVkZ2VlDQojQ2FsY3VsYXRlIGNvcmUgdGF4YSB0aGF0IDEwMCAlIG9mIE51ZGdlZSdzIHNhbXBsZXMgaGF2ZSBpbiBjb21tb24NCiNkYXQuZG9sX3dhdGVyLmZpbC40c2FtcC5jb3JlDQoNCmRhdC5kb2xfd2F0ZXIuZmlsLjRzYW1wLmNvcmUuTnVkZ2VlIDwtICBkYXQuZG9sX3dhdGVyLmZpbC40c2FtcC5jb3JlW2MoMjE6MjQpLF0NCg0KZGltKGRhdC5kb2xfd2F0ZXIuZmlsLjRzYW1wLmNvcmUuTnVkZ2VlKQ0KIzQgMTU3MQ0KDQpkYXQuZG9sX3dhdGVyLmZpbC40c2FtcC5jb3JlLmJpbmFyeS5OdWRnZWUgPC0gZGF0LmRvbF93YXRlci5maWwuNHNhbXAuY29yZS5OdWRnZWUNCg0KI0NvbnZlcnQgZGF0YWZyYW1lIGludG8gYmluYXJ5DQpkYXQuZG9sX3dhdGVyLmZpbC40c2FtcC5jb3JlLmJpbmFyeS5OdWRnZWVbXSA8LSArKGRhdC5kb2xfd2F0ZXIuZmlsLjRzYW1wLmNvcmUuYmluYXJ5Lk51ZGdlZSAgPiAwKQ0KI1ZpZXcoZGF0LmRvbF93YXRlci5maWwuNHNhbXAuY29yZS5iaW5hcnkuTnVkZ2VlIFssMToxMF0pDQoNCiNUcmFuc3Bvc2UgZGF0LmRvbF93YXRlci5maWwuNHNhbXAuY29yZS5iaW5hcnkuTnVkZ2VlDQpkYXQuZG9sX3dhdGVyLmZpbC40c2FtcC5jb3JlLmJpbmFyeS5OdWRnZWVfdCA8LSBhcy5kYXRhLmZyYW1lKHQoZGF0LmRvbF93YXRlci5maWwuNHNhbXAuY29yZS5iaW5hcnkuTnVkZ2VlKSkNCiNWaWV3KGRhdC5kb2xfd2F0ZXIuZmlsLjRzYW1wLmNvcmUuYmluYXJ5Lk51ZGdlZV90WzE6MTAsXSkNCg0KI0NyZWF0ZSBjb2wgVG90YWwucmVsLmFidW5kLg0KZGF0LmRvbF93YXRlci5maWwuNHNhbXAuY29yZS5iaW5hcnkuTnVkZ2VlX3QuMiA8LSBkYXQuZG9sX3dhdGVyLmZpbC40c2FtcC5jb3JlLmJpbmFyeS5OdWRnZWVfdCAlPiUgDQogIG11dGF0ZShUb3RhbC5yZWwuYWJ1bmQuek9UVSA9IHJvd1N1bXMoZGF0LmRvbF93YXRlci5maWwuNHNhbXAuY29yZS5iaW5hcnkuTnVkZ2VlX3QpLzQpDQoNCiNWaWV3KGRhdC5kb2xfd2F0ZXIuZmlsLjRzYW1wLmNvcmUuYmluYXJ5Lk51ZGdlZV90LjJbMToxMCxdKQ0KDQojR2l2ZSBkYXQuZG9sX3dhdGVyLmZpbC40c2FtcC5jb3JlLmJpbmFyeS5OdWRnZWVfdC4yIHJvd25hbWVzIGFzIGNvbCB2YXJpYWJsZQ0KZGF0LmRvbF93YXRlci5maWwuNHNhbXAuY29yZS5iaW5hcnkuTnVkZ2VlX3QuMiR2YXJpYWJsZSA8LSByb3duYW1lcyhkYXQuZG9sX3dhdGVyLmZpbC40c2FtcC5jb3JlLmJpbmFyeS5OdWRnZWVfdCkNCmRpbShkYXQuZG9sX3dhdGVyLmZpbC40c2FtcC5jb3JlLmJpbmFyeS5OdWRnZWVfdC4yKQ0KIzE0NzEgNg0KDQojUmVvcmRlcg0KZGF0LmRvbF93YXRlci5maWwuNHNhbXAuY29yZS5iaW5hcnkuTnVkZ2VlX3QuMyA8LSBkYXQuZG9sX3dhdGVyLmZpbC40c2FtcC5jb3JlLmJpbmFyeS5OdWRnZWVfdC4yWyxjKDUsNiwxOjQpXQ0KDQojSm9pbiBkYXQuZG9sX3dhdGVyLmZpbC40c2FtcC5jb3JlLmJpbmFyeS5OdWRnZWVfdC4zIHdpdGggYXR0cmlidXRlcy5taWdyYXRpb25fcmRwDQpDb3JlLnpPVFUuTnVkZ2VlLjRzYW1wIDwtIGRhdC5kb2xfd2F0ZXIuZmlsLjRzYW1wLmNvcmUuYmluYXJ5Lk51ZGdlZV90LjMgJT4lIGxlZnRfam9pbihhdHRyaWJ1dGVzLmRvbF9yZHBfd2F0ZXIuZmlsLjIpDQpkaW0oQ29yZS56T1RVLk51ZGdlZS40c2FtcCkNCiMgMTQ3MSAgIDEyDQoNCiNWaWV3KENvcmUuek9UVS5OdWRnZWUuNHNhbXBbMToxMCxdKQ0KDQojUmVvcmRlcg0KQ29yZS56T1RVLk51ZGdlZS40c2FtcF8yID0gQ29yZS56T1RVLk51ZGdlZS40c2FtcFssYygxLDIsMTEsMzo2KV0NCmRpbShDb3JlLnpPVFUuTnVkZ2VlLjRzYW1wXzIpDQojIzE0NzEgICAgNw0KDQojbmFtZXMoQ29yZS56T1RVLk51ZGdlZS40c2FtcF8yKQ0KDQojT3JkZXIgYWNjb3JkaW5nIHRvIENvcmUuek9UVS5OdWRnZWUuNHNhbXBfMiRUb3RhbC5yZWwuYWJ1bmQuek9UVQ0KQ29yZS56T1RVLk51ZGdlZS40c2FtcF8zID0gQ29yZS56T1RVLk51ZGdlZS40c2FtcF8yW29yZGVyKENvcmUuek9UVS5OdWRnZWUuNHNhbXBfMiRUb3RhbC5yZWwuYWJ1bmQuek9UVSksXQ0KI1ZpZXcoQ29yZS56T1RVLk51ZGdlZS40c2FtcF8zWywxOjJdKQ0KDQojQW55IGNvcmVPVFVzIGFib3ZlIDEwMCUgKD09IDEuMCk/DQpDb3JlLnpPVFUuTnVkZ2VlLjRzYW1wXzEuMCA9IENvcmUuek9UVS5OdWRnZWUuNHNhbXBfMyAlPiUgZmlsdGVyIChUb3RhbC5yZWwuYWJ1bmQuek9UVSA9PSAxLjApDQpkaW0oQ29yZS56T1RVLk51ZGdlZS40c2FtcF8xLjApDQojMTE1ICAgNw0KDQojIyMjIyMjDQojIyNIb3cgbWFueSB6T1RVcyBhcmUgcHJlc2VudCBwZXIgTnVkZ2VlJ3Mgc2FtcGxlIG9uIGF2ZXJhZ2U/DQpkYXQuZG9sX3dhdGVyLmZpbC40c2FtcC5jb3JlLmJpbmFyeS5OdWRnZWVfMiA9IGRhdC5kb2xfd2F0ZXIuZmlsLjRzYW1wLmNvcmUuYmluYXJ5Lk51ZGdlZSAlPiUgbXV0YXRlKHN1bV9vZl9jb3VudHMgPSByb3dTdW1zKGRhdC5kb2xfd2F0ZXIuZmlsLjRzYW1wLmNvcmUuYmluYXJ5Lk51ZGdlZSkpDQoNCm1lYW4oZGF0LmRvbF93YXRlci5maWwuNHNhbXAuY29yZS5iaW5hcnkuTnVkZ2VlXzIkc3VtX29mX2NvdW50cykNCiMyNDUNCg0KIyMjIyMjIw0KI1doYXQncyB0aGUgcHJldmFsZW5jZSBvZiB0aGUgY29yZSB6T1RVcyBpbiBOdWRnZWUgb3ZlciB0aGUgZm91ciBzYW1wbGVzPw0KDQojVmlldyhkYXQuZG9sX3dhdGVyLmZpbC40c2FtcC5jb3JlLk51ZGdlZVssMToxMF0pDQoNCkNvcmUuTnVkZ2VlID0gQ29yZS56T1RVLk51ZGdlZS40c2FtcF8xLjAkdmFyaWFibGUNCg0KZGF0LmRvbF93YXRlci5maWwuNHNhbXAuY29yZS5OdWRnZWVfMiA9IGRhdC5kb2xfd2F0ZXIuZmlsLjRzYW1wLmNvcmUuTnVkZ2VlL3Jvd1N1bXMoZGF0LmRvbF93YXRlci5maWwuNHNhbXAuY29yZS5OdWRnZWUpDQojVmlldyhkYXQuZG9sX3dhdGVyLmZpbC40c2FtcC5jb3JlLk51ZGdlZV8yKQ0KDQpkYXQuZG9sX3dhdGVyLmZpbC40c2FtcC5jb3JlLk51ZGdlZV8zID0gZGF0LmRvbF93YXRlci5maWwuNHNhbXAuY29yZS5OdWRnZWVfMlssQ29yZS5OdWRnZWVdDQojVmlldyhkYXQuZG9sX3dhdGVyLmZpbC40c2FtcC5jb3JlLk51ZGdlZV8zKQ0KDQojcm93bmFtZXMoZGF0LmRvbF93YXRlci5maWwuNHNhbXAuY29yZS5OdWRnZWVfMykNCg0KZGF0LmRvbF93YXRlci5maWwuNHNhbXAuY29yZS5OdWRnZWVfNCA9IGRhdC5kb2xfd2F0ZXIuZmlsLjRzYW1wLmNvcmUuTnVkZ2VlXzMgJT4lIG11dGF0ZShzdW1fb2Zfcm93cyA9IHJvd1N1bXMoZGF0LmRvbF93YXRlci5maWwuNHNhbXAuY29yZS5OdWRnZWVfMykpDQojVmlldyhkYXQuZG9sX3dhdGVyLmZpbC40c2FtcC5jb3JlLk51ZGdlZV80KQ0KZGF0LmRvbF93YXRlci5maWwuNHNhbXAuY29yZS5OdWRnZWVfNCRzdW1fb2Zfcm93cw0KDQojIk51ZGdlZV82IjogIDAuODM3NDc3NSANCiMiTnVkZ2VlXzEyIjogMC41OTY0MTg0IA0KIyJOdWRnZWVfMjAiOiAwLjkxOTg3NjUgDQojIk51ZGdlZV8yOCI6IDAuNzk2MDUwMCANCg0KbWVhbihkYXQuZG9sX3dhdGVyLmZpbC40c2FtcC5jb3JlLk51ZGdlZV80JHN1bV9vZl9yb3dzKQ0KIzAuNzg3NDU1Ng0KYGBgDQoNCiMjIyMjIyMjIyMjIyMjIyMjIyMjIyMjIyMjIyMjIyMjIyMjIyMjIyMjIyMjIA0KIyMjIyNEbyBkb2xwaGlucyBoYXZlIHNpbWlsYXIgaW50cmEtY29yZSB6T1RVcz8gTGV0J3MgY2hlY2shDQpgYGB7cn0NCiM0c2FtcGxlcyAoVU5yYXJlZmllZCkNCiMxMDAlDQpDb3JlLnpPVFUuNHNhbXBfMS4wID0gYyhDb3JlLnpPVFUuQ29lbi40c2FtcF8xLjAkdmFyaWFibGUsQ29yZS56T1RVLkV2aWUuNHNhbXBfMS4wJHZhcmlhYmxlLCBDb3JlLnpPVFUuS2lhbWEuNHNhbXBfMS4wJHZhcmlhYmxlLENvcmUuek9UVS5Nb2tpLjRzYW1wXzEuMCR2YXJpYWJsZSwNCiAgICAgICAgICAgICAgICAgICAgICAgQ29yZS56T1RVLlJCLjRzYW1wXzEuMCR2YXJpYWJsZSxDb3JlLnpPVFUuU2Nvb3Rlci40c2FtcF8xLjAkdmFyaWFibGUsDQogICAgICAgICAgICAgICAgICAgICAgIENvcmUuek9UVS5TaXJpdXMuNHNhbXBfMS4wJHZhcmlhYmxlLENvcmUuek9UVS5TcXVlYWsuNHNhbXBfMS4wJHZhcmlhYmxlLA0KICAgICAgICAgICAgICAgICAgICAgICBDb3JlLnpPVFUuU3RhcmJ1Y2suNHNhbXBfMS4wJHZhcmlhYmxlLENvcmUuek9UVS5Ib3dpZS40c2FtcF8xLjAkdmFyaWFibGUsDQogICAgICAgICAgICAgICAgICAgICAgIENvcmUuek9UVS5OdWRnZWUuNHNhbXBfMS4wJHZhcmlhYmxlKQ0KDQpsZW5ndGgoQ29yZS56T1RVLjRzYW1wXzEuMCkNCiMxNzAzDQpsZW5ndGgodW5pcXVlKENvcmUuek9UVS40c2FtcF8xLjApKQ0KIzUwMw0KDQpDb3JlLnpPVFUuNHNhbXBfMS4wLnVuaXF1ZSA9IHVuaXF1ZShDb3JlLnpPVFUuNHNhbXBfMS4wKQ0Kd3JpdGUuY3N2KENvcmUuek9UVS40c2FtcF8xLjAudW5pcXVlLGZpbGU9J0NvcmUuek9UVS40c2FtcF8xLjAudW5pcXVlX05FVy5jc3YnKQ0KYGBgDQoNCiMjIyMjIyMjIyMjIyMjIyMjIyMjICMjIyMjIyMjIyMjIyMjIyMjIyMjDQojIyNEZXRlcm1pbmUgcmVsLiBhYnVuZC4gb2YgaW50cmEtY29yZSB6T1RVcw0KYGBge3J9DQojIyNEZXRlcm1pbmUgcmVsLiBhYnVuZC4gb2YgaW50cmEtY29yZSB6T1RVcw0KDQpkaW0oZGF0LmRvbF93YXRlci5maWwuNHNhbXAuY29yZSkNCiM0NSAxNDcxDQoNCiNWaWV3KGRhdC5kb2xfd2F0ZXIuZmlsLjRzYW1wLmNvcmUpDQoNCmRhdC5kb2xfd2F0ZXIuZmlsLjRzYW1wLmNvcmUucmVsLmFidW5kID0gZGF0LmRvbF93YXRlci5maWwuNHNhbXAuY29yZS9yb3dTdW1zKGRhdC5kb2xfd2F0ZXIuZmlsLjRzYW1wLmNvcmUpDQoNCiNUcmFuc3Bvc2UNCmRhdC5kb2xfd2F0ZXIuZmlsLjRzYW1wLmNvcmUucmVsLmFidW5kX3QgPSBhcy5kYXRhLmZyYW1lKHQoZGF0LmRvbF93YXRlci5maWwuNHNhbXAuY29yZS5yZWwuYWJ1bmQpKQ0KDQpkYXQuZG9sX3dhdGVyLmZpbC40c2FtcC5jb3JlLnJlbC5hYnVuZF90LjIgPSBkYXQuZG9sX3dhdGVyLmZpbC40c2FtcC5jb3JlLnJlbC5hYnVuZF90ICU+JSBtdXRhdGUgKG1lYW4ucmVsLmFidW5kID0gcm93TWVhbnMoZGF0LmRvbF93YXRlci5maWwuNHNhbXAuY29yZS5yZWwuYWJ1bmRfdCksIHNkLnJlbC5hYnVuZCA9IHJvd1NkcyhkYXQuZG9sX3dhdGVyLmZpbC40c2FtcC5jb3JlLnJlbC5hYnVuZF90KSkNCg0Kcm93bmFtZXMoZGF0LmRvbF93YXRlci5maWwuNHNhbXAuY29yZS5yZWwuYWJ1bmRfdC4yKSA9IHJvd25hbWVzKGRhdC5kb2xfd2F0ZXIuZmlsLjRzYW1wLmNvcmUucmVsLmFidW5kX3QpDQoNCmRhdC5kb2xfd2F0ZXIuZmlsLjRzYW1wLmNvcmUucmVsLmFidW5kX3QuMiRyb3dfbWluaW11bSA9IGFwcGx5KGRhdC5kb2xfd2F0ZXIuZmlsLjRzYW1wLmNvcmUucmVsLmFidW5kX3QsIDEsIG1pbikNCg0KZGF0LmRvbF93YXRlci5maWwuNHNhbXAuY29yZS5yZWwuYWJ1bmRfdC4yJHJvd19tYXggPSBhcHBseShkYXQuZG9sX3dhdGVyLmZpbC40c2FtcC5jb3JlLnJlbC5hYnVuZF90LCAxLCBtYXgpDQoNCmRpbShkYXQuZG9sX3dhdGVyLmZpbC40c2FtcC5jb3JlLnJlbC5hYnVuZF90LjIpDQojMTQ3MSAgIDQ5DQoNCmRhdC5kb2xfd2F0ZXIuZmlsLjRzYW1wLmNvcmUucmVsLmFidW5kX3QuMyA9IGRhdC5kb2xfd2F0ZXIuZmlsLjRzYW1wLmNvcmUucmVsLmFidW5kX3QuMg0KDQpkYXQuZG9sX3dhdGVyLmZpbC40c2FtcC5jb3JlLnJlbC5hYnVuZF90LjMkdmFyaWFibGUgPSByb3duYW1lcyhkYXQuZG9sX3dhdGVyLmZpbC40c2FtcC5jb3JlLnJlbC5hYnVuZF90LjMpDQoNCmRhdC5kb2xfd2F0ZXIuZmlsLjRzYW1wLmNvcmUucmVsLmFidW5kX3QuNCA9IGRhdC5kb2xfd2F0ZXIuZmlsLjRzYW1wLmNvcmUucmVsLmFidW5kX3QuM1ssNDY6NTBdDQoNCmRhdC5kb2xfd2F0ZXIuZmlsLjRzYW1wLmNvcmUucmVsLmFidW5kX3QuNSA9IGRhdC5kb2xfd2F0ZXIuZmlsLjRzYW1wLmNvcmUucmVsLmFidW5kX3QuNCAlPiUgZmlsdGVyKG1lYW4ucmVsLmFidW5kID4gMCkNCg0Kcm93bmFtZXMoZGF0LmRvbF93YXRlci5maWwuNHNhbXAuY29yZS5yZWwuYWJ1bmRfdC41KSA9IGRhdC5kb2xfd2F0ZXIuZmlsLjRzYW1wLmNvcmUucmVsLmFidW5kX3QuNSR2YXJpYWJsZQ0KZGltKGRhdC5kb2xfd2F0ZXIuZmlsLjRzYW1wLmNvcmUucmVsLmFidW5kX3QuNSkNCiM4NzEgICAgMw0KDQptZWFuKGRhdC5kb2xfd2F0ZXIuZmlsLjRzYW1wLmNvcmUucmVsLmFidW5kX3QuNSRtZWFuLnJlbC5hYnVuZCkNCiMwLjAwMTE0ODEwNg0KbWVkaWFuKGRhdC5kb2xfd2F0ZXIuZmlsLjRzYW1wLmNvcmUucmVsLmFidW5kX3QuNSRtZWFuLnJlbC5hYnVuZCkNCiMwLjAwMDE5MTEyNw0Kc2QoZGF0LmRvbF93YXRlci5maWwuNHNhbXAuY29yZS5yZWwuYWJ1bmRfdC41JG1lYW4ucmVsLmFidW5kKQ0KIzAuMDAzOTQ4ODENCg0KZGF0LmRvbF93YXRlci5maWwuNHNhbXAuY29yZS5yZWwuYWJ1bmRfdC42IDwtIGRhdC5kb2xfd2F0ZXIuZmlsLjRzYW1wLmNvcmUucmVsLmFidW5kX3QuNQ0KDQpyb3duYW1lcyhkYXQuZG9sX3dhdGVyLmZpbC40c2FtcC5jb3JlLnJlbC5hYnVuZF90LjYpIDwtIGRhdC5kb2xfd2F0ZXIuZmlsLjRzYW1wLmNvcmUucmVsLmFidW5kX3QuNiR2YXJpYWJsZQ0KDQpkYXQuZG9sX3dhdGVyLmZpbC40c2FtcC5jb3JlLnJlbC5hYnVuZF90LmNvcmUgPSBkYXQuZG9sX3dhdGVyLmZpbC40c2FtcC5jb3JlLnJlbC5hYnVuZF90LjZbQ29yZS56T1RVLjRzYW1wXzEuMC51bmlxdWUsXQ0KZGltKGRhdC5kb2xfd2F0ZXIuZmlsLjRzYW1wLmNvcmUucmVsLmFidW5kX3QuY29yZSkNCiM1MDMgICAzDQojVmlldyhkYXQuZG9sX3dhdGVyLmZpbC40c2FtcC5jb3JlLnJlbC5hYnVuZF90LmNvcmUpDQoNCmRhdC5kb2xfd2F0ZXIuZmlsLjRzYW1wLmNvcmUucmVsLmFidW5kX3QuY29yZS4yID0gZGF0LmRvbF93YXRlci5maWwuNHNhbXAuY29yZS5yZWwuYWJ1bmRfdC5jb3JlW29yZGVyKGRhdC5kb2xfd2F0ZXIuZmlsLjRzYW1wLmNvcmUucmVsLmFidW5kX3QuY29yZSRtZWFuLnJlbC5hYnVuZCksXQ0KI1ZpZXcoZGF0LmRvbF93YXRlci5maWwuNHNhbXAuY29yZS5yZWwuYWJ1bmRfdC5jb3JlLjIpDQoNCnN1bShkYXQuZG9sX3dhdGVyLmZpbC40c2FtcC5jb3JlLnJlbC5hYnVuZF90LmNvcmUuMiRtZWFuLnJlbC5hYnVuZCkNCiMwLjk1NTA3Ng0KDQpkYXQuZG9sX3dhdGVyLmZpbC40c2FtcC5jb3JlLnJlbC5hYnVuZF90LmNvcmUuMiA8LSBkYXQuZG9sX3dhdGVyLmZpbC40c2FtcC5jb3JlLnJlbC5hYnVuZF90LmNvcmUuMltvcmRlcihkYXQuZG9sX3dhdGVyLmZpbC40c2FtcC5jb3JlLnJlbC5hYnVuZF90LmNvcmUuMiRtZWFuLnJlbC5hYnVuZCksXQ0KDQp3cml0ZS5jc3YoZGF0LmRvbF93YXRlci5maWwuNHNhbXAuY29yZS5yZWwuYWJ1bmRfdC5jb3JlLjIsIGZpbGU9J1JlbC5hYnVuZC56T1RVLmRvbC5jb3JlLmNzdicpDQoNCiMjIyMjIyMjIyMjIyMjDQojIyNSZWxhdGl2ZSBhYnVuZGFuY2Ugb2YgZG9scGhpbi1yZWxhdGVkIGludHJhLWNvcmUgaW4gZG9scGhpbnMNCg0KZGltKGRhdC5kb2xfd2F0ZXIuZmlsLjRzYW1wLmNvcmUucmVsLmFidW5kX3QuY29yZS4yKQ0KIzUwMyAyDQoNCkludHJhY29yZV9Eb2xwaGluLnJlbGF0ZWQgPC0gYygnWm90dTMxOScsJ1pvdHUxODQzJywnWm90dTIwJywnWm90dTIxNTcnLCdab3R1MycsJ1pvdHU0MycsICdab3R1NTgnLCdab3R1NzAnLCdab3R1NzI2JywnWm90dTgnLCdab3R1ODQzJw0KLCdab3R1OScsJ1pvdHUyMzEnLCdab3R1NTAxJywnWm90dTU2MCcsJ1pvdHU2NCcsJ1pvdHU5OTUnLCAnWm90dTY0OCcsJ1pvdHUyMDQ1JywnWm90dTU5JywnWm90dTQ0MycsJ1pvdHU1OTEnLCdab3R1MjInDQosJ1pvdHU4OScsJ1pvdHU0OTgnLCdab3R1NzI5JywnWm90dTIwMycsJ1pvdHUzMTQnLCAnWm90dTYnLCdab3R1ODcnLCdab3R1MjknLCdab3R1NDAzJywnWm90dTgwNicsJ1pvdHU4NjYnLCdab3R1MjM2MicsJ1pvdHU0MicsICdab3R1NzYwJywnWm90dTYyJywnWm90dTg2JywnWm90dTI4MScsJ1pvdHU4MCcsJ1pvdHU0NzcnLCdab3R1MjM0OCcsJ1pvdHUyNjI3JywgJ1pvdHUzMjgnLCAnWm90dTQwJyAsJ1pvdHUzOCcsJ1pvdHU0JywnWm90dTM0MycsJ1pvdHU5MycsJ1pvdHUyNDQnLCdab3R1Mzg4JykNCg0KZGF0LmRvbF93YXRlci5maWwuNHNhbXAuY29yZS5yZWwuYWJ1bmRfdC5jb3JlLjIubmV3IDwtICBkYXQuZG9sX3dhdGVyLmZpbC40c2FtcC5jb3JlLnJlbC5hYnVuZF90LmNvcmUuMltJbnRyYWNvcmVfRG9scGhpbi5yZWxhdGVkLF0NCg0KZGltKGRhdC5kb2xfd2F0ZXIuZmlsLjRzYW1wLmNvcmUucmVsLmFidW5kX3QuY29yZS4yLm5ldykNCiM1MiAgMg0KDQpzdW0oZGF0LmRvbF93YXRlci5maWwuNHNhbXAuY29yZS5yZWwuYWJ1bmRfdC5jb3JlLjIubmV3JG1lYW4ucmVsLmFidW5kKQ0KIzAuMzgzMDExDQoNCmBgYA0KDQojIyMjIyMjIyMjIyMjIyMjIyMjIyMjIyMjIyMjIyMjIyMjIyMjIyMjIyMjIyMjIyMjIyMjIyMjIyMjIyMNCiMjI0NyZWF0ZSBsaW5lIHBsb3Qgd2l0aCByZWwuYWJ1bmQuIG9mIGNvcmUNCmBgYHtyfQ0KIyMjV2UgbmVlZCB0byBjcmVhdGUgYSBkYXRhZnJhbWUgdGhhdCBoYXMgY29scyBJRCAoZG9scGhpbiBuYW1lcyksIElELjIgKCdOdWRnZWVfNicpLCBXZWUgJiBSZWwuYWJ1bmQuY29yZS4NCg0KIyMjQ29lbg0KY29yZS5Db2VuLnJlbC5hYnVuZCA8LSBhcy5kYXRhLmZyYW1lKGRhdC5kb2xfd2F0ZXIuZmlsLjRzYW1wLmNvcmUuQ29lbl80JHN1bV9vZl9yb3dzKQ0KbmFtZXMoY29yZS5Db2VuLnJlbC5hYnVuZCkgPC0gJ1JlbC5hYnVuZC5vZi5jb3JlJw0KDQpjb3JlLkNvZW4ucmVsLmFidW5kLjIgPC0gY29yZS5Db2VuLnJlbC5hYnVuZA0KY29yZS5Db2VuLnJlbC5hYnVuZC4yJElEIDwtIHJvd25hbWVzKGNvcmUuQ29lbi5yZWwuYWJ1bmQuMikNCg0KY29yZS5Db2VuLnJlbC5hYnVuZC4zIDwtIGNvcmUuQ29lbi5yZWwuYWJ1bmQuMg0KY29yZS5Db2VuLnJlbC5hYnVuZC4zJElELjIgPC0gY29yZS5Db2VuLnJlbC5hYnVuZC4zJElEDQoNCmNvcmUuQ29lbi5yZWwuYWJ1bmQuNCA9IHNlcGFyYXRlKGNvcmUuQ29lbi5yZWwuYWJ1bmQuMywgSUQsIGMoJ0lEJywnV2VlaycpKQ0KDQojIyNFdmllDQpjb3JlLkV2aWUucmVsLmFidW5kIDwtIGFzLmRhdGEuZnJhbWUoZGF0LmRvbF93YXRlci5maWwuNHNhbXAuY29yZS5FdmllXzQkc3VtX29mX3Jvd3MpDQpuYW1lcyhjb3JlLkV2aWUucmVsLmFidW5kKSA8LSAnUmVsLmFidW5kLm9mLmNvcmUnDQoNCmNvcmUuRXZpZS5yZWwuYWJ1bmQuMiA8LSBjb3JlLkV2aWUucmVsLmFidW5kDQpjb3JlLkV2aWUucmVsLmFidW5kLjIkSUQgPC0gcm93bmFtZXMoY29yZS5FdmllLnJlbC5hYnVuZC4yKQ0KDQpjb3JlLkV2aWUucmVsLmFidW5kLjMgPC0gY29yZS5FdmllLnJlbC5hYnVuZC4yDQpjb3JlLkV2aWUucmVsLmFidW5kLjMkSUQuMiA8LSBjb3JlLkV2aWUucmVsLmFidW5kLjMkSUQNCg0KY29yZS5FdmllLnJlbC5hYnVuZC40ID0gc2VwYXJhdGUoY29yZS5FdmllLnJlbC5hYnVuZC4zLCBJRCwgYygnSUQnLCdXZWVrJykpDQoNCiMjI0tpYW1hDQpjb3JlLktpYW1hLnJlbC5hYnVuZCA8LSBhcy5kYXRhLmZyYW1lKGRhdC5kb2xfd2F0ZXIuZmlsLjRzYW1wLmNvcmUuS2lhbWFfNCRzdW1fb2Zfcm93cykNCg0KbmFtZXMoY29yZS5LaWFtYS5yZWwuYWJ1bmQpIDwtICdSZWwuYWJ1bmQub2YuY29yZScNCg0KY29yZS5LaWFtYS5yZWwuYWJ1bmQuMiA8LSBjb3JlLktpYW1hLnJlbC5hYnVuZA0KY29yZS5LaWFtYS5yZWwuYWJ1bmQuMiRJRCA8LSByb3duYW1lcyhjb3JlLktpYW1hLnJlbC5hYnVuZC4yKQ0KDQpjb3JlLktpYW1hLnJlbC5hYnVuZC4zIDwtIGNvcmUuS2lhbWEucmVsLmFidW5kLjINCmNvcmUuS2lhbWEucmVsLmFidW5kLjMkSUQuMiA8LSBjb3JlLktpYW1hLnJlbC5hYnVuZC4zJElEDQoNCmNvcmUuS2lhbWEucmVsLmFidW5kLjQgPSBzZXBhcmF0ZShjb3JlLktpYW1hLnJlbC5hYnVuZC4zLCBJRCwgYygnSUQnLCdXZWVrJykpDQoNCiMjI01va2kNCmNvcmUuTW9raS5yZWwuYWJ1bmQgPC0gYXMuZGF0YS5mcmFtZShkYXQuZG9sX3dhdGVyLmZpbC40c2FtcC5jb3JlLk1va2lfNCRzdW1fb2Zfcm93cykNCg0KbmFtZXMoY29yZS5Nb2tpLnJlbC5hYnVuZCkgPC0gJ1JlbC5hYnVuZC5vZi5jb3JlJw0KDQpjb3JlLk1va2kucmVsLmFidW5kLjIgPC0gY29yZS5Nb2tpLnJlbC5hYnVuZA0KY29yZS5Nb2tpLnJlbC5hYnVuZC4yJElEIDwtIHJvd25hbWVzKGNvcmUuTW9raS5yZWwuYWJ1bmQuMikNCg0KY29yZS5Nb2tpLnJlbC5hYnVuZC4zIDwtIGNvcmUuTW9raS5yZWwuYWJ1bmQuMg0KY29yZS5Nb2tpLnJlbC5hYnVuZC4zJElELjIgPC0gY29yZS5Nb2tpLnJlbC5hYnVuZC4zJElEDQoNCmNvcmUuTW9raS5yZWwuYWJ1bmQuNCA9IHNlcGFyYXRlKGNvcmUuTW9raS5yZWwuYWJ1bmQuMywgSUQsIGMoJ0lEJywnV2VlaycpKQ0KDQojIyNSQg0KY29yZS5SQi5yZWwuYWJ1bmQgPC0gYXMuZGF0YS5mcmFtZShkYXQuZG9sX3dhdGVyLmZpbC40c2FtcC5jb3JlLlJCXzQkc3VtX29mX3Jvd3MpDQoNCm5hbWVzKGNvcmUuUkIucmVsLmFidW5kKSA8LSAnUmVsLmFidW5kLm9mLmNvcmUnDQoNCmNvcmUuUkIucmVsLmFidW5kLjIgPC0gY29yZS5SQi5yZWwuYWJ1bmQNCmNvcmUuUkIucmVsLmFidW5kLjIkSUQgPC0gcm93bmFtZXMoY29yZS5SQi5yZWwuYWJ1bmQuMikNCg0KY29yZS5SQi5yZWwuYWJ1bmQuMyA8LSBjb3JlLlJCLnJlbC5hYnVuZC4yDQpjb3JlLlJCLnJlbC5hYnVuZC4zJElELjIgPC0gY29yZS5SQi5yZWwuYWJ1bmQuMyRJRA0KDQpjb3JlLlJCLnJlbC5hYnVuZC40ID0gc2VwYXJhdGUoY29yZS5SQi5yZWwuYWJ1bmQuMywgSUQsIGMoJ0lEJywnV2VlaycpKQ0KDQojIyNTY29vdGVyDQpjb3JlLlNjb290ZXIucmVsLmFidW5kIDwtIGFzLmRhdGEuZnJhbWUoZGF0LmRvbF93YXRlci5maWwuNHNhbXAuY29yZS5TY29vdGVyXzQkc3VtX29mX3Jvd3MpDQoNCm5hbWVzKGNvcmUuU2Nvb3Rlci5yZWwuYWJ1bmQpIDwtICdSZWwuYWJ1bmQub2YuY29yZScNCg0KY29yZS5TY29vdGVyLnJlbC5hYnVuZC4yIDwtIGNvcmUuU2Nvb3Rlci5yZWwuYWJ1bmQNCmNvcmUuU2Nvb3Rlci5yZWwuYWJ1bmQuMiRJRCA8LSByb3duYW1lcyhjb3JlLlNjb290ZXIucmVsLmFidW5kLjIpDQoNCmNvcmUuU2Nvb3Rlci5yZWwuYWJ1bmQuMyA8LSBjb3JlLlNjb290ZXIucmVsLmFidW5kLjINCmNvcmUuU2Nvb3Rlci5yZWwuYWJ1bmQuMyRJRC4yIDwtIGNvcmUuU2Nvb3Rlci5yZWwuYWJ1bmQuMyRJRA0KDQpjb3JlLlNjb290ZXIucmVsLmFidW5kLjQgPSBzZXBhcmF0ZShjb3JlLlNjb290ZXIucmVsLmFidW5kLjMsIElELCBjKCdJRCcsJ1dlZWsnKSkNCg0KIyMjU2lyaXVzDQpjb3JlLlNpcml1cy5yZWwuYWJ1bmQgPC0gYXMuZGF0YS5mcmFtZShkYXQuZG9sX3dhdGVyLmZpbC40c2FtcC5jb3JlLlNpcml1c180JHN1bV9vZl9yb3dzKQ0KDQpuYW1lcyhjb3JlLlNpcml1cy5yZWwuYWJ1bmQpIDwtICdSZWwuYWJ1bmQub2YuY29yZScNCg0KY29yZS5TaXJpdXMucmVsLmFidW5kLjIgPC0gY29yZS5TaXJpdXMucmVsLmFidW5kDQpjb3JlLlNpcml1cy5yZWwuYWJ1bmQuMiRJRCA8LSByb3duYW1lcyhjb3JlLlNpcml1cy5yZWwuYWJ1bmQuMikNCg0KY29yZS5TaXJpdXMucmVsLmFidW5kLjMgPC0gY29yZS5TaXJpdXMucmVsLmFidW5kLjINCmNvcmUuU2lyaXVzLnJlbC5hYnVuZC4zJElELjIgPC0gY29yZS5TaXJpdXMucmVsLmFidW5kLjMkSUQNCg0KY29yZS5TaXJpdXMucmVsLmFidW5kLjQgPSBzZXBhcmF0ZShjb3JlLlNpcml1cy5yZWwuYWJ1bmQuMywgSUQsIGMoJ0lEJywnV2VlaycpKQ0KDQojIyNTcXVlYWsNCmNvcmUuU3F1ZWFrLnJlbC5hYnVuZCA8LSBhcy5kYXRhLmZyYW1lKGRhdC5kb2xfd2F0ZXIuZmlsLjRzYW1wLmNvcmUuU3F1ZWFrXzQkc3VtX29mX3Jvd3MpDQoNCm5hbWVzKGNvcmUuU3F1ZWFrLnJlbC5hYnVuZCkgPC0gJ1JlbC5hYnVuZC5vZi5jb3JlJw0KDQpjb3JlLlNxdWVhay5yZWwuYWJ1bmQuMiA8LSBjb3JlLlNxdWVhay5yZWwuYWJ1bmQNCmNvcmUuU3F1ZWFrLnJlbC5hYnVuZC4yJElEIDwtIHJvd25hbWVzKGNvcmUuU3F1ZWFrLnJlbC5hYnVuZC4yKQ0KDQpjb3JlLlNxdWVhay5yZWwuYWJ1bmQuMyA8LSBjb3JlLlNxdWVhay5yZWwuYWJ1bmQuMg0KY29yZS5TcXVlYWsucmVsLmFidW5kLjMkSUQuMiA8LSBjb3JlLlNxdWVhay5yZWwuYWJ1bmQuMyRJRA0KDQpjb3JlLlNxdWVhay5yZWwuYWJ1bmQuNCA9IHNlcGFyYXRlKGNvcmUuU3F1ZWFrLnJlbC5hYnVuZC4zLCBJRCwgYygnSUQnLCdXZWVrJykpDQoNCiMjI1N0YXJidWNrDQpjb3JlLlN0YXJidWNrLnJlbC5hYnVuZCA8LWFzLmRhdGEuZnJhbWUoZGF0LmRvbF93YXRlci5maWwuNHNhbXAuY29yZS5TdGFyYnVja180JHN1bV9vZl9yb3dzKQ0KDQpuYW1lcyhjb3JlLlN0YXJidWNrLnJlbC5hYnVuZCkgPC0gJ1JlbC5hYnVuZC5vZi5jb3JlJw0KDQpjb3JlLlN0YXJidWNrLnJlbC5hYnVuZC4yIDwtIGNvcmUuU3RhcmJ1Y2sucmVsLmFidW5kDQpjb3JlLlN0YXJidWNrLnJlbC5hYnVuZC4yJElEIDwtIHJvd25hbWVzKGNvcmUuU3RhcmJ1Y2sucmVsLmFidW5kLjIpDQoNCmNvcmUuU3RhcmJ1Y2sucmVsLmFidW5kLjMgPC0gY29yZS5TdGFyYnVjay5yZWwuYWJ1bmQuMg0KY29yZS5TdGFyYnVjay5yZWwuYWJ1bmQuMyRJRC4yIDwtIGNvcmUuU3RhcmJ1Y2sucmVsLmFidW5kLjMkSUQNCg0KY29yZS5TdGFyYnVjay5yZWwuYWJ1bmQuNCA9IHNlcGFyYXRlKGNvcmUuU3RhcmJ1Y2sucmVsLmFidW5kLjMsIElELCBjKCdJRCcsJ1dlZWsnKSkNCg0KIyMjSG93aWUNCmNvcmUuSG93aWUucmVsLmFidW5kIDwtYXMuZGF0YS5mcmFtZShkYXQuZG9sX3dhdGVyLmZpbC40c2FtcC5jb3JlLkhvd2llXzQkc3VtX29mX3Jvd3MpDQoNCm5hbWVzKGNvcmUuSG93aWUucmVsLmFidW5kKSA8LSAnUmVsLmFidW5kLm9mLmNvcmUnDQoNCmNvcmUuSG93aWUucmVsLmFidW5kLjIgPC0gY29yZS5Ib3dpZS5yZWwuYWJ1bmQNCmNvcmUuSG93aWUucmVsLmFidW5kLjIkSUQgPC0gcm93bmFtZXMoY29yZS5Ib3dpZS5yZWwuYWJ1bmQuMikNCg0KY29yZS5Ib3dpZS5yZWwuYWJ1bmQuMyA8LSBjb3JlLkhvd2llLnJlbC5hYnVuZC4yDQpjb3JlLkhvd2llLnJlbC5hYnVuZC4zJElELjIgPC0gY29yZS5Ib3dpZS5yZWwuYWJ1bmQuMyRJRA0KDQpjb3JlLkhvd2llLnJlbC5hYnVuZC40ID0gc2VwYXJhdGUoY29yZS5Ib3dpZS5yZWwuYWJ1bmQuMywgSUQsIGMoJ0lEJywnV2VlaycpKQ0KDQojIyNOdWRnZWUNCmNvcmUuTnVkZ2VlLnJlbC5hYnVuZCA8LSBhcy5kYXRhLmZyYW1lKGRhdC5kb2xfd2F0ZXIuZmlsLjRzYW1wLmNvcmUuTnVkZ2VlXzQkc3VtX29mX3Jvd3MpDQoNCm5hbWVzKGNvcmUuTnVkZ2VlLnJlbC5hYnVuZCkgPC0gJ1JlbC5hYnVuZC5vZi5jb3JlJw0KDQpjb3JlLk51ZGdlZS5yZWwuYWJ1bmQuMiA8LSBjb3JlLk51ZGdlZS5yZWwuYWJ1bmQNCmNvcmUuTnVkZ2VlLnJlbC5hYnVuZC4yJElEIDwtIHJvd25hbWVzKGNvcmUuTnVkZ2VlLnJlbC5hYnVuZC4yKQ0KDQpjb3JlLk51ZGdlZS5yZWwuYWJ1bmQuMyA8LSBjb3JlLk51ZGdlZS5yZWwuYWJ1bmQuMg0KY29yZS5OdWRnZWUucmVsLmFidW5kLjMkSUQuMiA8LSBjb3JlLk51ZGdlZS5yZWwuYWJ1bmQuMyRJRA0KDQpjb3JlLk51ZGdlZS5yZWwuYWJ1bmQuNCA9IHNlcGFyYXRlKGNvcmUuTnVkZ2VlLnJlbC5hYnVuZC4zLCBJRCwgYygnSUQnLCdXZWVrJykpDQoNCiMjIyNDb21iaW5lIGRhdGFmcmFtZXMgb2YgaW5kaXZpZHVhbCBkb2xwaGlucyB0byBvbmUgZGF0YWZyYW1lDQoNClJlbC5hYnVuZC5jb3JlIDwtIHJiaW5kKGNvcmUuQ29lbi5yZWwuYWJ1bmQuNCxjb3JlLkV2aWUucmVsLmFidW5kLjQsIGNvcmUuS2lhbWEucmVsLmFidW5kLjQsY29yZS5Nb2tpLnJlbC5hYnVuZC40LGNvcmUuUkIucmVsLmFidW5kLjQsIGNvcmUuU2Nvb3Rlci5yZWwuYWJ1bmQuNCxjb3JlLlNpcml1cy5yZWwuYWJ1bmQuNCxjb3JlLlNxdWVhay5yZWwuYWJ1bmQuNCwgY29yZS5TdGFyYnVjay5yZWwuYWJ1bmQuNCxjb3JlLkhvd2llLnJlbC5hYnVuZC40LGNvcmUuTnVkZ2VlLnJlbC5hYnVuZC40LA0KY29yZS5SQi5yZWwuYWJ1bmQuNCkNCg0KbmFtZXMoUmVsLmFidW5kLmNvcmUpWzFdID0gJ1JlbGF0aXZlLmFidW5kYW5jZS5vZi5jb3JlLnpPVFVzJw0KDQpSZWwuYWJ1bmQuY29yZSRXZWVrIDwtIGFzLmludGVnZXIoUmVsLmFidW5kLmNvcmUkV2VlaykNCg0KIyMjT3JkZXIgYnkgcmVsLmFidW5kLiBvZiBpbnRyYS1jb3JlDQpSZWwuYWJ1bmQuY29yZS4zIDwtIGFycmFuZ2UoUmVsLmFidW5kLmNvcmUsUmVsYXRpdmUuYWJ1bmRhbmNlLm9mLmNvcmUuek9UVXMpDQptZWFuKFJlbC5hYnVuZC5jb3JlLjMkUmVsYXRpdmUuYWJ1bmRhbmNlLm9mLmNvcmUuek9UVXMpDQojMC43MzQ5MjMxDQoNCk92ZXJ2aWV3LmludHJhX2NvcmUgPC0gUmVsLmFidW5kLmNvcmUuMyAlPiUgZ3JvdXBfYnkoSUQpICU+JSANCiAgc3VtbWFyaXplKG1pbihSZWxhdGl2ZS5hYnVuZGFuY2Uub2YuY29yZS56T1RVcyksDQogICAgICAgICAgICBtYXgoUmVsYXRpdmUuYWJ1bmRhbmNlLm9mLmNvcmUuek9UVXMpLCANCiAgICAgICAgICAgIG1lYW4oUmVsYXRpdmUuYWJ1bmRhbmNlLm9mLmNvcmUuek9UVXMpLA0KICAgICAgICAgICAgc2QoUmVsYXRpdmUuYWJ1bmRhbmNlLm9mLmNvcmUuek9UVXMpKQ0KDQpPdmVydmlldy5pbnRyYV9jb3JlLjIgPC0gUmVsLmFidW5kLmNvcmUuMyAlPiUgZ3JvdXBfYnkoSUQpICU+JSANCiAgICAgICAgICAgICAgICAgICAgICAgICAgICAgICBzdW1tYXJpemUobWVhbihSZWxhdGl2ZS5hYnVuZGFuY2Uub2YuY29yZS56T1RVcykpDQoNCm5hbWVzKE92ZXJ2aWV3LmludHJhX2NvcmUuMikgPC0gYygnSUQnLCAnSW50cmFfY29yZV9tZWFuJykNCg0KT3ZlcnZpZXcuaW50cmFfY29yZS4yIDwtIGFycmFuZ2UoT3ZlcnZpZXcuaW50cmFfY29yZS4yLCBJbnRyYV9jb3JlX21lYW4pDQoNCiNBZGQgY29sdW1uIHdoZXJlIEhvd2llIGFuZCBOdWRnZWUgYXJlIG1hcmtlZCB3aXRoIGFzdGVyaXNrIChhcyB0aGVzZSBkb2xwaGlucyByZWNlaXZlZCBhbnRpYmlvdGljcykNCg0KUmVsLmFidW5kLmNvcmVbNDEsMl0gPSAnTnVkZ2VlKicNClJlbC5hYnVuZC5jb3JlWzQyLDJdID0gJ051ZGdlZSonDQpSZWwuYWJ1bmQuY29yZVs0MywyXSA9ICdOdWRnZWUqJw0KUmVsLmFidW5kLmNvcmVbNDQsMl0gPSAnTnVkZ2VlKicNCg0KUmVsLmFidW5kLmNvcmVbMzcsMl0gPSAnSG93aWUqJw0KUmVsLmFidW5kLmNvcmVbMzgsMl0gPSAnSG93aWUqJw0KUmVsLmFidW5kLmNvcmVbMzksMl0gPSAnSG93aWUqJw0KUmVsLmFidW5kLmNvcmVbNDAsMl0gPSAnSG93aWUqJw0KDQpQbG90LmludHJhX2NvcmUucmVsLmFidW5kLm92ZXIudGltZSA9IA0KICANCiAgZ2dwbG90KGRhdGEgPSBSZWwuYWJ1bmQuY29yZSwgYWVzKHggPSBXZWVrLCB5ID0gUmVsYXRpdmUuYWJ1bmRhbmNlLm9mLmNvcmUuek9UVXMsIGdyb3VwPSBJRCxjb2xvciA9IElEKSkgKyANCiAgZ2VvbV9saW5lKGxpbmV0eXBlID0gImRhc2hlZCIpKw0KICB0aGVtZV9idygpKw0KICBnZW9tX3BvaW50KHNpemUgPSAyKSArIA0KICBzY2FsZV9jb2xvcl9tYW51YWwodmFsdWVzID0gYygncGluaycsJ3llbGxvdycsJ2RhcmsgZ3JlZW4nLCdvcmFuZ2UnLCdkYXJrIGdyZXknLA0KICAgICAgICAgICAgICAgICAgICAgICAgICAgICAgICAnZGFyayByZWQnLCdwdXJwbGUnLCdibGFjaycsJ2RhcmtzYWxtb24nLA0KICAgICAgICAgICAgICAgICAgICAgICAgICAgICAgICAndHVycXVvaXNlJywnZGFyayBibHVlJykpDQoNCmdnc2F2ZSgiUGxvdC5pbnRyYV9jb3JlLnJlbC5hYnVuZC5vdmVyLnRpbWUuanBnIiwgcGxvdCA9IFBsb3QuaW50cmFfY29yZS5yZWwuYWJ1bmQub3Zlci50aW1lICwgZGV2aWNlID0gJ2pwZycsIHdpZHRoID0gMTY4LCBoZWlnaHQgPSAxMzAsIHVuaXRzID0gIm1tIiwNCiAgICAgICBkcGkgPSAzMDAsIGxpbWl0c2l6ZSA9IFRSVUUpDQoNCiMjIyMjVHVybiBjb2wgJ1JlbGF0aXZlLmFidW5kYW5jZS5vZi5jb3JlLnpPVFVzJyBpbnRvICdSZWxhdGl2ZSBhYnVuZGFuY2Ugb2YgY29yZSAjek9UVXMgaW4gJScNCg0KUmVsLmFidW5kLmNvcmUuMiA8LSBSZWwuYWJ1bmQuY29yZQ0KDQpSZWwuYWJ1bmQuY29yZS4yJFJlbGF0aXZlLmFidW5kYW5jZS5vZi5jb3JlLnpPVFVzIDwtIDEwMCpSZWwuYWJ1bmQuY29yZS4yJFJlbGF0aXZlLmFidW5kYW5jZS5vZi5jb3JlLnpPVFVzDQoNClBsb3QucmVsLmFidW5kLm92ZXIudGltZS4yIDwtIA0KICANCiAgZ2dwbG90KGRhdGEgPSBSZWwuYWJ1bmQuY29yZS4yLCBhZXMoeCA9IFdlZWssIHkgPSBSZWxhdGl2ZS5hYnVuZGFuY2Uub2YuY29yZS56T1RVcywgZ3JvdXA9IElELGNvbG9yID0gSUQpKSArIA0KICBnZW9tX2xpbmUobGluZXR5cGUgPSAiZGFzaGVkIikrDQogIHRoZW1lX2J3KCkrDQogIGdlb21fcG9pbnQoc2l6ZSA9IDIpICsgDQogIGxhYnMoeSA9ICdSZWxhdGl2ZSBhYnVuZGFuY2Ugb2YgaW50cmEtY29yZSB6T1RVcyBpbiAlJywgeCA9ICdXZWVrcycpICsNCiAgc2NhbGVfY29sb3JfbWFudWFsKHZhbHVlcyA9IGMoJ3BpbmsnLCd5ZWxsb3cnLCdkYXJrIGdyZWVuJywnb3JhbmdlJywnZGFyayBncmV5JywNCiAgICAgICAgICAgICAgICAgICAgICAgICAgICAgICAgJ2RhcmsgcmVkJywncHVycGxlJywnYmxhY2snLCdkYXJrc2FsbW9uJywNCiAgICAgICAgICAgICAgICAgICAgICAgICAgICAgICAgJ3R1cnF1b2lzZScsJ2RhcmsgYmx1ZScpKQ0KDQpnZ3NhdmUoIlBsb3QucmVsLmFidW5kLm92ZXIudGltZS4yLmpwZyIsIHBsb3QgPSBQbG90LnJlbC5hYnVuZC5vdmVyLnRpbWUuMiAsIGRldmljZSA9ICdqcGcnLCB3aWR0aCA9IDIxMCwgaGVpZ2h0ID0gMTMwLCB1bml0cyA9ICJtbSIsDQogICAgICAgZHBpID0gMzAwLCBsaW1pdHNpemUgPSBUUlVFKQ0KDQpgYGANCg0KIyMjIyMjIyMjIyMjIyMjIyMjIyMjIyMjIyMjIyMjIyMjIyMjIyMjIyMjIyMjIyMjIw0KIyMjQ3JlYXRlIGZyZXF1ZW5jeSBoaXN0b2dyYW0gb2YgaW50cmEtY29yZQ0KYGBge3J9DQpDb3JlLnpPVFUuQ29lbi40c2FtcF8xLjBfMiA8LSBDb3JlLnpPVFUuQ29lbi40c2FtcF8xLjBbLC1jKDEsMyw1OjcpXQ0KQ29yZS56T1RVLkV2aWUuNHNhbXBfMS4wXzIgPC0gQ29yZS56T1RVLkV2aWUuNHNhbXBfMS4wWywtYygxLDMsNTo3KV0NCkNvcmUuek9UVS5Nb2tpLjRzYW1wXzEuMF8yIDwtIENvcmUuek9UVS5Nb2tpLjRzYW1wXzEuMFssLWMoMSwzLDU6NyldDQpDb3JlLnpPVFUuS2lhbWEuNHNhbXBfMS4wXzIgPC0gQ29yZS56T1RVLktpYW1hLjRzYW1wXzEuMFssLWMoMSwzLDU6NyldDQpDb3JlLnpPVFUuUkIuNHNhbXBfMS4wXzIgPC0gQ29yZS56T1RVLlJCLjRzYW1wXzEuMFssLWMoMSwzLDU6NyldDQpDb3JlLnpPVFUuU2Nvb3Rlci40c2FtcF8xLjBfMiA8LSBDb3JlLnpPVFUuU2Nvb3Rlci40c2FtcF8xLjBbLC1jKDEsMyw1OjcpXQ0KQ29yZS56T1RVLlNpcml1cy40c2FtcF8xLjBfMiA8LSBDb3JlLnpPVFUuU2lyaXVzLjRzYW1wXzEuMFssLWMoMSwzLDU6NyldDQpDb3JlLnpPVFUuU3F1ZWFrLjRzYW1wXzEuMF8yIDwtIENvcmUuek9UVS5TcXVlYWsuNHNhbXBfMS4wWywtYygxLDMsNTo3KV0NCkNvcmUuek9UVS5TdGFyYnVjay40c2FtcF8xLjBfMiA8LSBDb3JlLnpPVFUuU3RhcmJ1Y2suNHNhbXBfMS4wWywtYygxLDMsNTo3KV0NCkNvcmUuek9UVS5Ib3dpZS40c2FtcF8xLjBfMiA8LSBDb3JlLnpPVFUuSG93aWUuNHNhbXBfMS4wWywtYygxLDMsNTo3KV0NCkNvcmUuek9UVS5OdWRnZWUuNHNhbXBfMS4wXzIgPC0gQ29yZS56T1RVLk51ZGdlZS40c2FtcF8xLjBbLC1jKDEsMyw1OjcpXQ0KDQoNCkNvcmUuek9UVS5hbGwuZG9sIDwtIGZ1bGxfam9pbiAoYnkgPSBjKCd2YXJpYWJsZScpLCBDb3JlLnpPVFUuQ29lbi40c2FtcF8xLjBfMiwgQ29yZS56T1RVLkV2aWUuNHNhbXBfMS4wXzIpDQpDb3JlLnpPVFUuYWxsLmRvbC4yIDwtIGZ1bGxfam9pbiAoYnkgPSBjKCd2YXJpYWJsZScpLCBDb3JlLnpPVFUuYWxsLmRvbCwgQ29yZS56T1RVLk1va2kuNHNhbXBfMS4wXzIpDQpDb3JlLnpPVFUuYWxsLmRvbC4zIDwtIGZ1bGxfam9pbiAoYnkgPSBjKCd2YXJpYWJsZScpLCBDb3JlLnpPVFUuYWxsLmRvbC4yLCBDb3JlLnpPVFUuS2lhbWEuNHNhbXBfMS4wXzIpDQpDb3JlLnpPVFUuYWxsLmRvbC40IDwtIGZ1bGxfam9pbiAoYnkgPSBjKCd2YXJpYWJsZScpLCBDb3JlLnpPVFUuYWxsLmRvbC4zLCBDb3JlLnpPVFUuU2Nvb3Rlci40c2FtcF8xLjBfMikNCkNvcmUuek9UVS5hbGwuZG9sLjUgPC0gZnVsbF9qb2luIChieSA9IGMoJ3ZhcmlhYmxlJyksIENvcmUuek9UVS5hbGwuZG9sLjQsIENvcmUuek9UVS5SQi40c2FtcF8xLjBfMikNCkNvcmUuek9UVS5hbGwuZG9sLjYgPC0gZnVsbF9qb2luIChieSA9IGMoJ3ZhcmlhYmxlJyksIENvcmUuek9UVS5hbGwuZG9sLjUsIENvcmUuek9UVS5TaXJpdXMuNHNhbXBfMS4wXzIpDQpDb3JlLnpPVFUuYWxsLmRvbC43IDwtIGZ1bGxfam9pbiAoYnkgPSBjKCd2YXJpYWJsZScpLCBDb3JlLnpPVFUuYWxsLmRvbC42LCBDb3JlLnpPVFUuU3F1ZWFrLjRzYW1wXzEuMF8yKQ0KQ29yZS56T1RVLmFsbC5kb2wuOCA8LSBmdWxsX2pvaW4gKGJ5ID0gYygndmFyaWFibGUnKSwgQ29yZS56T1RVLmFsbC5kb2wuNywgQ29yZS56T1RVLlN0YXJidWNrLjRzYW1wXzEuMF8yKQ0KQ29yZS56T1RVLmFsbC5kb2wuOSA8LSBmdWxsX2pvaW4gKGJ5ID0gYygndmFyaWFibGUnKSwgQ29yZS56T1RVLmFsbC5kb2wuOCwgQ29yZS56T1RVLkhvd2llLjRzYW1wXzEuMF8yKQ0KQ29yZS56T1RVLmFsbC5kb2wuMTAgPC0gZnVsbF9qb2luIChieSA9IGMoJ3ZhcmlhYmxlJyksIENvcmUuek9UVS5hbGwuZG9sLjksIENvcmUuek9UVS5OdWRnZWUuNHNhbXBfMS4wXzIpDQoNCmRpbShDb3JlLnpPVFUuYWxsLmRvbC4xMCkNCg0KQ29yZS56T1RVLmFsbC5kb2wuMTEgPC0gQ29yZS56T1RVLmFsbC5kb2wuMTAgJT4lIG11dGF0ZSAoc3VtX29mX3Jvd3M9cm93U3VtcyhDb3JlLnpPVFUuYWxsLmRvbC4xMFssMjoxMl0sbmEucm09VFJVRSkpICU+JSBhcnJhbmdlKHN1bV9vZl9yb3dzKQ0KI1ZpZXcoQ29yZS56T1RVLmFsbC5kb2wuMTEpDQoNCkludHJhLmNvcmUuaGlzdG8gPC0gYXMuZGF0YS5mcmFtZShDb3JlLnpPVFUuYWxsLmRvbC4xMSRzdW1fb2Zfcm93cykNCm5hbWVzKEludHJhLmNvcmUuaGlzdG8pIDwtICdJbnRyYS5jb3JlLmhpc3RvJw0KDQojIyNEcmF3IGhpc3RvZ3JhbSB3aXRoIENvcmUuek9UVS5hbGwuZG9sLjExJHN1bV9vZl9yb3dzDQpJbnRyYS5jb3JlLmhpc3RvZ3JhbSA9DQogIGdncGxvdChJbnRyYS5jb3JlLmhpc3RvLCBhZXMoeCA9IEludHJhLmNvcmUuaGlzdG8pKSArDQogIGdlb21faGlzdG9ncmFtKGJpbndpZHRoID0gMC41KSsNCiAgdGhlbWVfYncoKSArDQogIHRoZW1lKHBsb3QudGl0bGUgPSBlbGVtZW50X3RleHQoc2l6ZSA9IDE0LCBmYW1pbHkgPSAiVGFob21hIiwgZmFjZSA9ICJib2xkIiksDQogICAgICAgIHRleHQgPSBlbGVtZW50X3RleHQoc2l6ZSA9IDEyLCBmYW1pbHkgPSAiVGFob21hIiksDQogICAgICAgIGF4aXMudGl0bGUgPSBlbGVtZW50X3RleHQoKSwNCiAgICAgICAgYXhpcy50ZXh0Lng9ZWxlbWVudF90ZXh0KHNpemUgPSAxMSkpICsNCiAgc2NhbGVfeF9jb250aW51b3VzKG5hbWUgPSAiTnVtYmVyIG9mIGRvbHBoaW5zIHRoYXQgaGFyYm91ciBjb3JlIHpPVFVzIiwgDQogICAgICAgICAgICAgICAgICAgICBicmVha3MgPSBzZXEoMToxMSkpICsNCiAgc2NhbGVfeV9jb250aW51b3VzKG5hbWUgPSAiTnVtYmVyIG9mIGludHJhLWNvcmUgek9UVXMiKQ0KDQpnZ3NhdmUoIkludHJhLmNvcmUuaGlzdG9ncmFtLmpwZyIsIHBsb3QgPSBJbnRyYS5jb3JlLmhpc3RvZ3JhbSAsIGRldmljZSA9ICdqcGcnLCB3aWR0aCA9IDE2OCwgaGVpZ2h0ID0gMTMwLCB1bml0cyA9ICJtbSIsDQogICAgICAgZHBpID0gMzAwLCBsaW1pdHNpemUgPSBUUlVFKQ0KYGBgDQoNCiMjIyMjIyMjIyMjIyMjIyMjIyMjIyMjIyMjIyMjIyMjIyMjIyMjIyMjIyMjIw0KIyMjIyMjI0RldGVybWluZSBpbnRlci1jb3JlIChiYWN0ZXJpYSBhbGwgZG9scGhpbnMgc2hhcmUgYXQgYW55IHBvaW50IGluIHRpbWUpDQpgYGB7cn0NCiMjI1VzZSBkYXQuZG9sX3dhdGVyLmZpbC50LjINCmRpbShkYXQuZG9sX3dhdGVyLmZpbC50LjIpDQojODMgMTQ3MQ0KZGF0LmRvbF93YXRlci5maWwudC4xNSA8LSBkYXQuZG9sX3dhdGVyLmZpbC50LjINCmRhdC5kb2xfd2F0ZXIuZmlsLnQuMTUkRG9scGhpbiA8LSByb3duYW1lcyhkYXQuZG9sX3dhdGVyLmZpbC50LjE1KQ0KZGF0LmRvbF93YXRlci5maWwudC4xNiA8LSBkYXQuZG9sX3dhdGVyLmZpbC50LjE1DQpkYXQuZG9sX3dhdGVyLmZpbC50LjE2JERvbHBoaW4uMiA8LSBkYXQuZG9sX3dhdGVyLmZpbC50LjE2JERvbHBoaW4NCg0KZGltKGRhdC5kb2xfd2F0ZXIuZmlsLnQuMTYpDQojODMgMTQ3Mw0KDQojIyNSZS1vcmRlciwgYnJpbmcgY29scyBEb2xwaGluICYgRG9scGhpbi4yIHRvIGZyb250DQpkYXQuZG9sX3dhdGVyLmZpbC50LjE3ID0gZGF0LmRvbF93YXRlci5maWwudC4xNlssYygxNDcyLDE0NzMsMToxNDcxKV0NCg0KZGF0LmRvbF93YXRlci5maWwudC4xOCA9IHNlcGFyYXRlKGRhdC5kb2xfd2F0ZXIuZmlsLnQuMTcsIERvbHBoaW4uMiwgYygnSUQnLCd0aW1lJykpDQoNCmRhdC5kb2xfd2F0ZXIuZmlsLnQuMTkgPSBkYXQuZG9sX3dhdGVyLmZpbC50LjE4W29yZGVyKGRhdC5kb2xfd2F0ZXIuZmlsLnQuMTgkdGltZSksXQ0KYGBgDQojIyMjIyMjIyMjIyMjIyMjIyMjIyMNCiMjI1NlcGFyYXRlIGludGVyLWNvcmUgYnkgd2Vlaw0KIyMjIyMjIyMjIyMjIyMjIyMjIyMjDQoNCiMjIyMjIyMjIyMjIyMjDQojIyNXZWVrMg0KYGBge3J9DQojIyMjIyMjIyMjIyMjIyMjIyMjIyMNCiMjMiAoMTAgZG9scGhpbnMpDQpkYXQuZG9sX3dhdGVyLmZpbC53ZWVrLjIgPC0gDQogIGRhdC5kb2xfd2F0ZXIuZmlsLnQuMTlbYygzMTo0MCksXQ0KDQojVmlldyhkYXQuZG9sX3dhdGVyLmZpbC53ZWVrLjJbLDE6MTBdKQ0KZGltKGRhdC5kb2xfd2F0ZXIuZmlsLndlZWsuMikNCiMxMCAxNDc0DQoNCndyaXRlLmNzdihkYXQuZG9sX3dhdGVyLmZpbC53ZWVrLjIsICdkYXQuZG9sX3dhdGVyLmZpbC53ZWVrLjIuY3N2JykNCg0KIyMjUmVsYXRpdmUgYWJ1bmRhbmNlDQpkYXQuZG9sX3dhdGVyLmZpbC53ZWVrLjIuMiA8LSBkYXQuZG9sX3dhdGVyLmZpbC53ZWVrLjJbLC1jKDEsMyldDQpkaW0oZGF0LmRvbF93YXRlci5maWwud2Vlay4yLjIpDQojMTAgMTQ3Mg0KDQpkYXQuZG9sX3dhdGVyLmZpbC53ZWVrLjJfcmVsLmFidW5kIDwtIGRhdC5kb2xfd2F0ZXIuZmlsLndlZWsuMi4yWywyOjE0NzJdIC9yb3dTdW1zKGRhdC5kb2xfd2F0ZXIuZmlsLndlZWsuMi4yWywyOjE0NzJdKQ0KDQojVHJhbnNwb3NlDQpkYXQuZG9sX3dhdGVyLmZpbC53ZWVrLjJfcmVsLmFidW5kX3QgPC0gYXMuZGF0YS5mcmFtZSh0KGRhdC5kb2xfd2F0ZXIuZmlsLndlZWsuMl9yZWwuYWJ1bmQpKQ0KDQpkYXQuZG9sX3dhdGVyLmZpbC53ZWVrLjJfcmVsLmFidW5kX3QkdmFyaWFibGUgPC0gcm93bmFtZXMoZGF0LmRvbF93YXRlci5maWwud2Vlay4yX3JlbC5hYnVuZF90KQ0KZGltKGRhdC5kb2xfd2F0ZXIuZmlsLndlZWsuMl9yZWwuYWJ1bmRfdCkNCiMxNDcxICAgMTENCg0KZGF0LmRvbF93YXRlci5maWwud2Vlay4yX3JlbC5hYnVuZF90LjIgPC0gZGF0LmRvbF93YXRlci5maWwud2Vlay4yX3JlbC5hYnVuZF90ICU+JSBtdXRhdGUobWVhbl9vZl9yb3dzID0gcm93TWVhbnMoZGF0LmRvbF93YXRlci5maWwud2Vlay4yX3JlbC5hYnVuZF90WywxOjEwXSkpDQoNClJlbC5hYnVuZF93ZWVrLjIgPC0gZGF0LmRvbF93YXRlci5maWwud2Vlay4yX3JlbC5hYnVuZF90LjJbLDExOjEyXQ0KDQojQ29udmVydCBkYXRhZnJhbWUgaW50byBiaW5hcnkNCmRhdC5kb2xfd2F0ZXIuZmlsLndlZWsuMi5iaW5hcnkgPC0gZGF0LmRvbF93YXRlci5maWwud2Vlay4yWyw0OjE0NzFdDQoNCmRhdC5kb2xfd2F0ZXIuZmlsLndlZWsuMi5iaW5hcnkgW10gIDwtICArKGRhdC5kb2xfd2F0ZXIuZmlsLndlZWsuMi5iaW5hcnkgID4gMCkNCg0KI1ZpZXcoZGF0LmRvbF93YXRlci5maWwud2Vlay4yLmJpbmFyeSBbLDE6MTBdKQ0KDQojVHJhbnNwb3NlIGRhdC5kb2xfd2F0ZXIuZmlsLndlZWsuMi5iaW5hcnkNCmRhdC5kb2xfd2F0ZXIuZmlsLndlZWsuMi5iaW5hcnlfdCA8LSBhcy5kYXRhLmZyYW1lKHQoZGF0LmRvbF93YXRlci5maWwud2Vlay4yLmJpbmFyeSkpDQojVmlldyhkYXQuZG9sX3dhdGVyLmZpbC53ZWVrLjIuYmluYXJ5WzE6MTAsXSkNCg0KI0NyZWF0ZSBjb2wgVG90YWwucmVsLmFidW5kLg0KZGF0LmRvbF93YXRlci5maWwud2Vlay4yLmJpbmFyeV90LjIgPC0gZGF0LmRvbF93YXRlci5maWwud2Vlay4yLmJpbmFyeV90ICU+JSANCiAgbXV0YXRlIChUb3RhbC5yZWwuYWJ1bmQuek9UVSA9IHJvd1N1bXMoZGF0LmRvbF93YXRlci5maWwud2Vlay4yLmJpbmFyeV90KS8xMCkNCg0KI1ZpZXcoZGF0LmRvbF93YXRlci5maWwud2Vlay4yLmJpbmFyeV90LjJbMToxMCxdKQ0KDQojR2l2ZSBkYXQuZG9sX3dhdGVyLmZpbC53ZWVrLjIuYmluYXJ5X3QuMiByb3duYW1lcyBhcyBjb2wgdmFyaWFibGUNCmRhdC5kb2xfd2F0ZXIuZmlsLndlZWsuMi5iaW5hcnlfdC4yJHZhcmlhYmxlIDwtIHJvd25hbWVzKGRhdC5kb2xfd2F0ZXIuZmlsLndlZWsuMi5iaW5hcnlfdCkNCmRpbShkYXQuZG9sX3dhdGVyLmZpbC53ZWVrLjIuYmluYXJ5X3QuMikNCiMxNDY4IDEyDQoNCiNSZW9yZGVyDQpkYXQuZG9sX3dhdGVyLmZpbC53ZWVrLjIuYmluYXJ5X3QuMyA8LSBkYXQuZG9sX3dhdGVyLmZpbC53ZWVrLjIuYmluYXJ5X3QuMlssYygxMiwxMSwxOjEwKV0NCg0KI0pvaW4gZGF0LmRvbF93YXRlci5maWwud2Vlay4yLmJpbmFyeV90LjMgd2l0aCBhdHRyaWJ1dGVzLmRvbF9yZHBfd2F0ZXIuZmlsLjINCkludGVyLmNvcmUuek9UVS53ZWVrLjIgPC0gZGF0LmRvbF93YXRlci5maWwud2Vlay4yLmJpbmFyeV90LjMgJT4lIGxlZnRfam9pbihhdHRyaWJ1dGVzLmRvbF9yZHBfd2F0ZXIuZmlsLjIpDQpkaW0oSW50ZXIuY29yZS56T1RVLndlZWsuMikNCiMxNDY4ICAgMTgNCiNWaWV3KEludGVyLmNvcmUuek9UVS53ZWVrLjJbMToxMCxdKQ0KDQojUmVvcmRlcg0KSW50ZXIuY29yZS56T1RVLndlZWsuMl8yIDwtIEludGVyLmNvcmUuek9UVS53ZWVrLjJbLGMoMSwyLDE3LDM6MTIpXQ0KZGltKEludGVyLmNvcmUuek9UVS53ZWVrLjJfMikNCiMjMTg4NSAxMw0KDQpuYW1lcyhJbnRlci5jb3JlLnpPVFUud2Vlay4yXzIpDQoNCiNPcmRlciBhY2NvcmRpbmcgdG8gSW50ZXIuY29yZS56T1RVLndlZWsuMl8yJFRvdGFsLnJlbC5hYnVuZC56T1RVDQpJbnRlci5jb3JlLnpPVFUud2Vlay4yXzMgPC0gSW50ZXIuY29yZS56T1RVLndlZWsuMl8yW29yZGVyKEludGVyLmNvcmUuek9UVS53ZWVrLjJfMiRUb3RhbC5yZWwuYWJ1bmQuek9UVSksXQ0KI1ZpZXcoSW50ZXIuY29yZS56T1RVLndlZWsuMl8zWywxOjJdKQ0KDQojQW55IGludGVyLmNvcmUuT1RVcyBhYm92ZSAxMDAlICg9PSAxLjApPw0KSW50ZXIuY29yZS56T1RVLndlZWsuMl8xLjAgPC0gSW50ZXIuY29yZS56T1RVLndlZWsuMl8zICU+JSBmaWx0ZXIgKFRvdGFsLnJlbC5hYnVuZC56T1RVID09IDEuMCkNCmRpbShJbnRlci5jb3JlLnpPVFUud2Vlay4yXzEuMCkNCiMzMg0KDQojRGV0ZXJtaW5lIHJlbGF0aXZlIGFidW5kYW5jZSBvZiB0aG9zZSBjb3JlIHpPVFVzDQpJbnRlci5jb3JlX3dlZWsuMiA8LSBJbnRlci5jb3JlLnpPVFUud2Vlay4yXzEuMCR2YXJpYWJsZQ0KDQpSZWwuYWJ1bmRfd2Vlay4yLjIgPC0gUmVsLmFidW5kX3dlZWsuMg0KDQpyb3duYW1lcyhSZWwuYWJ1bmRfd2Vlay4yLjIpIDwtIFJlbC5hYnVuZF93ZWVrLjIuMiR2YXJpYWJsZQ0KDQpSZWwuYWJ1bmRfd2Vlay4yLkNvcmUgPC0gUmVsLmFidW5kX3dlZWsuMi4yW0ludGVyLmNvcmVfd2Vlay4yLF0NCg0Kc3VtKFJlbC5hYnVuZF93ZWVrLjIuQ29yZVssMl0pDQojMC4yNzI5MDM0DQoNCndyaXRlLmNzdihSZWwuYWJ1bmRfd2Vlay4yLkNvcmUsICdSZWwuYWJ1bmRfd2Vlay4yLkNvcmUuY3N2JykNCmBgYA0KDQojIyMjIyMjIyMjIyMjIyMjIyMjIyMNCiMjV2VlayA2ICgxMCBkb2xwaGlucykNCmBgYHtyfQ0KIyMjIyMjIyMjIyMjIyMjIyMjIyMjDQojIyAoMTAgZG9scGhpbnMpDQpkYXQuZG9sX3dhdGVyLmZpbC53ZWVrLjYgPC0gDQogIGRhdC5kb2xfd2F0ZXIuZmlsLnQuMTlbYyg3MTo4MCksXQ0KDQojVmlldyhkYXQuZG9sX3dhdGVyLmZpbC53ZWVrLjZbLDE6MTBdKQ0KZGltKGRhdC5kb2xfd2F0ZXIuZmlsLndlZWsuNikNCiMxMCAxNDc0DQoNCndyaXRlLmNzdihkYXQuZG9sX3dhdGVyLmZpbC53ZWVrLjYsICdkYXQuZG9sX3dhdGVyLmZpbC53ZWVrLjYuY3N2JykNCg0KIyMjUmVsYXRpdmUgYWJ1bmRhbmNlDQpkYXQuZG9sX3dhdGVyLmZpbC53ZWVrLjYuMiA8LSBkYXQuZG9sX3dhdGVyLmZpbC53ZWVrLjZbLC1jKDEsMyldDQpkaW0oZGF0LmRvbF93YXRlci5maWwud2Vlay42LjIpDQojMTAgMTQ3Mg0KDQpkYXQuZG9sX3dhdGVyLmZpbC53ZWVrLjZfcmVsLmFidW5kIDwtIGRhdC5kb2xfd2F0ZXIuZmlsLndlZWsuNi4yWywyOjE0NzJdIC9yb3dTdW1zKGRhdC5kb2xfd2F0ZXIuZmlsLndlZWsuNi4yWywyOjE0NzJdKQ0KDQojVHJhbnNwb3NlDQpkYXQuZG9sX3dhdGVyLmZpbC53ZWVrLjZfcmVsLmFidW5kX3QgPC0gYXMuZGF0YS5mcmFtZSh0KGRhdC5kb2xfd2F0ZXIuZmlsLndlZWsuNl9yZWwuYWJ1bmQpKQ0KDQpkYXQuZG9sX3dhdGVyLmZpbC53ZWVrLjZfcmVsLmFidW5kX3QkdmFyaWFibGUgPC0gcm93bmFtZXMoZGF0LmRvbF93YXRlci5maWwud2Vlay42X3JlbC5hYnVuZF90KQ0KZGltKGRhdC5kb2xfd2F0ZXIuZmlsLndlZWsuNl9yZWwuYWJ1bmRfdCkNCiMxNDcxICAgMTENCg0KZGF0LmRvbF93YXRlci5maWwud2Vlay42X3JlbC5hYnVuZF90LjIgPC0gZGF0LmRvbF93YXRlci5maWwud2Vlay42X3JlbC5hYnVuZF90ICU+JSBtdXRhdGUobWVhbl9vZl9yb3dzID0gcm93TWVhbnMoZGF0LmRvbF93YXRlci5maWwud2Vlay42X3JlbC5hYnVuZF90WywxOjEwXSkpDQoNClJlbC5hYnVuZF93ZWVrLjYgPC0gZGF0LmRvbF93YXRlci5maWwud2Vlay42X3JlbC5hYnVuZF90LjJbLDExOjEyXQ0KDQojQ29udmVydCBkYXRhZnJhbWUgaW50byBiaW5hcnkNCmRhdC5kb2xfd2F0ZXIuZmlsLndlZWsuNi5iaW5hcnkgPC0gZGF0LmRvbF93YXRlci5maWwud2Vlay42Wyw0OjE0NzFdDQoNCmRhdC5kb2xfd2F0ZXIuZmlsLndlZWsuNi5iaW5hcnkgW10gIDwtICArKGRhdC5kb2xfd2F0ZXIuZmlsLndlZWsuNi5iaW5hcnkgID4gMCkNCg0KI1ZpZXcoZGF0LmRvbF93YXRlci5maWwud2Vlay42LmJpbmFyeSBbLDE6MTBdKQ0KDQojVHJhbnNwb3NlIGRhdC5kb2xfd2F0ZXIuZmlsLndlZWsuNi5iaW5hcnkNCmRhdC5kb2xfd2F0ZXIuZmlsLndlZWsuNi5iaW5hcnlfdCA8LSBhcy5kYXRhLmZyYW1lKHQoZGF0LmRvbF93YXRlci5maWwud2Vlay42LmJpbmFyeSkpDQojVmlldyhkYXQuZG9sX3dhdGVyLmZpbC53ZWVrLjYuYmluYXJ5WzE6MTAsXSkNCg0KI0NyZWF0ZSBjb2wgVG90YWwucmVsLmFidW5kLg0KZGF0LmRvbF93YXRlci5maWwud2Vlay42LmJpbmFyeV90LjIgPC0gZGF0LmRvbF93YXRlci5maWwud2Vlay42LmJpbmFyeV90ICU+JSANCiAgbXV0YXRlIChUb3RhbC5yZWwuYWJ1bmQuek9UVSA9IHJvd1N1bXMoZGF0LmRvbF93YXRlci5maWwud2Vlay42LmJpbmFyeV90KS8xMCkNCg0KI1ZpZXcoZGF0LmRvbF93YXRlci5maWwud2Vlay42LmJpbmFyeV90LjJbMToxMCxdKQ0KDQojR2l2ZSBkYXQuZG9sX3dhdGVyLmZpbC53ZWVrLjYuYmluYXJ5X3QuMiByb3duYW1lcyBhcyBjb2wgdmFyaWFibGUNCmRhdC5kb2xfd2F0ZXIuZmlsLndlZWsuNi5iaW5hcnlfdC4yJHZhcmlhYmxlIDwtIHJvd25hbWVzKGRhdC5kb2xfd2F0ZXIuZmlsLndlZWsuNi5iaW5hcnlfdCkNCmRpbShkYXQuZG9sX3dhdGVyLmZpbC53ZWVrLjYuYmluYXJ5X3QuMikNCiMxNDY4IDEyDQoNCiNSZW9yZGVyDQpkYXQuZG9sX3dhdGVyLmZpbC53ZWVrLjYuYmluYXJ5X3QuMyA8LSBkYXQuZG9sX3dhdGVyLmZpbC53ZWVrLjYuYmluYXJ5X3QuMlssYygxMiwxMSwxOjEwKV0NCg0KI0pvaW4gZGF0LmRvbF93YXRlci5maWwud2Vlay42LmJpbmFyeV90LjMgd2l0aCBhdHRyaWJ1dGVzLmRvbF9yZHBfd2F0ZXIuZmlsLjINCkludGVyLmNvcmUuek9UVS53ZWVrLjYgPC0gZGF0LmRvbF93YXRlci5maWwud2Vlay42LmJpbmFyeV90LjMgJT4lIGxlZnRfam9pbihhdHRyaWJ1dGVzLmRvbF9yZHBfd2F0ZXIuZmlsLjIpDQpkaW0oSW50ZXIuY29yZS56T1RVLndlZWsuNikNCiMxNDY4ICAgMTgNCiNWaWV3KEludGVyLmNvcmUuek9UVS53ZWVrLjZbMToxMCxdKQ0KDQojUmVvcmRlcg0KSW50ZXIuY29yZS56T1RVLndlZWsuNl8yIDwtIEludGVyLmNvcmUuek9UVS53ZWVrLjZbLGMoMSwyLDE3LDM6MTIpXQ0KZGltKEludGVyLmNvcmUuek9UVS53ZWVrLjZfMikNCiMjMTQ2OCAxMw0KDQpuYW1lcyhJbnRlci5jb3JlLnpPVFUud2Vlay42XzIpDQoNCiNPcmRlciBhY2NvcmRpbmcgdG8gSW50ZXIuY29yZS56T1RVLndlZWsuNl8yJFRvdGFsLnJlbC5hYnVuZC56T1RVDQpJbnRlci5jb3JlLnpPVFUud2Vlay42XzMgPC0gSW50ZXIuY29yZS56T1RVLndlZWsuNl8yW29yZGVyKEludGVyLmNvcmUuek9UVS53ZWVrLjZfMiRUb3RhbC5yZWwuYWJ1bmQuek9UVSksXQ0KI1ZpZXcoSW50ZXIuY29yZS56T1RVLndlZWsuNl8zWywxOjJdKQ0KDQojQW55IGludGVyLmNvcmUuT1RVcyBhYm92ZSAxMDAlICg9PSAxLjApPw0KSW50ZXIuY29yZS56T1RVLndlZWsuNl8xLjAgPC0gSW50ZXIuY29yZS56T1RVLndlZWsuNl8zICU+JSBmaWx0ZXIgKFRvdGFsLnJlbC5hYnVuZC56T1RVID09IDEuMCkNCmRpbShJbnRlci5jb3JlLnpPVFUud2Vlay42XzEuMCkNCiM0MA0KDQojRGV0ZXJtaW5lIHJlbGF0aXZlIGFidW5kYW5jZSBvZiB0aG9zZSBjb3JlIHpPVFVzDQpJbnRlci5jb3JlX3dlZWsuNiA8LSBJbnRlci5jb3JlLnpPVFUud2Vlay42XzEuMCR2YXJpYWJsZQ0KDQpSZWwuYWJ1bmRfd2Vlay42LjIgPC0gUmVsLmFidW5kX3dlZWsuNg0KDQpyb3duYW1lcyhSZWwuYWJ1bmRfd2Vlay42LjIpIDwtIFJlbC5hYnVuZF93ZWVrLjYuMiR2YXJpYWJsZQ0KDQpSZWwuYWJ1bmRfd2Vlay42LkNvcmUgPC0gUmVsLmFidW5kX3dlZWsuNi4yW0ludGVyLmNvcmVfd2Vlay42LF0NCg0Kc3VtKFJlbC5hYnVuZF93ZWVrLjYuQ29yZVssMl0pDQojMC4xODU2OTg2DQoNCndyaXRlLmNzdihSZWwuYWJ1bmRfd2Vlay42LkNvcmUsICdSZWwuYWJ1bmRfd2Vlay42LkNvcmUuY3N2JykNCmBgYA0KDQojIyMjIyMjIyMjIyMjIyMjIyMjIyMNCiMjV2VlayAxMSAoMTAgZG9scGhpbnMpDQpgYGB7cn0NCiMjIyMjIyMjIyMjIyMjIyMjIyMjIw0KIyMgKDEwIGRvbHBoaW5zKQ0KZGF0LmRvbF93YXRlci5maWwud2Vlay4xMSA8LSANCiAgZGF0LmRvbF93YXRlci5maWwudC4xOVtjKDE6MTApLF0NCg0KI1ZpZXcoZGF0LmRvbF93YXRlci5maWwud2Vlay4xMVssMToxMF0pDQpkaW0oZGF0LmRvbF93YXRlci5maWwud2Vlay4xMSkNCiMxMCAxNDc0DQoNCndyaXRlLmNzdihkYXQuZG9sX3dhdGVyLmZpbC53ZWVrLjExLCAnZGF0LmRvbF93YXRlci5maWwud2Vlay4xMS5jc3YnKQ0KDQojIyNSZWxhdGl2ZSBhYnVuZGFuY2UNCmRhdC5kb2xfd2F0ZXIuZmlsLndlZWsuMTEuMiA8LSBkYXQuZG9sX3dhdGVyLmZpbC53ZWVrLjExWywtYygxLDMpXQ0KZGltKGRhdC5kb2xfd2F0ZXIuZmlsLndlZWsuMTEuMikNCiMxMCAxNDcyDQoNCmRhdC5kb2xfd2F0ZXIuZmlsLndlZWsuMTFfcmVsLmFidW5kIDwtIGRhdC5kb2xfd2F0ZXIuZmlsLndlZWsuMTEuMlssMjoxNDcyXSAvcm93U3VtcyhkYXQuZG9sX3dhdGVyLmZpbC53ZWVrLjExLjJbLDI6MTQ3Ml0pDQoNCiNUcmFuc3Bvc2UNCmRhdC5kb2xfd2F0ZXIuZmlsLndlZWsuMTFfcmVsLmFidW5kX3QgPC0gYXMuZGF0YS5mcmFtZSh0KGRhdC5kb2xfd2F0ZXIuZmlsLndlZWsuMTFfcmVsLmFidW5kKSkNCg0KZGF0LmRvbF93YXRlci5maWwud2Vlay4xMV9yZWwuYWJ1bmRfdCR2YXJpYWJsZSA8LSByb3duYW1lcyhkYXQuZG9sX3dhdGVyLmZpbC53ZWVrLjExX3JlbC5hYnVuZF90KQ0KZGltKGRhdC5kb2xfd2F0ZXIuZmlsLndlZWsuMTFfcmVsLmFidW5kX3QpDQojMTQ3MSAgIDExDQoNCmRhdC5kb2xfd2F0ZXIuZmlsLndlZWsuMTFfcmVsLmFidW5kX3QuMiA8LSBkYXQuZG9sX3dhdGVyLmZpbC53ZWVrLjExX3JlbC5hYnVuZF90ICU+JSBtdXRhdGUobWVhbl9vZl9yb3dzID0gcm93TWVhbnMoZGF0LmRvbF93YXRlci5maWwud2Vlay4xMV9yZWwuYWJ1bmRfdFssMToxMF0pKQ0KDQpSZWwuYWJ1bmRfd2Vlay4xMSA8LSBkYXQuZG9sX3dhdGVyLmZpbC53ZWVrLjExX3JlbC5hYnVuZF90LjJbLDExOjEyXQ0KDQojQ29udmVydCBkYXRhZnJhbWUgaW50byBiaW5hcnkNCmRhdC5kb2xfd2F0ZXIuZmlsLndlZWsuMTEuYmluYXJ5IDwtIGRhdC5kb2xfd2F0ZXIuZmlsLndlZWsuMTFbLDQ6MTQ3MV0NCg0KZGF0LmRvbF93YXRlci5maWwud2Vlay4xMS5iaW5hcnkgW10gIDwtICArKGRhdC5kb2xfd2F0ZXIuZmlsLndlZWsuMTEuYmluYXJ5ICA+IDApDQoNCiNWaWV3KGRhdC5kb2xfd2F0ZXIuZmlsLndlZWsuMTEuYmluYXJ5IFssMToxMF0pDQoNCiNUcmFuc3Bvc2UgZGF0LmRvbF93YXRlci5maWwud2Vlay4xMS5iaW5hcnkNCmRhdC5kb2xfd2F0ZXIuZmlsLndlZWsuMTEuYmluYXJ5X3QgPC0gYXMuZGF0YS5mcmFtZSh0KGRhdC5kb2xfd2F0ZXIuZmlsLndlZWsuMTEuYmluYXJ5KSkNCiNWaWV3KGRhdC5kb2xfd2F0ZXIuZmlsLndlZWsuMTEuYmluYXJ5WzE6MTAsXSkNCg0KI0NyZWF0ZSBjb2wgVG90YWwucmVsLmFidW5kLg0KZGF0LmRvbF93YXRlci5maWwud2Vlay4xMS5iaW5hcnlfdC4yIDwtIGRhdC5kb2xfd2F0ZXIuZmlsLndlZWsuMTEuYmluYXJ5X3QgJT4lIA0KICBtdXRhdGUgKFRvdGFsLnJlbC5hYnVuZC56T1RVID0gcm93U3VtcyhkYXQuZG9sX3dhdGVyLmZpbC53ZWVrLjExLmJpbmFyeV90KS8xMCkNCg0KI1ZpZXcoZGF0LmRvbF93YXRlci5maWwud2Vlay4xMS5iaW5hcnlfdC4yWzE6MTAsXSkNCg0KI0dpdmUgZGF0LmRvbF93YXRlci5maWwud2Vlay4xMS5iaW5hcnlfdC4yIHJvd25hbWVzIGFzIGNvbCB2YXJpYWJsZQ0KZGF0LmRvbF93YXRlci5maWwud2Vlay4xMS5iaW5hcnlfdC4yJHZhcmlhYmxlIDwtIHJvd25hbWVzKGRhdC5kb2xfd2F0ZXIuZmlsLndlZWsuMTEuYmluYXJ5X3QpDQpkaW0oZGF0LmRvbF93YXRlci5maWwud2Vlay4xMS5iaW5hcnlfdC4yKQ0KIzE0NjggMTINCg0KI1Jlb3JkZXINCmRhdC5kb2xfd2F0ZXIuZmlsLndlZWsuMTEuYmluYXJ5X3QuMyA8LSBkYXQuZG9sX3dhdGVyLmZpbC53ZWVrLjExLmJpbmFyeV90LjJbLGMoMTIsMTEsMToxMCldDQoNCiNKb2luIGRhdC5kb2xfd2F0ZXIuZmlsLndlZWsuMTEuYmluYXJ5X3QuMyB3aXRoIGF0dHJpYnV0ZXMuZG9sX3JkcF93YXRlci5maWwuMg0KSW50ZXIuY29yZS56T1RVLndlZWsuMTEgPC0gZGF0LmRvbF93YXRlci5maWwud2Vlay4xMS5iaW5hcnlfdC4zICU+JSBsZWZ0X2pvaW4oYXR0cmlidXRlcy5kb2xfcmRwX3dhdGVyLmZpbC4yKQ0KZGltKEludGVyLmNvcmUuek9UVS53ZWVrLjExKQ0KIzE0NjggICAxOA0KI1ZpZXcoSW50ZXIuY29yZS56T1RVLndlZWsuMTFbMToxMCxdKQ0KDQojUmVvcmRlcg0KSW50ZXIuY29yZS56T1RVLndlZWsuMTFfMiA8LSBJbnRlci5jb3JlLnpPVFUud2Vlay4xMVssYygxLDIsMTcsMzoxMildDQpkaW0oSW50ZXIuY29yZS56T1RVLndlZWsuMTFfMikNCiMjMTQ2OCAxMw0KDQpuYW1lcyhJbnRlci5jb3JlLnpPVFUud2Vlay4xMV8yKQ0KDQojT3JkZXIgYWNjb3JkaW5nIHRvIEludGVyLmNvcmUuek9UVS53ZWVrLjExXzIkVG90YWwucmVsLmFidW5kLnpPVFUNCkludGVyLmNvcmUuek9UVS53ZWVrLjExXzMgPC0gSW50ZXIuY29yZS56T1RVLndlZWsuMTFfMltvcmRlcihJbnRlci5jb3JlLnpPVFUud2Vlay4xMV8yJFRvdGFsLnJlbC5hYnVuZC56T1RVKSxdDQojVmlldyhJbnRlci5jb3JlLnpPVFUud2Vlay4xMV8zWywxOjJdKQ0KDQojQW55IGludGVyLmNvcmUuT1RVcyBhYm92ZSAxMDAlICg9PSAxLjApPw0KSW50ZXIuY29yZS56T1RVLndlZWsuMTFfMS4wIDwtIEludGVyLmNvcmUuek9UVS53ZWVrLjExXzMgJT4lIGZpbHRlciAoVG90YWwucmVsLmFidW5kLnpPVFUgPT0gMS4wKQ0KZGltKEludGVyLmNvcmUuek9UVS53ZWVrLjExXzEuMCkNCiMzMw0KDQojRGV0ZXJtaW5lIHJlbGF0aXZlIGFidW5kYW5jZSBvZiB0aG9zZSBjb3JlIHpPVFVzDQpJbnRlci5jb3JlX3dlZWsuMTEgPC0gSW50ZXIuY29yZS56T1RVLndlZWsuMTFfMS4wJHZhcmlhYmxlDQoNClJlbC5hYnVuZF93ZWVrLjExLjIgPC0gUmVsLmFidW5kX3dlZWsuMTENCg0Kcm93bmFtZXMoUmVsLmFidW5kX3dlZWsuMTEuMikgPC0gUmVsLmFidW5kX3dlZWsuMTEuMiR2YXJpYWJsZQ0KDQpSZWwuYWJ1bmRfd2Vlay4xMS5Db3JlIDwtIFJlbC5hYnVuZF93ZWVrLjExLjJbSW50ZXIuY29yZV93ZWVrLjExLF0NCg0Kc3VtKFJlbC5hYnVuZF93ZWVrLjExLkNvcmVbLDJdKQ0KIzAuMjY1MjY2OQ0KDQp3cml0ZS5jc3YoUmVsLmFidW5kX3dlZWsuMTEuQ29yZSwgJ1JlbC5hYnVuZF93ZWVrLjExLkNvcmUuY3N2JykNCmBgYA0KDQojIyMjIyMjIyMjIyMjIyMjIyMjIyMNCiMjI1dlZWsgMTkgKDEwIGRvbHBoaW5zKQ0KYGBge3J9DQojIyMjIyMjIyMjIyMjIyMjIyMjIyMNCiMjMTkgKDEwIGRvbHBoaW5zKQ0KZGF0LmRvbF93YXRlci5maWwud2Vlay4xOSA8LSANCiAgZGF0LmRvbF93YXRlci5maWwudC4xOVtjKDIwOjI5KSxdDQoNCiNWaWV3KGRhdC5kb2xfd2F0ZXIuZmlsLndlZWsuMTlbLDE6MTBdKQ0KZGltKGRhdC5kb2xfd2F0ZXIuZmlsLndlZWsuMTkpDQojMTAgMTQ3NA0KDQp3cml0ZS5jc3YoZGF0LmRvbF93YXRlci5maWwud2Vlay4xOSwgJ2RhdC5kb2xfd2F0ZXIuZmlsLndlZWsuMTkuY3N2JykNCg0KIyMjUmVsYXRpdmUgYWJ1bmRhbmNlDQpkYXQuZG9sX3dhdGVyLmZpbC53ZWVrLjE5LjIgPC0gZGF0LmRvbF93YXRlci5maWwud2Vlay4xOVssLWMoMSwzKV0NCmRpbShkYXQuZG9sX3dhdGVyLmZpbC53ZWVrLjE5LjIpDQojMTAgMTQ3Mg0KDQpkYXQuZG9sX3dhdGVyLmZpbC53ZWVrLjE5X3JlbC5hYnVuZCA8LSBkYXQuZG9sX3dhdGVyLmZpbC53ZWVrLjE5LjJbLDI6MTQ3Ml0gL3Jvd1N1bXMoZGF0LmRvbF93YXRlci5maWwud2Vlay4xOS4yWywyOjE0NzJdKQ0KDQojVHJhbnNwb3NlDQpkYXQuZG9sX3dhdGVyLmZpbC53ZWVrLjE5X3JlbC5hYnVuZF90IDwtIGFzLmRhdGEuZnJhbWUodChkYXQuZG9sX3dhdGVyLmZpbC53ZWVrLjE5X3JlbC5hYnVuZCkpDQoNCmRhdC5kb2xfd2F0ZXIuZmlsLndlZWsuMTlfcmVsLmFidW5kX3QkdmFyaWFibGUgPC0gcm93bmFtZXMoZGF0LmRvbF93YXRlci5maWwud2Vlay4xOV9yZWwuYWJ1bmRfdCkNCmRpbShkYXQuZG9sX3dhdGVyLmZpbC53ZWVrLjE5X3JlbC5hYnVuZF90KQ0KIzE0NzEgICAxMQ0KDQpkYXQuZG9sX3dhdGVyLmZpbC53ZWVrLjE5X3JlbC5hYnVuZF90LjIgPC0gZGF0LmRvbF93YXRlci5maWwud2Vlay4xOV9yZWwuYWJ1bmRfdCAlPiUgbXV0YXRlKG1lYW5fb2Zfcm93cyA9IHJvd01lYW5zKGRhdC5kb2xfd2F0ZXIuZmlsLndlZWsuMTlfcmVsLmFidW5kX3RbLDE6MTBdKSkNCg0KUmVsLmFidW5kX3dlZWsuMTkgPC0gZGF0LmRvbF93YXRlci5maWwud2Vlay4xOV9yZWwuYWJ1bmRfdC4yWywxMToxMl0NCg0KI0NvbnZlcnQgZGF0YWZyYW1lIGludG8gYmluYXJ5DQpkYXQuZG9sX3dhdGVyLmZpbC53ZWVrLjE5LmJpbmFyeSA8LSBkYXQuZG9sX3dhdGVyLmZpbC53ZWVrLjE5Wyw0OjE0NzFdDQoNCmRhdC5kb2xfd2F0ZXIuZmlsLndlZWsuMTkuYmluYXJ5IFtdICA8LSAgKyhkYXQuZG9sX3dhdGVyLmZpbC53ZWVrLjE5LmJpbmFyeSAgPiAwKQ0KDQojVmlldyhkYXQuZG9sX3dhdGVyLmZpbC53ZWVrLjE5LmJpbmFyeSBbLDE6MTBdKQ0KDQojVHJhbnNwb3NlIGRhdC5kb2xfd2F0ZXIuZmlsLndlZWsuMTkuYmluYXJ5DQpkYXQuZG9sX3dhdGVyLmZpbC53ZWVrLjE5LmJpbmFyeV90IDwtIGFzLmRhdGEuZnJhbWUodChkYXQuZG9sX3dhdGVyLmZpbC53ZWVrLjE5LmJpbmFyeSkpDQojVmlldyhkYXQuZG9sX3dhdGVyLmZpbC53ZWVrLjE5LmJpbmFyeVsxOjEwLF0pDQoNCiNDcmVhdGUgY29sIFRvdGFsLnJlbC5hYnVuZC4NCmRhdC5kb2xfd2F0ZXIuZmlsLndlZWsuMTkuYmluYXJ5X3QuMiA8LSBkYXQuZG9sX3dhdGVyLmZpbC53ZWVrLjE5LmJpbmFyeV90ICU+JSANCiAgbXV0YXRlIChUb3RhbC5yZWwuYWJ1bmQuek9UVSA9IHJvd1N1bXMoZGF0LmRvbF93YXRlci5maWwud2Vlay4xOS5iaW5hcnlfdCkvMTApDQoNCiNWaWV3KGRhdC5kb2xfd2F0ZXIuZmlsLndlZWsuMTkuYmluYXJ5X3QuMlsxOjEwLF0pDQoNCiNHaXZlIGRhdC5kb2xfd2F0ZXIuZmlsLndlZWsuMTkuYmluYXJ5X3QuMiByb3duYW1lcyBhcyBjb2wgdmFyaWFibGUNCmRhdC5kb2xfd2F0ZXIuZmlsLndlZWsuMTkuYmluYXJ5X3QuMiR2YXJpYWJsZSA8LSByb3duYW1lcyhkYXQuZG9sX3dhdGVyLmZpbC53ZWVrLjE5LmJpbmFyeV90KQ0KZGltKGRhdC5kb2xfd2F0ZXIuZmlsLndlZWsuMTkuYmluYXJ5X3QuMikNCiMxNDY4IDEyDQoNCiNSZW9yZGVyDQpkYXQuZG9sX3dhdGVyLmZpbC53ZWVrLjE5LmJpbmFyeV90LjMgPC0gZGF0LmRvbF93YXRlci5maWwud2Vlay4xOS5iaW5hcnlfdC4yWyxjKDEyLDExLDE6MTApXQ0KDQojSm9pbiBkYXQuZG9sX3dhdGVyLmZpbC53ZWVrLjE5LmJpbmFyeV90LjMgd2l0aCBhdHRyaWJ1dGVzLmRvbF9yZHBfd2F0ZXIuZmlsLjINCkludGVyLmNvcmUuek9UVS53ZWVrLjE5IDwtIGRhdC5kb2xfd2F0ZXIuZmlsLndlZWsuMTkuYmluYXJ5X3QuMyAlPiUgbGVmdF9qb2luKGF0dHJpYnV0ZXMuZG9sX3JkcF93YXRlci5maWwuMikNCmRpbShJbnRlci5jb3JlLnpPVFUud2Vlay4xOSkNCiMxNDY4ICAgMTgNCiNWaWV3KEludGVyLmNvcmUuek9UVS53ZWVrLjE5WzE6MTAsXSkNCg0KI1Jlb3JkZXINCkludGVyLmNvcmUuek9UVS53ZWVrLjE5XzIgPC0gSW50ZXIuY29yZS56T1RVLndlZWsuMTlbLGMoMSwyLDE3LDM6MTIpXQ0KZGltKEludGVyLmNvcmUuek9UVS53ZWVrLjE5XzIpDQojIzE0NjggMTMNCg0KbmFtZXMoSW50ZXIuY29yZS56T1RVLndlZWsuMTlfMikNCg0KI09yZGVyIGFjY29yZGluZyB0byBJbnRlci5jb3JlLnpPVFUud2Vlay4xOV8yJFRvdGFsLnJlbC5hYnVuZC56T1RVDQpJbnRlci5jb3JlLnpPVFUud2Vlay4xOV8zIDwtIEludGVyLmNvcmUuek9UVS53ZWVrLjE5XzJbb3JkZXIoSW50ZXIuY29yZS56T1RVLndlZWsuMTlfMiRUb3RhbC5yZWwuYWJ1bmQuek9UVSksXQ0KI1ZpZXcoSW50ZXIuY29yZS56T1RVLndlZWsuMTlfM1ssMToyXSkNCg0KI0FueSBpbnRlci5jb3JlLk9UVXMgYWJvdmUgMTAwJSAoPT0gMS4wKT8NCkludGVyLmNvcmUuek9UVS53ZWVrLjE5XzEuMCA8LSBJbnRlci5jb3JlLnpPVFUud2Vlay4xOV8zICU+JSBmaWx0ZXIgKFRvdGFsLnJlbC5hYnVuZC56T1RVID09IDEuMCkNCmRpbShJbnRlci5jb3JlLnpPVFUud2Vlay4xOV8xLjApDQojNjYNCg0KI0RldGVybWluZSByZWxhdGl2ZSBhYnVuZGFuY2Ugb2YgdGhvc2UgY29yZSB6T1RVcw0KSW50ZXIuY29yZV93ZWVrLjE5IDwtIEludGVyLmNvcmUuek9UVS53ZWVrLjE5XzEuMCR2YXJpYWJsZQ0KDQpSZWwuYWJ1bmRfd2Vlay4xOS4yIDwtIFJlbC5hYnVuZF93ZWVrLjE5DQoNCnJvd25hbWVzKFJlbC5hYnVuZF93ZWVrLjE5LjIpIDwtIFJlbC5hYnVuZF93ZWVrLjE5LjIkdmFyaWFibGUNCg0KUmVsLmFidW5kX3dlZWsuMTkuMiA8LSBSZWwuYWJ1bmRfd2Vlay4xOS4yW0ludGVyLmNvcmVfd2Vlay4xOSxdDQoNCnN1bShJbnRlci5jb3JlX3dlZWsuMTlbLDJdKQ0KIzAuMTg1Njk4Ng0KDQojRGV0ZXJtaW5lIHJlbGF0aXZlIGFidW5kYW5jZSBvZiB0aG9zZSBjb3JlIHpPVFVzDQpJbnRlci5jb3JlX3dlZWsuMTkgPC0gSW50ZXIuY29yZS56T1RVLndlZWsuMTlfMS4wJHZhcmlhYmxlDQoNClJlbC5hYnVuZF93ZWVrLjE5LjIgPC0gUmVsLmFidW5kX3dlZWsuMTkNCg0Kcm93bmFtZXMoUmVsLmFidW5kX3dlZWsuMTkuMikgPC0gUmVsLmFidW5kX3dlZWsuMTkuMiR2YXJpYWJsZQ0KDQpSZWwuYWJ1bmRfd2Vlay4xOS5Db3JlIDwtIFJlbC5hYnVuZF93ZWVrLjE5LjJbSW50ZXIuY29yZV93ZWVrLjE5LF0NCg0Kc3VtKFJlbC5hYnVuZF93ZWVrLjE5LkNvcmVbLDJdKQ0KIzAuNDEzMjU3Mw0KYGBgDQoNCiMjIyMjIyMjIyMjIyMjIyMjIyMjIw0KIyNXZWVrIDI4ICgxMCBkb2xwaGlucykNCmBgYHtyfQ0KIyMjIyMjIyMjIyMjIyMjIyMjIyMjDQojIzI4ICgxMCBkb2xwaGlucykNCmRhdC5kb2xfd2F0ZXIuZmlsLndlZWsuMjggPC0gDQogIGRhdC5kb2xfd2F0ZXIuZmlsLnQuMTlbYyg0ODo1NyksXQ0KDQojVmlldyhkYXQuZG9sX3dhdGVyLmZpbC53ZWVrLjI4WywxOjEwXSkNCmRpbShkYXQuZG9sX3dhdGVyLmZpbC53ZWVrLjI4KQ0KIzEwIDE0NzQNCg0Kd3JpdGUuY3N2KGRhdC5kb2xfd2F0ZXIuZmlsLndlZWsuMjgsICdkYXQuZG9sX3dhdGVyLmZpbC53ZWVrLjI4LmNzdicpDQoNCiMjI1JlbGF0aXZlIGFidW5kYW5jZQ0KZGF0LmRvbF93YXRlci5maWwud2Vlay4yOC4yIDwtIGRhdC5kb2xfd2F0ZXIuZmlsLndlZWsuMjhbLC1jKDEsMyldDQpkaW0oZGF0LmRvbF93YXRlci5maWwud2Vlay4yOC4yKQ0KIzEwIDE0NzINCg0KZGF0LmRvbF93YXRlci5maWwud2Vlay4yOF9yZWwuYWJ1bmQgPC0gZGF0LmRvbF93YXRlci5maWwud2Vlay4yOC4yWywyOjE0NzJdIC9yb3dTdW1zKGRhdC5kb2xfd2F0ZXIuZmlsLndlZWsuMjguMlssMjoxNDcyXSkNCg0KI1RyYW5zcG9zZQ0KZGF0LmRvbF93YXRlci5maWwud2Vlay4yOF9yZWwuYWJ1bmRfdCA8LSBhcy5kYXRhLmZyYW1lKHQoZGF0LmRvbF93YXRlci5maWwud2Vlay4yOF9yZWwuYWJ1bmQpKQ0KDQpkYXQuZG9sX3dhdGVyLmZpbC53ZWVrLjI4X3JlbC5hYnVuZF90JHZhcmlhYmxlIDwtIHJvd25hbWVzKGRhdC5kb2xfd2F0ZXIuZmlsLndlZWsuMjhfcmVsLmFidW5kX3QpDQpkaW0oZGF0LmRvbF93YXRlci5maWwud2Vlay4yOF9yZWwuYWJ1bmRfdCkNCiMxNDcxICAgMTENCg0KZGF0LmRvbF93YXRlci5maWwud2Vlay4yOF9yZWwuYWJ1bmRfdC4yIDwtIGRhdC5kb2xfd2F0ZXIuZmlsLndlZWsuMjhfcmVsLmFidW5kX3QgJT4lIG11dGF0ZShtZWFuX29mX3Jvd3MgPSByb3dNZWFucyhkYXQuZG9sX3dhdGVyLmZpbC53ZWVrLjI4X3JlbC5hYnVuZF90WywxOjEwXSkpDQoNClJlbC5hYnVuZF93ZWVrLjI4IDwtIGRhdC5kb2xfd2F0ZXIuZmlsLndlZWsuMjhfcmVsLmFidW5kX3QuMlssMTE6MTJdDQoNCiNDb252ZXJ0IGRhdGFmcmFtZSBpbnRvIGJpbmFyeQ0KZGF0LmRvbF93YXRlci5maWwud2Vlay4yOC5iaW5hcnkgPC0gZGF0LmRvbF93YXRlci5maWwud2Vlay4yOFssNDoxNDcxXQ0KDQpkYXQuZG9sX3dhdGVyLmZpbC53ZWVrLjI4LmJpbmFyeSBbXSAgPC0gICsoZGF0LmRvbF93YXRlci5maWwud2Vlay4yOC5iaW5hcnkgID4gMCkNCg0KI1ZpZXcoZGF0LmRvbF93YXRlci5maWwud2Vlay4yOC5iaW5hcnkgWywxOjEwXSkNCg0KI1RyYW5zcG9zZSBkYXQuZG9sX3dhdGVyLmZpbC53ZWVrLjI4LmJpbmFyeQ0KZGF0LmRvbF93YXRlci5maWwud2Vlay4yOC5iaW5hcnlfdCA8LSBhcy5kYXRhLmZyYW1lKHQoZGF0LmRvbF93YXRlci5maWwud2Vlay4yOC5iaW5hcnkpKQ0KI1ZpZXcoZGF0LmRvbF93YXRlci5maWwud2Vlay4yOC5iaW5hcnlbMToxMCxdKQ0KDQojQ3JlYXRlIGNvbCBUb3RhbC5yZWwuYWJ1bmQuDQpkYXQuZG9sX3dhdGVyLmZpbC53ZWVrLjI4LmJpbmFyeV90LjIgPC0gZGF0LmRvbF93YXRlci5maWwud2Vlay4yOC5iaW5hcnlfdCAlPiUgDQogIG11dGF0ZSAoVG90YWwucmVsLmFidW5kLnpPVFUgPSByb3dTdW1zKGRhdC5kb2xfd2F0ZXIuZmlsLndlZWsuMjguYmluYXJ5X3QpLzEwKQ0KDQojVmlldyhkYXQuZG9sX3dhdGVyLmZpbC53ZWVrLjI4LmJpbmFyeV90LjJbMToxMCxdKQ0KDQojR2l2ZSBkYXQuZG9sX3dhdGVyLmZpbC53ZWVrLjI4LmJpbmFyeV90LjIgcm93bmFtZXMgYXMgY29sIHZhcmlhYmxlDQpkYXQuZG9sX3dhdGVyLmZpbC53ZWVrLjI4LmJpbmFyeV90LjIkdmFyaWFibGUgPC0gcm93bmFtZXMoZGF0LmRvbF93YXRlci5maWwud2Vlay4yOC5iaW5hcnlfdCkNCmRpbShkYXQuZG9sX3dhdGVyLmZpbC53ZWVrLjI4LmJpbmFyeV90LjIpDQojMTQ2OCAxMg0KDQojUmVvcmRlcg0KZGF0LmRvbF93YXRlci5maWwud2Vlay4yOC5iaW5hcnlfdC4zIDwtIGRhdC5kb2xfd2F0ZXIuZmlsLndlZWsuMjguYmluYXJ5X3QuMlssYygxMiwxMSwxOjEwKV0NCg0KI0pvaW4gZGF0LmRvbF93YXRlci5maWwud2Vlay4yOC5iaW5hcnlfdC4zIHdpdGggYXR0cmlidXRlcy5kb2xfcmRwX3dhdGVyLmZpbC4yDQpJbnRlci5jb3JlLnpPVFUud2Vlay4yOCA8LSBkYXQuZG9sX3dhdGVyLmZpbC53ZWVrLjI4LmJpbmFyeV90LjMgJT4lIGxlZnRfam9pbihhdHRyaWJ1dGVzLmRvbF9yZHBfd2F0ZXIuZmlsLjIpDQpkaW0oSW50ZXIuY29yZS56T1RVLndlZWsuMjgpDQojMTQ2OCAgIDE4DQojVmlldyhJbnRlci5jb3JlLnpPVFUud2Vlay4yOFsxOjEwLF0pDQoNCiNSZW9yZGVyDQpJbnRlci5jb3JlLnpPVFUud2Vlay4yOF8yIDwtIEludGVyLmNvcmUuek9UVS53ZWVrLjI4WyxjKDEsMiwxNywzOjEyKV0NCmRpbShJbnRlci5jb3JlLnpPVFUud2Vlay4yOF8yKQ0KIyMxNDY4IDEzDQoNCm5hbWVzKEludGVyLmNvcmUuek9UVS53ZWVrLjI4XzIpDQoNCiNPcmRlciBhY2NvcmRpbmcgdG8gSW50ZXIuY29yZS56T1RVLndlZWsuMjhfMiRUb3RhbC5yZWwuYWJ1bmQuek9UVQ0KSW50ZXIuY29yZS56T1RVLndlZWsuMjhfMyA8LSBJbnRlci5jb3JlLnpPVFUud2Vlay4yOF8yW29yZGVyKEludGVyLmNvcmUuek9UVS53ZWVrLjI4XzIkVG90YWwucmVsLmFidW5kLnpPVFUpLF0NCiNWaWV3KEludGVyLmNvcmUuek9UVS53ZWVrLjI4XzNbLDE6Ml0pDQoNCiNBbnkgaW50ZXIuY29yZS5PVFVzIGFib3ZlIDEwMCUgKD09IDEuMCk/DQpJbnRlci5jb3JlLnpPVFUud2Vlay4yOF8xLjAgPC0gSW50ZXIuY29yZS56T1RVLndlZWsuMjhfMyAlPiUgZmlsdGVyIChUb3RhbC5yZWwuYWJ1bmQuek9UVSA9PSAxLjApDQpkaW0oSW50ZXIuY29yZS56T1RVLndlZWsuMjhfMS4wKQ0KIzgNCg0KI0RldGVybWluZSByZWxhdGl2ZSBhYnVuZGFuY2Ugb2YgdGhvc2UgY29yZSB6T1RVcw0KSW50ZXIuY29yZV93ZWVrLjI4IDwtIEludGVyLmNvcmUuek9UVS53ZWVrLjI4XzEuMCR2YXJpYWJsZQ0KDQpSZWwuYWJ1bmRfd2Vlay4yOC4yIDwtIFJlbC5hYnVuZF93ZWVrLjI4DQoNCnJvd25hbWVzKFJlbC5hYnVuZF93ZWVrLjI4LjIpIDwtIFJlbC5hYnVuZF93ZWVrLjI4LjIkdmFyaWFibGUNCg0KUmVsLmFidW5kX3dlZWsuMjguQ29yZSA8LSBSZWwuYWJ1bmRfd2Vlay4yOC4yW0ludGVyLmNvcmVfd2Vlay42LF0NCg0Kc3VtKFJlbC5hYnVuZF93ZWVrLjI4LkNvcmVbLDJdKQ0KIzAuMTkzMjY4OA0KDQp3cml0ZS5jc3YoUmVsLmFidW5kX3dlZWsuMjguQ29yZSwgJ1JlbC5hYnVuZF93ZWVrLjI4LkNvcmUuY3N2JykNCmBgYA0KDQojIyMjIyMjIyMjIyMjIyMjIyMjIyMNCiMjI1dlZWsgMzcgKDEwIGRvbHBoaW5zKQ0KYGBge3J9DQojIyMjIyMjIyMjIyMjIyMjIyMjIyMNCiMjMzcgKDEwIGRvbHBoaW5zKQ0KZGF0LmRvbF93YXRlci5maWwud2Vlay4zNyA8LSANCiAgZGF0LmRvbF93YXRlci5maWwudC4xOVtjKDYxOjcwKSxdDQoNCiNWaWV3KGRhdC5kb2xfd2F0ZXIuZmlsLndlZWsuMzdbLDE6MTBdKQ0KZGltKGRhdC5kb2xfd2F0ZXIuZmlsLndlZWsuMzcpDQojMTAgMTQ3NA0KDQp3cml0ZS5jc3YoZGF0LmRvbF93YXRlci5maWwud2Vlay4zNywgJ2RhdC5kb2xfd2F0ZXIuZmlsLndlZWsuMzcuY3N2JykNCg0KIyMjUmVsYXRpdmUgYWJ1bmRhbmNlDQpkYXQuZG9sX3dhdGVyLmZpbC53ZWVrLjM3LjIgPC0gZGF0LmRvbF93YXRlci5maWwud2Vlay4zN1ssLWMoMSwzKV0NCmRpbShkYXQuZG9sX3dhdGVyLmZpbC53ZWVrLjM3LjIpDQojMTAgMTQ3Mg0KDQpkYXQuZG9sX3dhdGVyLmZpbC53ZWVrLjM3X3JlbC5hYnVuZCA8LSBkYXQuZG9sX3dhdGVyLmZpbC53ZWVrLjM3LjJbLDI6MTQ3Ml0gL3Jvd1N1bXMoZGF0LmRvbF93YXRlci5maWwud2Vlay4zNy4yWywyOjE0NzJdKQ0KDQojVHJhbnNwb3NlDQpkYXQuZG9sX3dhdGVyLmZpbC53ZWVrLjM3X3JlbC5hYnVuZF90IDwtIGFzLmRhdGEuZnJhbWUodChkYXQuZG9sX3dhdGVyLmZpbC53ZWVrLjM3X3JlbC5hYnVuZCkpDQoNCmRhdC5kb2xfd2F0ZXIuZmlsLndlZWsuMzdfcmVsLmFidW5kX3QkdmFyaWFibGUgPC0gcm93bmFtZXMoZGF0LmRvbF93YXRlci5maWwud2Vlay4zN19yZWwuYWJ1bmRfdCkNCmRpbShkYXQuZG9sX3dhdGVyLmZpbC53ZWVrLjM3X3JlbC5hYnVuZF90KQ0KIzE0NzEgICAxMQ0KDQpkYXQuZG9sX3dhdGVyLmZpbC53ZWVrLjM3X3JlbC5hYnVuZF90LjIgPC0gZGF0LmRvbF93YXRlci5maWwud2Vlay4zN19yZWwuYWJ1bmRfdCAlPiUgbXV0YXRlKG1lYW5fb2Zfcm93cyA9IHJvd01lYW5zKGRhdC5kb2xfd2F0ZXIuZmlsLndlZWsuMzdfcmVsLmFidW5kX3RbLDE6MTBdKSkNCg0KUmVsLmFidW5kX3dlZWsuMzcgPC0gZGF0LmRvbF93YXRlci5maWwud2Vlay4zN19yZWwuYWJ1bmRfdC4yWywxMToxMl0NCg0KI0NvbnZlcnQgZGF0YWZyYW1lIGludG8gYmluYXJ5DQpkYXQuZG9sX3dhdGVyLmZpbC53ZWVrLjM3LmJpbmFyeSA8LSBkYXQuZG9sX3dhdGVyLmZpbC53ZWVrLjM3Wyw0OjE0NzFdDQoNCmRhdC5kb2xfd2F0ZXIuZmlsLndlZWsuMzcuYmluYXJ5IFtdICA8LSAgKyhkYXQuZG9sX3dhdGVyLmZpbC53ZWVrLjM3LmJpbmFyeSAgPiAwKQ0KDQojVmlldyhkYXQuZG9sX3dhdGVyLmZpbC53ZWVrLjM3LmJpbmFyeSBbLDE6MTBdKQ0KDQojVHJhbnNwb3NlIGRhdC5kb2xfd2F0ZXIuZmlsLndlZWsuMzcuYmluYXJ5DQpkYXQuZG9sX3dhdGVyLmZpbC53ZWVrLjM3LmJpbmFyeV90IDwtIGFzLmRhdGEuZnJhbWUodChkYXQuZG9sX3dhdGVyLmZpbC53ZWVrLjM3LmJpbmFyeSkpDQojVmlldyhkYXQuZG9sX3dhdGVyLmZpbC53ZWVrLjM3LmJpbmFyeVsxOjEwLF0pDQoNCiNDcmVhdGUgY29sIFRvdGFsLnJlbC5hYnVuZC4NCmRhdC5kb2xfd2F0ZXIuZmlsLndlZWsuMzcuYmluYXJ5X3QuMiA8LSBkYXQuZG9sX3dhdGVyLmZpbC53ZWVrLjM3LmJpbmFyeV90ICU+JSANCiAgbXV0YXRlIChUb3RhbC5yZWwuYWJ1bmQuek9UVSA9IHJvd1N1bXMoZGF0LmRvbF93YXRlci5maWwud2Vlay4zNy5iaW5hcnlfdCkvMTApDQoNCiNWaWV3KGRhdC5kb2xfd2F0ZXIuZmlsLndlZWsuMzcuYmluYXJ5X3QuMlsxOjEwLF0pDQoNCiNHaXZlIGRhdC5kb2xfd2F0ZXIuZmlsLndlZWsuMzcuYmluYXJ5X3QuMiByb3duYW1lcyBhcyBjb2wgdmFyaWFibGUNCmRhdC5kb2xfd2F0ZXIuZmlsLndlZWsuMzcuYmluYXJ5X3QuMiR2YXJpYWJsZSA8LSByb3duYW1lcyhkYXQuZG9sX3dhdGVyLmZpbC53ZWVrLjM3LmJpbmFyeV90KQ0KZGltKGRhdC5kb2xfd2F0ZXIuZmlsLndlZWsuMzcuYmluYXJ5X3QuMikNCiMxNDY4IDEyDQoNCiNSZW9yZGVyDQpkYXQuZG9sX3dhdGVyLmZpbC53ZWVrLjM3LmJpbmFyeV90LjMgPC0gZGF0LmRvbF93YXRlci5maWwud2Vlay4zNy5iaW5hcnlfdC4yWyxjKDEyLDExLDE6MTApXQ0KDQojSm9pbiBkYXQuZG9sX3dhdGVyLmZpbC53ZWVrLjM3LmJpbmFyeV90LjMgd2l0aCBhdHRyaWJ1dGVzLmRvbF9yZHBfd2F0ZXIuZmlsLjINCkludGVyLmNvcmUuek9UVS53ZWVrLjM3IDwtIGRhdC5kb2xfd2F0ZXIuZmlsLndlZWsuMzcuYmluYXJ5X3QuMyAlPiUgbGVmdF9qb2luKGF0dHJpYnV0ZXMuZG9sX3JkcF93YXRlci5maWwuMikNCmRpbShJbnRlci5jb3JlLnpPVFUud2Vlay4zNykNCiMxNDY4ICAgMTgNCiNWaWV3KEludGVyLmNvcmUuek9UVS53ZWVrLjM3WzE6MTAsXSkNCg0KI1Jlb3JkZXINCkludGVyLmNvcmUuek9UVS53ZWVrLjM3XzIgPC0gSW50ZXIuY29yZS56T1RVLndlZWsuMzdbLGMoMSwyLDE3LDM6MTIpXQ0KZGltKEludGVyLmNvcmUuek9UVS53ZWVrLjM3XzIpDQojIzE0NjggMTMNCg0KbmFtZXMoSW50ZXIuY29yZS56T1RVLndlZWsuMzdfMikNCg0KI09yZGVyIGFjY29yZGluZyB0byBJbnRlci5jb3JlLnpPVFUud2Vlay4zN18yJFRvdGFsLnJlbC5hYnVuZC56T1RVDQpJbnRlci5jb3JlLnpPVFUud2Vlay4zN18zIDwtIEludGVyLmNvcmUuek9UVS53ZWVrLjM3XzJbb3JkZXIoSW50ZXIuY29yZS56T1RVLndlZWsuMzdfMiRUb3RhbC5yZWwuYWJ1bmQuek9UVSksXQ0KI1ZpZXcoSW50ZXIuY29yZS56T1RVLndlZWsuMzdfM1ssMToyXSkNCg0KI0FueSBpbnRlci5jb3JlLk9UVXMgYWJvdmUgMTAwJSAoPT0gMS4wKT8NCkludGVyLmNvcmUuek9UVS53ZWVrLjM3XzEuMCA8LSBJbnRlci5jb3JlLnpPVFUud2Vlay4zN18zICU+JSBmaWx0ZXIgKFRvdGFsLnJlbC5hYnVuZC56T1RVID09IDEuMCkNCmRpbShJbnRlci5jb3JlLnpPVFUud2Vlay4zN18xLjApDQojMTYNCg0KI0RldGVybWluZSByZWxhdGl2ZSBhYnVuZGFuY2Ugb2YgdGhvc2UgY29yZSB6T1RVcw0KSW50ZXIuY29yZV93ZWVrLjM3IDwtIEludGVyLmNvcmUuek9UVS53ZWVrLjM3XzEuMCR2YXJpYWJsZQ0KDQpSZWwuYWJ1bmRfd2Vlay4zNy4yIDwtIFJlbC5hYnVuZF93ZWVrLjM3DQoNCnJvd25hbWVzKFJlbC5hYnVuZF93ZWVrLjM3LjIpIDwtIFJlbC5hYnVuZF93ZWVrLjM3LjIkdmFyaWFibGUNCg0KUmVsLmFidW5kX3dlZWsuMzcuQ29yZSA8LSBSZWwuYWJ1bmRfd2Vlay4zNy4yW0ludGVyLmNvcmVfd2Vlay42LF0NCg0Kc3VtKFJlbC5hYnVuZF93ZWVrLjM3LkNvcmVbLDJdKQ0KIzAuMTY2NDI1Ng0KDQp3cml0ZS5jc3YoUmVsLmFidW5kX3dlZWsuMzcuQ29yZSwgJ1JlbC5hYnVuZF93ZWVrLjM3LkNvcmUuY3N2JykNCmBgYA0KIyMjIyMjIyMjIyMjIyMjIyMjIyMjIyMjIyMjIyMjIyMjIyMjIyMjIyMjIyMjIyMjIyMjIyMjIyMjIyMjIyMjIyMjIyMjIw0KIyMjIyMjI0hvdyBtYW55IGludGVyLWNvcmUgek9UVXMgZGlkIDEwIGRvbHBoaW5zIGF0IGFueSBzYW1wbGUgY29sbGVjdGlvbiBwb2ludCBpbiB0aW1lICh3ZWVrcyAyLCA2LCAxMSwgMTksIDI4LCAzNyk/DQpgYGB7cn0NCiMjIyNIb3cgbWFueSBpbnRlci1jb3JlIHpPVFVzIGRpZCAxMCBkb2xwaGlucyBhdCBhbnkgc2FtcGxlIGNvbGxlY3Rpb24gcG9pbnQgaW4gdGltZSAod2Vla3MgMiwgNiwgMTEsIDE5LCAyOCwgMzcpPw0KDQptZWFuKGxlbmd0aChJbnRlci5jb3JlLnpPVFUud2Vlay4yXzEuMCRUb3RhbC5yZWwuYWJ1bmQuek9UVSksbGVuZ3RoKEludGVyLmNvcmUuek9UVS53ZWVrLjZfMS4wJFRvdGFsLnJlbC5hYnVuZC56T1RVKSxsZW5ndGgoSW50ZXIuY29yZS56T1RVLndlZWsuMTFfMS4wJFRvdGFsLnJlbC5hYnVuZC56T1RVKSxsZW5ndGgoSW50ZXIuY29yZS56T1RVLndlZWsuMTlfMS4wJFRvdGFsLnJlbC5hYnVuZC56T1RVKSxsZW5ndGgoSW50ZXIuY29yZS56T1RVLndlZWsuMjhfMS4wJFRvdGFsLnJlbC5hYnVuZC56T1RVKSxsZW5ndGgoSW50ZXIuY29yZS56T1RVLndlZWsuMzdfMS4wJFRvdGFsLnJlbC5hYnVuZC56T1RVKSkNCiMzMg0Kc2QoYygxNiw4LDY2LDMzLDQwLDMyKSkNCiMyMA0KDQojIyNXaGF0IGlzIHRoZSBtZWFuIHJlbGF0aXZlIGFidW5kYW5jZSB0aGVzZSBpbnRlci1jb3JlIHpPVFVzIGFjY291bnRlZCBmb3IgaW4gZWFjaCBkb2xwaGluPw0KDQptZWFuKGMoc3VtKFJlbC5hYnVuZF93ZWVrLjIuQ29yZVssMl0pLHN1bShSZWwuYWJ1bmRfd2Vlay4xMS5Db3JlWywyXSksc3VtKFJlbC5hYnVuZF93ZWVrLjE5LkNvcmVbLDJdKSxzdW0oUmVsLmFidW5kX3dlZWsuMjguQ29yZVssMl0pLHN1bShSZWwuYWJ1bmRfd2Vlay4zNy5Db3JlWywyXSksc3VtKFJlbC5hYnVuZF93ZWVrLjYuQ29yZVssMl0pKSkNCiMwLjI0OTQ3MDENCg0Kc2QoYyhzdW0oUmVsLmFidW5kX3dlZWsuMi5Db3JlWywyXSksc3VtKFJlbC5hYnVuZF93ZWVrLjExLkNvcmVbLDJdKSxzdW0oUmVsLmFidW5kX3dlZWsuMTkuQ29yZVssMl0pLHN1bShSZWwuYWJ1bmRfd2Vlay4yOC5Db3JlWywyXSksc3VtKFJlbC5hYnVuZF93ZWVrLjM3LkNvcmVbLDJdKSxzdW0oUmVsLmFidW5kX3dlZWsuNi5Db3JlWywyXSkpKQ0KIzAuMDkxMzc0NzINCg0KbWluKGMoc3VtKFJlbC5hYnVuZF93ZWVrLjIuQ29yZVssMl0pLHN1bShSZWwuYWJ1bmRfd2Vlay4xMS5Db3JlWywyXSksc3VtKFJlbC5hYnVuZF93ZWVrLjE5LkNvcmVbLDJdKSxzdW0oUmVsLmFidW5kX3dlZWsuMjguQ29yZVssMl0pLHN1bShSZWwuYWJ1bmRfd2Vlay4zNy5Db3JlWywyXSksc3VtKFJlbC5hYnVuZF93ZWVrLjYuQ29yZVssMl0pKSkNCiMwLjE2NjQyNTYNCg0KbWF4KGMoc3VtKFJlbC5hYnVuZF93ZWVrLjIuQ29yZVssMl0pLHN1bShSZWwuYWJ1bmRfd2Vlay4xMS5Db3JlWywyXSksc3VtKFJlbC5hYnVuZF93ZWVrLjE5LkNvcmVbLDJdKSxzdW0oUmVsLmFidW5kX3dlZWsuMjguQ29yZVssMl0pLHN1bShSZWwuYWJ1bmRfd2Vlay4zNy5Db3JlWywyXSksc3VtKFJlbC5hYnVuZF93ZWVrLjYuQ29yZVssMl0pKSkNCiMwLjQxMzI1NzMNCg0KIyMjSG93IG1hbnkgaW50ZXItY29yZSB6T1RVcyB3ZXJlIGhhcmJvdXJlZCBieSB0aGUgZG9scGhpbnMgaW4gdG90YWw/DQoNCkludGVyLmNvcmVfdW5pcXVlIDwtIHVuaXF1ZShjKEludGVyLmNvcmUuek9UVS53ZWVrLjJfMS4wJHZhcmlhYmxlLCBJbnRlci5jb3JlLnpPVFUud2Vlay42XzEuMCR2YXJpYWJsZSwgSW50ZXIuY29yZS56T1RVLndlZWsuMTFfMS4wJHZhcmlhYmxlLCBJbnRlci5jb3JlLnpPVFUud2Vlay4xOV8xLjAkdmFyaWFibGUsIEludGVyLmNvcmUuek9UVS53ZWVrLjI4XzEuMCR2YXJpYWJsZSwgSW50ZXIuY29yZS56T1RVLndlZWsuMzdfMS4wJHZhcmlhYmxlKSkNCg0KbGVuZ3RoKEludGVyLmNvcmVfdW5pcXVlKQ0KIzk3DQoNCiNIb3cgbWFueSBvZiB0aGUgaW50ZXItY29yZSBpcyBpbmNsdWRlZCBpbiB0aGUgaW50cmEtY29yZT8NCkludGVyLmNvcmVfdW5pcXVlID0gZGF0LmRvbF93YXRlci5maWwuNHNhbXAuY29yZS5yZWwuYWJ1bmRfdC5jb3JlLjIkdmFyaWFibGUNCg0Kc2V0ZGlmZihJbnRlci5jb3JlX3VuaXF1ZSxJbnRyYS5jb3JlX3VuaXF1ZSkNCiNjaGFyYWN0ZXIoMCkNCg0KIyMjQ29yZS5pbnRlci51bmlxdWUgaXMgY29tcGxldGVseSBpbmNsdWRlZCBpbiBDb3JlLmludHJhIQ0KYGBgDQoNCiMjIyMjIyMjIyMjIyMjIyMjIyMjIyMjIyMNCiMjI0NyZWF0ZSBmcmVxdWVuY3kgaGlzdG9ncmFtIG9mIGludGVyLWNvcmUNCmBgYHtyfQ0KIyMjUHJlcGFyZSBkYXRhZnJhbWVzIChlLmcuLCBJbnRlci5jb3JlLnpPVFUud2Vlay4yXzEuMCkNCkludGVyLmNvcmUuYWxsLndlZWsuMiA8LSBJbnRlci5jb3JlLnpPVFUud2Vlay4yXzEuMFssYygxLDQpXQ0KSW50ZXIuY29yZS5hbGwud2Vlay42IDwtIEludGVyLmNvcmUuek9UVS53ZWVrLjZfMS4wWyxjKDEsNCldDQpJbnRlci5jb3JlLmFsbC53ZWVrLjExIDwtIEludGVyLmNvcmUuek9UVS53ZWVrLjExXzEuMFssYygxLDQpXQ0KSW50ZXIuY29yZS5hbGwud2Vlay4xOSA8LSBJbnRlci5jb3JlLnpPVFUud2Vlay4xOV8xLjBbLGMoMSw0KV0NCkludGVyLmNvcmUuYWxsLndlZWsuMjggPC0gSW50ZXIuY29yZS56T1RVLndlZWsuMjhfMS4wWyxjKDEsNCldDQpJbnRlci5jb3JlLmFsbC53ZWVrLjM3IDwtIEludGVyLmNvcmUuek9UVS53ZWVrLjM3XzEuMFssYygxLDQpXQ0KDQoNCkludGVyLmNvcmUuYWxsLndlZWtzIDwtIGZ1bGxfam9pbiAoYnkgPSBjKCd2YXJpYWJsZScpLCBJbnRlci5jb3JlLmFsbC53ZWVrLjIsIEludGVyLmNvcmUuYWxsLndlZWsuNikNCkludGVyLmNvcmUuYWxsLndlZWtzLjIgPC0gZnVsbF9qb2luIChieSA9IGMoJ3ZhcmlhYmxlJyksIEludGVyLmNvcmUuYWxsLndlZWtzLCBJbnRlci5jb3JlLmFsbC53ZWVrLjExKQ0KSW50ZXIuY29yZS5hbGwud2Vla3MuMyA8LSBmdWxsX2pvaW4gKGJ5ID0gYygndmFyaWFibGUnKSwgSW50ZXIuY29yZS5hbGwud2Vla3MuMiwgSW50ZXIuY29yZS5hbGwud2Vlay4xOSkNCkludGVyLmNvcmUuYWxsLndlZWtzLjQgPC0gZnVsbF9qb2luIChieSA9IGMoJ3ZhcmlhYmxlJyksIEludGVyLmNvcmUuYWxsLndlZWtzLjMsIEludGVyLmNvcmUuYWxsLndlZWsuMjgpDQpJbnRlci5jb3JlLmFsbC53ZWVrcy41IDwtIGZ1bGxfam9pbiAoYnkgPSBjKCd2YXJpYWJsZScpLCBJbnRlci5jb3JlLmFsbC53ZWVrcy40LCBJbnRlci5jb3JlLmFsbC53ZWVrLjM3KQ0KDQpkaW0oSW50ZXIuY29yZS5hbGwud2Vla3MuNSkNCiM5NyAgNw0KDQpJbnRlci5jb3JlLmFsbC53ZWVrcy42IDwtIEludGVyLmNvcmUuYWxsLndlZWtzLjUgJT4lIG11dGF0ZSAoc3VtX29mX3Jvd3M9cm93U3VtcyhJbnRlci5jb3JlLmFsbC53ZWVrcy41WywyOjddLG5hLnJtPVRSVUUpKSAlPiUgYXJyYW5nZShzdW1fb2Zfcm93cykNCiNWaWV3KEludGVyLmNvcmUuYWxsLndlZWtzLjYpDQoNCkludGVyLmNvcmUuaGlzdG8gPC0gYXMuZGF0YS5mcmFtZShJbnRlci5jb3JlLmFsbC53ZWVrcy42JHN1bV9vZl9yb3dzKQ0KbmFtZXMoSW50ZXIuY29yZS5oaXN0bykgPC0gJ0ludGVyLmNvcmUuaGlzdG8nDQoNCkludGVyLmNvcmUuaGlzdG9ncmFtID0NCiAgZ2dwbG90KEludGVyLmNvcmUuaGlzdG8sIGFlcyh4ID0gSW50ZXIuY29yZS5oaXN0bykpICsNCiAgZ2VvbV9oaXN0b2dyYW0oYmlud2lkdGggPSAwLjUpKw0KICB0aGVtZV9idygpICsNCiAgdGhlbWUocGxvdC50aXRsZSA9IGVsZW1lbnRfdGV4dChzaXplID0gMTQsIGZhbWlseSA9ICJUYWhvbWEiLCBmYWNlID0gImJvbGQiKSwNCiAgICAgICAgdGV4dCA9IGVsZW1lbnRfdGV4dChzaXplID0gMTIsIGZhbWlseSA9ICJUYWhvbWEiKSwNCiAgICAgICAgYXhpcy50aXRsZSA9IGVsZW1lbnRfdGV4dCgpLA0KICAgICAgICBheGlzLnRleHQueD1lbGVtZW50X3RleHQoc2l6ZSA9IDExKSkgKw0KICBzY2FsZV94X2NvbnRpbnVvdXMobmFtZSA9ICJOdW1iZXIgb2Ygc2FtcGxpbmcgcG9pbnRzIHdoZW4gaW50ZXItY29yZSB6T1RVcyB3ZXJlIGZvdW5kIiwgDQogICAgICAgICAgICAgICAgICAgICBicmVha3MgPSBzZXEoMTo2KSkgKw0KICBzY2FsZV95X2NvbnRpbnVvdXMobmFtZSA9ICJOdW1iZXIgb2YgaW50ZXItY29yZSB6T1RVcyIpDQoNCmdnc2F2ZSgiSW50ZXIuY29yZS5oaXN0b2dyYW0uanBnIiwgcGxvdCA9IEludGVyLmNvcmUuaGlzdG9ncmFtICwgZGV2aWNlID0gJ2pwZycsIHdpZHRoID0gMTY4LCBoZWlnaHQgPSAxMzAsIHVuaXRzID0gIm1tIiwNCiAgICAgICBkcGkgPSAzMDAsIGxpbWl0c2l6ZSA9IFRSVUUpDQpgYGANCg0KIyMjIyMjIyMjIyMjIyMjIyMjIyMjIyMjIyMjIyMjIyMjIyMjIyMjIyMjIyMjIyMNCiMjIyMjIyNUZW1wb3JhbCBkeW5hbWljcyBvZiBpbnRlcl9jb3JlOiBTY2F0dGVycGxvdA0KYGBge3J9DQojIyNDcmVhdGUgc2NhdHRlcnBsb3Qgb2YgaW50ZXJfY29yZSBvdmVyIHdlZWtzIDIsIDYsIDExLCAxOSwgMjgsIDM3DQpsZW5ndGgoSW50ZXIuY29yZS5hbGwud2Vlay4yJHZhcmlhYmxlKQ0KIzMyDQpsZW5ndGgoSW50ZXIuY29yZS5hbGwud2Vlay42JHZhcmlhYmxlKQ0KIzQwDQpsZW5ndGgoSW50ZXIuY29yZS5hbGwud2Vlay4xMSR2YXJpYWJsZSkNCiMzMw0KbGVuZ3RoKEludGVyLmNvcmUuYWxsLndlZWsuMTkkdmFyaWFibGUpDQojNjYNCmxlbmd0aChJbnRlci5jb3JlLmFsbC53ZWVrLjI4JHZhcmlhYmxlKQ0KIzgNCmxlbmd0aChJbnRlci5jb3JlLmFsbC53ZWVrLjM3JHZhcmlhYmxlKQ0KIzE2DQoNCkludGVyX2NvcmUgPC0gYXMubnVtZXJpYyhjKGxlbmd0aChJbnRlci5jb3JlLmFsbC53ZWVrLjIkdmFyaWFibGUpLGxlbmd0aChJbnRlci5jb3JlLmFsbC53ZWVrLjYkdmFyaWFibGUpLGxlbmd0aChJbnRlci5jb3JlLmFsbC53ZWVrLjExJHZhcmlhYmxlKSxsZW5ndGgoSW50ZXIuY29yZS5hbGwud2Vlay4xOSR2YXJpYWJsZSksbGVuZ3RoKEludGVyLmNvcmUuYWxsLndlZWsuMjgkdmFyaWFibGUpLGxlbmd0aChJbnRlci5jb3JlLmFsbC53ZWVrLjM3JHZhcmlhYmxlKSkpDQoNCldlZWsgPC0gYygyLDYsMTEsMTksMjgsMzcpDQoNCkludGVyX2NvcmVfYnkud2VlayA8LSBjYmluZChXZWVrLCBJbnRlcl9jb3JlKQ0KDQpJbnRlcl9jb3JlX2J5LndlZWsucGxvdCA8LSBnZ3Bsb3QoSW50ZXJfY29yZV9ieS53ZWVrLCBhZXMoeD1XZWVrLCB5PUludGVyX2NvcmUpKSArDQogIGdlb21fcG9pbnQoKSArIA0KICAgdGhlbWVfYncoKSAgKw0KICAgc2NhbGVfeV9jb250aW51b3VzKG5hbWUgPSAiTnVtYmVyIG9mIGludGVyLWNvcmUgek9UVXMiKSsgDQogIGdlb21fbGluZShsaW5ldHlwZSA9ICJkYXNoZWQiKQ0KDQpnZ3NhdmUoIkludGVyX2NvcmVfYnkud2Vlay5wbG90LmpwZyIsIHBsb3QgPSBJbnRlcl9jb3JlX2J5LndlZWsucGxvdCAsIGRldmljZSA9ICdqcGcnLCB3aWR0aCA9IDIwMCwgaGVpZ2h0ID0gMTMwLCB1bml0cyA9ICJtbSIsDQogICAgICAgZHBpID0gMzAwLCBsaW1pdHNpemUgPSBUUlVFKQ0KDQpgYGANCg0KIyMjIyMjIyMjIyMjIyMjIyMjIyMjIyMjIyMjIyMjIyMjIyMjIyMjIyMjIyMjIyMNCiMjIyMjIyNJbXBhY3Qgb2YgQUIgdHJlYXRtZW50IChhbGwgbGV2ZWxzKSAtLT4gbm9uLXNpZ25pZmljYW50DQpgYGB7cn0NCiMjI0NoZWNrIGFsbCBkb2xwaGlucyBmb3IgaW1wYWN0IG9mIEFCIHRyZWF0bWVudCAoTm9uZSwgQmVmb3JlLCBBZnRlciwgRGlyZWN0bHlfQWZ0ZXIpDQoNCiMjI1ByZXBhcmUgZGF0YXNldCwgY3JlYXRlIGNvbHMgSUQsIHRpbWUgYW5kIEFCLlRyZWF0bWVudC5hbGwuZG9sDQpkYXQuZG9sX3dhdGVyLmZpbC50LjMwIDwtIGRhdC5kb2xfd2F0ZXIuZmlsLnQuMg0KDQpkYXQuZG9sX3dhdGVyLmZpbC50LjMwJERvbHBoaW4gICA8LSByb3duYW1lcyhkYXQuZG9sX3dhdGVyLmZpbC50LjMwKQ0KZGF0LmRvbF93YXRlci5maWwudC4zMSAgICAgICAgICAgPC0gZGF0LmRvbF93YXRlci5maWwudC4zMA0KZGF0LmRvbF93YXRlci5maWwudC4zMSREb2xwaGluLjIgPC0gZGF0LmRvbF93YXRlci5maWwudC4zMSREb2xwaGluDQpkaW0oZGF0LmRvbF93YXRlci5maWwudC4zMSkNCiM4MyAxNDczDQoNCmRhdC5kb2xfd2F0ZXIuZmlsLnQuMzIgPC0gZGF0LmRvbF93YXRlci5maWwudC4zMVssYygxNDcyLDE0NzMsMToxNDcxKV0NCg0KZGF0LmRvbF93YXRlci5maWwudC4zMyA8LSBzZXBhcmF0ZShkYXQuZG9sX3dhdGVyLmZpbC50LjMyLERvbHBoaW4uMiwgYygnSUQnLCd0aW1lJykpDQpkYXQuZG9sX3dhdGVyLmZpbC50LjM0IDwtIGFycmFuZ2UoZGF0LmRvbF93YXRlci5maWwudC4zMywgRG9scGhpbikNCmRhdC5kb2xfd2F0ZXIuZmlsLnQuMzUgPC0gZGF0LmRvbF93YXRlci5maWwudC4zNFstYygyLDQzKSxdDQoNCiMjI0FkZCBjb2wgJ0FCLlRyZWF0bWVudC5hbGwuZG9sJw0KDQpBQi50cmVhdG1lbnQgPC0gYXMuZGF0YS5mcmFtZShjKHJlcCgnTm9uZScsMTEpLCByZXAoJ0FmdGVyJywzKSxyZXAoJ0JlZm9yZScsMSkscmVwKCdBZnRlcicsMSksIHJlcCgnRGlyZWN0bHlfYWZ0ZXInLDEpLCByZXAoJ0FmdGVyJywzKSwgcmVwKCdCZWZvcmUnLDEpLCByZXAoJ0FmdGVyJywyKSwgcmVwKCdCZWZvcmUnLDIpLCByZXAoJ05vbmUnLDExKSwNCiAgICAgICAgICAgICAgICAgICAgICAgICAgICAgICAgcmVwKCdCZWZvcmUnLDMpLCByZXAoJ0RpcmVjdGx5X2FmdGVyJywxKSwgcmVwKCdBZnRlcicsMykscmVwKCdCZWZvcmUnLDEpLCByZXAoJ05vbmUnLCAyOCksIA0KICAgICAgICAgICAgICAgICAgICAgICAgICAgICAgICByZXAoJ0JlZm9yZScsMyksIHJlcCgnRGlyZWN0bHlfYWZ0ZXInLDEpLCByZXAoJ0FmdGVyJywxKSwgcmVwKCdCZWZvcmUnLDEpLCByZXAoJ0FmdGVyJywyKSwgcmVwKCdCZWZvcmUnLDEpKSkNCg0KbmFtZXMoQUIudHJlYXRtZW50KTwtICdBQi5UcmVhdG1lbnQuYWxsLmRvbCcNCmRpbShBQi50cmVhdG1lbnQpDQoNCmRhdC5kb2xfd2F0ZXIuZmlsLnQuMzYgPC0gY2JpbmQoQUIudHJlYXRtZW50LGRhdC5kb2xfd2F0ZXIuZmlsLnQuMzUpDQojVmlldyhkYXQuZG9sX3dhdGVyLmZpbC50LjM2KQ0KZGltKGRhdC5kb2xfd2F0ZXIuZmlsLnQuMzYpDQojODEgMTQ3NQ0KDQpkYXQuZG9sX3dhdGVyLmZpbC50LjM2JGxvZ1RvdGFsQWJ1bmRhbmNlIDwtIGxvZyhhcHBseShkYXQuZG9sX3dhdGVyLmZpbC50LjM2Wyw1OjE0NzVdLDEsc3VtKSkNCg0Kek9UVS53YXRlci5maWwuQUIgPC0gbXZhYnVuZChkYXQuZG9sX3dhdGVyLmZpbC50LjM2Wyw1OjE0NzVdKQ0KDQpQcmUuZml0MS53YXRlci5maWwuQUIgPC0gbWFueWdsbSh6T1RVLndhdGVyLmZpbC5BQiB+IEFCLlRyZWF0bWVudC5hbGwuZG9sICsgdGltZSArIElEICsgb2Zmc2V0KGxvZ1RvdGFsQWJ1bmRhbmNlKSwgZGF0YSA9IGRhdC5kb2xfd2F0ZXIuZmlsLnQuMzYsIGZhbWlseT0ibmVnYXRpdmUuYmlub21pYWwiKQ0KDQojY2hlY2sgYXNzdW1wdGlvbnMNCnBsb3QoUHJlLmZpdDEud2F0ZXIuZmlsLkFCKQ0KDQojVGhpcyBpcyB0aGUgYWN0dWFsIHNpZ25pZmljYW5jZSB0ZXN0ICAgICAgICAgICAgICAgDQpmaXQuMS5kb2wudGVjaC5maWwuQUIgPSBtYW55Z2xtKHpPVFUud2F0ZXIuZmlsLkFCIH4gQUIuVHJlYXRtZW50LmFsbC5kb2wgKyB0aW1lICsgSUQgKyBvZmZzZXQobG9nVG90YWxBYnVuZGFuY2UpLCBkYXRhID0gZGF0LmRvbF93YXRlci5maWwudC4zNikNCmZpdC4yLmRvbC50ZWNoLmZpbC5BQiA9IG1hbnlnbG0oek9UVS53YXRlci5maWwuQUIgfiB0aW1lICsgSUQgKyBvZmZzZXQobG9nVG90YWxBYnVuZGFuY2UpLCBkYXRhID0gZGF0LmRvbF93YXRlci5maWwudC4zNikNCg0KI2FkanVzdGVkDQojZ2xvYmFsVGVzdDEuZG9sLndhdGVyLmZpbC5hZGp1c3RlZC5BQiA9IGFub3ZhKGZpdC4xLmRvbC50ZWNoLmZpbC5BQiwgZml0LjIuZG9sLnRlY2guZmlsLkFCLCBuQm9vdD0xMDAwLCBwLnVuaT0nYWRqdXN0ZWQnKQ0KDQojIE11bHRpdmFyaWF0ZSB0ZXN0Og0KIyAgICAgICAgICAgICAgICAgICAgICAgUmVzLkRmIERmLmRpZmYgIERldiBQcig+RGV2KSAgDQojIGZpdC4yLmRvbC50ZWNoLmZpbC5BQiAgICAgNTMgICAgICAgICAgICAgICAgICAgICAgICANCiMgZml0LjEuZG9sLnRlY2guZmlsLkFCICAgICA1MSAgICAgICAyIDMwNzEgICAgMC4wOTcgLg0KDQojc2F2ZShnbG9iYWxUZXN0MS5kb2wud2F0ZXIuZmlsLmFkanVzdGVkLkFCLCBmaWxlPSdnbG9iYWxUZXN0MS5kb2wud2F0ZXIuZmlsLmFkanVzdGVkLkFCLlJkYXRhJykNCmBgYA0KDQojIyMjIyMjIyMjIyMjIyMjIyMjIyMjIyMjIyMjIyMjIyMjIyMjIyMjIyMjIyMjIw0KIyMjIyMjI0ltcGFjdCBvZiBBQiB0cmVhdG1lbnQgKHNpY2sgdnMuIGhlYWx0aHkpDQpgYGB7cn0NCiMjI0NoZWNrIGFsbCBkb2xwaGlucyBmb3IgaW1wYWN0IG9mIEFCIHRyZWF0bWVudCAoTm9uZSAtLT4gJ2hlYWx0aHknLCAoQmVmb3JlLCBBZnRlciwgRGlyZWN0bHlfQWZ0ZXIpIC0tPiAnc2ljaycpDQoNCiMjI1VzZSBkYXQuZG9sX3dhdGVyLmZpbC50LjM1DQoNCiMjI0FkZCBjb2wgJ1RyZWF0bWVudCcgKHNpY2ssIGhlYWx0aHkpDQpUcmVhdG1lbnQgPC0gYyhyZXAoJ2hlYWx0aHknLCAxMSkscmVwKCdzaWNrJywxNCkscmVwKCdoZWFsdGh5JywgMTEpLCByZXAoJ3NpY2snLDgpLCByZXAoJ2hlYWx0aHknLCAyOCksIHJlcCgnc2ljaycsOSkpDQoNCmRhdC5kb2xfd2F0ZXIuZmlsLlRyZWF0bWVudCA8LSBjYmluZChUcmVhdG1lbnQsZGF0LmRvbF93YXRlci5maWwudC4zNSkNCiNWaWV3KGRhdC5kb2xfd2F0ZXIuZmlsLlRyZWF0bWVudCkNCmRpbShkYXQuZG9sX3dhdGVyLmZpbC5UcmVhdG1lbnQpDQojODEgMTQ3NQ0KDQpkYXQuZG9sX3dhdGVyLmZpbC5UcmVhdG1lbnQkRG9scGhpbiA8LSBhcy5mYWN0b3IoZGF0LmRvbF93YXRlci5maWwuVHJlYXRtZW50JERvbHBoaW4pDQpkYXQuZG9sX3dhdGVyLmZpbC5UcmVhdG1lbnQkSUQgPC0gYXMuZmFjdG9yKGRhdC5kb2xfd2F0ZXIuZmlsLlRyZWF0bWVudCRJRCkNCmRhdC5kb2xfd2F0ZXIuZmlsLlRyZWF0bWVudCR0aW1lIDwtIGFzLmZhY3RvcihkYXQuZG9sX3dhdGVyLmZpbC5UcmVhdG1lbnQkdGltZSkNCg0KZGF0LmRvbF93YXRlci5maWwuVHJlYXRtZW50JGxvZ1RvdGFsQWJ1bmRhbmNlIDwtIGxvZyhhcHBseShkYXQuZG9sX3dhdGVyLmZpbC5UcmVhdG1lbnRbLDU6MTQ3NV0sMSxzdW0pKQ0KDQp6T1RVLndhdGVyLmZpbC5UcmVhdG1lbnQgPC0gbXZhYnVuZChkYXQuZG9sX3dhdGVyLmZpbC5UcmVhdG1lbnRbLDU6MTQ3NV0pDQoNClByZS5maXQxLndhdGVyLmZpbC5UcmVhdG1lbnQgPC0gbWFueWdsbSh6T1RVLndhdGVyLmZpbC5UcmVhdG1lbnQgfiBUcmVhdG1lbnQgKyB0aW1lICsgSUQgKyBvZmZzZXQobG9nVG90YWxBYnVuZGFuY2UpLCBkYXRhID0gZGF0LmRvbF93YXRlci5maWwuVHJlYXRtZW50LCBmYW1pbHk9Im5lZ2F0aXZlLmJpbm9taWFsIikNCg0KI2NoZWNrIGFzc3VtcHRpb25zDQpwbG90KFByZS5maXQxLndhdGVyLmZpbC5UcmVhdG1lbnQpDQoNCiNUaGlzIGlzIHRoZSBhY3R1YWwgc2lnbmlmaWNhbmNlIHRlc3QgICAgICAgICAgICAgICANCmZpdC4xLmRvbC53YXRlci5maWwuVHJlYXRlbWVudCA8LSBtYW55Z2xtKHpPVFUud2F0ZXIuZmlsLlRyZWF0bWVudCB+IFRyZWF0bWVudCArIHRpbWUgKyBJRCArIG9mZnNldChsb2dUb3RhbEFidW5kYW5jZSksIGRhdGEgPSBkYXQuZG9sX3dhdGVyLmZpbC5UcmVhdG1lbnQpDQpmaXQuMi5kb2wud2F0ZXIuZmlsLlRyZWF0ZW1lbnQgPC0gbWFueWdsbSh6T1RVLndhdGVyLmZpbC5UcmVhdG1lbnQgfiB0aW1lICsgSUQgKyBvZmZzZXQobG9nVG90YWxBYnVuZGFuY2UpLCBkYXRhID0gZGF0LmRvbF93YXRlci5maWwuVHJlYXRtZW50KQ0KDQojYWRqdXN0ZWQNCiNnbG9iYWxUZXN0MS5kb2wud2F0ZXIuZmlsLmFkanVzdGVkLlRyZWF0bWVudCA8LSBhbm92YShmaXQuMS5kb2wud2F0ZXIuZmlsLlRyZWF0ZW1lbnQsIGZpdC4yLmRvbC53YXRlci5maWwuVHJlYXRlbWVudCwgbkJvb3Q9MTAwMCwgcC51bmk9J2FkanVzdGVkJykNCg0KI011bHRpdmFyaWF0ZSB0ZXN0Og0KIyAgICAgICAgICAgICAgICAgICAgICAgICAgICAgICBSZXMuRGYgRGYuZGlmZiAgIERldiBQcig+RGV2KQ0KI2ZpdC4xLmRvbC53YXRlci5maWwuVHJlYXRlbWVudCAgICAgNTMgICAgICAgICAgICAgICAgICAgICAgIA0KI2ZpdC4yLmRvbC53YXRlci5maWwuVHJlYXRlbWVudCAgICAgNTMgICAgICAgMCA3LjYwNyAgICAwLjEyMQ0KDQojc2F2ZShnbG9iYWxUZXN0MS5kb2wud2F0ZXIuZmlsLmFkanVzdGVkLlRyZWF0bWVudCwgZmlsZT0nZ2xvYmFsVGVzdDEuZG9sLndhdGVyLmZpbC5hZGp1c3RlZC5UcmVhdG1lbnQuUmRhdGEnKQ0KYGBgDQoNCiMjIyMjIyMjIyMjIyMjIyMjIyMjIyMjIyMjIyMjIyMjIyMjIyMjIyMjIyMjIyMjDQojIyMjIyMjbk1EUyBwbG90IGZvciB0cmVhdGVkIHZzIHVudHJlYXRlZCBkb2xwaGlucw0KYGBge3J9DQojIyNuTURTIHBsb3QgZm9yIEhlYWx0aCB2cyBTaWNrIGRvbHBoaW5zDQojUHJlcGFyZSBkYXRhc2V0IGRhdC5kb2xfd2F0ZXIuZmlsLnQuMzYNCg0KIyMjTWFrZSBjb2xzIDE6NCBtZXRhLWRhdGENCm1ldGFkYXRhX3dhdGVyLmZpbCA8LSBkYXQuZG9sX3dhdGVyLmZpbC50LjM2WywxOjRdDQoNCiMjI0FkZCBjb2x1bW4gJ1RyZWF0bWVudCcgKHNpY2ssIGhlYWx0aHkpIHRvIG1ldGFkYXRhX3dhdGVyLmZpbA0KVHJlYXRtZW50IDwtIGMocmVwKCdoZWFsdGh5JywgMTEpLHJlcCgnc2ljaycsMTQpLHJlcCgnaGVhbHRoeScsIDExKSwgcmVwKCdzaWNrJyw4KSwgcmVwKCdoZWFsdGh5JywgMjgpLCByZXAoJ3NpY2snLDkpKQ0KDQptZXRhZGF0YV93YXRlci5maWwuMiA8LSBjYmluZChUcmVhdG1lbnQsIG1ldGFkYXRhX3dhdGVyLmZpbCkNCg0KZGltKGRhdC5kb2xfd2F0ZXIuZmlsLnQuMzYpDQojODEgMTQ3Ng0KDQpyb3duYW1lcyhkYXQuZG9sX3dhdGVyLmZpbC50LjM2KSA8LSBkYXQuZG9sX3dhdGVyLmZpbC50LjM2JERvbHBoaW4NCmRhdC5kb2xfd2F0ZXIuZmlsLnQuMzcgPC0gZGF0LmRvbF93YXRlci5maWwudC4zNlssLWMoMTo0LCAxNDc2KV0NCg0KIyMjVHVybiBkYXQuZG9sX3dhdGVyLmZpbC50LjM3IGludG8gcmVsIGFidW5kDQpkYXQuZG9sX3dhdGVyLmZpbC50LjM3LnJlbC5hYnVuZCA8LSBkYXQuZG9sX3dhdGVyLmZpbC50LjM3L3Jvd1N1bXMoZGF0LmRvbF93YXRlci5maWwudC4zNykNCg0KI0NvbnZlcnQgdG8gbG9nDQpkYXQuZG9sX3dhdGVyLmZpbC50LjM3LnJlbC5hYnVuZC5sb2cgPC0gbG9nKGRhdC5kb2xfd2F0ZXIuZmlsLnQuMzcucmVsLmFidW5kICsxKQ0KDQojQnJheS1DdXJ0aXMgZGlzc2ltaWxhcml0eSBtYXRyaXggd2l0aCBsb2cNCmRhdC5kb2xfd2F0ZXIuZmlsLnQuMzcucmVsLmFidW5kLmxvZy5iYyA8LSB2ZWdkaXN0KGRhdC5kb2xfd2F0ZXIuZmlsLnQuMzcucmVsLmFidW5kLmxvZywgbWV0aG9kID0gImJyYXkiKQ0KDQojIyMjbk1EUw0KIyBubWRzIHBsb3Qgd2l0aCBkb3RzIG9mIDIgY29sb3VycywgKHN0cmVzcyBzaG91bGQgYmUgPDAuMikNCmRhdC5kb2xfd2F0ZXIuZmlsLnQuMzcucmVsLmFidW5kLmxvZy5oY2x1cy5tZHMgPC0gbWV0YU1EUyhkYXQuZG9sX3dhdGVyLmZpbC50LjM3LnJlbC5hYnVuZC5sb2cuYmMsIGF1dG90cmFuc2Zvcm0gPSBGLCB0cmFjZSA9IEYsIHRyeW1heD01MCkNCmRhdC5kb2xfd2F0ZXIuZmlsLnQuMzcucmVsLmFidW5kLmxvZy5oY2x1cy5tZHMgIyBzdHJlc3MgPSAwLjE4ODAyNTgNCg0KIyMjQ3JlYXRlIHBsb3RkYXRhDQpwbG90RGF0YS5kb2xwaGluLndhdGVyLmZpbC5BQiA8LSBkYXRhLmZyYW1lKGRhdC5kb2xfd2F0ZXIuZmlsLnQuMzcucmVsLmFidW5kLmxvZy5oY2x1cy5tZHMkcG9pbnRzLG1ldGFkYXRhX3dhdGVyLmZpbC4yJFRyZWF0bWVudCkNCm5hbWVzKHBsb3REYXRhLmRvbHBoaW4ud2F0ZXIuZmlsLkFCKSA9IGMoIngiLCJ5IiwiSUQiKQ0KDQojcGxvdERhdGEuZG9scGhpbi53YXRlci5maWwuQUIucmVtIDwtIHBsb3REYXRhLmRvbHBoaW4ud2F0ZXIuZmlsLkFCW3Bsb3REYXRhLmRvbHBoaW4ud2F0ZXIuZmlsLkFCJHggPCAwLjIsXQ0KDQpuTURTLndhdGVyLmZpbC5BQiA8LSANCiAgZ2dwbG90KHBsb3REYXRhLmRvbHBoaW4ud2F0ZXIuZmlsLkFCLCBhZXMoeCx5LGNvbG91cj1JRCkpICsgZ2VvbV9wb2ludCgpICsNCiAgdGhlbWVfYncoKSArDQogIHRoZW1lKHBsb3QudGl0bGUgPSBlbGVtZW50X3RleHQoc2l6ZSA9IDE0LCBmYW1pbHkgPSAiVGFob21hIiwgZmFjZSA9ICJib2xkIiksDQogICAgICAgIHRleHQgPSBlbGVtZW50X3RleHQoc2l6ZSA9IDEyLCBmYW1pbHkgPSAiVGFob21hIiksDQogICAgICAgIGF4aXMudGl0bGUgPSBlbGVtZW50X3RleHQoZmFjZT0iYm9sZCIpLA0KICAgICAgICBheGlzLnRleHQueD1lbGVtZW50X3RleHQoc2l6ZSA9IDExKSkgKw0KICAgICAgICBzY2FsZV9maWxsX2JyZXdlcihwYWxldHRlID0gIkFjY2VudCIpICsgDQogIGdlb21fdGV4dChsYWJlbCA9IHJvd25hbWVzKGRhdC5kb2xfd2F0ZXIuZmlsLnQuMzcucmVsLmFidW5kLmxvZykpICsNCiAgeGxhYigibk1EUyAxIikgKyB5bGFiKCJuTURTIDIiKQ0KDQpnZ3NhdmUoIm5NRFMud2F0ZXIuZmlsLkFCLm5hbWVzLmpwZyIsIHBsb3QgPSBuTURTLndhdGVyLmZpbC5BQiwgZGV2aWNlID0gJ2pwZycsIHdpZHRoID0gMTY4LCBoZWlnaHQgPSAxMDAsIHVuaXRzID0gIm1tIiwNCiAgICAgICBkcGkgPSAzMDAsIGxpbWl0c2l6ZSA9IFRSVUUpDQoNCiMjI1dpdGhvdXQgbmFtZXMNCm5NRFMud2F0ZXIuZmlsLkFCLm5vTmFtZXMgPC0gDQogIGdncGxvdChwbG90RGF0YS5kb2xwaGluLndhdGVyLmZpbC5BQiwgYWVzKHgseSxjb2xvdXI9SUQpKSArIGdlb21fcG9pbnQoKSArDQogIHRoZW1lX2J3KCkgKw0KICB0aGVtZShwbG90LnRpdGxlID0gZWxlbWVudF90ZXh0KHNpemUgPSAxNCwgZmFtaWx5ID0gIlRhaG9tYSIsIGZhY2UgPSAiYm9sZCIpLA0KICAgICAgICB0ZXh0ID0gZWxlbWVudF90ZXh0KHNpemUgPSAxMiwgZmFtaWx5ID0gIlRhaG9tYSIpLA0KICAgICAgICBheGlzLnRpdGxlID0gZWxlbWVudF90ZXh0KGZhY2U9ImJvbGQiKSwNCiAgICAgICAgYXhpcy50ZXh0Lng9ZWxlbWVudF90ZXh0KHNpemUgPSAxMSkpICsNCiAgc2NhbGVfZmlsbF9icmV3ZXIocGFsZXR0ZSA9ICJBY2NlbnQiKSArDQogIHhsYWIoIm5NRFMgMSIpICsgeWxhYigibk1EUyAyIikNCg0KZ2dzYXZlKCJuTURTLndhdGVyLmZpbC5BQi5ub05hbWVzLmpwZyIsIHBsb3QgPSBuTURTLndhdGVyLmZpbC5BQi5ub05hbWVzLCBkZXZpY2UgPSAnanBnJywgd2lkdGggPSAxNjgsIGhlaWdodCA9IDEwMCwgdW5pdHMgPSAibW0iLA0KICAgICAgIGRwaSA9IDMwMCwgbGltaXRzaXplID0gVFJVRSkNCmBgYA0KDQojIyMjIyMjIyMjIyMjIyMjIyMjIyMjIyMjIyMjIyMjIyMjIyMjIyMjIyMjIyMjIw0KIyMjIyMjI25NRFMgcGxvdCBmb3IgdHJlYXRlZCB2cyB1bnRyZWF0ZWQgZG9scGhpbnM6IFNob3cgYWxsIGxldmVscyANCmBgYHtyfQ0KIyMjbk1EUyBwbG90IGZvciBIZWFsdGggdnMgU2ljayBkb2xwaGlucw0KI1ByZXBhcmUgZGF0YXNldCBkYXQuZG9sX3dhdGVyLmZpbC50LjM2DQoNCiNtZXRhZGF0YV93YXRlci5maWwuMg0KI2RhdC5kb2xfd2F0ZXIuZmlsLnQuMzYNCg0KIyMjQ3JlYXRlIHBsb3RkYXRhDQpwbG90RGF0YS5kb2xwaGluLndhdGVyLmZpbC5BQi5hbGxMIDwtIGRhdGEuZnJhbWUoZGF0LmRvbF93YXRlci5maWwudC4zNy5yZWwuYWJ1bmQubG9nLmhjbHVzLm1kcyRwb2ludHMsbWV0YWRhdGFfd2F0ZXIuZmlsLjIkQUIuVHJlYXRtZW50LmFsbC5kb2wpDQpuYW1lcyhwbG90RGF0YS5kb2xwaGluLndhdGVyLmZpbC5BQi5hbGxMKSA9IGMoIngiLCJ5IiwiSUQiKQ0KDQojcGxvdERhdGEuZG9scGhpbi53YXRlci5maWwuQUIucmVtIDwtIHBsb3REYXRhLmRvbHBoaW4ud2F0ZXIuZmlsLkFCW3Bsb3REYXRhLmRvbHBoaW4ud2F0ZXIuZmlsLkFCJHggPCAwLjIsXQ0KDQpuTURTLndhdGVyLmZpbC5BQi5hbGxMIDwtIA0KICBnZ3Bsb3QocGxvdERhdGEuZG9scGhpbi53YXRlci5maWwuQUIuYWxsTCwgYWVzKHgseSxjb2xvdXI9SUQpKSArIGdlb21fcG9pbnQoKSArDQogIHRoZW1lX2J3KCkgKw0KICB0aGVtZShwbG90LnRpdGxlID0gZWxlbWVudF90ZXh0KHNpemUgPSAxNCwgZmFtaWx5ID0gIlRhaG9tYSIsIGZhY2UgPSAiY
[truncated: 75,959 more chars]
