# Supplementary material for: Highly abundant core taxa in the blow within and across captive bottlenose dolphins provide evidence for a temporally stable airway microbiota
Source: BMC Microbiol. 2021 Jan 9;21:20. doi: 10.1186/s12866-020-02076-z (PMC7796641; doi:10.1186/s12866-020-02076-z)
Supplement: Supplementary file 1 — Additional file 1: Fig. S1. Shows a scatterplot of the technical contaminant zOTUs of control samples (‘TRUE’ in green) and dolphins blow zOTUs (‘FALSE’ in red). The R package decontam determined 157 technical contaminants which were then deleted from the 81 dolphin blow samples. Fig. S2. Shows a histogram of the technical contaminant zOTUs of control samples (bars on the left) and dolphins blow zOTUs (bars on the right). The R package decontam determined 157 technical contaminants which were then deleted from the 81 dolphin blow samples. The figure shows the bimodal division between dolphin zOTUs and technical control zOTUs. Fig. S3. Rarefaction curves of dolphin blow samples. The majority of samples was sampled to saturation. Fig. S4. Rarefaction curves of pool water samples. The majority of samples was sampled to saturation. Fig. S5. Shows a scatterplot of the contaminant zOTUs of pool water samples (‘TRUE’ in green) and dolphins blow zOTUs (‘FALSE’ in red). The R package decontam determined 520 water contaminants which were then deleted from the 81 dolphin blow samples. Fig. S6. Shows a histogram of the water contaminant zOTUs of pool water samples (bars on the left) and dolphins blow zOTUs (bars on the right). The R package decontam determined 520 water contaminants which were then deleted from the 81 dolphin blow samples. The figure shows the bimodal division between dolphin zOTUs and water zOTUs. Fig. S7. Shows the alpha diversity parameter, richness, across 37 weeks of sample collection in the 13 study dolphins. Fig. S8. Shows the alpha diversity parameter, Shannon-Wiener diversity, across 37 weeks of sample collection in the 13 study dolphins. Fig. S9. Shows the alpha diversity parameter, Chao1, across 37 weeks of sample collection in the 13 study dolphins. Fig. S10. Shows the alpha diversity parameter, ACE, across 37 weeks of sample collection in the 13 study dolphins. Fig. S11. nMDS plot based on Bray-Curtis dissimilarity matrix of 81 dolphin blow and 28 [file 12866_2020_2076_MOESM1_ESM.zip › Supplements_ManuscriptDolphins_2ndRevision_clean.docx]

**Supplementary information**

**Highly abundant core taxa in the blow within and across captive bottlenose dolphins provide evidence for a temporally stable airway microbiota**

Catharina Vendl^1^, Tiffanie Nelson^2^, Belinda Ferrari^3^, Torsten Thomas^4^, Tracey Rogers^1^

^1^ Evolution and Ecology Research Centre, School of Biological, Earth and Environmental Sciences, University of New South Wales, Sydney, NSW, 2052, Australia.

^2^ Queensland Facility for Advanced Bioinformatics, Griffith University, Gold Coast, Southport, QLD, 4215, Australia.

^3^ School of Biotechnology and Biomolecular Sciences, University of New South Wales, Sydney, NSW, 2052, Australia.

^4^ Centre for Marine Science and Innovation, School of Biological, Earth and Environmental Sciences, University of New South Wales, Sydney, NSW, 2052, Australia.

*Corresponding: [c.vendl@unsw.edu.au](mailto:c.vendl@unsw.edu.au)

**Supplementary figures**


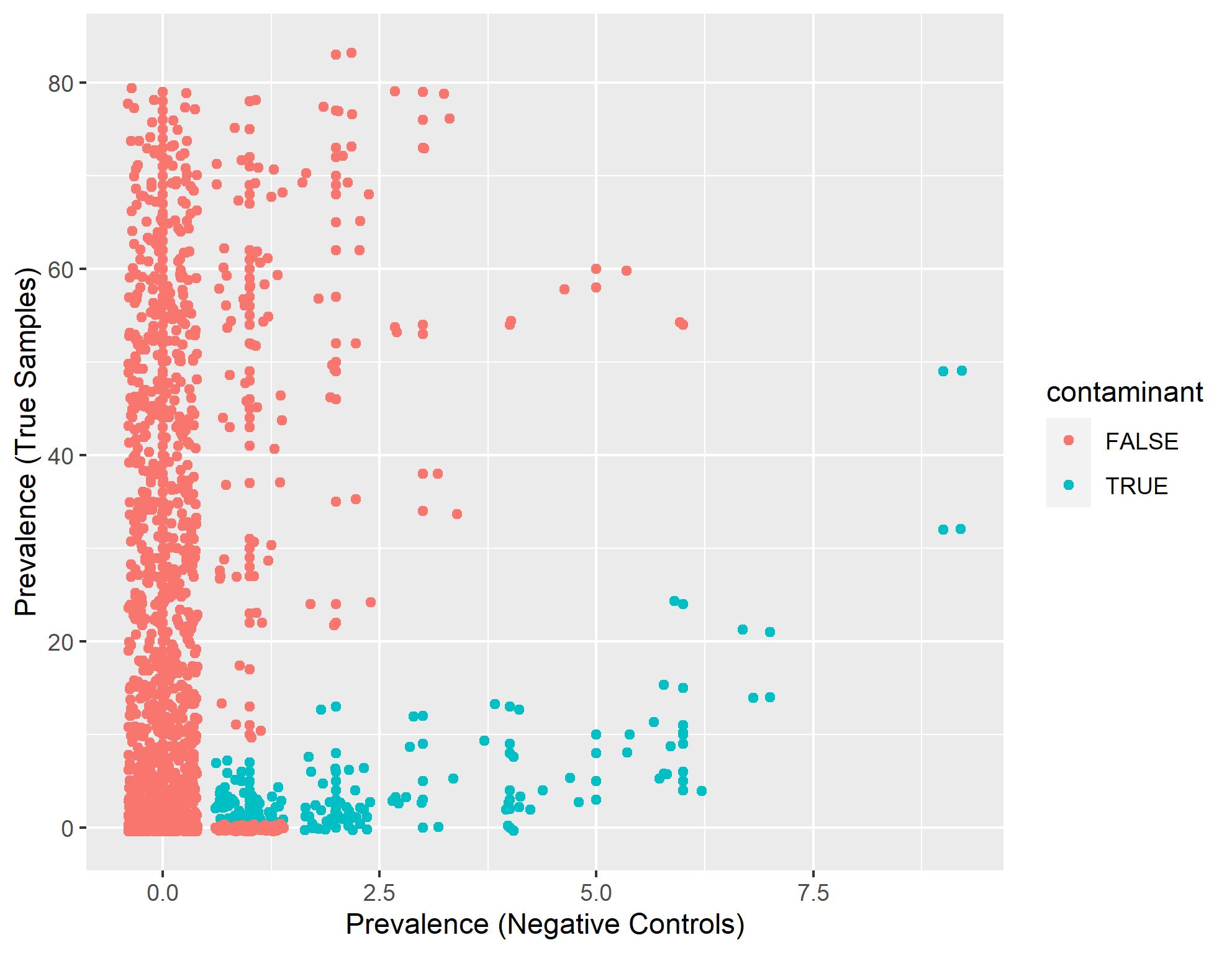
**FIG S1 shows a scatterplot of the technical contaminant zOTUs of control samples (‘TRUE’ in green) and dolphins blow zOTUs (‘FALSE’ in red).** The R package *decontam* determined 157 technical contaminants which were then deleted from the 81 dolphin blow samples.


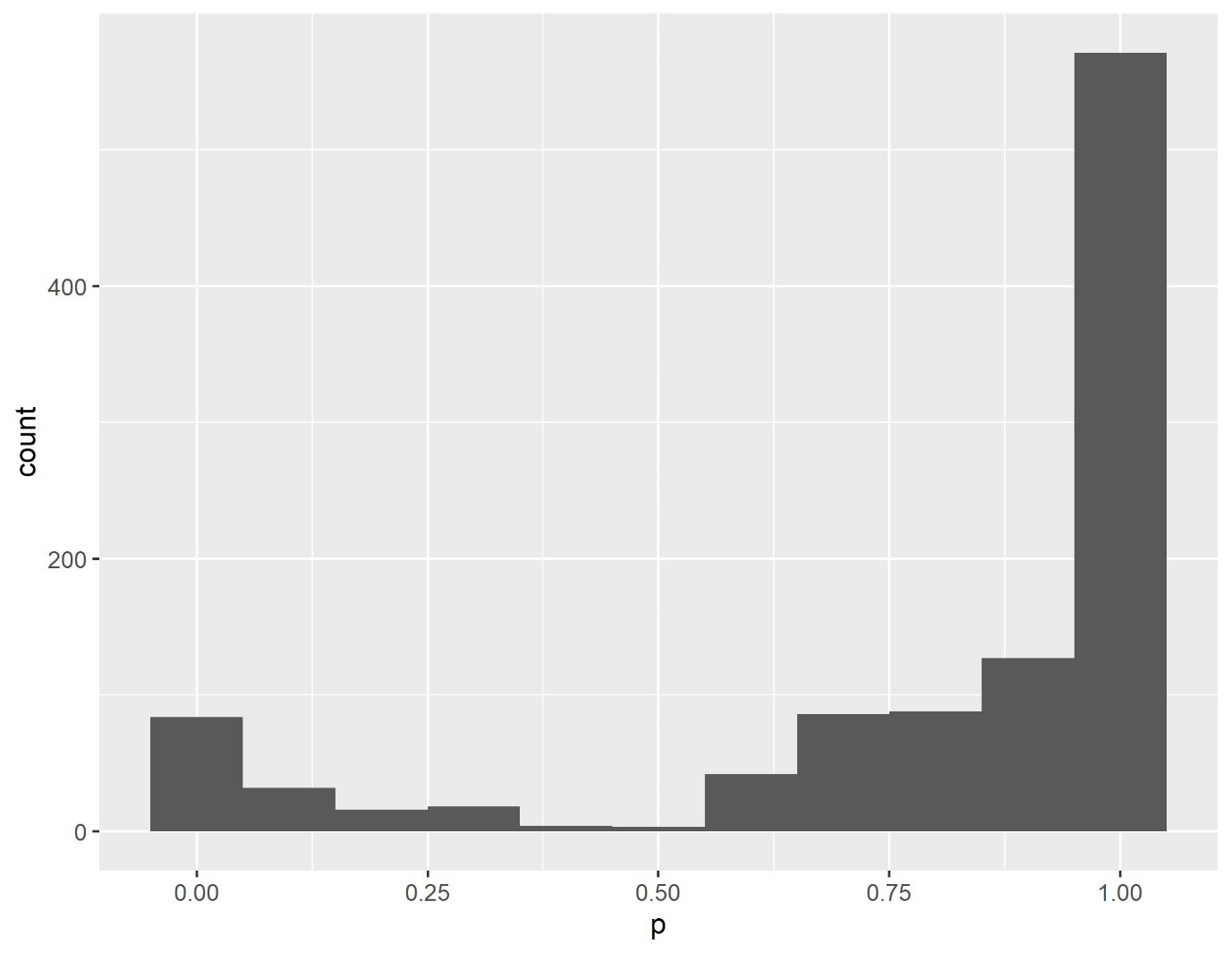
**FIG S2 shows a histogram of the technical contaminant zOTUs of control samples (bars on the left) and dolphins blow zOTUs (bars on the right).** The R package *decontam* determined 157 technical contaminants which were then deleted from the 81 dolphin blow samples. The figure shows the bimodal division between dolphin zOTUs and technical control zOTUs.

| 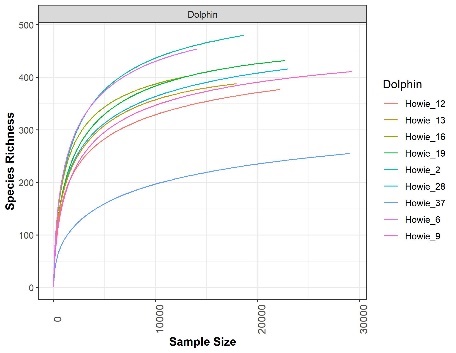 | 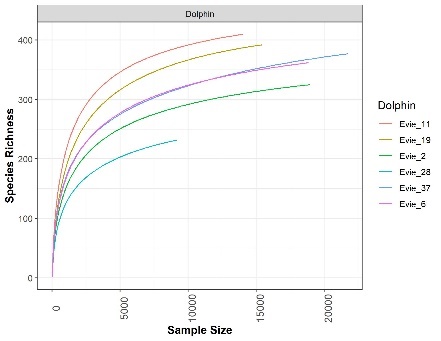 | 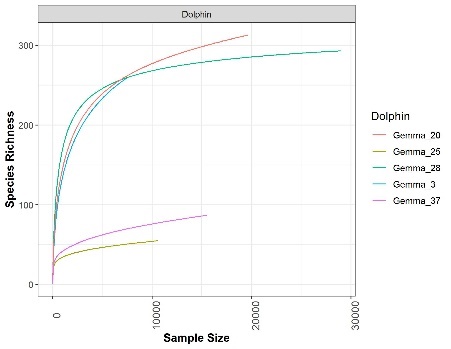 |
| --- | --- | --- |
| 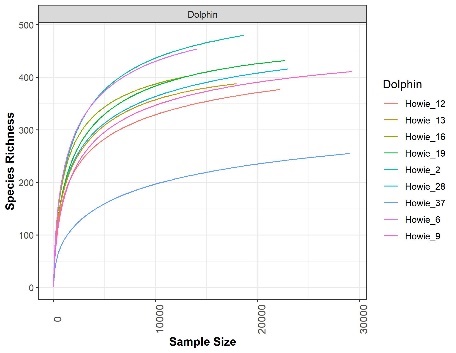 | **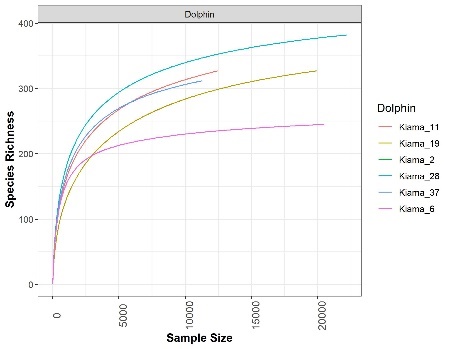** | **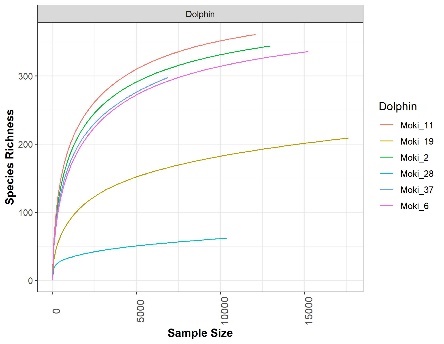** |
| 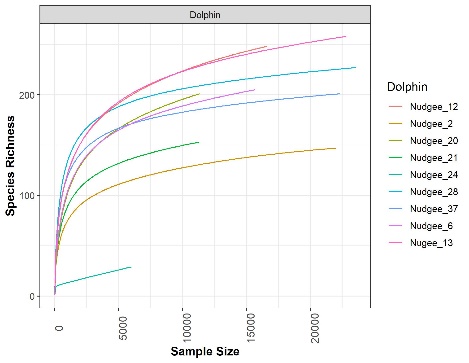 | 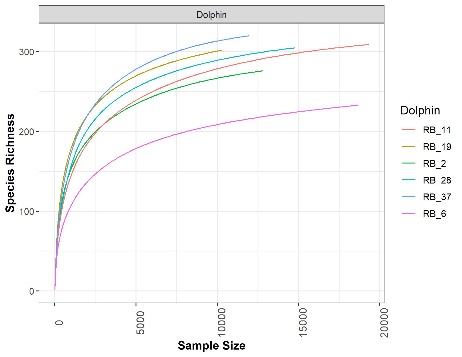 | 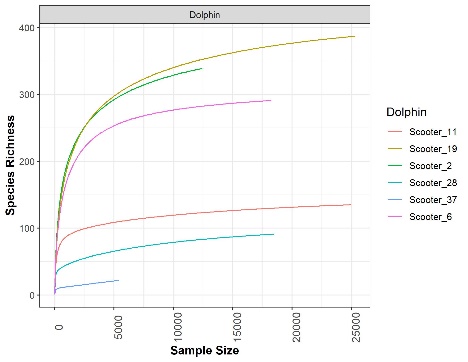 |
| 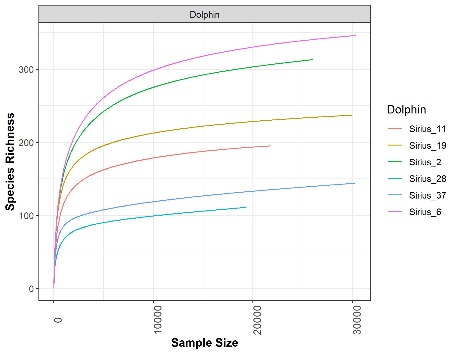 | 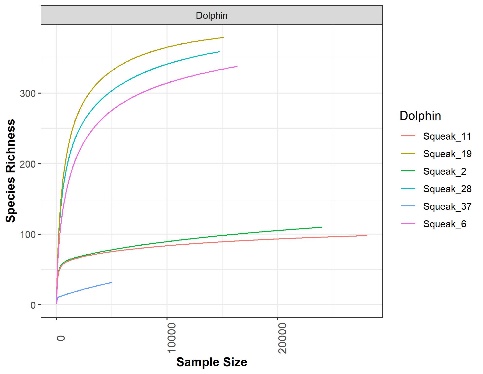 | 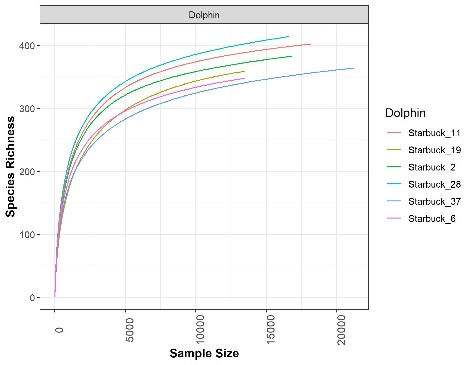 |
| 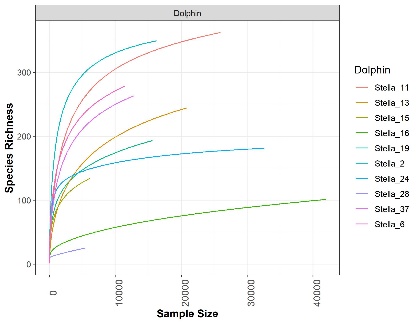 |  |  |

**FIG S3 Rarefaction curves of dolphin blow samples.** The majority of samples was sampled to saturation.

| 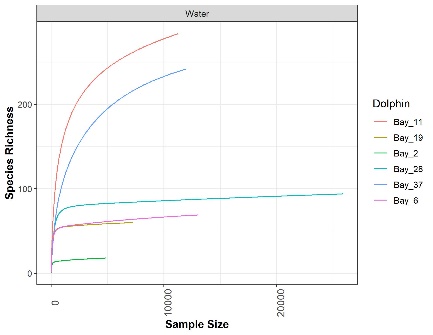 | 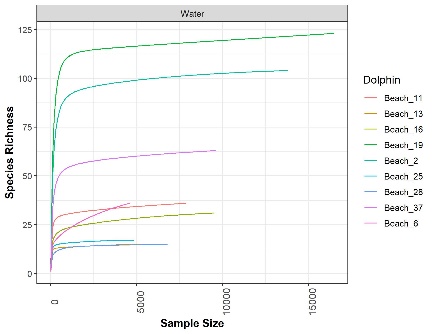 | 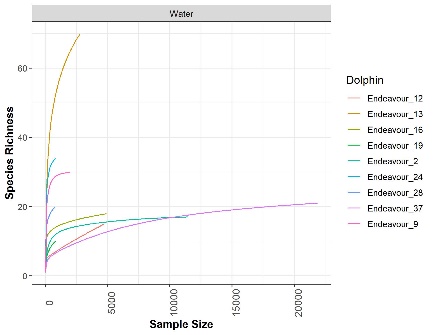 |
| --- | --- | --- |
| 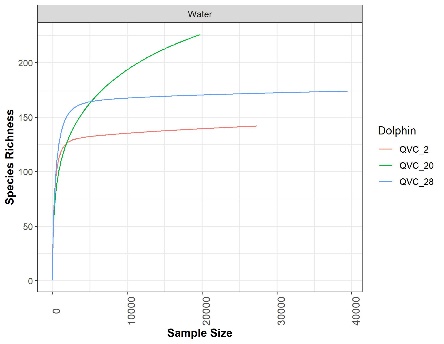 |  |  |

**FIG S4 Rarefaction curves of pool water samples.** The majority of samples was sampled to saturation.


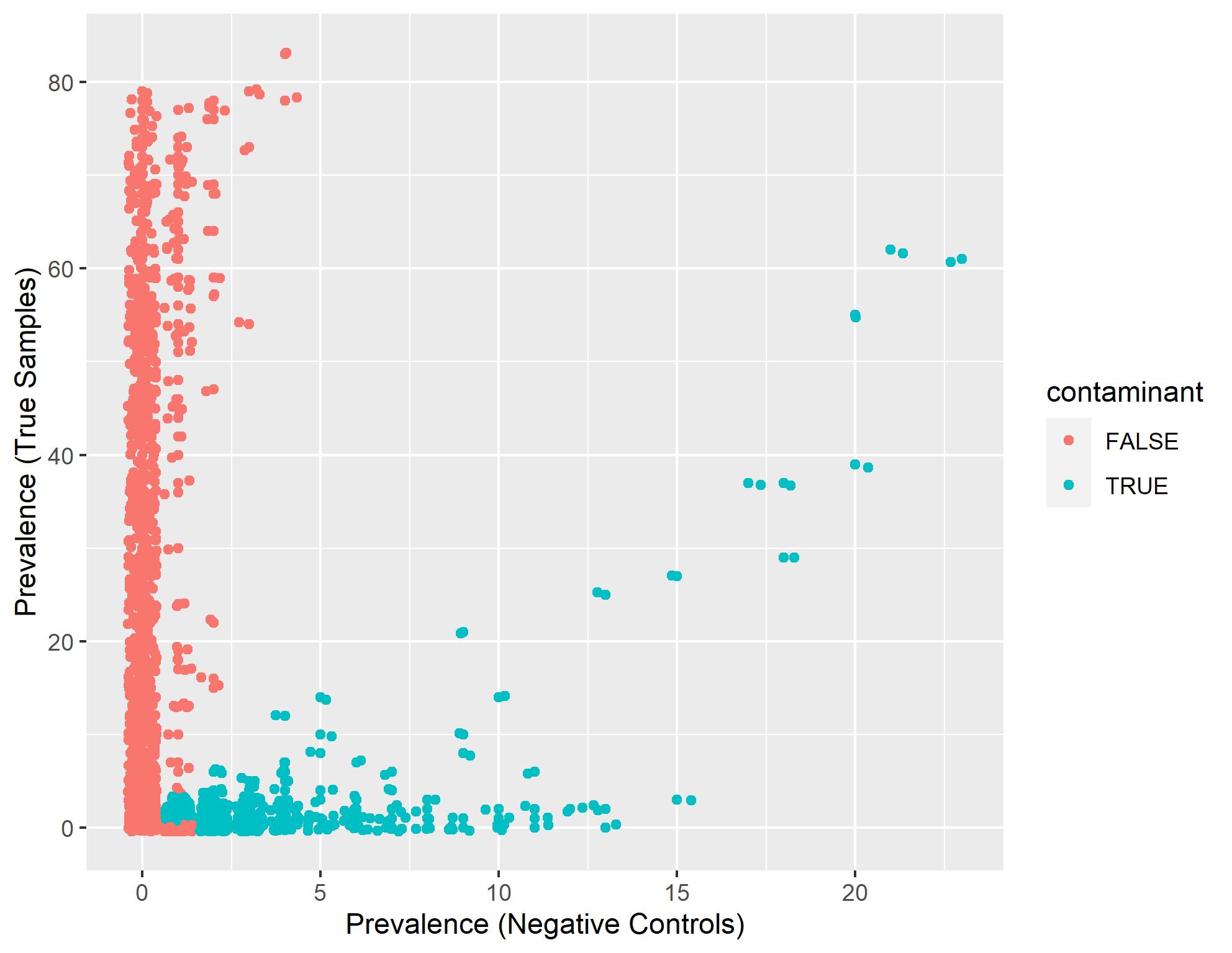
**FIG S5 shows a scatterplot of the contaminant zOTUs of pool water samples (‘TRUE’ in green) and dolphins blow zOTUs (‘FALSE’ in red).** The R package *decontam* determined 520 water contaminants which were then deleted from the 81 dolphin blow samples.

**
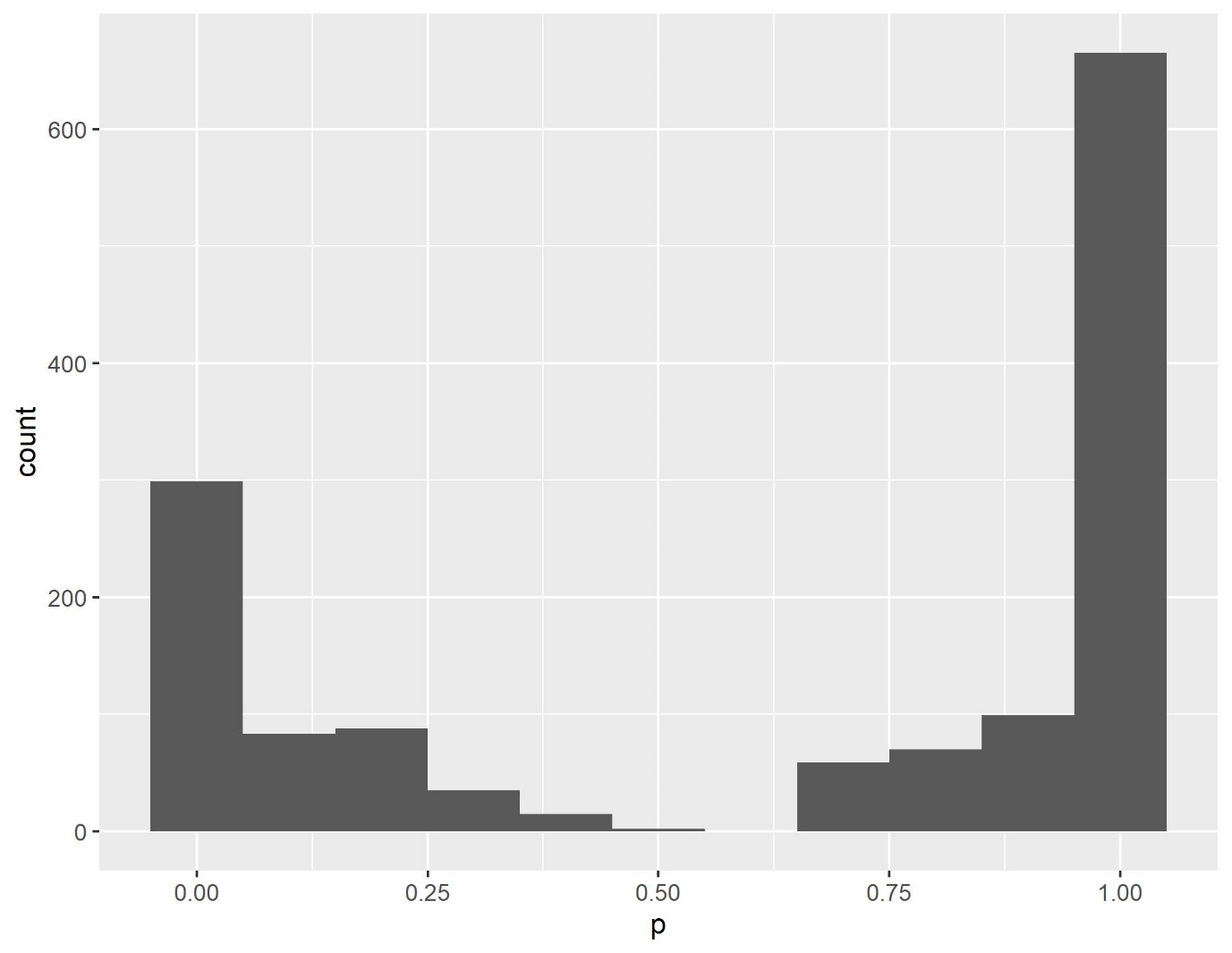
FIG S6 shows a histogram of the water contaminant zOTUs of pool water samples (bars on the left) and dolphins blow zOTUs (bars on the right).** The R package *decontam* determined 520 water contaminants which were then deleted from the 81 dolphin blow samples. The figure shows the bimodal division between dolphin zOTUs and water zOTUs.


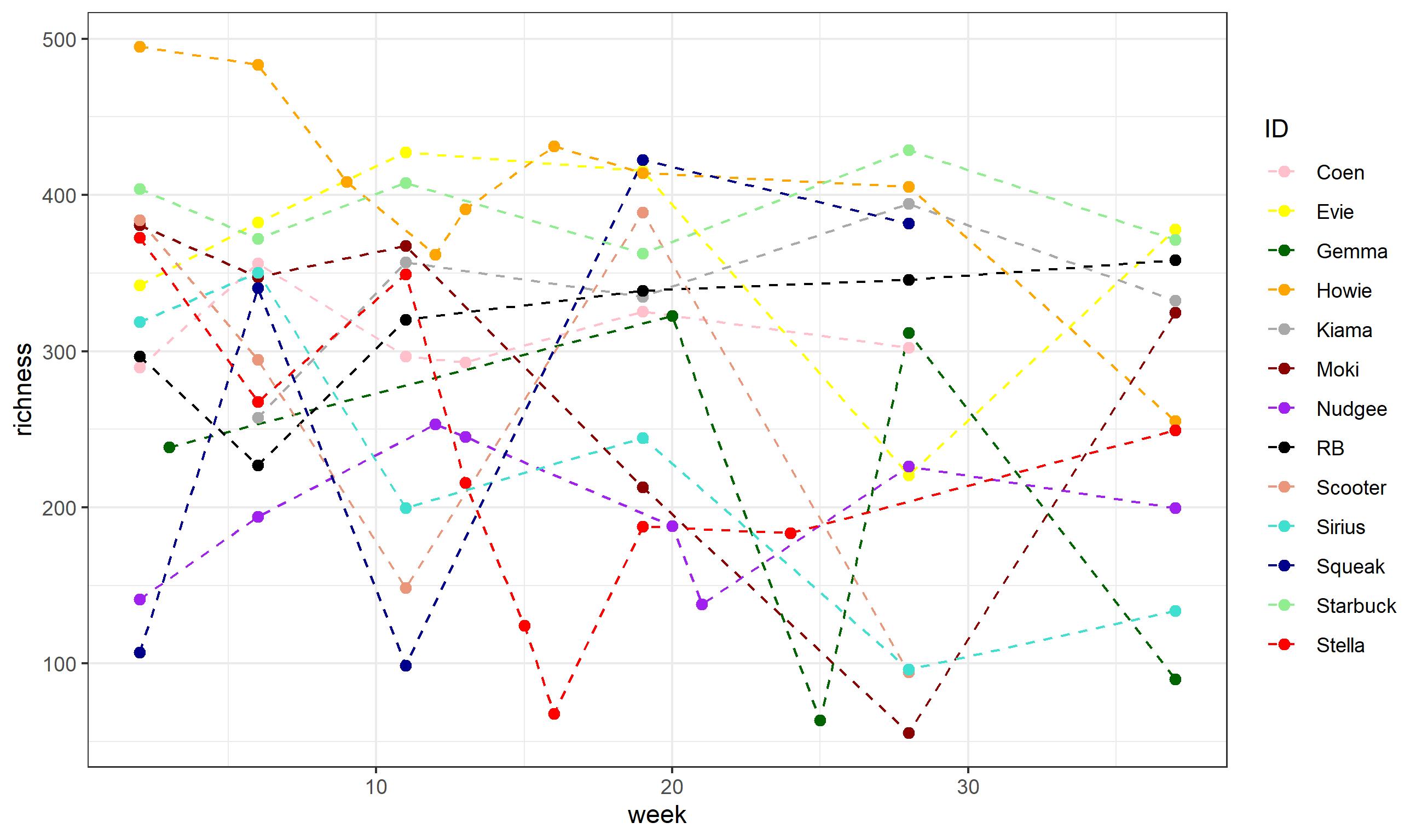


**FIG S7 shows the alpha diversity parameter, richness, across 37 weeks of sample collection in the 13 study dolphins.**


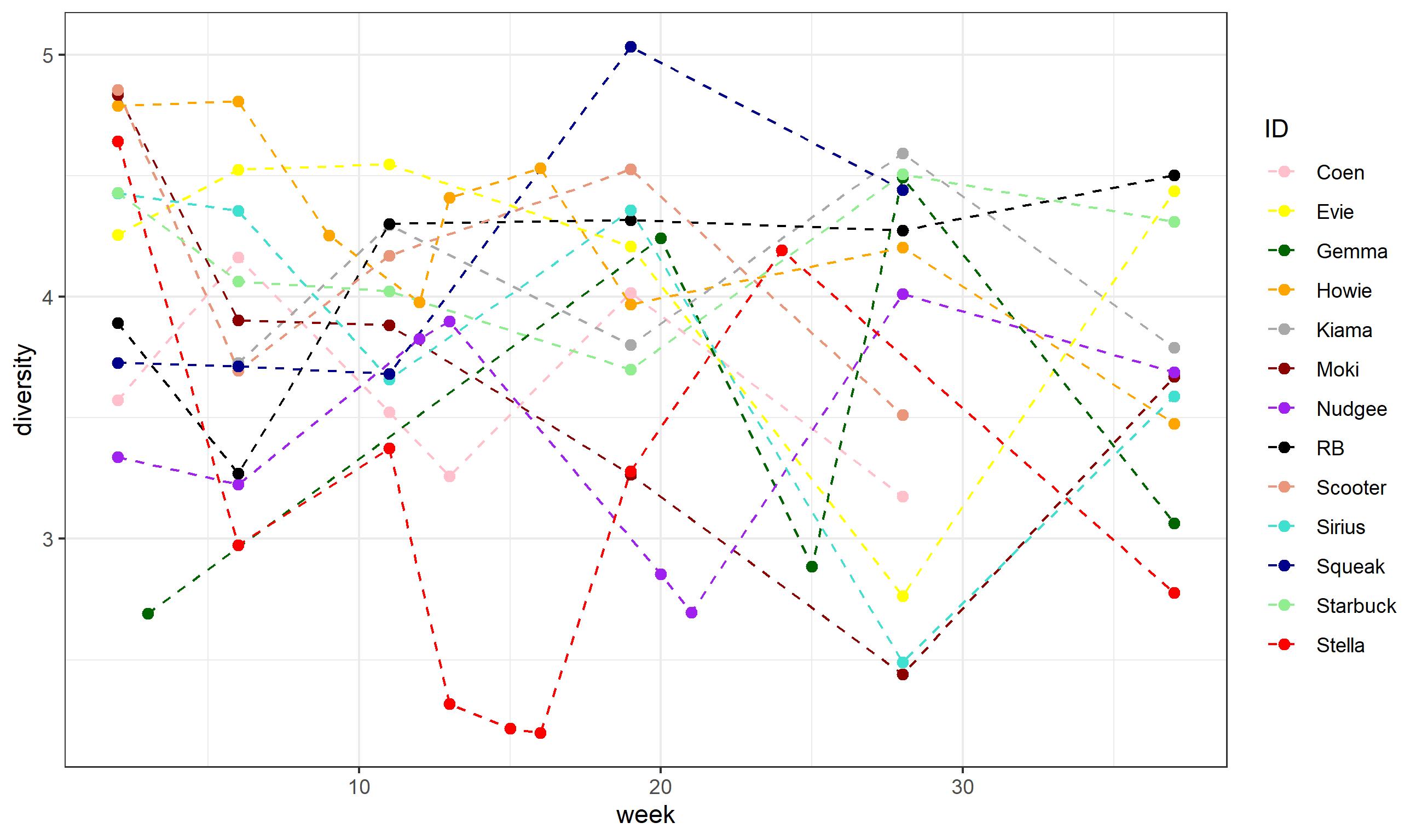
**FIG S8 shows the alpha diversity parameter, Shannon-Wiener diversity, across 37 weeks of sample collection in the 13 study dolphins.**


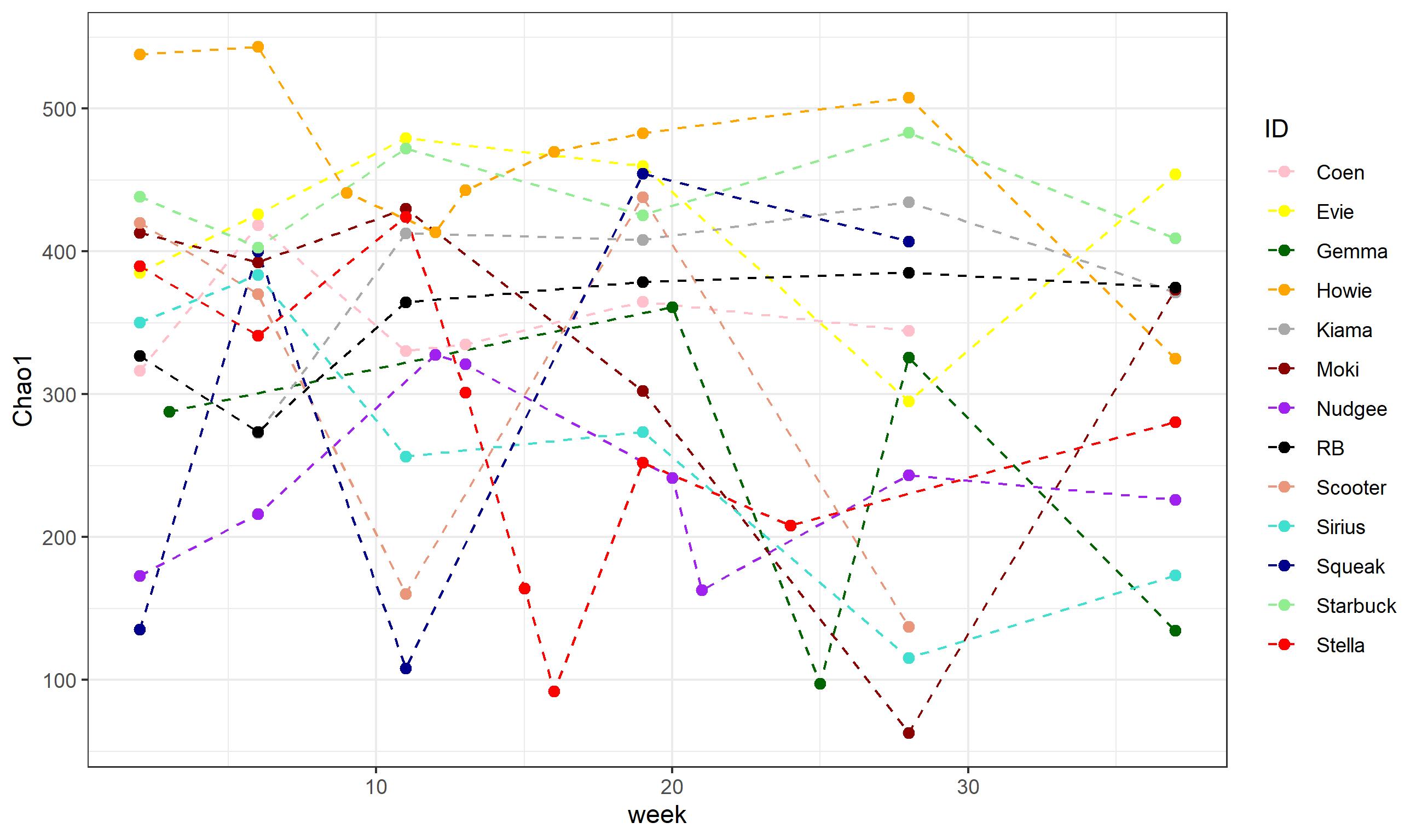


**FIG S9 shows the alpha diversity parameter, Chao1, across 37 weeks of sample collection in the 13 study dolphins.**


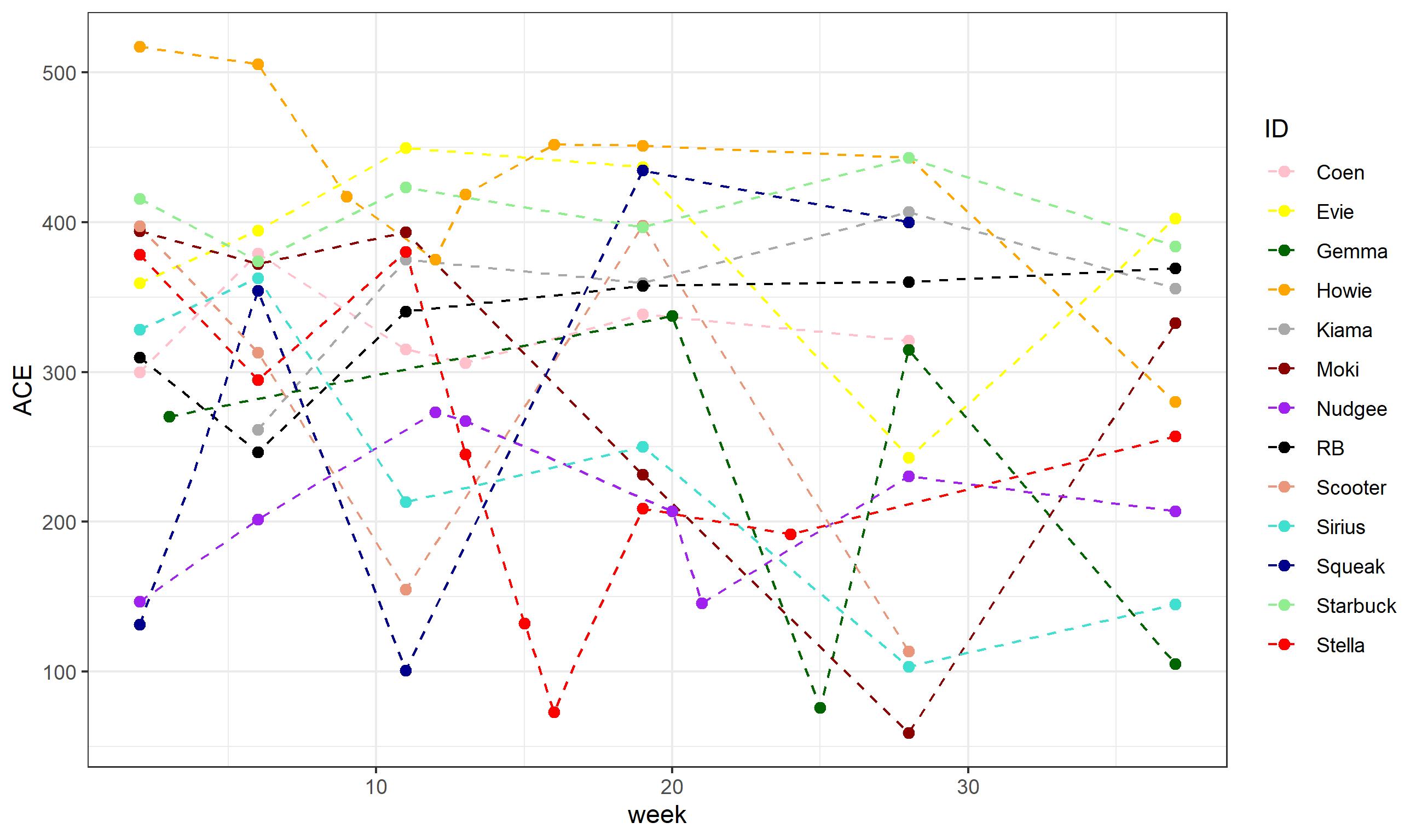


**FIG S10 shows the alpha diversity parameter, ACE, across 37 weeks of sample collection in the 13 study dolphins.**


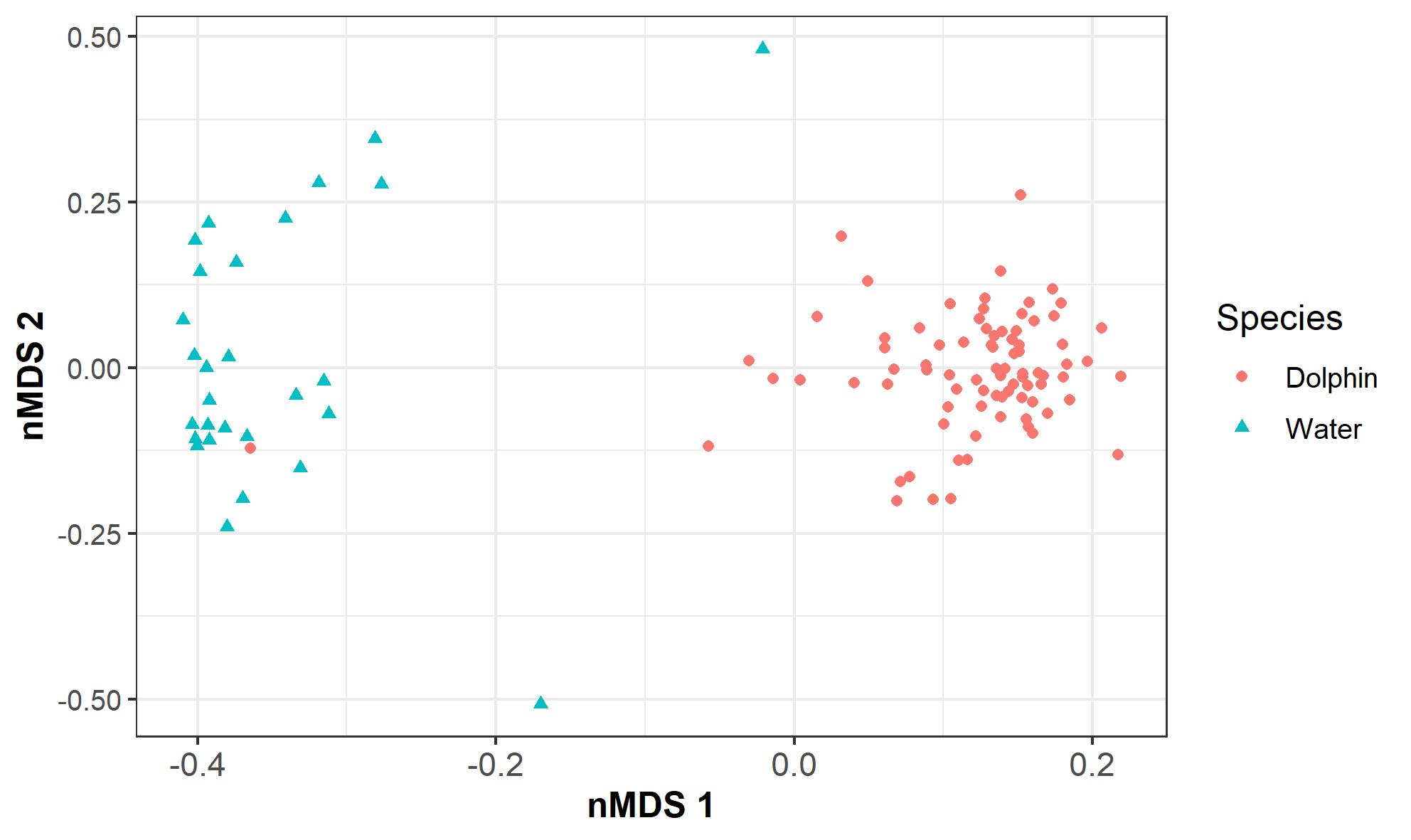


**FIG S11 nMDS plot based on Bray-Curtis dissimilarity matrix of 81 dolphin blow and 28 pool water samples.** A clear distinction between the microbial community composition in dolphin blow and pool water is evident.


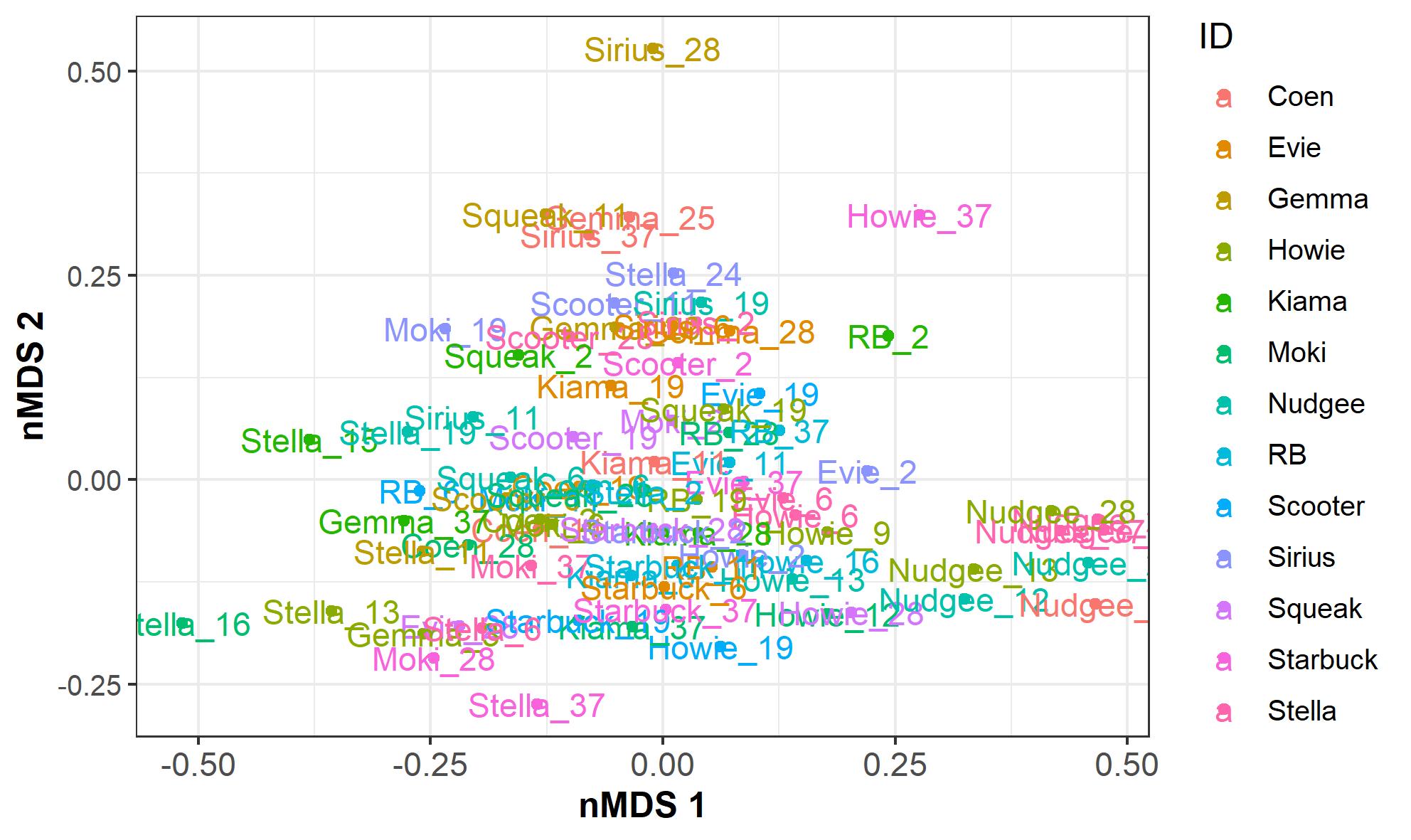


**FIG S12 nMDS plot based on Bray-Curtis dissimilarity matrix of 81 dolphin blow samples coloured according to their ID (individual dolphins).** Although not clearly evident in this plot, the *mvabund*-based analysis showed a significant impact of the factor ‘dolphin ID’.


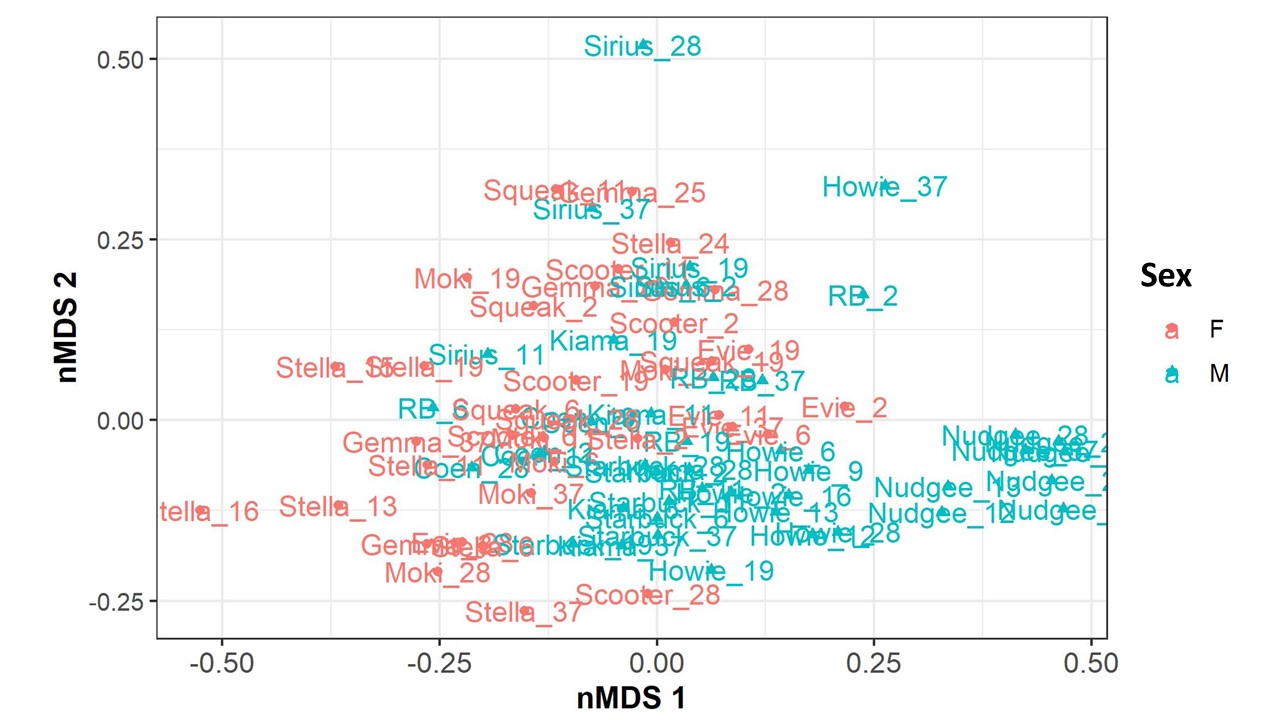


**FIG S13 nMDS plot based on Bray-Curtis dissimilarity matrix of 81 dolphin blow samples coloured according to the dolphins’ sex.** The diagram provides a hint that the *mvabund*-based analysis showed a significant impact of the factor ‘sex’ on the microbial communities


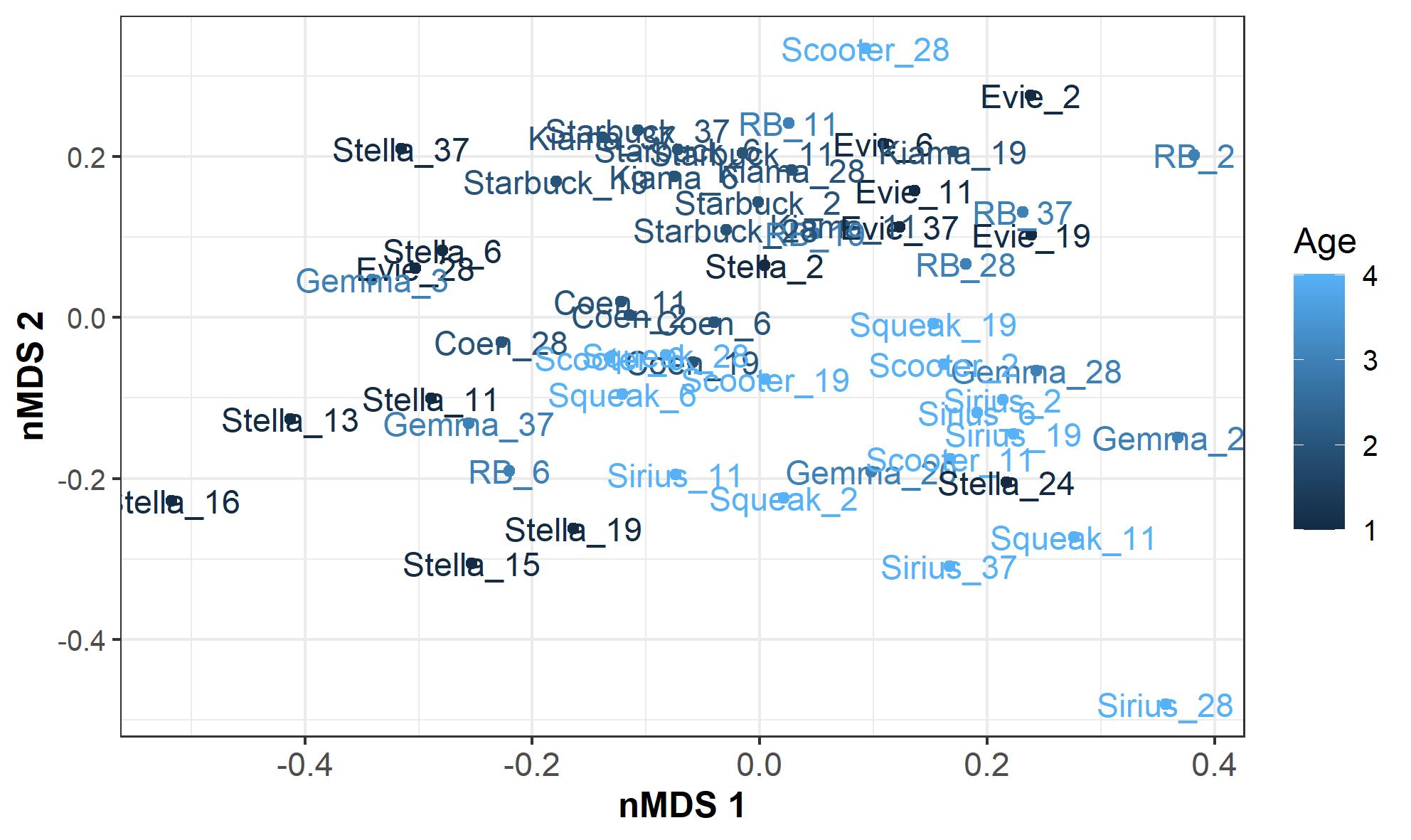


**FIG S14 nMDS plot based on Bray-Curtis dissimilarity matrix of 81 dolphin blow samples coloured according to the dolphins’ age groups.** The diagram provides some evidence that the *mvabund*-based analysis showed a significant impact of the factor ‘age’ on the microbial communities. The analysis was based on four age levels: 1: 0 – 10 years, 2: 11 – 20 years, 3: 21 – 30 years, 4: 31 – 40 years.


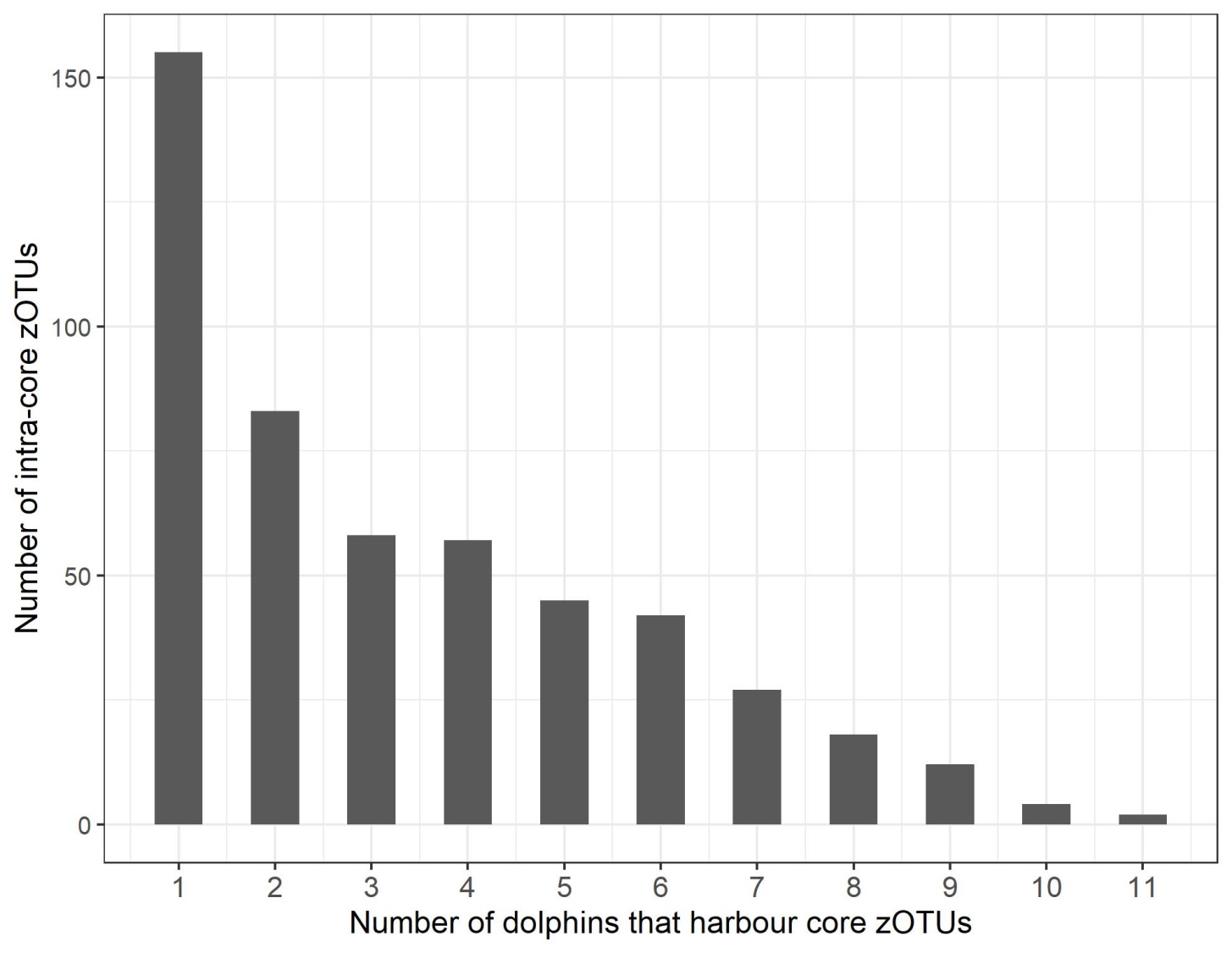


**FIG S15 Frequency histogram showing the presence of 503 intra-core zOTUs across 11 bottlenose dolphins.** Although a large number of intra-core zOTUs is present, only a minority is shared by most dolphins


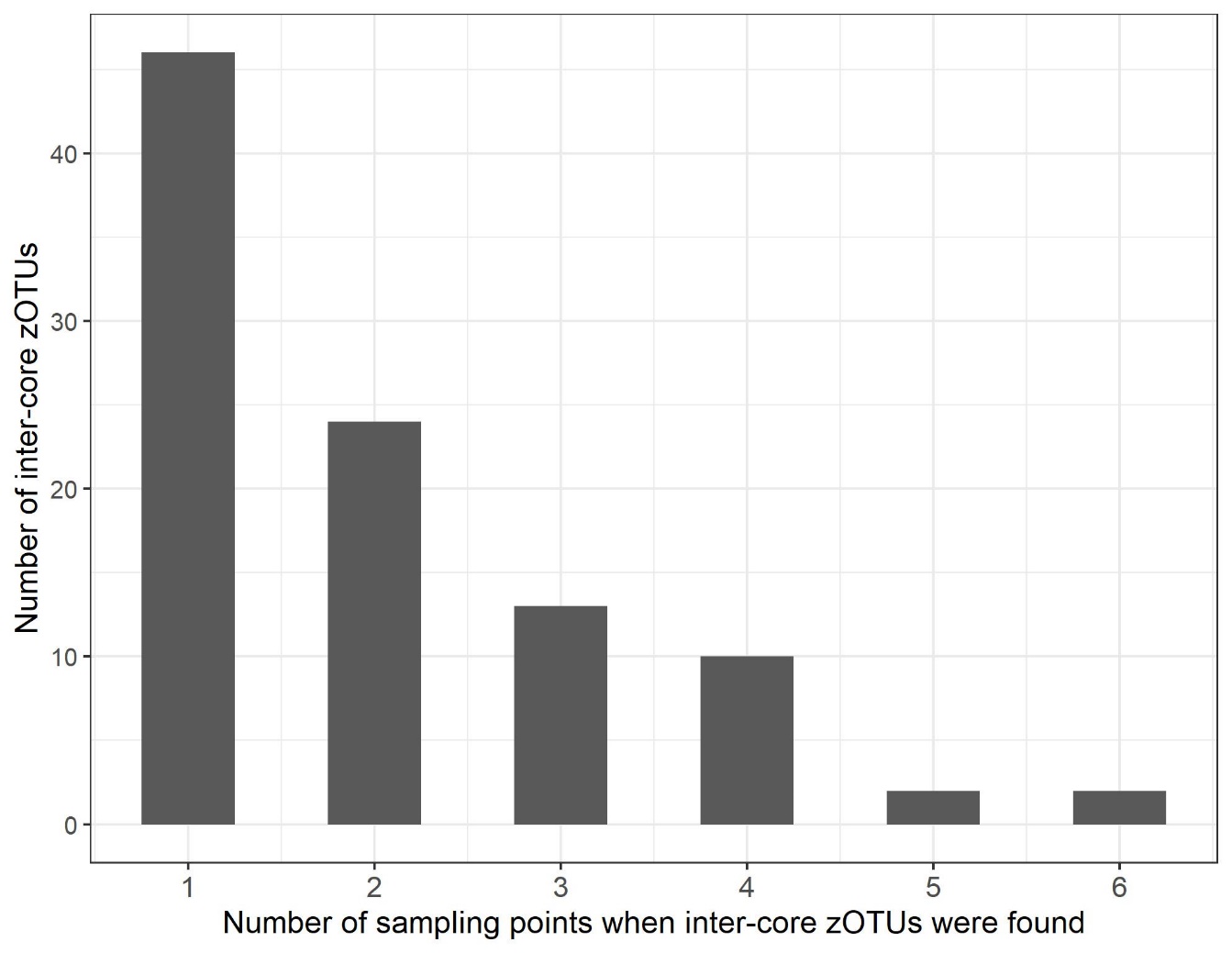


**FIG S16 Frequency histogram showing the presence of 97 inter-core zOTUs across ten dolphins** in week 2, 6, 11, 19, 28, 37. More than half of the inter-core zOTUs were only present at one sampling point.

**
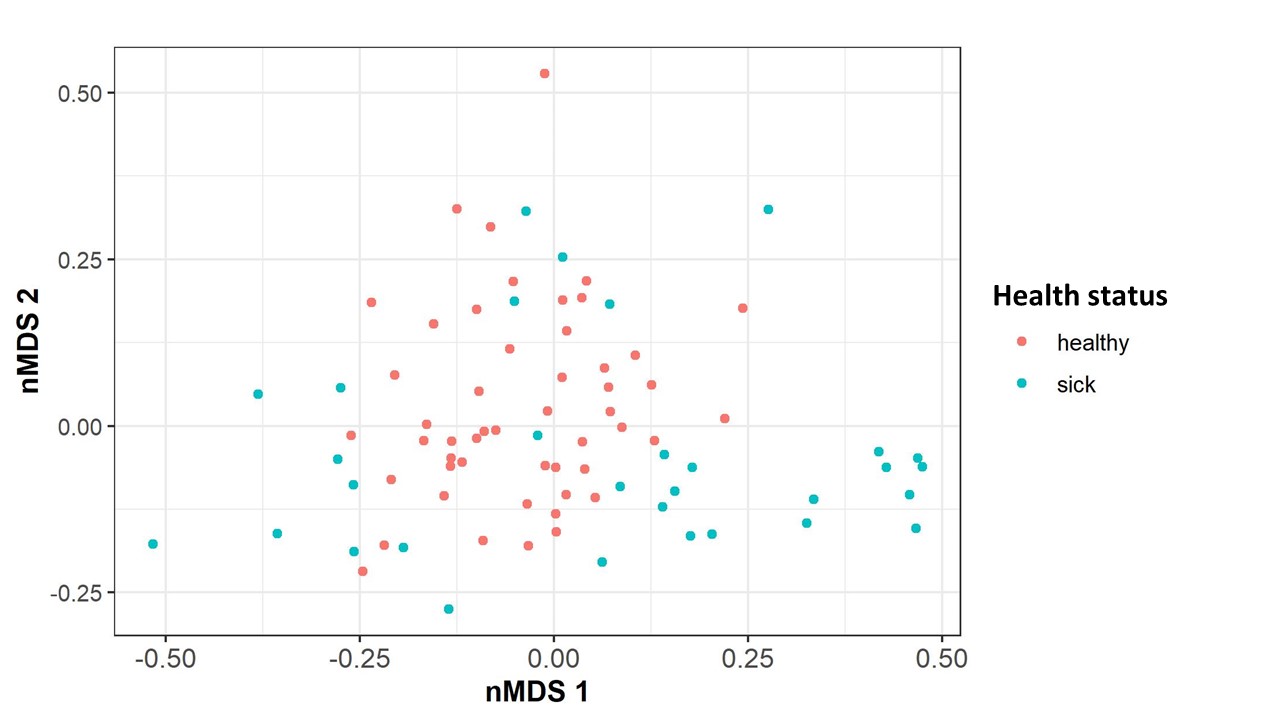
****FIG S17 nMDS plot based on Bray-Curtis dissimilarity matrix of 81 dolphin blow samples coloured according to their health status.** The mvabund-based analysis did not provide evidence for a general difference between healthy/untreated and sick/treated dolphin.


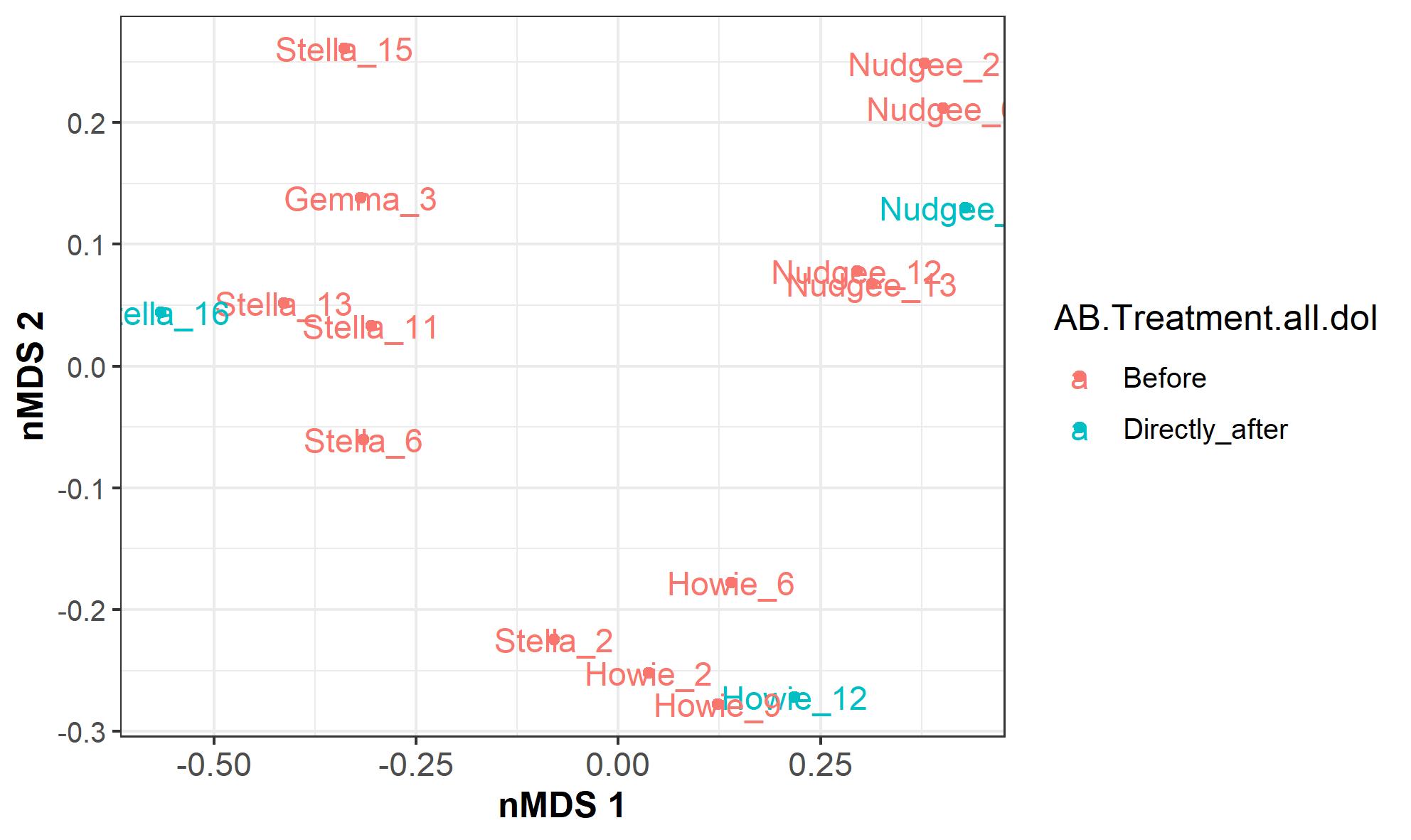


**FIG S18 nMDS plot based on Bray-Curtis dissimilarity matrix of 16 blow samples of dolphins that had received an antimicrobial treatment during the sample collection period.** The samples are coloured according to the timing of their collection relative to the timing of treatment (‘Before’: samples collected before treatment started; ‘Directly_after’: samples collected within one week after treatment). The mvabund-based analysis provided evidence for a significant difference between the ‘Before’ and the ‘Directly_after’ samples.


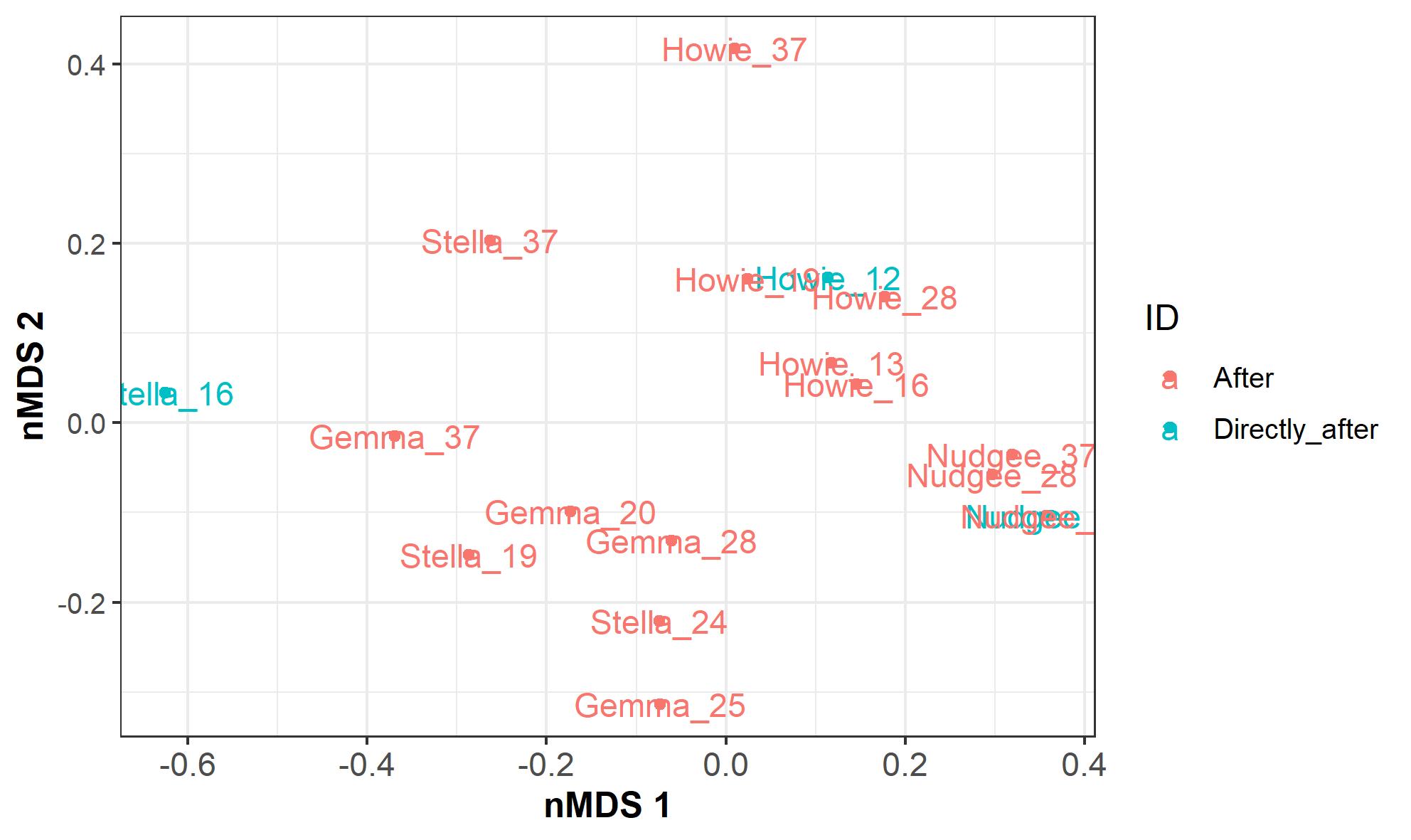


**FIG S19 nMDS plot based on Bray-Curtis dissimilarity matrix of 18 blow samples of dolphins that had received an antimicrobial treatment during the sample collection period.** The samples are coloured according to the timing of their collection relative to the timing of treatment (‘After’: samples collected at least two weeks after treatment started; ‘Directly_after’: samples collected within one week after treatment). The mvabund-based analysis provided evidence for a significant difference between the ‘After’ and the ‘Directly_after’ samples.

**
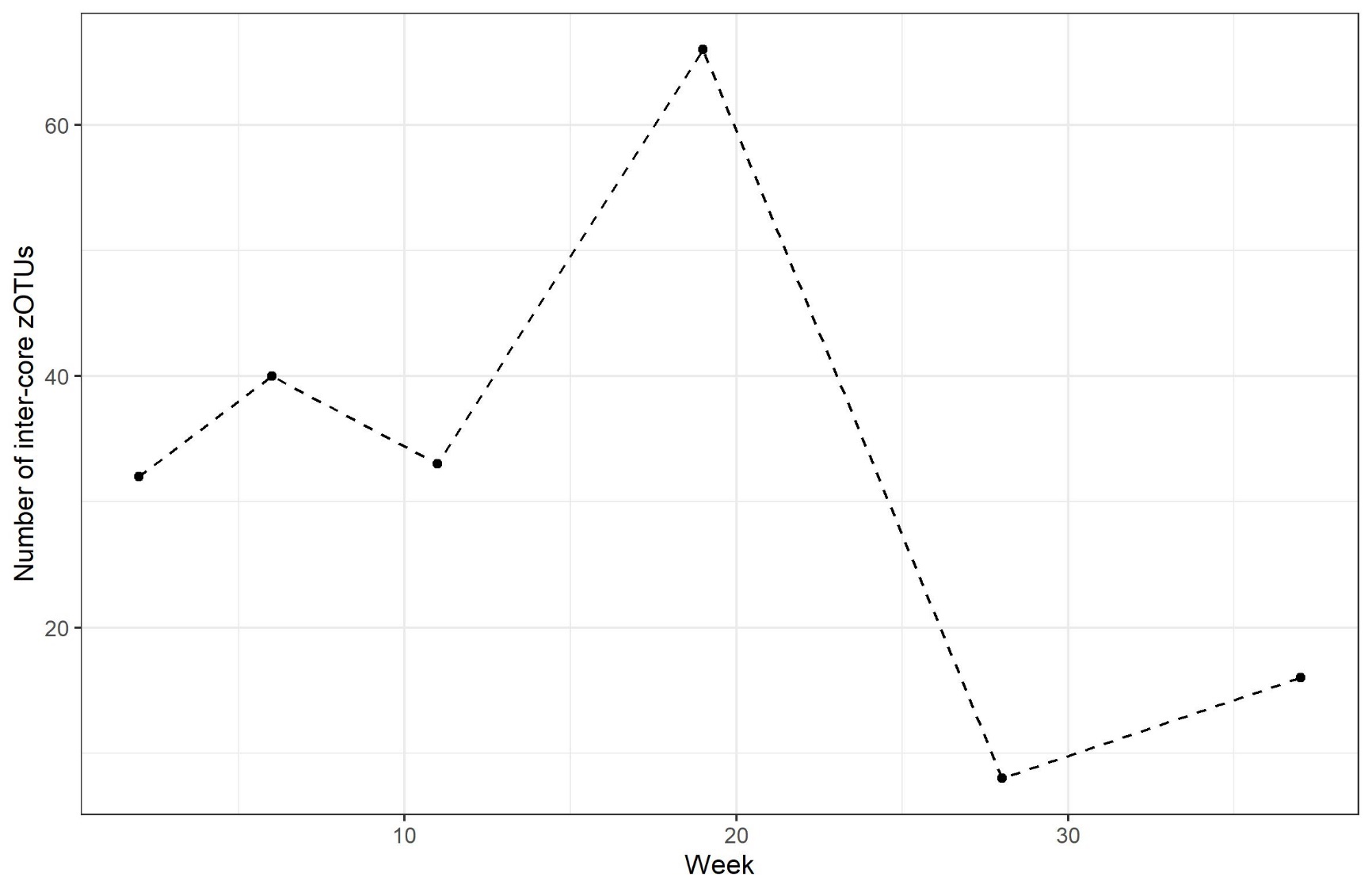
**

**FIG S20 This scatterplot shows the temporal dynamics of the inter-core in the studied dolphins from week 2 to 37.** The inter-core was defined as those zOTUs that were present across ten dolphins, randomly selected from our 13 study dolphins, at a specific time point. We picked weeks 2, 6, 11, 19, 28 and 37 as these points in time.

**Supplementary tables**

**Table S1 shows those 157 zOTUs and their taxonomy that were identified as technical contaminants and subsequently deleted from the dataset of dolphin blow microbiota.**

| No. | zOTU | Kingdom | Class | Order | Family | Genus |
| --- | --- | --- | --- | --- | --- | --- |
| 1 | Zotu10 | Bacteria | Proteobacteria | Alphaproteobacteria | *unclassified* | *unclassified* |
| 2 | Zotu100 | Bacteria | Firmicutes | Bacilli | *Bacillales* | *Planococcaceae* |
| 3 | Zotu1000 | Bacteria | Proteobacteria | Betaproteobacteria | *Hydrogenophilales* | *Hydrogenophilaceae* |
| 4 | Zotu101 | Bacteria | Firmicutes | Bacilli | *Bacillales* | *Planococcaceae* |
| 5 | Zotu1012 | Bacteria | Firmicutes | Bacilli | *Bacillales* | *Bacillaceae_1* |
| 6 | Zotu1079 | Bacteria | Firmicutes | Bacilli | *Bacillales* | *Bacillaceae_1* |
| 7 | Zotu1107 | Bacteria | Actinobacteria | Actinobacteria | *Actinomycetales* | *Corynebacteriaceae* |
| 8 | Zotu111 | Bacteria | Proteobacteria | Betaproteobacteria | *Rhodocyclales* | *Rhodocyclaceae* |
| 9 | Zotu1128 | Bacteria | Proteobacteria | Betaproteobacteria | *Burkholderiales* | *Comamonadaceae* |
| 10 | Zotu1133 | Bacteria | Firmicutes | Bacilli | *Bacillales* | *Bacillaceae_1* |
| 11 | Zotu114 | Bacteria | Firmicutes | Bacilli | *Bacillales* | *Planococcaceae* |
| 12 | Zotu1143 | Bacteria | Proteobacteria | Betaproteobacteria | *Burkholderiales* | *Comamonadaceae* |
| 13 | Zotu1148 | Bacteria | Proteobacteria | Alphaproteobacteria | *Sphingomonadales* | *Sphingomonadaceae* |
| 14 | Zotu1161 | Bacteria | Firmicutes | Bacilli | *Bacillales* | *Bacillaceae_1* |
| 15 | Zotu1180 | Bacteria | Proteobacteria | Betaproteobacteria | *unclassified* | *unclassified* |
| 16 | Zotu1206 | Bacteria | Firmicutes | Bacilli | *Bacillales* | *Bacillaceae_1* |
| 17 | Zotu1217 | Bacteria | Firmicutes | unclassified | *unclassified* | *unclassified* |
| 18 | Zotu122 | Bacteria | Proteobacteria | Alphaproteobacteria | *Caulobacterales* | *Hyphomonadaceae* |
| 19 | Zotu1229 | Bacteria | Bacteroidetes | Sphingobacteriia | *Sphingobacteriales* | *Saprospiraceae* |
| 20 | Zotu123 | Bacteria | Proteobacteria | Betaproteobacteria | *Burkholderiales* | *Burkholderiaceae* |
| 21 | Zotu1250 | Bacteria | Firmicutes | Bacilli | *Bacillales* | *Planococcaceae* |
| 22 | Zotu1268 | Bacteria | Deinococcus-Thermus | Deinococci | *Thermales* | *Thermaceae* |
| 23 | Zotu129 | Bacteria | Actinobacteria | Actinobacteria | *Actinomycetales* | *Micrococcaceae* |
| 24 | Zotu1333 | Bacteria | Firmicutes | Bacilli | *Bacillales* | *Bacillaceae_1* |
| 25 | Zotu1352 | Bacteria | Firmicutes | Bacilli | *Bacillales* | *Bacillaceae_1* |
| 26 | Zotu136 | Bacteria | Firmicutes | Bacilli | *Bacillales* | *Planococcaceae* |
| 27 | Zotu1366 | Bacteria | Firmicutes | Bacilli | *Bacillales* | *Bacillaceae_1* |
| 28 | Zotu1385 | Bacteria | Firmicutes | Bacilli | *Bacillales* | *Planococcaceae* |
| 29 | Zotu14 | Bacteria | Proteobacteria | Alphaproteobacteria | *unclassified* | *unclassified* |
| 30 | Zotu1541 | Bacteria | Proteobacteria | Alphaproteobacteria | *Rhodospirillales* | *unclassified* |
| 31 | Zotu1542 | Bacteria | Firmicutes | Bacilli | *Bacillales* | *Planococcaceae* |
| 32 | Zotu1552 | Bacteria | Firmicutes | Bacilli | *Lactobacillales* | *Leuconostocaceae* |
| 33 | Zotu163 | Bacteria | Proteobacteria | Gammaproteobacteria | *Enterobacteriales* | *Enterobacteriaceae* |
| 34 | Zotu1631 | Bacteria | Proteobacteria | Alphaproteobacteria | *Rhizobiales* | *Bradyrhizobiaceae* |
| 35 | Zotu164 | Bacteria | Proteobacteria | Gammaproteobacteria | *Enterobacteriales* | *Enterobacteriaceae* |
| 36 | Zotu1643 | Bacteria | Proteobacteria | Gammaproteobacteria | *Enterobacteriales* | *Enterobacteriaceae* |
| 37 | Zotu172 | Bacteria | Firmicutes | Bacilli | *Bacillales* | *Bacillaceae_1* |
| 38 | Zotu174 | Bacteria | Proteobacteria | Gammaproteobacteria | *Pseudomonadales* | *Moraxellaceae* |
| 39 | Zotu18 | Bacteria | Actinobacteria | Actinobacteria | *Actinomycetales* | *Propionibacteriaceae* |
| 40 | Zotu186 | Bacteria | Proteobacteria | Betaproteobacteria | *Burkholderiales* | *Comamonadaceae* |
| 41 | Zotu19 | Bacteria | Proteobacteria | Gammaproteobacteria | *Pseudomonadales* | *Pseudomonadaceae* |
| 42 | Zotu192 | Bacteria | Firmicutes | Bacilli | *Bacillales* | *Bacillaceae_1* |
| 43 | Zotu196 | Bacteria | Firmicutes | Bacilli | *Bacillales* | *Bacillaceae_1* |
| 44 | Zotu2 | Bacteria | Proteobacteria | Alphaproteobacteria | *Caulobacterales* | *Hyphomonadaceae* |
| 45 | Zotu201 | Bacteria | Proteobacteria | Alphaproteobacteria | *Caulobacterales* | *Hyphomonadaceae* |
| 46 | Zotu208 | Bacteria | Firmicutes | Bacilli | *Bacillales* | *Bacillaceae_1* |
| 47 | Zotu209 | Bacteria | Proteobacteria | Alphaproteobacteria | *unclassified* | *unclassified* |
| 48 | Zotu21 | Bacteria | Proteobacteria | Alphaproteobacteria | *Caulobacterales* | *Hyphomonadaceae* |
| 49 | Zotu2129 | Bacteria | Firmicutes | Bacilli | *Bacillales* | *Bacillaceae_1* |
| 50 | Zotu213 | Bacteria | unclassified | unclassified | *unclassified* | *unclassified* |
| 51 | Zotu215 | Bacteria | Proteobacteria | Gammaproteobacteria | *Enterobacteriales* | *Enterobacteriaceae* |
| 52 | Zotu217 | Bacteria | Proteobacteria | Alphaproteobacteria | *Caulobacterales* | *Caulobacteraceae* |
| 53 | Zotu218 | Bacteria | Proteobacteria | Gammaproteobacteria | *Oceanospirillales* | *Halomonadaceae* |
| 54 | Zotu220 | Bacteria | Proteobacteria | Gammaproteobacteria | *Pseudomonadales* | *Pseudomonadaceae* |
| 55 | Zotu222 | Bacteria | Proteobacteria | Alphaproteobacteria | *Caulobacterales* | *Caulobacteraceae* |
| 56 | Zotu2265 | Bacteria | Proteobacteria | Alphaproteobacteria | *Sphingomonadales* | *Sphingomonadaceae* |
| 57 | Zotu227 | Bacteria | Proteobacteria | Alphaproteobacteria | *Rhizobiales* | *Rhizobiaceae* |
| 58 | Zotu232 | Bacteria | Firmicutes | Bacilli | *Bacillales* | *Bacillaceae_1* |
| 59 | Zotu235 | Bacteria | Firmicutes | Bacilli | *Bacillales* | *Bacillaceae_1* |
| 60 | Zotu2353 | Bacteria | Firmicutes | Bacilli | *Bacillales* | *Planococcaceae* |
| 61 | Zotu2369 | Bacteria | Proteobacteria | Alphaproteobacteria | *unclassified* | *unclassified* |
| 62 | Zotu2408 | Bacteria | Firmicutes | Clostridia | *Clostridiales* | *Clostridiaceae_1* |
| 63 | Zotu241 | Bacteria | Proteobacteria | Gammaproteobacteria | *Enterobacteriales* | *Enterobacteriaceae* |
| 64 | Zotu246 | Bacteria | Proteobacteria | Betaproteobacteria | *Burkholderiales* | *Comamonadaceae* |
| 65 | Zotu247 | Bacteria | Firmicutes | Bacilli | *Bacillales* | *Bacillaceae_1* |
| 66 | Zotu249 | Bacteria | Proteobacteria | Gammaproteobacteria | *Oceanospirillales* | *Halomonadaceae* |
| 67 | Zotu251 | Bacteria | Proteobacteria | Gammaproteobacteria | *Enterobacteriales* | *Enterobacteriaceae* |
| 68 | Zotu252 | Bacteria | Actinobacteria | Actinobacteria | *Actinomycetales* | *unclassified* |
| 69 | Zotu2522 | Bacteria | Firmicutes | Bacilli | *Bacillales* | *Planococcaceae* |
| 70 | Zotu256 | Bacteria | Deinococcus-Thermus | Deinococci | *Thermales* | *Thermaceae* |
| 71 | Zotu262 | Bacteria | Firmicutes | Bacilli | *Bacillales* | *Bacillaceae_1* |
| 72 | Zotu264 | Bacteria | Proteobacteria | Gammaproteobacteria | *Alteromonadales* | *Pseudoalteromonadaceae* |
| 73 | Zotu265 | Bacteria | Proteobacteria | Gammaproteobacteria | *Legionellales* | *Coxiellaceae* |
| 74 | Zotu266 | Bacteria | Proteobacteria | Gammaproteobacteria | *Pseudomonadales* | *Moraxellaceae* |
| 75 | Zotu267 | Bacteria | Proteobacteria | Alphaproteobacteria | *unclassified* | *unclassified* |
| 76 | Zotu276 | Bacteria | Proteobacteria | Betaproteobacteria | *Burkholderiales* | *Burkholderiaceae* |
| 77 | Zotu285 | Bacteria | Firmicutes | Bacilli | *Bacillales* | *Bacillaceae_1* |
| 78 | Zotu286 | Bacteria | Firmicutes | Bacilli | *Bacillales* | *Bacillaceae_1* |
| 79 | Zotu289 | Bacteria | Deinococcus-Thermus | Deinococci | *Thermales* | *Thermaceae* |
| 80 | Zotu294 | Bacteria | Firmicutes | Bacilli | *Bacillales* | *Bacillaceae_1* |
| 81 | Zotu298 | Bacteria | Firmicutes | Bacilli | *Bacillales* | *Bacillaceae_1* |
| 82 | Zotu299 | Bacteria | Actinobacteria | Actinobacteria | *Rubrobacterales* | *Rubrobacteraceae* |
| 83 | Zotu30 | Bacteria | Proteobacteria | Gammaproteobacteria | *Enterobacteriales* | *Enterobacteriaceae* |
| 84 | Zotu305 | Bacteria | Actinobacteria | Actinobacteria | *Rubrobacterales* | *Rubrobacteraceae* |
| 85 | Zotu307 | Bacteria | Firmicutes | Bacilli | *Bacillales* | *Bacillaceae_1* |
| 86 | Zotu318 | Bacteria | Proteobacteria | Betaproteobacteria | *Hydrogenophilales* | *Hydrogenophilaceae* |
| 87 | Zotu321 | Bacteria | Proteobacteria | Gammaproteobacteria | *Pseudomonadales* | *Pseudomonadaceae* |
| 88 | Zotu324 | Bacteria | Firmicutes | Bacilli | *Bacillales* | *Bacillaceae_1* |
| 89 | Zotu338 | Bacteria | Proteobacteria | Alphaproteobacteria | *Caulobacterales* | *Caulobacteraceae* |
| 90 | Zotu339 | Bacteria | Proteobacteria | Betaproteobacteria | *Burkholderiales* | *Burkholderiaceae* |
| 91 | Zotu369 | Bacteria | Actinobacteria | Actinobacteria | *Actinomycetales* | *Micrococcaceae* |
| 92 | Zotu386 | Bacteria | Actinobacteria | Actinobacteria | *Actinomycetales* | *Corynebacteriaceae* |
| 93 | Zotu391 | Bacteria | Proteobacteria | Gammaproteobacteria | *Pseudomonadales* | *Pseudomonadaceae* |
| 94 | Zotu397 | Bacteria | Firmicutes | Clostridia | *Clostridiales* | *Syntrophomonadaceae* |
| 95 | Zotu398 | Bacteria | Proteobacteria | Gammaproteobacteria | *Oceanospirillales* | *Halomonadaceae* |
| 96 | Zotu404 | Bacteria | Firmicutes | Bacilli | *Bacillales* | *Planococcaceae* |
| 97 | Zotu408 | Bacteria | unclassified | unclassified | *unclassified* | *unclassified* |
| 98 | Zotu413 | Bacteria | Firmicutes | Bacilli | *Bacillales* | *Bacillaceae_1* |
| 99 | Zotu418 | Bacteria | Proteobacteria | Betaproteobacteria | *Burkholderiales* | *Burkholderiaceae* |
| 100 | Zotu426 | Bacteria | Proteobacteria | Betaproteobacteria | *Burkholderiales* | *Oxalobacteraceae* |
| 101 | Zotu433 | Bacteria | unclassified | unclassified | *unclassified* | *unclassified* |
| 102 | Zotu439 | Bacteria | Proteobacteria | Gammaproteobacteria | *Pseudomonadales* | *Pseudomonadaceae* |
| 103 | Zotu449 | Bacteria | Firmicutes | Bacilli | *Bacillales* | *Planococcaceae* |
| 104 | Zotu486 | Bacteria | Proteobacteria | Gammaproteobacteria | *Pseudomonadales* | *Pseudomonadaceae* |
| 105 | Zotu488 | Bacteria | Chloroflexi | Chloroflexia | *Kallotenuales* | *Kallotenuaceae* |
| 106 | Zotu50 | Bacteria | Proteobacteria | Gammaproteobacteria | *Enterobacteriales* | *Enterobacteriaceae* |
| 107 | Zotu503 | Bacteria | Bacteroidetes | Sphingobacteriia | *Sphingobacteriales* | *Chitinophagaceae* |
| 108 | Zotu518 | Bacteria | Deinococcus-Thermus | Deinococci | *Thermales* | *Thermaceae* |
| 109 | Zotu52 | Bacteria | Proteobacteria | Betaproteobacteria | *Burkholderiales* | *Burkholderiaceae* |
| 110 | Zotu531 | Bacteria | Deinococcus-Thermus | Deinococci | *Thermales* | *Thermaceae* |
| 111 | Zotu546 | Bacteria | Proteobacteria | Betaproteobacteria | *Burkholderiales* | *Burkholderiaceae* |
| 112 | Zotu549 | Bacteria | Firmicutes | Bacilli | *Bacillales* | *Bacillaceae_1* |
| 113 | Zotu553 | Bacteria | Firmicutes | Bacilli | *Bacillales* | *Bacillaceae_1* |
| 114 | Zotu558 | Bacteria | Deinococcus-Thermus | Deinococci | *Thermales* | *Thermaceae* |
| 115 | Zotu561 | Bacteria | Firmicutes | Bacilli | *Bacillales* | *Bacillaceae_1* |
| 116 | Zotu562 | Bacteria | Firmicutes | Bacilli | *Bacillales* | *Bacillaceae_1* |
| 117 | Zotu564 | Bacteria | Proteobacteria | Gammaproteobacteria | *Pseudomonadales* | *Pseudomonadaceae* |
| 118 | Zotu569 | Bacteria | Firmicutes | Bacilli | *Bacillales* | *Bacillaceae_1* |
| 119 | Zotu579 | Bacteria | Actinobacteria | Actinobacteria | *Actinomycetales* | *Micrococcaceae* |
| 120 | Zotu607 | Bacteria | Firmicutes | Bacilli | *Bacillales* | *Bacillaceae_1* |
| 121 | Zotu612 | Bacteria | Firmicutes | Clostridia | *Clostridiales* | *Syntrophomonadaceae* |
| 122 | Zotu619 | Bacteria | Proteobacteria | Alphaproteobacteria | *Rhizobiales* | *Bradyrhizobiaceae* |
| 123 | Zotu624 | Bacteria | Actinobacteria | Actinobacteria | *Actinomycetales* | *Mycobacteriaceae* |
| 124 | Zotu63 | Bacteria | Firmicutes | Bacilli | *Bacillales* | *Bacillaceae_1* |
| 125 | Zotu653 | Bacteria | Actinobacteria | Actinobacteria | *Actinomycetales* | *unclassified* |
| 126 | Zotu659 | Bacteria | Proteobacteria | Gammaproteobacteria | *Alteromonadales* | *Pseudoalteromonadaceae* |
| 127 | Zotu66 | Bacteria | Proteobacteria | Betaproteobacteria | *Burkholderiales* | *Oxalobacteraceae* |
| 128 | Zotu660 | Bacteria | Actinobacteria | Actinobacteria | *Actinomycetales* | *Microbacteriaceae* |
| 129 | Zotu665 | Bacteria | Bacteroidetes | unclassified | *unclassified* | *unclassified* |
| 130 | Zotu667 | Bacteria | Firmicutes | Bacilli | *Bacillales* | *Bacillaceae_1* |
| 131 | Zotu68 | Bacteria | Firmicutes | Bacilli | *Bacillales* | *Planococcaceae* |
| 132 | Zotu686 | Bacteria | Firmicutes | Bacilli | *Bacillales* | *Planococcaceae* |
| 133 | Zotu687 | Bacteria | Firmicutes | Bacilli | *Bacillales* | *Planococcaceae* |
| 134 | Zotu696 | Bacteria | Proteobacteria | Gammaproteobacteria | *Enterobacteriales* | *Enterobacteriaceae* |
| 135 | Zotu718 | Bacteria | Proteobacteria | Alphaproteobacteria | *Rhizobiales* | *Rhizobiaceae* |
| 136 | Zotu722 | Bacteria | Proteobacteria | unclassified | *unclassified* | *unclassified* |
| 137 | Zotu728 | Bacteria | Firmicutes | Bacilli | *Bacillales* | *Planococcaceae* |
| 138 | Zotu756 | Bacteria | Firmicutes | Bacilli | *Bacillales* | *Bacillaceae_1* |
| 139 | Zotu769 | Bacteria | Firmicutes | Bacilli | *Bacillales* | *unclassified* |
| 140 | Zotu817 | Bacteria | Firmicutes | Bacilli | *Bacillales* | *Bacillaceae_1* |
| 141 | Zotu818 | Bacteria | Proteobacteria | Betaproteobacteria | *Hydrogenophilales* | *Hydrogenophilaceae* |
| 142 | Zotu828 | Bacteria | Firmicutes | Bacilli | *Bacillales* | *Bacillaceae_1* |
| 143 | Zotu83 | Bacteria | Firmicutes | Bacilli | *Bacillales* | *Planococcaceae* |
| 144 | Zotu845 | Bacteria | Firmicutes | Bacilli | *Bacillales* | *Bacillaceae_1* |
| 145 | Zotu868 | Bacteria | Firmicutes | Bacilli | *Bacillales* | *Planococcaceae* |
| 146 | Zotu90 | Bacteria | Firmicutes | Bacilli | *Bacillales* | *Bacillaceae_1* |
| 147 | Zotu91 | Bacteria | Firmicutes | Bacilli | *Bacillales* | *Planococcaceae* |
| 148 | Zotu919 | Bacteria | Firmicutes | Bacilli | *Bacillales* | *Bacillaceae_1* |
| 149 | Zotu927 | Bacteria | Proteobacteria | Alphaproteobacteria | *Rhizobiales* | *Phyllobacteriaceae* |
| 150 | Zotu94 | Bacteria | Firmicutes | Bacilli | *Bacillales* | *Planococcaceae* |
| 151 | Zotu944 | Bacteria | Firmicutes | Bacilli | *Bacillales* | *Bacillaceae_1* |
| 152 | Zotu957 | Bacteria | Firmicutes | Bacilli | *Bacillales* | *Sporolactobacillaceae* |
| 153 | Zotu96 | Bacteria | Proteobacteria | Gammaproteobacteria | *Pseudomonadales* | *Moraxellaceae* |
| 154 | Zotu97 | Bacteria | Cyanobacteria/Chloroplast | Cyanobacteria | *Family_VIII* | *GpVIII* |
| 155 | Zotu977 | Bacteria | Proteobacteria | Alphaproteobacteria | *unclassified* | *unclassified* |
| 156 | Zotu991 | Bacteria | Proteobacteria | Betaproteobacteria | *Hydrogenophilales* | *Hydrogenophilaceae* |
| 157 | Zotu994 | Bacteria | Parcubacteria | unclassified | *unclassified* | *unclassified* |

**Table S2 Alpha diversity parameters of dolphin blow microbiota for each time point over the sampling period of 37 weeks: richness, diversity, Chao1 and ACE species estimators.**

| **Dolphin ID** | **Week** | **Richness** | **Diversity** | **Chao1** | **ACE** |
| --- | --- | --- | --- | --- | --- |
| Coen | 2 | 290 | 3.57 | 317 | 299.92 |
| Coen | 6 | 302 | 4.16 | 419 | 378.97 |
| Coen | 11 | 326 | 3.52 | 330 | 315.33 |
| Coen | 19 | 293 | 4.02 | 365 | 321.04 |
| Coen | 28 | 356 | 3.18 | 345 | 359.27 |
| Evie | 2 | 342 | 4.26 | 385 | 394.27 |
| Evie | 6 | 221 | 4.52 | 426 | 449.38 |
| Evie | 11 | 416 | 4.55 | 479 | 436.68 |
| Evie | 19 | 427 | 4.21 | 460 | 242.75 |
| Evie | 28 | 378 | 2.76 | 295 | 402.33 |
| Evie | 37 | 382 | 4.44 | 454 | 270.08 |
| Gemma | 3 | 239 | 2.69 | 288 | 337.58 |
| Gemma | 20 | 312 | 4.24 | 361 | 75.8 |
| Gemma | 25 | 64 | 2.89 | 97 | 314.75 |
| Gemma | 28 | 323 | 4.49 | 326 | 105.07 |
| Gemma | 37 | 90 | 3.06 | 135 | 517.1 |
| Howie | 2 | 495 | 4.79 | 538 | 505.55 |
| Howie | 6 | 405 | 4.81 | 543 | 417.14 |
| Howie | 9 | 431 | 4.25 | 441 | 374.96 |
| Howie | 12 | 414 | 3.98 | 414 | 418.36 |
| Howie | 13 | 391 | 4.41 | 443 | 451.69 |
| Howie | 16 | 362 | 4.53 | 470 | 450.91 |
| Howie | 19 | 255 | 3.97 | 483 | 442.82 |
| Howie | 28 | 408 | 4.20 | 508 | 280.18 |
| Kiama | 6 | 395 | 3.73 | 273 | 375.17 |
| Kiama | 11 | 335 | 4.30 | 413 | 359.24 |
| Kiama | 19 | 357 | 3.80 | 408 | 406.74 |
| Kiama | 28 | 332 | 4.59 | 435 | 355.69 |
| Kiama | 37 | 257 | 3.79 | 371 | 394.03 |
| Moki | 2 | 381 | 4.83 | 413 | 372.31 |
| Moki | 6 | 56 | 3.90 | 392 | 393.14 |
| Moki | 11 | 213 | 3.88 | 430 | 231.36 |
| Moki | 19 | 367 | 3.26 | 302 | 59.17 |
| Moki | 28 | 325 | 2.44 | 63 | 332.53 |
| Moki | 37 | 348 | 3.67 | 373 | 146.87 |
| Nudgee | 2 | 141 | 3.34 | 173 | 201.57 |
| Nudgee | 6 | 226 | 3.22 | 216 | 273.05 |
| Nudgee | 12 | 253 | 3.83 | 328 | 267.21 |
| Nudgee | 13 | 138 | 3.90 | 321 | 207.19 |
| Nudgee | 20 | 188 | 2.85 | 241 | 145.56 |
| Nudgee | 21 | 200 | 2.69 | 163 | 230.38 |
| Nudgee | 28 | 194 | 4.01 | 243 | 207.2 |
| Nudgee | 37 | 245 | 3.69 | 226 | 309.65 |
| RB | 2 | 297 | 3.89 | 327 | 246.55 |
| RB | 6 | 346 | 3.27 | 274 | 340.41 |
| RB | 11 | 339 | 4.30 | 364 | 357.49 |
| RB | 19 | 320 | 4.32 | 379 | 359.98 |
| RB | 28 | 358 | 4.27 | 385 | 369.09 |
| RB | 37 | 227 | 4.50 | 375 | 397.37 |
| Scooter | 2 | 384 | 4.85 | 420 | 313.09 |
| Scooter | 6 | 95 | 3.69 | 370 | 154.96 |
| Scooter | 11 | 389 | 4.17 | 160 | 397.67 |
| Scooter | 19 | 149 | 4.53 | 438 | 113.75 |
| Scooter | 28 | 295 | 3.51 | 137 | 328.34 |
| Sirius | 2 | 319 | 4.43 | 350 | 362.69 |
| Sirius | 6 | 96 | 4.35 | 383 | 213.25 |
| Sirius | 11 | 245 | 3.66 | 256 | 250.32 |
| Sirius | 19 | 200 | 4.36 | 274 | 103.49 |
| Sirius | 28 | 134 | 2.49 | 115 | 145.05 |
| Sirius | 37 | 350 | 3.59 | 173 | 131.35 |
| Squeak | 2 | 107 | 3.73 | 136 | 354.38 |
| Squeak | 6 | 382 | 3.71 | 400 | 100.63 |
| Squeak | 11 | 422 | 3.68 | 108 | 434.57 |
| Squeak | 19 | 99 | 5.03 | 454 | 399.76 |
| Squeak | 28 | 340 | 4.44 | 407 | 415.51 |
| Starbuck | 2 | 404 | 4.43 | 439 | 373.89 |
| Starbuck | 6 | 429 | 4.06 | 403 | 423.09 |
| Starbuck | 11 | 363 | 4.02 | 472 | 396.66 |
| Starbuck | 19 | 408 | 3.70 | 425 | 443.13 |
| Starbuck | 28 | 371 | 4.51 | 483 | 383.9 |
| Starbuck | 37 | 372 | 4.31 | 409 | 378.24 |
| Stella | 2 | 373 | 4.64 | 390 | 294.79 |
| Stella | 6 | 188 | 2.97 | 341 | 380.33 |
| Stella | 11 | 68 | 3.37 | 424 | 245.13 |
| Stella | 13 | 216 | 2.32 | 301 | 132.23 |
| Stella | 15 | 349 | 2.22 | 164 | 73.08 |
| Stella | 16 | 183 | 2.20 | 92 | 209.04 |
| Stella | 19 | 124 | 3.28 | 252 | 191.59 |
| Stella | 24 | 249 | 4.19 | 208 | 257.13 |
| Stella | 37 | 268 | 2.78 | 280 | 299.92 |

**Other supplementary material**

**S1 R code of statistical data analysis**: S1_*RCode_Dolphin.blow.manuscript.nb*
